# Proteome-wide Mendelian randomization of Adverse Outcomes in Human Heart Failure

**DOI:** 10.1161/JAHA.123.031154

**Published:** 2024-02-29

**Authors:** Marie-Joe Dib, Michael Levin, Lei Zhao, Zhaoqing Wang, Christina Ebert, Oday Salman, Joe D. Azzo, Sushrima Gan, Payman Zamani, Jordana B. Cohen, Dipender Gill, Stephen Burgess, Loukas Zagkos, Vanessa van Empel, A. Mark Richards, Rob Doughty, Ali Javaheri, Douglas L. Mann, Ernst Rietzschell, Karl Kammerhoff, Joseph Gogain, Peter Schafer, Dietmar A. Seiffert, Francisco Ramirez-Valle, David A. Gordon, Thomas P. Cappola, Julio A. Chirinos

**Affiliations:** 1 Division of Cardiovascular Medicine, Hospital of the University of Pennsylvania, Philadelphia PA; 2 Bristol-Myers Squibb Company, Lawrenceville, NJ; 3 University of Pennsylvania Perelman School of Medicine, Philadelphia, PA; 4 Renal-Electrolyte and Hypertension Division, Perelman School of Medicine, University of Pennsylvania, Philadelphia PA; 5 Center for Clinical Epidemiology and Biostatistics, Perelman School of Medicine, University of Pennsylvania, Philadelphia, Pennsylvania; 6 Chief Scientific Advisor Office, Research and Early Development, Novo Nordisk, Copenhagen, Denmark; 7 Department of Epidemiology and Biostatistics, School of Public Health, Imperial College London, UK; 8 MRC Integrative Epidemiology Unit, University of Bristol, Bristol, UK; 9 Department of Public Health and Primary Care, University of Cambridge, Cambridge, UK; 10 Department of Cardiology, Maastricht University Medical Center, Maastricht, The Netherlands; 11 Cardiovascular Research Institute, National University of Singapore, Singapore; 12 Christchurch Heart Institute, University of Otago, Christchurch, New Zealand; 13 Washington University School of Medicine, St. Louis, MO; 14 Department of Cardiovascular Diseases, Ghent University Hospital, Ghent, Belgium; 15 SomaLogic, Inc., Boulder, CO, USA

**Keywords:** heart failure, HFrEF, Mendelian randomization, proteomics

## Abstract

**Background:**

Identifying novel molecular drivers of disease progression in heart failure (HF) is a high-priority goal that may provide new therapeutic targets to improve patient outcomes.

**Objectives:**

We aimed to assess the relationship between plasma proteins and adverse outcomes in HF and assess the causal role of various proteins using Mendelian randomization (MR).

**Methods:**

We measured ~5,000 plasma proteins (SomaScan assay) among 1,964 participants with HF with a reduced left ventricular ejection fraction enrolled in the Penn HF study (PHFS). We assessed the observational relationship between plasma proteins and: 1) all-cause death; 2) death or HF-related hospital admission (DHFA). Proteins significantly associated with outcomes were the subject of two-sample Mendelian randomization (MR) and colocalization analyses using blood protein quantitative trait loci (pQTL) from the deCODE and Fenland studies to test the putative causal effects of these proteins on HF outcomes.

**Results:**

After correction for multiple comparisons, we found 243 and 126 proteins significantly associated with death and DHFA, respectively. Mendelian randomization and colocalization analyses provided converging evidence of potentially causal effects of 6 proteins (CCDC126, CD55, CCL14, NEGR1, SVEP1 and ADH7) on DHFA and 11 proteins on death (RSPO4, FCN2, IGLL1, HPGDS, FGF23, EFEMP1, STC1, ATOX1, FCN2, SVEP1, ANG) at nominal significance (*P* < 0.05).

**Conclusions:**

Our study implicates multiple novel proteins in HF and provides preliminary evidence of a potentially causal association between plasma levels of 17 circulating plasma proteins the risk for adverse outcomes in human HF. Whether these proteins represent suitable therapeutic targets should be the focus of future studies.

## Introduction

Heart failure (HF) is a highly prevalent condition associated with significant morbidity and mortality. HF currently affects >6 million individuals in the United States, and the prevalence continues to increase (1,2). Although significant advances have been made in the treatment of heart failure with reduced ejection fraction (HFrEF), patients with all forms of HF remain at substantial residual risk of progression and adverse outcomes despite treatment with available guideline-directed medical therapy (3). Therefore, the identification of new molecular therapeutic targets is needed. While preclinical models are valuable tools to evaluate candidate disease mechanisms and therapeutic targets, identifying relevant targets in human HF is highly desirable.

Plasma biomarkers may offer insights into mechanisms related to adverse outcomes in HF. Historically, candidate biomarkers such as natriuretic peptides and troponin have been investigated as markers of adverse outcomes in human HF (4,5). More recently, technological advances have enabled the assessment of thousands of circulating proteins using aptamer-based approaches (6–11). Early versions of these proteomics panels have been applied in observational studies designed to evaluate limited sets of proteins in relatively small cohorts with heart failure, identifying candidate biomarkers that may be putative therapeutic targets or indicators of prognosis (12–14). These methods have also been applied among larger population cohorts, pairing plasma proteomics with genome-wide genotyping among studies including tens of thousands of participants (15–17). These larger studies have provided important insights into the genetic regulation of the plasma proteome, providing a foundation for evaluating the causal effects of plasma proteins on the development and progression of various conditions. However, studies assessing the putative causal relationship between the plasma proteome and the risk of adverse outcomes in human HF are not available.

Traditional observational study designs may be limited by residual confounding and reverse causality, limiting the ability to estimate the causal effects of exposures on outcomes of interest. Mendelian randomization (MR) is a technique which enables the estimation of causal effects in observational analyses. MR leverages the naturally randomized allocation of genetic variants among the population as instrumental variables to mimic a randomized controlled trial. Under certain assumptions, this approach estimates the casual effects of exposures on outcomes (18). Here, we sought to evaluate the role of plasma proteins on the risk of adverse outcomes in HF through complementary epidemiologic and MR approaches.

## Methods

### Study population

We studied 2,248 participants from the Penn Heart Failure Study (PHFS), a prospective cohort study of HF patients recruited at the University of Pennsylvania (Philadelphia, PA), Case Western Reserve University (Cleveland, OH), and the University of Wisconsin (Madison, WI) between 2003-2011. Patients with a clinical diagnosis of HF as determined by a HF specialist were enrolled. At the time of enrolment, standardized questionnaires were administered to participants and their physicians to obtain detailed clinical data as described previously (19,20). Participants with expected mortality of 6 months or less from a non-cardiac condition, as judged by their treating physician, mechanical circulatory support, or inability to provide informed consent were excluded. Participants provided written informed consent.

### Plasma proteomics measurements

We utilized the SomaScan® assay version 4, which is a multiplexed, modified aptamer-based binding-assay. This assay includes 4,979 modified aptamer reagents to 4,776 unique protein targets. The SomaScan assay utilizes Slow-Off-rate Modified Aptamer (SOMAmer) reagents, which are chemically modified nucleotides, to bind and quantify target proteins in relative fluorescent units directly proportional to the amount of target protein in the sample.

### Association between plasma proteins and outcomes

We assessed the associations between the levels of plasma proteins measured in the SomaScan assay and: 1) all-cause death; 2) death or HF-related hospital admission (DHFA). Associations between adverse outcomes and individual proteins that were significant, with a corrected *P*<0.05, were then taken forward to further analyses using genomic data.

### Genotype data and genetic association analyses

PHFS participants were genotyped with the Illumina OminExpress+Exome (n=1334) or Illumina Infinium Multi-Ethnic Global-8 v1.0 (n=880) arrays (total n=2214).(21) Imputation was performed separately for the two arrays, using the Michigan Imputation Server (1000 Genomes Phase 3 Version 5 (hg19)) as the reference panel with the Minimac4 pipeline.(22) We assessed the association between genetic variants and outcomes. Details on these genetic association analyses can be found in **Supplementary Methods**.

### Collider Bias Adjustment

Our genetic association analyses with death and DHFA involved outcomes in the context of established HF, and were thereby conditional upon HF liability, potentially introducing collider bias in subsequent analyses. We used the Slope-Hunter method (23) to adjust for potential collider bias using the SlopeHunter R package. We used genome-wide summary statistics from the HEart failure Molecular Epidemiology for Therapeutic targetS (HERMES) Consortium (24) to correct for associations with the risk of developing HF. Using a cluster-based method, Slope-Hunter categorizes independent genetic variants associated with the risk of HF into (1) variants affecting HF incidence only, and (2) variants affecting both HF incidence and adverse outcomes in the context of established HF. A correction factor is then estimated through the slope of the regression of adverse outcomes on HF risk associations for a subset of variants, and is then applied to the full set variants under the assumption that variant-HF risk and variant-HF outcome associations are linear with no interactions.

### Mendelian randomization

#### Causal inference using two-sample MR:

**Figure 1:**
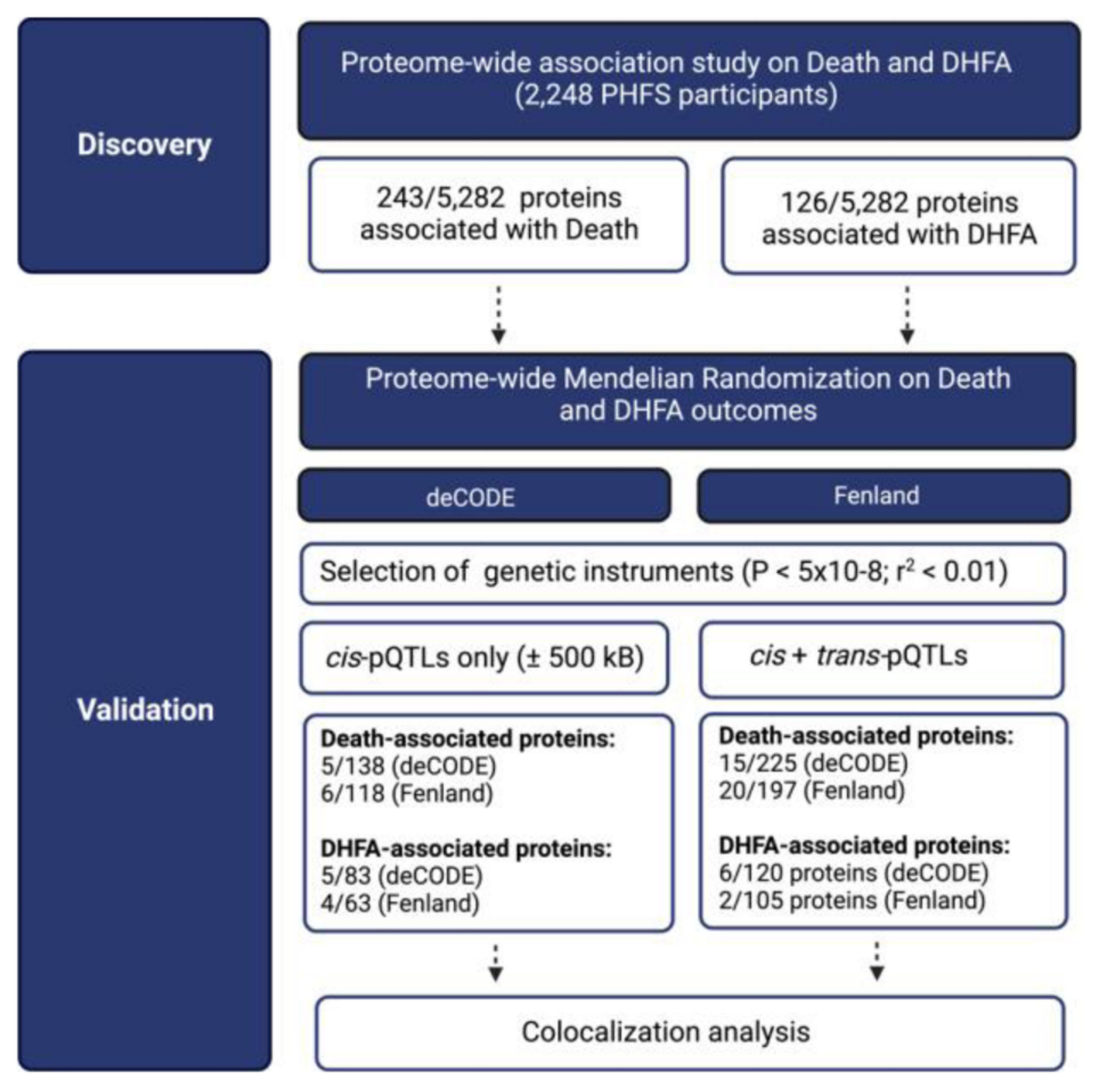
Proteome-wide association study of 5,282 proteins for death and DHFA outcomes: a Mendelian randomization (MR) framework.

We conducted MR analyses to estimate the effect of genetically-predicted plasma protein levels on the collider bias-adjusted risk of all-cause mortality and DHFA. MR is a statistical approach which uses genetic variants as instrumental variables to proxy the causal effects of an exposure on an outcome of interest. MR has three key instrumental variable assumptions: 1) relevance, 2) independence, and 3) exclusion restriction. Using proteomics data from the Icelandic deCODE and Fenland studies, we first identified *cis* (within ± 500 KB of the protein-encoding region) and *trans* (outside of the 500KB window) - acting protein quantitative trail loci (pQTLs) for circulating protein levels of each of the identified proteins in the discovery phase (Figure 1). Proteins were analysed if pQTLs with association *p* < 5 x 10^-8^ were available. Our primary analysis was restricted to *cis*-pQTLs as they are more likely to have protein-specific effects than *trans*-pQTLs (25). We then broadened our analysis to include *trans*-pQTLs as genetic instruments. Further details on the selection of genetic instruments and MR sensitivity measures can be found in the **Supplementary Methods** section. Since in this analysis, we only assessed candidate proteins that demonstrated a significant association with outcomes in our proteome-wide association study, we report nominally significant results (P < 0.05), along with estimated Odds ratios (OR) and 95% confidence intervals (CI) for the causal effect of these proteins.

### Genetic colocalization analyses

Upon identifying MR evidence of a causal effect, we conducted sensitivity colocalization analyses to confirm that the proteins with a putative causal effect on Death and DHFA (as indicated by MR) were regulated by the same causal variant (26). The colocalization method calculates posterior probabilities (PP) for the competing hypotheses of H_0_ (no causal variants, H_1_ (causal variant for trait 1, the protein level in our case), H_2_ (causal variant for trait 2, the HF outcomes in our case), H_3_ (distinct causal variants for traits 1 and 2), and, H_4_ (shared causal variants for traits 1 and 2, which supports a causal association). The prior probability that a SNP is causal to one trait in a region was set to 10^-4^. If the PP that one SNP is shared for traits 1 and 2 was greater than 0.8, we regarded it as a colocalization signal, supporting a causal effect of the protein on the outcome. Conditional colocalization (PP_H4_/[PP_H4_ + PP_H3_]) was performed to evaluate the evidence supporting a shared causal variant, conditional on the presence of causal variants for each trait in the region. We also conducted sensitivity analyses, described in **Supplementary Methods** to consider whether conclusions were robust.

### Statistical Analyses

Continuous data are shown as mean ± standard deviation (SD) for normally distributed variables and compared using the nonpaired Student’s *t*-test. Non-normally distributed continuous variables are presented as median (interquartile range) and were analyzed using the Kruskal-Wallis test. Categorical variables are shown as counts (percentages) and analyzed using the chi-square test or Fisher’s exact test, as appropriate.

We assessed the relationship between individual biomarkers and the risk of death or DHFA using Cox proportional hazards regression. Hazard ratios (HRs) for all proteins are standardized (expressed per SD increase, or 1-point increase in the *z* score). We corrected the alpha error for multiple comparisons based on the principal components underlying the variability of all measured proteins, as previously described (11,27–29). We built unadjusted Cox models and models adjusted for the Meta-Analysis Global Group in Chronic Heart Failure (MAGGIC) risk score (30) plus NT-proBNP. The MAGGIC risk score is composed on 13 clinical variables (age, sex, body mass index, systolic blood pressure, LV EF, serum creatinine, current smoking, diabetes mellitus, chronic obstructive pulmonary disease, New York Heart Association class, HF duration >18 months, β-blocker use, and angiotensin-converting enzyme inhibitor use). Since we did not have information regarding COPD and the time of HF duration in this cohort, we assigned a value of zero to all participants for these 2 factors when computing the MAGGIC risk score.

Statistical significance was defined as a corrected 2-tailed *p* value < 0.05. Analyses were performed using the Matlab statistics and ML toolbox (Matlab 2022a, the Mathworks, Natick, Massachusetts).

Genomic analyses were performed using R version 4.0.3. We used the **MendelianRandomization** and **TwoSampleMR** packages in R to perform all MR analyses. Colocalization was performed using **Coloc** (version 3.2-1). Collider bias adjustment was performed on **SlopeHunter** (version 0.0.2). Volcano plots were generated using **ggrepel** and **EnhancedVolcano** on R.

## Results

Baseline characteristics of PHFS participants are shown in Table 1. Out of 2,248 participants with SomaScan data at baseline, 2,234 had follow-up data. Median follow up was 1,485 days for death and 917 days for DHFA. During follow-up, 575 participants died and 1,167 participants reached the composite outcome of DHFA.

### Association between measured protein levels and the risk of DHFA: Survival analysis

We found 1,475 proteins to be significantly associated with the risk of DHFA. The hazard ratios and P values are included in Supplementary Table 1. A volcano plot showing the relationship between baseline plasma protein levels and the risk of incident DHFA is shown in Supplementary Figure 1A.

The top proteins positively associated with the risk of DHFA included natriuretic peptide B (NPPB; Standardized [Std] HR=1.80; *P*<0.0001), thrombospondin 2 (THBS2; Std HR=1.74; *P*<0.0001), A disintegrin and metalloproteinase with thrombospondin motifs-like protein (ADAMTSL; Std HR=1.72, *P*<0.0001), sushi, von Willebrand factor type A, EGF and pentraxin domain containing 1 (SVEP1; Std HR=1.63, *P*<0.0001), chemokine (C-C motif) ligand 14 (CCL14; Std HR=1.61, *P*<0.0001), follistatin-related protein 3 (FSTL3; Std HR=1.62, *P*<0.0001), insulin like growth factor binding protein 7 (IGFBP7; Std HR=1.65, *P*<0.0001), angiopoietin 2 (ANGPT2; 1.61, *P*<0.0001), growth differentiation factor 15 (GDF15; Std HR=1.63, P<0.0001), and spondin 1 (SPON1; Std HR=1.62, *P*<0.0001). The top proteins that were negatively associated with the risk of DHFA included Cartilage intermediate layer protein 2 (CILP2; Std HR=0.63, *P*<0.0001), vitamin K-dependent protein C (PROC; Std HR=0.68, *P*<0.0001), ficolin-3 (FCN3; Std HR 0.71, *P*<0.0001), butyrylcholinesterase (BCHE; Std HR=0.68, *P*<0.0001), coagulation factor VII (F7; Std HR=0.70, P<0.0001), cadherin 3 (CDH3; Std HR=0.69, *P*<0.0001), SET (Std HR=0.70, *P*<0.0001), C-Type Lectin Domain Family 3 Member B (CLEC3B; Std HR=0.70, *P*<0.0001), plasminogen (PLG; Std HR=0.70, *P*<0.0001), and hematopoietic prostaglandin-D synthase (HPGD; Std HR=0.72, *P*<0.0001).

**Figure 2:**
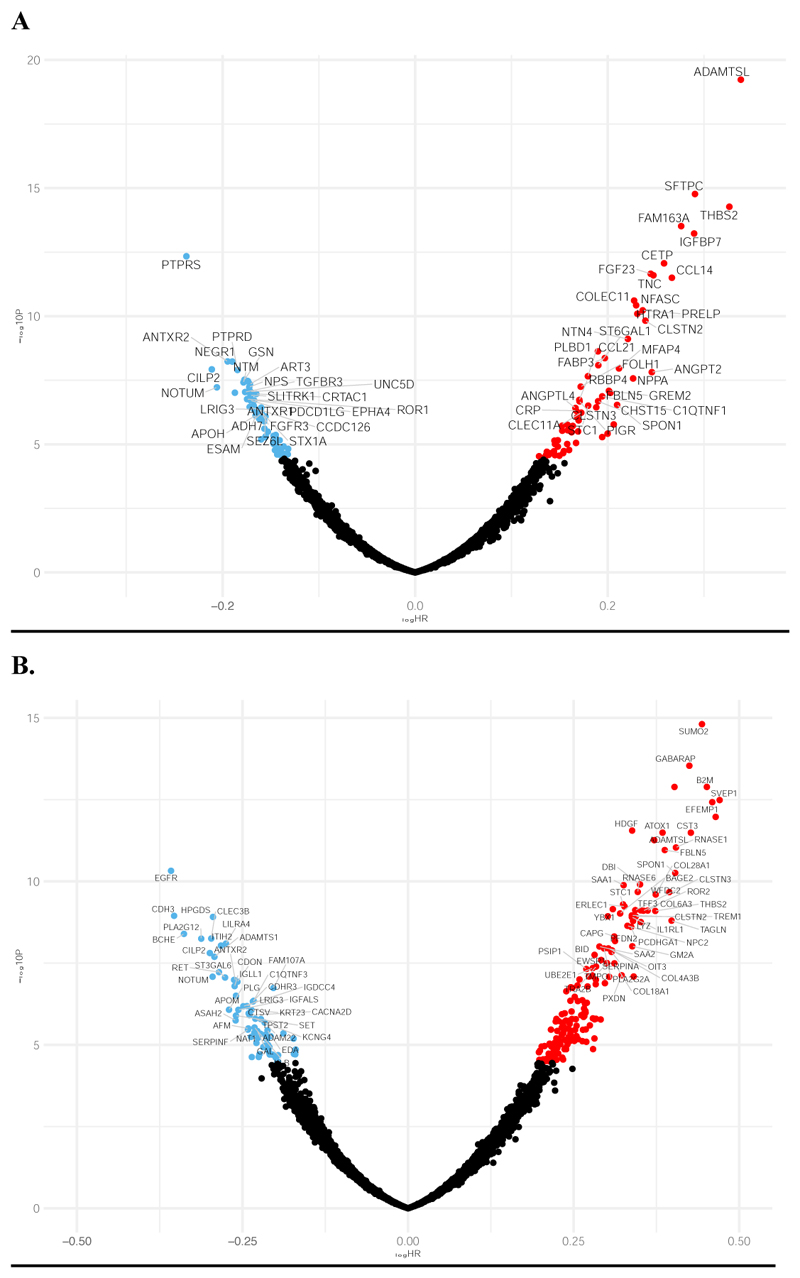
Volcano plots demonstrating associations between all plasma proteins measured in PHFS proteins and (A) death and heart failure related hospital admission (DHFA) and (B) all-cause mortality in the adjusted cox model. This plot shows standardized HR against the log-10 *p* value, to better visualize the importance of each biomarker in order of significance. The nominal significance level and the alpha-corrected significance level are represented by dashed lines on the y axis. Only the top 100 proteins positively or negatively associated with death are labelled. Adjusted models include 1,889 participants who had sufficient data for computation of the baseline MAGGIC risk score and had follow-up data.

In analyses adjusted for the MAGGIC risk score and NT-proBNP, we found 126 proteins to be significantly associated with the risk of DHFA. The hazard ratios and P values are included in Supplementary Table 2. A volcano plot showing the relationship between baseline plasma protein levels and the risk of incident DHFA is shown in Figure 2A. The top proteins positively associated with the risk of DHFA included ADAMTSL (Std HR=1.40, *P*<0.0001), surfactant protein C (SFTPC; Std HR=1.33, *P*<0.0001), thrombospondin 2 (THBS2; Std HR=1.38, P<0.0001), FAM163A (Std HR=1.32, P<0.0001), IGFBP7 (Std HR=1.33, P<0.0001), cholesteryl ester transfer protein (CETP; Std HR=1.29, P=1.29x10-9), FGF23 (Std HR=1.27, P<0.0001), tenascin C (TNC; Std HR=1.28, P<0.0001), CCL14 (Std HR=1.30, *P*<0.0001), and Collectin Subfamily Member 11 (COLEC11; Std HR=1.25, *P*<0.0001). The top proteins that were negatively associated with the risk of DHFA include protein tyrosine phosphatase receptor type S (PTPRS; Std HR=0.78, *P*<0.0001), anthrax toxin receptor 2 (ANTXR2; Std HR=0.82, *P*<0.0001), neuronal growth regulator 1 (NEGR1; Std HR=0.82; *P*=8.80x10^-6^), CILP2 (Std HR=0.81, *P*<0.0001), protein tyrosine phosphatase receptor type D (PTPRD; Std HR=0.83, *P*<0.0001), gelsolin (GSN; Std HR=0.84, *P*<0.0001), neurotrimin (NTM; Std HR= 0.83, *P*<0.0001), ecto-ADP-ribosyltransferase-3 (ART3; Std HR=0.84, *P*<0.0001), neuropeptide S (NPS; Std HR=0.84, *P*<0.0001) and Notum, palmitoleoyl-protein carboxylesterase (NOTUM; Std HR=0.81, *P*<0.0001). Table 2 shows standardized HRs and P values for the top proteins positively and negatively associated with the risk of DHFA.

### Mendelian Randomization analyses: genetically-predicted protein levels and the risk of DHFA

Among the 126 proteins associated with DHFA in the adjusted analyses above, we found 83 proteins in the deCODE study and 63 proteins in the Fenland study with available *cis*-pQTLs as genetic instruments (Figure 1). We report the mean F statistics for each genetically-instrumented protein in Supplementary Tables 5-12. The F statistic for all identified genetic instruments was >10, validating the robustness of our selected instruments.

**Figure 4:**
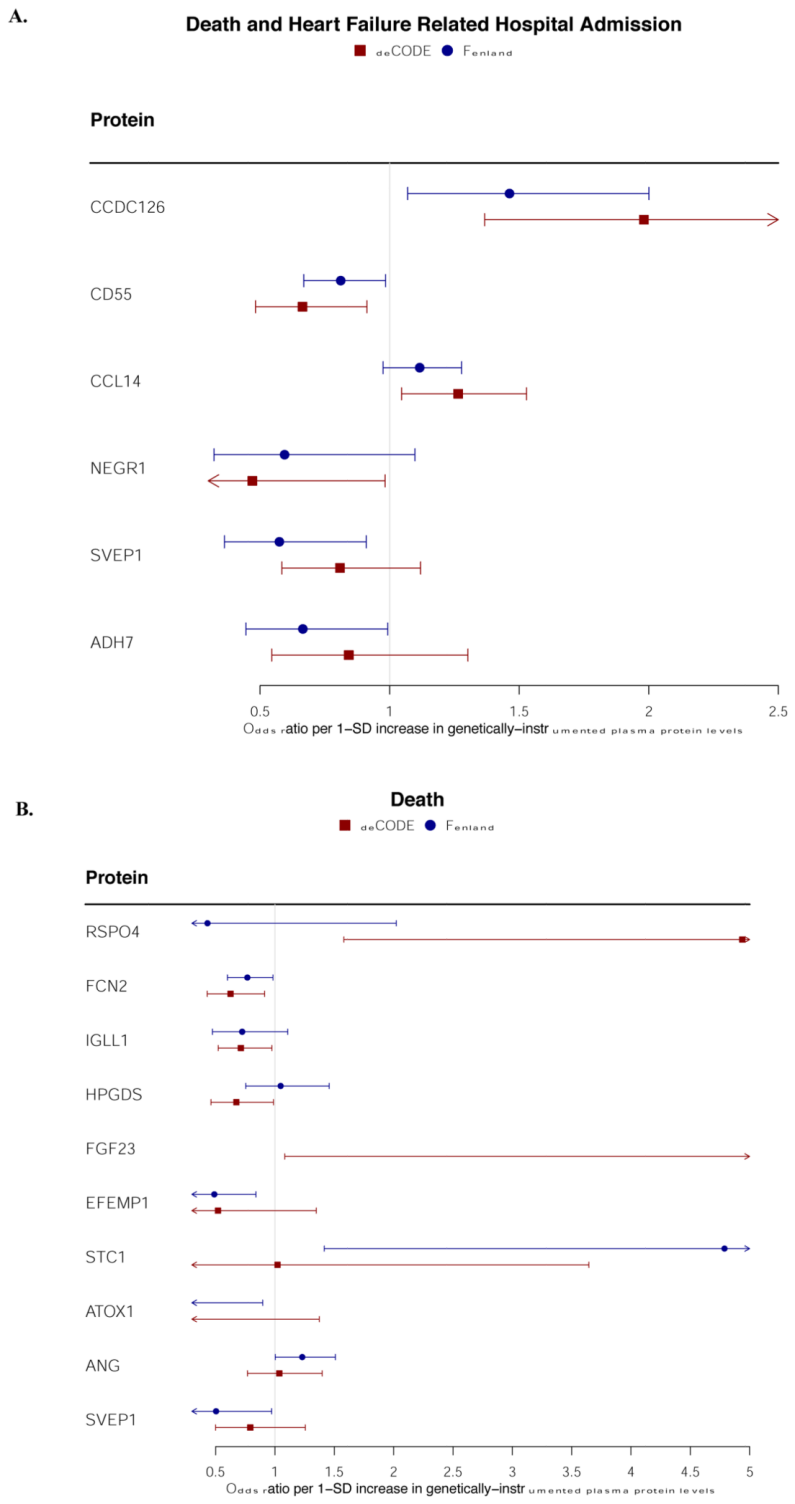
Two-sample MR effect estimates per 1-SD higher circulating plasma protein levels for (A) death and heart failure related hospital admission (DHFA) and (B) death. Only associations between genetically-predicted proteins and HF related outcomes with statistically significant estimates (P <0.05) in either deCODE or Fenland cohorts are reported.

Our *cis*-MR analyses highlighted 5 out of 83 proteins to be significantly associated with DHFA using deCODE genetic instruments as exposures, and 4 out of 63 proteins using Fenland genetic instruments as exposures (see Supplementary Tables 5-6 for full results). Genetically predicted levels of 2 proteins were significantly associated with risk of DHFA across both analyses, 3 were associated with the risk of DHFA only in deCODE-based analyses, whereas 2 were associated with the risk of DHFA only in Fenland-based analyses. The 2 proteins that replicated across the 2 sets of analyses were coiled-coil domain containing 126 (CCDC126), which was associated with an increased risk of DHFA (deCODE: Std OR=1.98, 95%CI=1.36-2.87, *P*=0.0003; Fenland: Std OR=1.46, 95%CI=1.06-2.00, *P*=0.02), and complement decay-accelerating factor (CD55), which was associated with a reduced risk of DHFA (deCODE: Std OR=0.66, 95%CI=0.48-0.91, *P*=0.01; Fenland: Std OR=0.81, 95%CI=0.67-0.98, *P*=0.03). Elevated genetically-predicted levels of NEGR1, SVEP1 and alcohol dehydrogenase 7 (ADH7) were also associated with a reduced risk of DHFA, while elevated genetically-predicted levels of CCL14 were associated with an increased risk of DHFA (Figure 4A). When more than 2 genetic instruments were available, weighted median-MR and MR-Egger estimates were consistent with MR-IVW estimates (Supplementary Tables 5-6).

We also performed sensitivity MR analyses using *cis*- and *trans*-pQTLs for each of the proteins above. In these analyses, findings for CD55 and CCL14 were replicated with consistent directions of effect across both sets of genetic instruments (Supplementary Tables 9-10).

Genetically-predicted levels of several additional proteins were found to be associated with the risk of DHFA were found in *cis*- and *trans*-pQTL MR analyses, but were not associated in *cis*-only analyses, reducing confidence regarding their causal effect. These are listed in Supplementary Tables 9-10.

### Association between measured protein levels and the risk of death: Survival analysis

We found 2,062 proteins to be significantly associated with the risk of death. A volcano plot showing the relationship between baseline plasma protein levels and the risk of incident death is shown in Supplementary Figure 1B. The hazard ratios, 95%CIs and P values are included in Supplementary Table 3.

The top proteins positively associated with the risk of death included GDF15 (Std HR= 2.35, *P*<0.0001), follistatin-related protein 3 (FSTL3) (Std HR=2.31, *P*<0.0001), β2 microglobulin (B2M) (Std HR=2.22, *P*<0.0001), transgelin (TAGLN; Std HR=2.19, *P*<0.0001), cystatin C (CST3; Std HR=2.16, *P*<0.0001), EFEMP1 (Std HR=2.24, *P*<0.0001), WAP four-disulfide core domain 2 (WFDC2; Std HR=, *P*<0.0001), ribonuclease A family member 1 (RNASE1; Std HR=2.13, *P*<0.0001), SVEP1 (Std HR=2.21, *P*<0.0001), and small ubiquitin-like modifier 2 (SUMO2; Std HR=2.07, *P*<0.0001). The top 10 proteins negatively associated with the risk of death included CDH3 (Std HR=0.49, *P*<0.0001), epidermal growth factor receptor (EGFR; Std HR=0.51, *P*<0.0001), BCHE (Std HR= 0.49, *P*<0.0001), growth hormone receptor (GHR; Std HR= 0.51, P<0.0001), CILP2 (Std HR=0.53, *P*<0.0001), NOTUM (Std HR=0.53, *P*<0.0001), Rho guanine nucleotide exchange factor 1 (ARHGEF1; Std HR=0.57, *P*<0.0001), PLA2G12 (Std HR=0.54, *P*<0.0001), SET (Std HR=0.55, *P*<0.0001), and PLG (Std HR=0.54, *P*<0.0001).

We report 243 proteins to be significantly associated with death in analyses adjusted for the MAGGIC risk score and NT-proBNP. A volcano plot showing the relationship between baseline plasma protein levels and the risk of incident death is shown in Figure 2B. Hazard ratios, 95%CIs and P values are included in Supplementary Table 4.

In our adjusted analyses, the top proteins that were positively associated with risk of death included SUMO2 (Std HR=1.56, *P*<0.0001), GDF15 (Std HR=1.68, *P*<0.0001), FSTL3 (Std HR=1.66, *P*<0.0001), gamma-aminobutyric acid A receptors (GABARAP; Std HR=1.53, *P*<0.0001), B2M (Std HR=1.57, *P*<0.0001), SVEP1 (Std HR=1.60, *P*<0.0001), EGF-containing fibulin-like extracellular matrix protein 1 (EFEMP1; Std HR=1.53, *P*<0.0001), heparin binding growth factor (HDGF; Std HR=1.40, *P*<0.0001), antioxidant 1 copper chaperone (ATOX1; Std HR=1.47, *P*<0.0001), and CST3 (Std HR=1.53, *P*<0.0001). Conversely, the top 10 proteins that were negatively associated with risk of death included EGFR (Std HR=0.70, *P*<0.0001), CDH3 (Std HR=0.70, *P*<0.0001), HPGDS (Std HR=0.74, *P*<0.0001), BCHE (Std HR=0.71, *P*<0.0001), CLEC3B (Std HR=0.74, *P*<0.0001), PLA2G12 (Std HR=0.73, *P*<0.0001), leukocyte immunoglobulin-like receptor subfamily A member 4 (LILRA4; Std HR=0.76, *P*<0.0001), inter-alpha-trypsin inhibitor heavy chain 2 (ITIH2; Std HR=0.75, *P*<0.0001), CILP2 (Std HR=0.74, *P*<0.0001), and ADAMTS1 (Std HR=0.74, *P*<0.0001).

### Mendelian Randomization analyses: genetically-predicted protein levels and the risk of death

Among the proteins associated with death in the analyses above, we found 138 proteins in the deCODE study and 118 proteins in the Fenland study with available *cis*-pQTLs as genetic instruments for death (Figure 1). Five out of 138 deCODE proteins had supportive evidence for a potentially causal relationship with death, as well as 6 out of 118 Fenland proteins (see Supplementary Tables 7-8 for full results).

**Figure 5:**
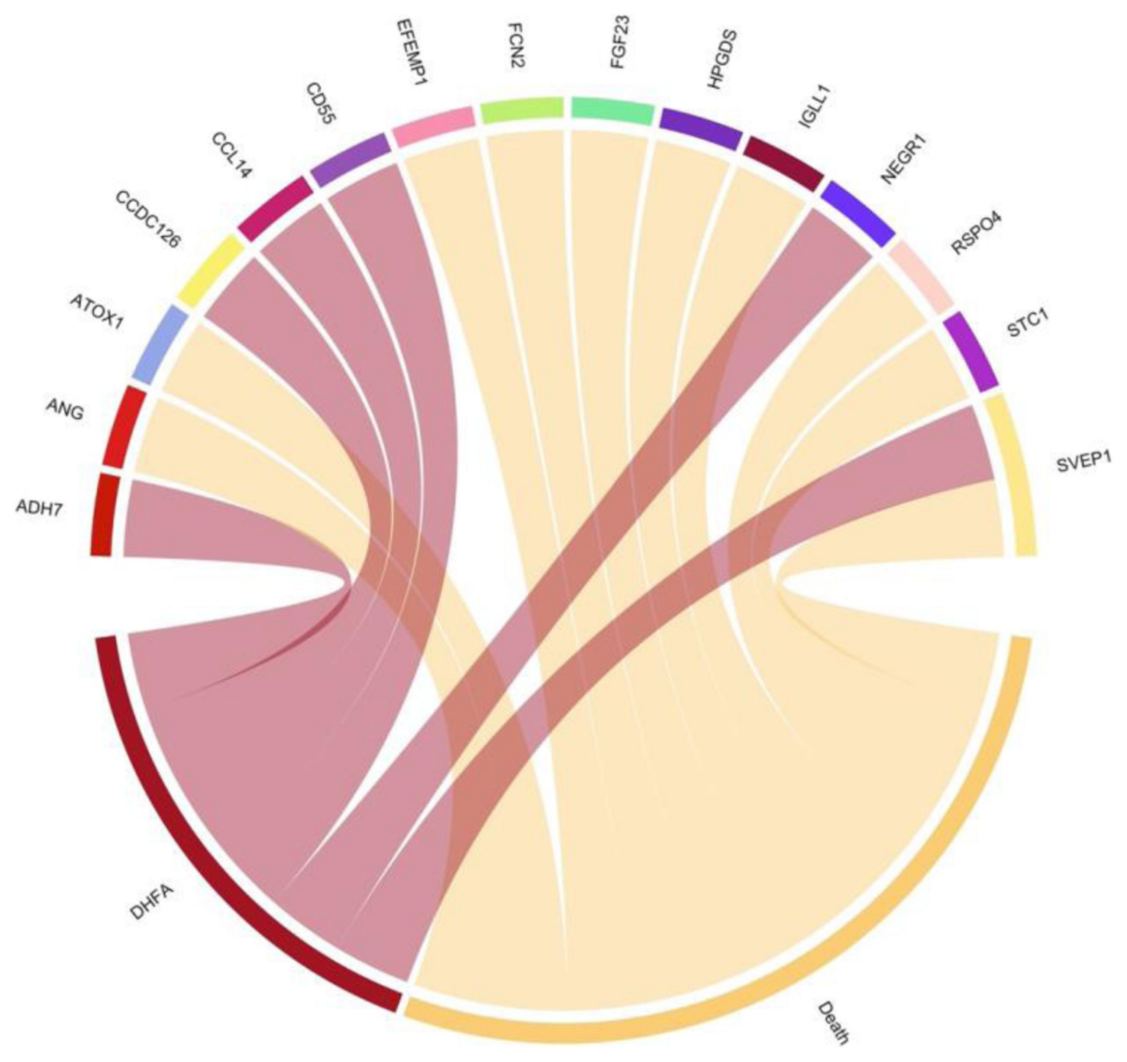
Circos plot showing the relationships between heart failure (HF) related outcomes and significantly associated proteins from *cis*-Mendelian randomization (MR).

**Figure 6:**
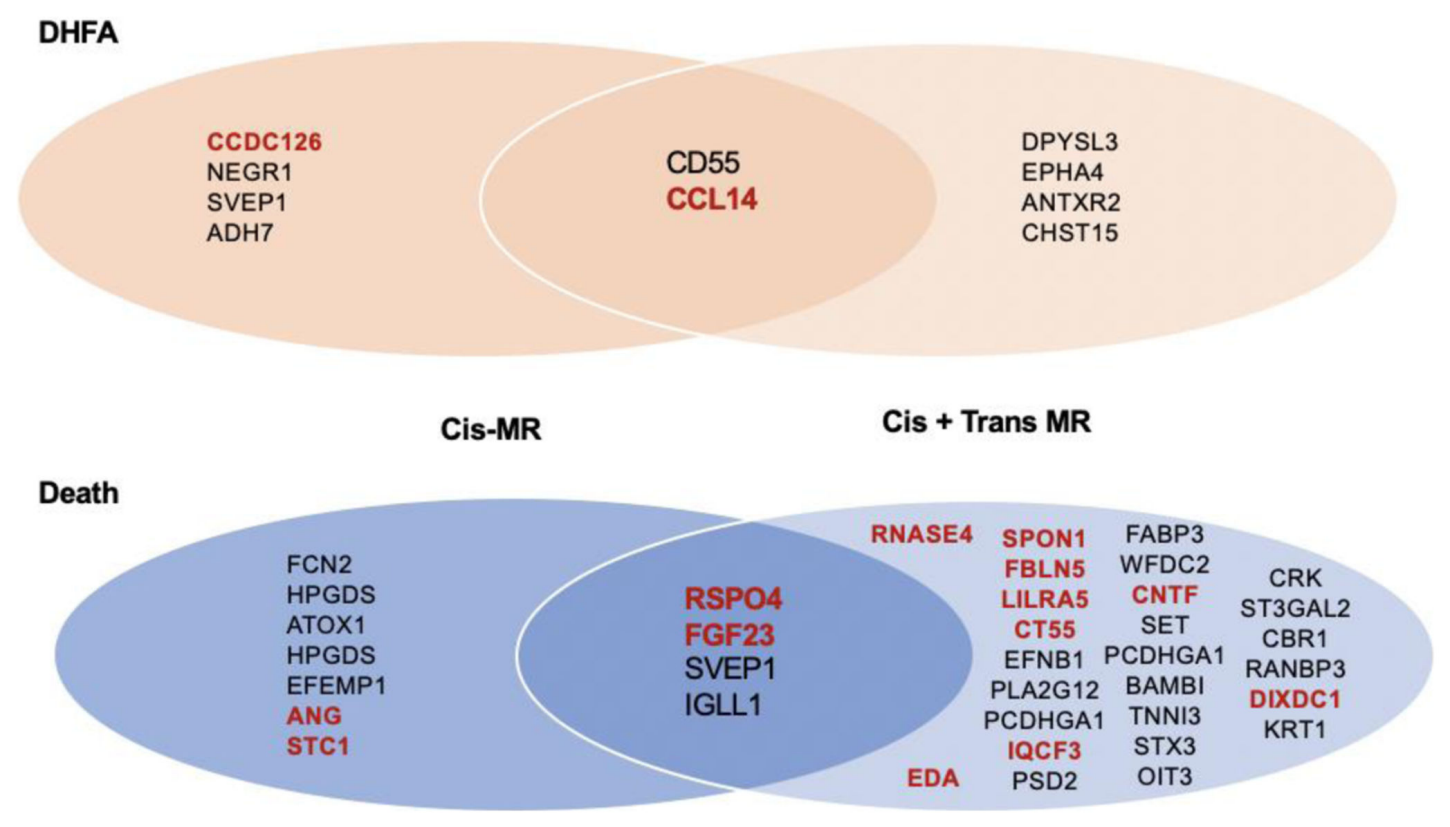
Summary of proteome-wide Mendelian randomization analyses for heart failure (HF) adverse outcomes death and death and HF-related hospital admission (DHFA). Proteins that have detrimental effects are bolded and shown in red.

*Cis*-MR analyses highlighted that increased genetically-predicted circulating levels of FCN2 had a protective effect on death using pQTLs from both the deCODE and Fenland cohorts (deCODE: Std OR=0.62, 95%CI=0.43-0.91; P=0.014; Fenland: Std OR = 0.77, 95%CI=0.60-0.98; P=0.036). Analyses further highlight potentially protective effects of IGLL1 (deCODE: Std OR=0.71, 95%CI=0.52-0.97, *P*=0.03), HPGDS (deCODE: Std OR=0.67, 95%CI=0.46-0.98, *P*=0.04), EFEMP1 (Fenland: OR=0.49, 95%CI=0.28-0.84, *P*=0.009), ATOX1 (Fenland: Std OR=0.21,95%CI=0.05-0.89, *P*=0.03), and SVEP1 (Fenland: Std OR=0.50, 95%CI= 0.26-0.97, *P*=0.04) on death, and potentially detrimental effects of RSPO4 (deCODE: Std OR=4.95, 95%CI=1.58-15.42, *P*=0.005), FGF23 (deCODE: Std OR=18.05, 95%CI=1.08-301.17, *P*=0.04), STC1 (Std OR=4.78, 95%CI=1.41-16.19, *P*=0.01), ANG (Fenland: Std OR=1.23, 95%CI=1.00-1.51, *P*=0.05) on mortality outcomes (Figure 4B). When more than 2 genetic instruments were available, weighted median-MR and MR-Egger estimates were consistent with MR-IVW estimates (Supplementary Tables 11-12). Findings for RSPO4, IGLL1, FCN2, FGF23, SVEP1 were replicated when we used both *cis-* and *trans-* acting pQTLs for analyses (Supplementary Tables 7-10) (Table 1). Conventional MR analysis also identified and replicated an association between FBLN5 and death (deCODE: Std OR=2.63, 95%CI=1.15-6.01, *P*=0.02; Fenland: Std OR=2.33, 95%CI=1.09-4.98, *P*=0.03; Supplementary Tables 9-10). Lower levels of circulating EPH4 were associated with increased risk of DHFA in conventional MR analyses (deCODE: Std OR=0.81, 95%CI=0.68-0.98, *P*=0.03; Fenland: Std OR=0.77, 95%CI=0.61-0.96, *P*=0.02; Supplementary Tables 7-8). A summary of these results can be found in Supplementary Table 11, and Figures 5-6.

### Genetic colocalization sensitivity analyses

Genetic colocalization analyses was performed to assess the robustness of the MR estimates among the proteins that had consistently significant effects in our *cis* and conventional MR analyses. Findings for CD55, CCDC126 and CCL14 were consistent for DHFA and showed support of colocalization between plasma protein levels and HF related adverse outcomes in their respective gene coding regions, with conditional colocalization probability PP_(H4/[H3+H4]_>0.90. Particularly, elevated CCDC126 levels colocalized with DHFA with a PP_H4_ > 0.70. Similarly, CD55 colocalized with DHFA with a PPH4 ~ 0.70. Similarly, RSPO4, FGF23, FCN2, IGLL1 and FBLN5 had consistent MR findings in relation to all-cause mortality, with reported conditional colocalization probabilities exceeding 0.95 with both HF related outcomes. MR analyses highlighted a potentially causal effect of SVEP1 on both death and DHFA, and colocalization analyses were supportive of a shared causal variant between circulating protein levels and HF outcomes within the gene region (PP_(H4/[H3+H4])_ =0.88). Full genetic colocalization results are reported in Supplementary Table 12. For all proteins, posterior probabilities for H_3_ – *i.e*., the hypothesis that both traits share distinct causal variants – were all < 0.07.

## Discussion

To our knowledge, our study is the first to comprehensively investigate associations between ~5000 proteins and adverse outcomes in established HF. We leveraged a well-characterized population of 2,248 individuals from the PHFS, combined with genomic data from independent cohorts to gain insights into the putative causal effects of plasma proteins on human HF using an MR framework. A total of 1,477 and 2,064 proteins had associations with the risk of DHFA and mortality, respectively, while MR analyses provided support for potential causality for 6 proteins for DHFA and 11 proteins for all-cause mortality. Our findings confirm associations from prior epidemiologic studies, identify novel proteins associated with HF outcomes, and establish genetic evidence to support future mechanistic studies of novel therapeutic targets.

### Proteins with Observational Evidence of a role in HF related outcomes

Our observational analyses replicated findings from several prior epidemiologic studies. Consistent with Gui *et al*., who developed a proteomic risk score for HFrEF, STC1 and REN were associated with increased risk of mortality, while BCHE and EGFR were associated with decreased risk, although several other proteins were not replicated in our analyses (ASM, UGT1A6, CA6 and APBB3) (7). Our proteome-wide survival analyses notably confirmed previously reported positive associations in HFrEF between outcomes and SVEP1 (31), ANGPT2 (32), CETP (33), EFEMP1 (31), and negative associations with CLEC3B, CILP2, NPS, GSN, PTPRS, amongst other proteins. Previous positive associations have also been established between SUMO2 and cardiac function through *in vitro* studies, although it was not specific to HFrEF (34).

In addition to replicating prior findings, our study identified a large number of novel associations between circulating protein levels and the risk of incident DHFA or death. We also report a large number of prognostic associations of circulating plasma proteins that are independent of standard clinical prognostic tools (MAGGIC risk score and NT-proBNP), which were identified at proteome-wide levels of statistical significance. Biomarkers that are used routinely in clinical practice are limited, do not fully predict the incident risk of outcomes, and may not represent therapeutic targets. Our study identified several proteomic candidates for development, but further research will be required to assess the potential clinical role of these biomarkers.

### Evidence of Causality between Proteins and HF related Outcomes

#### Genomic analyses

In addition to our analyses regarding observed protein levels and incident outcomes, we performed causal inference analyses to further screen and prioritize biomarkers for HF risk detection and therapeutic development. Examining the proteins that had supporting evidence for causality yields biological insights into the underlying mechanisms of HF. Specifically, we used *cis*-MR analysis as our main method to provide estimates with higher confidence, and accounted for LD correlation structure within the causal model. We found evidence supporting a causal relationship for 6 proteins out of the 126 DHFA-associated proteins from our adjusted observational analyses, and 11 out of 243 proteins for all-cause mortality.

#### Proteins with evidence of a causal detrimental effect on Death and/or DHFA

Our epidemiologic, MR, and colocalization analyses report a strong effect of FGF23 on death among individuals with HF. This is in line with the literature, as FGF23 has been previously associated with heart failure and atrial fibrillation (35–39), as well as with outcomes in established HFpEF or HFrEF (37,40–44), although the prognostic value has been limited in some studies (45) FGF23 is a hormone produced by osteocytes and osteoblasts which aids in phosphate excretion by the kidney and plays a role in vitamin D biosynthesis. Individuals with chronic kidney disease (CKD) who also have high levels of plasma FGF23 tend to have left-ventricular dysfunction, and FGF23 levels have also been associated with ischemic heart disease prognosis independently of kidney function (46,47). Experimental activation of the FGF-23/FGFR pathway has been implicated in cardiac hypertrophy and fibrosis, supporting its role as a potential therapeutic target (48). While prior MR studies have investigated the role of genetically-determined FGF23 with the risk of HF development (49,50), our MR analysis is the first to assess the causal relationship between FGF23 levels and outcomes in established HF.

Our findings further confirm results from prior MR studies of HF and cardiovascular disease. For example, Moncla *et al*. identified a potentially causal effect of higher genetically predicted CCDC126 levels on prevalent HF (51). Similarly, higher CCL14 levels have been previously reported to play a role in adverse kidney events after cardiac surgery (52), and to be associated with cardiovascular events (53). We now extend these associations to include an association between genetically-predicted levels of CCDC126 and outcomes in established HF. RSPO4 levels have been recently associated with echocardiographic variables and natriuretic peptide levels (54); however, there are no other reports of its association with cardiovascular events in other studies. We found an association between genetically-predicted levels of RSPO4 and the risk of death in established HF, which warrants further investigation in epidemiological, clinical and experimental settings.

#### Proteins with evidence of a causal protective effect on death and/or DHFA

We identified several plasma proteins with potential protective effects on death and/or DHFA. HPGDS was identified in this group. HPGDS is a member of the glutathione S-transferase super family of enzymes that catalyses the conjugation of electrophilic substances with reduced glutathione, and serves as a regulator of the inflammatory response. Although the role of HPGDS in HF has not been previously investigated, experimental data indicates that it has anti-inflammatory effects (55,56). HF is associated with activation of the innate and adaptive immune systems, and therapeutic targeting of inflammatory signalling with canakinumab was associated with decreased hospitalization for HF and DHFA in the Canakinumab Anti-inflammatory Thrombosis Outcome Study (CANTOS) (57,58). In summary, these findings suggest that targeting inflammatory signalling mediated by HPGDS may represent a novel therapeutic target to improve HF outcomes.

ADH7 is a member of the alcohol dehydrogenase family. Data previously suggested that elevated cardiac acetaldehyde exposure via ADH may exacerbate alcohol-induced myocardial dysfunction, hypertrophy and insulin sensitivity, indicative of a key role of ADH proteins in insulin resistance and alcohol-induced cardiac dysfunction (59). The enzyme is known to be inefficient in ethanol oxidation but is most active as a retinol dehydrogenase, participating in the synthesis of retinoic acid, an important hormone for cellular differentiation. ADH7 differs from the other ADH family proteins in that it is expressed to a larger extent in the stomach and airway epithelium rather than the liver. While the exact mechanism linking ADH7 to HF prognosis is not yet clear, our MR findings support further investigation as a candidate target.

We identified an association between higher genetically-predicted levels SVEP1 plasma proteins and a lower risk of both death and DHFA, suggesting a potential protective effect. SVEP1 is an extracellular matrix protein expressed in vascular smooth muscle cells (VSMCs) within the atherosclerotic plaque. Some observational studies have identified a positive association between measured SVEP1 levels and the risk of coronary artery disease (60), as we do in our observational PHFS analyses. An atheroprotective role for SVEP1 has been previously reported in the literature (61) through a potential lipid metabolism independent mechanism. Similarly, a missense variant (encoding p.D2702G) in the *SVEP1* gene was previously associated with an increased risk of coronary artery disease, while haploinsufficiency in mice has been linked to reduced burden of atherosclerosis (60,62). Our MR analyses suggest a potential protective role of circulating SVEP1. We should note, however, that these findings are discordant in direction of effect with our observation that increased circulating SVEP1 is strongly associated with an increased risk of adverse HF outcomes. First, this discordance may be attributable to differences in selection or collider biases in observational settings, or incomplete adjustment for collider bias in our MR analysis. Second, the increase in circulating SVEP1 may reflect decreased tissue-level activity, which would be consistent from the protective findings of haploinsufficiency in mice. Given the combination of factors that influence measured protein levels in plasma, the specific mechanisms linking SVEP1 with adverse outcomes in HF warrants further investigation.

Our MR findings further report NEGR1 as having a potentially protective effect on HF outcomes and prognosis. NEGR1 has been suggested to regulate cellular fat content via CD36 expression regulation (63), and its gene *NEGR1* has been identified as risk locus for obesity in genome-wide association studies. It has been suggested as having a role in psychiatric disorders in mouse model studies (64,65). In human studies, the SNP rs10789336 in *NEGR1* was associated with expression levels of RPL31P12 in brain tissues, and with the risk for major depressive disorder (MDD) (66), implying shared genetic liability between mood disorders and cardiovascular disease risk. More recently, an inverse association between NEGR1 and incident HF was identified in a proteome-wide association study (67). Our results validate this finding, and call for further investigation of the mechanism by which NEGR1 may impact outcomes in HF.

We identified discordant associations between measured and genetically-predicted levels of EFEMP1 and all-cause mortality. Levels of plasma EFEMP1 have been previously associated with incident HF in a community-based study, with a reported HR of 1.34 [1.14-1.57] (68). This is in line with findings, in which measured plasma levels of EFEMP1 were associated with an increased risk of all-cause death. However, our collider bias adjusted MR analysis reported a protective association between genetically-predicted levels of EFEMP1 and mortality (Fenland: Std OR=0.49; 95%CI=0.28-0.84). Like SVEP1, EFEMP1 is an extracellular matrix protein. The discordant observational and MR associations may reflect differences in the presence of collider bias, incomplete adjustment for confounding, or may reflect a more complex biological mechanism that dissociates tissue and plasma levels, and warrants further investigation.

#### Strengths and limitations

Our study has notable strengths. Given the increased power and comprehensive proteomics platform, our study provided a unique opportunity to identify and report hundreds of proteins associated with the risk of HF adverse outcomes. To our knowledge, this is the largest study to comprehensively investigate the associations of ~5000 proteins on HF outcomes from a large HF patient cohort, as well as their putative causal effect using an MR framework. The combination of survival analysis and genetic epidemiology (MR and colocalization) methods increase the robustness of our findings. However, as with all studies, our findings should be interpreted within the context of their limitations. First, the PHFS cohort included primarily individuals with HFrEF, which may limit the generalizability of results to patients with HFpEF. Additionally, because we based our MR analyses on the significant results from the PHFS survival analyses of measured protein levels, we may have missed proteins that, despite having a causal role on outcomes, might not have been identified in initial survival analyses due to confounding. However, we chose this trade-off to increase the validity/reliability of our results. Next, MR analyses rely on a number of assumptions that impact the reliability of causal inference, which we aimed to overcome through a series of complementary methods. Firstly, we restricted MR analyses to *cis*-acting genetic instruments to limit horizontal pleiotropy. Colocalization analysis is recommended in conjunction with *cis*-MR for sound interpretation of MR findings (69). We also therefore implemented colocalization analyses to test whether MR assumptions were violated at each locus. For instance, low posterior probabilities for H3 (the hypothesis that the exposure and outcome share distinct causal variants) at all loci provided supporting evidence that the assumption of no pleiotropy in our MR analyses was not violated due to linkage disequilibrium. We also corrected for correlation between genetic instruments in our inverse variance weighted MR analyses to minimize multicollinearity in the model and avoid false positive results. Another consideration is that our findings may have been conditioned on HF, since all individuals in our cohort were selected on the basis of the presence of established HF. This introduces potential collider bias, which we specifically accounted for in our analyses. Lastly, causal inferences made through MR are suggestive of potential causal effects of genetic predisposition of an exposure (protein levels) on an outcome of interest (HF risk and mortality) but does not provide evidence that therapeutic intervention targeting the exposure will change the outcome. Similarly, MR instruments are used as a proxy of lifelong genetic effects, which may differ from pharmacologic interventions that may occur over shorter time periods with different potency. Further investigation, including more specific drug target analyses and validation in model systems is required to establish the causal mechanisms and effects of protein inhibition or stimulation on HF outcomes.

## Conclusions

We investigated the effects of ~5000 proteins on adverse outcomes in adults with HF in the PHFS cohort, replicating several associations in an integrative MR framework using independent summary statistics to derive valid and robust genetic instruments. The discovery of novel HF biomarkers highlights the significance in the scope of large cohort proteomic association studies.

## Supplementary Material

Supplementary material

## Figures and Tables

**Figure 3:**
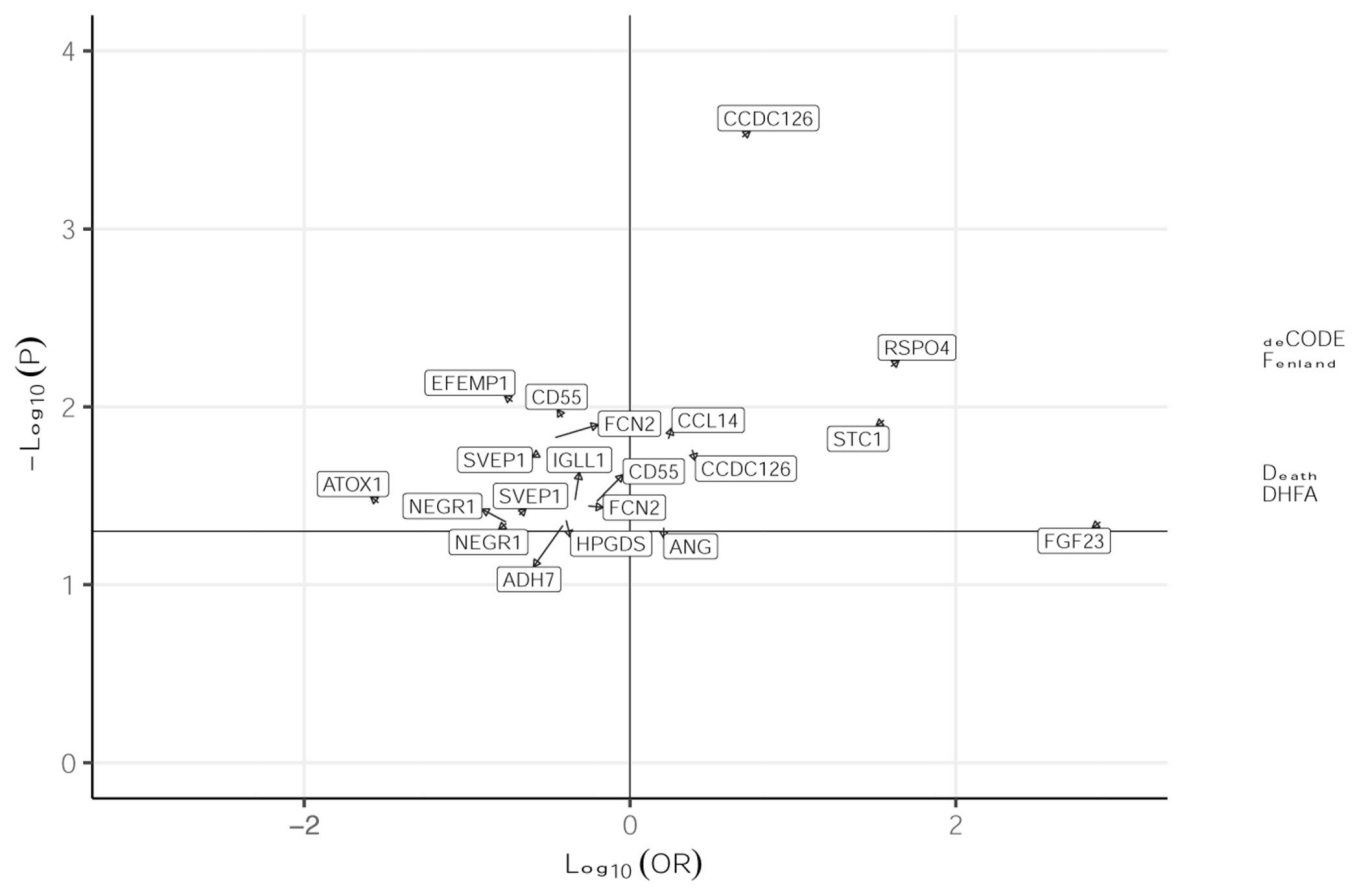
Proteome-wide *cis*-Mendelian randomization study results for associations between genetically-instrumented proteins from deCODE and Fenland and death and death and heart failure-related hospital admission (DHFA) in the PHFS study. Log (OR) regression estimates are plotted against -log(P-value). The dashed line represents the 5% alpha threshold. Abbreviations: ADH7, alcohol dehydrogenase 7; ANG, angiogenin; ATOX1, antioxidant 1 copper chaperone; CCDC126, coiled-coil domain containing 126; CD55, decay-accelerating factor; CCL14, chemokine (C-C motif) ligand 14; FCN2, ficolin 2; FGF23, fibroblast growth factor-23; HPGDS, hematopoietic prostaglandin D synthase; IGLL1, immunoglobulin lambda like polypeptide 1; SVEP1, sushi, von Willebrand factor type A, EGF and pentraxin domain containing 1; NEGR1, neuronal growth factor 1; STC1, stanniocalcin-1 ; RSPO4, R-spondin-4.

**Table 1:** General characteristic of study participants with versus without available plasma samples.

	Participants without available proteomics (n=131)	Participants with available proteomics (n=2,248)	*P* value
**Demographic Characteristics**			
Age, y	59.9 (22.2)	54.52 (15.78)	0.0033
Male sex	47 (56.63%)	1483 (66.35%)	0.0764
Race			<0.0001
White	78 (65.00%)	1641 (73.00%)	
Black	3 (2.50%)	471 (20.95%)	
Hispanic	1 (0.83%)	21 (0.93%)	
Other	38 (31.67%)	115 (5.12%)	
BMI, kg/m^2^	30.14 (9.05)	29.38 (6.84)	0.3261
Systolic BP, mmHg	111.62 (25.49)	113.06 (20.13)	0.5213
Diastolic BP, mmHg	67.72 (15.20)	68.70 (11.81)	0.4652
LV ejection fraction, %	33.93 (25.48)	28.69 (16.33)	0.0076
**Laboratory parameters**			
eGFR	48.18 (30.22)	52.39 (25.49)	0.1145
Pro-BNP	661.06 (2,393.18)	897.58 (909.33)	0.2158
**Medication uses**			
ACEI ARB	69 (82.14%)	1,903 (84.65%)	0.5380
Aldosterone antagonist	26 (30.95%)	773 (34.39%)	0.5597
Aspirin	55 (65.48%)	1287 (57.25%)	0.1448
Beta blocker	75 (89.29%)	1,973 (87.77%)	0.8647
Calcium channel blocker	13 (15.48%)	205 (9.12%)	0.0562
Hydralazine	5 (5.95%)	191 (8.50%)	0.5476
Nitrate	8 (9.52%)	357 (15.88%)	0.1272
Statin	48 (57.14%)	1,169 (52.00%)	0.3751
Warfarin	28 (33.33%)	851 (37.86%)	0.4245
Insulin	12 (14.29%)	284 (12.63%)	0.6175
**Medical History**			
Diabetes Mellitus	25 (29.76%)	642 (28.56%)	0.8064
History of coronary stent	18 (21.43%)	498 (22.15%)	1.0000
Coronary artery bypass graft	15 (17.86%)	417 (18.55%)	1.0000
Atrial fibrillation/flutter	30 (35.71%)	821 (36.52%)	0.9087
History of smoking	4 (4.76%)	203 (9.03%)	0.2389
**NYHA **			
Class I - II	56	1376	
Class III - IV	26	843	

ACE, angiotensin-converting enzyme; ARB, angiotensin-receptor blockers; BMI, body mass index; BP, blood pressure; eGFR, estimated glomerular filtration rate; LV, left ventricle; NYHA, New York Heart Association; pro-BNP, pro-B-type natriuretic peptide. Numbers indicate means and standard deviations.

**Table 2:** List of top 20 proteins associated with DHFA and death with a P value < 0.0001 in the PHFS.

Protein Name	Standardized Hazard Ratio	Alpha corrected P- value	UniProt ID	Symbol
**DHFA**				
A disintegrin and metalloproteinase with thrombospondin motifs like 2	1.403	P<0.0001	Q86TH1	ADAMTSL
Pulmonary surfactant-associated protein C	1.337	2.48E-12	P11686	SFTPC
Thrombospondin-2	1.386	7.93E-12	P35442	THBS2
Protein FAM163A	1.318	4.55E-11	Q96GL9	FAM163A
Insulin-like growth factor-binding protein 7	1.336	8.84E-11	Q16270	IGFBP7
Cholesteryl ester transfer protein	1.295	1.29E-09	P11597	CETP
Fibroblast growth factor 23	1.277	3.29E-09	Q9GZV9	FGF23
Tenascin	1.281	3.78E-09	P24821	TNC
C-C motif chemokine	1.306	4.70E-09	Q16627	CCL14
Collectin-11	1.255	3.65E-08	Q9BWP8	COLEC11
Receptor-type tyrosine-protein phosphatase				
S	0.789	6.84E-10	Q13332	PTPRS
Anthrax toxin receptor 2	0.823	8.66E-06	P58335	ANTXR2
Neuronal growth regulator 1	0.827	8.80E-06	Q7Z3B1	NEGR1
Cartilage intermediate layer protein 2	0.810	1.77E-05	Q8IUL8	CILP2
Receptor-type tyrosine-protein phosphatase			P23468	PTPRD
delta	0.832	1.86E-05		
Gelsolin	0.838	4.47E-05	P06396	GSN
Neurotrimin	0.837	5.73E-05	Q9P121	NTM
Ecto-ADP-ribosyltransferase 3	0.842	6.70E-05	Q13508	ART3
Neuropeptide S	0.842	8.67E-05	P0C0P6	NPS
Notum, palmitoleoyl-protein carboxylesterase	0.814	8.89E-05	Q6P988	NOTUM
**Death**				
Small ubiquitin-related modifier 2	1.559	2.31E-12	P61956	SUMO2
Growth/differentiation factor 15	1.684	5.12E-12	Q99988	GDF15
Follistatin-related protein 3	1.660	2.03E-11	O95633	FSTL3
Gamma-aminobutyric acid receptor-associated protein-like 1	1.529	4.31E-11	Q9H0R8	GABARAP
Beta-2-microglobulin	1.570	1.92E-10	P61769	B2M
Gamma-aminobutyric acid receptor-associated protein-like 1	1.495	1.92E-10	O95166	GABARAP
Sushi, von Willebrand factor type A, EGF and pentraxin domain-containing protein 1	1.601	4.85E-10	Q4LDE5	SVEP1
EGF-containing fibulin-like extracellular matrix protein 1	1.583	5.62E-10	Q12805	EFEMP1
Hepatoma-derived growth factor	1.403	4.15E-09	P51858	HDGF
Antioxidant 1 Copper Chaperone	1.469	4.80E-09	P01034	ATOX1
Epidermal growth factor receptor	0.699	7.07E-08	P00533	EGFR
Cadherin-3	0.702	1.68E-06	P22223	CDH3
Hematopoietic prostaglandin D synthase	0.745	1.82E-06	O60760	HPGDS
Cholinesterase	0.713	6.00E-06	P06276	BCHE
Group XIIB secretory phospholipase A2-like protein	0.743	8.40E-06	Q9BX93	PLA2G12
Leukocyte immunoglobulin-like receptor subfamily A member 4	0.760	1.19E-05	P19823	LILRA4
Inter-alpha-trypsin inhibitor heavy chain H2	0.754	1.37E-05	P19823	ITIH2
Cartilage intermediate layer protein 2	0.741	2.32E-05	Q8IUL8	CILP2
A disintegrin and metalloproteinase with thrombospondin motifs 1	0.746	3.02E-05	Q76LX8	ADAMTS1
Proto-oncogene tyrosine-protein kinase receptor	0.752	8.86E-05	P07949	RET
