# Supplementary material for: Proteome-wide Mendelian randomization of Adverse Outcomes in Human Heart Failure
Source: J Am Heart Assoc. Author manuscript; Available in PMC 2024 May 8. (PMC10944037; doi:10.1161/JAHA.123.031154)
Supplement: Supplementary material [file EMS195147-supplement-Supplementary_material.pdf]

Supplemental Material

Table S1. General characteristic of PHFS study participants with versus without available plasma samples.

	Penn Heart Failure Study Cohort			Washington University Study Cohort		
	**Participants without available proteomics (n=131)**	**Participants with available proteomics (n=2,248)**	***P***	**Participants without available proteomics (n=10)**	**Participants with available proteomics (n=1,080)**	***P***
**Demographic Characteristics**						
Age, y	59.9 (22.2)	54.52 (15.78)	0.0033	56.1 (46.3 to 65.8)	54.8 (53.9 to 55.6)	0.7732
Male sex	47 (56.63%)	1483 (66.35%)	0.0764	7 (70.00%)	689 (63.80%)	1
Race			<0.0001			
White	78 (65.00%)	1641 (73.00%)		6 (60.00%)	782 (72.81%)	
Black	3 (2.50%)	471 (20.95%)		4 (40.00%)	290 (27.00%)	
Hispanic	1 (0.83%)	21 (0.93%)		0 (0.00%)	1 (0.09%)	
Other	38 (31.67%)	115 (5.12%)		0 (0.00%)	1 (0.09%)	
BMI, kg/m^2	30.14 (9.05)	29.38 (6.84)	0.3261	31.1 (25 to 37.2)	30.6 (30 to 31.1)	0.8394
Systolic BP, mmHg	111.62 (25.49)	113.06 (20.13)	0.5213	110 (98 to 123)	114 (112 to 115)	0.5961
Diastolic BP, mmHg	67.72 (15.20)	68.70 (11.81)	0.4652	63.1 (56.1 to 70.1)	70.5 (69.8 to 71.1)	0.0397
LV ejection fraction, %	33.93 (25.48)	28.69 (16.33)	0.0076	29.5 (18.5 to 40.5)	29.9 (29 to 30.8)	0.9353
**Laboratory parameters**						
eGFR	48.18 (30.22)	52.39 (25.49)	0.1145			
Pro-BNP	661.06 (2,393.18)	897.58 (909.33)	0.2158			
**Medication uses**						
ACEI ARB	69 (82.14%)	1,903 (84.65%)	0.538	5 (50.00%)	803 (74.35%)	0.1371
Aldosterone antagonist	26 (30.95%)	773 (34.39%)	0.5597	5 (50.00%)	452 (41.85%)	0.75
Aspirin	55 (65.48%)	1287 (57.25%)	0.1448	5 (50.00%)	739 (68.43%)	0.3036
Beta blocker	75 (89.29%)	1,973 (87.77%)	0.8647	7 (70.00%)	871 (80.65%)	0.4191

Calcium channel blocker	13 (15.48%)	205 (9.12%)	0.0562	1 (10.00%)	102 (9.44%)	1
Hydralazine	5 (5.95%)	191 (8.50%)	0.5476	3 (30.00%)	156 (14.44%)	0.1684
Nitrate	8 (9.52%)	357 (15.88%)	0.1272	1 (10.00%)	189 (17.50%)	1
Statin	48 (57.14%)	1,169 (52.00%)	0.3751	5 (50.00%)	520 (48.15%)	1
Warfarin	28 (33.33%)	851 (37.86%)	0.4245	4 (40.00%)	345 (31.94%)	0.7345
Insulin	12 (14.29%)	284 (12.63%)	0.6175	1 (10.00%)	189 (17.50%)	1
**Medical History**						
Diabetes Mellitus	25 (29.76%)	642 (28.56%)	0.8064	2 (20.00%)	334 (30.93%)	0.7324
History of coronary stent	18 (21.43%)	498 (22.15%)	1	2 (20.00%)	260 (25.32%)	1
Coronary artery bypass graft	15 (17.86%)	417 (18.55%)	1	0 (0.00%)	190 (19.67%)	0.3567
Atrial fibrillation/flutter	30 (35.71%)	821 (36.52%)	0.9087	4 (40.00%)	385 (37.45%)	1
History of smoking	4 (4.76%)	203 (9.03%)	0.2389	4 (40.00%)	622 (66.10%)	0.0993
**NYHA**						
Class I - II	56	1376				
Class III - IV	26	843				
ACE, angiotensin-converting enzyme; ARB, angiotensin-receptor blockers; BMI, body mass index; BP, blood pressure; eGFR, estimated glomerular filtration rate; LV, left ventricle; NYHA, New York Heart Association; pro-BNP, pro-B-type natriuretic peptide. Numbers indicate means and standard deviations.						

Table S2. General characteristic of study participants in the Washington University cohort that reached outcomes vs participants that did not reach outcomes.

		Participants with available proteomics (n=1,080)				
	**Participants who did not reach DHFA outcome (n=699)**	**Participants who reached DHFA outcome (n=381)**	***P***	**Participants who did not reach death outcome (n=900)**	**Participants who reached death outcome (n=180)**	***P***
**Demographic Characteristics**	Mean (95% CI), or Median (IQR) if indicated			Mean (95% CI, or IQR if indicated)		
Age, y	57.7 (48.1,64.1)	58.1 (49.9,65.4)	0.3674	57.3 (47.8,63.8)	61 (53.3,68.1)	<0.0001
Male sex	425 (60.80%)	264 (69.29%)	0.0055	556 (61.78%)	133 (73.89%)	0.0020
Race						
White	538 (77.52%)	244 (64.21%)	<0.0001	646 (72.26%)	136 (75.56%)	0.3645
Black	154 (22.19%)	136 (35.79%)	<0.0001	246 (27.52%)	44 (24.44%)	0.3970
Asian	1 (0.14%)	0 (0.00%)	1.0000*	1 (0.11%)	0 (0.00%)	1.0000*
Other	1 (0.14%)	0 (0.00%)	1.0000*	1 (0.11%)	0 (0.00%)	1.0000*
BMI, kg/m^2	30.3 (26,36)	30.1 (25.4,36)	0.6970	30.6 (26.2,36.6)	27.8 (24.5,33.7)	<0.0001
Systolic BP, mmHg	113 (103,127)	107 (98,123)	<0.0001	113 (102,128)	104 (96,118)	<0.0001
Diastolic BP, mmHg	72 (66,78)	68 (62,75)	<0.0001	71 (65,78)	66 (61,73)	<0.0001
LV ejection fraction, %	35 (IQR 24,50)	25 (IQR 18,35)	<0.0001	32 (IQR 22,48)	24 (IQR 19.5,32.5)	<0.0001
**Laboratory parameters**						
eGFR	64.2 (48.3,79.8)	51 (34.4,68.8)	<0.0001	62.9 (46.4,79)	46.9 (32.2,63.9)	<0.0001
Pro-BNP	4346 (IQR 2049,10988)	16049 (IQR 7978,28881)	<0.0001	5795 (IQR 2445,14979)	21675 (IQR 10640,35980)	<0.0001
**Medication uses**						

ACEI ARB	566 (80.97%)	237 (62.20%)	<0.0001	709 (78.78%)	94 (52.22%)	<0.0001
Aldosterone antagonist	278 (39.77%)	174 (45.67%)	0.0604	370 (41.11%)	82 (45.56%)	0.2698
Aspirin	468 (66.95%)	271 (71.13%)	0.1583	617 (68.56%)	122 (67.78%)	0.8376
Beta blocker	592 (84.69%)	279 (73.23%)	<0.0001	756 (84.00%)	115 (63.89%)	<0.0001
Calcium channel blocker	72 (10.30%)	30 (7.87%)	0.1926	96 (10.67%)	6 (3.33%)	0.0021
Hydralazine	67 (9.59%)	89 (23.36%)	<0.0001	122 (13.56%)	34 (18.89%)	0.0632
Nitrate	94 (13.45%)	95 (24.93%)	<0.0001	154 (17.11%)	35 (19.44%)	0.4520
Statin	320 (45.78%)	200 (52.49%)	0.0349	416 (46.22%)	104 (57.78%)	0.0046
Warfarin	193 (27.61%)	152 (39.90%)	<0.0001	264 (29.33%)	81 (45.00%)	<0.0001
Insulin	88 (12.59%)	101 (26.51%)	<0.0001	146 (16.22%)	43 (23.89%)	0.0135
**Medical History**						
Diabetes Mellitus	188 (26.90%)	146 (38.32%)	0.0001	273 (30.33%)	61 (33.89%)	0.3461
Atrial fibrillation/flutter	205 (30.60%)	180 (50.28%)	<0.0001	284 (32.98%)	101 (60.48%)	<0.0001
History of coronary stent	134 (20.09%)	126 (35.00%)	<0.0001	188 (21.89%)	72 (42.86%)	<0.0001
Coronary artery bypass graft	96 (15.21%)	94 (28.06%)	<0.0001	133 (16.46%)	57 (36.08%)	<0.0001
History of smoking	74 (12.21%)	31 (9.42%)	0.1971	515 (65.69%)	107 (68.15%)	0.5516
**NYHA**						
Class I	75 (10.73%)	4 (1.05%)	<0.0001	79 (8.78%)	0 (0.00%)	<0.0001
Class II	416 (59.51%)	99 (25.98%)	<0.0001	481 (53.44%)	34 (18.89%)	<0.0001
Class III - IV	31 (4.43%)	87 (22.83%)	<0.0001	340 (37.78%)	146 (81.11%)	<0.0001
ACE, angiotensin-converting enzyme; ARB, angiotensin-receptor blockers; BMI, body mass index; BP, blood pressure; eGFR, estimated glomerular filtration rate; LV, left ventricle; NYHA, New York Heart Association; pro-BNP, pro-B-type natriuretic peptide. Numbers indicate means and standard deviations.						

Table S3. Results from unadjusted cox regression models for DHFA outcomes in the PHFS, replicated in the WashU cohort.

SOMA_ID	PHFS						WashU					
	N	sHR	Std.LB.95CI	Std_UB_95CI	Alpha.corrected.P.value	minus_log10_P	NA	sHR	Std.LB.95CI	Std_UB_95CI	minus_log10_P	P_value
SOMA_P16860_NPPB_7655_11	2234	1.804	1.699	1.916	0.00E+00	81.501	1077	1.753	1.632	1.882	53.083	<0.0001
SOMA_P35442_THBS2_3339_33	2234	1.738	1.634	1.848	0.00E+00	68.814	1077	1.635	1.533	1.744	50.083	<0.0001
SOMA_Q86TH1_ADAMTSL_6379_62	2234	1.716	1.616	1.823	0.00E+00	68.141	1077	1.534	1.436	1.639	36.308	<0.0001
SOMA_Q4LDE5_SVEP1_11109_56	2234	1.633	1.538	1.734	0.00E+00	57.03	1077	1.441	1.363	1.524	37.274	<0.0001
SOMA_Q16627_CCL14_2900_53	2234	1.612	1.52	1.709	0.00E+00	56.807	1077	1.510	1.429	1.594	48.533	<0.0001
SOMA_O95633_FSTL3_3438_10	2234	1.624	1.53	1.724	0.00E+00	56.506	1077	1.489	1.401	1.583	36.451	<0.0001
SOMA_Q16270_IGFBP7_3320_49	2234	1.647	1.549	1.751	0.00E+00	56.482	1077	1.681	1.560	1.812	41.634	<0.0001
SOMA_O15123_ANGPT2_2602_2	2234	1.606	1.515	1.703	0.00E+00	56.078	1077	1.748	1.629	1.877	52.925	<0.0001
SOMA_Q4LDE5_SVEP1_11178_21	2234	1.625	1.53	1.726	0.00E+00	55.416	1077	1.454	1.375	1.539	38.225	<0.0001
SOMA_Q99988_GDF15_4374_45	2234	1.627	1.531	1.729	0.00E+00	54.723	1077	1.590	1.488	1.700	41.849	<0.0001
SOMA_Q9HCB6_SPON1_4297_62	2234	1.619	1.524	1.72	0.00E+00	54.391	1077	1.457	1.379	1.540	39.881	<0.0001
SOMA_P11686_SFTPC_5738_25	2234	1.616	1.522	1.717	0.00E+00	54.184	NA	NA	NA	NA	NA	NA
SOMA_Q8IUL8_CILP2_8841_65	2234	0.631	0.595	0.669	0.00E+00	53.214	1077	0.485	0.422	0.559	23.080	<0.0001
SOMA_P01160_NPPA_5443_62	2234	1.567	1.478	1.66	0.00E+00	51.14	1077	1.667	1.553	1.789	44.572	<0.0001
SOMA_Q6P988_NOTUM_8252_2	2234	0.637	0.6	0.675	0.00E+00	51.03	1077	0.388	0.316	0.476	18.767	<0.0001
SOMA_Q96GL9_FAM163A_6260_14	2234	1.578	1.486	1.676	0.00E+00	49.405	NA	NA	NA	NA	NA	NA

SOMA_Q2UY09_COL28A1_10702_1	2234	1.543	1.457	1.635	0.00E+00	48.747	1077	1.301	1.229	1.377	18.889	<0.0001
SOMA_P12111_COL6A3_11196_31	2234	1.543	1.457	1.635	0.00E+00	48.471	1077	1.201	1.140	1.265	11.282	<0.0001
SOMA_Q9UBX5_FBLN5_15585_304	2234	1.574	1.481	1.672	0.00E+00	48.025	1077	1.849	1.711	1.999	53.404	<0.0001
SOMA_Q9GZV9_FGF23_3807_1	2234	1.533	1.447	1.624	0.00E+00	47.356	1077	1.349	1.270	1.432	21.880	<0.0001
SOMA_P61769_B2M_3485_28	2234	1.534	1.448	1.624	0.00E+00	47.348	1077	1.240	1.177	1.307	15.015	<0.0001
SOMA_Q9BQT9_CLSTN3_6291_55	2234	1.525	1.441	1.614	0.00E+00	47.339	1077	1.281	1.205	1.362	14.792	<0.0001
SOMA_P55083_MFAP4_5636_10	2234	1.55	1.461	1.644	0.00E+00	47.249	1077	1.536	1.441	1.638	38.656	<0.0001
SOMA_P01034_CST3_2609_59	2234	1.527	1.442	1.617	0.00E+00	47.034	1077	1.330	1.257	1.407	22.276	<0.0001
SOMA_P11597_CETP_7131_207	2234	1.561	1.469	1.658	0.00E+00	46.504	NA	NA	NA	NA	NA	NA
SOMA_Q96GP6_SCARF2_8956_96	2234	1.502	1.42	1.587	0.00E+00	45.944	1077	1.426	1.342	1.515	29.881	<0.0001
SOMA_Q9H4D0_CLSTN2_18882_7	2234	1.543	1.453	1.64	0.00E+00	44.046	1077	1.372	1.312	1.435	42.438	<0.0001
SOMA_Q9H0R8_GABARAP_12661_44	2234	1.495	1.414	1.582	0.00E+00	44.032	1077	1.058	1.011	1.108	1.813	0.0154
SOMA_Q01995_TAGLN_15640_54	2234	1.503	1.419	1.592	0.00E+00	42.991	1077	1.290	1.221	1.362	19.100	<0.0001
SOMA_P39060_COL18A1_2201_17	2234	1.502	1.418	1.591	0.00E+00	42.977	1077	1.393	1.315	1.477	28.299	<0.0001
SOMA_Q9H772_GREM2_5598_3	2234	1.525	1.436	1.62	0.00E+00	42.369	1077	1.224	1.171	1.279	18.343	<0.0001
SOMA_P24821_TNC_4155_3	2234	1.526	1.437	1.621	0.00E+00	42.248	1077	1.689	1.568	1.820	42.713	<0.0001
SOMA_Q2I0M5_RSPO4_8464_31	2234	1.508	1.422	1.599	0.00E+00	42.247	1077	1.175	1.127	1.225	13.630	<0.0001
SOMA_P51888_PRELP_5675_6	2234	1.528	1.439	1.624	0.00E+00	42.128	NA	NA	NA	NA	NA	NA
SOMA_O95166_GABARAP_17735_130	2234	1.473	1.393	1.556	0.00E+00	41.99	1077	1.191	1.134	1.250	11.672	<0.0001

SOMA_Q12805_EFEMP1_8480_29	2234	1.509	1.422	1.601	0.00E+00	41.21	1077	1.321	1.255	1.390	25.678	<0.0001
SOMA_P07998_RNASE1_7211_2	2234	1.491	1.407	1.58	0.00E+00	41.168	1077	1.173	1.115	1.233	9.309	<0.0001
SOMA_Q8N2S1_LTBP4_13133_73	2234	1.509	1.42	1.602	0.00E+00	40.015	1077	1.686	1.562	1.820	40.081	<0.0001
SOMA_Q9BXJ1_C1QTNF1_6304_8	2234	1.492	1.406	1.583	0.00E+00	38.962	1077	1.338	1.261	1.419	21.457	<0.0001
SOMA_Q07654_TFF3_4721_54	2234	1.458	1.378	1.542	0.00E+00	38.582	1077	1.197	1.132	1.264	9.727	<0.0001
SOMA_P61916_NPC2_6259_60	2234	1.475	1.392	1.564	0.00E+00	38.384	NA	NA	NA	NA	NA	NA
SOMA_O94856_NFASC_7179_69	2234	1.501	1.412	1.595	0.00E+00	37.991	1077	1.264	1.194	1.337	15.256	<0.0001
SOMA_Q9BU40_CHRDL1_3362_61	2234	1.472	1.389	1.56	0.00E+00	37.924	1077	1.429	1.327	1.538	20.641	<0.0001
SOMA_P01011_SERPINA_4153_11	2234	1.478	1.393	1.567	0.00E+00	37.876	1077	1.309	1.246	1.376	25.468	<0.0001
SOMA_Q8WWZ8_OIT3_6296_36	2234	1.449	1.37	1.533	0.00E+00	37.745	1077	1.192	1.123	1.265	8.215	<0.0001
SOMA_P61626_LYZ_4920_10	2234	1.458	1.377	1.544	0.00E+00	37.607	1077	1.272	1.198	1.351	14.316	<0.0001
SOMA_Q14508_WFDC2_11388_75	2234	1.475	1.39	1.564	0.00E+00	37.584	1077	1.607	1.492	1.732	34.926	<0.0001
SOMA_Q92626_PXDN_13463_1	2234	1.463	1.381	1.551	0.00E+00	37.142	1077	1.202	1.142	1.266	11.498	<0.0001
SOMA_P17900_GM2A_15441_6	2234	1.462	1.38	1.55	0.00E+00	37.09	1077	1.242	1.176	1.311	14.123	<0.0001
SOMA_P52823_STC1_4930_21	2234	1.476	1.391	1.566	0.00E+00	37.059	1077	1.397	1.294	1.508	16.922	<0.0001
SOMA_P34096_RNASE4_5644_60	2234	1.455	1.374	1.54	0.00E+00	37.01	1077	1.657	1.521	1.805	30.186	<0.0001
SOMA_Q07654_TFF3_8323_163	2234	1.441	1.362	1.524	0.00E+00	36.686	1077	1.131	1.068	1.198	4.573	<0.0001
SOMA_P04070_PROC_2961_1	2234	0.681	0.642	0.723	0.00E+00	36.603	1077	0.678	0.611	0.753	12.380	<0.0001
SOMA_Q93091_RNASE6_5646_20	2234	1.452	1.371	1.538	0.00E+00	36.574	1077	1.151	1.099	1.205	8.709	<0.0001

SOMA_Q12841_FSTL1_13112_179	2234	1.478	1.391	1.57	0.00E+00	36.117	1077	1.700	1.578	1.831	43.647	<0.0001
SOMA_Q9Y5H3_PCDHGA1_6321_65	2234	1.454	1.372	1.541	0.00E+00	35.815	1077	1.030	0.972	1.092	0.502	0.315
SOMA_Q92743_HTRA1_15594_47	2234	1.463	1.378	1.553	0.00E+00	35.05	1077	1.447	1.346	1.555	23.118	<0.0001
SOMA_O75636_FCN3_5462_62	2234	0.708	0.671	0.748	0.00E+00	34.82	1077	0.556	0.497	0.623	23.421	<0.0001
SOMA_Q7LFX5_CHST15_4469_78	2234	1.475	1.387	1.568	0.00E+00	34.818	1077	1.499	1.400	1.605	30.315	<0.0001
SOMA_Q06141_REG3A_15304_1	2234	1.456	1.372	1.545	0.00E+00	34.711	1077	1.269	1.200	1.342	16.120	<0.0001
SOMA_P42167_TMPO_8265_225	2234	1.431	1.352	1.515	0.00E+00	34.508	1077	1.177	1.096	1.264	5.113	<0.0001
SOMA_P06276_BCHE_15514_26	2234	0.682	0.642	0.725	0.00E+00	34.346	1077	0.462	0.414	0.516	42.099	<0.0001
SOMA_Q02487_DSC2_13126_52	2234	1.445	1.363	1.532	0.00E+00	34.321	1077	1.170	1.111	1.233	8.433	<0.0001
SOMA_P40261_NNMT_19376_74	2234	1.437	1.357	1.523	0.00E+00	34.317	1077	1.042	0.980	1.108	0.723	0.1891
SOMA_P07108_DBI_16919_1	2234	1.429	1.349	1.514	0.00E+00	33.297	1077	1.116	1.029	1.209	2.105	0.0079
SOMA_O14786_NRP1_5542_22	2234	1.471	1.382	1.565	0.00E+00	33.233	1077	1.145	1.099	1.194	9.765	<0.0001
SOMA_P15090_FABP4_15386_7	2234	1.434	1.353	1.52	0.00E+00	33.025	1077	1.554	1.437	1.682	27.425	<0.0001
SOMA_Q16629_SRSF7_12987_12	2234	1.42	1.341	1.504	0.00E+00	32.638	1077	1.394	1.308	1.485	23.865	<0.0001
SOMA_P08709_F7_3184_25	2234	0.702	0.663	0.744	0.00E+00	32.255	1077	0.630	0.566	0.702	16.327	<0.0001
SOMA_P22223_CDH3_2643_57	2234	0.691	0.65	0.734	0.00E+00	32.102	1077	0.497	0.443	0.557	32.401	<0.0001
SOMA_P03973_SLPI_4413_3	2234	1.426	1.345	1.512	0.00E+00	31.833	1077	1.146	1.105	1.188	12.586	<0.0001
SOMA_P30050_RPL12_19183_164	2234	1.405	1.328	1.486	0.00E+00	31.814	1077	1.200	1.102	1.307	4.550	<0.0001
SOMA_P05413_FABP3_5437_63	2234	1.412	1.333	1.495	0.00E+00	31.49	1077	1.325	1.239	1.417	15.616	<0.0001

SOMA_Q01105_SET_5364_7	2234	0.7	0.66	0.743	0.00E+00	31.474	1077	0.651	0.585	0.726	14.148	<0.0001
SOMA_O00622_CYR61_6264_9	2234	1.397	1.321	1.478	0.00E+00	30.852	NA	NA	NA	NA	NA	NA
SOMA_Q99784_OLFM1_5703_26	2234	1.415	1.335	1.5	0.00E+00	30.82	1077	1.089	1.044	1.136	4.120	<0.0001
SOMA_P05452_CLEC3B_5701_81	2234	0.702	0.662	0.745	0.00E+00	30.777	1077	0.582	0.522	0.648	22.208	<0.0001
SOMA_P61956_SUMO2_19555_1	2234	1.413	1.333	1.497	0.00E+00	30.718	1077	1.149	1.091	1.211	6.723	<0.0001
SOMA_O60330_PCDHGA1_6938_21	2234	1.403	1.325	1.485	0.00E+00	30.595	1077	1.072	1.024	1.122	2.536	0.0029
SOMA_P07478_PRSS2_5034_79	2234	1.423	1.341	1.51	0.00E+00	30.538	1077	1.159	1.107	1.214	9.384	<0.0001
SOMA_P00747_PLG_3710_49	2234	0.7	0.659	0.743	0.00E+00	30.519	1077	0.592	0.529	0.663	19.096	<0.0001
SOMA_Q9BQI0_AIF1L_18871_24	2234	1.41	1.33	1.494	0.00E+00	30.453	1077	1.316	1.217	1.422	11.284	<0.0001
SOMA_Q01974_ROR2_7861_9	2234	1.41	1.33	1.494	0.00E+00	30.442	1077	1.164	1.104	1.227	7.823	<0.0001
SOMA_P45379_TNNT2_5315_22	2234	1.41	1.33	1.494	0.00E+00	30.438	1077	1.215	1.149	1.284	11.123	<0.0001
SOMA_Q13790_APOF_12370_30	2234	1.417	1.335	1.503	0.00E+00	30.202	1077	1.566	1.464	1.674	38.811	<0.0001
SOMA_Q01638_IL1RL1_4234_8	2234	1.437	1.351	1.528	0.00E+00	30.18	1077	1.399	1.328	1.474	35.867	<0.0001
SOMA_Q9NQ30_ESM1_3805_16	2234	1.433	1.348	1.524	0.00E+00	30.061	1077	1.378	1.302	1.460	27.328	<0.0001
SOMA_Q9NP99_TREM1_9266_1	2234	1.407	1.327	1.491	0.00E+00	29.973	1077	1.338	1.260	1.420	21.004	<0.0001
SOMA_P62995_TRA2B_12373_73	2234	1.38	1.306	1.458	0.00E+00	29.745	1077	1.144	1.094	1.196	8.596	<0.0001
SOMA_Q9Y5I4_PCDHAC2_9361_7	2234	1.393	1.317	1.475	0.00E+00	29.71	1077	1.054	0.993	1.117	1.087	0.0819
SOMA_O60760_HPGDS_12549_33	2234	0.72	0.681	0.762	0.00E+00	29.695	1077	0.539	0.459	0.634	13.159	<0.0001
SOMA_Q9UJJ9_GNPTG_10666_7	2234	1.407	1.327	1.492	0.00E+00	29.632	1077	1.330	1.247	1.419	17.244	<0.0001

SOMA_O00244_ATOX1_19233_75	2234	1.402	1.323	1.485	0.00E+00	29.553	1077	1.080	1.033	1.128	3.173	0.0007
SOMA_Q9BX93_PLA2G12_9380_2	2234	0.705	0.664	0.749	0.00E+00	29.53	1077	0.486	0.421	0.561	22.231	<0.0001
SOMA_P58335_ANTXR2_15559_5	2234	0.717	0.677	0.759	0.00E+00	29.157	1077	0.601	0.534	0.677	16.452	<0.0001
SOMA_Q9HC57_WFDC1_9316_67	2234	1.388	1.311	1.469	0.00E+00	28.798	1077	1.416	1.329	1.509	26.257	<0.0001
SOMA_Q9GZX9_TWSG1_9234_8	2234	1.402	1.322	1.487	0.00E+00	28.797	1077	1.284	1.208	1.365	15.020	<0.0001
SOMA_Q9BWP8_COLEC11_4430_44	2234	1.42	1.336	1.51	0.00E+00	28.356	1077	1.264	1.190	1.343	13.507	<0.0001
SOMA_Q16663_CCL15_18289_16	2234	1.384	1.307	1.465	0.00E+00	28.352	1077	1.459	1.358	1.568	24.039	<0.0001
SOMA_P19429_TNNI3_5441_67	2234	1.379	1.303	1.458	0.00E+00	28.351	1077	1.013	0.935	1.097	0.123	0.7528
SOMA_P52943_CRIP2_9053_16	2234	1.387	1.309	1.469	0.00E+00	28.014	1077	1.066	1.015	1.118	1.994	0.0101
SOMA_O95393_BMP10_3587_53	2234	1.37	1.296	1.449	0.00E+00	27.782	1077	1.229	1.154	1.309	9.859	<0.0001
SOMA_P18065_IGFBP2_8469_41	2234	1.4	1.319	1.486	0.00E+00	27.777	1077	1.562	1.451	1.682	31.437	<0.0001
SOMA_P00533_EGFR_2677_1	2234	0.723	0.682	0.765	0.00E+00	27.684	1077	0.702	0.633	0.778	10.755	<0.0001
SOMA_Q9HAV5_EDA2R_3083_71	2234	1.368	1.294	1.447	0.00E+00	27.607	1077	1.106	1.056	1.159	4.726	<0.0001
SOMA_Q8WWX9_SELM_15336_7	2234	1.382	1.305	1.464	0.00E+00	27.598	NA	NA	NA	NA	NA	NA
SOMA_O75015_FCGR3B_3311_27	2234	1.399	1.318	1.485	0.00E+00	27.591	1077	1.455	1.358	1.560	25.483	<0.0001
SOMA_Q14512_FGFBP1_15494_11	2234	1.405	1.322	1.493	0.00E+00	27.379	1077	1.228	1.152	1.308	9.553	<0.0001
SOMA_Q13308_PTK7_9525_1	2234	1.394	1.314	1.479	0.00E+00	27.348	1077	1.610	1.489	1.740	32.476	<0.0001
SOMA_Q9BY76_ANGPTL4_3796_79	2234	1.378	1.301	1.459	0.00E+00	27.175	1077	1.235	1.168	1.306	12.802	<0.0001
SOMA_Q9Y5C1_ANGPTL3_10391_1	2234	1.381	1.303	1.463	0.00E+00	27.148	1077	1.360	1.250	1.480	12.010	<0.0001

SOMA_Q9UNI1_CELA1_6107_3	2234	1.398	1.317	1.485	0.00E+00	27.075	1077	1.658	1.518	1.812	28.322	<0.0001
SOMA_Q03405_PLAUR_2652_15	2234	1.35	1.279	1.424	0.00E+00	26.97	1077	1.456	1.360	1.559	26.607	<0.0001
SOMA_Q9NVD7_PARVA_13434_172	2234	1.366	1.291	1.446	0.00E+00	26.665	1077	1.079	1.034	1.127	3.252	0.0006
SOMA_O60938_KERA_10758_2	2234	1.403	1.32	1.492	0.00E+00	26.566	1077	1.455	1.379	1.534	42.897	<0.0001
SOMA_O43251_RBFOX2_11462_8	2234	1.363	1.289	1.442	0.00E+00	26.449	1077	1.033	0.975	1.094	0.569	0.27
SOMA_P09529_INHBB_13676_46	2234	1.386	1.306	1.47	0.00E+00	26.43	1077	1.236	1.177	1.297	17.069	<0.0001
SOMA_P21757_MSR1_15533_97	2234	1.375	1.298	1.458	0.00E+00	26.222	1077	1.362	1.266	1.466	15.967	<0.0001
SOMA_Q8NBM8_PCYOX1L_5599_88	2234	1.372	1.295	1.453	0.00E+00	26.144	1077	1.080	1.020	1.143	2.103	0.0079
SOMA_Q01844_EWSR1_12988_49	2234	1.366	1.29	1.446	0.00E+00	26.105	1077	1.269	1.187	1.356	11.667	<0.0001
SOMA_O60462_NRP2_15387_44	2234	1.385	1.305	1.471	0.00E+00	25.726	1077	1.703	1.575	1.841	39.819	<0.0001
SOMA_P07858_CTSB_8007_19	2234	1.385	1.304	1.471	0.00E+00	25.542	1077	1.534	1.434	1.640	34.872	<0.0001
SOMA_O95445_APOM_10445_20	2234	0.726	0.684	0.771	0.00E+00	25.312	1077	0.593	0.491	0.715	7.314	<0.0001
SOMA_Q86Y30_BAGE2_6294_11	2234	1.36	1.284	1.44	0.00E+00	25.285	1077	1.096	1.021	1.176	1.947	0.0113
SOMA_O15013_ARHGEF1_9061_3	2234	0.734	0.693	0.777	0.00E+00	25.272	1077	0.555	0.419	0.736	4.355	<0.0001
SOMA_P06401_PGR_15693_9	2234	1.363	1.286	1.444	0.00E+00	25.141	1077	1.094	0.993	1.205	1.163	0.0687
SOMA_Q9BUD6_SPON2_8099_42	2234	1.378	1.298	1.463	0.00E+00	25.131	1077	1.580	1.462	1.707	30.131	<0.0001
SOMA_Q9NR28_DIABLO_3122_6	2234	1.364	1.287	1.446	0.00E+00	24.818	1077	1.050	0.985	1.119	0.880	0.1317
SOMA_P09455_RBP1_19279_42	2234	1.358	1.282	1.438	0.00E+00	24.732	1077	1.036	0.980	1.095	0.669	0.2144
SOMA_Q8NBJ4_GOLM1_17456_53	2234	1.368	1.29	1.452	0.00E+00	24.725	1077	1.265	1.208	1.324	23.037	<0.0001

SOMA_P41271_NBL1_2944_66	2234	1.364	1.287	1.446	0.00E+00	24.719	1077	1.149	1.091	1.210	6.785	<0.0001
SOMA_P56704_WNT3A_13236_25	2234	0.743	0.702	0.785	0.00E+00	24.7	1077	0.542	0.459	0.640	12.281	<0.0001
SOMA_Q96EP1_CHFR_11320_29	2234	0.736	0.695	0.78	0.00E+00	24.552	1077	0.423	0.330	0.541	11.087	<0.0001
SOMA_Q9C002_NMES1_6406_3	2234	0.721	0.678	0.767	0.00E+00	24.458	1077	0.616	0.526	0.723	8.577	<0.0001
SOMA_P82980_RBP5_19241_31	2234	1.361	1.284	1.443	0.00E+00	24.44	1077	1.345	1.259	1.437	17.964	<0.0001
SOMA_P11362_FGFR1_5532_53	2234	1.374	1.294	1.46	0.00E+00	24.418	1077	1.396	1.317	1.479	28.617	<0.0001
SOMA_P08949_NMB_9321_400	2234	1.36	1.283	1.441	0.00E+00	24.404	1077	1.089	1.040	1.141	3.588	0.0003
SOMA_Q8TC05_MDM1_7898_29	2234	1.354	1.279	1.434	0.00E+00	24.36	1077	1.015	0.937	1.099	0.144	0.7183
SOMA_P08670_VIM_15540_6	2234	1.336	1.264	1.411	0.00E+00	24.311	1077	1.072	0.994	1.156	1.144	0.0718
SOMA_Q8IZS8_CACNA2D_8885_6	2234	0.743	0.702	0.786	0.00E+00	24.268	1077	0.658	0.582	0.743	10.694	<0.0001
SOMA_P52798_EFNA4_2614_28	2234	1.355	1.279	1.436	0.00E+00	24.209	1077	1.153	1.090	1.220	6.111	<0.0001
SOMA_P22466_GAL_13389_8	2234	0.754	0.715	0.796	0.00E+00	24.206	NA	NA	NA	NA	NA	NA
SOMA_Q13145_BAMBI_8811_24	2234	1.348	1.273	1.427	0.00E+00	24.084	1077	1.108	1.042	1.179	2.942	0.0011
SOMA_P35858_IGFALS_6605_17	2234	0.735	0.693	0.78	0.00E+00	24.082	1077	0.671	0.607	0.742	14.176	<0.0001
SOMA_P18440_NAT1_12632_14	2234	0.732	0.69	0.777	0.00E+00	24.009	1077	0.486	0.402	0.588	12.931	<0.0001
SOMA_Q9ULB5_CDH7_7959_34	2234	0.747	0.707	0.79	0.00E+00	23.993	1077	0.427	0.349	0.522	16.006	<0.0001
SOMA_Q8IZT8_HS3ST5_10731_10	2234	0.729	0.687	0.775	0.00E+00	23.916	1077	0.358	0.261	0.493	9.576	<0.0001
SOMA_Q96DZ1_ERLEC1_8957_72	2234	1.347	1.273	1.427	0.00E+00	23.817	1077	1.105	1.040	1.175	2.891	0.0013
SOMA_Q8WXI7_MUC16_15565_102	2234	1.358	1.28	1.44	0.00E+00	23.771	1077	1.074	1.033	1.117	3.437	0.0004

SOMA_Q93070_ART4_6576_1	2234	1.37	1.289	1.455	0.00E+00	23.722	1077	1.352	1.276	1.431	24.202	<0.0001
SOMA_P49755_TMED10_6506_54	2234	1.349	1.274	1.429	0.00E+00	23.696	1077	1.156	1.093	1.223	6.448	<0.0001
SOMA_O15525_MAFG_19281_86	2234	1.343	1.269	1.421	0.00E+00	23.576	1077	1.072	1.020	1.127	2.224	0.006
SOMA_P10912_GHR_2948_58	2234	0.736	0.694	0.781	0.00E+00	23.56	1077	0.578	0.512	0.653	18.044	<0.0001
SOMA_Q6IN85_SMEK1_10866_60	2234	1.348	1.272	1.428	0.00E+00	23.471	NA	NA	NA	NA	NA	NA
SOMA_Q9BUJ0_ABHD14A_5715_4	2234	1.341	1.267	1.419	0.00E+00	23.467	1077	1.048	0.966	1.137	0.587	0.2589
SOMA_O43915_FIGF_13098_93	2234	1.343	1.269	1.422	0.00E+00	23.38	NA	NA	NA	NA	NA	NA
SOMA_Q15485_FCN2_13717_15	2234	0.741	0.699	0.785	0.00E+00	23.322	1077	0.787	0.710	0.873	5.223	<0.0001
SOMA_Q9Y240_CLEC11A_4500_50	2234	1.369	1.288	1.455	0.00E+00	23.221	1077	1.416	1.320	1.519	21.409	<0.0001
SOMA_P98160_HSPG2_15626_223	2234	1.363	1.283	1.447	0.00E+00	23.199	1077	1.169	1.130	1.209	18.664	<0.0001
SOMA_P08253_MMP2_4160_49	2234	1.36	1.28	1.444	0.00E+00	22.964	1077	1.615	1.499	1.740	35.597	<0.0001
SOMA_Q9H4F8_SMOC1_13118_5	2234	1.355	1.276	1.438	0.00E+00	22.821	1077	1.291	1.211	1.376	14.213	<0.0001
SOMA_O00453_LST1_9531_24	2234	1.34	1.265	1.419	0.00E+00	22.801	1077	1.223	1.135	1.317	6.888	<0.0001
SOMA_P15907_ST6GAL1_6035_2	2234	1.33	1.258	1.407	0.00E+00	22.716	1077	1.070	0.993	1.152	1.131	0.074
SOMA_P54826_GAS1_5463_22	2234	1.348	1.271	1.43	0.00E+00	22.593	1077	1.371	1.281	1.467	19.191	<0.0001
SOMA_O75356_ENTPD5_4437_56	2234	0.744	0.702	0.789	0.00E+00	22.563	1077	0.572	0.507	0.644	19.298	<0.0001
SOMA_P06127_CD5_5596_75	2234	1.362	1.281	1.448	0.00E+00	22.477	1077	1.440	1.335	1.554	20.425	<0.0001
SOMA_P28799_GRN_4992_49	2234	1.357	1.278	1.442	0.00E+00	22.422	1077	1.383	1.291	1.481	19.546	<0.0001
SOMA_P07333_CSF1R_13682_47	2234	1.351	1.273	1.435	0.00E+00	22.261	1077	1.395	1.292	1.506	16.735	<0.0001

SOMA_Q9NS68_TNFRSF1_5131_15	2234	1.331	1.258	1.41	0.00E+00	22.108	1077	1.127	1.067	1.190	4.731	<0.0001
SOMA_Q9UHD0_IL19_3035_80	2234	1.36	1.279	1.446	0.00E+00	22.108	1077	1.420	1.336	1.509	28.521	<0.0001
SOMA_P10145_CXCL8_3447_64	2234	1.349	1.271	1.432	0.00E+00	21.952	1077	1.129	1.080	1.179	7.227	<0.0001
SOMA_O75056_SDC3_16612_28	2234	0.745	0.703	0.79	0.00E+00	21.95	1077	0.428	0.353	0.520	16.869	<0.0001
SOMA_Q9Y5G2_PCDHGB2_10803_22	2234	1.339	1.263	1.42	0.00E+00	21.943	NA	NA	NA	NA	NA	NA
SOMA_Q9NZV1_CRIM1_15492_1	2234	1.323	1.25	1.399	0.00E+00	21.72	1077	1.006	0.928	1.091	0.055	0.8814
SOMA_Q12907_LMAN2_7638_30	2234	0.735	0.69	0.782	0.00E+00	21.669	1077	0.504	0.427	0.596	15.053	<0.0001
SOMA_P20333_TNFRSF1_8368_102	2234	1.34	1.263	1.421	0.00E+00	21.632	1077	1.343	1.266	1.424	22.246	<0.0001
SOMA_Q14162_SCARF1_5129_12	2234	1.337	1.261	1.418	0.00E+00	21.608	1077	1.139	1.047	1.240	2.627	0.0024
SOMA_Q9NTX7_RNF146_11401_181	2234	0.757	0.715	0.8	0.00E+00	21.531	1077	1.115	1.038	1.197	2.562	0.0027
SOMA_Q86Y82_STX12_10418_36	2234	1.326	1.253	1.404	0.00E+00	21.519	1077	1.025	0.932	1.127	0.215	0.6101
SOMA_P20333_TNFRSF1_3152_57	2234	1.337	1.261	1.418	0.00E+00	21.513	1077	1.287	1.209	1.370	14.671	<0.0001
SOMA_Q86VW1_SLC22A1_9969_8	2234	0.756	0.714	0.8	0.00E+00	21.352	1077	0.505	0.436	0.584	19.186	<0.0001
SOMA_P19438_TNFRSF1_2654_19	2234	1.335	1.259	1.416	0.00E+00	21.336	1077	1.297	1.226	1.372	18.704	<0.0001
SOMA_P40121_CAPG_4968_50	2234	1.33	1.255	1.409	0.00E+00	21.282	1077	1.241	1.163	1.323	10.329	<0.0001
SOMA_P48304_REG1B_16770_3	2234	1.334	1.258	1.415	0.00E+00	21.28	1077	1.330	1.244	1.421	16.444	<0.0001
SOMA_P01588_EPO_5813_58	2234	1.351	1.271	1.436	0.00E+00	21.257	1077	1.249	1.193	1.308	20.790	<0.0001
SOMA_P0DJI8_SAA1_15515_2	2234	1.329	1.255	1.409	0.00E+00	21.208	1077	1.404	1.316	1.498	24.154	<0.0001
SOMA_Q8TDQ0_HAVCR2_5134_52	2234	1.335	1.258	1.416	0.00E+00	21.091	1077	1.373	1.285	1.468	20.137	<0.0001

SOMA_Q9UNK0_STX8_10903_50	2234	1.324	1.25	1.402	0.00E+00	20.934	1077	0.986	0.892	1.089	0.111	0.7745
SOMA_Q9Y5P4_COL4A3B_13950_9	2234	1.32	1.247	1.398	0.00E+00	20.915	NA	NA	NA	NA	NA	NA
SOMA_Q06033_ITIH3_7145_1	2234	1.332	1.256	1.413	0.00E+00	20.864	1077	1.440	1.354	1.532	30.444	<0.0001
SOMA_P80188_LCN2_2836_68	2234	1.329	1.253	1.409	0.00E+00	20.856	1077	1.153	1.097	1.211	7.708	<0.0001
SOMA_P35968_KDR_3651_50	2234	0.755	0.713	0.8	0.00E+00	20.812	1077	0.822	0.739	0.915	3.484	0.0003
SOMA_P21815_IBSP_3415_61	2234	1.346	1.266	1.431	0.00E+00	20.803	1077	1.394	1.323	1.469	34.415	<0.0001
SOMA_Q16666_IFI16_13940_19	2234	1.309	1.238	1.383	0.00E+00	20.69	1077	1.069	0.986	1.158	0.984	0.1037
SOMA_P05451_REG1A_13095_51	2234	1.329	1.253	1.409	0.00E+00	20.612	1077	1.169	1.102	1.240	6.658	<0.0001
SOMA_Q9UKP5_ADAMTS6_6441_62	2234	1.317	1.244	1.395	0.00E+00	20.51	1077	1.069	0.982	1.163	0.907	0.1238
SOMA_Q9Y240_CLEC11A_2966_65	2234	1.342	1.263	1.427	0.00E+00	20.5	1077	1.408	1.316	1.507	22.431	<0.0001
SOMA_Q9HCZ1_ZNF334_12763_69	2234	0.763	0.721	0.807	0.00E+00	20.45	1077	0.795	0.502	1.261	0.482	0.3298
SOMD P01876 IGHA1 I 110897 PASS	2234	1.32	1.246	1.398	0.00E+00	20.376	NA	NA	NA	NA	NA	NA
SOMD P01877 IGHA1 I 110897 PASS	2234	1.32	1.246	1.398	0.00E+00	20.376	NA	NA	NA	NA	NA	NA
SOMA_P12110_COL6A2_16753_46	2234	1.315	1.242	1.392	0.00E+00	20.373	1077	1.141	1.098	1.184	11.014	<0.0001
SOMA_Q9UHX1_PUF60_10575_31	2234	1.339	1.26	1.423	0.00E+00	20.318	1077	1.210	1.158	1.264	16.953	<0.0001
SOMA_Q96RJ3_TNFRSF1_5383_14	2234	0.749	0.705	0.795	0.00E+00	20.259	1077	1.019	0.946	1.098	0.210	0.6166
SOMA_P19827_ITIH1_7955_195	2234	0.752	0.709	0.798	0.00E+00	20.239	1077	0.590	0.536	0.650	26.093	<0.0001
SOMA_Q99969_RARRES2_3079_62	2234	1.317	1.244	1.395	0.00E+00	20.215	1077	1.329	1.213	1.457	8.901	<0.0001
SOMA_Q9NR71_ASAH2_3212_30	2234	0.754	0.711	0.8	0.00E+00	20.091	1077	0.635	0.552	0.729	9.889	<0.0001

SOMA_P51884_LUM_13114_50	2234	1.322	1.247	1.402	0.00E+00	20.035	1077	1.299	1.209	1.396	12.037	<0.0001
SOMA_Q2MKA7_RSPO1_16614_27	2234	1.334	1.255	1.417	0.00E+00	19.997	1077	1.275	1.200	1.355	14.334	<0.0001
SOMA_Q9Y5H2_PCDHGA1_6563_78	2234	1.324	1.248	1.405	0.00E+00	19.972	NA	NA	NA	NA	NA	NA
SOMA_P14555_PLA2G2A_2692_74	2234	1.325	1.249	1.406	0.00E+00	19.965	1077	1.228	1.165	1.294	13.671	<0.0001
SOMA_P16035_TIMP2_2278_61	2234	1.327	1.251	1.409	0.00E+00	19.908	1077	1.583	1.464	1.712	29.744	<0.0001
SOMA_Q9UHV9_PFDN2_19243_2	2234	1.31	1.237	1.387	0.00E+00	19.871	1077	1.131	1.077	1.188	6.032	<0.0001
SOMA_Q9H3U7_SMOC2_15635_4	2234	1.32	1.245	1.4	0.00E+00	19.808	1077	1.225	1.160	1.294	12.647	<0.0001
SOMA_P31415_CASQ1_11263_57	2234	0.753	0.709	0.799	0.00E+00	19.781	1077	0.704	0.615	0.806	6.471	<0.0001
SOMA_Q5TAT6_COL13A1_6570_1	2234	0.754	0.71	0.801	0.00E+00	19.68	1077	0.736	0.633	0.855	4.199	<0.0001
SOMA_P84090_ERH_11614_29	2234	1.303	1.232	1.378	0.00E+00	19.672	1077	1.069	0.986	1.159	0.974	0.1062
SOMA_Q7L1S5_CHST9_11646_4	2234	1.317	1.243	1.397	0.00E+00	19.672	1077	1.039	0.980	1.102	0.694	0.2025
SOMA_Q7Z2W9_MRPL21_8942_2	2234	0.755	0.712	0.802	0.00E+00	19.661	1077	0.688	0.587	0.807	5.377	<0.0001
SOMA_P07339_CTSD_5508_62	2234	1.315	1.241	1.394	0.00E+00	19.639	1077	1.073	1.014	1.135	1.842	0.0144
SOMA_Q9H665_IGFLR1_7244_16	2234	1.313	1.239	1.391	0.00E+00	19.618	1077	1.116	1.054	1.181	3.814	0.0002
SOMA_P43652_AFM_4763_31	2234	0.756	0.712	0.802	0.00E+00	19.613	1077	0.602	0.544	0.665	22.239	<0.0001
SOMA_Q96R05_RBP7_14208_3	2234	1.317	1.242	1.396	0.00E+00	19.591	1077	1.434	1.336	1.539	22.667	<0.0001
SOMA_P00742_F10_3077_66	2234	0.766	0.723	0.81	0.00E+00	19.584	1077	0.802	0.726	0.886	4.898	<0.0001
SOMA_P07911_UMOD_9451_20	2234	1.319	1.244	1.399	0.00E+00	19.536	1077	1.400	1.289	1.520	14.868	<0.0001
SOMA_Q16842_ST3GAL2_6281_51	2234	1.309	1.236	1.386	0.00E+00	19.529	1077	1.048	0.963	1.140	0.556	0.2782

SOMA_P00742_F10_4878_3	2234	0.766	0.724	0.811	0.00E+00	19.517	1077	0.807	0.731	0.890	4.703	<0.0001
SOMA_Q9H3T3_SEMA6B_5121_3	2234	1.333	1.254	1.418	0.00E+00	19.469	1077	1.309	1.240	1.382	21.479	<0.0001
SOMA_Q9BXJ4_C1QTNF3_7251_64	2234	0.758	0.715	0.804	0.00E+00	19.437	1077	0.453	0.382	0.538	18.708	<0.0001
SOMA_P07949_RET_3220_40	2234	0.76	0.717	0.806	0.00E+00	19.375	1077	0.704	0.582	0.850	3.575	0.0003
SOMA_O95150_TNFSF15_2968_61	2234	1.307	1.234	1.384	0.00E+00	19.358	1077	1.181	1.119	1.247	8.712	<0.0001
SOMA_Q15768_EFNB3_2514_65	2234	1.299	1.228	1.373	0.00E+00	19.29	1077	1.135	1.073	1.199	5.105	<0.0001
SOMA_Q9NXW2_DNAJB12_8006_12	2234	1.302	1.231	1.378	0.00E+00	19.18	1077	1.067	1.022	1.113	2.511	0.0031
SOMA_Q03403_TFF2_9191_8	2234	1.321	1.245	1.403	0.00E+00	19.148	1077	1.273	1.197	1.354	13.862	<0.0001
SOMA_P03952_KLKB1_4152_58	2234	0.761	0.718	0.807	0.00E+00	19.131	1077	0.607	0.547	0.674	19.958	<0.0001
SOMD P07942 LAMA2 L 18347 15 PASS	2234	1.325	1.247	1.408	0.00E+00	19.103	NA	NA	NA	NA	NA	NA
SOMD P11047 LAMA2 L 18347 15 PASS	2234	1.325	1.247	1.408	0.00E+00	19.103	NA	NA	NA	NA	NA	NA
SOMD P24043 LAMA2 L 18347 15 PASS	2234	1.325	1.247	1.408	0.00E+00	19.103	NA	NA	NA	NA	NA	NA
SOMA_Q09028_RBBP4_15331_47	2234	1.306	1.233	1.384	0.00E+00	19.094	1077	1.130	1.052	1.213	3.118	0.0008
SOMA_P49961_ENTPD1_7999_23	2234	1.317	1.241	1.398	0.00E+00	19.079	1077	1.101	1.057	1.147	5.442	<0.0001
SOMA_Q92874_DNASE1L_6324_11	2234	1.319	1.243	1.4	0.00E+00	19.068	1077	1.056	1.004	1.111	1.468	0.0341
SOMA_Q15485_FCN2_3313_21	2234	0.765	0.723	0.811	0.00E+00	19.045	1077	0.922	0.828	1.026	0.862	0.1376
SOMA_Q08999_RBL2_13565_2	2234	1.301	1.23	1.377	0.00E+00	19.04	1077	1.346	1.261	1.437	18.439	<0.0001
SOMA_Q13126_MTAP_9910_9	2234	0.767	0.724	0.812	0.00E+00	19.007	1077	0.206	0.128	0.332	10.138	<0.0001
SOMA_P13942_COL11A2_11278_4	2234	0.758	0.714	0.805	0.00E+00	18.991	1077	0.719	0.633	0.817	6.361	<0.0001

SOMA_Q9H5V8_CDCP1_16818_200	2234	1.304	1.231	1.38	0.00E+00	18.966	1077	1.298	1.206	1.396	11.535	<0.0001
SOMA_Q14392_LRRC32_7551_33	2234	1.314	1.239	1.394	0.00E+00	18.89	1077	1.034	0.980	1.092	0.654	0.2216
SOMA_Q96T51_RUFY1_11425_31	2234	0.766	0.723	0.811	0.00E+00	18.852	1077	0.595	0.482	0.736	5.807	<0.0001
SOMA_Q15848_ADIPOQ_3554_24	2234	1.319	1.242	1.4	0.00E+00	18.848	1077	1.625	1.506	1.754	35.131	<0.0001
SOMA_Q96EE4_CCDC126_6388_21	2234	0.765	0.722	0.811	0.00E+00	18.842	1077	0.294	0.182	0.477	6.185	<0.0001
SOMA_Q99727_TIMP4_6462_12	2234	1.307	1.233	1.385	0.00E+00	18.766	1077	1.234	1.151	1.323	8.421	<0.0001
SOMA_P10082_PYY_3727_35	2234	1.31	1.235	1.389	0.00E+00	18.715	1077	1.384	1.281	1.495	15.875	<0.0001
SOMA_Q96PQ1_SIGLEC1_8352_26	2234	0.754	0.709	0.802	0.00E+00	18.682	1077	0.594	0.521	0.679	13.861	<0.0001
SOMA_Q8TDY8_IGDCC4_9793_145	2234	0.761	0.717	0.808	0.00E+00	18.64	1077	0.894	0.733	1.090	0.572	0.2681
SOMA_Q9Y4K0_LOXL2_6504_65	2234	1.296	1.225	1.371	0.00E+00	18.615	1077	1.125	1.069	1.183	5.246	<0.0001
SOMA_P23280_CA6_3352_80	2234	0.76	0.716	0.807	0.00E+00	18.545	1077	0.652	0.568	0.748	9.025	<0.0001
SOMA_P22004_BMP6_8459_10	2234	1.313	1.237	1.394	0.00E+00	18.509	1077	1.579	1.469	1.697	34.825	<0.0001
SOMA_P45877_PPIC_18819_21	2234	1.295	1.224	1.371	0.00E+00	18.382	1077	1.235	1.152	1.324	8.512	<0.0001
SOMA_P15056_BRAF_15669_7	2234	0.797	0.758	0.838	0.00E+00	18.312	1077	0.843	0.727	0.979	1.604	0.0249
SOMA_Q8WU39_PACAP_16322_10	2234	1.304	1.23	1.382	0.00E+00	18.303	NA	NA	NA	NA	NA	NA
SOMA_P02778_CXCL10_4141_79	2234	1.293	1.222	1.368	0.00E+00	18.263	1077	1.153	1.095	1.214	7.137	<0.0001
SOMA_Q5EE01_CENPW_8864_59	2234	0.771	0.728	0.816	0.00E+00	18.251	1077	0.986	0.883	1.102	0.094	0.8058
SOMA_Q14195_DPYSL3_12707_26	2234	0.763	0.719	0.81	0.00E+00	18.226	1077	0.830	0.736	0.937	2.600	0.0025
SOMA_Q6NSJ0_KIAA116_8068_43	2234	0.777	0.735	0.822	0.00E+00	18.184	NA	NA	NA	NA	NA	NA

SOMA_P55058_PLTP_15475_4	2234	1.319	1.24	1.402	0.00E+00	18.097	1077	1.392	1.297	1.495	19.183	<0.0001
SOMA_P14210_HGF_2681_23	2234	1.302	1.228	1.38	0.00E+00	18.052	1077	1.212	1.138	1.290	8.735	<0.0001
SOMA_Q12907_LMAN2_9468_8	2234	1.307	1.232	1.387	0.00E+00	18.026	1077	1.239	1.171	1.310	13.067	<0.0001
SOMA_P35318_ADM_14115_34	2234	0.767	0.723	0.814	0.00E+00	18.002	1077	0.612	0.529	0.709	10.350	<0.0001
SOMD P06732 CKB CKM 3714 49 PASS	2234	0.767	0.723	0.814	0.00E+00	17.967	NA	NA	NA	NA	NA	NA
SOMD P12277 CKB CKM 3714 49 PASS	2234	0.767	0.723	0.814	0.00E+00	17.967	NA	NA	NA	NA	NA	NA
SOMA_Q6NUJ2_C11orf8_11116_16	2234	0.77	0.727	0.816	0.00E+00	17.947	1077	0.694	0.629	0.766	12.392	<0.0001
SOMA_Q9NRN5_OLFML3_8660_5	2234	1.305	1.23	1.385	0.00E+00	17.937	1077	1.269	1.151	1.399	5.786	<0.0001
SOMA_Q9HB63_NTN4_3327_27	2234	1.324	1.244	1.409	0.00E+00	17.878	1077	1.317	1.255	1.382	28.665	<0.0001
SOMA_P0DJI9_SAA2_18832_65	2234	1.296	1.223	1.373	0.00E+00	17.811	1077	1.189	1.131	1.250	11.046	<0.0001
SOMA_Q9BQI7_PSD2_9118_7	2234	0.765	0.721	0.813	0.00E+00	17.797	1077	0.547	0.395	0.758	3.551	0.0003
SOMA_P22748_CA4_14069_61	2234	0.77	0.726	0.816	0.00E+00	17.77	1077	0.769	0.490	1.206	0.598	0.2525
SOMA_O14791_APOL1_11510_31	2234	0.765	0.72	0.812	0.00E+00	17.741	1077	0.719	0.646	0.800	8.874	<0.0001
SOMA_O75462_CRLF1_14747_9	2234	1.298	1.224	1.375	0.00E+00	17.733	1077	1.073	1.011	1.138	1.691	0.0204
SOMA_Q02818_NUCB1_10451_11	2234	1.3	1.226	1.378	0.00E+00	17.731	1077	1.123	1.079	1.169	7.811	<0.0001
SOMA_P35442_THBS2_14111_15	2234	1.291	1.219	1.367	0.00E+00	17.721	1077	1.007	0.923	1.098	0.054	0.8823
SOMA_O75339_CILP_5717_2	2234	1.287	1.217	1.362	0.00E+00	17.712	1077	1.007	0.911	1.113	0.049	0.8927
SOMA_Q14116_IL18_5661_15	2234	1.288	1.217	1.364	0.00E+00	17.671	1077	1.101	1.044	1.162	3.352	0.0004
SOMA_P16152_CBR1_12381_26	2234	1.295	1.222	1.372	0.00E+00	17.665	1077	1.323	1.238	1.413	15.961	<0.0001

SOMA_P05305_EDN1_6495_14	2234	1.285	1.214	1.359	0.00E+00	17.613	1077	1.060	1.007	1.116	1.571	0.0268
SOMA_Q9UKK6_NXT1_9942_2	2234	1.292	1.22	1.368	0.00E+00	17.575	1077	1.074	1.010	1.141	1.655	0.0221
SOMA_O75344_FKBP6_12529_32	2234	0.769	0.725	0.816	0.00E+00	17.52	1077	0.770	0.617	0.962	1.670	0.0214
SOMA_Q76LX8_ADAMTS1_3175_51	2234	0.768	0.724	0.815	0.00E+00	17.517	1077	0.668	0.599	0.744	12.598	<0.0001
SOMA_O95715_CXCL14_5730_60	2234	1.288	1.217	1.364	0.00E+00	17.512	1077	1.087	0.999	1.183	1.288	0.0515
SOMA_P09958_FURIN_6276_16	2234	1.3	1.226	1.38	0.00E+00	17.467	1077	1.017	0.948	1.091	0.191	0.644
SOMA_Q9UBP4_DKK3_3607_71	2234	1.302	1.227	1.382	0.00E+00	17.437	1077	1.231	1.167	1.297	13.779	<0.0001
SOMA_Q8N257_HIST3H2_18823_52	2234	1.279	1.21	1.353	0.00E+00	17.429	NA	NA	NA	NA	NA	NA
SOMA_Q76M96_CCDC80_3234_23	2234	1.298	1.223	1.376	0.00E+00	17.356	1077	1.158	1.085	1.237	4.917	<0.0001
SOMA_Q12904_AIMP1_2714_78	2234	0.785	0.743	0.829	0.00E+00	17.302	1077	0.195	0.124	0.307	11.732	<0.0001
SOMA_Q9UMF0_ICAM5_8245_27	2234	1.299	1.224	1.378	0.00E+00	17.232	1077	1.249	1.164	1.339	9.279	<0.0001
SOMA_Q96D42_HAVCR1_9021_1	2234	1.287	1.215	1.363	0.00E+00	17.167	1077	1.039	0.971	1.112	0.569	0.2696
SOMA_Q8TAT2_FGFBP3_11219_95	2234	1.308	1.23	1.39	0.00E+00	17.166	1077	1.249	1.182	1.320	14.444	<0.0001
SOMA_P29317_EPHA2_4834_61	2234	1.295	1.221	1.373	0.00E+00	17.149	1077	1.248	1.182	1.317	15.011	<0.0001
SOMA_P0DMV9_HSPA1B_18901_26	2234	1.303	1.227	1.384	0.00E+00	17.135	1077	1.361	1.258	1.473	13.670	<0.0001
SOMA_Q8WVN6_SECTM1_13093_6	2234	1.298	1.223	1.377	0.00E+00	17.128	1077	1.081	1.018	1.147	1.968	0.0108
SOMA_P14543_NID1_3213_65	2234	1.296	1.222	1.375	0.00E+00	17.098	1077	1.441	1.329	1.562	18.087	<0.0001
SOMA_O75473_LGR5_16296_43	2234	0.78	0.737	0.825	0.00E+00	17.011	1077	0.858	0.543	1.355	0.292	0.5111
SOMA_Q8NBS9_TXNDC5_11212_7	2234	1.291	1.218	1.368	0.00E+00	16.989	1077	1.127	1.049	1.211	2.955	0.0011

SOMA_Q9NZK5_CECR1_6077_63	2234	1.295	1.221	1.374	0.00E+00	16.956	NA	NA	NA	NA	NA	NA
SOMA_Q16778_HIST2H2_14143_8	2234	1.275	1.206	1.348	0.00E+00	16.884	NA	NA	NA	NA	NA	NA
SOMA_O00548_DLL1_5349_69	2234	1.285	1.213	1.362	0.00E+00	16.859	1077	1.228	1.157	1.304	10.816	<0.0001
SOMA_P11142_HSPA8_5903_91	2234	1.279	1.209	1.354	0.00E+00	16.803	1077	1.130	1.075	1.188	5.804	<0.0001
SOMA_P38571_LIPA_7219_152	2234	1.277	1.207	1.351	0.00E+00	16.78	NA	NA	NA	NA	NA	NA
SOMA_Q5JXA9_SIRPB2_5669_26	2234	1.278	1.208	1.353	0.00E+00	16.764	1077	1.063	0.983	1.151	0.895	0.1273
SOMA_P98172_EFNB1_13104_32	2234	1.284	1.212	1.36	0.00E+00	16.728	1077	1.126	1.063	1.193	4.278	<0.0001
SOMA_P22692_IGFBP4_2950_57	2234	1.291	1.217	1.369	0.00E+00	16.72	1077	1.239	1.162	1.320	10.306	<0.0001
SOMA_Q99542_MMP19_6425_87	2234	1.297	1.221	1.377	0.00E+00	16.581	1077	1.554	1.439	1.678	28.491	<0.0001
SOMA_P07360_C8G_14708_59	2234	0.775	0.731	0.822	0.00E+00	16.566	1077	0.802	0.723	0.889	4.580	<0.0001
SOMA_Q9BXY4_RSPO3_8427_118	2234	1.291	1.217	1.37	0.00E+00	16.486	1077	1.204	1.133	1.279	8.754	<0.0001
SOMA_P00747_PLG_4151_6	2234	0.777	0.733	0.824	0.00E+00	16.483	1077	0.620	0.560	0.685	19.953	<0.0001
SOMA_P41222_PTGDS_10514_5	2234	1.287	1.214	1.365	0.00E+00	16.475	1077	1.253	1.173	1.339	10.641	<0.0001
SOMA_P03950_ANG_4874_3	2234	1.282	1.21	1.358	0.00E+00	16.438	1077	1.333	1.220	1.457	9.723	<0.0001
SOMA_P05160_F13B_5658_64	2234	0.77	0.725	0.818	0.00E+00	16.431	1077	0.740	0.667	0.821	7.832	<0.0001
SOMA_Q01449_MYL7_19296_51	2234	0.778	0.734	0.825	0.00E+00	16.429	1077	0.564	0.505	0.629	23.853	<0.0001
SOMA_P59901_LILRA4_8299_66	2234	0.779	0.735	0.826	0.00E+00	16.425	1077	0.624	0.517	0.752	6.143	<0.0001
SOMA_Q6UXM1_LRIG3_3322_52	2234	0.78	0.736	0.827	0.00E+00	16.339	1077	0.850	0.767	0.942	2.694	0.002
SOMA_P15692_VEGFA_2597_8	2234	1.285	1.212	1.362	0.00E+00	16.299	1077	1.346	1.257	1.441	16.749	<0.0001

SOMA_P55774_CCL18_3044_3	2234	1.285	1.212	1.363	0.00E+00	16.298	1077	1.334	1.220	1.460	9.494	<0.0001
SOMA_P08237_PFKM_17384_110	2234	0.774	0.728	0.821	0.00E+00	16.295	1077	0.599	0.493	0.729	6.523	<0.0001
SOMA_P07766_CD3E_8069_85	2234	0.784	0.74	0.83	1.65E-13	16.149	1077	0.744	0.669	0.826	7.490	<0.0001
SOMA_Q12794_HYAL1_8309_12	2234	0.783	0.739	0.829	1.65E-13	16.105	1077	0.758	0.678	0.849	5.805	<0.0001
SOMA_Q9UK23_NAGPA_11208_15	2234	0.786	0.742	0.831	1.65E-13	16.083	1077	0.830	0.748	0.922	3.301	0.0005
SOMA_Q765I0_UTS2B_6290_3	2234	1.274	1.203	1.348	1.65E-13	16.038	1077	1.090	1.017	1.167	1.845	0.0143
SOMA_Q9P0K1_ADAM22_7933_75	2234	0.784	0.74	0.83	1.65E-13	16.023	1077	0.997	0.895	1.111	0.018	0.959
SOMA_Q9HBE5_IL21R_9366_54	2234	1.273	1.202	1.347	1.65E-13	16.003	1077	1.148	1.082	1.217	5.400	<0.0001
SOMA_Q8N6M8_IQCF1_7991_54	2234	0.788	0.745	0.834	1.65E-13	15.999	1077	0.697	0.542	0.896	2.305	0.005
SOMA_P21246_PTN_3045_72	2234	1.286	1.212	1.364	1.65E-13	15.998	1077	1.304	1.219	1.395	13.986	<0.0001
SOMA_P04179_SOD2_5008_51	2234	0.777	0.732	0.825	1.65E-13	15.984	1077	0.735	0.634	0.852	4.355	<0.0001
SOMA_Q12906_ILF3_12759_47	2234	1.271	1.201	1.345	1.65E-13	15.954	1077	1.215	1.141	1.295	8.876	<0.0001
SOMA_Q9H6Z4_RANBP3_14037_18	2234	1.288	1.214	1.368	1.65E-13	15.935	1077	1.209	1.121	1.305	6.001	<0.0001
SOMA_Q01523_DEFA5_16785_45	2234	1.28	1.207	1.357	1.65E-13	15.89	1077	1.155	1.076	1.240	4.179	<0.0001
SOMA_O76076_WISP2_6392_7	2234	1.276	1.205	1.352	1.65E-13	15.879	NA	NA	NA	NA	NA	NA
SOMA_P02748_C9_3060_43	2234	1.276	1.204	1.351	1.65E-13	15.852	1077	1.573	1.422	1.740	17.940	<0.0001
SOMA_P42345_FRAP1_16780_6	2234	1.271	1.201	1.346	3.31E-13	15.663	NA	NA	NA	NA	NA	NA
SOMA_P43363_MAGEA10_13610_9	2234	0.794	0.752	0.839	3.31E-13	15.646	1077	0.630	0.531	0.747	6.990	<0.0001
SOMA_P02743_APCS_2474_54	2234	0.779	0.734	0.827	3.31E-13	15.642	1077	0.675	0.609	0.747	13.376	<0.0001

SOMA_P49767_VEGFC_3132_1	2234	1.265	1.196	1.338	3.31E-13	15.64	1077	1.047	0.958	1.144	0.507	0.3112
SOMA_P09669_COX6C_8903_1	2234	0.793	0.751	0.838	3.31E-13	15.631	1077	0.271	0.153	0.480	5.111	<0.0001
SOMA_Q9C005_DPY30_13943_38	2234	1.282	1.208	1.361	3.31E-13	15.585	1077	1.320	1.223	1.424	12.092	<0.0001
SOMA_Q96MM7_HS6ST2_13524_25	2234	1.274	1.202	1.35	4.96E-13	15.538	1077	1.369	1.287	1.456	22.534	<0.0001
SOMA_P49913_CAMP_15481_45	2234	0.776	0.731	0.825	4.96E-13	15.528	1077	0.868	0.779	0.968	1.976	0.0106
SOMA_Q86TD4_SRL_10940_25	2234	1.276	1.203	1.353	4.96E-13	15.528	1077	1.207	1.141	1.276	10.474	<0.0001
SOMA_Q86Y01_DTX1_11430_49	2234	0.786	0.742	0.832	4.96E-13	15.527	1077	0.628	0.559	0.705	14.463	<0.0001
SOMA_Q15063_POSTN_3457_57	2234	1.282	1.208	1.361	4.96E-13	15.478	1077	1.415	1.301	1.539	15.259	<0.0001
SOMA_P43307_SSR1_8106_15	2234	0.775	0.729	0.824	4.96E-13	15.428	1077	0.991	0.883	1.111	0.059	0.8731
SOMA_P67809_YBX1_9751_72	2234	1.27	1.199	1.346	6.61E-13	15.361	1077	1.270	1.182	1.364	10.245	<0.0001
SOMA_P07585_DCN_2666_53	2234	1.262	1.193	1.335	6.61E-13	15.334	1077	1.076	1.034	1.120	3.458	0.0003
SOMA_P23528_CFL1_4203_50	2234	0.785	0.741	0.833	8.27E-13	15.299	1077	0.707	0.623	0.801	7.192	<0.0001
SOMA_Q5JTV8_TOR1AIP_9039_47	2234	0.793	0.75	0.839	8.27E-13	15.286	1077	0.654	0.582	0.735	11.992	<0.0001
SOMA_Q16512_PKN1_12562_1	2234	0.781	0.736	0.829	8.27E-13	15.282	1077	0.843	0.742	0.958	2.049	0.0089
SOMA_Q9BR61_ACBD6_19341_36	2234	1.273	1.201	1.349	8.27E-13	15.275	1077	0.971	0.879	1.072	0.254	0.5567
SOMA_Q9UN70_PCDHGC3_7859_21	2234	0.782	0.737	0.83	8.27E-13	15.262	1077	0.752	0.639	0.885	3.225	0.0006
SOMA_P13497_BMP1_3348_49	2234	0.79	0.746	0.836	8.27E-13	15.251	1077	0.628	0.560	0.704	14.636	<0.0001
SOMA_Q6UX46_FAM150B_6284_7	2234	1.264	1.194	1.338	8.27E-13	15.241	NA	NA	NA	NA	NA	NA
SOMA_Q7Z5A7_FAM19A5_5609_92	2234	1.264	1.195	1.338	8.27E-13	15.241	NA	NA	NA	NA	NA	NA

SOMA_Q13426_XRCC4_9886_28	2234	1.269	1.198	1.345	8.27E-13	15.239	1077	1.044	0.978	1.114	0.698	0.2007
SOMA_P01009_SERPINA_3580_25	2234	1.277	1.204	1.356	9.92E-13	15.162	1077	1.560	1.427	1.705	21.998	<0.0001
SOMA_P10147_CCL3_3040_59	2234	1.267	1.196	1.342	9.92E-13	15.157	1077	1.177	1.114	1.245	8.097	<0.0001
SOMA_Q99435_NELL2_6022_57	2234	1.272	1.2	1.349	9.92E-13	15.157	1077	1.068	1.021	1.116	2.410	0.0039
SOMA_A6NGZ8_SMIM9_8888_33	2234	0.79	0.746	0.837	1.16E-12	15.128	1077	0.893	0.780	1.022	1.000	0.1001
SOMA_Q9BXR6_CFHR5_16055_3	2234	0.785	0.74	0.833	1.16E-12	15.12	1077	0.858	0.774	0.951	2.436	0.0037
SOMA_O14907_TAX1BP3_12498_12	2234	1.262	1.192	1.335	1.16E-12	15.116	1077	0.943	0.854	1.043	0.595	0.2542
SOMA_Q14449_GRB14_13628_58	2234	0.785	0.74	0.833	1.32E-12	15.067	1077	0.795	0.671	0.942	2.086	0.0082
SOMA_P11021_HSPA5_16588_10	2234	1.265	1.194	1.34	1.65E-12	14.937	1077	1.283	1.189	1.384	9.839	<0.0001
SOMA_P28325_CST5_3803_10	2234	1.272	1.199	1.349	1.82E-12	14.931	1077	1.133	1.081	1.188	6.669	<0.0001
SOMA_Q08708_CD300C_5066_134	2234	1.273	1.2	1.351	1.82E-12	14.897	1077	1.215	1.147	1.288	10.398	<0.0001
SOMA_P10451_SPP1_13113_7	2234	1.269	1.197	1.345	1.98E-12	14.87	1077	1.215	1.156	1.277	13.862	<0.0001
SOMD O14793 GDF11 M 27654 PASS	2234	0.78	0.734	0.829	2.15E-12	14.858	NA	NA	NA	NA	NA	NA
SOMD O95390 GDF11 M 27654 PASS	2234	0.78	0.734	0.829	2.15E-12	14.858	NA	NA	NA	NA	NA	NA
SOMA_Q96KN2_CNDP1_5456_59	2234	0.785	0.74	0.833	2.15E-12	14.837	1077	0.637	0.569	0.712	14.667	<0.0001
SOMA_Q13976_PRKG1_13067_5	2234	0.794	0.75	0.84	2.31E-12	14.817	1077	0.249	0.152	0.408	7.458	<0.0001
SOMA_P05026_ATP1B1_13392_13	2234	0.795	0.752	0.842	2.48E-12	14.77	1077	0.626	0.559	0.700	15.439	<0.0001
SOMA_Q16762_TST_12663_1	2234	1.278	1.203	1.358	2.48E-12	14.766	1077	1.306	1.202	1.420	9.513	<0.0001
SOMA_O00300_TNFRSF1_8304_50	2234	1.272	1.199	1.349	2.64E-12	14.764	1077	1.365	1.264	1.474	14.733	<0.0001

SOMA_P20800_EDN2_12574_36	2234	1.259	1.19	1.333	2.81E-12	14.731	1077	1.084	1.021	1.150	2.098	0.008
SOMA_O60260_PARK2_13013_41	2234	0.83	0.793	0.869	2.81E-12	14.727	NA	NA	NA	NA	NA	NA
SOMA_P55773_CCL23_2913_1	2234	1.276	1.202	1.356	2.81E-12	14.727	1077	1.301	1.229	1.378	18.524	<0.0001
SOMA_P08697_SERPINF_3024_18	2234	0.789	0.744	0.837	2.81E-12	14.719	1077	0.765	0.691	0.846	6.718	<0.0001
SOMA_P02008_HBZ_6919_3	2234	0.787	0.742	0.835	2.98E-12	14.699	1077	0.645	0.535	0.779	5.321	<0.0001
SOMA_Q99645_EPYC_9278_9	2234	1.267	1.195	1.343	3.14E-12	14.677	NA	NA	NA	NA	NA	NA
SOMA_P42785_PRCP_5722_78	2234	0.787	0.741	0.835	3.31E-12	14.654	1077	0.663	0.590	0.744	11.398	<0.0001
SOMA_O60911_CTSV_3364_76	2234	0.79	0.746	0.838	3.47E-12	14.632	1077	0.949	0.815	1.104	0.306	0.4945
SOMA_P0C0P6_NPS_6390_18	2234	0.791	0.747	0.839	3.47E-12	14.629	1077	0.564	0.501	0.635	20.376	<0.0001
SOMA_P51965_UBE2E1_14326_4	2234	1.267	1.195	1.344	3.47E-12	14.625	1077	0.978	0.883	1.082	0.178	0.6634
SOMA_Q9NR34_MAN1C1_13427_66	2234	1.267	1.195	1.343	3.64E-12	14.62	1077	1.290	1.225	1.359	21.191	<0.0001
SOMA_Q6ZTQ4_CDHR3_8222_49	2234	0.787	0.742	0.835	4.13E-12	14.557	1077	1.012	0.917	1.117	0.091	0.81
SOMA_O94813_SLIT2_18930_28	2234	1.27	1.196	1.347	4.79E-12	14.494	1077	1.069	1.009	1.132	1.628	0.0236
SOMA_Q8WYQ3_CHCHD10_11270_17	2234	1.259	1.189	1.333	5.12E-12	14.468	1077	1.071	1.003	1.144	1.397	0.0401
SOMA_Q8N2K1_UBE2J2_8802_24	2234	1.269	1.196	1.346	5.29E-12	14.451	NA	NA	NA	NA	NA	NA
SOMA_P01833_PIGR_3216_2	2234	1.269	1.196	1.346	5.95E-12	14.402	1077	1.182	1.114	1.253	7.527	<0.0001
SOMA_Q8NBJ4_GOLM1_8983_7	2234	1.26	1.19	1.335	6.28E-12	14.37	1077	1.042	0.980	1.106	0.729	0.1868
SOMA_O60939_SCN2B_8353_15	2234	0.796	0.751	0.842	6.94E-12	14.331	1077	0.952	0.835	1.086	0.334	0.4631
SOMA_P21583_KITLG_9377_25	2234	0.79	0.745	0.838	6.94E-12	14.329	1077	0.782	0.551	1.112	0.768	0.1708

SOMA_P14649_MYL6B_14227_21	2234	1.266	1.193	1.343	7.11E-12	14.32	1077	1.166	1.104	1.232	7.376	<0.0001
SOMA_Q8N1N2_DYNAP_10692_48	2234	1.26	1.189	1.335	7.27E-12	14.314	NA	NA	NA	NA	NA	NA
SOMA_P19823_ITIH2_9326_33	2234	0.788	0.743	0.837	7.60E-12	14.292	1077	0.676	0.613	0.746	14.223	<0.0001
SOMA_Q9UN71_PCDHGB4_10721_76	2234	1.258	1.187	1.332	8.10E-12	14.26	NA	NA	NA	NA	NA	NA
SOMA_P02741_CRP_4337_49	2234	1.263	1.191	1.34	8.27E-12	14.259	1077	1.563	1.414	1.728	17.656	<0.0001
SOMA_P28066_PSMA5_18925_24	2234	0.801	0.757	0.847	9.42E-12	14.202	1077	0.689	0.620	0.765	11.467	<0.0001
SOMA_P40394_ADH7_11377_19	2234	0.792	0.747	0.84	1.07E-11	14.145	1077	0.873	0.746	1.020	1.057	0.0877
SOMA_P0C7M6_IQCF3_13439_6	2234	0.79	0.745	0.839	1.07E-11	14.138	1077	0.898	0.720	1.121	0.465	0.343
SOMA_Q6WN34_CHRDL2_6086_15	2234	1.263	1.191	1.34	1.09E-11	14.136	1077	1.326	1.235	1.425	13.971	<0.0001
SOMA_Q9Y286_SIGLEC7_2742_68	2234	1.262	1.19	1.339	1.22E-11	14.087	1077	1.086	1.045	1.129	4.542	<0.0001
SOMA_O14763_TNFRSF1_5534_49	2234	1.246	1.178	1.317	1.47E-11	14.006	1077	1.165	1.091	1.245	5.228	<0.0001
SOMA_P78310_CXADR_11204_80	2234	0.792	0.746	0.84	1.49E-11	14	1077	0.470	0.377	0.586	10.642	<0.0001
SOMA_O14793_MSTN_14583_49	2234	0.788	0.742	0.837	1.55E-11	13.98	1077	0.893	0.757	1.053	0.751	0.1776
SOMA_Q9UHG3_PCYOX1_6431_68	2234	0.791	0.745	0.839	1.60E-11	13.969	1077	0.682	0.608	0.765	10.229	<0.0001
SOMA_P09651_HNRNPA1_12466_7	2234	1.247	1.179	1.318	1.72E-11	13.939	1077	0.985	0.885	1.097	0.107	0.7821
SOMA_Q155Q3_DIXDC1_13441_30	2234	0.79	0.744	0.839	1.72E-11	13.938	1077	0.914	0.790	1.059	0.635	0.2316
SOMA_Q9NPY3_CD93_14136_234	2234	1.261	1.189	1.337	1.88E-11	13.9	1077	1.404	1.299	1.516	17.166	<0.0001
SOMA_P35247_SFTPD_19590_46	2234	1.254	1.184	1.328	1.93E-11	13.886	1077	1.292	1.202	1.389	11.338	<0.0001
SOMA_O14594_NCAN_15573_110	2234	0.797	0.752	0.844	2.02E-11	13.869	1077	0.771	0.690	0.861	5.396	<0.0001

SOMA_Q8TBE7_SLC35G2_13501_10	2234	0.792	0.746	0.84	2.25E-11	13.82	1077	0.605	0.497	0.736	6.311	<0.0001
SOMA_O00478_BTN3A3_17692_2	2234	1.257	1.186	1.333	2.33E-11	13.805	1077	1.103	1.036	1.174	2.645	0.0023
SOMA_Q9UMF0_ICAM5_5124_69	2234	1.26	1.188	1.337	2.56E-11	13.764	1077	1.343	1.228	1.469	10.015	<0.0001
SOMA_P08571_CD14_8969_49	2234	1.267	1.193	1.346	2.64E-11	13.75	1077	1.084	1.031	1.140	2.809	0.0016
SOMA_P19957_PI3_4982_54	2234	1.257	1.186	1.333	2.64E-11	13.75	1077	1.274	1.193	1.361	12.357	<0.0001
SOMA_Q63HQ2_EGFLAM_12338_27	2234	1.268	1.194	1.348	2.89E-11	13.711	1077	1.135	1.086	1.187	7.670	<0.0001
SOMA_Q9H2A7_CXCL16_2436_49	2234	1.261	1.188	1.338	2.94E-11	13.705	1077	1.393	1.296	1.498	18.592	<0.0001
SOMA_Q7Z4F1_LRP10_16610_13	2234	1.252	1.182	1.326	3.16E-11	13.674	1077	1.249	1.185	1.316	15.981	<0.0001
SOMA_Q10472_GALNT1_7090_17	2234	1.256	1.185	1.332	3.31E-11	13.653	1077	1.074	1.027	1.123	2.798	0.0016
SOMA_Q8IW41_MAPKAPK_8382_47	2234	0.803	0.758	0.849	3.75E-11	13.599	1077	0.864	0.771	0.967	1.958	0.011
SOMA_P61201_COPS2_14029_42	2234	0.794	0.748	0.843	3.77E-11	13.596	1077	0.651	0.564	0.751	8.438	<0.0001
SOMA_O00339_MATN2_3325_2	2234	1.257	1.185	1.334	3.98E-11	13.573	1077	1.333	1.238	1.435	13.542	<0.0001
SOMA_Q07021_C1QBP_4967_1	2234	0.797	0.752	0.845	4.07E-11	13.565	1077	0.980	0.854	1.125	0.110	0.7759
SOMA_Q7L7L0_HIST3H2_14144_3	2234	1.242	1.175	1.313	4.08E-11	13.563	NA	NA	NA	NA	NA	NA
SOMA_Q15762_CD226_5062_60	2234	1.246	1.177	1.318	4.17E-11	13.554	1077	1.132	1.065	1.202	4.177	<0.0001
SOMA_Q9UKZ9_PCOLCE2_6081_52	2234	0.796	0.75	0.844	4.23E-11	13.546	1077	0.593	0.532	0.661	20.272	<0.0001
SOMA_Q9BUN1_MENT_5744_12	2234	0.798	0.753	0.846	4.46E-11	13.523	1077	0.636	0.544	0.744	7.824	<0.0001
SOMA_P51858_HDGF_8953_47	2234	1.251	1.181	1.326	4.55E-11	13.515	1077	1.087	1.033	1.144	2.868	0.0014
SOMA_P16234_PDGFRA_10366_11	2234	1.25	1.18	1.325	4.69E-11	13.501	1077	1.053	0.987	1.123	0.929	0.1177

SOMA_P13686_ACP5_3232_28	2234	0.793	0.746	0.842	4.76E-11	13.496	1077	0.833	0.738	0.940	2.533	0.0029
SOMA_P10721_KIT_2475_1	2234	0.795	0.749	0.843	4.81E-11	13.491	1077	0.797	0.715	0.888	4.362	<0.0001
SOMA_P51858_HDGF_16758_96	2234	1.253	1.182	1.328	4.84E-11	13.488	1077	1.110	1.053	1.170	4.005	<0.0001
SOMA_Q9UBI4_STOML1_17344_23	2234	0.795	0.749	0.843	4.88E-11	13.485	1077	0.466	0.354	0.615	7.190	<0.0001
SOMA_O43915_FIGF_14705_1	2234	1.247	1.178	1.32	4.99E-11	13.474	NA	NA	NA	NA	NA	NA
SOMA_Q9HCK4_ROBO2_5116_62	2234	1.256	1.184	1.333	5.95E-11	13.399	1077	1.349	1.249	1.457	13.528	<0.0001
SOMA_O60880_SH2D1A_4567_82	2234	1.24	1.173	1.312	6.08E-11	13.389	1077	1.036	0.947	1.134	0.354	0.4429
SOMA_P01298_PPY_4588_1	2234	1.252	1.181	1.328	6.23E-11	13.378	1077	1.203	1.134	1.275	9.212	<0.0001
SOMA_P01033_TIMP1_2211_9	2234	1.267	1.191	1.347	7.16E-11	13.318	1077	1.532	1.432	1.639	34.344	<0.0001
SOMA_P19174_PLCG1_4563_61	2234	0.818	0.776	0.862	7.49E-11	13.299	1077	0.896	0.716	1.119	0.478	0.3324
SOMA_Q12884_FAP_5029_3	2234	0.796	0.751	0.845	7.59E-11	13.293	1077	0.865	0.762	0.982	1.600	0.0251
SOMA_P55291_CDH15_5410_53	2234	0.8	0.754	0.848	7.85E-11	13.278	1077	0.882	0.762	1.021	1.036	0.0921
SOMA_Q07820_MCL1_10396_6	2234	1.252	1.181	1.327	8.18E-11	13.26	1077	1.048	0.972	1.130	0.655	0.2211
SOMA_Q9H2U2_PPA2_18307_71	2234	0.804	0.76	0.851	8.40E-11	13.249	1077	0.961	0.841	1.098	0.250	0.5626
SOMA_O43508_TNFSF12_5939_42	2234	0.793	0.747	0.843	8.79E-11	13.228	1077	1.037	0.951	1.131	0.389	0.4079
SOMA_O95319_CELF2_7245_2	2234	1.236	1.169	1.307	1.06E-10	13.149	1077	1.117	1.041	1.198	2.690	0.002
SOMA_Q8IYN2_TCEAL8_19109_32	2234	0.804	0.76	0.852	1.09E-10	13.136	1077	0.954	0.842	1.081	0.336	0.461
SOMA_Q96E93_KLRG1_10548_35	2234	1.252	1.18	1.328	1.19E-10	13.098	NA	NA	NA	NA	NA	NA
SOMD P0DML2 CSH1 CS 13103 125 PASS	2234	0.801	0.756	0.849	1.25E-10	13.075	NA	NA	NA	NA	NA	NA

SOMD P0DML3 CSH1 CS 13103 125 PASS	2234	0.801	0.756	0.849	1.25E-10	13.075	NA	NA	NA	NA	NA	NA
SOMA_P23381_WARS_9870_17	2234	1.254	1.182	1.331	1.29E-10	13.062	NA	NA	NA	NA	NA	NA
SOMA_O75674_TOM1L1_13652_2	2234	0.795	0.749	0.845	1.43E-10	13.017	1077	0.882	0.748	1.041	0.863	0.1371
SOMA_Q9HCR9_PDE11A_5252_33	2234	0.804	0.76	0.852	1.47E-10	13.006	1077	0.915	0.828	1.013	1.064	0.0862
SOMA_P20783_NTF3_4145_58	2234	1.252	1.18	1.328	1.57E-10	12.977	1077	1.120	1.071	1.172	6.074	<0.0001
SOMA_P00995_SPINK1_8243_55	2234	1.239	1.171	1.311	1.82E-10	12.913	1077	0.963	0.842	1.101	0.237	0.5796
SOMA_P02647_APOA1_2750_3	2234	0.802	0.757	0.851	1.87E-10	12.901	1077	0.630	0.564	0.704	15.477	<0.0001
SOMA_P01019_AGT_3484_60	2234	0.796	0.75	0.846	1.97E-10	12.878	1077	0.859	0.767	0.963	2.042	0.0091
SOMA_Q8N565_MREG_19145_4	2234	0.802	0.756	0.85	2.50E-10	12.776	1077	0.985	0.886	1.097	0.104	0.7876
SOMA_P00451_F8_13499_30	2234	1.24	1.171	1.312	2.57E-10	12.763	1077	1.746	1.597	1.910	33.555	<0.0001
SOMA_Q9H9V4_RNF122_11160_56	2234	0.804	0.759	0.852	2.74E-10	12.736	1077	0.641	0.545	0.754	7.107	<0.0001
SOMA_P01270_PTH_5954_62	2234	1.239	1.17	1.312	2.77E-10	12.731	1077	1.062	1.017	1.109	2.163	0.0069
SOMA_Q9BXJ4_C1QTNF3_10749_18	2234	1.241	1.172	1.315	2.94E-10	12.705	NA	NA	NA	NA	NA	NA
SOMA_Q9H2B2_SYT4_17355_56	2234	0.799	0.753	0.848	3.05E-10	12.689	1077	0.775	0.669	0.899	3.134	0.0007
SOMA_P29353_SHC1_5272_55	2234	1.236	1.168	1.308	3.22E-10	12.665	1077	0.904	0.817	1.001	1.278	0.0528
SOMA_P48775_TDO2_9880_33	2234	0.805	0.759	0.853	3.82E-10	12.591	1077	0.671	0.579	0.779	6.818	<0.0001
SOMA_P02786_TFRC_6895_1	2234	1.251	1.178	1.328	3.87E-10	12.585	1077	1.130	1.089	1.172	10.184	<0.0001
SOMA_Q8IVM0_CCDC50_12399_194	2234	1.241	1.171	1.314	3.91E-10	12.581	1077	0.947	0.831	1.079	0.383	0.4143
SOMA_P00734_F2_4157_2	2234	0.803	0.757	0.852	4.06E-10	12.565	1077	0.309	0.221	0.431	11.272	<0.0001

SOMA_P00797_REN_3396_54	2234	1.247	1.175	1.323	4.58E-10	12.512	1077	1.284	1.174	1.405	7.294	<0.0001
SOMA_Q13277_STX3_7186_111	2234	1.234	1.166	1.306	4.64E-10	12.506	1077	1.062	0.991	1.137	1.056	0.0878
SOMA_Q2Y0W8_SLC4A8_12798_46	2234	0.802	0.756	0.851	4.81E-10	12.491	1077	1.014	0.915	1.124	0.106	0.7841
SOMA_Q8N0W4_NLGN4X_5357_60	2234	1.231	1.164	1.302	5.16E-10	12.46	1077	1.077	0.999	1.161	1.274	0.0532
SOMA_Q8N8U9_BMPER_15368_3	2234	1.238	1.169	1.312	5.17E-10	12.459	1077	1.234	1.170	1.302	13.903	<0.0001
SOMA_P35318_ADM_7922_5	2234	1.243	1.172	1.317	5.19E-10	12.458	1077	0.978	0.877	1.091	0.161	0.6899
SOMA_Q9UBX7_KLK11_2831_29	2234	1.237	1.168	1.31	5.25E-10	12.453	1077	1.243	1.178	1.311	14.601	<0.0001
SOMA_P68431_HIST1H3_14146_92	2234	1.231	1.164	1.302	5.49E-10	12.433	NA	NA	NA	NA	NA	NA
SOMA_P19256_CD58_10938_13	2234	1.234	1.166	1.306	5.69E-10	12.418	1077	1.150	1.087	1.217	5.922	<0.0001
SOMA_Q9UII4_HERC5_12934_1	2234	0.805	0.759	0.853	5.77E-10	12.412	1077	0.628	0.520	0.757	5.930	<0.0001
SOMA_Q9NX14_NDUFB11_7747_47	2234	0.81	0.765	0.857	5.92E-10	12.401	1077	0.807	0.660	0.986	1.448	0.0357
SOMA_Q9BXY4_RSPO3_13094_75	2234	1.241	1.17	1.315	6.37E-10	12.369	1077	1.126	1.071	1.183	5.535	<0.0001
SOMA_P09603_CSF1_3738_54	2234	1.23	1.163	1.301	6.56E-10	12.356	1077	1.053	1.000	1.109	1.320	0.0479
SOMA_Q9UBX1_CTSF_9212_22	2234	0.807	0.762	0.855	7.16E-10	12.318	1077	0.703	0.621	0.796	7.592	<0.0001
SOMA_P52758_HRSP12_14636_25	2234	1.239	1.169	1.313	7.31E-10	12.309	NA	NA	NA	NA	NA	NA
SOMA_P40189_IL6ST_2620_4	2234	1.252	1.178	1.33	7.49E-10	12.299	1077	1.604	1.475	1.744	27.704	<0.0001
SOMA_O95185_UNC5C_5139_32	2234	1.247	1.174	1.323	7.58E-10	12.293	1077	1.312	1.207	1.425	9.790	<0.0001
SOMA_Q16832_DDR2_15381_45	2234	1.239	1.169	1.314	7.91E-10	12.275	1077	1.022	0.955	1.093	0.279	0.5262
SOMA_P22626_HNRNPA2_5351_52	2234	1.226	1.16	1.296	8.24E-10	12.257	1077	1.010	0.915	1.114	0.074	0.844

SOMA_O75475_PSIP1_17176_13	2234	1.24	1.17	1.315	8.37E-10	12.25	1077	1.149	1.079	1.223	4.823	<0.0001
SOMA_P02458_COL2A1_18875_125	2234	0.806	0.76	0.854	8.42E-10	12.248	1077	0.602	0.503	0.720	7.495	<0.0001
SOMA_O14558_HSPB6_19127_1	2234	1.241	1.17	1.316	8.73E-10	12.232	1077	1.300	1.219	1.387	14.767	<0.0001
SOMA_P43489_TNFRSF4_3730_81	2234	1.228	1.161	1.298	8.84E-10	12.226	1077	1.013	0.938	1.094	0.132	0.7378
SOMA_Q9NPA1_KCNMB3_8905_20	2234	0.807	0.761	0.855	8.98E-10	12.22	1077	0.708	0.614	0.816	5.749	<0.0001
SOMA_Q5GFL6_VWA2_7128_9	2234	0.811	0.766	0.859	1.02E-09	12.163	1077	0.727	0.283	1.864	0.295	0.5069
SOMA_Q86VZ4_LRP11_15472_16	2234	1.243	1.171	1.319	1.02E-09	12.163	1077	1.294	1.214	1.378	14.760	<0.0001
SOMA_Q86SI9_C5orf38_6378_2	2234	0.805	0.759	0.854	1.06E-09	12.148	1077	0.636	0.563	0.719	12.370	<0.0001
SOMA_P37023_ACVRL1_16318_12	2234	1.237	1.167	1.311	1.07E-09	12.144	1077	1.099	1.040	1.161	3.062	0.0009
SOMA_P02679_FGG_4989_7	2234	1.235	1.165	1.308	1.14E-09	12.117	1077	1.356	1.258	1.462	14.724	<0.0001
SOMD P05556 ITGA1 I 3503 4 FLAG	2234	1.234	1.165	1.307	1.15E-09	12.113	NA	NA	NA	NA	NA	NA
SOMD P56199 ITGA1 I 3503 4 FLAG	2234	1.234	1.165	1.307	1.15E-09	12.113	NA	NA	NA	NA	NA	NA
SOMA_P61077_UBE2D3_19280_29	2234	0.803	0.757	0.853	1.16E-09	12.107	1077	0.177	0.073	0.426	3.948	0.0001
SOMA_Q96PZ7_CSMD1_9598_23	2234	0.805	0.758	0.854	1.19E-09	12.099	1077	0.568	0.483	0.668	11.160	<0.0001
SOMA_Q13332_PTPRS_6049_64	2234	0.808	0.762	0.857	1.20E-09	12.094	1077	0.890	0.802	0.989	1.513	0.0307
SOMA_Q9H4I3_TRABD_11262_39	2234	0.804	0.757	0.853	1.20E-09	12.094	1077	0.755	0.606	0.940	1.916	0.0121
SOMA_Q9NNZ3_DNAJC4_8653_132	2234	0.806	0.76	0.855	1.23E-09	12.084	1077	0.803	0.700	0.921	2.762	0.0017
SOMA_Q9UIS9_MBD1_14294_61	2234	0.806	0.759	0.855	1.24E-09	12.078	1077	0.615	0.543	0.696	13.750	<0.0001
SOMA_Q92974_ARHGEF2_12848_9	2234	0.808	0.762	0.856	1.27E-09	12.07	1077	0.853	0.675	1.078	0.738	0.1826

SOMA_P78333_GPC5_4991_12	2234	0.81	0.764	0.858	1.46E-09	12.008	1077	0.257	0.171	0.388	10.082	<0.0001
SOMA_O43927_CXCL13_3487_32	2234	1.235	1.166	1.309	1.47E-09	12.005	1077	1.232	1.165	1.304	12.499	<0.0001
SOMA_P0CG48_UBC_6647_55	2234	1.255	1.179	1.336	1.48E-09	12.004	1077	1.195	1.104	1.293	4.991	<0.0001
SOMA_Q9H999_PANK3_12658_72	2234	0.806	0.76	0.855	1.64E-09	11.959	1077	0.759	0.646	0.893	3.069	0.0009
SOMA_Q13822_ENPP2_16892_23	2234	1.244	1.171	1.321	1.65E-09	11.954	1077	1.247	1.163	1.338	9.181	<0.0001
SOMA_Q15526_SURF1_8009_121	2234	0.81	0.765	0.859	1.71E-09	11.939	1077	0.809	0.676	0.967	1.708	0.0196
SOMA_P48052_CPA2_9276_7	2234	0.854	0.818	0.892	1.74E-09	11.933	1077	0.883	0.686	1.136	0.478	0.3326
SOMA_Q9Y3E2_BOLA1_15370_5	2234	1.234	1.164	1.308	1.74E-09	11.931	1077	1.045	0.957	1.140	0.482	0.3299
SOMA_Q9UJZ1_STOML2_6555_58	2234	0.81	0.765	0.859	1.85E-09	11.906	1077	0.959	0.813	1.132	0.206	0.6217
SOMA_Q8WY21_SORCS1_15636_49	2234	1.237	1.166	1.312	1.90E-09	11.895	1077	1.111	1.062	1.162	5.357	<0.0001
SOMA_P05060_CHGB_8235_48	2234	1.24	1.168	1.315	1.99E-09	11.873	1077	1.185	1.116	1.257	7.601	<0.0001
SOMA_Q6UXG2_KIAA132_10637_50	2234	0.823	0.78	0.869	2.07E-09	11.858	NA	NA	NA	NA	NA	NA
SOMA_Q68G75_LEMD1_8040_9	2234	1.231	1.162	1.303	2.13E-09	11.845	1077	1.056	0.974	1.147	0.727	0.1877
SOMA_P55055_NR1H2_9016_12	2234	0.811	0.765	0.859	2.31E-09	11.808	1077	0.675	0.549	0.829	3.760	0.0002
SOMA_Q8N2Q7_NLGN1_15620_4	2234	1.239	1.168	1.315	2.37E-09	11.798	1077	1.180	1.125	1.239	10.752	<0.0001
SOMA_O75771_RAD51L3_12554_10	2234	0.813	0.768	0.861	2.43E-09	11.788	NA	NA	NA	NA	NA	NA
SOMA_Q9Y2G5_POFUT2_6042_52	2234	1.225	1.158	1.296	2.78E-09	11.728	NA	NA	NA	NA	NA	NA
SOMA_P22792_CPN2_6415_90	2234	0.805	0.757	0.855	2.81E-09	11.724	1077	0.674	0.600	0.758	10.404	<0.0001
SOMA_P29120_PCSK1_13388_57	2234	1.233	1.163	1.308	3.06E-09	11.688	1077	1.167	1.105	1.233	7.478	<0.0001

SOMA_Q8IXJ6_SIRT2_5030_52	2234	0.806	0.759	0.856	3.07E-09	11.686	1077	0.460	0.333	0.636	5.586	<0.0001
SOMA_Q6EMK4_VASN_5682_13	2234	1.244	1.171	1.323	3.11E-09	11.68	1077	1.408	1.302	1.521	17.229	<0.0001
SOMA_Q96JJ7_TMX3_5654_70	2234	1.233	1.163	1.307	3.24E-09	11.663	1077	1.233	1.135	1.340	6.126	<0.0001
SOMA_Q9BYZ8_REG4_11102_22	2234	1.234	1.164	1.308	3.25E-09	11.661	1077	1.152	1.055	1.259	2.782	0.0017
SOMA_P23560_BDNF_14047_78	2234	0.814	0.769	0.862	3.35E-09	11.648	1077	0.625	0.544	0.718	10.567	<0.0001
SOMA_Q14498_RBM39_4284_18	2234	1.219	1.153	1.289	3.58E-09	11.619	1077	1.066	0.992	1.146	1.095	0.0803
SOMA_P55957_BID_5798_3	2234	1.233	1.163	1.307	3.72E-09	11.602	1077	1.066	1.022	1.112	2.487	0.0033
SOMA_Q13137_CALCOCO_12534_10	2234	1.239	1.167	1.316	3.84E-09	11.588	1077	1.169	1.100	1.241	6.425	<0.0001
SOMA_P17096_HMGA1_16536_3	2234	1.222	1.155	1.293	3.92E-09	11.579	1077	1.073	1.020	1.129	2.168	0.0068
SOMA_P07711_CTSL_9275_2	2234	1.237	1.165	1.313	4.07E-09	11.564	NA	NA	NA	NA	NA	NA
SOMA_P13987_CD59_11514_196	2234	1.231	1.161	1.305	4.12E-09	11.558	1077	1.293	1.212	1.380	14.100	<0.0001
SOMA_Q13478_IL18R1_14079_14	2234	1.233	1.163	1.308	4.44E-09	11.525	1077	1.087	1.035	1.142	3.082	0.0008
SOMA_O14737_PDCD5_12517_52	2234	1.236	1.165	1.312	4.51E-09	11.518	1077	1.143	1.038	1.258	2.195	0.0064
SOMD P02671 FGA FGB 490756 PASS	2234	1.23	1.16	1.304	4.52E-09	11.518	NA	NA	NA	NA	NA	NA
SOMD P02675 FGA FGB 490756 PASS	2234	1.23	1.16	1.304	4.52E-09	11.518	NA	NA	NA	NA	NA	NA
SOMD P02679 FGA FGB 490756 PASS	2234	1.23	1.16	1.304	4.52E-09	11.518	NA	NA	NA	NA	NA	NA
SOMA_Q96DX5_ASB9_19601_15	2234	1.226	1.158	1.298	4.68E-09	11.503	1077	1.314	1.226	1.409	13.816	<0.0001
SOMA_P07195_LDHB_3890_8	2234	1.235	1.163	1.31	4.82E-09	11.489	1077	1.095	1.000	1.198	1.310	0.049
SOMA_Q9HCE7_SMURF1_11557_3	2234	1.221	1.155	1.292	5.00E-09	11.474	1077	1.022	0.952	1.098	0.261	0.5479

SOMA_P03971_AMH_4923_79	2234	0.811	0.764	0.86	5.17E-09	11.46	1077	0.718	0.642	0.803	8.165	<0.0001
SOMA_Q8IZJ1_UNC5B_7776_20	2234	1.23	1.161	1.305	5.48E-09	11.434	1077	1.275	1.184	1.372	9.945	<0.0001
SOMA_P20774_OGN_17224_12	2234	1.225	1.157	1.297	5.60E-09	11.424	1077	1.114	1.050	1.182	3.472	0.0003
SOMA_P01138_NGF_5801_72	2234	1.222	1.155	1.293	5.64E-09	11.422	1077	1.062	1.008	1.118	1.631	0.0234
SOMA_Q15582_TGFBI_3283_21	2234	1.244	1.17	1.324	5.64E-09	11.422	1077	1.256	1.144	1.379	5.799	<0.0001
SOMA_P98179_RBM3_12747_89	2234	1.228	1.159	1.301	5.71E-09	11.416	1077	0.952	0.863	1.051	0.482	0.3297
SOMA_P19404_NDUFV2_7748_11	2234	1.236	1.164	1.312	5.77E-09	11.411	1077	1.163	1.064	1.270	3.089	0.0008
SOMA_Q16661_GUCA2B_6223_5	2234	1.231	1.161	1.305	5.86E-09	11.405	1077	1.058	0.989	1.131	0.985	0.1035
SOMA_P50897_PPT1_9244_27	2234	1.237	1.165	1.314	5.90E-09	11.402	1077	1.106	1.021	1.199	1.874	0.0134
SOMA_P09211_GSTP1_4911_49	2234	1.235	1.163	1.31	6.02E-09	11.393	1077	1.043	0.946	1.150	0.401	0.3969
SOMA_Q96DR5_BPIFA2_16302_11	2234	0.809	0.763	0.859	6.14E-09	11.385	1077	0.698	0.611	0.797	6.987	<0.0001
SOMA_P55040_GEM_12817_1	2234	0.814	0.768	0.863	6.67E-09	11.349	1077	0.685	0.546	0.860	2.963	0.0011
SOMA_P51580_TPMT_11218_84	2234	0.814	0.767	0.863	6.74E-09	11.345	1077	0.661	0.577	0.757	8.690	<0.0001
SOMA_O00253_AGRP_2813_11	2234	1.236	1.164	1.312	7.10E-09	11.321	1077	1.058	1.010	1.108	1.758	0.0174
SOMA_Q9UHG2_PCSK1N_9391_60	2234	1.228	1.159	1.302	7.45E-09	11.301	1077	1.016	0.943	1.095	0.170	0.6755
SOMA_Q8IYJ3_SYTL1_12892_10	2234	0.82	0.775	0.867	7.50E-09	11.298	1077	0.994	0.897	1.102	0.040	0.9128
SOMA_P15814_IGLL1_6485_59	2234	0.813	0.766	0.862	8.06E-09	11.267	1077	0.755	0.661	0.863	4.432	<0.0001
SOMA_P35030_PRSS3_18864_7	2234	1.235	1.163	1.311	8.11E-09	11.264	1077	1.124	1.048	1.205	2.950	0.0011
SOMA_Q8IYJ0_PIANP_9599_6	2234	1.232	1.161	1.307	8.14E-09	11.262	1077	1.076	1.029	1.125	2.914	0.0012

SOMA_P06396_GSN_16607_78	2234	0.811	0.764	0.861	8.57E-09	11.24	1077	0.756	0.685	0.834	7.620	<0.0001
SOMA_Q8WXH2_JPH3_9089_77	2234	0.811	0.764	0.861	8.56E-09	11.24	1077	1.053	0.968	1.146	0.636	0.2311
SOMA_Q8TDF5_NETO1_15298_199	2234	1.219	1.152	1.289	8.66E-09	11.236	1077	1.058	0.997	1.123	1.192	0.0643
SOMA_P21781_FGF7_4487_1	2234	1.23	1.16	1.305	8.88E-09	11.224	1077	1.011	0.915	1.117	0.083	0.8256
SOMA_Q14012_CAMK1_3592_4	2234	0.818	0.772	0.866	8.94E-09	11.221	1077	0.619	0.539	0.711	10.862	<0.0001
SOMA_O75534_CSDE1_12735_39	2234	0.815	0.769	0.864	9.20E-09	11.209	1077	0.974	0.857	1.107	0.161	0.6907
SOMA_O43155_FLRT2_13122_19	2234	1.233	1.162	1.309	9.27E-09	11.206	1077	1.335	1.241	1.436	14.065	<0.0001
SOMA_P36222_CHI3L1_11104_13	2234	1.225	1.156	1.298	1.10E-08	11.132	1077	1.217	1.134	1.306	7.236	<0.0001
SOMA_P47895_ALDH1A3_9835_16	2234	0.816	0.77	0.865	1.12E-08	11.124	1077	0.675	0.593	0.767	8.691	<0.0001
SOMA_Q86U06_RBM23_11590_5	2234	1.213	1.148	1.282	1.25E-08	11.077	1077	1.058	0.979	1.143	0.815	0.1531
SOMA_000000_LOC6524_6561_77	2234	0.813	0.766	0.863	1.31E-08	11.055	NA	NA	NA	NA	NA	NA
SOMA_P20807_CAPN3_12385_4	2234	0.814	0.767	0.863	1.31E-08	11.055	1077	0.710	0.510	0.989	1.370	0.0426
SOMA_O95407_TNFRSF6_5070_76	2234	1.219	1.151	1.29	1.32E-08	11.053	1077	1.072	1.009	1.139	1.632	0.0233
SOMA_Q13261_IL15RA_14054_17	2234	1.231	1.159	1.306	1.34E-08	11.046	1077	1.228	1.157	1.302	10.997	<0.0001
SOMA_O15075_DCLK1_17156_72	2234	1.226	1.156	1.3	1.37E-08	11.037	1077	1.158	1.097	1.222	7.091	<0.0001
SOMA_Q96J42_TXNDC15_6366_38	2234	1.227	1.157	1.302	1.38E-08	11.032	1077	1.229	1.154	1.308	9.932	<0.0001
SOMA_Q9GZN4_PRSS22_4534_10	2234	1.224	1.155	1.297	1.53E-08	10.987	1077	1.349	1.255	1.451	15.131	<0.0001
SOMA_O43278_SPINT1_2828_82	2234	0.82	0.774	0.868	1.64E-08	10.959	1077	0.883	0.781	0.999	1.319	0.048
SOMA_O75054_IGSF3_9715_15	2234	1.227	1.156	1.302	1.86E-08	10.903	1077	1.185	1.117	1.257	7.749	<0.0001

SOMA_P12644_BMP4_15667_39	2234	1.235	1.162	1.313	1.94E-08	10.885	1077	1.506	1.396	1.625	25.245	<0.0001
SOMA_Q9UM47_NOTCH3_5108_72	2234	1.224	1.154	1.297	1.97E-08	10.879	1077	1.480	1.352	1.619	16.831	<0.0001
SOMA_Q16352_INA_11436_6	2234	1.213	1.147	1.283	2.08E-08	10.855	1077	1.082	1.027	1.141	2.472	0.0034
SOMA_P14621_ACYP2_12812_25	2234	1.226	1.156	1.301	2.13E-08	10.845	1077	1.041	0.941	1.151	0.359	0.438
SOMA_Q10469_MGAT2_6909_40	2234	0.815	0.769	0.865	2.13E-08	10.844	1077	0.760	0.661	0.874	3.914	0.0001
SOMA_Q9UBC7_GALP_9398_30	2234	0.865	0.829	0.902	2.43E-08	10.788	1077	0.820	0.599	1.122	0.668	0.215
SOMA_Q14393_GAS6_15391_114	2234	1.23	1.158	1.306	2.48E-08	10.779	1077	1.438	1.343	1.539	24.952	<0.0001
SOMA_O75398_DEAF1_6369_82	2234	0.819	0.772	0.868	2.57E-08	10.763	1077	0.741	0.598	0.919	2.201	0.0063
SOMA_Q9UGN4_CD300A_5630_48	2234	1.227	1.156	1.303	2.64E-08	10.751	1077	1.295	1.206	1.391	11.876	<0.0001
SOMA_P07148_FABP1_11516_7	2234	1.226	1.155	1.301	2.93E-08	10.706	1077	1.209	1.139	1.283	9.382	<0.0001
SOMA_P14778_IL1R1_2991_9	2234	1.238	1.163	1.317	2.97E-08	10.7	1077	1.589	1.466	1.723	28.562	<0.0001
SOMA_Q9UK55_SERPINA_13119_26	2234	0.818	0.771	0.867	3.19E-08	10.669	1077	0.817	0.734	0.908	3.725	0.0002
SOMA_P40238_MPL_3473_78	2234	0.873	0.839	0.908	3.23E-08	10.664	1077	0.584	0.506	0.674	12.633	<0.0001
SOMA_Q96JX3_SERAC1_8985_13	2234	0.819	0.772	0.868	3.46E-08	10.634	1077	0.817	0.666	1.002	1.279	0.0527
SOMA_Q13158_FADD_16593_3	2234	0.831	0.787	0.878	3.51E-08	10.627	1077	0.497	0.417	0.593	14.152	<0.0001
SOMA_Q86Z14_KLB_19557_3	2234	0.817	0.77	0.867	3.59E-08	10.618	1077	0.705	0.609	0.815	5.593	<0.0001
SOMA_Q96H15_TIMD4_15449_33	2234	1.224	1.153	1.299	3.65E-08	10.61	1077	1.211	1.153	1.273	13.385	<0.0001
SOMA_Q9NTG7_SIRT3_17495_141	2234	0.819	0.773	0.869	3.93E-08	10.579	1077	0.437	0.314	0.608	6.019	<0.0001
SOMA_Q9BYB0_SHANK3_13242_134	2234	1.214	1.147	1.286	4.06E-08	10.565	1077	0.898	0.808	0.998	1.332	0.0465

SOMA_Q93045_STMN2_10900_272	2234	1.219	1.15	1.293	4.15E-08	10.555	1077	1.000	0.908	1.100	0.002	0.9962
SOMA_P30101_PDIA3_4719_58	2234	0.814	0.766	0.865	4.20E-08	10.549	1077	0.829	0.741	0.928	2.954	0.0011
SOMA_P01584_IL1B_3037_62	2234	1.215	1.147	1.287	4.22E-08	10.548	1077	1.086	1.016	1.160	1.828	0.0149
SOMA_Q15399_TLR1_11149_3	2234	0.815	0.767	0.866	4.32E-08	10.537	1077	0.896	0.668	1.202	0.333	0.4646
SOMA_O15031_PLXNB2_9216_100	2234	1.224	1.153	1.3	4.33E-08	10.536	1077	1.263	1.172	1.360	9.093	<0.0001
SOMA_P01189_POMC_9204_33	2234	1.219	1.15	1.293	4.57E-08	10.513	1077	1.064	1.007	1.125	1.556	0.0278
SOMA_P08133_ANXA6_5335_73	2234	0.823	0.777	0.872	4.69E-08	10.501	1077	0.650	0.582	0.726	13.681	<0.0001
SOMA_P18428_LBP_3074_6	2234	1.223	1.153	1.298	4.96E-08	10.477	1077	1.471	1.354	1.598	19.278	<0.0001
SOMA_Q96CA5_BIRC7_15504_39	2234	1.216	1.147	1.288	5.23E-08	10.454	1077	1.149	1.083	1.219	5.384	<0.0001
SOMA_Q15561_TEAD4_12516_13	2234	1.212	1.145	1.283	5.51E-08	10.432	1077	0.984	0.876	1.105	0.103	0.7891
SOMA_Q8WZ42_TTN_11352_42	2234	1.213	1.145	1.284	5.55E-08	10.429	1077	1.155	1.090	1.223	6.007	<0.0001
SOMA_Q9NQ79_CRTAC1_5632_6	2234	0.821	0.774	0.87	5.65E-08	10.421	1077	0.641	0.568	0.722	12.410	<0.0001
SOMA_P16519_PCSK2_6117_4	2234	1.218	1.149	1.291	5.70E-08	10.417	1077	1.058	0.996	1.123	1.185	0.0653
SOMA_P84095_RHOG_12540_25	2234	0.817	0.77	0.868	6.12E-08	10.386	1077	0.723	0.626	0.835	5.030	<0.0001
SOMA_P63302_SEPW1_18310_26	2234	1.219	1.149	1.293	6.24E-08	10.378	NA	NA	NA	NA	NA	NA
SOMA_Q6UW15_REG3G_15476_6	2234	1.217	1.148	1.291	6.40E-08	10.367	1077	1.133	1.072	1.197	5.031	<0.0001
SOMA_P00740_F9_5307_12	2234	0.824	0.778	0.873	6.50E-08	10.36	1077	0.834	0.755	0.921	3.449	0.0004
SOMA_Q9BXJ5_C1QTNF2_8820_2	2234	0.821	0.774	0.871	7.42E-08	10.302	NA	NA	NA	NA	NA	NA
SOMA_O15389_SIGLEC5_16792_4	2234	1.216	1.147	1.288	7.49E-08	10.298	1077	1.381	1.266	1.506	12.494	<0.0001

SOMA_Q9HAV7_GRPEL1_7113_1	2234	1.216	1.147	1.289	7.55E-08	10.295	1077	1.164	1.068	1.268	3.290	0.0005
SOMA_P05231_IL6_4673_13	2234	1.212	1.145	1.284	7.68E-08	10.288	1077	1.093	1.037	1.152	3.055	0.0009
SOMA_P00746_CFD_2946_52	2234	1.219	1.149	1.294	7.95E-08	10.272	1077	1.404	1.285	1.534	13.236	<0.0001
SOMA_P16112_ACAN_3280_49	2234	0.843	0.801	0.887	7.95E-08	10.272	1077	0.765	0.650	0.900	2.898	0.0013
SOMA_P17813_ENG_4908_6	2234	1.224	1.152	1.3	8.62E-08	10.237	1077	1.672	1.534	1.823	30.947	<0.0001
SOMA_O15467_CCL16_4913_78	2234	1.208	1.142	1.279	8.89E-08	10.224	1077	1.317	1.221	1.421	12.036	<0.0001
SOMA_Q13838_DDX39B_9742_59	2234	1.208	1.141	1.278	9.11E-08	10.214	1077	1.036	0.942	1.139	0.329	0.4686
SOMA_O14791_APOL1_9506_10	2234	0.818	0.77	0.869	9.11E-08	10.213	1077	0.851	0.688	1.054	0.857	0.1389
SOMA_Q9BXN2_CLEC7A_3603_60	2234	1.204	1.139	1.273	9.23E-08	10.208	1077	1.109	1.024	1.200	1.960	0.011
SOMA_Q96GP6_SCARF2_9925_56	2234	1.203	1.138	1.272	9.42E-08	10.199	1077	1.015	0.922	1.117	0.120	0.7589
SOMA_Q99941_ATF6B_11387_3	2234	0.823	0.776	0.872	9.73E-08	10.185	1077	0.652	0.565	0.753	8.262	<0.0001
SOMA_Q96J94_PIWIL1_12793_4	2234	0.818	0.77	0.869	9.94E-08	10.175	1077	1.078	1.007	1.153	1.522	0.0301
SOMA_Q16881_TXNRD1_13967_14	2234	1.215	1.146	1.289	1.09E-07	10.135	1077	0.921	0.826	1.027	0.855	0.1396
SOMA_P04843_RPN1_6458_6	2234	1.206	1.14	1.276	1.10E-07	10.131	1077	1.350	1.242	1.467	11.762	<0.0001
SOMA_O94768_STK17B_5249_31	2234	0.819	0.772	0.87	1.11E-07	10.128	1077	0.942	0.835	1.063	0.475	0.3347
SOMA_Q9UQE7_SMC3_14324_52	2234	1.206	1.14	1.276	1.14E-07	10.118	1077	1.011	0.912	1.121	0.078	0.8348
SOMA_O94907_DKK1_3535_84	2234	0.823	0.776	0.873	1.17E-07	10.105	1077	0.791	0.703	0.890	4.025	<0.0001
SOMA_P21854_CD72_7009_8	2234	1.211	1.143	1.282	1.17E-07	10.105	1077	1.081	1.034	1.130	3.241	0.0006
SOMA_P21860_ERBB3_2617_56	2234	0.826	0.779	0.875	1.23E-07	10.084	1077	0.837	0.740	0.947	2.327	0.0047

SOMA_O75121_MFAP3L_8837_8	2234	0.821	0.774	0.872	1.25E-07	10.077	1077	0.860	0.701	1.055	0.831	0.1475
SOMA_Q9UBU2_DKK2_15678_71	2234	0.824	0.777	0.874	1.30E-07	10.059	1077	0.745	0.667	0.832	6.729	<0.0001
SOMA_Q8WXC3_PYDC1_12835_101	2234	0.826	0.779	0.875	1.33E-07	10.05	1077	1.000	0.903	1.107	0.002	0.9945
SOMA_Q9Y274_ST3GAL6_6947_4	2234	0.829	0.783	0.877	1.36E-07	10.039	1077	0.698	0.635	0.767	13.170	<0.0001
SOMA_P12268_IMPDH2_5250_53	2234	0.824	0.777	0.874	1.47E-07	10.007	1077	0.827	0.724	0.945	2.278	0.0053
SOMA_Q14624_ITIH4_4811_33	2234	1.215	1.145	1.289	1.50E-07	9.996	1077	1.602	1.460	1.756	22.846	<0.0001
SOMA_Q07960_ARHGAP1_11955_1	2234	1.211	1.143	1.284	1.53E-07	9.987	1077	0.977	0.858	1.112	0.142	0.7203
SOMA_Q9Y3E7_VPS24_12508_9	2234	1.211	1.143	1.283	1.54E-07	9.985	NA	NA	NA	NA	NA	NA
SOMA_P02786_TFRC_8795_48	2234	1.219	1.148	1.294	1.59E-07	9.971	1077	1.335	1.270	1.404	28.600	<0.0001
SOMA_O00182_LGALS9_9197_4	2234	1.209	1.141	1.281	1.67E-07	9.951	1077	1.089	1.036	1.144	3.099	0.0008
SOMA_Q05513_PRKCZ_2645_54	2234	1.202	1.137	1.272	1.73E-07	9.935	1077	1.116	1.048	1.188	3.211	0.0006
SOMA_Q6ZMJ2_SCARA5_10419_1	2234	1.216	1.146	1.291	1.81E-07	9.914	1077	1.472	1.367	1.585	23.931	<0.0001
SOMA_Q8NAU1_FNDC5_8041_5	2234	0.819	0.771	0.87	1.83E-07	9.91	NA	NA	NA	NA	NA	NA
SOMA_P00740_F9_4876_32	2234	0.828	0.782	0.877	2.07E-07	9.857	1077	0.834	0.755	0.922	3.444	0.0004
SOMA_P35613_BSG_3585_54	2234	1.204	1.138	1.275	2.07E-07	9.857	1077	1.253	1.175	1.337	11.070	<0.0001
SOMA_P32004_L1CAM_4246_40	2234	0.825	0.778	0.875	2.26E-07	9.818	1077	0.811	0.729	0.902	3.927	0.0001
SOMA_Q96LW7_C9orf89_7778_104	2234	1.211	1.142	1.284	2.26E-07	9.818	NA	NA	NA	NA	NA	NA
SOMA_P60568_IL2_3070_1	2234	0.826	0.779	0.876	2.27E-07	9.817	1077	0.992	0.895	1.101	0.054	0.8838
SOMA_Q6UXN2_TREML4_11139_4	2234	1.212	1.143	1.286	2.40E-07	9.794	NA	NA	NA	NA	NA	NA

SOMA_Q01459_CTBS_6115_40	2234	1.216	1.145	1.291	2.45E-07	9.783	NA	NA	NA	NA	NA	NA
SOMA_Q99759_MAP3K3_12990_39	2234	1.201	1.135	1.27	2.54E-07	9.767	1077	1.052	0.993	1.113	1.072	0.0847
SOMA_Q8IYJ0_PIANP_14114_18	2234	1.21	1.141	1.283	2.58E-07	9.761	1077	1.110	1.062	1.161	5.338	<0.0001
SOMA_P11498_PC_13990_1	2234	1.208	1.14	1.28	2.63E-07	9.753	1077	1.145	1.075	1.220	4.620	<0.0001
SOMA_Q99439_CNN2_18877_15	2234	1.212	1.142	1.286	2.65E-07	9.75	1077	0.996	0.901	1.102	0.025	0.9447
SOMA_P00734_F2_5316_54	2234	0.826	0.779	0.876	2.71E-07	9.74	1077	0.907	0.822	1.001	1.280	0.0525
SOMD P0C0L4 C4A C4B 2182 54 PASS	2234	0.826	0.778	0.876	2.78E-07	9.728	NA	NA	NA	NA	NA	NA
SOMD P0C0L5 C4A C4B 2182 54 PASS	2234	0.826	0.778	0.876	2.78E-07	9.728	NA	NA	NA	NA	NA	NA
SOMA_P22897_MRC1_2637_77	2234	1.215	1.144	1.289	2.82E-07	9.723	1077	1.260	1.195	1.328	17.052	<0.0001
SOMA_Q7LG56_RRM2B_8925_25	2234	1.202	1.136	1.272	2.90E-07	9.711	1077	0.911	0.820	1.011	1.105	0.0785
SOMA_Q13219_PAPPA_4148_49	2234	1.209	1.14	1.281	2.96E-07	9.702	1077	1.290	1.212	1.374	14.691	<0.0001
SOMA_P23468_PTPRD_9296_15	2234	0.831	0.784	0.88	2.96E-07	9.701	1077	0.761	0.679	0.852	5.610	<0.0001
SOMA_O95867_LY6G6C_6256_9	2234	1.199	1.134	1.269	3.04E-07	9.69	1077	1.018	0.921	1.126	0.141	0.723
SOMA_P39900_MMP12_4496_60	2234	1.211	1.142	1.285	3.12E-07	9.678	1077	1.092	0.999	1.194	1.270	0.0536
SOMA_Q02223_TNFRSF1_2665_26	2234	1.213	1.143	1.288	3.14E-07	9.676	1077	1.107	1.053	1.162	4.246	<0.0001
SOMA_Q9Y4X1_UGT2A1_8907_11	2234	0.828	0.781	0.878	3.18E-07	9.67	1077	0.673	0.591	0.767	8.641	<0.0001
SOMA_P01213_PDYN_19638_9	2234	0.83	0.784	0.879	3.21E-07	9.666	1077	0.674	0.604	0.752	11.758	<0.0001
SOMA_Q9UBP0_SPAST_8388_24	2234	0.822	0.774	0.873	3.26E-07	9.66	1077	1.039	0.979	1.103	0.695	0.2019
SOMA_Q6UXB4_CLEC4G_10781_19	2234	1.212	1.142	1.287	3.29E-07	9.656	1077	1.206	1.129	1.287	7.674	<0.0001

SOMA_Q9UNW1_MINPP1_5586_66	2234	1.217	1.145	1.293	3.44E-07	9.637	1077	1.345	1.255	1.442	16.297	<0.0001
SOMA_Q9BT09_CNPY3_17327_3	2234	1.209	1.14	1.281	3.48E-07	9.631	1077	1.193	1.110	1.282	5.800	<0.0001
SOMA_Q9UKY0_PRND_18231_147	2234	0.836	0.791	0.884	3.49E-07	9.63	1077	1.051	0.972	1.137	0.675	0.2115
SOMA_Q8WWA0_ITLN1_18830_1	2234	1.217	1.145	1.293	3.53E-07	9.625	1077	1.375	1.274	1.484	15.532	<0.0001
SOMA_P50452_SERPINB_19130_81	2234	1.207	1.139	1.279	3.54E-07	9.624	1077	1.099	1.037	1.165	2.838	0.0015
SOMA_P21964_COMT_18382_109	2234	0.831	0.784	0.88	3.64E-07	9.611	1077	0.563	0.473	0.669	10.127	<0.0001
SOMA_P61371_ISL1_11549_6	2234	0.84	0.795	0.886	3.69E-07	9.605	1077	1.073	1.006	1.146	1.475	0.0335
SOMA_Q9Y5Y6_ST14_10927_65	2234	0.865	0.827	0.905	3.74E-07	9.6	NA	NA	NA	NA	NA	NA
SOMA_P02654_APOC1_15364_101	2234	0.83	0.784	0.88	3.95E-07	9.576	1077	0.809	0.731	0.896	4.348	<0.0001
SOMA_Q6P4A8_PLBD1_6315_58	2234	1.202	1.135	1.272	4.08E-07	9.563	1077	0.991	0.898	1.095	0.064	0.8638
SOMA_P46531_NOTCH1_5107_7	2234	0.826	0.778	0.876	4.12E-07	9.558	1077	0.968	0.873	1.074	0.266	0.5424
SOMA_O15400_STX7_8274_64	2234	1.206	1.138	1.278	4.16E-07	9.554	1077	0.973	0.874	1.082	0.215	0.6092
SOMA_P27918_CFP_2960_66	2234	0.827	0.78	0.877	4.19E-07	9.55	1077	0.639	0.576	0.708	16.910	<0.0001
SOMA_Q9Y5Q6_INSL5_10462_14	2234	1.206	1.138	1.279	4.26E-07	9.543	1077	1.043	0.981	1.109	0.740	0.1818
SOMA_Q96BQ1_FAM3D_13102_1	2234	1.204	1.137	1.276	4.38E-07	9.531	1077	1.106	1.012	1.208	1.585	0.026
SOMA_P01042_KNG1_15343_337	2234	0.832	0.786	0.881	4.46E-07	9.523	1077	0.663	0.560	0.785	5.715	<0.0001
SOMA_Q9P0T7_TMEM9_8597_1	2234	0.838	0.793	0.885	4.52E-07	9.517	1077	0.750	0.669	0.841	6.035	<0.0001
SOMA_P01563_IFNA2_3497_13	2234	0.844	0.8	0.89	4.67E-07	9.503	1077	0.820	0.704	0.955	1.969	0.0107
SOMA_O15130_NPFF_5617_41	2234	0.828	0.781	0.878	4.77E-07	9.494	1077	0.936	0.759	1.155	0.268	0.5397

SOMA_Q8IZP7_HS6ST3_18896_23	2234	0.828	0.781	0.878	4.79E-07	9.492	1077	0.773	0.674	0.886	3.659	0.0002
SOMA_Q9Y3E1_HDGFRP3_18899_82	2234	1.197	1.132	1.266	4.99E-07	9.475	NA	NA	NA	NA	NA	NA
SOMA_Q9UKM7_MAN1B1_7071_23	2234	0.832	0.785	0.881	5.09E-07	9.466	1077	0.820	0.736	0.915	3.444	0.0004
SOMA_P15428_HPGD_4995_16	2234	0.825	0.776	0.876	5.15E-07	9.461	1077	0.696	0.579	0.836	3.965	0.0001
SOMA_Q96PB7_OLFM3_11143_32	2234	0.831	0.784	0.88	5.38E-07	9.442	1077	0.883	0.726	1.074	0.671	0.2132
SOMA_P00736_C1R_3285_23	2234	0.831	0.785	0.881	5.47E-07	9.435	1077	0.928	0.837	1.029	0.802	0.1576
SOMA_P17174_GOT1_4912_17	2234	0.827	0.779	0.878	5.50E-07	9.432	1077	0.721	0.633	0.822	5.996	<0.0001
SOMA_O15146_MUSK_11547_84	2234	0.83	0.783	0.88	5.66E-07	9.42	1077	0.752	0.589	0.960	1.657	0.022
SOMA_Q9BY79_MFRP_3685_53	2234	1.198	1.132	1.267	5.97E-07	9.397	1077	0.964	0.816	1.139	0.175	0.6681
SOMA_P11684_SCGB1A1_10569_28	2234	1.202	1.135	1.273	6.07E-07	9.39	NA	NA	NA	NA	NA	NA
SOMA_Q06481_APLP2_10627_87	2234	1.202	1.134	1.273	6.71E-07	9.346	1077	1.079	0.984	1.183	0.975	0.1058
SOMA_Q14289_PTK2B_8918_64	2234	0.832	0.785	0.881	6.76E-07	9.343	1077	0.721	0.617	0.841	4.486	<0.0001
SOMA_P19320_VCAM1_2967_8	2234	1.211	1.14	1.286	7.06E-07	9.324	1077	1.294	1.198	1.399	10.211	<0.0001
SOMA_O43567_RNF13_8087_250	2234	0.83	0.782	0.88	7.11E-07	9.321	1077	0.558	0.436	0.714	5.431	<0.0001
SOMA_Q86UL8_MAGI2_14066_49	2234	1.201	1.133	1.272	7.29E-07	9.31	1077	0.966	0.850	1.098	0.225	0.595
SOMA_Q8N474_SFRP1_3221_54	2234	1.219	1.145	1.297	7.40E-07	9.304	1077	1.293	1.213	1.379	14.423	<0.0001
SOMA_P84022_SMAD3_10363_13	2234	0.838	0.793	0.886	7.64E-07	9.29	1077	0.805	0.729	0.889	4.739	<0.0001
SOMA_O75556_SCGB2A1_5001_6	2234	0.83	0.783	0.88	7.66E-07	9.289	1077	0.669	0.556	0.805	4.692	<0.0001
SOMA_Q08ET2_SIGLEC1_8248_222	2234	1.208	1.138	1.282	7.65E-07	9.289	1077	1.360	1.255	1.473	13.301	<0.0001

SOMA_Q6Q788_APOA5_15363_32	2234	0.827	0.779	0.878	8.44E-07	9.246	1077	0.698	0.607	0.803	6.309	<0.0001
SOMA_Q9NPJ6_MED4_14021_81	2234	0.828	0.78	0.879	8.62E-07	9.237	1077	0.817	0.659	1.013	1.182	0.0657
SOMA_P27216_ANXA13_17835_28	2234	0.831	0.784	0.881	8.70E-07	9.233	1077	0.865	0.551	1.360	0.275	0.5306
SOMA_P13726_F3_4931_59	2234	1.198	1.131	1.268	8.73E-07	9.232	1077	1.068	1.017	1.121	2.082	0.0083
SOMA_O60565_GREM1_18878_15	2234	1.2	1.133	1.271	9.01E-07	9.218	1077	1.105	1.054	1.159	4.409	<0.0001
SOMA_P13501_CCL5_5480_49	2234	0.836	0.79	0.885	9.02E-07	9.218	1077	0.655	0.586	0.733	12.782	<0.0001
SOMA_Q96S55_WRNIP1_12528_40	2234	0.834	0.787	0.883	9.05E-07	9.216	1077	0.738	0.466	1.170	0.708	0.1958
SOMA_Q92804_TAF15_16865_62	2234	1.196	1.13	1.266	9.44E-07	9.198	1077	0.987	0.890	1.094	0.097	0.7996
SOMA_P46459_NSF_13992_12	2234	0.829	0.781	0.88	1.02E-06	9.165	1077	0.772	0.659	0.903	2.894	0.0013
SOMA_Q4G148_GXYLT1_8229_1	2234	0.832	0.785	0.882	1.05E-06	9.151	1077	0.873	0.740	1.029	0.978	0.1053
SOMA_Q9NZC2_TREM2_16300_4	2234	1.203	1.134	1.276	1.06E-06	9.15	1077	1.225	1.119	1.340	4.993	<0.0001
SOMA_P52209_PGD_4187_49	2234	0.83	0.782	0.881	1.17E-06	9.106	1077	0.815	0.730	0.910	3.541	0.0003
SOMA_P23443_RPS6KB1_8962_48	2234	0.828	0.78	0.88	1.18E-06	9.102	1077	0.876	0.731	1.050	0.821	0.151
SOMA_P61964_WDR5_17760_128	2234	1.202	1.133	1.274	1.23E-06	9.084	1077	1.047	0.955	1.148	0.479	0.3321
SOMA_Q04609_FOLH1_5478_50	2234	1.196	1.13	1.266	1.23E-06	9.084	1077	1.111	1.047	1.180	3.298	0.0005
SOMA_O95825_CRYZL1_9207_60	2234	0.832	0.784	0.882	1.34E-06	9.046	1077	0.737	0.626	0.868	3.599	0.0003
SOMA_O95831_AIFM1_13424_51	2234	1.196	1.13	1.267	1.41E-06	9.025	NA	NA	NA	NA	NA	NA
SOMA_O43921_EFNA2_14124_6	2234	1.201	1.133	1.274	1.41E-06	9.023	1077	1.178	1.105	1.257	6.234	<0.0001
SOMA_O95998_IL18BP_3073_51	2234	1.205	1.135	1.28	1.42E-06	9.02	1077	1.314	1.224	1.409	13.539	<0.0001

SOMA_Q9H6X2_ANTXR1_10464_6	2234	0.84	0.795	0.889	1.44E-06	9.014	1077	0.501	0.379	0.663	5.890	<0.0001
SOMA_Q7Z2E3_APTX_17736_105	2234	0.848	0.805	0.894	1.45E-06	9.011	1077	0.783	0.634	0.967	1.641	0.0229
SOMA_Q9UJA9_ENPP5_6556_5	2234	0.839	0.793	0.888	1.46E-06	9.008	1077	0.890	0.802	0.988	1.538	0.029
SOMA_Q5VVJ2_MYSM1_11536_9	2234	0.833	0.785	0.883	1.47E-06	9.006	1077	0.445	0.256	0.773	2.388	0.0041
SOMA_Q92688_ANP32B_4194_26	2234	1.202	1.133	1.275	1.57E-06	8.976	1077	1.104	1.046	1.164	3.554	0.0003
SOMA_Q9UEW3_MARCO_9003_99	2234	0.853	0.81	0.898	1.70E-06	8.942	1077	0.813	0.697	0.948	2.075	0.0084
SOMA_Q6P5S2_LEG1_7154_92	2234	0.836	0.789	0.885	1.71E-06	8.939	1077	0.827	0.736	0.929	2.857	0.0014
SOMA_P78346_RPP30_17511_10	2234	0.847	0.803	0.894	1.79E-06	8.92	1077	0.698	0.510	0.955	1.610	0.0245
SOMA_O95631_NTN1_6649_51	2234	1.207	1.136	1.283	1.84E-06	8.908	1077	1.209	1.131	1.292	7.663	<0.0001
SOMA_P55001_MFAP2_9294_45	2234	1.196	1.129	1.267	1.88E-06	8.899	1077	1.038	0.982	1.097	0.729	0.1868
SOMA_P48740_MASP1_3605_77	2234	0.835	0.787	0.885	1.92E-06	8.89	1077	0.988	0.886	1.101	0.085	0.8227
SOMA_P27707_DCK_9836_20	2234	0.881	0.846	0.918	1.98E-06	8.877	1077	0.777	0.628	0.963	1.676	0.0211
SOMA_Q8IZJ1_UNC5B_15394_79	2234	1.199	1.131	1.272	1.98E-06	8.877	1077	1.202	1.124	1.286	7.107	<0.0001
SOMD P02671 FGA FGB 279662 PASS	2234	1.192	1.126	1.262	2.03E-06	8.866	NA	NA	NA	NA	NA	NA
SOMD P02675 FGA FGB 279662 PASS	2234	1.192	1.126	1.262	2.03E-06	8.866	NA	NA	NA	NA	NA	NA
SOMD P02679 FGA FGB 279662 PASS	2234	1.192	1.126	1.262	2.03E-06	8.866	NA	NA	NA	NA	NA	NA
SOMA_Q9NQX5_NPDC1_10424_31	2234	1.2	1.131	1.273	2.04E-06	8.864	1077	1.109	1.056	1.165	4.409	<0.0001
SOMA_Q92832_NELL1_6544_33	2234	0.836	0.789	0.886	2.25E-06	8.82	1077	0.868	0.729	1.032	0.962	0.1093
SOMA_P06127_CD5_14065_11	2234	0.832	0.784	0.883	2.33E-06	8.806	1077	0.972	0.846	1.116	0.165	0.6834

SOMA_Q86VB7_CD163_5028_59	2234	1.2	1.131	1.274	2.44E-06	8.785	1077	1.406	1.293	1.529	14.757	<0.0001
SOMA_P04843_RPN1_10490_3	2234	1.193	1.127	1.264	2.46E-06	8.781	1077	1.022	0.950	1.099	0.249	0.564
SOMA_P27348_YWHAQ_7625_27	2234	1.194	1.127	1.265	2.64E-06	8.752	1077	0.896	0.790	1.017	1.047	0.0898
SOMA_Q99075_HBEGF_14094_29	2234	0.837	0.79	0.887	2.64E-06	8.751	1077	0.690	0.610	0.779	8.589	<0.0001
SOMA_Q12913_PTPRJ_8250_2	2234	0.837	0.79	0.887	2.72E-06	8.739	1077	0.839	0.733	0.961	1.947	0.0113
SOMA_Q96LA6_FCRL1_5728_60	2234	0.834	0.786	0.885	2.77E-06	8.731	1077	0.859	0.749	0.985	1.523	0.03
SOMA_Q5JXM2_METTL24_9470_15	2234	1.194	1.127	1.265	2.97E-06	8.7	1077	1.130	1.060	1.204	3.778	0.0002
SOMA_P37837_TALDO1_11347_9	2234	1.204	1.133	1.279	3.15E-06	8.675	1077	1.177	1.080	1.283	3.665	0.0002
SOMA_P60953_CDC42_9840_2	2234	0.836	0.789	0.887	3.16E-06	8.673	1077	0.820	0.745	0.903	4.266	<0.0001
SOMA_Q8N423_LILRB2_5091_28	2234	1.187	1.122	1.255	3.20E-06	8.668	1077	1.173	1.111	1.239	7.963	<0.0001
SOMA_Q9HD15_SRA1_18220_141	2234	1.196	1.128	1.268	3.20E-06	8.668	1077	1.125	1.033	1.226	2.161	0.0069
SOMA_O95379_TNFAIP8_12563_2	2234	0.83	0.781	0.882	3.20E-06	8.667	1077	0.682	0.582	0.800	5.625	<0.0001
SOMA_Q6IA17_SIGIRR_8326_63	2234	0.837	0.79	0.887	3.23E-06	8.663	1077	1.024	0.937	1.119	0.219	0.6037
SOMA_P30153_PPP2R1A_12621_55	2234	0.834	0.786	0.885	3.24E-06	8.662	1077	0.919	0.829	1.019	0.962	0.1092
SOMA_Q13740_ALCAM_5451_1	2234	1.203	1.132	1.278	3.28E-06	8.658	1077	1.260	1.181	1.345	11.502	<0.0001
SOMA_Q9Y5U2_TSSC4_18181_2	2234	1.198	1.129	1.271	3.49E-06	8.63	1077	0.947	0.856	1.049	0.528	0.2963
SOMA_Q07444_KLRC3_11571_75	2234	0.832	0.783	0.883	3.69E-06	8.605	1077	0.799	0.578	1.105	0.755	0.1757
SOMA_Q8N912_NRAC_13464_8	2234	0.833	0.785	0.885	3.83E-06	8.59	1077	0.938	0.799	1.101	0.361	0.4358
SOMA_Q13506_NAB1_13933_276	2234	0.834	0.786	0.886	3.84E-06	8.589	1077	0.864	0.747	1.001	1.293	0.0509

SOMA_Q9NRR1_CYTL1_8402_22	2234	1.193	1.126	1.264	3.89E-06	8.583	1077	1.186	1.092	1.289	4.289	<0.0001
SOMA_Q9UPQ9_TNRC6B_9808_41	2234	1.193	1.125	1.264	4.05E-06	8.565	1077	1.036	0.940	1.141	0.322	0.4764
SOMA_Q96P31_FCRL3_4440_15	2234	1.192	1.125	1.262	4.06E-06	8.564	1077	1.045	0.991	1.101	0.977	0.1054
SOMA_P17693_HLA_G_19636_23	2234	1.19	1.124	1.261	4.09E-06	8.561	1077	1.211	1.137	1.290	8.569	<0.0001
SOMA_Q15121_PEA15_18833_76	2234	1.193	1.126	1.264	4.10E-06	8.561	1077	1.067	1.013	1.124	1.854	0.014
SOMA_P36776_LONP1_6398_12	2234	1.188	1.122	1.257	4.18E-06	8.552	1077	0.849	0.763	0.945	2.554	0.0028
SOMA_P04180_LCAT_15413_3	2234	0.833	0.785	0.885	4.42E-06	8.528	1077	1.116	1.047	1.189	3.099	0.0008
SOMA_P24310_COX7A1_8390_25	2234	0.839	0.791	0.889	4.49E-06	8.52	1077	1.027	0.935	1.128	0.237	0.5797
SOMA_Q8WUM4_PDCD6IP_18174_79	2234	0.836	0.788	0.887	4.70E-06	8.501	1077	0.753	0.664	0.855	4.915	<0.0001
SOMA_Q6UWS5_PET117_19303_64	2234	1.202	1.131	1.278	4.94E-06	8.479	1077	1.247	1.157	1.343	8.164	<0.0001
SOMA_P18075_BMP7_2972_57	2234	1.195	1.126	1.267	5.05E-06	8.47	1077	1.020	0.953	1.091	0.248	0.5648
SOMA_P0DMV8_HSPA1A_4124_24	2234	1.19	1.123	1.261	5.08E-06	8.467	1077	1.305	1.215	1.402	12.577	<0.0001
SOMA_Q9UKW4_VAV3_9830_109	2234	0.837	0.789	0.888	5.48E-06	8.434	1077	0.622	0.538	0.719	9.795	<0.0001
SOMA_Q9UN86_G3BP2_9831_12	2234	1.187	1.121	1.256	5.59E-06	8.426	1077	0.960	0.869	1.061	0.370	0.4264
SOMA_O60383_GDF9_3067_67	2234	1.181	1.118	1.249	5.66E-06	8.42	1077	1.170	1.082	1.265	4.075	<0.0001
SOMA_A8MVW0_FAM171A_13479_8	2234	0.838	0.79	0.889	5.79E-06	8.41	1077	0.826	0.702	0.973	1.656	0.0221
SOMA_Q96C10_DHX58_14012_17	2234	0.835	0.786	0.887	5.93E-06	8.4	1077	1.072	0.997	1.152	1.224	0.0596
SOMA_P58499_FAM3B_9177_6	2234	1.193	1.125	1.266	6.15E-06	8.384	1077	1.195	1.102	1.295	4.783	<0.0001
SOMA_Q4KMG0_CDON_4541_49	2234	0.839	0.791	0.889	6.51E-06	8.36	1077	0.832	0.743	0.931	2.855	0.0014

SOMA_O75821_EIF3G_11454_87	2234	1.186	1.12	1.255	6.54E-06	8.357	1077	0.894	0.811	0.985	1.617	0.0242
SOMA_Q9UBM8_MGAT4C_7208_60	2234	1.186	1.12	1.255	6.61E-06	8.352	1077	1.034	0.963	1.109	0.448	0.3567
SOMA_P68402_PAFAH1B_2642_4	2234	1.188	1.121	1.258	6.67E-06	8.349	1077	1.095	1.025	1.170	2.156	0.007
SOMA_P29323_EPHB2_8225_86	2234	1.192	1.124	1.265	6.98E-06	8.329	1077	1.244	1.159	1.336	8.811	<0.0001
SOMA_Q9BW91_NUDT9_9482_110	2234	1.195	1.126	1.269	6.98E-06	8.329	1077	1.036	0.936	1.147	0.304	0.4962
SOMA_Q9NQ75_CASS4_12855_16	2234	0.835	0.787	0.887	7.06E-06	8.324	1077	0.870	0.774	0.979	1.688	0.0205
SOMA_P09972_ALDOC_9876_20	2234	0.835	0.786	0.887	7.12E-06	8.32	1077	0.992	0.876	1.124	0.044	0.9046
SOMA_Q6UWV6_ENPP7_4435_66	2234	1.194	1.125	1.267	7.22E-06	8.314	1077	1.308	1.222	1.401	14.016	<0.0001
SOMA_Q99674_CGREF1_6257_56	2234	1.196	1.126	1.27	7.25E-06	8.312	1077	1.193	1.127	1.264	8.792	<0.0001
SOMA_P52803_EFNA5_2615_60	2234	1.193	1.125	1.266	7.31E-06	8.309	1077	1.182	1.108	1.262	6.345	<0.0001
SOMA_P06729_CD2_7100_31	2234	1.184	1.119	1.253	7.44E-06	8.302	1077	1.262	1.172	1.358	9.207	<0.0001
SOMA_P07477_PRSS1_3049_61	2234	1.191	1.123	1.263	7.66E-06	8.289	1077	1.099	1.013	1.192	1.646	0.0226
SOMA_Q9GZZ8_LACRT_7163_26	2234	0.84	0.792	0.89	7.82E-06	8.28	1077	0.803	0.707	0.911	3.165	0.0007
SOMA_O15537_RS1_6497_10	2234	0.834	0.785	0.887	7.83E-06	8.279	1077	0.904	0.756	1.081	0.569	0.2697
SOMA_O00585_CCL21_2516_57	2234	1.189	1.122	1.26	7.87E-06	8.277	1077	1.364	1.277	1.457	19.626	<0.0001
SOMA_P07988_SFTPB_10672_75	2234	1.192	1.124	1.265	8.11E-06	8.264	1077	1.162	1.101	1.226	7.332	<0.0001
SOMA_O14641_DVL2_13575_40	2234	0.84	0.792	0.89	8.16E-06	8.261	1077	0.880	0.795	0.974	1.867	0.0136
SOMA_Q9UBT3_DKK4_3365_7	2234	0.84	0.792	0.891	8.22E-06	8.258	1077	0.811	0.719	0.914	3.211	0.0006
SOMA_Q13291_SLAMF1_7953_20	2234	1.19	1.122	1.261	8.70E-06	8.233	1077	1.151	1.101	1.204	9.222	<0.0001

SOMA_P14207_FOLR2_15587_20	2234	1.194	1.125	1.267	8.91E-06	8.223	1077	1.183	1.121	1.248	8.986	<0.0001
SOMA_P20618_PSMB1_12612_37	2234	1.184	1.118	1.253	9.06E-06	8.216	1077	1.272	1.185	1.366	10.488	<0.0001
SOMA_O60704_TPST2_8024_64	2234	0.842	0.794	0.892	9.32E-06	8.203	1077	0.703	0.615	0.805	6.539	<0.0001
SOMA_P43251_BTD_15644_1	2234	0.842	0.795	0.892	9.50E-06	8.195	1077	0.734	0.659	0.817	7.741	<0.0001
SOMA_Q86UD1_OAF_6414_8	2234	1.178	1.114	1.245	9.82E-06	8.181	1077	1.407	1.282	1.543	12.297	<0.0001
SOMA_Q08830_FGL1_5581_28	2234	1.19	1.122	1.262	1.01E-05	8.167	1077	1.403	1.292	1.522	15.241	<0.0001
SOMA_P0DMM9_SULT1A3_13944_3	2234	0.842	0.795	0.893	1.02E-05	8.165	1077	0.707	0.634	0.789	9.205	<0.0001
SOMA_Q13162_PRDX4_7789_182	2234	1.183	1.117	1.252	1.03E-05	8.162	1077	1.040	0.965	1.122	0.516	0.3048
SOMA_Q13522_PPP1R1A_17706_4	2234	1.193	1.124	1.266	1.03E-05	8.161	1077	1.157	1.091	1.227	5.883	<0.0001
SOMA_Q9UEF7_KL_15384_15	2234	0.842	0.794	0.892	1.06E-05	8.148	1077	0.957	0.856	1.069	0.362	0.4342
SOMA_P16949_STMN1_17367_5	2234	1.191	1.122	1.263	1.08E-05	8.139	1077	1.044	0.959	1.136	0.494	0.3209
SOMA_Q9BZX2_UCK2_12515_45	2234	0.847	0.8	0.896	1.10E-05	8.131	1077	1.008	0.915	1.111	0.058	0.8744
SOMA_P04155_TFF1_9185_15	2234	1.192	1.123	1.266	1.12E-05	8.125	1077	1.294	1.197	1.399	10.100	<0.0001
SOMA_Q14126_DSG2_9484_75	2234	1.192	1.123	1.265	1.13E-05	8.118	1077	1.412	1.300	1.533	15.670	<0.0001
SOMA_Q8N5H7_SH2D3C_12704_26	2234	0.839	0.791	0.891	1.14E-05	8.115	1077	0.701	0.524	0.938	1.774	0.0168
SOMA_P04275_VWF_3050_7	2234	1.183	1.117	1.252	1.21E-05	8.091	1077	1.409	1.302	1.525	16.844	<0.0001
SOMA_O15460_P4HA2_11348_132	2234	0.838	0.789	0.89	1.22E-05	8.085	1077	0.956	0.817	1.119	0.239	0.577
SOMA_P60981_DSTN_18883_4	2234	0.845	0.798	0.895	1.22E-05	8.085	1077	0.717	0.650	0.790	10.747	<0.0001
SOMA_P42680_TEC_16079_2	2234	0.847	0.8	0.896	1.23E-05	8.084	1077	0.746	0.671	0.830	7.159	<0.0001

SOMA_Q8N5G2_TMEM57_12367_52	2234	0.84	0.791	0.891	1.24E-05	8.079	NA	NA	NA	NA	NA	NA
SOMA_P06681_C2_3186_2	2234	1.189	1.121	1.262	1.24E-05	8.078	1077	1.398	1.267	1.543	10.603	<0.0001
SOMA_P05154_SERPINA_3389_7	2234	0.845	0.798	0.895	1.28E-05	8.065	1077	0.624	0.570	0.683	23.770	<0.0001
SOMA_O15204_ADAMDEC_9115_78	2234	1.179	1.115	1.247	1.28E-05	8.064	1077	1.093	1.017	1.174	1.826	0.0149
SOMA_Q8N3Z0_PRSS35_9983_97	2234	0.847	0.8	0.896	1.31E-05	8.056	1077	0.667	0.374	1.190	0.768	0.1708
SOMA_Q7Z434_MAVS_8783_216	2234	1.187	1.12	1.259	1.32E-05	8.051	NA	NA	NA	NA	NA	NA
SOMA_O43704_SULT1B1_12671_35	2234	0.843	0.795	0.893	1.33E-05	8.049	1077	1.016	0.913	1.131	0.113	0.7715
SOMA_Q9H4P4_RNF41_13411_21	2234	1.179	1.115	1.248	1.33E-05	8.048	1077	1.048	0.946	1.161	0.434	0.3684
SOMA_Q13283_G3BP1_19266_35	2234	1.185	1.118	1.255	1.35E-05	8.044	1077	0.897	0.814	0.989	1.527	0.0297
SOMA_Q9BQF6_SENP7_12626_6	2234	0.839	0.791	0.891	1.34E-05	8.044	1077	1.062	0.994	1.135	1.121	0.0756
SOMA_P05546_SERPIND_3316_58	2234	0.841	0.793	0.892	1.35E-05	8.043	1077	0.707	0.637	0.784	10.351	<0.0001
SOMA_Q8NBJ7_SUMF2_6069_71	2234	1.187	1.119	1.258	1.37E-05	8.037	1077	1.183	1.117	1.253	8.043	<0.0001
SOMA_P08311_CTSG_2431_17	2234	1.181	1.116	1.25	1.50E-05	7.997	1077	1.116	1.034	1.205	2.317	0.0048
SOMA_P49913_CAMP_9384_17	2234	0.838	0.789	0.89	1.50E-05	7.997	1077	0.939	0.843	1.047	0.592	0.2558
SOMA_P08254_MMP3_2788_55	2234	0.839	0.79	0.891	1.53E-05	7.987	1077	0.351	0.203	0.608	3.733	0.0002
SOMA_Q14118_DAG1_8369_102	2234	1.182	1.116	1.251	1.54E-05	7.984	1077	1.087	1.034	1.143	2.943	0.0011
SOMA_P17948_FLT1_16315_105	2234	1.198	1.126	1.274	1.60E-05	7.969	1077	1.115	1.061	1.172	4.701	<0.0001
SOMA_Q9Y6W8_ICOS_14084_191	2234	1.185	1.118	1.256	1.76E-05	7.928	NA	NA	NA	NA	NA	NA
SOMA_Q9Y646_CPQ_9394_19	2234	0.845	0.798	0.896	1.76E-05	7.926	1077	0.839	0.754	0.935	2.841	0.0014

SOMA_Q86U17_SERPINA_9002_36	2234	1.192	1.122	1.267	1.89E-05	7.897	1077	1.614	1.467	1.775	22.097	<0.0001
SOMA_P0C0S5_H2AFZ_4163_5	2234	1.179	1.114	1.248	1.92E-05	7.89	NA	NA	NA	NA	NA	NA
SOMA_Q15768_EFNB3_7785_1	2234	0.847	0.799	0.897	1.95E-05	7.883	1077	1.018	0.930	1.115	0.159	0.694
SOMA_Q96JJ6_JPH4_9111_40	2234	0.844	0.796	0.895	1.98E-05	7.877	1077	1.039	0.966	1.118	0.516	0.3047
SOMA_Q96GA7_SDSL_17777_31	2234	1.189	1.12	1.262	2.05E-05	7.86	1077	0.958	0.850	1.079	0.323	0.4758
SOMA_P81277_PRLH_6543_182	2234	0.844	0.796	0.895	2.12E-05	7.847	1077	1.007	0.902	1.125	0.047	0.8976
SOMA_O43557_TNFSF14_5988_49	2234	0.842	0.793	0.894	2.24E-05	7.822	1077	0.794	0.619	1.018	1.160	0.0691
SOMA_Q9UN76_SLC6A14_13053_6	2234	0.867	0.825	0.911	2.26E-05	7.818	1077	0.542	0.423	0.694	5.879	<0.0001
SOMA_O14773_TPP1_17691_1	2234	1.188	1.119	1.262	2.28E-05	7.815	1077	1.038	0.972	1.109	0.575	0.2663
SOMA_P30305_CDC25B_12427_8	2234	0.866	0.824	0.91	2.28E-05	7.814	1077	0.677	0.555	0.825	3.943	0.0001
SOMA_O95841_ANGPTL1_11142_11	2234	1.182	1.115	1.252	2.34E-05	7.803	1077	1.180	1.114	1.250	7.808	<0.0001
SOMA_Q9UBP8_KAAG1_19492_5	2234	0.848	0.8	0.898	2.36E-05	7.8	1077	0.991	0.868	1.130	0.052	0.8876
SOMA_Q08629_SPOCK1_5490_53	2234	0.841	0.792	0.893	2.40E-05	7.792	1077	0.889	0.772	1.025	0.978	0.1052
SOMA_P62318_SNRPD3_17774_38	2234	0.841	0.792	0.893	2.43E-05	7.787	1077	0.836	0.690	1.012	1.183	0.0656
SOMA_P02774_GC_15589_1	2234	0.847	0.8	0.898	2.60E-05	7.757	1077	0.898	0.806	1.000	1.307	0.0493
SOMA_P18627_LAG3_9950_229	2234	1.186	1.118	1.259	2.65E-05	7.75	1077	1.063	0.972	1.163	0.742	0.181
SOMA_P12259_F5_4906_35	2234	0.851	0.804	0.9	2.68E-05	7.745	1077	0.702	0.630	0.783	9.790	<0.0001
SOMA_Q92729_PTPRU_8337_65	2234	1.186	1.118	1.259	2.71E-05	7.74	1077	1.266	1.157	1.384	6.620	<0.0001
SOMA_P58417_NXPH1_4562_1	2234	0.846	0.798	0.897	2.89E-05	7.712	1077	0.749	0.670	0.837	6.449	<0.0001

SOMA_Q00613_HSF1_11616_9	2234	1.176	1.111	1.245	2.91E-05	7.709	1077	1.026	0.969	1.088	0.422	0.3787
SOMA_Q8N5Y8_PARP16_7881_244	2234	0.845	0.797	0.896	2.93E-05	7.706	1077	0.987	0.865	1.126	0.073	0.8461
SOMA_P01730_CD4_3143_3	2234	1.174	1.11	1.241	2.94E-05	7.705	1077	1.067	0.984	1.158	0.934	0.1164
SOMA_Q15637_SF1_12777_11	2234	1.176	1.111	1.245	3.07E-05	7.685	1077	1.019	0.938	1.107	0.187	0.6499
SOMA_Q92851_CASP10_5340_24	2234	0.842	0.793	0.894	3.10E-05	7.682	1077	1.039	0.979	1.103	0.692	0.2032
SOMA_Q9Y4D7_PLXND1_19561_216	2234	1.183	1.115	1.255	3.09E-05	7.682	1077	1.312	1.213	1.418	10.937	<0.0001
SOMA_Q96NW4_ANKRD27_12445_50	2234	0.847	0.799	0.897	3.12E-05	7.679	1077	1.024	0.943	1.112	0.239	0.5772
SOMA_P46108_CRK_4976_57	2234	1.182	1.115	1.254	3.12E-05	7.678	1077	1.004	0.909	1.110	0.030	0.9327
SOMA_O75078_ADAM11_6586_19	2234	1.183	1.115	1.254	3.17E-05	7.672	1077	1.098	1.035	1.164	2.730	0.0019
SOMA_Q9ULS5_TMCC3_8922_4	2234	0.842	0.793	0.895	3.28E-05	7.657	1077	0.718	0.623	0.828	5.254	<0.0001
SOMA_P02765_AHSG_3581_53	2234	0.847	0.799	0.898	3.52E-05	7.626	1077	1.016	0.923	1.118	0.125	0.7496
SOMA_Q9NPH9_IL26_16760_2	2234	0.846	0.798	0.897	3.53E-05	7.625	1077	0.513	0.395	0.667	6.212	<0.0001
SOMA_P19338_NCL_13655_34	2234	1.176	1.111	1.245	3.57E-05	7.62	1077	1.053	0.984	1.127	0.875	0.1334
SOMA_O76095_JTB_9038_12	2234	1.185	1.116	1.258	3.59E-05	7.618	1077	1.127	1.065	1.192	4.493	<0.0001
SOMA_O60909_B4GALT2_9595_11	2234	0.849	0.802	0.9	3.81E-05	7.591	1077	0.902	0.770	1.057	0.696	0.2014
SOMA_O14543_SOCS3_11440_58	2234	0.843	0.794	0.895	3.83E-05	7.59	1077	0.689	0.531	0.893	2.317	0.0048
SOMA_O60235_TMPRSS1_6547_83	2234	0.845	0.796	0.897	3.91E-05	7.581	1077	0.934	0.803	1.088	0.419	0.3811
SOMA_Q15181_PPA1_5021_13	2234	0.843	0.794	0.896	4.05E-05	7.565	1077	0.832	0.726	0.954	2.067	0.0086
SOMA_Q9HCN8_SDF2L1_6990_44	2234	1.181	1.113	1.252	4.06E-05	7.565	1077	1.052	0.981	1.128	0.805	0.1566

SOMA_P04264_KRT1_9931_20	2234	1.182	1.115	1.254	4.08E-05	7.562	1077	1.189	1.124	1.257	8.932	<0.0001
SOMA_Q9NVA2_SEPT11_12620_3	2234	1.181	1.114	1.252	4.09E-05	7.561	NA	NA	NA	NA	NA	NA
SOMA_Q6PUV4_CPLX2_15321_8	2234	1.183	1.115	1.255	4.11E-05	7.559	1077	1.125	1.064	1.190	4.437	<0.0001
SOMA_Q8N6T3_ARFGAP1_11556_19	2234	0.846	0.797	0.897	4.11E-05	7.559	1077	0.636	0.550	0.736	8.945	<0.0001
SOMA_Q9UHI8_ADAMTS1_3174_2	2234	1.172	1.108	1.24	4.31E-05	7.539	1077	1.008	0.926	1.098	0.069	0.8532
SOMA_P09012_SNRPA_12678_66	2234	1.17	1.107	1.237	4.55E-05	7.515	1077	0.999	0.899	1.110	0.009	0.9792
SOMA_O00391_QSOX1_6070_11	2234	0.853	0.807	0.903	4.57E-05	7.513	NA	NA	NA	NA	NA	NA
SOMA_Q9Y458_TBX22_11146_4	2234	1.174	1.109	1.242	4.67E-05	7.504	1077	1.061	0.996	1.130	1.169	0.0678
SOMA_Q8TDN1_KCNG4_13525_17	2234	0.85	0.802	0.9	4.73E-05	7.498	1077	0.885	0.761	1.030	0.942	0.1144
SOMA_P53814_SMTN_12546_1	2234	1.173	1.108	1.241	4.80E-05	7.492	1077	0.876	0.724	1.061	0.754	0.1761
SOMA_Q6UWE3_CLPSL2_7767_1	2234	0.85	0.802	0.9	4.87E-05	7.485	1077	0.972	0.854	1.107	0.174	0.6705
SOMA_Q9C075_KRT23_14237_1	2234	0.845	0.795	0.897	4.96E-05	7.477	NA	NA	NA	NA	NA	NA
SOMA_P00441_SOD1_2794_60	2234	1.17	1.106	1.237	5.40E-05	7.441	1077	1.095	1.006	1.193	1.442	0.0361
SOMA_O15068_MCF2L_13934_3	2234	0.85	0.803	0.901	5.49E-05	7.434	1077	0.946	0.821	1.091	0.351	0.4457
SOMA_P05543_SERPINA_2706_69	2234	0.848	0.8	0.899	5.72E-05	7.416	1077	0.880	0.793	0.975	1.833	0.0147
SOMA_P12273_PIP_6060_2	2234	0.851	0.803	0.901	5.80E-05	7.409	1077	0.828	0.735	0.933	2.706	0.002
SOMA_P16403_HIST1H1_2987_37	2234	1.167	1.104	1.233	6.14E-05	7.385	NA	NA	NA	NA	NA	NA
SOMA_P03951_F11_2190_55	2234	0.846	0.797	0.898	6.15E-05	7.384	1077	0.704	0.633	0.784	9.912	<0.0001
SOMA_Q9NSY2_STARD5_17383_4	2234	1.175	1.109	1.245	6.23E-05	7.379	1077	1.004	0.909	1.108	0.026	0.942

SOMA_P31994_FCGR2B_3310_62	2234	1.178	1.111	1.249	6.24E-05	7.378	1077	1.258	1.157	1.367	7.143	<0.0001
SOMA_Q8TBP5_FAM174A_6597_24	2234	0.847	0.798	0.899	6.28E-05	7.375	1077	0.553	0.343	0.890	1.833	0.0147
SOMA_Q92765_FRZB_13740_51	2234	1.19	1.118	1.266	6.40E-05	7.366	1077	1.091	1.034	1.151	2.862	0.0014
SOMA_P58340_MLF1_17820_170	2234	0.85	0.802	0.901	6.69E-05	7.347	1077	0.897	0.763	1.055	0.721	0.1901
SOMA_P57087_JAM2_2997_8	2234	1.179	1.111	1.251	6.90E-05	7.334	1077	1.140	1.061	1.226	3.433	0.0004
SOMA_P09486_SPARC_3043_49	2234	0.853	0.806	0.903	7.13E-05	7.32	1077	0.701	0.630	0.781	10.018	<0.0001
SOMA_Q9NRA0_SPHK2_4468_21	2234	0.872	0.83	0.916	7.15E-05	7.318	1077	0.772	0.631	0.943	1.945	0.0113
SOMA_Q9UBP6_METTL1_12514_16	2234	0.846	0.797	0.899	7.32E-05	7.309	1077	0.730	0.591	0.901	2.462	0.0035
SOMA_O15072_ADAMTS3_8845_2	2234	0.847	0.798	0.899	7.43E-05	7.302	1077	0.923	0.784	1.087	0.470	0.3386
SOMA_P33763_S100A5_15303_63	2234	0.857	0.811	0.906	7.56E-05	7.295	1077	0.799	0.716	0.891	4.230	<0.0001
SOMA_Q13045_FLII_12677_164	2234	1.165	1.103	1.23	7.71E-05	7.286	1077	0.722	0.630	0.827	5.571	<0.0001
SOMA_Q6UW56_ATRAID_10823_19	2234	1.174	1.108	1.244	8.06E-05	7.266	NA	NA	NA	NA	NA	NA
SOMA_Q96P66_GPR101_11371_1	2234	0.85	0.801	0.901	8.15E-05	7.262	1077	0.881	0.704	1.103	0.570	0.2693
SOMA_Q8N104_DEFB106_5664_57	2234	0.848	0.799	0.9	8.22E-05	7.258	1077	1.012	0.923	1.109	0.094	0.8059
SOMA_Q86VZ4_LRP11_6713_4	2234	1.179	1.111	1.251	8.30E-05	7.254	1077	1.270	1.190	1.356	12.234	<0.0001
SOMA_Q9H3S3_TMPRSS5_8002_27	2234	0.872	0.83	0.916	8.60E-05	7.238	1077	0.639	0.303	1.348	0.620	0.2399
SOMA_Q9P244_LRFN1_7910_41	2234	0.85	0.801	0.901	8.73E-05	7.232	1077	0.714	0.540	0.945	1.735	0.0184
SOMA_Q6UX15_LAYN_2635_61	2234	1.177	1.109	1.248	9.01E-05	7.218	1077	1.058	1.006	1.113	1.564	0.0273
SOMA_O75973_C1QL1_6404_20	2234	1.186	1.115	1.261	9.46E-05	7.197	1077	1.123	1.064	1.185	4.605	<0.0001

SOMA_Q9P121_NTM_8428_102	2234	0.849	0.8	0.901	9.84E-05	7.18	1077	0.842	0.755	0.940	2.676	0.0021
SOMA_Q9Y2Q3_GSTK1_13474_40	2234	0.852	0.803	0.903	1.01E-04	7.167	1077	0.805	0.729	0.890	4.647	<0.0001
SOMA_P56937_HSD17B7_8272_22	2234	0.847	0.797	0.9	1.04E-04	7.156	1077	1.046	0.981	1.116	0.767	0.1711
SOMA_P35557_GCK_12960_9	2234	0.847	0.797	0.9	1.05E-04	7.152	1077	0.463	0.370	0.580	10.664	<0.0001
SOMA_Q9Y6J6_KCNE2_10427_2	2234	1.174	1.107	1.244	1.05E-04	7.152	NA	NA	NA	NA	NA	NA
SOMD P01374 LTA LTB 35056 PASS	2234	0.85	0.802	0.902	1.05E-04	7.152	NA	NA	NA	NA	NA	NA
SOMD Q06643 LTA LTB 35056 PASS	2234	0.85	0.802	0.902	1.05E-04	7.152	NA	NA	NA	NA	NA	NA
SOMA_P10092_CALCB_17170_15	2234	1.176	1.108	1.247	1.10E-04	7.132	1077	1.092	1.038	1.149	3.178	0.0007
SOMA_Q9H0N5_PCBD2_6899_37	2234	0.852	0.803	0.903	1.12E-04	7.125	1077	0.791	0.675	0.928	2.412	0.0039
SOMA_P59861_DEFB131_6934_8	2234	1.172	1.106	1.242	1.15E-04	7.111	NA	NA	NA	NA	NA	NA
SOMA_O15247_CLIC2_17837_5	2234	1.178	1.11	1.251	1.16E-04	7.108	1077	1.171	1.099	1.248	5.913	<0.0001
SOMA_Q9NS85_CA10_13666_222	2234	0.854	0.806	0.904	1.17E-04	7.106	1077	0.828	0.694	0.988	1.444	0.036
SOMA_Q8NFT8_DNER_9769_48	2234	0.854	0.806	0.904	1.18E-04	7.102	1077	1.022	0.939	1.113	0.215	0.6098
SOMA_P26441_CNTF_3489_9	2234	0.887	0.849	0.927	1.19E-04	7.098	1077	0.802	0.629	1.022	1.131	0.074
SOMA_Q99519_NEU1_15426_5	2234	0.854	0.806	0.905	1.19E-04	7.096	1077	0.948	0.813	1.105	0.306	0.4944
SOMA_Q9GZT5_WNT10A_10044_12	2234	0.852	0.804	0.903	1.22E-04	7.087	1077	0.807	0.717	0.909	3.390	0.0004
SOMA_Q9H2X0_CHRD_13438_115	2234	1.166	1.102	1.233	1.24E-04	7.08	1077	1.087	1.007	1.174	1.496	0.0319
SOMA_P51451_BLK_11338_49	2234	0.849	0.8	0.902	1.25E-04	7.077	1077	0.960	0.839	1.098	0.260	0.5499
SOMA_O43897_TLL1_6383_90	2234	0.852	0.804	0.904	1.26E-04	7.071	1077	0.876	0.790	0.973	1.885	0.013

SOMA_P51911_CNN1_15566_10	2234	1.169	1.104	1.238	1.27E-04	7.069	1077	0.958	0.863	1.064	0.371	0.4259
SOMA_Q9BWV2_SPATA9_7809_22	2234	1.162	1.1	1.228	1.27E-04	7.068	1077	1.045	0.961	1.137	0.523	0.3002
SOMA_Q9ULB1_NRXN1_8971_9	2234	0.853	0.805	0.904	1.33E-04	7.049	1077	0.815	0.723	0.918	3.108	0.0008
SOMA_Q8N6C8_LILRA3_6391_52	2234	1.166	1.102	1.234	1.35E-04	7.042	1077	1.306	1.202	1.419	9.559	<0.0001
SOMA_P10966_CD8B_9310_2	2234	0.856	0.809	0.906	1.35E-04	7.041	1077	0.856	0.700	1.047	0.885	0.1304
SOMA_P15153_RAC2_18950_13	2234	0.854	0.806	0.905	1.36E-04	7.04	1077	0.804	0.733	0.881	5.503	<0.0001
SOMA_O95157_NXPH3_6054_6	2234	0.855	0.807	0.906	1.41E-04	7.024	1077	0.834	0.724	0.961	1.921	0.012
SOMA_P43080_GUCA1A_10008_43	2234	0.852	0.804	0.904	1.42E-04	7.019	1077	1.012	0.919	1.114	0.088	0.8158
SOMA_P02775_PPBP_16765_52	2234	0.858	0.811	0.908	1.44E-04	7.015	1077	0.700	0.629	0.780	10.085	<0.0001
SOMA_Q9UEU0_VTI1B_8963_8	2234	1.161	1.099	1.227	1.47E-04	7.006	1077	0.862	0.769	0.967	1.950	0.0112
SOMA_Q13884_SNTB1_9078_207	2234	0.856	0.809	0.907	1.47E-04	7.005	1077	0.694	0.628	0.767	12.174	<0.0001
SOMA_Q9NX62_IMPAD1_9231_23	2234	0.855	0.808	0.906	1.48E-04	7.003	NA	NA	NA	NA	NA	NA
SOMA_Q9NZ72_STMN3_8019_73	2234	1.168	1.103	1.237	1.55E-04	6.983	1077	1.130	1.042	1.225	2.509	0.0031
SOMA_P49862_KLK7_3378_49	2234	0.848	0.798	0.901	1.55E-04	6.981	1077	0.456	0.343	0.608	7.115	<0.0001
SOMA_Q5T653_MRPL2_19132_1	2234	0.849	0.799	0.902	1.56E-04	6.981	1077	0.963	0.856	1.083	0.278	0.5274
SOMA_P40225_THPO_8059_1	2234	0.857	0.809	0.907	1.58E-04	6.975	1077	0.826	0.536	1.273	0.412	0.3874
SOMA_Q6UX41_BTNL8_9026_40	2234	1.16	1.098	1.226	1.58E-04	6.975	1077	0.982	0.877	1.099	0.127	0.7472
SOMA_Q9UI46_DNAI1_11396_39	2234	0.852	0.803	0.904	1.63E-04	6.962	1077	0.673	0.483	0.936	1.726	0.0188
SOMA_P58062_SPINK7_10974_20	2234	1.175	1.107	1.246	1.63E-04	6.96	1077	1.201	1.131	1.274	8.762	<0.0001

SOMA_Q86YW5_TREML1_11147_17	2234	0.853	0.805	0.905	1.64E-04	6.958	1077	0.766	0.634	0.926	2.237	0.0058
SOMD P19875 CXCL3 C 314849 FLAG	2234	0.856	0.809	0.907	1.68E-04	6.948	NA	NA	NA	NA	NA	NA
SOMD P19876 CXCL3 C 314849 FLAG	2234	0.856	0.809	0.907	1.68E-04	6.948	NA	NA	NA	NA	NA	NA
SOMA_P01275_GCG_4891_50	2234	1.17	1.104	1.24	1.69E-04	6.945	1077	1.219	1.120	1.327	5.329	<0.0001
SOMA_O15041_SEMA3E_5363_51	2234	1.17	1.104	1.24	1.74E-04	6.932	1077	1.038	0.982	1.096	0.734	0.1844
SOMA_Q9BUR5_APOO_9373_405	2234	0.89	0.852	0.929	1.75E-04	6.931	NA	NA	NA	NA	NA	NA
SOMA_Q9P2E9_RRBP1_9913_4	2234	1.17	1.104	1.24	1.75E-04	6.93	1077	0.985	0.878	1.105	0.099	0.7954
SOMA_Q9H773_DCTPP1_4314_12	2234	1.179	1.109	1.253	1.79E-04	6.921	1077	1.426	1.318	1.543	17.944	<0.0001
SOMA_Q86SJ2_AMIGO2_14134_49	2234	1.176	1.107	1.249	1.86E-04	6.904	1077	1.062	1.009	1.117	1.683	0.0208
SOMA_P18859_ATP5J_7788_1	2234	1.169	1.104	1.239	1.86E-04	6.903	NA	NA	NA	NA	NA	NA
SOMA_Q14145_KEAP1_12568_14	2234	0.853	0.804	0.905	1.93E-04	6.888	1077	0.912	0.785	1.060	0.638	0.2303
SOMA_P01242_GH2_10978_39	2234	1.164	1.1	1.232	2.00E-04	6.871	1077	1.075	1.029	1.123	2.889	0.0013
SOMA_P50895_BCAM_2816_50	2234	1.165	1.101	1.233	2.22E-04	6.827	1077	1.048	0.993	1.106	1.070	0.0852
SOMA_O60568_PLOD3_10612_18	2234	1.175	1.106	1.248	2.24E-04	6.822	1077	1.168	1.065	1.280	3.001	0.001
SOMA_P55285_CDH6_16312_45	2234	0.856	0.808	0.907	2.29E-04	6.814	1077	1.022	0.896	1.165	0.126	0.7483
SOMA_P40197_GP5_7185_29	2234	0.854	0.805	0.906	2.29E-04	6.812	1077	0.709	0.639	0.787	10.006	<0.0001
SOMA_P39059_COL15A1_8974_172	2234	1.17	1.103	1.241	2.30E-04	6.81	1077	1.185	1.094	1.283	4.542	<0.0001
SOMA_P99999_CYCS_2942_50	2234	0.866	0.821	0.914	2.31E-04	6.81	1077	0.747	0.609	0.916	2.294	0.0051
SOMA_Q5VWZ2_LYPLAL1_12428_2	2234	0.849	0.799	0.903	2.35E-04	6.802	1077	0.898	0.757	1.066	0.658	0.2199

SOMA_Q99490_AGAP2_14291_53	2234	0.858	0.81	0.909	2.35E-04	6.802	1077	0.522	0.417	0.653	7.924	<0.0001
SOMA_Q9H0H5_RACGAP1_13587_10	2234	1.177	1.108	1.251	2.36E-04	6.8	1077	1.312	1.211	1.421	10.613	<0.0001
SOMA_O00515_LAD1_6407_63	2234	0.858	0.81	0.909	2.36E-04	6.799	1077	1.000	0.900	1.111	0.002	0.995
SOMA_Q96A83_COL26A1_9539_25	2234	0.851	0.801	0.904	2.38E-04	6.796	NA	NA	NA	NA	NA	NA
SOMA_Q9NUQ9_FAM49B_19176_27	2234	1.166	1.101	1.234	2.43E-04	6.788	NA	NA	NA	NA	NA	NA
SOMA_P32455_GBP1_15326_64	2234	1.17	1.103	1.241	2.46E-04	6.783	1077	1.142	1.059	1.232	3.256	0.0006
SOMA_Q9P126_CLEC1B_4332_6	2234	0.856	0.807	0.907	2.47E-04	6.78	1077	0.873	0.789	0.966	2.073	0.0084
SOMD P63098 PPP3CA 490372 FLAG	2234	0.855	0.806	0.906	2.48E-04	6.778	NA	NA	NA	NA	NA	NA
SOMD Q08209 PPP3CA 490372 FLAG	2234	0.855	0.806	0.906	2.48E-04	6.778	NA	NA	NA	NA	NA	NA
SOMA_Q13261_IL15RA_3445_53	2234	1.164	1.099	1.232	2.53E-04	6.77	1077	1.063	0.982	1.151	0.884	0.1306
SOMA_Q8TE54_SLC26A7_13979_3	2234	1.161	1.098	1.228	2.56E-04	6.764	1077	1.128	1.059	1.202	3.701	0.0002
SOMA_Q9UP52_TFR2_11537_12	2234	0.854	0.805	0.906	2.63E-04	6.753	1077	0.933	0.734	1.186	0.243	0.5714
SOMA_P55000_SLURP1_6401_73	2234	1.168	1.102	1.238	2.64E-04	6.751	NA	NA	NA	NA	NA	NA
SOMA_P09681_GIP_16292_288	2234	1.164	1.099	1.232	2.66E-04	6.748	1077	1.172	1.114	1.234	8.966	<0.0001
SOMA_Q13361_MFAP5_6440_31	2234	0.855	0.806	0.907	2.69E-04	6.742	1077	0.752	0.496	1.141	0.745	0.1799
SOMA_Q15126_PMVK_12450_42	2234	0.856	0.808	0.908	2.70E-04	6.742	1077	0.855	0.760	0.963	2.015	0.0097
SOMA_P19021_PAM_5620_13	2234	1.147	1.089	1.207	2.76E-04	6.731	1077	1.358	1.245	1.482	11.276	<0.0001
SOMA_Q08116_RGS1_18945_11	2234	1.167	1.101	1.237	2.76E-04	6.731	1077	1.080	1.003	1.163	1.379	0.0417
SOMA_Q9H492_MAP1LC3_17781_191	2234	1.172	1.104	1.244	2.83E-04	6.721	1077	1.196	1.086	1.317	3.560	0.0003

SOMA_P31943_HNRNPH1_14309_8	2234	0.862	0.815	0.911	2.84E-04	6.72	1077	0.988	0.892	1.095	0.086	0.8206
SOMA_P41235_HNF4A_10041_3	2234	0.854	0.805	0.906	2.84E-04	6.719	1077	0.801	0.716	0.896	3.968	0.0001
SOMA_Q9UM21_MGAT4A_9392_43	2234	0.87	0.826	0.917	2.85E-04	6.717	1077	0.893	0.675	1.182	0.368	0.4284
SOMA_Q8N128_FAM177A_8039_41	2234	0.854	0.805	0.906	2.87E-04	6.714	1077	0.759	0.677	0.852	5.519	<0.0001
SOMA_Q14554_PDIA5_5593_11	2234	0.857	0.808	0.908	2.98E-04	6.699	1077	0.785	0.711	0.867	5.758	<0.0001
SOMA_Q13018_PLA2R1_10916_44	2234	1.168	1.101	1.238	3.00E-04	6.696	1077	1.475	1.347	1.614	16.498	<0.0001
SOMA_O60504_SORBS3_12976_49	2234	1.167	1.101	1.237	3.08E-04	6.685	1077	0.942	0.741	1.196	0.205	0.6237
SOMA_Q9NZU0_FLRT3_13123_3	2234	1.169	1.102	1.24	3.08E-04	6.685	1077	1.134	1.089	1.180	9.062	<0.0001
SOMA_Q96NZ8_WFIKKN1_3191_50	2234	0.863	0.816	0.912	3.17E-04	6.672	1077	0.907	0.765	1.077	0.576	0.2656
SOMA_P13716_ALAD_15523_9	2234	0.855	0.806	0.907	3.21E-04	6.666	1077	0.777	0.692	0.872	4.735	<0.0001
SOMA_Q7Z408_CSMD2_9971_5	2234	1.16	1.097	1.227	3.25E-04	6.66	1077	1.040	0.946	1.143	0.380	0.4166
SOMA_P05230_FGF1_3486_58	2234	0.886	0.847	0.928	3.36E-04	6.646	1077	0.937	0.791	1.111	0.341	0.4565
SOMA_Q9H4G4_GLIPR2_15522_2	2234	0.857	0.808	0.908	3.40E-04	6.641	1077	0.832	0.752	0.920	3.464	0.0003
SOMA_Q6ZMC9_SIGLEC1_10847_1	2234	1.167	1.1	1.237	3.43E-04	6.638	1077	1.059	0.982	1.142	0.868	0.1356
SOMA_Q6ZVN8_HFE2_3332_57	2234	0.854	0.805	0.907	3.43E-04	6.638	NA	NA	NA	NA	NA	NA
SOMA_P01127_PDGFB_4149_8	2234	0.86	0.812	0.91	3.46E-04	6.634	1077	0.748	0.675	0.828	7.564	<0.0001
SOMA_Q9NYJ7_DLL3_9974_8	2234	0.859	0.811	0.91	3.53E-04	6.624	1077	0.291	0.184	0.463	6.765	<0.0001
SOMA_Q9UBG0_MRC2_3041_55	2234	1.166	1.1	1.237	3.71E-04	6.604	1077	1.326	1.213	1.449	9.258	<0.0001
SOMA_Q8N695_SLC5A8_13691_10	2234	0.858	0.81	0.91	3.79E-04	6.594	1077	0.876	0.787	0.975	1.819	0.0152

SOMA_P55735_SEC13_14689_3	2234	0.855	0.805	0.907	3.87E-04	6.585	1077	0.738	0.657	0.829	6.489	<0.0001
SOMA_P10720_PF4V1_5663_18	2234	0.856	0.806	0.908	3.97E-04	6.574	1077	1.060	0.989	1.136	0.998	0.1004
SOMA_O95156_NXPH2_9511_61	2234	0.856	0.806	0.908	3.98E-04	6.573	1077	0.992	0.889	1.105	0.056	0.8786
SOMA_Q96AT9_RPE_12646_2	2234	0.858	0.809	0.909	3.98E-04	6.573	1077	0.851	0.763	0.950	2.407	0.0039
SOMA_Q5T2T1_MPP7_12732_13	2234	0.872	0.828	0.919	4.13E-04	6.557	1077	0.836	0.669	1.043	0.951	0.112
SOMA_Q08AG7_MZT1_8072_19	2234	0.854	0.804	0.907	4.16E-04	6.553	NA	NA	NA	NA	NA	NA
SOMA_P35754_GLRX_18386_36	2234	1.158	1.095	1.225	4.23E-04	6.547	1077	0.937	0.748	1.172	0.247	0.5669
SOMA_P12034_FGF5_3065_65	2234	1.159	1.096	1.227	4.32E-04	6.537	1077	0.986	0.885	1.099	0.096	0.802
SOMA_P78504_JAG1_5092_51	2234	1.167	1.1	1.238	4.33E-04	6.536	1077	1.320	1.242	1.404	18.387	<0.0001
SOMA_P54760_EPHB4_15530_33	2234	1.168	1.1	1.239	4.38E-04	6.531	1077	1.321	1.235	1.412	15.452	<0.0001
SOMA_Q9UJW8_ZNF180_12771_19	2234	0.857	0.808	0.909	4.41E-04	6.528	1077	0.924	0.799	1.069	0.539	0.2889
SOMA_Q13753_LAMC2_9580_5	2234	1.163	1.098	1.232	4.46E-04	6.524	1077	1.102	1.054	1.153	4.740	<0.0001
SOMA_O43866_CD5L_3293_2	2234	1.164	1.098	1.234	4.56E-04	6.514	1077	1.107	1.013	1.210	1.599	0.0252
SOMA_P09960_LTA4H_3204_2	2234	1.172	1.103	1.245	4.69E-04	6.502	1077	1.221	1.146	1.301	9.215	<0.0001
SOMA_Q99706_KIR2DL4_5095_21	2234	1.161	1.096	1.229	4.79E-04	6.492	1077	1.086	1.035	1.139	3.155	0.0007
SOMA_A2RUU4_CLPSL1_9526_3	2234	1.168	1.1	1.24	4.88E-04	6.484	1077	1.058	1.004	1.114	1.466	0.0342
SOMA_P48426_PIP4K2A_12697_30	2234	0.858	0.809	0.91	4.97E-04	6.477	1077	0.762	0.689	0.843	6.895	<0.0001
SOMA_Q07325_CXCL9_9188_119	2234	1.161	1.097	1.23	5.05E-04	6.469	1077	1.018	0.938	1.106	0.176	0.6673
SOMA_P07225_PROS1_2700_56	2234	0.862	0.814	0.913	5.08E-04	6.467	1077	0.907	0.808	1.018	1.017	0.0962

SOMA_P26640_VARS_13083_18	2234	0.86	0.811	0.911	5.14E-04	6.462	NA	NA	NA	NA	NA	NA
SOMA_P52272_HNRNPM_12783_29	2234	1.16	1.095	1.228	5.22E-04	6.455	1077	1.013	0.922	1.112	0.100	0.7941
SOMA_P84157_MXRA7_8005_1	2234	1.169	1.101	1.241	5.22E-04	6.455	1077	1.170	1.107	1.236	7.559	<0.0001
SOMA_Q9UK80_USP21_12681_63	2234	0.867	0.821	0.916	5.23E-04	6.454	1077	0.578	0.404	0.827	2.575	0.0027
SOMA_Q15797_SMAD1_9838_4	2234	0.858	0.809	0.91	5.24E-04	6.453	1077	0.716	0.643	0.798	8.932	<0.0001
SOMA_Q8NEQ5_C1orf16_6896_3	2234	0.86	0.811	0.911	5.26E-04	6.452	1077	0.656	0.569	0.757	8.177	<0.0001
SOMA_Q9Y680_FKBP7_9288_7	2234	1.161	1.096	1.229	5.29E-04	6.45	1077	0.968	0.848	1.105	0.202	0.6286
SOMA_Q96PJ5_FCRL4_8973_23	2234	0.862	0.815	0.913	5.33E-04	6.446	1077	0.864	0.607	1.231	0.378	0.4186
SOMA_Q9HCU0_CD248_15491_20	2234	1.164	1.098	1.234	5.41E-04	6.439	1077	1.347	1.240	1.462	11.862	<0.0001
SOMA_P52848_NDST1_6927_7	2234	0.859	0.81	0.911	5.47E-04	6.435	1077	0.808	0.724	0.901	3.911	0.0001
SOMA_P25686_DNAJB2_11438_6	2234	1.162	1.097	1.232	5.51E-04	6.432	1077	0.866	0.779	0.964	2.068	0.0085
SOMA_P07492_GRP_5897_58	2234	0.858	0.809	0.91	5.77E-04	6.412	1077	1.052	0.984	1.125	0.860	0.138
SOMA_P41208_CETN2_13078_3	2234	0.858	0.808	0.91	5.86E-04	6.405	1077	0.747	0.644	0.867	3.890	0.0001
SOMA_P21741_MDK_2911_27	2234	1.173	1.103	1.248	5.91E-04	6.401	1077	1.247	1.165	1.335	9.652	<0.0001
SOMA_Q9NS98_SEMA3G_5628_21	2234	0.864	0.817	0.914	6.05E-04	6.391	1077	0.818	0.735	0.910	3.656	0.0002
SOMA_P04114_APOB_2797_56	2234	0.859	0.81	0.911	6.15E-04	6.384	1077	1.000	0.903	1.108	0.000	0.9996
SOMA_Q14956_GPNMB_8289_8	2234	1.165	1.098	1.235	6.21E-04	6.38	1077	1.136	1.063	1.213	3.776	0.0002
SOMA_Q9ULZ9_MMP17_2838_53	2234	1.161	1.096	1.23	6.31E-04	6.373	1077	1.160	1.073	1.253	3.763	0.0002
SOMA_Q6S5H5_POTEG_7960_53	2234	0.859	0.81	0.911	6.35E-04	6.37	1077	1.017	0.938	1.103	0.170	0.6766

SOMA_P23141_CES1_15487_164	2234	0.861	0.812	0.912	6.45E-04	6.363	1077	0.775	0.679	0.883	3.858	0.0001
SOMA_P55287_CDH11_7763_25	2234	0.863	0.815	0.914	6.63E-04	6.351	1077	0.688	0.396	1.196	0.734	0.1847
SOMA_P31949_S100A11_14011_17	2234	1.166	1.098	1.238	6.92E-04	6.333	1077	0.918	0.737	1.142	0.356	0.4409
SOMA_P49796_RGS3_12827_37	2234	0.855	0.804	0.909	7.06E-04	6.324	1077	1.069	0.984	1.161	0.950	0.1122
SOMA_Q9HAW9_UGT1A8_8899_75	2234	0.859	0.809	0.911	7.07E-04	6.324	1077	0.986	0.885	1.099	0.095	0.8028
SOMA_Q14376_GALE_11457_53	2234	0.858	0.808	0.911	7.10E-04	6.321	1077	0.688	0.592	0.798	6.064	<0.0001
SOMA_P11908_PRPS2_18329_4	2234	0.861	0.812	0.913	7.31E-04	6.309	1077	0.798	0.679	0.938	2.203	0.0063
SOMA_A6H8Y1_BDP1_8929_7	2234	0.862	0.813	0.913	7.34E-04	6.307	1077	0.880	0.765	1.011	1.144	0.0717
SOMA_Q9C098_DCLK3_7826_1	2234	0.856	0.805	0.909	7.45E-04	6.301	1077	1.035	0.961	1.114	0.436	0.3666
SOMA_Q9H2E6_SEMA6A_7945_10	2234	1.17	1.1	1.244	7.76E-04	6.283	1077	1.400	1.287	1.523	14.403	<0.0001
SOMA_Q7Z553_MDGA2_19372_7	2234	0.859	0.809	0.911	7.80E-04	6.281	1077	0.903	0.702	1.162	0.368	0.4282
SOMA_P13667_PDIA4_7146_16	2234	1.159	1.094	1.227	8.44E-04	6.246	1077	1.049	0.979	1.123	0.749	0.1783
SOMA_Q9H488_POFUT1_5634_39	2234	0.858	0.808	0.911	8.48E-04	6.244	1077	0.772	0.658	0.905	2.845	0.0014
SOMA_Q15303_ERBB4_2618_10	2234	0.894	0.855	0.934	8.55E-04	6.241	1077	0.954	0.832	1.094	0.300	0.5017
SOMA_Q13017_ARHGAP5_14748_31	2234	0.871	0.825	0.919	8.58E-04	6.239	1077	0.809	0.697	0.939	2.267	0.0054
SOMA_P29622_SERPINA_3449_58	2234	0.858	0.808	0.911	8.61E-04	6.238	1077	0.683	0.620	0.753	13.890	<0.0001
SOMA_Q7Z4V5_HDGFRP2_4553_65	2234	1.152	1.09	1.218	8.73E-04	6.232	NA	NA	NA	NA	NA	NA
SOMA_O95229_ZWINT_18286_3	2234	0.863	0.814	0.914	8.79E-04	6.229	1077	0.984	0.878	1.103	0.106	0.784
SOMA_O14683_TP53I11_13022_20	2234	0.859	0.809	0.912	8.80E-04	6.228	1077	0.704	0.607	0.816	5.479	<0.0001

SOMA_Q9H5V8_CDCP1_8589_13	2234	0.876	0.832	0.923	8.94E-04	6.222	1077	1.019	0.916	1.134	0.135	0.7322
SOMA_P12318_FCGR2A_3309_2	2234	1.165	1.097	1.237	9.13E-04	6.212	1077	1.217	1.108	1.337	4.383	<0.0001
SOMA_Q15291_RBBP5_13631_1	2234	1.158	1.093	1.226	9.24E-04	6.207	1077	1.043	0.954	1.139	0.451	0.3543
SOMA_O15232_MATN3_19361_78	2234	1.167	1.098	1.241	9.45E-04	6.197	1077	1.267	1.198	1.339	16.133	<0.0001
SOMA_O00214_LGALS8_4909_68	2234	0.859	0.809	0.912	9.67E-04	6.187	1077	1.039	0.947	1.140	0.384	0.4129
SOMA_Q99726_SLC30A3_9081_39	2234	0.863	0.815	0.915	9.75E-04	6.184	1077	0.872	0.740	1.027	0.997	0.1008
SOMA_P07306_ASGR1_5452_71	2234	1.167	1.098	1.24	9.83E-04	6.18	1077	1.405	1.319	1.497	25.360	<0.0001
SOMA_P41252_IARS_12815_9	2234	0.855	0.804	0.909	9.88E-04	6.178	NA	NA	NA	NA	NA	NA
SOMA_Q13586_STIM1_8916_32	2234	0.864	0.815	0.915	1.03E-03	6.158	1077	0.861	0.772	0.960	2.167	0.0068
SOMA_O60676_CST8_10572_65	2234	1.16	1.094	1.23	1.09E-03	6.136	1077	1.311	1.224	1.404	14.082	<0.0001
SOMA_P53999_SUB1_19236_24	2234	1.154	1.09	1.221	1.09E-03	6.136	1077	1.057	0.971	1.150	0.699	0.2
SOMA_Q13247_SRSF6_11573_3	2234	1.159	1.093	1.228	1.10E-03	6.129	1077	1.146	1.084	1.211	5.801	<0.0001
SOMA_Q8NDI1_EHBP1_12813_18	2234	0.866	0.818	0.917	1.11E-03	6.129	1077	0.906	0.769	1.067	0.624	0.2377
SOMA_Q13588_GRAP_12820_1	2234	0.864	0.815	0.915	1.11E-03	6.126	1077	0.854	0.755	0.966	1.913	0.0122
SOMA_P02776_PF4_2697_7	2234	0.864	0.815	0.916	1.14E-03	6.114	1077	0.714	0.634	0.804	7.559	<0.0001
SOMA_P55773_CCL23_3028_36	2234	1.163	1.095	1.234	1.15E-03	6.113	1077	1.299	1.204	1.401	10.824	<0.0001
SOMA_Q8TEY5_CREB3L4_11308_8	2234	1.16	1.094	1.231	1.16E-03	6.11	1077	1.105	1.035	1.179	2.572	0.0027
SOMA_Q5DID0_UMODL1_9114_84	2234	0.861	0.811	0.913	1.16E-03	6.108	1077	0.989	0.890	1.100	0.074	0.8427
SOMA_Q96IW7_SEC22A_13713_164	2234	0.86	0.81	0.913	1.17E-03	6.105	1077	0.920	0.806	1.049	0.670	0.2138

SOMA_P28330_ACADL_17857_6	2234	0.866	0.818	0.917	1.19E-03	6.096	1077	1.070	0.985	1.163	0.965	0.1083
SOMA_P53990_IST1_12434_25	2234	0.863	0.814	0.915	1.21E-03	6.09	1077	0.757	0.685	0.837	7.335	<0.0001
SOMA_Q00887_PSG9_9335_28	2234	1.16	1.093	1.23	1.23E-03	6.083	1077	1.049	0.989	1.113	0.961	0.1094
SOMA_P12931_SRC_15433_4	2234	0.866	0.818	0.917	1.27E-03	6.069	1077	0.727	0.654	0.809	8.397	<0.0001
SOMA_Q8WUE5_CT55_9363_11	2234	0.893	0.853	0.934	1.28E-03	6.066	1077	1.001	0.899	1.113	0.004	0.991
SOMA_Q12846_STX4_10668_5	2234	1.153	1.09	1.221	1.29E-03	6.062	1077	0.878	0.789	0.976	1.785	0.0164
SOMA_P30990_NTS_7857_22	2234	1.154	1.09	1.222	1.29E-03	6.061	1077	1.092	1.015	1.176	1.719	0.0191
SOMA_Q49AH0_CDNF_4962_52	2234	1.158	1.092	1.228	1.32E-03	6.053	1077	1.109	1.054	1.168	4.134	<0.0001
SOMA_Q9BQB4_SOST_13101_60	2234	1.157	1.092	1.227	1.33E-03	6.05	1077	1.092	1.010	1.180	1.565	0.0272
SOMA_P15918_RAG1_11311_79	2234	0.86	0.81	0.913	1.34E-03	6.044	1077	1.008	0.911	1.114	0.055	0.8816
SOMA_P04629_NTRK1_3477_63	2234	1.153	1.09	1.221	1.35E-03	6.041	1077	1.038	0.957	1.126	0.437	0.3654
SOMA_P13674_P4HA1_11645_9	2234	1.152	1.089	1.219	1.35E-03	6.041	1077	1.088	1.018	1.164	1.866	0.0136
SOMA_Q6UWR7_ENPP6_15579_26	2234	0.864	0.814	0.916	1.38E-03	6.033	1077	1.005	0.914	1.105	0.034	0.9249
SOMA_P78356_PIP4K2B_18232_42	2234	0.863	0.813	0.915	1.41E-03	6.025	1077	0.745	0.676	0.821	8.578	<0.0001
SOMA_Q16740_CLPP_17196_5	2234	1.151	1.088	1.217	1.42E-03	6.021	1077	0.975	0.855	1.111	0.154	0.7017
SOMA_Q8NBP7_PCSK9_5231_79	2234	0.864	0.814	0.916	1.43E-03	6.017	1077	0.883	0.794	0.983	1.643	0.0228
SOMA_P30711_GSTT1_19230_12	2234	1.158	1.092	1.228	1.44E-03	6.015	1077	1.018	0.939	1.104	0.176	0.6664
SOMA_P15291_B4GALT1_13381_49	2234	1.164	1.095	1.237	1.48E-03	6.002	1077	1.424	1.328	1.528	22.349	<0.0001
SOMA_P38646_HSPA9_13492_44	2234	1.158	1.092	1.228	1.48E-03	6.001	1077	1.177	1.097	1.263	5.204	<0.0001

SOMA_Q92519_TRIB2_12363_70	2234	0.895	0.856	0.936	1.49E-03	5.998	1077	0.955	0.824	1.107	0.265	0.5428
SOMA_Q5GAN6_RNASE10_5602_62	2234	1.155	1.09	1.224	1.54E-03	5.985	1077	1.081	1.025	1.140	2.360	0.0044
SOMA_P21333_FLNA_11171_25	2234	1.147	1.085	1.211	1.55E-03	5.982	1077	0.844	0.718	0.992	1.400	0.0398
SOMA_Q8IZF2_ADGRF5_6409_57	2234	0.864	0.815	0.916	1.57E-03	5.975	1077	0.669	0.496	0.902	2.076	0.0084
SOMA_P08833_IGFBP1_2771_35	2234	1.153	1.089	1.221	1.58E-03	5.973	1077	1.175	1.102	1.252	6.094	<0.0001
SOMA_Q5T4F4_ZFYVE27_9102_28	2234	0.861	0.81	0.914	1.59E-03	5.972	1077	1.083	0.996	1.177	1.207	0.0621
SOMA_P60903_S100A10_15318_75	2234	0.863	0.813	0.916	1.59E-03	5.97	1077	0.899	0.770	1.050	0.745	0.1798
SOMA_O76096_CST7_3302_58	2234	1.153	1.089	1.221	1.61E-03	5.966	1077	0.962	0.857	1.080	0.287	0.5164
SOMA_Q9UM44_HHLA2_14132_21	2234	0.862	0.812	0.915	1.62E-03	5.962	1077	1.030	0.962	1.103	0.406	0.3923
SOMA_P12931_SRC_5488_74	2234	0.869	0.821	0.919	1.63E-03	5.961	1077	0.733	0.668	0.804	10.369	<0.0001
SOMA_Q96IY4_CPB2_3518_54	2234	0.866	0.817	0.917	1.65E-03	5.954	1077	0.830	0.748	0.921	3.354	0.0004
SOMA_Q9HBX9_RXFP1_14135_3	2234	0.865	0.816	0.917	1.66E-03	5.953	1077	0.992	0.893	1.101	0.058	0.874
SOMA_Q99584_S100A13_7223_60	2234	1.164	1.095	1.237	1.66E-03	5.952	1077	1.376	1.291	1.467	21.698	<0.0001
SOMA_Q9UK76_HN1_19335_2	2234	0.862	0.813	0.915	1.67E-03	5.951	NA	NA	NA	NA	NA	NA
SOMA_P04085_PDGFA_4499_21	2234	0.867	0.819	0.918	1.67E-03	5.949	1077	0.756	0.680	0.841	6.647	<0.0001
SOMA_P00387_CYB5R3_7215_18	2234	0.867	0.818	0.918	1.68E-03	5.948	1077	0.784	0.468	1.312	0.451	0.3539
SOMA_Q9ULZ1_APLN_6622_90	2234	0.86	0.81	0.914	1.68E-03	5.948	1077	0.929	0.787	1.098	0.412	0.3874
SOMA_Q96IZ0_PAWR_9565_6	2234	0.866	0.817	0.918	1.71E-03	5.939	1077	0.898	0.804	1.004	1.236	0.058
SOMA_Q9GZP0_PDGFD_17140_57	2234	0.866	0.817	0.918	1.72E-03	5.936	1077	0.809	0.724	0.905	3.697	0.0002

SOMA_Q86VR8_FJX1_7921_65	2234	1.158	1.091	1.228	1.73E-03	5.934	1077	1.255	1.163	1.355	8.198	<0.0001
SOMA_Q96MU8_KREMEN1_17331_138	2234	1.157	1.091	1.227	1.75E-03	5.93	1077	1.379	1.295	1.470	22.634	<0.0001
SOMA_Q8NC24_RELL2_6600_70	2234	0.862	0.812	0.915	1.75E-03	5.929	1077	0.978	0.866	1.105	0.142	0.7209
SOMA_P12314_FCGR1A_3312_64	2234	1.15	1.087	1.216	1.80E-03	5.916	1077	1.003	0.915	1.099	0.021	0.9532
SOMA_P05161_ISG15_14151_4	2234	1.153	1.088	1.221	1.81E-03	5.915	1077	1.028	0.938	1.126	0.252	0.5591
SOMA_Q9UBL0_ARPP21_12860_7	2234	0.863	0.813	0.916	1.85E-03	5.904	1077	1.025	0.941	1.117	0.243	0.5713
SOMA_Q9Y4P9_SPEF1_12876_39	2234	0.863	0.812	0.916	1.90E-03	5.894	1077	0.958	0.824	1.114	0.236	0.5803
SOMA_P08590_MYL3_18376_19	2234	1.157	1.091	1.228	1.92E-03	5.889	1077	1.185	1.118	1.257	7.838	<0.0001
SOMA_Q9H9Q2_COPS7B_12384_92	2234	0.866	0.817	0.918	1.92E-03	5.888	1077	0.814	0.717	0.923	2.856	0.0014
SOMA_P01024_C3_5803_24	2234	1.157	1.091	1.227	1.93E-03	5.887	1077	1.208	1.150	1.269	13.360	<0.0001
SOMA_P14138_EDN3_15383_200	2234	0.865	0.815	0.917	1.95E-03	5.882	1077	0.723	0.464	1.125	0.824	0.15
SOMA_P39656_DDOST_17161_1	2234	1.16	1.092	1.231	1.97E-03	5.878	1077	1.053	0.954	1.164	0.516	0.305
SOMA_Q92859_NEO1_8900_28	2234	1.156	1.09	1.226	2.00E-03	5.872	1077	1.365	1.258	1.481	13.144	<0.0001
SOMA_O95429_BAG4_12844_10	2234	1.15	1.087	1.217	2.01E-03	5.869	1077	1.010	0.917	1.112	0.072	0.8473
SOMA_Q9UBL9_P2RX2_9493_56	2234	0.863	0.813	0.916	2.01E-03	5.869	NA	NA	NA	NA	NA	NA
SOMA_Q16401_PSMD5_10716_35	2234	0.889	0.847	0.932	2.02E-03	5.868	1077	0.899	0.762	1.060	0.689	0.2048
SOMA_A6NI73_LILRA5_7787_25	2234	1.155	1.089	1.225	2.03E-03	5.866	1077	1.267	1.169	1.373	8.105	<0.0001
SOMA_P51665_PSMD7_3898_5	2234	1.15	1.087	1.217	2.03E-03	5.864	1077	1.019	0.945	1.099	0.207	0.6202
SOMA_Q9BRX8_FAM213A_13423_94	2234	0.862	0.811	0.916	2.05E-03	5.861	NA	NA	NA	NA	NA	NA

SOMA_Q96E11_MRRF_12355_223	2234	0.874	0.827	0.923	2.08E-03	5.854	1077	0.844	0.672	1.059	0.848	0.142
SOMA_Q86Y29_BAGE3_6442_6	2234	0.866	0.817	0.918	2.19E-03	5.831	1077	0.723	0.649	0.805	8.463	<0.0001
SOMA_O00755_WNT7A_4889_82	2234	0.863	0.813	0.916	2.26E-03	5.818	1077	0.998	0.901	1.104	0.015	0.9652
SOMA_Q9H3U1_UNC45A_13082_9	2234	0.869	0.82	0.92	2.26E-03	5.817	1077	0.797	0.722	0.880	5.166	<0.0001
SOMA_Q16549_PCSK7_4459_68	2234	0.866	0.816	0.918	2.28E-03	5.814	1077	0.861	0.771	0.962	2.100	0.0079
SOMA_Q92692_PVRL2_6245_4	2234	1.159	1.091	1.231	2.29E-03	5.813	NA	NA	NA	NA	NA	NA
SOMA_P05412_JUN_10356_21	2234	0.864	0.815	0.917	2.32E-03	5.808	1077	0.723	0.595	0.878	2.967	0.0011
SOMA_Q8N387_MUC15_6417_55	2234	1.156	1.09	1.226	2.32E-03	5.808	NA	NA	NA	NA	NA	NA
SOMA_Q9UHD9_UBQLN2_19121_3	2234	1.154	1.088	1.223	2.35E-03	5.802	1077	0.921	0.830	1.022	0.919	0.1205
SOMA_Q9NZ94_NLGN3_8941_4	2234	1.149	1.086	1.216	2.38E-03	5.797	1077	1.004	0.920	1.095	0.031	0.9319
SOMA_P54577_YARS_19270_26	2234	0.869	0.821	0.92	2.45E-03	5.783	NA	NA	NA	NA	NA	NA
SOMA_Q12860_CNTN1_2974_61	2234	0.866	0.817	0.919	2.45E-03	5.783	1077	0.851	0.764	0.948	2.479	0.0033
SOMA_Q9NRX6_TMEM167_13421_17	2234	0.864	0.814	0.917	2.50E-03	5.774	1077	1.021	0.935	1.115	0.193	0.6408
SOMA_Q8N6P7_IL22RA1_3620_67	2234	1.152	1.087	1.221	2.51E-03	5.773	1077	1.059	1.004	1.118	1.444	0.036
SOMA_Q16548_BCL2A1_3413_50	2234	0.866	0.817	0.919	2.53E-03	5.77	1077	0.812	0.701	0.940	2.279	0.0053
SOMD O75462 CRLF1 C 2607 54 PASS	2234	0.89	0.848	0.934	2.58E-03	5.76	NA	NA	NA	NA	NA	NA
SOMD Q9UBD9 CRLF1 C 2607 54 PASS	2234	0.89	0.848	0.934	2.58E-03	5.76	NA	NA	NA	NA	NA	NA
SOMA_Q9UHP3_USP25_9215_117	2234	0.862	0.812	0.916	2.59E-03	5.759	1077	0.944	0.807	1.105	0.325	0.4733
SOMA_O94933_SLITRK3_10565_19	2234	0.868	0.819	0.92	2.63E-03	5.752	1077	0.901	0.807	1.007	1.177	0.0665

SOMA_Q6ZMJ4_IL34_4556_10	2234	0.865	0.814	0.918	2.66E-03	5.748	1077	0.919	0.828	1.020	0.944	0.1138
SOMA_Q96A26_FAM162A_6531_29	2234	1.147	1.084	1.213	2.66E-03	5.748	1077	1.021	0.930	1.121	0.179	0.6626
SOMA_P35900_KRT20_12975_11	2234	1.156	1.089	1.227	2.69E-03	5.743	1077	1.033	0.954	1.118	0.377	0.42
SOMA_Q13613_MTMR1_11167_6	2234	0.864	0.814	0.918	2.74E-03	5.735	1077	1.086	1.014	1.164	1.746	0.0179
SOMA_P50591_TNFSF10_8833_20	2234	1.146	1.084	1.213	2.75E-03	5.733	1077	0.952	0.786	1.153	0.213	0.613
SOMA_P05186_ALPL_16926_44	2234	1.154	1.088	1.224	2.86E-03	5.716	1077	1.041	0.979	1.107	0.711	0.1946
SOMA_P49703_ARL4D_18413_24	2234	1.152	1.087	1.221	2.86E-03	5.716	1077	0.958	0.807	1.138	0.202	0.6287
SOMA_O43896_KIF1C_9899_28	2234	1.144	1.082	1.209	2.87E-03	5.714	1077	1.011	0.929	1.099	0.095	0.8042
SOMA_Q92988_DLX4_11910_27	2234	0.872	0.824	0.923	2.90E-03	5.71	1077	0.826	0.716	0.953	2.055	0.0088
SOMA_O75436_VPS26A_19293_6	2234	1.15	1.086	1.219	2.94E-03	5.704	1077	0.835	0.757	0.922	3.482	0.0003
SOMA_P04808_RLN1_6300_14	2234	1.158	1.09	1.23	2.99E-03	5.697	NA	NA	NA	NA	NA	NA
SOMA_Q96KQ7_EHMT2_5843_60	2234	0.867	0.817	0.92	2.99E-03	5.696	1077	0.894	0.775	1.032	0.897	0.1267
SOMA_Q5XLA6_CARD17_18267_74	2234	0.863	0.812	0.917	3.01E-03	5.694	1077	0.974	0.863	1.099	0.172	0.6723
SOMA_Q9H8J5_MANSC1_9557_5	2234	1.156	1.089	1.228	3.05E-03	5.688	1077	1.316	1.226	1.413	13.459	<0.0001
SOMA_O60609_GFRA3_2505_49	2234	1.15	1.085	1.218	3.09E-03	5.682	1077	1.028	0.962	1.098	0.382	0.4152
SOMA_Q14213_EBI3_10851_77	2234	1.156	1.089	1.227	3.13E-03	5.677	1077	1.194	1.131	1.260	9.896	<0.0001
SOMA_Q8IZU9_KIRREL3_4557_61	2234	0.901	0.862	0.94	3.15E-03	5.673	1077	0.820	0.691	0.973	1.638	0.023
SOMA_Q2TBF2_WSCD2_6274_15	2234	1.15	1.085	1.218	3.17E-03	5.672	1077	0.959	0.865	1.062	0.378	0.419
SOMA_P15529_CD46_17682_1	2234	1.156	1.089	1.228	3.21E-03	5.665	1077	1.167	1.093	1.246	5.405	<0.0001

SOMA_Q8NFZ4_NLGN2_9772_153	2234	1.153	1.087	1.223	3.26E-03	5.659	1077	1.064	1.004	1.128	1.443	0.0361
SOMA_P00749_PLAU_4158_54	2234	1.15	1.086	1.219	3.30E-03	5.653	1077	1.085	1.013	1.162	1.704	0.0198
SOMA_P02675_FGB_18890_227	2234	0.869	0.82	0.921	3.34E-03	5.648	1077	0.885	0.769	1.018	1.059	0.0872
SOMA_P53805_RCAN1_13465_5	2234	0.867	0.817	0.92	3.35E-03	5.647	1077	1.074	0.989	1.167	1.050	0.0892
SOMA_Q9UHF5_IL17B_3499_77	2234	0.897	0.857	0.938	3.36E-03	5.646	1077	0.953	0.843	1.077	0.355	0.4412
SOMA_Q9ULW6_NAP1L2_13529_39	2234	0.864	0.813	0.918	3.37E-03	5.644	1077	0.903	0.752	1.084	0.565	0.272
SOMA_P50238_CRIP1_18275_5	2234	0.87	0.821	0.922	3.40E-03	5.641	1077	0.699	0.621	0.788	8.456	<0.0001
SOMA_P52799_EFNB2_14131_37	2234	1.155	1.088	1.225	3.41E-03	5.639	1077	1.240	1.163	1.323	10.196	<0.0001
SOMA_Q16620_NTRK2_4866_59	2234	0.866	0.815	0.919	3.52E-03	5.626	1077	0.898	0.785	1.026	0.947	0.1131
SOMA_Q9BV40_VAMP8_7064_2	2234	0.868	0.818	0.92	3.58E-03	5.618	1077	1.114	1.048	1.184	3.273	0.0005
SOMA_O15173_PGRMC2_8681_93	2234	0.866	0.816	0.919	3.60E-03	5.616	1077	0.966	0.847	1.100	0.223	0.5986
SOMA_O60479_DLX3_11422_2	2234	0.871	0.823	0.923	3.66E-03	5.609	1077	1.033	0.963	1.108	0.441	0.3625
SOMA_Q9NVH1_DNAJC11_9783_75	2234	1.146	1.083	1.213	3.66E-03	5.609	1077	1.065	0.991	1.143	1.071	0.0849
SOMA_O43813_LANCL1_13986_6	2234	0.872	0.824	0.923	3.66E-03	5.608	1077	0.765	0.688	0.851	6.121	<0.0001
SOMA_Q8TDX7_NEK7_12703_6	2234	0.87	0.822	0.922	3.68E-03	5.606	1077	0.733	0.664	0.809	9.132	<0.0001
SOMA_P42679_MATK_3823_9	2234	1.144	1.082	1.211	3.70E-03	5.603	1077	1.014	0.930	1.105	0.120	0.7584
SOMA_Q09FC8_ZNF415_12811_55	2234	0.862	0.811	0.917	3.76E-03	5.596	1077	0.980	0.868	1.105	0.131	0.7393
SOMA_Q8TAC9_SCAMP5_13509_5	2234	0.864	0.814	0.919	3.77E-03	5.595	1077	1.070	1.000	1.145	1.315	0.0484
SOMA_Q86SG7_LYG2_15416_54	2234	0.871	0.823	0.923	3.79E-03	5.594	1077	0.958	0.832	1.104	0.255	0.5556

SOMA_O75962_TRIO_11126_102	2234	0.866	0.816	0.919	3.80E-03	5.592	1077	0.943	0.800	1.112	0.312	0.4875
SOMA_P32926_DSG3_16317_20	2234	0.871	0.823	0.923	3.88E-03	5.584	1077	0.714	0.621	0.821	5.645	<0.0001
SOMA_P00491_PNP_15435_4	2234	0.868	0.818	0.921	3.93E-03	5.577	1077	0.767	0.682	0.864	4.942	<0.0001
SOMA_P01178_OXT_8356_88	2234	1.146	1.082	1.212	3.97E-03	5.573	1077	1.077	1.005	1.153	1.450	0.0355
SOMA_Q9Y281_CFL2_15339_32	2234	1.151	1.085	1.221	3.98E-03	5.572	1077	1.261	1.176	1.353	9.985	<0.0001
SOMA_P13929_ENO3_16616_137	2234	0.869	0.819	0.921	4.00E-03	5.57	1077	1.046	0.971	1.128	0.625	0.2373
SOMA_Q2TAL6_VWC2_15308_108	2234	1.149	1.084	1.217	4.09E-03	5.56	1077	1.243	1.143	1.353	6.368	<0.0001
SOMA_Q96E17_RAB3C_19586_89	2234	0.871	0.822	0.923	4.16E-03	5.553	1077	0.809	0.736	0.889	4.996	<0.0001
SOMA_O95363_FARS2_13941_82	2234	0.877	0.83	0.927	4.22E-03	5.547	1077	0.896	0.709	1.132	0.448	0.3565
SOMA_O75884_RBBP9_10064_12	2234	0.864	0.813	0.919	4.29E-03	5.539	1077	0.534	0.424	0.673	6.993	<0.0001
SOMA_P01375_TNF_5936_53	2234	1.149	1.084	1.217	4.32E-03	5.537	1077	1.036	0.957	1.122	0.424	0.3766
SOMA_P43378_PTPN9_12633_3	2234	0.87	0.821	0.922	4.36E-03	5.532	1077	0.979	0.862	1.110	0.132	0.7379
SOMA_Q9GZP0_PDGFD_9341_1	2234	1.146	1.083	1.214	4.40E-03	5.528	1077	1.019	0.923	1.125	0.150	0.7079
SOMA_O43825_B3GALT2_11638_42	2234	0.875	0.828	0.926	4.43E-03	5.526	1077	0.883	0.754	1.035	0.906	0.1241
SOMA_P00568_AK1_5012_67	2234	0.869	0.819	0.922	4.42E-03	5.526	1077	0.752	0.671	0.842	6.103	<0.0001
SOMA_O95696_BRD1_11607_15	2234	1.14	1.079	1.205	4.48E-03	5.521	1077	0.976	0.866	1.100	0.161	0.6906
SOMA_Q8WWK9_CKAP2_5345_51	2234	0.87	0.821	0.923	4.49E-03	5.52	1077	0.685	0.609	0.771	9.492	<0.0001
SOMA_Q14914_PTGR1_19617_5	2234	1.151	1.085	1.22	4.51E-03	5.518	1077	1.088	0.993	1.192	1.152	0.0704
SOMA_Q8IZ57_NRSN1_11654_77	2234	0.869	0.819	0.922	4.55E-03	5.514	1077	1.081	1.010	1.157	1.593	0.0255

SOMA_Q96A84_EMID1_13021_12	2234	0.875	0.827	0.925	4.55E-03	5.514	NA	NA	NA	NA	NA	NA
SOMA_P49006_MARCKSL_19175_18	2234	1.144	1.081	1.21	4.58E-03	5.511	1077	1.126	1.044	1.214	2.690	0.002
SOMA_P05162_LGALS2_3033_57	2234	1.147	1.083	1.215	4.66E-03	5.503	1077	1.030	0.962	1.103	0.408	0.391
SOMA_P18031_PTPN1_3005_5	2234	0.869	0.819	0.922	4.76E-03	5.494	1077	0.805	0.701	0.924	2.703	0.002
SOMA_Q8NFP4_MDGA1_16900_29	2234	1.146	1.082	1.214	4.77E-03	5.494	1077	1.089	0.988	1.200	1.061	0.087
SOMA_B1AKI9_ISM1_8355_80	2234	1.144	1.081	1.211	4.83E-03	5.487	NA	NA	NA	NA	NA	NA
SOMA_P61601_NCALD_17816_58	2234	0.878	0.832	0.928	4.88E-03	5.483	1077	0.895	0.718	1.114	0.496	0.3191
SOMA_Q6ZUB0_SPATA31_9524_46	2234	0.868	0.817	0.921	4.97E-03	5.475	1077	0.912	0.618	1.346	0.192	0.6425
SOMA_Q14974_KPNB1_3887_90	2234	0.871	0.822	0.923	5.04E-03	5.469	1077	0.828	0.750	0.913	3.821	0.0002
SOMA_Q15465_SHH_2743_5	2234	0.872	0.823	0.924	5.07E-03	5.467	1077	1.025	0.948	1.108	0.273	0.5337
SOMA_P08684_CYP3A4_2943_5	2234	0.872	0.823	0.924	5.31E-03	5.446	1077	0.706	0.626	0.796	7.864	<0.0001
SOMA_P22607_FGFR3_13669_6	2234	0.869	0.819	0.922	5.36E-03	5.443	1077	0.933	0.842	1.035	0.722	0.1896
SOMA_Q15648_MED1_3892_21	2234	1.143	1.08	1.209	5.48E-03	5.433	1077	1.037	0.959	1.121	0.444	0.3599
SOMA_Q15102_PAFAH1B_12374_8	2234	0.87	0.82	0.923	5.49E-03	5.432	1077	0.663	0.568	0.773	6.770	<0.0001
SOMA_P14550_AKR1A1_4192_10	2234	1.147	1.082	1.215	5.65E-03	5.419	1077	0.936	0.835	1.048	0.603	0.2494
SOMA_P18850_ATF6_11277_23	2234	0.872	0.822	0.924	5.67E-03	5.418	1077	0.917	0.826	1.017	0.990	0.1024
SOMA_Q9Y653_GPR56_18893_26	2234	1.152	1.085	1.223	5.80E-03	5.408	NA	NA	NA	NA	NA	NA
SOMA_P17936_IGFBP3_2571_12	2234	0.868	0.817	0.922	5.98E-03	5.395	1077	0.881	0.794	0.978	1.749	0.0178
SOMA_Q01469_FABP5_4985_11	2234	1.14	1.078	1.206	6.05E-03	5.39	1077	0.657	0.238	1.813	0.379	0.4174

SOMA_P55287_CDH11_16305_10	2234	1.147	1.082	1.215	6.14E-03	5.384	1077	1.137	1.044	1.238	2.508	0.0031
SOMA_Q92888_ARHGEF1_13976_9	2234	0.873	0.824	0.925	6.13E-03	5.384	1077	0.857	0.736	0.999	1.315	0.0484
SOMA_P79483_HLA_DRB_6962_5	2234	0.871	0.822	0.924	6.20E-03	5.379	1077	0.870	0.769	0.984	1.582	0.0262
SOMA_Q7Z4H8_KDELC2_8296_117	2234	0.869	0.818	0.922	6.23E-03	5.377	NA	NA	NA	NA	NA	NA
SOMA_P37173_TGFBR2_5133_17	2234	0.87	0.819	0.923	6.41E-03	5.364	1077	0.957	0.842	1.087	0.303	0.4977
SOMA_Q8NFS9_GCNT2_7143_9	2234	1.141	1.079	1.207	6.48E-03	5.36	NA	NA	NA	NA	NA	NA
SOMA_P54750_PDE1A_5253_1	2234	1.141	1.079	1.207	6.51E-03	5.358	1077	0.982	0.873	1.105	0.116	0.7654
SOMA_Q9BYC5_FUT8_8244_16	2234	0.871	0.82	0.924	6.55E-03	5.355	1077	0.819	0.693	0.969	1.708	0.0196
SOMA_Q13148_TARDBP_12046_51	2234	0.894	0.852	0.938	6.57E-03	5.354	1077	0.849	0.741	0.974	1.711	0.0195
SOMA_Q9UMR7_CLEC4A_8853_2	2234	0.875	0.827	0.926	6.58E-03	5.353	1077	1.068	0.967	1.179	0.708	0.1959
SOMA_O14732_IMPA2_12581_39	2234	0.869	0.818	0.923	6.69E-03	5.346	1077	0.989	0.885	1.106	0.071	0.8485
SOMA_Q8NCG5_CHST4_18876_77	2234	1.145	1.081	1.214	6.73E-03	5.343	1077	1.078	1.019	1.139	2.067	0.0086
SOMA_P31749_AKT1_15627_83	2234	0.872	0.822	0.924	6.78E-03	5.34	1077	0.778	0.707	0.858	6.403	<0.0001
SOMA_P01579_IFNG_15346_31	2234	0.871	0.821	0.924	6.79E-03	5.339	1077	0.932	0.818	1.061	0.541	0.2877
SOMA_P53365_ARFIP2_12630_8	2234	0.873	0.824	0.925	6.82E-03	5.338	1077	1.000	0.903	1.108	0.001	0.9974
SOMA_Q9UKW6_ELF5_13457_33	2234	0.869	0.819	0.923	6.96E-03	5.329	1077	0.967	0.841	1.112	0.195	0.638
SOMA_P31321_PRKAR1B_12479_50	2234	1.141	1.079	1.208	7.01E-03	5.326	1077	1.018	0.931	1.114	0.161	0.6898
SOMA_Q9H4I9_SMDT1_9497_3	2234	0.872	0.823	0.925	7.03E-03	5.324	NA	NA	NA	NA	NA	NA
SOMA_P26440_IVD_17737_7	2234	0.873	0.823	0.925	7.16E-03	5.316	1077	0.818	0.739	0.904	4.058	<0.0001

SOMA_Q8IUB5_WFDC13_9345_436	2234	0.886	0.841	0.933	7.17E-03	5.316	1077	1.031	0.955	1.113	0.357	0.4398
SOMA_P17948_FLT1_8231_122	2234	1.157	1.087	1.232	7.21E-03	5.313	1077	1.133	1.080	1.188	6.507	<0.0001
SOMA_P51148_RAB5C_14287_6	2234	0.875	0.826	0.926	7.28E-03	5.309	1077	0.766	0.698	0.841	7.691	<0.0001
SOMA_Q13591_SEMA5A_13132_14	2234	1.15	1.083	1.221	7.30E-03	5.308	1077	1.216	1.114	1.327	4.917	<0.0001
SOMA_P51814_ZNF41_10003_15	2234	0.869	0.818	0.923	7.52E-03	5.295	1077	0.932	0.811	1.070	0.502	0.3144
SOMA_Q9NXA8_SIRT5_12461_8	2234	0.87	0.819	0.924	7.52E-03	5.295	1077	0.924	0.805	1.060	0.588	0.2584
SOMA_Q13477_MADCAM1_11258_41	2234	0.871	0.821	0.924	7.58E-03	5.292	1077	0.772	0.655	0.908	2.735	0.0018
SOMA_Q16222_UAP1_13580_2	2234	0.878	0.83	0.929	7.64E-03	5.288	1077	0.863	0.670	1.111	0.597	0.2531
SOMA_O14598_VCY_6295_67	2234	0.876	0.828	0.928	7.67E-03	5.287	NA	NA	NA	NA	NA	NA
SOMD P31749 AKT1 AK 339268 PASS	2234	0.873	0.824	0.926	7.67E-03	5.286	NA	NA	NA	NA	NA	NA
SOMD P31751 AKT1 AK 339268 PASS	2234	0.873	0.824	0.926	7.67E-03	5.286	NA	NA	NA	NA	NA	NA
SOMD Q9Y243 AKT1 AK 339268 PASS	2234	0.873	0.824	0.926	7.67E-03	5.286	NA	NA	NA	NA	NA	NA
SOMA_Q8N9N8_EIF1AD_13545_97	2234	1.142	1.078	1.209	8.26E-03	5.254	1077	0.844	0.637	1.117	0.629	0.2351
SOMA_P01591_IGJ_15306_20	2234	0.875	0.826	0.927	8.36E-03	5.249	NA	NA	NA	NA	NA	NA
SOMA_O60861_GAS7_17721_82	2234	0.882	0.835	0.931	8.38E-03	5.248	1077	1.055	0.989	1.125	0.976	0.1058
SOMA_P54764_EPHA4_16288_17	2234	0.872	0.822	0.925	8.43E-03	5.245	1077	1.011	0.911	1.121	0.075	0.8416
SOMA_Q06190_PPP2R3A_13665_35	2234	0.875	0.826	0.927	8.61E-03	5.236	1077	1.068	0.990	1.153	1.052	0.0888
SOMA_P08581_MET_2837_3	2234	0.873	0.824	0.926	8.69E-03	5.232	1077	0.906	0.814	1.009	1.133	0.0735
SOMA_O00142_TK2_19114_8	2234	0.872	0.822	0.926	8.71E-03	5.231	1077	1.003	0.916	1.097	0.021	0.9537

SOMA_O14960_LECT2_16763_11	2234	0.872	0.821	0.925	8.76E-03	5.228	1077	0.639	0.572	0.713	14.899	<0.0001
SOMA_Q16619_CTF1_2889_37	2234	0.894	0.852	0.938	8.83E-03	5.225	1077	0.801	0.616	1.042	1.006	0.0986
SOMA_Q9BRR6_ADPGK_6221_1	2234	1.15	1.082	1.222	8.86E-03	5.224	1077	1.262	1.176	1.354	10.035	<0.0001
SOMA_Q12836_ZP4_7766_25	2234	0.871	0.82	0.925	8.99E-03	5.217	1077	0.964	0.858	1.084	0.265	0.543
SOMA_P40259_CD79B_6351_55	2234	0.872	0.822	0.925	9.20E-03	5.207	1077	1.011	0.921	1.110	0.086	0.8195
SOMA_P48643_CCT5_12569_25	2234	0.875	0.826	0.927	9.26E-03	5.204	1077	0.818	0.740	0.905	4.000	0.0001
SOMA_Q8NFX7_STXBP6_18308_30	2234	0.877	0.828	0.928	9.33E-03	5.201	1077	0.664	0.549	0.803	4.623	<0.0001
SOMA_Q16206_ENOX2_13422_66	2234	0.886	0.84	0.934	9.77E-03	5.181	1077	1.044	0.967	1.127	0.563	0.2738
SOMA_P15531_NME1_5909_51	2234	0.876	0.827	0.928	9.80E-03	5.18	1077	1.054	0.981	1.132	0.815	0.1531
SOMA_Q6UWV7_FAM159A_13431_74	2234	1.142	1.078	1.211	9.89E-03	5.176	NA	NA	NA	NA	NA	NA
SOMA_Q9P121_NTM_10907_116	2234	0.874	0.824	0.926	9.92E-03	5.174	1077	0.927	0.837	1.028	0.820	0.1512
SOMA_Q15363_TMED2_10761_5	2234	1.139	1.076	1.206	1.00E-02	5.17	1077	1.132	1.058	1.212	3.458	0.0003
SOMA_Q9P218_COL20A1_8804_39	2234	0.873	0.823	0.926	1.00E-02	5.169	1077	0.992	0.891	1.104	0.053	0.8844
SOMA_O75791_GRAP2_16074_12	2234	0.877	0.828	0.928	1.01E-02	5.168	1077	0.811	0.705	0.933	2.474	0.0034
SOMA_P50461_CSRP3_9171_11	2234	1.144	1.079	1.212	1.04E-02	5.155	1077	1.097	0.996	1.209	1.217	0.0606
SOMA_P15248_IL9_5834_18	2234	1.144	1.079	1.213	1.05E-02	5.15	1077	1.073	1.023	1.126	2.376	0.0042
SOMA_Q9H6B4_CLMP_10440_26	2234	1.146	1.08	1.216	1.05E-02	5.15	1077	1.034	0.964	1.110	0.458	0.3482
SOMA_Q8WWU5_TCP11_8334_65	2234	0.875	0.826	0.928	1.05E-02	5.148	1077	0.979	0.877	1.093	0.149	0.7103
SOMA_Q9UHY1_NRBP1_12616_45	2234	0.877	0.828	0.929	1.06E-02	5.145	1077	0.847	0.709	1.012	1.169	0.0678

SOMA_P31751_AKT2_14685_17	2234	0.872	0.821	0.926	1.07E-02	5.142	1077	0.725	0.622	0.845	4.415	<0.0001
SOMA_P17405_SMPD1_10818_36	2234	1.144	1.078	1.213	1.07E-02	5.14	1077	1.165	1.073	1.266	3.528	0.0003
SOMA_Q9HAU4_SMURF2_13985_12	2234	0.868	0.816	0.923	1.07E-02	5.14	1077	1.050	0.978	1.128	0.749	0.1783
SOMA_O95969_SCGB1D2_6508_68	2234	0.873	0.823	0.926	1.09E-02	5.135	1077	0.803	0.698	0.925	2.622	0.0024
SOMA_O95460_MATN4_7083_74	2234	0.871	0.82	0.925	1.10E-02	5.128	1077	0.954	0.829	1.097	0.294	0.5086
SOMA_Q9Y5U9_IER3IP1_6412_26	2234	0.875	0.826	0.928	1.11E-02	5.126	NA	NA	NA	NA	NA	NA
SOMA_Q8TAD4_SLC30A5_9594_30	2234	0.875	0.825	0.927	1.12E-02	5.121	1077	0.860	0.769	0.963	2.060	0.0087
SOMA_Q99728_BARD1_13977_28	2234	0.871	0.819	0.925	1.14E-02	5.114	1077	0.608	0.127	2.904	0.274	0.5326
SOMA_P49863_GZMK_9545_156	2234	0.872	0.821	0.926	1.15E-02	5.11	1077	0.950	0.772	1.167	0.205	0.6232
SOMA_P31948_STIP1_5489_18	2234	0.875	0.826	0.928	1.16E-02	5.105	1077	0.844	0.752	0.947	2.422	0.0038
SOMA_Q16875_PFKFB3_12456_5	2234	0.877	0.828	0.929	1.18E-02	5.1	1077	0.940	0.828	1.067	0.474	0.3354
SOMA_O15305_PMM2_17794_6	2234	0.876	0.826	0.928	1.18E-02	5.097	1077	0.832	0.753	0.920	3.508	0.0003
SOMA_P12821_ACE_10714_7	2234	0.876	0.827	0.929	1.19E-02	5.096	1077	0.673	0.582	0.779	6.985	<0.0001
SOMA_Q16644_MAPKAPK_3822_54	2234	0.876	0.826	0.928	1.19E-02	5.094	1077	0.809	0.733	0.892	4.674	<0.0001
SOMA_Q9Y231_FUT9_6991_24	2234	0.882	0.835	0.932	1.21E-02	5.089	1077	0.924	0.810	1.053	0.630	0.2344
SOMA_O95989_NUDT3_12796_44	2234	1.142	1.077	1.21	1.22E-02	5.084	1077	0.991	0.879	1.118	0.052	0.8878
SOMA_Q9H3W5_LRRN3_10471_25	2234	0.88	0.832	0.931	1.23E-02	5.08	NA	NA	NA	NA	NA	NA
SOMA_P51946_CCNH_9848_22	2234	1.138	1.075	1.205	1.24E-02	5.078	1077	1.060	0.986	1.138	0.946	0.1133
SOMA_Q4V9L6_TMEM119_11110_4	2234	0.871	0.82	0.926	1.24E-02	5.077	1077	0.920	0.783	1.081	0.504	0.3133

SOMA_Q5VU65_NUP210L_9606_4	2234	1.141	1.076	1.209	1.25E-02	5.074	1077	0.955	0.830	1.097	0.290	0.513
SOMA_P14618_PKM2_4240_31	2234	0.88	0.831	0.931	1.25E-02	5.073	NA	NA	NA	NA	NA	NA
SOMA_Q96A72_MAGOHB_16875_13	2234	1.137	1.074	1.202	1.26E-02	5.069	1077	0.998	0.881	1.131	0.010	0.9765
SOMA_Q6UX71_PLXDC2_11342_59	2234	0.876	0.827	0.929	1.27E-02	5.065	1077	0.862	0.751	0.991	1.437	0.0365
SOMA_Q495A1_TIGIT_16299_13	2234	0.878	0.829	0.93	1.28E-02	5.064	1077	0.573	0.390	0.841	2.354	0.0044
SOMA_P05019_IGF1_2952_75	2234	0.871	0.82	0.926	1.28E-02	5.063	1077	0.916	0.823	1.020	0.954	0.1111
SOMA_P09326_CD48_3292_75	2234	1.146	1.079	1.217	1.28E-02	5.062	1077	1.203	1.119	1.293	6.298	<0.0001
SOMA_P30419_NMT1_5196_7	2234	0.875	0.825	0.928	1.28E-02	5.062	1077	1.030	0.946	1.122	0.303	0.4978
SOMA_Q969J5_IL22RA2_5087_5	2234	0.873	0.823	0.927	1.31E-02	5.053	1077	0.776	0.667	0.902	2.998	0.001
SOMA_P01732_CD8A_15462_28	2234	1.144	1.078	1.213	1.32E-02	5.051	1077	1.226	1.135	1.323	6.727	<0.0001
SOMA_Q15722_LTB4R_13477_65	2234	0.878	0.83	0.93	1.33E-02	5.046	1077	0.678	0.578	0.795	5.775	<0.0001
SOMA_Q6PL18_ATAD2_13043_157	2234	0.901	0.861	0.944	1.34E-02	5.044	1077	0.958	0.839	1.095	0.273	0.5336
SOMA_P30041_PRDX6_5018_68	2234	0.875	0.825	0.928	1.38E-02	5.03	1077	0.722	0.654	0.797	9.994	<0.0001
SOMA_O43427_FIBP_19169_88	2234	0.872	0.82	0.926	1.40E-02	5.022	1077	0.729	0.651	0.816	7.352	<0.0001
SOMA_Q9Y5F6_PCDHGC5_7983_1	2234	0.87	0.819	0.926	1.40E-02	5.022	1077	0.960	0.844	1.093	0.269	0.5382
SOMA_P35916_FLT4_16035_8	2234	1.144	1.078	1.214	1.41E-02	5.02	1077	1.459	1.352	1.574	21.595	<0.0001
SOMA_Q9BTV7_CABLES2_7105_7	2234	0.875	0.825	0.928	1.41E-02	5.02	1077	1.028	0.952	1.109	0.317	0.4816
SOMA_Q9NR61_DLL4_3305_6	2234	1.141	1.076	1.21	1.44E-02	5.012	1077	1.061	1.000	1.125	1.285	0.0519
SOMA_Q92598_HSPH1_17704_74	2234	1.14	1.076	1.209	1.49E-02	4.997	1077	0.944	0.767	1.161	0.234	0.583

SOMA_O75521_ECI2_12859_33	2234	0.876	0.827	0.929	1.52E-02	4.987	1077	0.941	0.849	1.044	0.598	0.2526
SOMA_Q8TEB7_RNF128_8633_18	2234	0.874	0.823	0.928	1.57E-02	4.975	1077	0.907	0.785	1.048	0.730	0.1864
SOMA_P45973_CBX5_4540_11	2234	0.876	0.825	0.929	1.58E-02	4.97	1077	0.998	0.892	1.115	0.015	0.9666
SOMA_Q9BZM6_ULBP1_3081_70	2234	1.135	1.073	1.201	1.61E-02	4.962	1077	0.848	0.569	1.263	0.380	0.4167
SOMA_Q8NBI6_XXYLT1_6375_75	2234	1.141	1.076	1.211	1.62E-02	4.96	1077	1.097	1.051	1.145	4.594	<0.0001
SOMA_P35052_GPC1_8697_38	2234	0.877	0.827	0.93	1.62E-02	4.959	1077	1.006	0.911	1.111	0.040	0.9114
SOMA_O95295_SNAPIN_6975_52	2234	0.886	0.839	0.935	1.63E-02	4.958	1077	0.911	0.667	1.245	0.252	0.5594
SOMA_P09493_TPM1_5033_27	2234	0.873	0.822	0.928	1.64E-02	4.956	1077	1.102	1.024	1.186	2.013	0.0097
SOMA_Q16557_PSG3_6444_15	2234	1.138	1.074	1.206	1.63E-02	4.956	1077	1.051	0.993	1.113	1.060	0.0871
SOMA_P56279_TCL1A_17210_2	2234	0.875	0.824	0.928	1.64E-02	4.954	1077	0.816	0.670	0.995	1.353	0.0443
SOMA_Q8N302_AGGF1_8051_10	2234	0.876	0.825	0.929	1.67E-02	4.947	1077	0.935	0.813	1.074	0.468	0.3407
SOMA_P55209_NAP1L1_13636_20	2234	0.879	0.83	0.931	1.73E-02	4.932	1077	0.779	0.709	0.855	6.778	<0.0001
SOMA_P06400_RB1_5024_67	2234	0.874	0.823	0.928	1.74E-02	4.928	1077	0.873	0.727	1.048	0.835	0.1462
SOMA_Q9C000_NLRP1_11661_11	2234	0.876	0.825	0.929	1.75E-02	4.926	1077	1.237	1.136	1.346	6.068	<0.0001
SOMA_O43765_SGTA_3868_8	2234	0.878	0.829	0.931	1.77E-02	4.922	1077	0.798	0.721	0.883	4.864	<0.0001
SOMA_P06744_GPI_4272_46	2234	0.875	0.824	0.929	1.81E-02	4.911	1077	0.858	0.776	0.948	2.565	0.0027
SOMA_Q9BSI4_TINF2_10638_1	2234	0.891	0.846	0.938	1.84E-02	4.905	1077	0.661	0.351	1.246	0.697	0.201
SOMA_Q9BY11_PACSIN1_12676_1	2234	0.88	0.832	0.932	1.86E-02	4.899	1077	1.035	0.928	1.156	0.271	0.5359
SOMA_P28067_HLA_DMA_10639_1	2234	1.137	1.073	1.205	1.87E-02	4.896	NA	NA	NA	NA	NA	NA

SOMA_A6NI73_LILRA5_8766_29	2234	1.142	1.076	1.211	1.88E-02	4.894	1077	1.299	1.206	1.399	11.258	<0.0001
SOMA_Q6UXQ4_C2orf66_5677_15	2234	0.878	0.828	0.931	1.89E-02	4.893	1077	0.621	0.202	1.905	0.393	0.4049
SOMA_P43357_MAGEA3_12576_21	2234	0.878	0.828	0.931	1.90E-02	4.891	1077	1.027	0.935	1.128	0.239	0.5762
SOMA_O00443_PIK3C2A_14028_22	2234	0.88	0.831	0.932	1.91E-02	4.887	1077	0.775	0.624	0.963	1.672	0.0213
SOMA_O14929_HAT1_19327_31	2234	0.879	0.829	0.931	1.92E-02	4.886	1077	0.899	0.801	1.009	1.150	0.0707
SOMA_Q5J8M3_EMC4_13516_46	2234	0.879	0.829	0.932	2.01E-02	4.865	1077	0.608	0.497	0.743	5.911	<0.0001
SOMA_Q9NRW1_RAB6B_14271_23	2234	0.88	0.831	0.932	2.01E-02	4.865	1077	0.786	0.713	0.868	5.766	<0.0001
SOMA_Q9UKL4_GJD2_11678_105	2234	0.876	0.825	0.93	2.01E-02	4.864	1077	0.940	0.804	1.100	0.355	0.4413
SOMA_Q14155_ARHGEF7_13932_45	2234	0.884	0.836	0.934	2.06E-02	4.854	1077	1.011	0.905	1.130	0.071	0.8488
SOMA_P43405_SYK_10722_13	2234	0.882	0.834	0.934	2.09E-02	4.847	1077	0.749	0.644	0.871	3.780	0.0002
SOMA_Q9UPY8_MAPRE3_16885_49	2234	0.881	0.832	0.933	2.10E-02	4.846	1077	0.839	0.765	0.921	3.655	0.0002
SOMA_Q8N428_GALNT16_8923_94	2234	1.137	1.073	1.205	2.13E-02	4.84	1077	1.095	1.010	1.188	1.558	0.0277
SOMA_Q9UNA0_ADAMTS5_3168_8	2234	1.136	1.072	1.203	2.13E-02	4.84	1077	1.097	1.038	1.160	2.968	0.0011
SOMA_A0PJX4_SHISA3_7057_18	2234	1.141	1.075	1.211	2.15E-02	4.837	NA	NA	NA	NA	NA	NA
SOMA_Q96HD1_CRELD1_7628_40	2234	1.138	1.074	1.207	2.19E-02	4.829	1077	1.195	1.083	1.320	3.379	0.0004
SOMA_Q9GZN6_SLC6A16_13056_18	2234	0.9	0.859	0.944	2.20E-02	4.826	1077	0.995	0.901	1.098	0.038	0.9152
SOMA_P19835_CEL_9796_4	2234	0.88	0.83	0.932	2.20E-02	4.825	1077	0.827	0.731	0.936	2.568	0.0027
SOMA_O95479_H6PD_7161_25	2234	1.143	1.076	1.215	2.22E-02	4.822	1077	1.405	1.302	1.516	17.797	<0.0001
SOMA_Q5BIV9_SPRN_13447_42	2234	1.142	1.075	1.213	2.22E-02	4.822	1077	1.058	0.990	1.131	1.030	0.0934

SOMA_P42830_CXCL5_2979_8	2234	0.895	0.851	0.941	2.24E-02	4.818	1077	1.009	0.915	1.112	0.064	0.8637
SOMA_P08319_ADH4_8325_37	2234	0.875	0.824	0.93	2.25E-02	4.816	1077	0.915	0.810	1.033	0.817	0.1524
SOMA_Q8N475_FSTL5_7099_33	2234	0.88	0.83	0.932	2.25E-02	4.815	1077	0.993	0.895	1.102	0.049	0.8935
SOMA_A6NC05_C5orf63_13378_80	2234	0.882	0.833	0.934	2.34E-02	4.799	1077	1.041	0.951	1.139	0.414	0.3853
SOMA_O00238_BMPR1B_10550_37	2234	0.893	0.849	0.94	2.35E-02	4.796	1077	1.060	0.981	1.145	0.850	0.1412
SOMA_Q13907_IDI1_17712_7	2234	1.139	1.074	1.209	2.37E-02	4.793	1077	1.118	1.013	1.234	1.571	0.0268
SOMA_P35813_PPM1A_12619_14	2234	1.139	1.074	1.209	2.39E-02	4.789	1077	1.069	1.005	1.138	1.466	0.0342
SOMA_Q8WTT0_CLEC4C_9094_5	2234	0.878	0.828	0.932	2.39E-02	4.789	1077	0.814	0.713	0.930	2.619	0.0024
SOMA_Q96A57_TMEM230_11542_11	2234	0.884	0.836	0.935	2.41E-02	4.785	1077	0.939	0.737	1.195	0.217	0.6064
SOMA_P30040_ERP29_13728_19	2234	0.879	0.829	0.932	2.44E-02	4.781	1077	0.809	0.720	0.910	3.403	0.0004
SOMA_P49773_HINT1_5900_11	2234	1.137	1.072	1.205	2.43E-02	4.781	1077	1.051	0.995	1.110	1.109	0.0778
SOMA_Q9UQ80_PA2G4_4258_15	2234	0.879	0.829	0.932	2.45E-02	4.778	1077	0.805	0.733	0.884	5.198	<0.0001
SOMA_Q96LB9_PGLYRP3_10561_5	2234	0.885	0.836	0.935	2.52E-02	4.765	1077	1.048	0.980	1.121	0.771	0.1696
SOMA_P35520_CBS_10086_39	2234	0.881	0.832	0.934	2.56E-02	4.76	1077	1.102	1.009	1.205	1.503	0.0314
SOMA_O43761_SYNGR3_13375_48	2234	0.877	0.826	0.931	2.60E-02	4.752	1077	1.014	0.938	1.095	0.135	0.7325
SOMA_P36268_GGT2_6334_9	2234	0.878	0.828	0.932	2.60E-02	4.752	1077	0.819	0.692	0.970	1.677	0.021
SOMA_Q99733_NAP1L4_19188_21	2234	0.881	0.832	0.934	2.61E-02	4.751	1077	0.796	0.714	0.887	4.458	<0.0001
SOMA_Q11201_ST3GAL1_5657_28	2234	0.884	0.836	0.935	2.63E-02	4.747	1077	0.933	0.827	1.053	0.584	0.2603
SOMA_P49959_MRE11A_11319_106	2234	0.879	0.828	0.932	2.70E-02	4.736	NA	NA	NA	NA	NA	NA

SOMA_Q6ZSG1_RNF165_11561_32	2234	1.135	1.071	1.203	2.70E-02	4.735	NA	NA	NA	NA	NA	NA
SOMA_P09496_CLTA_19257_11	2234	1.145	1.076	1.219	2.79E-02	4.722	1077	1.075	0.982	1.177	0.931	0.1171
SOMA_P08684_CYP3A4_7879_12	2234	0.879	0.828	0.932	2.79E-02	4.72	1077	0.932	0.764	1.138	0.309	0.4914
SOMA_Q99497_PARK7_19545_145	2234	0.878	0.827	0.932	2.80E-02	4.72	1077	1.018	0.896	1.156	0.105	0.7858
SOMA_Q5S007_LRRK2_10990_21	2234	0.882	0.832	0.934	2.86E-02	4.711	1077	0.678	0.519	0.885	2.376	0.0042
SOMA_Q9Y2I2_NTNG1_5637_81	2234	1.136	1.071	1.204	2.91E-02	4.703	1077	1.116	1.065	1.171	5.241	<0.0001
SOMA_Q8TE99_ACPL2_6079_59	2234	0.898	0.854	0.943	2.92E-02	4.702	NA	NA	NA	NA	NA	NA
SOMA_Q92478_CLEC2B_7786_83	2234	0.88	0.829	0.933	2.92E-02	4.701	1077	1.051	0.985	1.123	0.878	0.1325
SOMA_Q9BXB1_LGR4_16304_6	2234	0.878	0.827	0.932	2.92E-02	4.701	1077	1.067	0.995	1.144	1.170	0.0676
SOMA_P27469_G0S2_8931_124	2234	0.88	0.83	0.933	2.94E-02	4.699	1077	0.972	0.848	1.114	0.165	0.6837
SOMA_Q9Y3C6_PPIL1_9884_8	2234	0.882	0.832	0.934	2.96E-02	4.695	1077	0.760	0.651	0.888	3.250	0.0006
SOMA_Q16186_ADRM1_9057_19	2234	0.875	0.823	0.931	3.02E-02	4.686	1077	0.994	0.896	1.103	0.041	0.9098
SOMA_Q04828_AKR1C1_12618_50	2234	0.879	0.828	0.933	3.10E-02	4.675	1077	0.619	0.460	0.832	2.825	0.0015
SOMA_Q9Y4Z0_LSM4_16854_17	2234	0.881	0.83	0.934	3.12E-02	4.673	1077	0.887	0.789	0.996	1.362	0.0434
SOMA_O75096_LRP4_19558_10	2234	1.134	1.07	1.202	3.13E-02	4.67	1077	1.117	1.048	1.190	3.179	0.0007
SOMA_Q9Y2C2_UST_8364_74	2234	0.879	0.828	0.933	3.15E-02	4.667	1077	1.017	0.916	1.129	0.122	0.7557
SOMA_O75828_CBR3_14091_42	2234	0.885	0.836	0.936	3.16E-02	4.666	1077	0.617	0.546	0.698	13.881	<0.0001
SOMA_P22362_CCL1_2770_51	2234	0.879	0.828	0.933	3.20E-02	4.66	1077	0.850	0.745	0.969	1.812	0.0154
SOMA_Q86UX7_FERMT3_12599_10	2234	0.878	0.827	0.932	3.26E-02	4.652	1077	0.810	0.735	0.892	4.673	<0.0001

SOMA_Q9UBP9_GULP1_19180_38	2234	0.879	0.828	0.933	3.40E-02	4.634	1077	0.844	0.762	0.936	2.883	0.0013
SOMA_P21757_MSR1_11207_3	2234	0.884	0.835	0.936	3.43E-02	4.63	1077	0.929	0.793	1.088	0.442	0.3612
SOMA_Q9NYA1_SPHK1_4294_16	2234	0.884	0.835	0.936	3.44E-02	4.629	1077	0.832	0.752	0.921	3.387	0.0004
SOMA_O00541_PES1_4267_81	2234	1.131	1.068	1.197	3.45E-02	4.627	1077	0.987	0.885	1.102	0.086	0.8211
SOMA_P31947_SFN_4829_43	2234	1.141	1.073	1.213	3.46E-02	4.627	1077	1.106	1.054	1.161	4.336	<0.0001
SOMA_O75354_ENTPD6_8932_1	2234	0.88	0.83	0.934	3.47E-02	4.625	1077	0.744	0.587	0.944	1.831	0.0147
SOMA_Q99729_HNRNPAB_8894_80	2234	1.131	1.068	1.197	3.47E-02	4.625	1077	0.966	0.862	1.083	0.255	0.5565
SOMA_Q5T601_ADGRF1_11243_90	2234	0.883	0.833	0.935	3.49E-02	4.623	1077	1.051	0.970	1.140	0.648	0.2251
SOMA_P10696_ALPPL2_6706_18	2234	0.888	0.841	0.938	3.55E-02	4.615	NA	NA	NA	NA	NA	NA
SOMA_O76064_RNF8_14663_44	2234	0.881	0.831	0.934	3.56E-02	4.614	1077	0.873	0.721	1.056	0.792	0.1615
SOMA_P62308_SNRPG_19276_124	2234	1.13	1.068	1.197	3.56E-02	4.613	1077	0.582	0.448	0.756	4.288	<0.0001
SOMA_O75503_CLN5_8874_53	2234	1.137	1.071	1.207	3.58E-02	4.611	1077	1.047	0.969	1.130	0.614	0.2431
SOMA_P28074_PSMB5_12580_7	2234	0.878	0.827	0.933	3.61E-02	4.608	1077	1.056	0.985	1.132	0.913	0.1221
SOMA_P08069_IGF1R_4232_19	2234	1.141	1.073	1.213	3.61E-02	4.607	1077	1.488	1.355	1.634	16.019	<0.0001
SOMA_P30086_PEBP1_4276_10	2234	1.134	1.069	1.202	3.61E-02	4.607	1077	0.925	0.826	1.036	0.750	0.178
SOMA_Q9UN37_VPS4A_11476_43	2234	0.883	0.834	0.936	3.62E-02	4.607	1077	0.848	0.766	0.938	2.837	0.0015
SOMA_Q07507_DPT_4979_34	2234	1.132	1.068	1.199	3.63E-02	4.605	1077	1.061	0.984	1.145	0.905	0.1245
SOMA_Q9NRY6_PLSCR3_11407_57	2234	0.881	0.83	0.934	3.63E-02	4.605	1077	0.953	0.830	1.093	0.309	0.4913
SOMA_Q96CN7_ISOC1_9816_37	2234	1.132	1.069	1.199	3.69E-02	4.597	1077	0.853	0.772	0.942	2.753	0.0018

SOMA_Q8NAT1_POMGNT2_6359_50	2234	1.134	1.07	1.202	3.71E-02	4.596	1077	1.034	0.962	1.110	0.437	0.3653
SOMA_Q8IY33_MICALL2_12891_1	2234	0.883	0.833	0.936	3.72E-02	4.594	1077	1.014	0.927	1.108	0.115	0.7668
SOMA_Q8TCF1_ZFAND1_19173_5	2234	0.884	0.834	0.936	3.72E-02	4.594	1077	0.740	0.669	0.818	8.407	<0.0001
SOMA_Q96QV1_HHIP_10833_64	2234	0.876	0.824	0.932	3.84E-02	4.581	1077	0.900	0.788	1.028	0.914	0.1218
SOMA_Q4G0W2_DUSP28_9940_35	2234	0.883	0.833	0.936	3.87E-02	4.576	1077	0.806	0.726	0.894	4.356	<0.0001
SOMA_Q6PCB0_VWA1_6385_63	2234	1.132	1.068	1.2	3.88E-02	4.575	1077	1.140	1.089	1.192	7.870	<0.0001
SOMA_Q9HB29_IL1RL2_2994_71	2234	0.891	0.844	0.94	3.90E-02	4.573	1077	0.849	0.715	1.007	1.221	0.0601
SOMA_P50570_DNM2_11572_4	2234	0.883	0.834	0.936	3.93E-02	4.569	1077	0.813	0.737	0.898	4.356	<0.0001
SOMA_Q13077_TRAF1_17747_45	2234	0.884	0.835	0.937	3.95E-02	4.567	1077	0.927	0.834	1.030	0.796	0.1598
SOMA_Q6NUJ1_PSAPL1_8814_33	2234	0.883	0.833	0.936	3.98E-02	4.565	1077	0.644	0.548	0.756	7.050	<0.0001
SOMA_P05362_ICAM1_4342_10	2234	1.131	1.067	1.197	4.06E-02	4.556	1077	1.286	1.170	1.414	6.695	<0.0001
SOMA_Q9UN19_DAPP1_11615_16	2234	0.885	0.836	0.937	4.07E-02	4.554	1077	0.746	0.681	0.817	9.581	<0.0001
SOMA_P53779_MAPK10_15418_25	2234	0.886	0.837	0.938	4.09E-02	4.552	1077	0.812	0.724	0.911	3.398	0.0004
SOMA_P17535_JUND_19602_36	2234	1.131	1.068	1.198	4.13E-02	4.548	1077	1.068	1.002	1.140	1.353	0.0444
SOMA_Q96JN8_NEURL4_13468_5	2234	0.882	0.832	0.936	4.21E-02	4.539	1077	0.927	0.711	1.209	0.240	0.5757
SOMA_Q8IYS5_OSCAR_7116_31	2234	0.88	0.828	0.934	4.28E-02	4.532	1077	1.031	0.955	1.114	0.364	0.4322
SOMA_Q9BUP0_EFHD1_19616_100	2234	0.882	0.832	0.936	4.28E-02	4.532	1077	0.881	0.752	1.033	0.927	0.1183
SOMA_Q03692_COL10A1_15653_9	2234	1.13	1.067	1.197	4.31E-02	4.529	1077	1.033	0.978	1.092	0.614	0.2431
SOMA_Q9UBM4_OPTC_15430_165	2234	0.884	0.834	0.937	4.38E-02	4.522	1077	0.956	0.800	1.144	0.204	0.6253

SOMA_A4D1S0_KLRG2_10762_2	2234	0.882	0.831	0.936	4.56E-02	4.504	1077	1.034	0.946	1.130	0.331	0.4664
SOMA_P11487_FGF3_7894_155	2234	1.128	1.066	1.194	4.63E-02	4.497	1077	1.015	0.927	1.112	0.126	0.7487
SOMA_P43405_SYK_11378_37	2234	0.884	0.834	0.937	4.64E-02	4.496	1077	0.886	0.794	0.990	1.494	0.0321
SOMA_Q14435_GALNT3_6593_5	2234	1.128	1.066	1.193	4.65E-02	4.495	1077	1.071	1.010	1.136	1.668	0.0215
SOMA_O60476_MAN1A2_9077_10	2234	0.881	0.83	0.935	4.66E-02	4.494	1077	0.920	0.817	1.035	0.788	0.1628
SOMA_Q9HC16_APOBEC3_13930_3	2234	0.884	0.834	0.937	4.68E-02	4.492	1077	0.810	0.730	0.899	4.145	<0.0001
SOMA_P45985_MAP2K4_5242_37	2234	0.884	0.834	0.937	4.71E-02	4.489	1077	0.781	0.701	0.871	5.115	<0.0001
SOMA_Q02742_GCNT1_7016_12	2234	1.127	1.065	1.192	4.75E-02	4.486	1077	1.073	1.008	1.142	1.558	0.0277
SOMA_O43464_HTRA2_3317_33	2234	1.133	1.068	1.202	4.76E-02	4.485	1077	1.039	0.935	1.154	0.322	0.4769
SOMA_P33764_S100A3_15297_3	2234	0.895	0.849	0.943	4.77E-02	4.484	1077	1.112	1.047	1.180	3.278	0.0005
SOMA_P04040_CAT_3488_64	2234	0.881	0.83	0.935	4.81E-02	4.48	1077	0.638	0.548	0.743	8.148	<0.0001
SOMA_Q5T2D2_TREML2_5736_1	2234	0.881	0.83	0.935	4.86E-02	4.476	1077	0.928	0.828	1.040	0.705	0.1973
SOMA_O95881_TXNDC12_19334_62	2234	1.13	1.067	1.198	4.90E-02	4.472	1077	0.863	0.784	0.951	2.530	0.0029
SOMA_P34810_CD68_18922_27	2234	1.133	1.068	1.202	4.92E-02	4.47	1077	1.009	0.920	1.108	0.073	0.8454
SOMA_O14625_CXCL11_3038_9	2234	1.131	1.067	1.199	4.93E-02	4.469	1077	1.206	1.136	1.280	9.140	<0.0001

Table S4. Results from adjusted cox regression models for DHFA outcomes in the PHFS, replicated in the WashU cohort.

SOMA_ID	PHFS						WashU					
	N	sHR	Standardized.LB.95CI	Standardized.UB.95CI	Alpha.corrected.P.value	minus_log10_P	N	sHR	Standardized_LB_95CI	Standardized_UB_95CI	minus_log10_P	P_value
SOMA_Q86TH1_ADAMTSL_6379_62	1889	1.403	1.304	1.508	0	19.229	1077	1.534	1.436	1.639	36.308	<0.0001
SOMA_P11686_SFTPC_5738_25	1889	1.337	1.245	1.437	2.48E-12	14.768	NA	NA	NA	NA	NA	NA
SOMA_P35442_THBS2_3339_33	1889	1.386	1.277	1.504	7.93E-12	14.272	1077	1.635	1.533	1.744	50.083	<0.0001
SOMA_Q96GL9_FAM163A_6260_14	1889	1.318	1.228	1.416	4.55E-11	13.515	NA	NA	NA	NA	NA	NA
SOMA_Q16270_IGFBP7_3320_49	1889	1.336	1.239	1.441	8.84E-11	13.226	1077	1.681	1.560	1.812	41.634	<0.0001
SOMA_Q13332_PTPRS_6049_64	1889	0.789	0.739	0.841	6.84E-10	12.338	1077	0.890	0.802	0.989	1.513	0.0307
SOMA_P11597_CETP_7131_207	1889	1.295	1.206	1.39	1.29E-09	12.062	NA	NA	NA	NA	NA	NA
SOMA_Q9GZV9_FGF23_3807_1	1889	1.277	1.193	1.368	3.29E-09	11.655	1077	1.349	1.270	1.432	21.880	<0.0001
SOMA_P24821_TNC_4155_3	1889	1.281	1.195	1.373	3.78E-09	11.595	1077	1.689	1.568	1.820	42.713	<0.0001
SOMA_Q16627_CCL14_2900_53	1889	1.306	1.211	1.407	4.70E-09	11.5	1077	1.510	1.429	1.594	48.533	<0.0001
SOMA_Q9BWP8_COLEC11_4430_44	1889	1.255	1.174	1.342	3.65E-08	10.61	1077	1.264	1.190	1.343	13.507	<0.0001
SOMA_O94856_NFASC_7179_69	1889	1.258	1.175	1.347	5.60E-08	10.425	1077	1.264	1.194	1.337	15.256	<0.0001
SOMA_P51888_PRELP_5675_6	1889	1.267	1.18	1.36	8.80E-08	10.228	NA	NA	NA	NA	NA	NA
SOMA_Q92743_HTRA1_15594_47	1889	1.26	1.175	1.35	1.20E-07	10.095	1077	1.447	1.346	1.555	23.118	<0.0001
SOMA_Q9H4D0_CLSTN2_18882_7	1889	1.27	1.181	1.367	2.23E-07	9.825	1077	1.372	1.312	1.435	42.438	<0.0001

SOMA_Q9HB63_NTN4_3327_27	1889	1.247	1.162	1.338	1.15E-06	9.112	1077	1.317	1.255	1.382	28.665	<0.0001
SOMA_Q6P4A8_PLBD1_6315_58	1889	1.209	1.136	1.287	3.52E-06	8.627	1077	0.991	0.898	1.095	0.064	0.8638
SOMA_P05413_FABP3_5437_63	1889	1.218	1.14	1.301	6.44E-06	8.364	1077	1.325	1.239	1.417	15.616	<0.0001
SOMA_P58335_ANTXR2_15559_5	1889	0.823	0.771	0.879	8.66E-06	8.235	1077	0.601	0.534	0.677	16.452	<0.0001
SOMA_Q7Z3B1_NEGR1_7050_5	1889	0.827	0.776	0.882	8.80E-06	8.229	1077	1.028	0.948	1.115	0.299	0.5026
SOMA_P15907_ST6GAL1_6035_2	1889	1.21	1.134	1.29	1.22E-05	8.087	1077	1.070	0.993	1.152	1.131	0.074
SOMA_P55083_MFAP4_5636_10	1889	1.236	1.149	1.329	1.62E-05	7.962	1077	1.536	1.441	1.638	38.656	<0.0001
SOMA_Q8IUL8_CILP2_8841_65	1889	0.81	0.753	0.871	1.77E-05	7.924	1077	0.485	0.422	0.559	23.080	<0.0001
SOMA_P23468_PTPRD_9296_15	1889	0.832	0.78	0.886	1.86E-05	7.903	1077	0.761	0.679	0.852	5.610	<0.0001
SOMA_O15123_ANGPT2_2602_2	1889	1.279	1.174	1.392	2.27E-05	7.816	1077	1.748	1.629	1.877	52.925	<0.0001
SOMA_O00585_CCL21_2516_57	1889	1.197	1.124	1.274	3.29E-05	7.656	1077	1.364	1.277	1.457	19.626	<0.0001
SOMA_P01160_NPPA_5443_62	1889	1.254	1.158	1.358	4.00E-05	7.571	1077	1.667	1.553	1.789	44.572	<0.0001
SOMA_P06396_GSN_4775_34	1889	0.838	0.787	0.892	4.47E-05	7.522	1077	0.839	0.734	0.960	1.977	0.0105
SOMA_Q7Z3B1_NEGR1_13109_82	1889	0.841	0.79	0.894	5.06E-05	7.469	1077	1.087	0.990	1.193	1.090	0.0813
SOMA_Q9P121_NTM_10907_116	1889	0.837	0.785	0.892	5.73E-05	7.415	1077	0.927	0.837	1.028	0.820	0.1512
SOMA_Q13508_ART3_7970_315	1889	0.842	0.792	0.896	6.70E-05	7.347	1077	1.168	1.075	1.268	3.610	0.0002
SOMA_Q04609_FOLH1_5478_50	1889	1.188	1.116	1.264	8.36E-05	7.251	1077	1.111	1.047	1.180	3.298	0.0005
SOMA_P0C0P6_NPS_6390_18	1889	0.842	0.791	0.896	8.67E-05	7.235	1077	0.564	0.501	0.635	20.376	<0.0001
SOMA_Q6P988_NOTUM_8252_2	1889	0.814	0.756	0.877	8.89E-05	7.224	1077	0.388	0.316	0.476	18.767	<0.0001

SOMA_Q9H772_GREM2_5598_3	1889	1.223	1.136	1.316	0.000122	7.087	1077	1.224	1.171	1.279	18.343	<0.0001
SOMA_Q6UXZ4_UNC5D_16307_22	1889	0.843	0.792	0.897	0.000131	7.056	1077	1.084	0.992	1.185	1.122	0.0754
SOMA_Q03167_TGFBR3_3009_3	1889	0.838	0.786	0.894	0.000136	7.041	1077	1.260	1.156	1.373	6.875	<0.0001
SOMA_Q8N6G6_ADAMTSL_16890_37	1889	0.829	0.774	0.888	0.000144	7.014	1077	1.234	1.129	1.348	5.454	<0.0001
SOMA_Q9UBX5_FBLN5_15585_304	1889	1.225	1.137	1.32	0.000146	7.008	1077	1.849	1.711	1.999	53.404	<0.0001
SOMA_Q9NQ79_CRTAC1_5632_6	1889	0.848	0.798	0.901	0.000161	6.967	1077	0.641	0.568	0.722	12.410	<0.0001
SOMA_Q96PX8_SLITRK1_15539_15	1889	0.843	0.791	0.898	0.000181	6.915	1077	1.033	0.946	1.128	0.325	0.4733
SOMA_Q9BXJ1_C1QTNF1_6304_8	1889	1.214	1.13	1.305	2.00E-04	6.872	1077	1.338	1.261	1.419	21.457	<0.0001
SOMA_Q01973_ROR1_2590_69	1889	0.84	0.787	0.897	0.000257	6.763	1077	1.279	1.174	1.395	7.661	<0.0001
SOMA_Q09028_RBBP4_15331_47	1889	1.186	1.112	1.264	0.000275	6.733	1077	1.130	1.052	1.213	3.118	0.0008
SOMA_P54764_EPHA4_16288_17	1889	0.846	0.794	0.901	0.000287	6.714	1077	1.011	0.911	1.121	0.075	0.8416
SOMA_Q9BQ51_PDCD1LG_3004_67	1889	0.843	0.79	0.899	0.000288	6.714	1077	1.056	0.978	1.141	0.787	0.1634
SOMA_Q7LFX5_CHST15_4469_78	1889	1.209	1.126	1.299	0.00031	6.681	1077	1.499	1.400	1.605	30.315	<0.0001
SOMA_P01833_PIGR_3216_2	1889	1.186	1.112	1.265	0.000313	6.677	1077	1.182	1.114	1.253	7.527	<0.0001
SOMA_Q9HCB6_SPON1_4297_62	1889	1.233	1.138	1.336	0.000431	6.538	1077	1.457	1.379	1.540	39.881	<0.0001
SOMA_P52823_STC1_4930_21	1889	1.197	1.117	1.282	0.000462	6.508	1077	1.397	1.294	1.508	16.922	<0.0001
SOMA_P06396_GSN_16607_78	1889	0.846	0.793	0.902	0.000476	6.495	1077	0.756	0.685	0.834	7.620	<0.0001
SOMA_Q96EE4_CCDC126_6388_21	1889	0.842	0.788	0.9	0.000521	6.456	1077	0.294	0.182	0.477	6.185	<0.0001

SOMA_Q9BQT9_CLSTN3_6291_55	1889	1.207	1.123	1.298	0.000535	6.444	1077	1.281	1.205	1.362	14.792	<0.0001
SOMA_Q9H6X2_ANTXR1_10464_6	1889	0.852	0.801	0.906	0.000554	6.429	1077	0.501	0.379	0.663	5.890	<0.0001
SOMA_Q9BY76_ANGPTL4_3796_79	1889	1.181	1.108	1.26	0.000571	6.416	1077	1.235	1.168	1.306	12.802	<0.0001
SOMA_Q6UXM1_LRIG3_3322_52	1889	0.846	0.793	0.903	0.00064	6.367	1077	0.850	0.767	0.942	2.694	0.002
SOMA_P22607_FGFR3_13669_6	1889	0.851	0.8	0.906	0.000698	6.329	1077	0.933	0.842	1.035	0.722	0.1896
SOMA_P02741_CRP_4337_49	1889	1.182	1.108	1.262	0.000729	6.31	1077	1.563	1.414	1.728	17.656	<0.0001
SOMA_Q16623_STX1A_19553_14	1889	0.852	0.801	0.907	0.000783	6.279	1077	0.858	0.751	0.981	1.608	0.0246
SOMA_Q9Y240_CLEC11A_4500_50	1889	1.188	1.111	1.272	0.000867	6.235	1077	1.416	1.320	1.519	21.409	<0.0001
SOMA_P40394_ADH7_11377_19	1889	0.853	0.801	0.908	0.000905	6.216	1077	0.873	0.746	1.020	1.057	0.0877
SOMA_Q96AP7_ESAM_7841_84	1889	0.848	0.794	0.905	0.0011	6.13	1077	1.142	1.043	1.250	2.382	0.0041
SOMA_P02749_APOH_8288_27	1889	0.847	0.793	0.905	0.00112	6.123	1077	1.093	0.987	1.210	1.056	0.088
SOMA_Q9BYH1_SEZ6L_19563_3	1889	0.856	0.805	0.911	0.00122	6.087	1077	1.061	0.974	1.155	0.751	0.1775
SOMA_Q9UHD0_IL19_3035_80	1889	1.183	1.106	1.265	0.00131	6.054	1077	1.420	1.336	1.509	28.521	<0.0001
SOMA_Q8N3J6_CADM2_16907_3	1889	0.85	0.797	0.907	0.00147	6.004	1077	1.035	0.975	1.099	0.596	0.2535
SOMD P63098 PPP3CA 4903 72 FLAG	1889	0.852	0.8	0.909	0.00157	5.978	NA	NA	NA	NA	NA	NA
SOMD Q08209 PPP3CA 4903 72 FLAG	1889	0.852	0.8	0.909	0.00157	5.978	NA	NA	NA	NA	NA	NA
SOMA_O00622_CYR61_6264_9	1889	1.185	1.107	1.269	0.00173	5.934	NA	NA	NA	NA	NA	NA
SOMA_P08174_CD55_5069_9	1889	0.855	0.802	0.911	0.00187	5.9	1077	1.207	1.125	1.295	6.791	<0.0001

SOMA_Q4LDE5_SVEP1_11109_56	1889	1.229	1.13	1.337	0.00249	5.776	1077	1.441	1.363	1.524	37.274	<0.0001
SOMA_Q15768_EFNB3_2514_65	1889	1.171	1.098	1.25	0.00258	5.761	1077	1.135	1.073	1.199	5.105	<0.0001
SOMA_O95319_CELF2_7245_2	1889	1.165	1.094	1.241	0.0028	5.726	1077	1.117	1.041	1.198	2.690	0.002
SOMA_Q9Y240_CLEC11A_2966_65	1889	1.178	1.101	1.26	0.00285	5.718	1077	1.408	1.316	1.507	22.431	<0.0001
SOMA_O60760_HPGDS_12549_33	1889	0.855	0.801	0.913	0.00365	5.61	1077	0.539	0.459	0.634	13.159	<0.0001
SOMA_P08670_VIM_15540_6	1889	1.172	1.097	1.252	0.00419	5.549	1077	1.072	0.994	1.156	1.144	0.0718
SOMA_Q9Y4K0_LOXL2_6504_65	1889	1.165	1.093	1.242	0.00437	5.532	1077	1.125	1.069	1.183	5.246	<0.0001
SOMA_Q14512_FGFBP1_15494_11	1889	1.184	1.103	1.272	0.00468	5.502	1077	1.228	1.152	1.308	9.553	<0.0001
SOMA_A6NGN9_IGLON5_6478_2	1889	0.858	0.805	0.915	0.00478	5.492	1077	1.024	0.928	1.131	0.196	0.6361
SOMA_P19429_TNNI3_5441_67	1889	1.176	1.099	1.26	0.00501	5.472	1077	1.013	0.935	1.097	0.123	0.7528
SOMA_Q4LDE5_SVEP1_11178_21	1889	1.221	1.122	1.329	0.00568	5.417	1077	1.454	1.375	1.539	38.225	<0.0001
SOMA_Q9BRK3_MXRA8_10521_10	1889	0.865	0.813	0.92	0.00641	5.365	1077	0.993	0.898	1.099	0.047	0.8974
SOMA_Q9P121_NTM_8428_102	1889	0.863	0.811	0.919	0.00695	5.329	1077	0.842	0.755	0.940	2.676	0.0021
SOMA_Q9P2E7_PCDH10_9018_38	1889	0.856	0.801	0.915	0.00718	5.315	1077	1.056	0.978	1.141	0.784	0.1644
SOMA_O95633_FSTL3_3438_10	1889	1.214	1.117	1.32	0.00773	5.283	1077	1.489	1.401	1.583	36.451	<0.0001
SOMA_Q6UY11_DLK2_9359_9	1889	0.856	0.801	0.915	0.00782	5.278	1077	1.204	1.129	1.284	7.768	<0.0001
SOMA_P04070_PROC_2961_1	1889	0.852	0.795	0.914	0.00952	5.192	1077	0.678	0.611	0.753	12.380	<0.0001
SOMA_O00468_AGRN_15483_377	1889	1.16	1.087	1.237	0.0103	5.157	1077	1.218	1.112	1.334	4.687	<0.0001
SOMA_Q86Y29_BAGE3_6442_6	1889	0.869	0.817	0.924	0.0103	5.157	1077	0.723	0.649	0.805	8.463	<0.0001

SOMA_O14773_TPP1_17691_1	1889	1.156	1.085	1.231	0.0107	5.143	1077	1.038	0.972	1.109	0.575	0.2663
SOMA_Q9UBU2_DKK2_15678_71	1889	0.864	0.811	0.921	0.0107	5.14	1077	0.745	0.667	0.832	6.729	<0.0001
SOMA_P25311_AZGP1_9312_8	1889	0.865	0.812	0.922	0.0118	5.098	1077	1.202	1.093	1.320	3.865	0.0001
SOMA_Q9H0R8_GABARAP_12661_44	1889	1.182	1.098	1.272	0.0131	5.054	1077	1.058	1.011	1.108	1.813	0.0154
SOMA_Q8IZS8_CACNA2D_8885_6	1889	0.863	0.809	0.921	0.0131	5.054	1077	0.658	0.582	0.743	10.694	<0.0001
SOMA_Q8WXD2_SCG3_7957_2	1889	0.865	0.811	0.922	0.0136	5.038	1077	1.178	1.079	1.286	3.595	0.0003
SOMA_P28799_GRN_4992_49	1889	1.171	1.092	1.256	0.0144	5.012	1077	1.383	1.291	1.481	19.546	<0.0001
SOMA_Q8N257_HIST3H2_18823_52	1889	1.156	1.084	1.233	0.0145	5.008	NA	NA	NA	NA	NA	NA
SOMA_P01019_AGT_3484_60	1889	0.864	0.809	0.922	0.0145	5.007	1077	0.859	0.767	0.963	2.042	0.0091
SOMA_Q9NQ75_CASS4_12855_16	1889	0.865	0.811	0.923	0.015	4.995	1077	0.870	0.774	0.979	1.688	0.0205
SOMA_P14784_IL2RB_9343_16	1889	0.872	0.821	0.927	0.0179	4.916	1077	0.938	0.734	1.200	0.213	0.6118
SOMA_O95393_BMP10_3587_53	1889	1.16	1.085	1.239	0.0181	4.91	1077	1.229	1.154	1.309	9.859	<0.0001
SOMA_Q9UBX1_CTSF_9212_22	1889	0.867	0.813	0.925	0.0215	4.835	1077	0.703	0.621	0.796	7.592	<0.0001
SOMA_Q13017_ARHGAP5_14748_31	1889	0.876	0.826	0.93	0.0222	4.822	1077	0.809	0.697	0.939	2.267	0.0054
SOMA_Q9HCR9_PDE11A_5252_33	1889	0.87	0.817	0.927	0.0228	4.81	1077	0.915	0.828	1.013	1.064	0.0862
SOMA_P24592_IGFBP6_14088_38	1889	0.868	0.814	0.926	0.0233	4.801	1077	1.189	1.083	1.306	3.534	0.0003
SOMA_O95166_GABARAP_17735_130	1889	1.173	1.091	1.262	0.0244	4.78	1077	1.191	1.134	1.250	11.672	<0.0001
SOMA_Q8IW52_SLITRK4_7139_14	1889	0.865	0.809	0.924	0.0248	4.773	1077	1.330	1.236	1.431	13.591	<0.0001
SOMA_Q9BXJ4_C1QTNF3_7251_64	1889	0.869	0.815	0.926	0.026	4.753	1077	0.453	0.382	0.538	18.708	<0.0001

SOMA_Q9UNI1_CELA1_6107_3	1889	1.167	1.087	1.253	0.0269	4.736	1077	1.658	1.518	1.812	28.322	<0.0001
SOMA_P14210_HGF_2681_23	1889	1.155	1.081	1.234	0.0275	4.728	1077	1.212	1.138	1.290	8.735	<0.0001
SOMA_Q8TEU8_WFIKKN2_3235_50	1889	0.866	0.811	0.925	0.0279	4.721	1077	1.234	1.130	1.348	5.551	<0.0001
SOMA_O43916_CHST1_7803_4	1889	1.147	1.077	1.221	0.0287	4.708	1077	0.934	0.723	1.207	0.222	0.6003
SOMA_O15197_EPHB6_5078_82	1889	0.869	0.815	0.927	0.0294	4.697	1077	1.146	1.088	1.206	6.682	<0.0001
SOMA_P01011_SERPINA_4153_11	1889	1.166	1.086	1.252	0.0324	4.655	1077	1.309	1.246	1.376	25.468	<0.0001
SOMA_Q02818_NUCB1_10451_11	1889	1.15	1.078	1.227	0.0325	4.653	1077	1.123	1.079	1.169	7.811	<0.0001
SOMA_P25189_MPZ_10615_18	1889	0.874	0.821	0.93	0.0347	4.625	1077	0.765	0.495	1.183	0.641	0.2283
SOMA_Q96IZ0_PAWR_9565_6	1889	0.876	0.824	0.932	0.036	4.608	1077	0.898	0.804	1.004	1.236	0.058
SOMA_Q14195_DPYSL3_12707_26	1889	0.867	0.811	0.926	0.0364	4.603	1077	0.830	0.736	0.937	2.600	0.0025
SOMA_P02679_FGG_4989_7	1889	1.149	1.077	1.225	0.0378	4.587	1077	1.356	1.258	1.462	14.724	<0.0001
SOMA_Q7L7L0_HIST3H2_14144_3	1889	1.146	1.075	1.221	0.0378	4.587	NA	NA	NA	NA	NA	NA
SOMA_Q8NCW5_APOA1BP_16621_77	1889	0.869	0.815	0.928	0.0381	4.584	NA	NA	NA	NA	NA	NA
SOMA_P62995_TRA2B_12373_73	1889	1.156	1.081	1.237	0.0385	4.578	1077	1.144	1.094	1.196	8.596	<0.0001
SOMA_Q8WWZ8_OIT3_6296_36	1889	1.162	1.083	1.246	0.0405	4.557	1077	1.192	1.123	1.265	8.215	<0.0001
SOMA_Q53FT3_C11orf7_8686_342	1889	1.137	1.071	1.208	0.0425	4.535	NA	NA	NA	NA	NA	NA
SOMA_P07585_DCN_2666_53	1889	1.145	1.074	1.22	0.0451	4.509	1077	1.076	1.034	1.120	3.458	0.0003
SOMA_Q9UBI4_STOML1_17344_23	1889	0.874	0.819	0.931	0.0484	4.477	1077	0.466	0.354	0.615	7.190	<0.0001

Table S5. Cis- Mendelian randomization estimates for the associations between genetically predited protein levels using pQTLs from the deCODE cohort and DHFA as an outcome

UniProtID	Protein	aptamer_id	outcome	snps	ivw.OR	ivw.l	ivw.u	ivw.p	wm.OR	wm.l	wm.u	wm.p	egger.OR	egger.l	egger.u	egger.p	Egger.intercept.p	Q.p	I2	F.statistic	p.adj
Q96EE4	CCDC126	6388_21	DHFA	3	1.981	1.367	2.872	0.0003	1.754	1.197	2.570	0.004	1.029	0.233	4.537	0.976	0.492	0.025	72.94	406.197	0.026
P08174	CD55	5069_9	DHFA	3	0.664	0.483	0.913	0.0116	0.633	0.461	0.868	0.005	0.549	0.328	0.921	0.264	0.565	0.443	0.00	391.876	0.424
Q16627	CCL14	2900_53	DHFA	3	1.264	1.046	1.529	0.0153	1.256	1.010	1.561	0.041	1.336	0.950	1.879	0.344	0.733	0.455	0.00	1240.748	0.424
Q7Z3B1	NEGR1	13109_82	DHFA	2	0.470	0.225	0.983	0.0448	----	----	----	----	----	----	----	----	----	----	----	124.724	0.673
Q7Z3B1	NEGR1	7050_5	DHFA	2	0.470	0.225	0.983	0.0448	----	----	----	----	----	----	----	----	----	----	----	124.724	0.673
O95633	FSTL3	3438_10	DHFA	2	0.368	0.131	1.032	0.0575	----	----	----	----	----	----	----	----	----	----	----	52.908	0.673
Q9BXJ4	C1QTNF3	7251_64	DHFA	2	0.511	0.252	1.033	0.0616	----	----	----	----	----	----	----	----	----	----	----	137.169	0.673
Q6UXZ4	UNC5D	16307_22	DHFA	1	2.548	0.931	6.971	0.0686	----	----	----	----	----	----	----	----	----	----	----	148.043	0.673
Q6UXM1	LRIG3	3322_52	DHFA	5	0.770	0.578	1.025	0.0730	0.766	0.549	1.068	0.116	0.790	0.439	1.423	0.490	0.911	0.951	0.00	278.048	0.673
Q9UBX1	CTSF	9212_22	DHFA	1	0.623	0.350	1.108	0.1071	----	----	----	----	----	----	----	----	----	----	----	444.355	0.784
Q9HB63	NTN4	3327_27	DHFA	6	1.404	0.901	2.187	0.1337	1.549	0.955	2.512	0.076	1.990	0.883	4.487	0.172	0.372	0.680	0.00	136.248	0.784
Q9BYH1	SEZ6L	19563_3	DHFA	4	1.641	0.843	3.195	0.1451	1.767	0.820	3.811	0.146	2.686	0.561	12.860	0.342	0.569	0.772	0.00	76.758	0.784
P52823	STC1	4930_21	DHFA	2	1.972	0.785	4.951	0.1482	----	----	----	----	----	----	----	----	----	----	----	77.345	0.784
Q9H4D0	CLSTN2	18882_7	DHFA	12	1.217	0.926	1.598	0.1590	1.092	0.771	1.547	0.618	0.793	0.435	1.449	0.469	0.157	0.602	0.00	183.841	0.784
P58335	ANTXR2	15559_5	DHFA	5	0.732	0.471	1.136	0.1642	0.683	0.404	1.155	0.155	0.686	0.259	1.822	0.505	0.929	0.757	0.00	129.597	0.784
Q9NQ79	CRTAC1	5632_6	DHFA	5	1.185	0.932	1.506	0.1659	1.144	0.892	1.468	0.290	0.838	0.475	1.480	0.586	0.280	0.667	0.00	828.524	0.784

O00468	AGRN	15483_377	DHFA	4	0.792	0.558	1.125	0.1927	0.760	0.520	1.111	0.157	0.570	0.215	1.509	0.375	0.554	0.681	0.00	257.149	0.784
Q4LDE5	SVEP1	11109_56	DHFA	11	0.809	0.584	1.120	0.2009	0.828	0.551	1.244	0.364	0.689	0.257	1.842	0.476	0.743	0.890	0.00	143.880	0.784
Q4LDE5	SVEP1	11178_21	DHFA	11	0.809	0.584	1.120	0.2009	0.828	0.543	1.263	0.381	0.689	0.257	1.842	0.476	0.743	0.890	0.00	143.880	0.784
Q9H6X2	ANTXR1	10464_6	DHFA	1	3.707	0.470	29.209	0.2135	----	----	----	----	----	----	----	----	----	----	----	30.720	0.784
Q13332	PTPRS	6049_64	DHFA	2	2.441	0.598	9.961	0.2136	----	----	----	----	----	----	----	----	----	----	----	42.137	0.784
P04070	PROC	2961_1	DHFA	1	0.661	0.335	1.304	0.2321	----	----	----	----	----	----	----	----	----	----	----	315.084	0.784
P16860	NPPB	7655_11	DHFA	4	0.815	0.583	1.140	0.2323	0.827	0.593	1.153	0.262	0.696	0.358	1.355	0.398	0.673	0.851	0.00	357.377	0.784
P16860	NPPB	16751_15	DHFA	4	0.815	0.583	1.140	0.2323	0.827	0.586	1.166	0.278	0.696	0.358	1.355	0.398	0.673	0.851	0.00	357.377	0.784
Q9H772	GREM2	5598_3	DHFA	1	2.638	0.530	13.127	0.2360	----	----	----	----	----	----	----	----	----	----	----	29.996	0.784
O94856	NFASC	7179_69	DHFA	6	1.145	0.906	1.446	0.2581	1.182	0.926	1.509	0.180	1.263	0.804	1.982	0.368	0.623	0.685	0.00	364.222	0.816
Q9NQ75	CASS4	12855_16	DHFA	1	3.126	0.421	23.231	0.2654	----	----	----	----	----	----	----	----	----	----	----	49.988	0.816
Q8TEU8	WFIKKN2	3235_50	DHFA	8	1.113	0.915	1.355	0.2853	1.161	0.929	1.450	0.190	1.128	0.753	1.689	0.581	0.974	0.563	0.00	398.653	0.846
Q9BWP8	COLEC11	4430_44	DHFA	13	1.082	0.933	1.254	0.2987	1.096	0.921	1.305	0.301	1.058	0.850	1.316	0.625	0.821	0.972	0.00	755.874	0.855
P14784	IL2RB	9343_16	DHFA	1	0.622	0.226	1.709	0.3572	----	----	----	----	----	----	----	----	----	----	----	138.914	0.858
P14210	HGF	2681_23	DHFA	1	1.557	0.573	4.232	0.3852	----	----	----	----	----	----	----	----	----	----	----	158.506	0.858
P02741	CRP	4337_49	DHFA	3	1.279	0.716	2.285	0.4051	1.228	0.686	2.201	0.489	1.353	0.353	5.178	0.735	0.954	0.682	0.00	148.443	0.858
Q9Y240	CLEC11A	4500_50	DHFA	6	1.231	0.753	2.013	0.4074	1.435	0.817	2.521	0.208	2.391	0.845	6.761	0.176	0.228	0.383	5.31	96.590	0.858
Q9Y240	CLEC11A	2966_65	DHFA	6	1.231	0.753	2.013	0.4074	1.435	0.802	2.568	0.223	2.391	0.845	6.761	0.176	0.228	0.383	5.31	96.590	0.858
Q8NCW5	APOA1BP	16621_77	DHFA	2	0.839	0.552	1.274	0.4102	----	----	----	----	----	----	----	----	----	----	----	192.186	0.858

P54764	EPHA4	16288_17	DHFA	4	0.792	0.454	1.381	0.4110	0.783	0.414	1.479	0.451	0.316	0.046	2.159	0.361	0.426	0.737	0.00	115.407	0.858
Q7LFX5	CHST15	4469_78	DHFA	2	0.714	0.315	1.620	0.4200	----	----	----	----	----	----	----	----	----	----	----	98.055	0.858
P01019	AGT	3484_60	DHFA	5	1.194	0.776	1.835	0.4203	1.047	0.655	1.674	0.847	1.108	0.507	2.421	0.814	0.835	0.742	0.00	112.644	0.858
O00585	CCL21	2516_57	DHFA	3	0.734	0.343	1.568	0.4240	0.866	0.375	1.998	0.736	4.872	0.491	48.359	0.405	0.339	0.237	30.63	86.536	0.858
P01011	SERPINA	4153_11	DHFA	9	0.882	0.643	1.209	0.4351	0.913	0.625	1.333	0.637	0.797	0.475	1.336	0.417	0.656	0.847	0.00	167.086	0.858
P40394	ADH7	11377_19	DHFA	5	0.843	0.545	1.302	0.4404	0.796	0.542	1.169	0.245	0.547	0.305	0.981	0.136	0.141	0.341	11.31	215.386	0.858
Q9HCB6	SPON1	4297_62	DHFA	6	1.136	0.819	1.576	0.4439	1.152	0.816	1.626	0.423	1.276	0.669	2.436	0.500	0.729	0.816	0.00	245.511	0.858
Q86TH1	ADAMTSL	6379_62	DHFA	5	0.783	0.403	1.524	0.4722	0.691	0.327	1.460	0.333	0.170	0.018	1.624	0.221	0.251	0.509	0.00	71.495	0.858
P08670	VIM	15540_6	DHFA	1	2.080	0.281	15.388	0.4732	----	----	----	----	----	----	----	----	----	----	----	42.214	0.858
Q8WXD2	SCG3	7957_2	DHFA	2	0.907	0.689	1.194	0.4882	----	----	----	----	----	----	----	----	----	----	----	1335.886	0.858
Q9P2E7	PCDH10	9018_38	DHFA	1	1.283	0.613	2.688	0.5083	----	----	----	----	----	----	----	----	----	----	----	279.636	0.858
P06396	GSN	4775_34	DHFA	2	1.141	0.770	1.691	0.5108	----	----	----	----	----	----	----	----	----	----	----	222.152	0.858
P06396	GSN	16607_78	DHFA	2	1.141	0.770	1.691	0.5108	----	----	----	----	----	----	----	----	----	----	----	222.152	0.858
Q8N6G6	ADAMTSL	16890_37	DHFA	9	1.094	0.835	1.432	0.5147	1.197	0.885	1.619	0.243	0.979	0.660	1.453	0.920	0.394	0.644	0.00	216.845	0.858
Q9P121	NTM	10907_116	DHFA	16	1.075	0.851	1.359	0.5441	0.994	0.775	1.274	0.961	0.873	0.603	1.265	0.486	0.259	0.200	22.33	234.931	0.858
Q9P121	NTM	8428_102	DHFA	16	1.075	0.851	1.359	0.5441	0.994	0.778	1.270	0.960	0.873	0.603	1.265	0.486	0.259	0.200	22.33	234.931	0.858
P35442	THBS2	3339_33	DHFA	12	0.927	0.727	1.184	0.5450	0.935	0.718	1.218	0.619	0.946	0.590	1.518	0.823	0.966	0.179	27.09	339.267	0.858
P55083	MFAP4	5636_10	DHFA	2	1.289	0.547	3.037	0.5608	----	----	----	----	----	----	----	----	----	----	----	113.580	0.858
Q6UY11	DLK2	9359_9	DHFA	3	1.171	0.671	2.043	0.5791	1.059	0.598	1.876	0.845	0.403	0.109	1.486	0.402	0.312	0.050	66.66	162.745	0.858

Q8IUL8	CILP2	8841_65	DHFA	2	1.286	0.510	3.241	0.5935	----	----	----	----	----	----	----	----	----	----	----	71.239	0.858
P28799	GRN	4992_49	DHFA	2	1.124	0.731	1.729	0.5935	----	----	----	----	----	----	----	----	----	----	----	295.011	0.858
P22607	FGFR3	13669_6	DHFA	4	0.869	0.515	1.465	0.5977	0.825	0.485	1.402	0.477	0.224	0.013	3.967	0.415	0.441	0.410	0.00	165.965	0.858
Q9GZV9	FGF23	3807_1	DHFA	1	0.592	0.078	4.501	0.6122	----	----	----	----	----	----	----	----	----	----	----	39.598	0.858
P02679	FGG	4989_7	DHFA	1	1.190	0.586	2.418	0.6307	----	----	----	----	----	----	----	----	----	----	----	240.630	0.858
Q8IZS8	CACNA2D	8885_6	DHFA	13	0.911	0.620	1.337	0.6330	0.845	0.559	1.276	0.422	0.523	0.264	1.038	0.091	0.097	0.154	28.91	117.913	0.858
Q6P988	NOTUM	8252_2	DHFA	3	1.192	0.571	2.488	0.6401	1.136	0.532	2.426	0.742	1.390	0.242	7.991	0.775	0.875	0.537	0.00	128.839	0.858
P25311	AZGP1	9312_8	DHFA	1	1.128	0.680	1.874	0.6407	----	----	----	----	----	----	----	----	----	----	----	719.883	0.858
Q02818	NUCB1	10451_11	DHFA	2	0.872	0.439	1.732	0.6950	----	----	----	----	----	----	----	----	----	----	----	148.659	0.916
O15123	ANGPT2	2602_2	DHFA	2	1.133	0.531	2.420	0.7468	----	----	----	----	----	----	----	----	----	----	----	127.787	0.941
Q96AP7	ESAM	7841_84	DHFA	6	1.072	0.689	1.666	0.7589	1.171	0.720	1.904	0.525	1.213	0.541	2.719	0.663	0.721	0.939	0.00	118.335	0.941
Q96PX8	SLITRK1	15539_15	DHFA	1	0.788	0.169	3.672	0.7617	----	----	----	----	----	----	----	----	----	----	----	55.950	0.941
Q9UBX5	FBLN5	15585_304	DHFA	2	0.861	0.319	2.320	0.7672	----	----	----	----	----	----	----	----	----	----	----	64.952	0.941
Q13508	ART3	7970_315	DHFA	5	0.943	0.636	1.398	0.7708	0.967	0.666	1.403	0.859	1.375	0.595	3.178	0.510	0.413	0.310	16.43	255.593	0.941
Q04609	FOLH1	5478_50	DHFA	2	1.098	0.565	2.135	0.7833	----	----	----	----	----	----	----	----	----	----	----	160.416	0.942
Q03167	TGFBR3	3009_3	DHFA	3	0.915	0.406	2.061	0.8305	1.453	0.547	3.855	0.453	4.306	0.032	582.904	0.664	0.646	0.154	46.47	66.131	0.971
Q9BQ51	PDCD1LG	3004_67	DHFA	7	1.029	0.757	1.397	0.8566	1.137	0.813	1.591	0.453	0.972	0.517	1.826	0.932	0.839	0.762	0.00	220.270	0.971
P24821	TNC	4155_3	DHFA	8	0.984	0.788	1.228	0.8847	0.941	0.766	1.157	0.566	0.936	0.560	1.562	0.807	0.970	0.109	40.45	1130.013	0.971
Q14512	FGFBP1	15494_11	DHFA	1	0.920	0.291	2.914	0.8878	----	----	----	----	----	----	----	----	----	----	----	78.625	0.971

P02749	APOH	8288_27	DHFA	3	1.038	0.616	1.747	0.8889	1.105	0.620	1.968	0.734	2.029	0.240	17.189	0.633	0.628	0.241	29.66	166.342	0.971
P01833	PIGR	3216_2	DHFA	1	1.050	0.522	2.114	0.8910	----	----	----	----	----	----	----	----	----	----	----	287.048	0.971
Q92743	HTRA1	15594_47	DHFA	2	0.943	0.356	2.498	0.9063	----	----	----	----	----	----	----	----	----	----	----	95.601	0.971
O15197	EPHB6	5078_82	DHFA	3	1.025	0.662	1.587	0.9124	1.061	0.667	1.689	0.801	1.282	0.595	2.765	0.640	0.605	0.742	0.00	247.511	0.971
Q16270	IGFBP7	3320_49	DHFA	6	0.983	0.722	1.339	0.9128	0.971	0.698	1.351	0.862	1.118	0.568	2.202	0.763	0.670	0.924	0.00	253.280	0.971
O14773	TPP1	17691_1	DHFA	3	1.027	0.556	1.895	0.9328	1.067	0.569	1.999	0.840	1.782	0.084	37.597	0.774	0.778	0.895	0.00	111.356	0.977
O60760	HPGDS	12549_33	DHFA	3	0.992	0.761	1.293	0.9544	0.971	0.745	1.265	0.826	1.025	0.368	2.860	0.969	0.953	0.669	0.00	1112.143	0.977
P24592	IGFBP6	14088_38	DHFA	1	0.966	0.243	3.847	0.9607	----	----	----	----	----	----	----	----	----	----	----	62.462	0.977
A6NGN9	IGLON5	6478_2	DHFA	2	1.014	0.416	2.472	0.9755	----	----	----	----	----	----	----	----	----	----	----	91.119	0.977
Q01973	ROR1	2590_69	DHFA	5	0.994	0.683	1.449	0.9766	0.927	0.612	1.402	0.719	0.571	0.276	1.184	0.229	0.182	0.406	0.00	226.732	0.977

Table S6. Cis- Mendelian randomization estimates for the associations between genetically predited protein levels using pQTLs from the Fenland cohort and DHFA as an outcome

UniProtID	Protein	SomaScan.id	outcome	snps	ivw.OR	ivw.l	ivw.u	ivw.p	wm.OR	wm.l	wm.u	wm.p	egger.OR	egger.l	egger.u	egger.p	Egger.intercept.p	Q.p	I2	F.statistic	p.adjd
Q96EE4	CCDC126	SeqId_6388_21	DHFA	1	1.463	1.070	2.001	0.017	----	----	----	----	----	----	----	----	----	----	----	628.338	0.549
Q4LDE5	SVEP1	SeqId_11109_56	DHFA	3	0.575	0.363	0.910	0.018	0.573	0.346	0.951	0.031	0.537	0.106	2.724	0.590	0.946	0.897	0.000	92.583	0.549
P08174	CD55	SeqId_5069_9	DHFA	1	0.811	0.669	0.985	0.034	----	----	----	----	----	----	----	----	----	----	----	1334.684	0.549
P40394	ADH7	SeqId_11377_19	DHFA	1	0.665	0.446	0.993	0.046	----	----	----	----	----	----	----	----	----	----	----	454.465	0.549
Q9BXJ4	C1QTNF3	SeqId_7251_64	DHFA	1	0.628	0.385	1.023	0.062	----	----	----	----	----	----	----	----	----	----	----	223.424	0.549
Q6UXM1	LRIG3	SeqId_3322_52	DHFA	1	0.487	0.228	1.040	0.063	----	----	----	----	----	----	----	----	----	----	----	109.551	0.549
P14784	IL2RB	SeqId_9343_16	DHFA	1	0.397	0.147	1.077	0.070	----	----	----	----	----	----	----	----	----	----	----	55.675	0.549
O00468	AGRN	SeqId_15483_377	DHFA	1	0.755	0.546	1.044	0.089	----	----	----	----	----	----	----	----	----	----	----	460.818	0.549
Q7Z3B1	NEGR1	SeqId_7050_5	DHFA	1	0.595	0.322	1.098	0.097	----	----	----	----	----	----	----	----	----	----	----	149.592	0.549
Q7Z3B1	NEGR1	SeqId_13109_82	DHFA	1	0.651	0.390	1.088	0.101	----	----	----	----	----	----	----	----	----	----	----	209.136	0.549
Q16627	CCL14	SeqId_2900_53	DHFA	1	1.116	0.975	1.278	0.111	----	----	----	----	----	----	----	----	----	----	----	3428.753	0.549
P58335	ANTXR2	SeqId_15559_5	DHFA	2	0.755	0.532	1.070	0.114	----	----	----	----	----	----	----	----	----	----	----	207.014	0.549
Q4LDE5	SVEP1	SeqId_11178_21	DHFA	1	0.567	0.279	1.152	0.116	----	----	----	----	----	----	----	----	----	----	----	126.563	0.549
Q13508	ART3	SeqId_7970_315	DHFA	1	1.450	0.897	2.344	0.129	----	----	----	----	----	----	----	----	----	----	----	250.972	0.549
Q9HB63	NTN4	SeqId_3327_27	DHFA	2	1.355	0.914	2.008	0.131	----	----	----	----	----	----	----	----	----	----	----	212.673	0.549
Q96AP7	ESAM	SeqId_7841_84	DHFA	1	1.282	0.908	1.808	0.158	----	----	----	----	----	----	----	----	----	----	----	525.008	0.621

P04070	PROC	SeqId_2961_1	DHFA	1	0.659	0.357	1.215	0.182	----	----	----	----	----	----	----	----	----	----	----	145.852	0.671
Q9NQ79	CRTAC1	SeqId_5632_6	DHFA	4	1.142	0.935	1.394	0.192	1.120	0.910	1.379	0.284	0.933	0.593	1.468	0.793	0.433	0.414	0.000	362.835	0.671
O94856	NFASC	SeqId_7179_69	DHFA	1	1.139	0.931	1.393	0.206	----	----	----	----	----	----	----	----	----	----	----	1195.184	0.671
Q9UBX1	CTSF	SeqId_9212_22	DHFA	1	0.756	0.485	1.177	0.215	----	----	----	----	----	----	----	----	----	----	----	309.760	0.671
Q86TH1	ADAMTSL	SeqId_6379_62	DHFA	1	0.542	0.202	1.454	0.224	----	----	----	----	----	----	----	----	----	----	----	49.000	0.671
Q6P988	NOTUM	SeqId_8252_2	DHFA	1	0.758	0.455	1.263	0.288	----	----	----	----	----	----	----	----	----	----	----	136.819	0.763
Q9BWP8	COLEC11	SeqId_4430_44	DHFA	3	1.073	0.938	1.228	0.302	1.075	0.936	1.234	0.307	1.119	0.818	1.529	0.610	0.813	0.443	0.000	1070.781	0.763
P52823	STC1	SeqId_4930_21	DHFA	1	1.615	0.641	4.069	0.309	----	----	----	----	----	----	----	----	----	----	----	89.114	0.763
Q02818	NUCB1	SeqId_10451_11	DHFA	1	1.464	0.698	3.070	0.313	----	----	----	----	----	----	----	----	----	----	----	100.000	0.763
Q8WXD2	SCG3	SeqId_7957_2	DHFA	1	0.894	0.719	1.112	0.315	----	----	----	----	----	----	----	----	----	----	----	1438.577	0.763
Q9UBX5	FBLN5	SeqId_15585_304	DHFA	1	1.570	0.621	3.971	0.341	----	----	----	----	----	----	----	----	----	----	----	55.184	0.768
Q03167	TGFBR3	SeqId_3009_3	DHFA	1	1.559	0.618	3.930	0.347	----	----	----	----	----	----	----	----	----	----	----	61.035	0.768
P16860	NPPB	SeqId_7655_11	DHFA	1	0.827	0.548	1.248	0.365	----	----	----	----	----	----	----	----	----	----	----	332.361	0.768
P02741	CRP	SeqId_4337_49	DHFA	1	1.299	0.727	2.321	0.378	----	----	----	----	----	----	----	----	----	----	----	161.653	0.768
Q8IZS8	CACNA2D	SeqId_8885_6	DHFA	2	0.815	0.517	1.284	0.378	----	----	----	----	----	----	----	----	----	----	----	139.947	0.768
O60760	HPGDS	SeqId_12549_33	DHFA	1	1.108	0.875	1.403	0.395	----	----	----	----	----	----	----	----	----	----	----	1068.787	0.778
Q01973	ROR1	SeqId_2590_69	DHFA	1	0.782	0.436	1.401	0.408	----	----	----	----	----	----	----	----	----	----	----	169.000	0.779
Q16270	IGFBP7	SeqId_3320_49	DHFA	1	0.897	0.680	1.184	0.444	----	----	----	----	----	----	----	----	----	----	----	722.266	0.802
Q6UY11	DLK2	SeqId_9359_9	DHFA	1	1.278	0.680	2.400	0.446	----	----	----	----	----	----	----	----	----	----	----	139.240	0.802

Q9P121	NTM	SeqId_10907_116	DHFA	2	1.140	0.793	1.638	0.480	----	----	----	----	----	----	----	----	----	----	----	267.101	0.810
Q8NCW5	APOA1BP	SeqId_16621_77	DHFA	1	0.878	0.609	1.266	0.488	----	----	----	----	----	----	----	----	----	----	----	316.840	0.810
Q9P121	NTM	SeqId_8428_102	DHFA	2	1.077	0.872	1.330	0.493	----	----	----	----	----	----	----	----	----	----	----	788.125	0.810
Q8TEU8	WFIKKN2	SeqId_3235_50	DHFA	2	1.104	0.828	1.472	0.502	----	----	----	----	----	----	----	----	----	----	----	845.539	0.810
Q9H4D0	CLSTN2	SeqId_18882_7	DHFA	4	1.091	0.814	1.462	0.561	1.045	0.749	1.457	0.796	0.933	0.514	1.696	0.842	0.617	0.816	0.000	141.186	0.876
P01019	AGT	SeqId_3484_60	DHFA	2	1.108	0.770	1.593	0.582	----	----	----	----	----	----	----	----	----	----	----	191.910	0.876
P28799	GRN	SeqId_4992_49	DHFA	1	1.125	0.737	1.717	0.585	----	----	----	----	----	----	----	----	----	----	----	266.778	0.876
Q8N6G6	ADAMTSL	SeqId_16890_37	DHFA	2	1.111	0.745	1.657	0.606	----	----	----	----	----	----	----	----	----	----	----	134.436	0.876
Q92743	HTRA1	SeqId_15594_47	DHFA	2	0.759	0.257	2.243	0.618	----	----	----	----	----	----	----	----	----	----	----	89.057	0.876
P01833	PIGR	SeqId_3216_2	DHFA	1	1.154	0.639	2.084	0.636	----	----	----	----	----	----	----	----	----	----	----	169.000	0.876
Q9NQ75	CASS4	SeqId_12855_16	DHFA	1	1.174	0.588	2.343	0.650	----	----	----	----	----	----	----	----	----	----	----	100.000	0.876
Q9BQ51	PDCD1LG	SeqId_3004_67	DHFA	1	1.052	0.843	1.314	0.654	----	----	----	----	----	----	----	----	----	----	----	1230.005	0.876
P22607	FGFR3	SeqId_13669_6	DHFA	1	0.831	0.341	2.028	0.685	----	----	----	----	----	----	----	----	----	----	----	74.225	0.884
P54764	EPHA4	SeqId_16288_17	DHFA	2	1.116	0.650	1.917	0.691	----	----	----	----	----	----	----	----	----	----	----	96.263	0.884
Q7LFX5	CHST15	SeqId_4469_78	DHFA	1	1.144	0.574	2.280	0.701	----	----	----	----	----	----	----	----	----	----	----	97.516	0.884
O15123	ANGPT2	SeqId_2602_2	DHFA	1	1.198	0.418	3.430	0.736	----	----	----	----	----	----	----	----	----	----	----	54.128	0.907
P25311	AZGP1	SeqId_9312_8	DHFA	1	1.048	0.785	1.400	0.749	----	----	----	----	----	----	----	----	----	----	----	768.082	0.907
P02679	FGG	SeqId_4989_7	DHFA	1	1.104	0.549	2.220	0.780	----	----	----	----	----	----	----	----	----	----	----	115.879	0.918
P24821	TNC	SeqId_4155_3	DHFA	1	1.015	0.911	1.132	0.787	----	----	----	----	----	----	----	----	----	----	----	8503.474	0.918

P01011	SERPINA	SeqId_4153_11	DHFA	3	0.973	0.754	1.254	0.831	0.982	0.753	1.281	0.896	1.024	0.701	1.497	0.921	0.780	0.692	0.000	336.270	0.931
O00585	CCL21	SeqId_2516_57	DHFA	1	0.887	0.288	2.732	0.834	----	----	----	----	----	----	----	----	----	----	----	44.444	0.931
P55083	MFAP4	SeqId_5636_10	DHFA	1	1.087	0.477	2.479	0.843	----	----	----	----	----	----	----	----	----	----	----	80.003	0.931
Q9BRK3	MXRA8	SeqId_10521_10	DHFA	1	0.944	0.479	1.862	0.868	----	----	----	----	----	----	----	----	----	----	----	85.734	0.943
P08670	VIM	SeqId_15540_6	DHFA	2	1.074	0.390	2.958	0.890	----	----	----	----	----	----	----	----	----	----	----	102.495	0.951
Q9HCB6	SPON1	SeqId_4297_62	DHFA	2	1.029	0.628	1.687	0.909	----	----	----	----	----	----	----	----	----	----	----	484.503	0.951
O15197	EPHB6	SeqId_5078_82	DHFA	1	1.042	0.459	2.366	0.922	----	----	----	----	----	----	----	----	----	----	----	55.992	0.951
P06396	GSN	SeqId_4775_34	DHFA	1	1.027	0.539	1.954	0.936	----	----	----	----	----	----	----	----	----	----	----	133.691	0.951
P35442	THBS2	SeqId_3339_33	DHFA	2	1.003	0.829	1.214	0.972	----	----	----	----	----	----	----	----	----	----	----	841.976	0.972

Table S7. Mendelian randomization estimates for the associations between genetically predited protein levels using pQTLs from the deCODE cohort and DHFA as an outcome

UniProtID	Protein	aptamer_id	outcome	snps	ivw.OR	ivw.l	ivw.u	ivw.p	wm.OR	wm.l	wm.u	wm.p	egger.OR	egger.l	egger.u	egger.p	Egger.intercept.p	Q.p	I2	F.statistic	p.adjd
P08174	CD55	5069_9	DHFA	14	0.679	0.515	0.896	0.006	0.655	0.483	0.888	0.006	0.673	0.445	1.016	0.084	0.902	0.776	0.00	131.08	0.711
Q16627	CCL14	2900_53	DHFA	14	1.249	1.050	1.485	0.012	1.189	0.948	1.491	0.135	1.290	1.030	1.615	0.046	0.470	0.897	0.00	299.33	0.711
Q14195	DPYSL3	12707_26	DHFA	3	0.385	0.170	0.872	0.022	0.384	0.153	0.967	0.042	0.905	0.039	21.060	0.961	0.675	0.855	0.00	67.25	0.711
P54764	EPHA4	16288_17	DHFA	23	0.820	0.683	0.985	0.034	0.793	0.633	0.995	0.045	0.787	0.618	1.002	0.065	0.535	0.986	0.00	163.16	0.711
P58335	ANTXR2	15559_5	DHFA	16	0.718	0.528	0.977	0.035	0.716	0.461	1.110	0.135	0.770	0.358	1.657	0.514	0.822	0.595	0.00	83.38	0.711
Q7LFX5	CHST15	4469_78	DHFA	16	0.755	0.570	0.998	0.049	0.579	0.416	0.808	0.001	0.591	0.351	0.997	0.069	0.281	0.124	30.01	124.97	0.711
Q96EE4	CCDC126	6388_21	DHFA	24	1.320	0.995	1.750	0.054	1.525	1.131	2.056	0.006	1.507	0.937	2.423	0.105	0.556	0.114	26.65	118.72	0.711
O43916	CHST1	7803_4	DHFA	2	0.222	0.047	1.038	0.056	----	----	----	----	----	----	----	----	----	----	----	40.60	0.711
Q7Z3B1	NEGR1	7050_5	DHFA	8	0.603	0.359	1.015	0.057	0.622	0.317	1.219	0.167	0.387	0.086	1.732	0.261	0.551	0.908	0.00	60.91	0.711
Q7Z3B1	NEGR1	13109_82	DHFA	14	0.677	0.451	1.015	0.059	0.539	0.306	0.949	0.032	0.945	0.279	3.196	0.929	0.561	0.617	0.00	59.21	0.711
Q4LDE5	SVEP1	11109_56	DHFA	43	0.838	0.694	1.013	0.068	0.840	0.634	1.114	0.226	1.046	0.696	1.570	0.831	0.297	0.627	0.00	84.63	0.739
O00468	AGRN	15483_377	DHFA	20	0.817	0.651	1.027	0.083	0.716	0.522	0.983	0.039	0.617	0.402	0.946	0.040	0.159	0.747	0.00	114.78	0.832
O95633	FSTL3	3438_10	DHFA	3	0.455	0.182	1.138	0.092	0.597	0.200	1.784	0.356	0.532	0.000	4126.085	0.913	0.983	0.287	19.94	48.57	0.837
Q96IZ0	PAWR	9565_6	DHFA	6	0.551	0.271	1.123	0.101	0.613	0.238	1.578	0.310	3.872	0.020	745.902	0.641	0.502	0.432	0.00	47.14	0.837
Q03167	TGFBR3	3009_3	DHFA	15	0.717	0.480	1.072	0.105	1.022	0.595	1.753	0.938	0.869	0.289	2.612	0.807	0.737	0.240	19.14	63.62	0.837
Q8N3J6	CADM2	16907_3	DHFA	4	0.343	0.089	1.324	0.120	0.529	0.166	1.680	0.280	0.049	0.000	4253.859	0.656	0.770	0.123	48.03	41.03	0.904

Q8WWZ8	OIT3	6296_36	DHFA	3	2.409	0.743	7.809	0.143	3.205	0.726	14.149	0.124	0.178	0.001	46.986	0.653	0.522	0.646	0.00	39.59	0.983
Q16623	STX1A	19553_14	DHFA	8	0.677	0.391	1.171	0.163	0.648	0.350	1.200	0.167	0.480	0.135	1.706	0.300	0.561	0.253	22.12	91.82	0.983
P02741	CRP	4337_49	DHFA	18	0.828	0.628	1.092	0.182	0.972	0.668	1.414	0.882	0.740	0.405	1.354	0.343	0.568	0.262	16.00	120.64	0.983
Q9Y240	CLEC11A	4500_50	DHFA	29	1.192	0.921	1.544	0.182	1.304	0.875	1.944	0.193	1.742	0.868	3.497	0.130	0.287	0.797	0.00	59.14	0.983
Q92743	HTRA1	15594_47	DHFA	8	0.597	0.279	1.275	0.183	0.540	0.241	1.210	0.134	0.503	0.061	4.184	0.549	0.855	0.117	39.30	52.20	0.983
Q8IZS8	CACNA2D	8885_6	DHFA	30	0.830	0.631	1.092	0.183	0.778	0.577	1.051	0.101	0.669	0.416	1.074	0.107	0.323	0.171	19.62	97.67	0.983
Q6UXM1	LRIG3	3322_52	DHFA	49	0.905	0.774	1.059	0.212	0.853	0.675	1.077	0.181	0.899	0.677	1.194	0.465	0.840	0.277	9.98	114.64	0.983
Q13332	PTPRS	6049_64	DHFA	7	1.706	0.697	4.176	0.242	1.440	0.616	3.371	0.400	4.946	0.353	69.330	0.289	0.407	0.047	52.89	55.11	0.983
P0C0P6	NPS	6390_18	DHFA	4	0.690	0.364	1.309	0.256	0.925	0.483	1.772	0.815	0.409	0.103	1.627	0.332	0.480	0.291	19.72	89.37	0.983
Q9BXJ4	C1QTNF3	7251_64	DHFA	7	0.726	0.415	1.270	0.262	0.610	0.316	1.180	0.142	0.357	0.116	1.096	0.132	0.208	0.470	0.00	65.02	0.983
Q9HCB6	SPON1	4297_62	DHFA	15	1.174	0.885	1.557	0.266	1.153	0.812	1.637	0.428	1.180	0.695	2.002	0.550	0.977	0.901	0.00	125.07	0.983
O94856	NFASC	7179_69	DHFA	12	1.159	0.884	1.520	0.284	1.184	0.918	1.527	0.193	1.083	0.730	1.607	0.700	0.777	0.107	35.36	205.01	0.983
Q9BYH1	SEZ6L	19563_3	DHFA	6	1.353	0.751	2.437	0.313	1.371	0.689	2.726	0.369	2.985	0.744	11.969	0.198	0.293	0.763	0.00	66.61	0.983
Q96PX8	SLITRK1	15539_15	DHFA	4	0.626	0.248	1.583	0.323	0.775	0.261	2.303	0.646	1.154	0.009	156.589	0.960	0.827	0.743	0.00	43.32	0.983
P62995	TRA2B	12373_73	DHFA	2	2.034	0.489	8.458	0.329	----	----	----	----	----	----	----	----	----	----	----	45.17	0.983
Q9HCR9	PDE11A	5252_33	DHFA	3	1.756	0.565	5.453	0.330	1.538	0.394	6.010	0.536	66.936	0.000	40110491.150	0.647	0.683	0.177	42.27	42.33	0.983
O14773	TPP1	17691_1	DHFA	11	1.213	0.819	1.795	0.335	1.303	0.800	2.122	0.287	1.426	0.499	4.074	0.525	0.760	0.513	0.00	79.29	0.983
Q86Y29	BAGE3	6442_6	DHFA	7	0.788	0.477	1.301	0.351	1.061	0.567	1.985	0.853	0.468	0.167	1.311	0.208	0.310	0.586	0.00	67.49	0.983
P15907	ST6GAL1	6035_2	DHFA	1	2.850	0.311	26.130	0.354	----	----	----	----	----	----	----	----	----	----	----	29.90	0.983

P08670	VIM	15540_6	DHFA	5	0.719	0.358	1.445	0.354	0.510	0.219	1.188	0.119	0.131	0.007	2.320	0.260	0.315	0.428	0.00	63.31	0.983
O00585	CCL21	2516_57	DHFA	48	1.099	0.894	1.352	0.371	1.273	0.937	1.729	0.122	1.301	0.915	1.849	0.150	0.314	0.126	19.31	88.69	0.983
Q9H772	GREM2	5598_3	DHFA	15	1.171	0.827	1.659	0.375	0.934	0.640	1.362	0.721	0.645	0.399	1.044	0.098	0.011	0.053	40.27	115.28	0.983
P01011	SERPINA	4153_11	DHFA	11	0.875	0.647	1.182	0.383	0.906	0.623	1.318	0.606	0.793	0.486	1.293	0.376	0.647	0.931	0.00	144.95	0.983
P16860	NPPB	16751_15	DHFA	1	0.620	0.211	1.819	0.384	----	----	----	----	----	----	----	----	----	----	----	126.64	0.983
P19429	TNNI3	5441_67	DHFA	1	2.449	0.326	18.390	0.384	----	----	----	----	----	----	----	----	----	----	----	38.50	0.983
P01160	NPPA	5443_62	DHFA	5	1.219	0.770	1.931	0.399	1.293	0.787	2.123	0.311	1.399	0.621	3.152	0.477	0.773	0.868	0.00	143.13	0.983
O95166	GABARAP	17735_130	DHFA	3	0.682	0.280	1.664	0.401	0.943	0.355	2.506	0.906	31.445	0.275	3593.376	0.389	0.353	0.211	35.76	63.67	0.983
Q6UXZ4	UNC5D	16307_22	DHFA	5	1.436	0.616	3.344	0.402	1.526	0.640	3.637	0.340	22.936	1.272	413.740	0.124	0.144	0.233	28.26	64.39	0.983
Q6P988	NOTUM	8252_2	DHFA	9	1.234	0.753	2.022	0.403	1.041	0.561	1.933	0.899	1.428	0.499	4.089	0.528	0.692	0.634	0.00	80.77	0.983
Q9Y240	CLEC11A	2966_65	DHFA	27	1.127	0.851	1.492	0.405	1.227	0.803	1.875	0.345	1.918	0.897	4.100	0.105	0.183	0.642	0.00	57.44	0.983
Q8NCW5	APOA1BP	16621_77	DHFA	2	0.839	0.552	1.274	0.410	----	----	----	----	----	----	----	----	----	----	----	192.19	0.983
Q9UHD0	IL19	3035_80	DHFA	33	1.065	0.915	1.238	0.416	1.076	0.883	1.311	0.469	1.084	0.876	1.340	0.464	0.880	0.616	0.00	249.19	0.983
P35442	THBS2	3339_33	DHFA	28	0.911	0.727	1.141	0.417	0.911	0.703	1.180	0.478	0.986	0.691	1.407	0.938	0.599	0.072	29.71	181.38	0.983
P52823	STC1	4930_21	DHFA	9	1.188	0.778	1.813	0.426	1.582	0.894	2.798	0.115	0.757	0.201	2.853	0.693	0.489	0.330	12.57	93.50	0.983
O60760	HPGDS	12549_33	DHFA	36	0.917	0.740	1.137	0.432	0.944	0.729	1.221	0.659	1.035	0.733	1.463	0.846	0.340	0.177	17.80	142.14	0.983
Q7L7L0	HIST3H2	14144_3	DHFA	6	0.759	0.380	1.517	0.435	0.696	0.285	1.698	0.426	0.668	0.116	3.842	0.675	0.890	0.805	0.00	52.34	0.983
O95393	BMP10	3587_53	DHFA	4	1.265	0.700	2.285	0.436	1.381	0.692	2.754	0.360	1.708	0.565	5.161	0.443	0.628	0.674	0.00	109.04	0.983
Q9BQT9	CLSTN3	6291_55	DHFA	3	1.570	0.493	4.993	0.445	2.170	0.492	9.575	0.306	0.001	0.000	52016557.050	0.680	0.663	0.399	0.00	41.21	0.983

Q9H4D0	CLSTN2	18882_7	DHFA	27	1.087	0.870	1.360	0.462	1.065	0.770	1.472	0.704	1.137	0.709	1.824	0.598	0.964	0.858	0.00	107.55	0.983
Q16270	IGFBP7	3320_49	DHFA	26	0.911	0.707	1.175	0.474	0.970	0.694	1.356	0.860	1.209	0.765	1.909	0.424	0.147	0.692	0.00	99.61	0.983
Q8N6G6	ADAMTSL	16890_37	DHFA	15	1.094	0.850	1.409	0.485	1.052	0.763	1.452	0.755	1.031	0.717	1.482	0.872	0.500	0.929	0.00	144.92	0.983
Q9BY76	ANGPTL4	3796_79	DHFA	3	1.160	0.762	1.766	0.488	1.232	0.791	1.917	0.356	1.625	0.796	3.318	0.409	0.468	0.490	0.00	285.75	0.983
P40394	ADH7	11377_19	DHFA	12	0.893	0.646	1.235	0.494	0.824	0.564	1.204	0.317	0.666	0.406	1.092	0.138	0.145	0.633	0.00	117.02	0.983
Q9UBU2	DKK2	15678_71	DHFA	29	1.087	0.846	1.398	0.514	1.126	0.772	1.642	0.538	1.268	0.695	2.314	0.445	0.576	0.412	3.51	71.99	0.983
Q9BQ51	PDCD1LG	3004_67	DHFA	15	0.923	0.724	1.175	0.515	1.107	0.794	1.543	0.550	0.884	0.569	1.374	0.593	0.805	0.521	0.00	157.22	0.983
A6NGN9	IGLON5	6478_2	DHFA	6	0.809	0.414	1.583	0.536	0.971	0.433	2.178	0.943	0.785	0.069	8.966	0.855	0.968	0.805	0.00	51.78	0.983
Q14512	FGFBP1	15494_11	DHFA	9	0.825	0.445	1.531	0.542	0.959	0.435	2.112	0.917	1.639	0.255	10.548	0.619	0.475	0.300	16.03	42.04	0.983
O15123	ANGPT2	2602_2	DHFA	10	0.880	0.584	1.327	0.542	1.073	0.632	1.820	0.795	0.845	0.353	2.022	0.715	0.837	0.799	0.00	73.73	0.983
Q15768	EFNB3	2514_65	DHFA	2	1.288	0.545	3.039	0.564	----	----	----	----	----	----	----	----	----	----	----	97.41	0.983
P16860	NPPB	7655_11	DHFA	7	0.856	0.502	1.462	0.570	0.825	0.587	1.161	0.270	0.814	0.311	2.125	0.691	0.922	0.013	62.81	227.18	0.983
P02749	APOH	8288_27	DHFA	7	0.863	0.516	1.444	0.575	0.864	0.473	1.578	0.635	1.700	0.647	4.468	0.331	0.169	0.376	6.83	91.79	0.983
P04070	PROC	2961_1	DHFA	15	0.913	0.660	1.262	0.581	0.921	0.679	1.249	0.597	0.845	0.565	1.264	0.428	0.841	0.165	26.28	183.85	0.983
Q9UBI4	STOML1	17344_23	DHFA	4	1.266	0.545	2.941	0.583	1.266	0.473	3.390	0.639	1.585	0.235	10.692	0.683	0.833	0.745	0.00	43.18	0.983
Q8IW52	SLITRK4	7139_14	DHFA	3	0.761	0.279	2.077	0.594	0.790	0.239	2.611	0.699	0.093	0.000	9974.128	0.757	0.781	0.539	0.00	44.29	0.983
P05413	FABP3	5437_63	DHFA	3	1.224	0.570	2.628	0.605	0.731	0.301	1.772	0.488	0.033	0.002	0.587	0.259	0.240	0.035	70.28	80.74	0.983
Q9GZV9	FGF23	3807_1	DHFA	1	0.592	0.078	4.501	0.612	----	----	----	----	----	----	----	----	----	----	----	39.60	0.983
P14784	IL2RB	9343_16	DHFA	14	0.924	0.673	1.268	0.624	1.220	0.808	1.843	0.344	2.118	1.018	4.409	0.068	0.050	0.489	0.00	123.82	0.983

Q8N257	HIST3H2	18823_52	DHFA	5	0.856	0.458	1.600	0.626	0.775	0.372	1.614	0.496	0.613	0.183	2.051	0.485	0.546	0.441	0.00	77.65	0.983
Q9BWP8	COLEC11	4430_44	DHFA	43	1.031	0.911	1.167	0.630	1.096	0.922	1.303	0.300	1.088	0.905	1.309	0.376	0.418	0.804	0.00	276.77	0.983
P24821	TNC	4155_3	DHFA	71	1.028	0.913	1.157	0.651	0.957	0.808	1.133	0.610	0.936	0.775	1.131	0.497	0.384	0.750	0.00	200.39	0.983
Q9UNI1	CELA1	6107_3	DHFA	67	1.032	0.897	1.187	0.660	1.115	0.906	1.373	0.304	1.120	0.878	1.429	0.364	0.370	0.563	0.00	120.28	0.983
Q01973	ROR1	2590_69	DHFA	16	0.924	0.638	1.341	0.679	0.826	0.557	1.225	0.342	0.898	0.455	1.773	0.760	0.727	0.144	27.78	98.61	0.983
P01019	AGT	3484_60	DHFA	8	1.086	0.733	1.609	0.681	1.044	0.649	1.681	0.858	1.210	0.603	2.430	0.611	0.702	0.707	0.00	93.50	0.983
Q96AP7	ESAM	7841_84	DHFA	14	0.936	0.677	1.294	0.688	1.089	0.723	1.640	0.683	0.848	0.455	1.583	0.615	0.739	0.965	0.00	85.21	0.983
Q8WXD2	SCG3	7957_2	DHFA	31	0.957	0.744	1.231	0.733	0.979	0.766	1.252	0.866	1.139	0.792	1.639	0.489	0.269	0.056	30.54	144.64	0.983
Q9BXJ1	C1QTNF1	6304_8	DHFA	17	1.042	0.798	1.361	0.762	1.104	0.788	1.547	0.565	1.209	0.771	1.896	0.421	0.438	0.380	6.39	154.91	0.983
Q9UBX5	FBLN5	15585_304	DHFA	5	1.093	0.608	1.965	0.766	1.117	0.571	2.185	0.747	0.946	0.113	7.915	0.962	0.916	0.767	0.00	85.81	0.983
Q09028	RBBP4	15331_47	DHFA	4	1.143	0.438	2.983	0.785	1.197	0.382	3.749	0.757	6.876	0.008	5664.397	0.630	0.653	0.600	0.00	44.81	0.983
P14210	HGF	2681_23	DHFA	4	1.087	0.576	2.052	0.796	0.863	0.423	1.762	0.686	0.835	0.237	2.942	0.805	0.671	0.761	0.00	107.91	0.983
P28799	GRN	4992_49	DHFA	32	0.977	0.815	1.171	0.797	1.026	0.800	1.317	0.839	1.064	0.783	1.445	0.696	0.521	0.959	0.00	127.87	0.983
Q9UBX1	CTSF	9212_22	DHFA	28	0.969	0.761	1.234	0.798	0.979	0.699	1.370	0.901	0.836	0.549	1.274	0.413	0.427	0.655	0.00	94.16	0.983
O00622	CYR61	6264_9	DHFA	3	1.188	0.316	4.470	0.799	1.847	0.417	8.172	0.419	5175.807	0.056	477868876.300	0.381	0.389	0.292	18.79	40.57	0.983
Q8TEU8	WFIKKN2	3235_50	DHFA	24	1.025	0.848	1.239	0.799	1.123	0.890	1.416	0.328	1.261	0.937	1.697	0.140	0.153	0.253	15.01	164.44	0.983
Q9NQ75	CASS4	12855_16	DHFA	4	1.134	0.427	3.012	0.801	1.213	0.510	2.884	0.662	15.087	1.451	156.877	0.151	0.148	0.121	48.45	73.03	0.983
Q9NQ79	CRTAC1	5632_6	DHFA	35	1.018	0.879	1.179	0.809	1.096	0.887	1.355	0.394	0.905	0.724	1.130	0.383	0.190	0.707	0.00	270.83	0.983
O15197	EPHB6	5078_82	DHFA	7	1.066	0.630	1.804	0.811	1.054	0.687	1.616	0.811	1.520	0.686	3.369	0.342	0.209	0.087	43.69	124.48	0.983

P22607	FGFR3	13669_6	DHFA	7	0.906	0.405	2.026	0.811	0.839	0.490	1.437	0.523	1.245	0.121	12.777	0.861	0.815	0.007	65.81	112.26	0.983
Q9HB63	NTN4	3327_27	DHFA	13	1.046	0.721	1.516	0.814	1.416	0.888	2.257	0.144	1.929	0.952	3.910	0.096	0.095	0.436	1.00	89.09	0.983
Q9H0R8	GABARAP	12661_44	DHFA	2	0.878	0.297	2.601	0.815	----	----	----	----	----	----	----	----	----	----	----	61.32	0.983
P06396	GSN	4775_34	DHFA	14	0.958	0.669	1.372	0.816	1.130	0.752	1.696	0.556	1.151	0.647	2.046	0.642	0.464	0.199	23.53	65.84	0.983
P55083	MFAP4	5636_10	DHFA	24	0.951	0.621	1.456	0.816	1.076	0.669	1.729	0.763	0.853	0.250	2.909	0.802	0.862	0.054	33.91	51.59	0.983
P23468	PTPRD	9296_15	DHFA	5	1.117	0.434	2.875	0.818	0.983	0.341	2.833	0.975	3.115	0.088	110.034	0.576	0.597	0.930	0.00	34.00	0.983
Q86TH1	ADAMTSL	6379_62	DHFA	9	0.941	0.558	1.586	0.818	0.978	0.502	1.904	0.947	0.314	0.051	1.921	0.250	0.258	0.751	0.00	65.79	0.983
P01833	PIGR	3216_2	DHFA	12	0.931	0.493	1.758	0.825	0.900	0.499	1.624	0.727	0.736	0.139	3.910	0.727	0.730	0.018	52.02	66.26	0.983
Q8IUL8	CILP2	8841_65	DHFA	6	0.928	0.457	1.886	0.837	1.010	0.433	2.354	0.982	2.473	0.279	21.941	0.462	0.394	0.641	0.00	50.14	0.983
Q13017	ARHGAP5	14748_31	DHFA	4	0.910	0.363	2.282	0.841	1.088	0.367	3.227	0.879	13841.794	0.073	2634693261.000	0.264	0.260	0.387	0.89	40.62	0.983
Q9P121	NTM	10907_116	DHFA	26	0.978	0.782	1.224	0.846	1.049	0.801	1.375	0.727	0.903	0.639	1.276	0.569	0.671	0.184	19.74	201.85	0.983
Q9P121	NTM	8428_102	DHFA	29	1.019	0.835	1.244	0.852	0.984	0.770	1.258	0.897	0.951	0.695	1.301	0.757	0.618	0.374	5.98	150.42	0.983
O95319	CELF2	7245_2	DHFA	3	1.094	0.386	3.100	0.866	1.278	0.374	4.369	0.696	0.351	0.020	6.305	0.607	0.556	0.701	0.00	46.94	0.989
Q9BRK3	MXRA8	10521_10	DHFA	11	0.965	0.620	1.502	0.876	1.119	0.623	2.011	0.707	1.550	0.307	7.817	0.608	0.583	0.953	0.00	55.63	0.991
Q9H6X2	ANTXR1	10464_6	DHFA	7	0.973	0.598	1.584	0.912	0.988	0.521	1.873	0.971	0.319	0.083	1.229	0.158	0.159	0.560	0.00	79.81	0.991
Q02818	NUCB1	10451_11	DHFA	9	0.973	0.581	1.630	0.918	0.924	0.496	1.721	0.803	1.188	0.348	4.053	0.791	0.721	0.708	0.00	65.91	0.991
P25189	MPZ	10615_18	DHFA	26	0.991	0.809	1.214	0.931	0.920	0.704	1.203	0.544	1.125	0.768	1.649	0.550	0.387	0.660	0.00	123.58	0.991
P02679	FGG	4989_7	DHFA	6	1.023	0.594	1.759	0.936	1.126	0.608	2.084	0.706	1.561	0.467	5.216	0.510	0.493	0.973	0.00	76.21	0.991
P06396	GSN	16607_78	DHFA	18	0.990	0.745	1.315	0.946	1.099	0.776	1.558	0.595	1.142	0.737	1.772	0.560	0.395	0.326	10.77	65.00	0.991

Q04609	FOLH1	5478_50	DHFA	9	0.985	0.611	1.589	0.950	0.910	0.562	1.471	0.699	0.877	0.478	1.609	0.684	0.942	0.909	0.00	101.14	0.991
Q53FT3	C11orf7	8686_342	DHFA	3	1.030	0.392	2.706	0.952	1.555	0.526	4.598	0.425	0.000	0.000	22.268	0.396	0.394	0.063	63.88	53.94	0.991
Q9P2E7	PCDH10	9018_38	DHFA	5	1.013	0.611	1.679	0.959	1.109	0.621	1.981	0.727	1.689	0.512	5.570	0.452	0.456	0.628	0.00	115.87	0.991
Q6P4A8	PLBD1	6315_58	DHFA	2	0.968	0.246	3.807	0.963	----	----	----	----	----	----	----	----	----	----	----	40.64	0.991
Q4LDE5	SVEP1	11178_21	DHFA	37	1.004	0.838	1.204	0.965	1.175	0.892	1.548	0.252	1.338	0.959	1.868	0.095	0.067	0.547	0.00	98.87	0.991
P25311	AZGP1	9312_8	DHFA	10	0.995	0.666	1.486	0.979	1.147	0.717	1.837	0.567	1.567	0.811	3.029	0.218	0.128	0.377	7.07	110.99	0.991
P24592	IGFBP6	14088_38	DHFA	5	1.008	0.465	2.185	0.984	0.972	0.393	2.405	0.951	0.860	0.058	12.721	0.919	0.918	0.747	0.00	47.91	0.991
Q13508	ART3	7970_315	DHFA	20	0.998	0.750	1.330	0.991	0.970	0.664	1.417	0.876	1.045	0.622	1.756	0.869	0.850	0.475	0.00	99.75	0.991
Q6UY11	DLK2	9359_9	DHFA	7	1.003	0.582	1.730	0.991	1.068	0.646	1.768	0.797	0.829	0.250	2.750	0.772	0.698	0.227	26.38	97.19	0.991

Table S8. Mendelian randomization estimates for the associations between genetically predited protein levels using pQTLs from the Fenland cohort and DHFA as an outcome

UniProtID	Protein	SomaScan.id	outcome	snps	ivw.OR	ivw.l	ivw.u	ivw.p	wm.OR	wm.l	wm.u	wm.p	egger.OR	egger.l	egger.u	egger.p	Egger.intercept.p	Q.p	I2	F.statistic	p.ajd
P54764	EPHA4	SeqId_16288_17	DHFA	3	0.768	0.612	0.964	0.023	0.749	0.608	0.922	0.006	0.653	0.485	0.880	0.218	0.389	0.269	23.821	669.096	0.913
P08174	CD55	SeqId_5069_9	DHFA	1	0.811	0.669	0.985	0.034	----	----	----	----	----	----	----	----	----	----	----	1334.684	0.913
Q96EE4	CCDC126	SeqId_6388_21	DHFA	4	1.255	0.995	1.583	0.055	1.290	1.000	1.665	0.050	1.488	0.656	3.373	0.442	0.708	0.363	5.907	284.446	0.913
P52823	STC1	SeqId_4930_21	DHFA	2	0.506	0.251	1.017	0.056	----	----	----	----	----	----	----	----	----	----	----	49.809	0.913
Q9BXJ4	C1QTNF3	SeqId_7251_64	DHFA	1	0.628	0.385	1.023	0.062	----	----	----	----	----	----	----	----	----	----	----	223.424	0.913
O00468	AGRN	SeqId_15483_377	DHFA	3	0.796	0.627	1.012	0.063	0.796	0.623	1.018	0.069	0.496	0.069	3.551	0.612	0.718	0.885	0.000	283.813	0.913
Q6UXM1	LRIG3	SeqId_3322_52	DHFA	2	0.578	0.321	1.040	0.068	----	----	----	----	----	----	----	----	----	----	----	88.648	0.913
P58335	ANTXR2	SeqId_15559_5	DHFA	1	0.651	0.405	1.046	0.076	----	----	----	----	----	----	----	----	----	----	----	228.766	0.913
O00585	CCL21	SeqId_2516_57	DHFA	2	1.318	0.965	1.800	0.082	----	----	----	----	----	----	----	----	----	----	----	283.447	0.913
Q8IZS8	CACNA2D	SeqId_8885_6	DHFA	3	0.747	0.531	1.050	0.093	0.744	0.512	1.081	0.121	0.728	0.195	2.724	0.720	0.976	0.926	0.000	166.301	0.913
Q7Z3B1	NEGR1	SeqId_7050_5	DHFA	1	0.595	0.322	1.098	0.097	----	----	----	----	----	----	----	----	----	----	----	149.592	0.913
Q96AP7	ESAM	SeqId_7841_84	DHFA	2	1.286	0.930	1.778	0.128	----	----	----	----	----	----	----	----	----	----	----	288.679	0.953
Q16627	CCL14	SeqId_2900_53	DHFA	2	1.108	0.971	1.264	0.128	----	----	----	----	----	----	----	----	----	----	----	1795.903	0.953
Q13508	ART3	SeqId_7970_315	DHFA	1	1.450	0.897	2.344	0.129	----	----	----	----	----	----	----	----	----	----	----	250.972	0.953
Q03167	TGFBR3	SeqId_3009_3	DHFA	3	1.459	0.886	2.400	0.138	1.604	0.906	2.839	0.105	11006.855	0.000	1554777904810.230	0.509	0.522	0.624	0.000	70.336	0.953
P63098	PPP3CA	SeqId_4903_72	DHFA	2	1.201	0.906	1.590	0.202	----	----	----	----	----	----	----	----	----	----	----	409.183	0.985

Q08209	PPP3CA	SeqId_4903_72	DHFA	2	1.201	0.906	1.590	0.202	----	----	----	----	----	----	----	----	----	----	----	409.183	0.985
O94856	NFASC	SeqId_7179_69	DHFA	1	1.139	0.931	1.393	0.206	----	----	----	----	----	----	----	----	----	----	----	1195.184	0.985
Q8N3J6	CADM2	SeqId_16907_3	DHFA	1	0.475	0.148	1.526	0.211	----	----	----	----	----	----	----	----	----	----	----	51.178	0.985
P28799	GRN	SeqId_4992_49	DHFA	4	1.085	0.953	1.237	0.219	1.095	0.956	1.255	0.191	1.107	0.866	1.415	0.502	0.869	0.953	0.000	1146.633	0.985
Q15768	EFNB3	SeqId_2514_65	DHFA	1	0.549	0.191	1.575	0.265	----	----	----	----	----	----	----	----	----	----	----	61.562	0.985
Q7LFX5	CHST15	SeqId_4469_78	DHFA	3	0.852	0.642	1.130	0.266	0.863	0.650	1.146	0.310	0.501	0.220	1.140	0.347	0.407	0.404	0.000	250.144	0.985
Q14195	DPYSL3	SeqId_12707_26	DHFA	2	0.585	0.225	1.522	0.272	----	----	----	----	----	----	----	----	----	----	----	77.007	0.985
Q9BRK3	MXRA8	SeqId_10521_10	DHFA	3	0.763	0.469	1.240	0.274	0.728	0.427	1.241	0.243	1.311	0.351	4.893	0.756	0.545	0.676	0.000	69.430	0.985
O14773	TPP1	SeqId_17691_1	DHFA	3	1.264	0.824	1.937	0.283	1.223	0.725	2.065	0.450	8.753	0.126	610.430	0.499	0.534	0.580	0.000	105.571	0.985
Q9BXJ1	C1QTNF1	SeqId_6304_8	DHFA	6	1.082	0.933	1.254	0.297	1.110	0.942	1.308	0.213	1.180	0.912	1.525	0.276	0.465	0.953	0.000	510.182	0.985
Q9UBX1	CTSF	SeqId_9212_22	DHFA	3	0.879	0.684	1.129	0.311	0.873	0.666	1.144	0.325	0.622	0.210	1.846	0.549	0.638	0.636	0.000	282.997	0.985
Q02818	NUCB1	SeqId_10451_11	DHFA	1	1.464	0.698	3.070	0.313	----	----	----	----	----	----	----	----	----	----	----	100.000	0.985
Q9UBX5	FBLN5	SeqId_15585_304	DHFA	2	1.320	0.769	2.263	0.314	----	----	----	----	----	----	----	----	----	----	----	84.990	0.985
Q8WXD2	SCG3	SeqId_7957_2	DHFA	1	0.894	0.719	1.112	0.315	----	----	----	----	----	----	----	----	----	----	----	1438.577	0.985
P01160	NPPA	SeqId_5443_62	DHFA	1	1.285	0.756	2.182	0.354	----	----	----	----	----	----	----	----	----	----	----	191.090	0.985
O95393	BMP10	SeqId_3587_53	DHFA	1	1.346	0.718	2.525	0.354	----	----	----	----	----	----	----	----	----	----	----	135.654	0.985
P16860	NPPB	SeqId_7655_11	DHFA	1	0.827	0.548	1.248	0.365	----	----	----	----	----	----	----	----	----	----	----	332.361	0.985
Q9UHD0	IL19	SeqId_3035_80	DHFA	2	1.067	0.925	1.230	0.375	----	----	----	----	----	----	----	----	----	----	----	1479.453	0.985
Q14512	FGFBP1	SeqId_15494_11	DHFA	1	0.618	0.213	1.793	0.376	----	----	----	----	----	----	----	----	----	----	----	56.250	0.985

Q9HB63	NTN4	SeqId_3327_27	DHFA	2	1.284	0.738	2.236	0.376	----	----	----	----	----	----	----	----	----	----	----	153.824	0.985
O60760	HPGDS	SeqId_12549_33	DHFA	1	1.108	0.875	1.403	0.395	----	----	----	----	----	----	----	----	----	----	----	1068.787	0.985
Q01973	ROR1	SeqId_2590_69	DHFA	1	0.782	0.436	1.401	0.408	----	----	----	----	----	----	----	----	----	----	----	169.000	0.985
Q7Z3B1	NEGR1	SeqId_13109_82	DHFA	3	0.826	0.518	1.318	0.423	0.857	0.547	1.341	0.499	0.294	0.076	1.139	0.327	0.361	0.288	19.586	103.103	0.985
Q6P988	NOTUM	SeqId_8252_2	DHFA	2	0.835	0.536	1.302	0.426	----	----	----	----	----	----	----	----	----	----	----	100.984	0.985
P40394	ADH7	SeqId_11377_19	DHFA	1	2.381	0.273	20.788	0.433	----	----	----	----	----	----	----	----	----	----	----	10.332	0.985
Q16270	IGFBP7	SeqId_3320_49	DHFA	1	0.897	0.680	1.184	0.444	----	----	----	----	----	----	----	----	----	----	----	722.266	0.985
Q6UY11	DLK2	SeqId_9359_9	DHFA	1	1.278	0.680	2.400	0.446	----	----	----	----	----	----	----	----	----	----	----	139.240	0.985
Q4LDE5	SVEP1	SeqId_11109_56	DHFA	4	0.830	0.497	1.386	0.476	0.771	0.500	1.190	0.241	0.694	0.080	6.057	0.773	0.882	0.115	49.477	111.525	0.985
P11686	SFTPC	SeqId_5738_25	DHFA	5	1.073	0.884	1.302	0.477	1.036	0.818	1.313	0.770	1.100	0.705	1.715	0.703	0.910	0.613	0.000	298.269	0.985
Q8NCW5	APOA1BP	SeqId_16621_77	DHFA	1	0.878	0.609	1.266	0.488	----	----	----	----	----	----	----	----	----	----	----	316.840	0.985
P02741	CRP	SeqId_4337_49	DHFA	5	0.879	0.603	1.280	0.500	0.965	0.642	1.451	0.863	1.142	0.167	7.805	0.901	0.802	0.177	36.642	116.166	0.985
Q6P4A8	PLBD1	SeqId_6315_58	DHFA	1	0.722	0.274	1.903	0.510	----	----	----	----	----	----	----	----	----	----	----	69.098	0.985
P19429	TNNI3	SeqId_5441_67	DHFA	1	0.774	0.343	1.747	0.538	----	----	----	----	----	----	----	----	----	----	----	75.939	0.985
Q9P121	NTM	SeqId_8428_102	DHFA	3	1.066	0.868	1.310	0.541	1.070	0.862	1.328	0.540	1.218	0.728	2.040	0.590	0.678	0.796	0.000	553.426	0.985
Q8TEU8	WFIKKN2	SeqId_3235_50	DHFA	1	1.061	0.875	1.287	0.545	----	----	----	----	----	----	----	----	----	----	----	1587.716	0.985
Q9BWP8	COLEC11	SeqId_4430_44	DHFA	3	1.042	0.909	1.195	0.551	1.042	0.907	1.198	0.560	1.169	0.921	1.483	0.422	0.456	0.514	0.000	1071.434	0.985
Q9Y240	CLEC11A	SeqId_4500_50	DHFA	1	0.743	0.267	2.066	0.569	----	----	----	----	----	----	----	----	----	----	----	54.533	0.985
Q8WWZ8	OIT3	SeqId_6296_36	DHFA	1	0.777	0.320	1.887	0.577	----	----	----	----	----	----	----	----	----	----	----	74.225	0.985

P14210	HGF	SeqId_2681_23	DHFA	1	0.816	0.399	1.669	0.577	----	----	----	----	----	----	----	----	----	----	----	98.577	0.985
P05413	FABP3	SeqId_5437_63	DHFA	1	0.719	0.208	2.478	0.601	----	----	----	----	----	----	----	----	----	----	----	49.000	0.985
Q8N257	HIST3H2	SeqId_18823_52	DHFA	1	0.808	0.356	1.832	0.610	----	----	----	----	----	----	----	----	----	----	----	103.361	0.985
Q7L7L0	HIST3H2	SeqId_14144_3	DHFA	1	0.773	0.287	2.078	0.610	----	----	----	----	----	----	----	----	----	----	----	77.134	0.985
P25311	AZGP1	SeqId_9312_8	DHFA	2	1.070	0.813	1.407	0.631	----	----	----	----	----	----	----	----	----	----	----	417.778	0.985
P01833	PIGR	SeqId_3216_2	DHFA	1	1.154	0.639	2.084	0.636	----	----	----	----	----	----	----	----	----	----	----	169.000	0.985
P01019	AGT	SeqId_3484_60	DHFA	3	1.078	0.784	1.483	0.642	1.051	0.755	1.464	0.768	1.085	0.462	2.547	0.882	0.990	0.485	0.000	144.273	0.985
P22607	FGFR3	SeqId_13669_6	DHFA	1	0.831	0.341	2.028	0.685	----	----	----	----	----	----	----	----	----	----	----	74.225	0.985
P11597	CETP	SeqId_7131_207	DHFA	2	0.973	0.850	1.113	0.687	----	----	----	----	----	----	----	----	----	----	----	2065.014	0.985
Q9H6X2	ANTXR1	SeqId_10464_6	DHFA	1	0.822	0.314	2.148	0.689	----	----	----	----	----	----	----	----	----	----	----	77.189	0.985
Q9P121	NTM	SeqId_10907_116	DHFA	4	1.067	0.767	1.484	0.700	1.092	0.752	1.585	0.644	1.599	0.675	3.788	0.398	0.425	0.786	0.000	161.385	0.985
P14784	IL2RB	SeqId_9343_16	DHFA	1	1.223	0.439	3.409	0.700	----	----	----	----	----	----	----	----	----	----	----	45.761	0.985
Q9H4D0	CLSTN2	SeqId_18882_7	DHFA	3	1.061	0.781	1.440	0.705	1.021	0.725	1.438	0.905	0.964	0.520	1.790	0.927	0.787	0.757	0.000	171.914	0.985
Q9H772	GREM2	SeqId_5598_3	DHFA	3	1.117	0.612	2.038	0.718	1.226	0.601	2.499	0.576	7.963	0.026	2412.507	0.606	0.621	0.662	0.000	52.617	0.985
Q9NQ79	CRTAC1	SeqId_5632_6	DHFA	4	0.975	0.851	1.118	0.720	0.915	0.779	1.076	0.284	0.857	0.596	1.233	0.494	0.528	0.421	0.000	843.294	0.985
Q92743	HTRA1	SeqId_15594_47	DHFA	1	1.131	0.554	2.309	0.734	----	----	----	----	----	----	----	----	----	----	----	111.420	0.985
O15123	ANGPT2	SeqId_2602_2	DHFA	1	1.198	0.418	3.430	0.736	----	----	----	----	----	----	----	----	----	----	----	54.128	0.985
Q96GL9	FAM163A	SeqId_6260_14	DHFA	3	0.960	0.745	1.236	0.750	0.984	0.765	1.265	0.900	1.024	0.592	1.769	0.947	0.837	0.825	0.000	309.242	0.985
P0C0P6	NPS	SeqId_6390_18	DHFA	1	1.131	0.503	2.541	0.766	----	----	----	----	----	----	----	----	----	----	----	77.440	0.985

P02679	FGG	SeqId_4989_7	DHFA	1	1.104	0.549	2.220	0.780	----	----	----	----	----	----	----	----	----	----	----	115.879	0.985
Q09028	RBBP4	SeqId_15331_47	DHFA	1	1.129	0.476	2.678	0.784	----	----	----	----	----	----	----	----	----	----	----	79.621	0.985
Q13017	ARHGAP5	SeqId_14748_31	DHFA	1	1.120	0.499	2.513	0.784	----	----	----	----	----	----	----	----	----	----	----	90.982	0.985
Q53FT3	C11orf7	SeqId_8686_342	DHFA	1	1.145	0.437	3.000	0.784	----	----	----	----	----	----	----	----	----	----	----	64.000	0.985
P51888	PRELP	SeqId_5675_6	DHFA	3	0.985	0.882	1.099	0.785	0.983	0.882	1.097	0.762	0.950	0.789	1.144	0.685	0.709	0.484	0.000	2754.252	0.985
Q9UNI1	CELA1	SeqId_6107_3	DHFA	3	0.978	0.835	1.146	0.786	0.980	0.836	1.150	0.808	0.983	0.749	1.290	0.921	0.974	0.958	0.000	986.360	0.985
Q9BQ51	PDCD1LG	SeqId_3004_67	DHFA	3	0.971	0.784	1.204	0.791	0.984	0.802	1.207	0.876	1.219	0.821	1.811	0.506	0.418	0.303	16.145	511.556	0.985
O95633	FSTL3	SeqId_3438_10	DHFA	1	0.868	0.301	2.506	0.794	----	----	----	----	----	----	----	----	----	----	----	54.533	0.985
Q9HCB6	SPON1	SeqId_4297_62	DHFA	3	1.047	0.739	1.483	0.795	1.100	0.851	1.423	0.466	1.205	0.481	3.021	0.758	0.787	0.142	48.718	343.123	0.985
Q9HCR9	PDE11A	SeqId_5252_33	DHFA	2	1.071	0.604	1.896	0.815	----	----	----	----	----	----	----	----	----	----	----	91.423	0.985
Q8N6G6	ADAMTSL	SeqId_16890_37	DHFA	1	1.052	0.681	1.626	0.819	----	----	----	----	----	----	----	----	----	----	----	206.989	0.985
P35442	THBS2	SeqId_3339_33	DHFA	1	0.976	0.793	1.202	0.820	----	----	----	----	----	----	----	----	----	----	----	1413.760	0.985
P25189	MPZ	SeqId_10615_18	DHFA	6	0.969	0.727	1.291	0.829	0.895	0.627	1.276	0.538	1.527	0.586	3.978	0.435	0.384	0.494	0.000	135.145	0.985
Q9BY76	ANGPTL4	SeqId_3796_79	DHFA	3	1.031	0.781	1.361	0.830	1.027	0.760	1.388	0.861	1.507	0.806	2.817	0.421	0.411	0.413	0.000	234.818	0.985
P55083	MFAP4	SeqId_5636_10	DHFA	1	1.087	0.477	2.479	0.843	----	----	----	----	----	----	----	----	----	----	----	80.003	0.985
Q96IZ0	PAWR	SeqId_9565_6	DHFA	2	0.933	0.442	1.970	0.855	----	----	----	----	----	----	----	----	----	----	----	56.084	0.985
Q9NQ75	CASS4	SeqId_12855_16	DHFA	2	1.052	0.605	1.831	0.857	----	----	----	----	----	----	----	----	----	----	----	85.151	0.985
Q86TH1	ADAMTSL	SeqId_6379_62	DHFA	4	1.057	0.568	1.968	0.861	1.001	0.561	1.787	0.997	0.905	0.056	14.543	0.950	0.920	0.152	43.325	66.345	0.985
Q86Y29	BAGE3	SeqId_6442_6	DHFA	1	1.086	0.394	2.990	0.873	----	----	----	----	----	----	----	----	----	----	----	58.778	0.985

P24821	TNC	SeqId_4155_3	DHFA	3	1.008	0.909	1.118	0.875	1.013	0.912	1.125	0.809	1.043	0.889	1.224	0.697	0.684	0.769	0.000	2989.859	0.985
Q9UBU2	DKK2	SeqId_15678_71	DHFA	3	1.032	0.663	1.609	0.888	0.917	0.566	1.485	0.724	0.012	0.000	2.663	0.355	0.352	0.269	23.943	131.887	0.985
P08670	VIM	SeqId_15540_6	DHFA	2	1.074	0.390	2.958	0.890	----	----	----	----	----	----	----	----	----	----	----	102.495	0.985
O00622	CYR61	SeqId_6264_9	DHFA	2	0.967	0.554	1.687	0.905	----	----	----	----	----	----	----	----	----	----	----	62.839	0.991
O15197	EPHB6	SeqId_5078_82	DHFA	1	1.042	0.459	2.366	0.922	----	----	----	----	----	----	----	----	----	----	----	55.992	0.991
Q4LDE5	SVEP1	SeqId_11178_21	DHFA	3	0.976	0.558	1.709	0.933	0.978	0.620	1.543	0.924	0.608	0.056	6.589	0.753	0.752	0.132	50.671	132.234	0.991
P06396	GSN	SeqId_4775_34	DHFA	1	1.027	0.539	1.954	0.936	----	----	----	----	----	----	----	----	----	----	----	133.691	0.991
P04070	PROC	SeqId_2961_1	DHFA	2	0.986	0.665	1.461	0.943	----	----	----	----	----	----	----	----	----	----	----	392.281	0.991
Q13332	PTPRS	SeqId_6049_64	DHFA	1	0.981	0.329	2.922	0.973	----	----	----	----	----	----	----	----	----	----	----	46.046	0.994
Q9H0R8	GABARAP	SeqId_12661_44	DHFA	1	0.989	0.429	2.281	0.979	----	----	----	----	----	----	----	----	----	----	----	103.101	0.994
O95166	GABARAP	SeqId_17735_130	DHFA	1	0.989	0.447	2.188	0.979	----	----	----	----	----	----	----	----	----	----	----	114.325	0.994
Q16623	STX1A	SeqId_19553_14	DHFA	2	0.995	0.612	1.619	0.985	----	----	----	----	----	----	----	----	----	----	----	121.464	0.994
P01011	SERPINA	SeqId_4153_11	DHFA	2	0.999	0.769	1.299	0.996	----	----	----	----	----	----	----	----	----	----	----	478.382	0.996

Table S9. Results from unadjusted cox regression models for death outcomes in the PHFS, replicated in the WashU cohort.

SOMA_ID	PHFS						WashU					
	N	sHR	Standardized.LB.95CI	Standardized.UB.95CI	Alpha.corrected.P.value	minus_log10_P	N	sHR	Standardized_LB_95CI	Standardized_UB_95CI	minus_log10_P	P_value
SOMA_Q99988_GDF15_4374_45	2209	2.346	2.145	2.567	0	76.545	1077	1.843	1.697	2.002	47.022	<0.0001
SOMA_O95633_FSTL3_3438_10	2209	2.311	2.111	2.529	0	73.129	1077	1.647	1.525	1.778	36.345	<0.0001
SOMA_P61769_B2M_3485_28	2209	2.217	2.034	2.416	0	72.766	1077	1.334	1.252	1.421	18.196	<0.0001
SOMA_Q01995_TAGLN_15640_54	2209	2.19	2.009	2.387	0	70.396	1077	1.374	1.287	1.467	20.666	<0.0001
SOMA_P01034_CST3_2609_59	2209	2.162	1.986	2.353	0	70.279	1077	1.440	1.341	1.546	22.925	<0.0001
SOMA_Q12805_EFEMP1_8480_29	2209	2.244	2.051	2.454	0	68.859	1077	1.415	1.329	1.506	26.964	<0.0001
SOMA_Q14508_WFDC2_11388_75	2209	2.16	1.98	2.356	0	66.971	1077	1.917	1.737	2.115	37.520	<0.0001
SOMA_P07998_RNASE1_7211_2	2209	2.134	1.959	2.325	0	66.697	1077	1.247	1.177	1.320	13.347	<0.0001
SOMA_Q4LDE5_SVEP1_11109_56	2209	2.212	2.021	2.421	0	65.943	1077	1.478	1.378	1.585	27.295	<0.0001
SOMA_Q4LDE5_SVEP1_11178_21	2209	2.2	2.01	2.408	0	64.979	1077	1.501	1.397	1.612	28.003	<0.0001
SOMA_P61956_SUMO2_19555_1	2209	2.074	1.907	2.256	0	64.181	1077	1.207	1.134	1.285	8.459	<0.0001
SOMA_Q2UY09_COL28A1_10702_1	2209	2.108	1.933	2.298	0	63.46	1077	1.394	1.297	1.499	18.761	<0.0001
SOMA_Q01974_ROR2_7861_9	2209	2.057	1.89	2.238	0	62.331	1077	1.243	1.170	1.320	11.674	<0.0001
SOMA_P16860_NPPB_7655_11	2209	2.208	2.012	2.423	0	61.998	1077	1.840	1.676	2.020	36.899	<0.0001
SOMA_P12111_COL6A3_11196_31	2209	2.081	1.907	2.27	0	60.526	1077	1.271	1.193	1.353	13.000	<0.0001
SOMA_Q07654_TFF3_4721_54	2209	1.972	1.817	2.139	0	59.263	1077	1.243	1.161	1.332	9.258	<0.0001

SOMA_Q9H0R8_GABARAP_12661_44	2209	1.984	1.827	2.154	0	59.231	1077	1.083	1.028	1.141	2.576	0.0027
SOMA_O00244_ATOX1_19233_75	2209	1.984	1.827	2.155	0	58.933	1077	1.106	1.051	1.165	3.884	0.0001
SOMA_Q9HCB6_SPON1_4297_62	2209	2.105	1.925	2.302	0	58.932	1077	1.494	1.392	1.603	28.245	<0.0001
SOMA_P17900_GM2A_15441_6	2209	2.044	1.875	2.228	0	58.568	1077	1.338	1.253	1.428	17.641	<0.0001
SOMA_Q9BU40_CHRDL1_3362_61	2209	2.044	1.875	2.23	0	58.095	1077	1.632	1.486	1.791	24.031	<0.0001
SOMA_O95166_GABARAP_17735_130	2209	1.913	1.768	2.07	0	57.737	1077	1.251	1.185	1.321	15.276	<0.0001
SOMA_Q92626_PXDN_13463_1	2209	1.99	1.83	2.163	0	57.592	1077	1.282	1.206	1.363	14.730	<0.0001
SOMA_Q96GP6_SCARF2_8956_96	2209	1.885	1.744	2.038	0	56.787	1077	1.550	1.435	1.674	28.199	<0.0001
SOMA_P39060_COL18A1_2201_17	2209	1.956	1.801	2.125	0	56.109	1077	1.534	1.426	1.649	29.980	<0.0001
SOMA_P61916_NPC2_6259_60	2209	1.993	1.83	2.171	0	55.736	NA	NA	NA	NA	NA	NA
SOMA_P22223_CDH3_2643_57	2209	0.491	0.449	0.536	0	55.163	1077	0.376	0.320	0.441	31.865	<0.0001
SOMA_Q06141_REG3A_15304_1	2209	2.011	1.843	2.193	0	55.158	1077	1.399	1.315	1.489	25.410	<0.0001
SOMA_Q07654_TFF3_8323_163	2209	1.92	1.77	2.083	0	54.736	1077	1.141	1.063	1.226	3.546	0.0003
SOMA_P07108_DBI_16919_1	2209	1.95	1.793	2.119	0	54.648	1077	1.244	1.135	1.363	5.541	<0.0001
SOMA_Q02487_DSC2_13126_52	2209	1.985	1.821	2.163	0	54.448	1077	1.238	1.164	1.317	10.876	<0.0001
SOMA_P35442_THBS2_3339_33	2209	2.03	1.857	2.219	0	54.216	1077	1.760	1.625	1.907	42.975	<0.0001
SOMA_P00533_EGFR_2677_1	2209	0.509	0.468	0.554	0	54.2	1077	0.588	0.506	0.683	11.421	<0.0001
SOMA_O15123_ANGPT2_2602_2	2209	1.955	1.797	2.127	0	54.153	1077	1.923	1.745	2.119	39.056	<0.0001
SOMA_Q9UBX5_FBLN5_15585_304	2209	2.04	1.865	2.232	0	53.817	1077	2.197	1.971	2.449	44.982	<0.0001

SOMA_Q9NP99_TREM1_9266_1	2209	1.952	1.794	2.125	0	53.553	1077	1.448	1.350	1.553	24.491	<0.0001
SOMA_P06276_BCHE_15514_26	2209	0.49	0.448	0.537	0	53.211	1077	0.368	0.312	0.434	31.770	<0.0001
SOMA_Q93091_RNASE6_5646_20	2209	1.935	1.78	2.104	0	53.162	1077	1.179	1.117	1.245	8.526	<0.0001
SOMA_Q9BQT9_CLSTN3_6291_55	2209	1.94	1.782	2.111	0	52.525	1077	1.335	1.236	1.441	12.679	<0.0001
SOMA_P08949_NMB_9321_400	2209	1.906	1.755	2.07	0	52.475	1077	1.103	1.042	1.169	3.082	0.0008
SOMA_Q9Y5H3_PCDHGA1_6321_65	2209	1.862	1.719	2.016	0	52.161	1077	1.040	0.961	1.125	0.482	0.3293
SOMA_P19438_TNFRSF1_2654_19	2209	1.887	1.739	2.047	0	51.881	1077	1.420	1.327	1.520	23.540	<0.0001
SOMA_Q8N2S1_LTBP4_13133_73	2209	1.916	1.763	2.084	0	51.643	1077	1.972	1.779	2.185	37.711	<0.0001
SOMA_Q8WWX9_SELM_15336_7	2209	1.864	1.72	2.02	0	51.382	NA	NA	NA	NA	NA	NA
SOMA_P61626_LYZ_4920_10	2209	1.924	1.767	2.094	0	51.003	1077	1.364	1.263	1.472	14.655	<0.0001
SOMA_P10912_GHR_2948_58	2209	0.514	0.472	0.56	0	50.779	1077	0.379	0.309	0.465	19.971	<0.0001
SOMA_Q8IUL8_CILP2_8841_65	2209	0.531	0.489	0.577	0	50.166	1077	0.379	0.303	0.474	16.778	<0.0001
SOMA_Q13790_APOF_12370_30	2209	1.938	1.777	2.114	0	49.666	1077	1.774	1.634	1.927	41.385	<0.0001
SOMA_Q9GZX9_TWSG1_9234_8	2209	1.938	1.777	2.115	0	49.616	1077	1.413	1.316	1.517	20.771	<0.0001
SOMA_P41271_NBL1_2944_66	2209	1.926	1.766	2.099	0	49.291	1077	1.216	1.146	1.290	10.116	<0.0001
SOMA_Q6P988_NOTUM_8252_2	2209	0.535	0.493	0.581	0	49.222	1077	0.217	0.156	0.303	18.728	<0.0001
SOMA_Q9UJJ9_GNPTG_10666_7	2209	1.812	1.675	1.96	0	48.932	1077	1.462	1.350	1.584	19.951	<0.0001
SOMA_Q9HC57_WFDC1_9316_67	2209	1.822	1.683	1.974	0	48.544	1077	1.509	1.390	1.638	22.045	<0.0001
SOMA_Q16627_CCL14_2900_53	2209	1.938	1.774	2.116	0	48.302	1077	1.622	1.510	1.741	39.856	<0.0001

SOMA_P49755_TMED10_6506_54	2209	1.882	1.729	2.047	0	48.125	1077	1.227	1.150	1.310	9.181	<0.0001
SOMA_P18065_IGFBP2_8469_41	2209	1.992	1.817	2.184	0	48.105	1077	1.779	1.619	1.954	32.417	<0.0001
SOMA_Q86TH1_ADAMTSL_6379_62	2209	1.933	1.77	2.111	0	48.086	1077	1.684	1.544	1.838	30.996	<0.0001
SOMA_P29317_EPHA2_4834_61	2209	1.83	1.687	1.986	0	46.831	1077	1.333	1.251	1.421	18.026	<0.0001
SOMA_P54826_GAS1_5463_22	2209	1.83	1.686	1.986	0	46.742	1077	1.457	1.336	1.588	16.911	<0.0001
SOMA_Q9BQI0_AIF1L_18871_24	2209	1.872	1.719	2.039	0	46.485	1077	1.380	1.257	1.514	10.966	<0.0001
SOMA_O15013_ARHGEF1_9061_3	2209	0.576	0.534	0.62	0	46.408	1077	0.476	0.307	0.739	3.031	0.0009
SOMA_O14786_NRP1_5542_22	2209	1.872	1.719	2.039	0	46.27	1077	1.182	1.124	1.243	10.127	<0.0001
SOMA_P20333_TNFRSF1_8368_102	2209	1.886	1.73	2.056	0	46.165	1077	1.174	1.120	1.230	10.746	<0.0001
SOMA_Q01844_EWSR1_12988_49	2209	1.802	1.663	1.953	0	45.887	1077	1.368	1.257	1.488	12.501	<0.0001
SOMA_Q9Y5P4_COL4A3B_13950_9	2209	1.829	1.684	1.987	0	45.771	NA	NA	NA	NA	NA	NA
SOMA_Q9BX93_PLA2G12_9380_2	2209	0.538	0.494	0.586	0	45.35	1077	0.371	0.298	0.462	18.092	<0.0001
SOMA_Q01638_IL1RL1_4234_8	2209	1.901	1.74	2.077	0	45.308	1077	1.479	1.387	1.576	32.380	<0.0001
SOMA_P34096_RNASE4_5644_60	2209	1.787	1.65	1.935	0	45.279	1077	1.901	1.685	2.145	24.727	<0.0001
SOMA_Q01105_SET_5364_7	2209	0.547	0.503	0.594	0	45.241	1077	0.512	0.438	0.598	16.512	<0.0001
SOMA_Q9H6Z4_RANBP3_14037_18	2209	1.891	1.732	2.065	0	45.14	1077	1.372	1.258	1.496	12.136	<0.0001
SOMA_P42167_TMPO_8265_225	2209	1.842	1.692	2.005	0	44.595	1077	1.253	1.145	1.371	6.013	<0.0001
SOMA_P01011_SERPINA_4153_11	2209	1.862	1.707	2.031	0	43.941	1077	1.414	1.336	1.495	32.713	<0.0001
SOMA_P00747_PLG_3710_49	2209	0.537	0.493	0.586	0	43.875	1077	0.455	0.386	0.536	20.219	<0.0001

SOMA_P20333_TNFRSF1_3152_57	2209	1.859	1.704	2.028	0	43.772	1077	1.085	1.033	1.139	2.945	0.0011
SOMA_Q03405_PLAUR_2652_15	2209	1.537	1.447	1.633	0	43.388	1077	1.622	1.494	1.762	29.869	<0.0001
SOMA_P21757_MSR1_15533_97	2209	1.79	1.649	1.943	0	43.376	1077	1.540	1.408	1.685	20.496	<0.0001
SOMA_Q9UHV9_PFDN2_19243_2	2209	1.809	1.664	1.967	0	43.23	1077	1.127	1.061	1.198	3.986	0.0001
SOMA_O95445_APOM_10445_20	2209	0.553	0.509	0.601	0	42.95	1077	0.491	0.371	0.651	6.137	<0.0001
SOMA_P03973_SLPI_4413_3	2209	1.817	1.669	1.977	0	42.95	1077	1.159	1.106	1.216	9.058	<0.0001
SOMA_P30050_RPL12_19183_164	2209	1.699	1.576	1.832	0	42.878	1077	1.345	1.212	1.493	7.605	<0.0001
SOMA_Q2I0M5_RSPO4_8464_31	2209	1.859	1.702	2.03	0	42.734	1077	1.176	1.108	1.247	7.106	<0.0001
SOMA_Q8TDQ0_HAVCR2_5134_52	2209	1.814	1.667	1.973	0	42.716	1077	1.553	1.434	1.681	26.833	<0.0001
SOMA_P15090_FABP4_15386_7	2209	1.828	1.678	1.991	0	42.705	1077	1.778	1.600	1.975	26.065	<0.0001
SOMA_Q9BUD6_SPON2_8099_42	2209	1.817	1.669	1.978	0	42.603	1077	1.761	1.592	1.948	27.416	<0.0001
SOMA_Q9UNK0_STX8_10903_50	2209	1.795	1.652	1.951	0	42.435	1077	1.102	0.966	1.257	0.834	0.1465
SOMA_Q9NS68_TNFRSF1_5131_15	2209	1.8	1.655	1.957	0	42.423	1077	1.161	1.085	1.242	4.784	<0.0001
SOMA_Q96DZ1_ERLEC1_8957_72	2209	1.787	1.645	1.942	0	42.176	1077	1.123	1.048	1.203	3.029	0.0009
SOMA_P52823_STC1_4930_21	2209	1.833	1.681	1.999	0	42.12	1077	1.667	1.518	1.830	26.010	<0.0001
SOMA_P05452_CLEC3B_5701_81	2209	0.548	0.503	0.597	0	41.937	1077	0.480	0.411	0.560	19.874	<0.0001
SOMA_Q96PQ1_SIGLEC1_8352_26	2209	0.576	0.532	0.623	0	41.825	1077	0.404	0.330	0.495	17.669	<0.0001
SOMA_O60330_PCDHGA1_6938_21	2209	1.793	1.649	1.949	0	41.819	1077	1.067	0.999	1.141	1.266	0.0542
SOMA_Q86Y82_STX12_10418_36	2209	1.73	1.599	1.871	0	41.743	1077	1.123	0.990	1.273	1.147	0.0713

SOMA_Q9C005_DPY30_13943_38	2209	1.817	1.667	1.979	0	41.734	1077	1.448	1.322	1.587	14.757	<0.0001
SOMA_P45379_TNNT2_5315_22	2209	1.801	1.655	1.959	0	41.69	1077	1.201	1.127	1.280	7.694	<0.0001
SOMA_Q8WWZ8_OIT3_6296_36	2209	1.759	1.622	1.909	0	41.389	1077	1.214	1.121	1.315	5.745	<0.0001
SOMA_Q9H4F8_SMOC1_13118_5	2209	1.784	1.641	1.939	0	41.299	1077	1.346	1.236	1.466	11.078	<0.0001
SOMA_P40121_CAPG_4968_50	2209	1.798	1.652	1.958	0	40.919	1077	1.273	1.177	1.376	8.823	<0.0001
SOMA_P52798_EFNA4_2614_28	2209	1.747	1.61	1.896	0	40.031	1077	1.228	1.150	1.311	9.047	<0.0001
SOMA_Q9NXW2_DNAJB12_8006_12	2209	1.633	1.52	1.756	0	39.699	1077	1.079	1.020	1.142	2.099	0.008
SOMA_P98172_EFNB1_13104_32	2209	1.759	1.618	1.911	0	39.675	1077	1.200	1.127	1.278	7.856	<0.0001
SOMA_P07949_RET_3220_40	2209	0.569	0.523	0.618	0	39.596	1077	0.252	0.170	0.372	11.350	<0.0001
SOMA_Q12907_LMAN2_9468_8	2209	1.787	1.639	1.947	0	39.211	1077	1.343	1.256	1.435	17.268	<0.0001
SOMA_P08697_SERPINF_3024_18	2209	0.565	0.519	0.615	0	39.152	1077	0.683	0.592	0.787	6.848	<0.0001
SOMA_P18440_NAT1_12632_14	2209	0.573	0.527	0.622	0	38.92	1077	0.321	0.241	0.429	13.869	<0.0001
SOMA_P16152_CBR1_12381_26	2209	1.774	1.629	1.932	0	38.835	1077	1.472	1.363	1.588	22.467	<0.0001
SOMA_Q13145_BAMBI_8811_24	2209	1.729	1.593	1.877	0	38.414	1077	1.172	1.095	1.255	5.267	<0.0001
SOMA_P41222_PTGDS_10514_5	2209	1.756	1.614	1.911	0	38.332	1077	1.382	1.277	1.496	14.937	<0.0001
SOMA_Q9H4D0_CLSTN2_18882_7	2209	1.818	1.662	1.988	0	38.327	1077	1.290	1.222	1.363	19.345	<0.0001
SOMA_Q9Y5I4_PCDHAC2_9361_7	2209	1.713	1.58	1.857	0	38.208	1077	1.013	0.898	1.144	0.081	0.8291
SOMA_P09529_INHBB_13676_46	2209	1.764	1.62	1.922	0	38.169	1077	1.289	1.213	1.370	15.569	<0.0001
SOMA_Q8NBS9_TXNDC5_11212_7	2209	1.776	1.629	1.936	0	38.005	1077	1.243	1.151	1.343	7.532	<0.0001

SOMA_Q7Z4F1_LRP10_16610_13	2209	1.627	1.512	1.751	0	37.981	1077	1.348	1.268	1.433	21.062	<0.0001
SOMA_O14558_HSPB6_19127_1	2209	1.691	1.562	1.831	0	37.786	1077	1.408	1.300	1.526	16.308	<0.0001
SOMA_P35858_IGFALS_6605_17	2209	0.569	0.522	0.62	0	37.775	1077	0.544	0.470	0.629	15.710	<0.0001
SOMA_Q2MKA7_RSPO1_16614_27	2209	1.728	1.591	1.877	0	37.689	1077	1.258	1.157	1.368	7.080	<0.0001
SOMA_Q12841_FSTL1_13112_179	2209	1.753	1.61	1.91	0	37.216	1077	1.731	1.591	1.884	36.297	<0.0001
SOMA_Q16629_SRSF7_12987_12	2209	1.71	1.576	1.856	0	37.083	1077	1.507	1.395	1.628	24.666	<0.0001
SOMA_Q16270_IGFBP7_3320_49	2209	1.791	1.639	1.958	0	37.01	1077	1.774	1.610	1.954	30.299	<0.0001
SOMA_Q76LX8_ADAMTS1_3175_51	2209	0.581	0.535	0.631	0	37.007	1077	0.533	0.456	0.624	14.301	<0.0001
SOMA_Q9H665_IGFLR1_7244_16	2209	1.702	1.569	1.846	0	36.936	1077	1.182	1.108	1.260	6.463	<0.0001
SOMA_P51965_UBE2E1_14326_4	2209	1.692	1.561	1.833	0	36.823	1077	1.071	0.936	1.227	0.497	0.3181
SOMA_Q9UHX1_PUF60_10575_31	2209	1.69	1.559	1.831	0	36.66	1077	1.223	1.161	1.288	13.409	<0.0001
SOMA_P40261_NNMT_19376_74	2209	1.68	1.551	1.819	0	36.633	1077	1.073	1.003	1.148	1.393	0.0404
SOMA_Q13261_IL15RA_14054_17	2209	1.721	1.584	1.871	0	36.623	1077	1.350	1.263	1.443	18.068	<0.0001
SOMA_Q03403_TFF2_9191_8	2209	1.746	1.602	1.902	0	36.557	1077	1.396	1.300	1.499	19.396	<0.0001
SOMA_Q9BXJ1_C1QTNF1_6304_8	2209	1.722	1.584	1.872	0	36.502	1077	1.455	1.357	1.561	24.894	<0.0001
SOMA_Q86Y30_BAGE2_6294_11	2209	1.721	1.582	1.871	0	36.307	1077	1.119	1.020	1.226	1.770	0.017
SOMA_P80188_LCN2_2836_68	2209	1.742	1.599	1.897	0	36.283	1077	1.212	1.149	1.279	11.680	<0.0001
SOMA_O75056_SDC3_16612_28	2209	0.579	0.532	0.63	0	36.207	1077	0.233	0.170	0.321	18.474	<0.0001
SOMA_P05451_REG1A_13095_51	2209	1.733	1.592	1.886	0	36.19	1077	1.252	1.171	1.339	10.348	<0.0001

SOMA_P14649_MYL6B_14227_21	2209	1.704	1.569	1.85	0	35.988	1077	1.203	1.126	1.286	7.355	<0.0001
SOMA_Q9NR71_ASAH2_3212_30	2209	0.575	0.527	0.627	0	35.494	1077	0.357	0.277	0.459	14.986	<0.0001
SOMA_Q9H772_GREM2_5598_3	2209	1.684	1.552	1.827	0	35.304	1077	1.252	1.179	1.330	12.561	<0.0001
SOMA_P43652_AFM_4763_31	2209	0.569	0.521	0.622	0	35.266	1077	0.494	0.428	0.571	20.954	<0.0001
SOMA_P51858_HDGF_8953_47	2209	1.674	1.543	1.815	0	34.864	1077	1.096	1.024	1.174	2.081	0.0083
SOMA_O60911_CTSV_3364_76	2209	0.592	0.545	0.643	0	34.702	1077	0.730	0.491	1.085	0.922	0.1196
SOMA_P62995_TRA2B_12373_73	2209	1.645	1.52	1.78	0	34.203	1077	1.190	1.131	1.251	10.923	<0.0001
SOMA_O60760_HPGDS_12549_33	2209	0.607	0.561	0.657	0	34.18	1077	0.336	0.261	0.434	16.290	<0.0001
SOMA_Q86VZ4_LRP11_15472_16	2209	1.705	1.566	1.856	0	34.111	1077	1.380	1.274	1.496	14.362	<0.0001
SOMA_Q8NBJ4_GOLM1_17456_53	2209	1.716	1.575	1.87	0	34.108	1077	1.271	1.204	1.342	17.346	<0.0001
SOMA_Q16663_CCL15_18289_16	2209	1.711	1.571	1.864	0	34.107	1077	1.512	1.366	1.673	14.870	<0.0001
SOMA_P48304_REG1B_16770_3	2209	1.695	1.558	1.844	0	33.906	1077	1.493	1.381	1.614	23.294	<0.0001
SOMA_O14737_PDCD5_12517_52	2209	1.684	1.549	1.831	0	33.678	1077	1.337	1.172	1.525	4.831	<0.0001
SOMA_Q9HAV5_EDA2R_3083_71	2209	1.67	1.538	1.813	0	33.614	1077	1.106	1.037	1.180	2.676	0.0021
SOMA_P11362_FGFR1_5532_53	2209	1.749	1.599	1.913	0	33.612	1077	1.473	1.368	1.587	23.956	<0.0001
SOMA_Q14162_SCARF1_5129_12	2209	1.699	1.56	1.85	0	33.521	1077	1.281	1.154	1.422	5.495	<0.0001
SOMA_P07478_PRSS2_5034_79	2209	1.712	1.57	1.867	0	33.386	1077	1.213	1.152	1.277	12.707	<0.0001
SOMA_P55957_BID_5798_3	2209	1.669	1.537	1.812	0	33.384	1077	1.068	1.001	1.139	1.338	0.046
SOMA_Q12906_ILF3_12759_47	2209	1.668	1.536	1.812	0	33.29	1077	1.280	1.187	1.380	9.806	<0.0001

SOMA_P07858_CTSB_8007_19	2209	1.751	1.6	1.917	0	33.205	1077	1.603	1.487	1.729	33.714	<0.0001
SOMA_P11686_SFTPC_5738_25	2209	1.719	1.575	1.876	0	33.164	NA	NA	NA	NA	NA	NA
SOMA_Q96DX5_ASB9_19601_15	2209	1.651	1.522	1.791	0	32.974	1077	1.328	1.224	1.441	10.951	<0.0001
SOMA_P46108_CRK_4976_57	2209	1.639	1.513	1.775	0	32.923	1077	1.168	1.013	1.348	1.483	0.0329
SOMA_O75339_CILP_5717_2	2209	1.661	1.53	1.803	0	32.913	1077	1.096	0.955	1.259	0.715	0.1928
SOMA_Q8IZJ1_UNC5B_7776_20	2209	1.633	1.508	1.768	0	32.819	1077	1.430	1.306	1.565	14.064	<0.0001
SOMA_P05060_CHGB_8235_48	2209	1.689	1.55	1.84	0	32.39	1077	1.281	1.198	1.371	12.280	<0.0001
SOMA_Q99784_OLFM1_5703_26	2209	1.689	1.55	1.84	0	32.307	1077	1.071	1.003	1.143	1.393	0.0405
SOMA_P19827_ITIH1_7955_195	2209	0.6	0.552	0.653	0	32.27	1077	0.499	0.436	0.571	23.349	<0.0001
SOMA_Q9HD15_SRA1_18220_141	2209	1.682	1.545	1.832	0	32.2	1077	1.227	1.114	1.351	4.518	<0.0001
SOMA_O43251_RBFOX2_11462_8	2209	1.662	1.529	1.807	0	32.191	1077	1.053	0.986	1.125	0.911	0.1226
SOMA_P31415_CASQ1_11263_57	2209	0.587	0.538	0.641	0	32.187	1077	0.535	0.435	0.660	8.362	<0.0001
SOMA_P03952_KLKB1_4152_58	2209	0.6	0.551	0.652	0	32.083	1077	0.502	0.430	0.586	17.617	<0.0001
SOMA_O76076_WISP2_6392_7	2209	1.652	1.521	1.795	0	32.025	NA	NA	NA	NA	NA	NA
SOMA_O14791_APOL1_11510_31	2209	0.588	0.539	0.641	0	32.019	1077	0.490	0.418	0.575	17.851	<0.0001
SOMA_Q9Y3E2_BOLA1_15370_5	2209	1.642	1.513	1.782	0	31.914	1077	1.083	0.988	1.187	1.060	0.0871
SOMA_Q8IZJ1_UNC5B_15394_79	2209	1.625	1.5	1.76	0	31.913	1077	1.311	1.209	1.421	10.293	<0.0001
SOMA_Q07960_ARHGAP1_11955_1	2209	1.616	1.493	1.749	0	31.908	1077	0.992	0.845	1.164	0.036	0.9201
SOMA_Q9GZV9_FGF23_3807_1	2209	1.665	1.531	1.811	0	31.866	1077	1.401	1.310	1.499	22.067	<0.0001

SOMA_P01160_NPPA_5443_62	2209	1.692	1.551	1.845	0	31.818	1077	1.613	1.479	1.760	26.244	<0.0001
SOMA_Q12907_LMAN2_7638_30	2209	0.597	0.548	0.65	0	31.786	1077	0.295	0.227	0.384	18.956	<0.0001
SOMA_Q99727_TIMP4_6462_12	2209	1.685	1.546	1.836	0	31.747	1077	1.304	1.190	1.429	7.880	<0.0001
SOMA_Q9NTX7_RNF146_11401_181	2209	0.622	0.575	0.673	0	31.744	1077	0.000	0.000	0.000	12.096	<0.0001
SOMA_P29353_SHC1_5272_55	2209	1.622	1.497	1.757	0	31.68	1077	0.947	0.818	1.097	0.329	0.4692
SOMA_O75015_FCGR3B_3311_27	2209	1.7	1.557	1.856	0	31.603	1077	1.612	1.477	1.760	25.918	<0.0001
SOMA_Q8WXI7_MUC16_15565_102	2209	1.678	1.54	1.829	0	31.411	1077	1.063	1.002	1.127	1.381	0.0416
SOMA_P0DJI8_SAA1_15515_2	2209	1.675	1.537	1.826	0	31.227	1077	1.572	1.452	1.702	28.311	<0.0001
SOMA_Q9BR61_ACBD6_19341_36	2209	1.646	1.514	1.788	0	31.129	1077	1.136	0.978	1.319	1.026	0.0941
SOMA_Q9NVD7_PARVA_13434_172	2209	1.589	1.471	1.717	0	31.023	1077	1.107	1.053	1.163	4.160	<0.0001
SOMA_P14555_PLA2G2A_2692_74	2209	1.682	1.542	1.835	0	31.02	1077	1.305	1.225	1.390	15.785	<0.0001
SOMA_P13987_CD59_11514_196	2209	1.563	1.45	1.684	0	30.929	1077	1.421	1.313	1.538	17.523	<0.0001
SOMA_O00548_DLL1_5349_69	2209	1.665	1.529	1.813	0	30.919	1077	1.320	1.227	1.421	12.965	<0.0001
SOMA_Q9H3U7_SMOC2_15635_4	2209	1.646	1.514	1.79	0	30.763	1077	1.296	1.215	1.383	14.469	<0.0001
SOMA_P09211_GSTP1_4911_49	2209	1.695	1.551	1.852	0	30.674	1077	1.136	1.000	1.291	1.301	0.05
SOMA_P51858_HDGF_16758_96	2209	1.643	1.511	1.786	0	30.617	1077	1.124	1.049	1.204	3.069	0.0009
SOMA_O95998_IL18BP_3073_51	2209	1.638	1.508	1.78	0	30.54	1077	1.500	1.379	1.631	20.519	<0.0001
SOMA_Q76M96_CCDC80_3234_23	2209	1.654	1.519	1.801	0	30.32	1077	1.267	1.180	1.360	10.250	<0.0001
SOMA_P68402_PAFAH1B_2642_4	2209	1.594	1.473	1.725	0	30.31	1077	1.092	1.022	1.167	2.016	0.0096

SOMA_P11597_CETP_7131_207	2209	1.685	1.543	1.842	0	30.167	NA	NA	NA	NA	NA	NA
SOMA_Q9BUJ0_ABHD14A_5715_4	2209	1.626	1.497	1.766	0	30.115	1077	1.093	0.990	1.206	1.112	0.0774
SOMA_Q9UII4_HERC5_12934_1	2209	0.62	0.571	0.672	0	30.028	1077	0.634	0.482	0.832	2.980	0.001
SOMA_O00478_BTN3A3_17692_2	2209	1.643	1.51	1.788	0	30.014	1077	1.172	1.095	1.254	5.338	<0.0001
SOMA_Q9H5V8_CDCP1_16818_200	2209	1.597	1.475	1.729	0	29.967	1077	1.349	1.237	1.473	10.774	<0.0001
SOMA_Q07820_MCL1_10396_6	2209	1.6	1.477	1.734	0	29.915	1077	1.085	0.995	1.183	1.184	0.0655
SOMD O14793 GDF11 M 2765 4 PASS	2209	0.588	0.537	0.644	0	29.871	NA	NA	NA	NA	NA	NA
SOMD O95390 GDF11 M 2765 4 PASS	2209	0.588	0.537	0.644	0	29.871	NA	NA	NA	NA	NA	NA
SOMA_Q96GL9_FAM163A_6260_14	2209	1.661	1.523	1.811	0	29.809	NA	NA	NA	NA	NA	NA
SOMA_Q13308_PTK7_9525_1	2209	1.656	1.519	1.805	0	29.765	1077	1.551	1.430	1.683	25.398	<0.0001
SOMA_Q96EP1_CHFR_11320_29	2209	0.623	0.574	0.675	0	29.751	1077	0.370	0.257	0.532	7.085	<0.0001
SOMA_Q7LFX5_CHST15_4469_78	2209	1.663	1.525	1.814	0	29.69	1077	1.625	1.483	1.780	24.750	<0.0001
SOMA_P48740_MASP1_3605_77	2209	0.626	0.578	0.678	0	29.672	1077	0.426	0.246	0.737	2.642	0.0023
SOMA_O14793_MSTN_14583_49	2209	0.607	0.557	0.661	0	29.643	1077	0.896	0.703	1.144	0.421	0.3789
SOMA_P52943_CRIP2_9053_16	2209	1.612	1.485	1.749	0	29.616	1077	1.067	0.993	1.148	1.107	0.0781
SOMA_O00300_TNFRSF1_8304_50	2209	1.649	1.513	1.796	0	29.562	1077	1.508	1.360	1.672	14.180	<0.0001
SOMA_P21246_PTN_3045_72	2209	1.605	1.48	1.74	0	29.547	1077	1.277	1.164	1.400	6.680	<0.0001
SOMA_O95185_UNC5C_5139_32	2209	1.661	1.522	1.812	0	29.496	1077	1.523	1.367	1.697	13.618	<0.0001
SOMA_P55291_CDH15_5410_53	2209	0.631	0.583	0.683	0	29.468	1077	0.730	0.549	0.970	1.519	0.0303

SOMA_Q9Y5U2_TSSC4_18181_2	2209	1.582	1.462	1.712	0	29.451	1077	0.999	0.865	1.155	0.003	0.992
SOMA_P05413_FABP3_5437_63	2209	1.631	1.499	1.773	0	29.432	1077	1.452	1.333	1.583	16.814	<0.0001
SOMA_O43155_FLRT2_13122_19	2209	1.623	1.493	1.764	0	29.334	1077	1.437	1.314	1.573	14.545	<0.0001
SOMA_P39900_MMP12_4496_60	2209	1.669	1.528	1.823	0	29.282	1077	1.196	1.074	1.331	2.969	0.0011
SOMA_P22466_GAL_13389_8	2209	0.657	0.611	0.706	0	29.279	NA	NA	NA	NA	NA	NA
SOMA_P63302_SEPW1_18310_26	2209	1.574	1.456	1.702	0	29.27	NA	NA	NA	NA	NA	NA
SOMA_Q93045_STMN2_10900_272	2209	1.615	1.487	1.755	0	29.224	1077	1.068	0.983	1.160	0.927	0.1183
SOMA_Q07021_C1QBP_4967_1	2209	0.629	0.58	0.681	0	29.148	1077	1.041	0.949	1.142	0.402	0.3966
SOMA_Q9BT09_CNPY3_17327_3	2209	1.636	1.503	1.781	0	29.143	1077	1.351	1.248	1.464	12.805	<0.0001
SOMA_O75475_PSIP1_17176_13	2209	1.614	1.486	1.753	0	29.053	1077	1.196	1.112	1.287	5.866	<0.0001
SOMA_P00747_PLG_4151_6	2209	0.641	0.593	0.692	0	28.983	1077	0.481	0.421	0.550	25.914	<0.0001
SOMA_P51884_LUM_13114_50	2209	1.646	1.509	1.794	0	28.894	1077	1.283	1.159	1.420	5.792	<0.0001
SOMA_Q96KN2_CNDP1_5456_59	2209	0.603	0.553	0.659	0	28.873	1077	0.534	0.451	0.633	12.458	<0.0001
SOMA_Q9NR28_DIABLO_3122_6	2209	1.587	1.464	1.719	0	28.83	1077	1.083	1.005	1.167	1.436	0.0366
SOMA_Q14449_GRB14_13628_58	2209	0.618	0.569	0.672	0	28.722	1077	0.846	0.676	1.060	0.837	0.1457
SOMA_P19174_PLCG1_4563_61	2209	0.68	0.636	0.728	0	28.72	1077	0.967	0.767	1.218	0.111	0.7741
SOMA_O75473_LGR5_16296_43	2209	0.65	0.603	0.701	0	28.71	1077	0.919	0.603	1.401	0.158	0.6956
SOMA_Q6PUV4_CPLX2_15321_8	2209	1.625	1.493	1.768	0	28.671	1077	1.185	1.113	1.261	6.916	<0.0001
SOMA_P37023_ACVRL1_16318_12	2209	1.582	1.46	1.713	0	28.662	1077	1.070	1.009	1.136	1.623	0.0238

SOMA_Q6UX46_FAM150B_6284_7	2209	1.598	1.472	1.734	0	28.569	NA	NA	NA	NA	NA	NA
SOMA_O60938_KERA_10758_2	2209	1.612	1.483	1.752	0	28.561	1077	1.510	1.404	1.624	27.751	<0.0001
SOMA_O75636_FCN3_5462_62	2209	0.661	0.615	0.711	0	28.537	1077	0.532	0.451	0.628	13.025	<0.0001
SOMA_Q8WVN6_SECTM1_13093_6	2209	1.651	1.512	1.801	0	28.537	1077	1.134	1.064	1.209	3.940	0.0001
SOMA_O43921_EFNA2_14124_6	2209	1.559	1.442	1.684	0	28.504	1077	1.283	1.191	1.383	10.188	<0.0001
SOMA_P04264_KRT1_9931_20	2209	1.636	1.501	1.783	0	28.503	1077	1.259	1.186	1.336	13.555	<0.0001
SOMA_Q9BXJ4_C1QTNF3_7251_64	2209	0.632	0.583	0.685	0	28.467	1077	0.302	0.235	0.388	20.203	<0.0001
SOMA_Q9NQX5_NPDC1_10424_31	2209	1.573	1.453	1.703	0	28.457	1077	1.157	1.096	1.220	7.018	<0.0001
SOMA_P0C7M6_IQCF3_13439_6	2209	0.638	0.59	0.69	0	28.436	1077	0.198	0.107	0.365	6.659	<0.0001
SOMA_O94856_NFASC_7179_69	2209	1.664	1.522	1.819	0	28.38	1077	1.327	1.233	1.429	13.368	<0.0001
SOMA_O00622_CYR61_6264_9	2209	1.597	1.472	1.734	0	28.368	NA	NA	NA	NA	NA	NA
SOMA_Q9Y3E7_VPS24_12508_9	2209	1.621	1.489	1.764	0	28.335	NA	NA	NA	NA	NA	NA
SOMA_P67809_YBX1_9751_72	2209	1.608	1.479	1.747	0	28.281	1077	1.350	1.234	1.478	10.154	<0.0001
SOMA_Q9UJZ1_STOML2_6555_58	2209	0.645	0.597	0.696	0	28.188	1077	1.051	0.924	1.194	0.347	0.4495
SOMA_Q01523_DEFA5_16785_45	2209	1.633	1.498	1.78	0	28.099	1077	1.230	1.127	1.344	5.399	<0.0001
SOMA_P22692_IGFBP4_2950_57	2209	1.497	1.394	1.607	0	28.079	1077	1.345	1.242	1.456	12.521	<0.0001
SOMA_Q16832_DDR2_15381_45	2209	1.589	1.464	1.724	0	28.048	1077	1.043	0.964	1.128	0.526	0.298
SOMA_P01033_TIMP1_2211_9	2209	1.637	1.501	1.785	0	28.045	1077	1.744	1.605	1.896	38.241	<0.0001
SOMA_O43915_FIGF_13098_93	2209	1.575	1.454	1.706	0	28.029	NA	NA	NA	NA	NA	NA

SOMA_Q68G75_LEMD1_8040_9	2209	1.597	1.471	1.735	0	27.947	1077	1.086	0.979	1.204	0.921	0.12
SOMA_Q9UN70_PCDHGC3_7859_21	2209	0.616	0.565	0.671	0	27.921	1077	0.653	0.501	0.851	2.799	0.0016
SOMA_Q9NR34_MAN1C1_13427_66	2209	1.52	1.411	1.636	0	27.745	1077	1.339	1.262	1.421	21.465	<0.0001
SOMA_P0DJI9_SAA2_18832_65	2209	1.622	1.489	1.767	0	27.702	1077	1.260	1.192	1.332	15.503	<0.0001
SOMA_P51888_PRELP_5675_6	2209	1.643	1.505	1.795	0	27.673	NA	NA	NA	NA	NA	NA
SOMA_Q99542_MMP19_6425_87	2209	1.633	1.497	1.781	0	27.654	1077	1.948	1.772	2.141	42.824	<0.0001
SOMA_P04070_PROC_2961_1	2209	0.612	0.561	0.667	0	27.642	1077	0.598	0.511	0.700	9.867	<0.0001
SOMA_P43307_SSR1_8106_15	2209	0.61	0.558	0.666	0	27.612	1077	0.009	0.003	0.027	16.489	<0.0001
SOMA_Q06033_ITIH3_7145_1	2209	1.593	1.467	1.731	0	27.581	1077	1.532	1.411	1.664	23.574	<0.0001
SOMA_P35968_KDR_3651_50	2209	0.628	0.578	0.682	0	27.569	1077	0.776	0.664	0.907	2.838	0.0015
SOMD P01876 IGHA1 I 11089 7 PASS	2209	1.611	1.48	1.753	0	27.496	NA	NA	NA	NA	NA	NA
SOMD P01877 IGHA1 I 11089 7 PASS	2209	1.611	1.48	1.753	0	27.496	NA	NA	NA	NA	NA	NA
SOMA_O14907_TAX1BP3_12498_12	2209	1.571	1.45	1.703	0	27.454	1077	1.066	0.926	1.228	0.426	0.3746
SOMA_P98179_RBM3_12747_89	2209	1.589	1.463	1.726	0	27.365	1077	1.115	0.964	1.289	0.844	0.1431
SOMA_P10451_SPP1_13113_7	2209	1.616	1.483	1.76	0	27.285	1077	1.139	1.073	1.208	4.735	<0.0001
SOMD P06732 CKB CKM 3714 49 PASS	2209	0.614	0.562	0.67	0	27.246	NA	NA	NA	NA	NA	NA
SOMD P12277 CKB CKM 3714 49 PASS	2209	0.614	0.562	0.67	0	27.246	NA	NA	NA	NA	NA	NA
SOMA_P58335_ANTXR2_15559_5	2209	0.625	0.574	0.68	0	27.227	1077	0.512	0.427	0.613	12.355	<0.0001
SOMA_Q86VZ4_LRP11_6713_4	2209	1.621	1.487	1.767	0	27.225	1077	1.335	1.230	1.449	11.367	<0.0001

SOMA_Q08708_CD300C_5066_134	2209	1.626	1.49	1.774	0	27.183	1077	1.318	1.234	1.408	15.574	<0.0001
SOMA_Q9C002_NMES1_6406_3	2209	0.603	0.55	0.66	0	27.151	1077	0.462	0.359	0.596	8.559	<0.0001
SOMA_Q13291_SLAMF1_7953_20	2209	1.577	1.453	1.711	0	27.084	1077	1.051	0.978	1.130	0.755	0.1756
SOMA_Q2Y0W8_SLC4A8_12798_46	2209	0.632	0.582	0.686	0	27.044	1077	1.011	0.886	1.154	0.062	0.8668
SOMA_P14621_ACYP2_12812_25	2209	1.594	1.466	1.733	0	27.04	1077	1.143	0.989	1.320	1.157	0.0697
SOMA_P28325_CST5_3803_10	2209	1.615	1.482	1.761	0	27.037	1077	1.169	1.101	1.240	6.620	<0.0001
SOMA_O75356_ENTPD5_4437_56	2209	0.628	0.578	0.683	0	27.035	1077	0.505	0.423	0.603	13.443	<0.0001
SOMA_Q8WU39_PACAP_16322_10	2209	1.608	1.476	1.751	0	27.003	NA	NA	NA	NA	NA	NA
SOMA_P14778_IL1R1_2991_9	2209	1.623	1.487	1.77	0	26.99	1077	1.672	1.529	1.828	28.654	<0.0001
SOMA_P06401_PGR_15693_9	2209	1.577	1.453	1.711	0	26.95	1077	1.187	1.044	1.350	2.063	0.0086
SOMA_P10721_KIT_2475_1	2209	0.614	0.562	0.67	0	26.885	1077	0.629	0.532	0.744	7.214	<0.0001
SOMA_Q15485_FCN2_13717_15	2209	0.635	0.585	0.689	0	26.856	1077	0.695	0.597	0.808	5.660	<0.0001
SOMA_P08571_CD14_8969_49	2209	1.611	1.479	1.756	0	26.8	1077	1.132	1.074	1.193	5.391	<0.0001
SOMA_Q8NBM8_PCYOX1L_5599_88	2209	1.579	1.454	1.714	0	26.759	1077	1.084	0.993	1.184	1.138	0.0728
SOMA_P02765_AHSG_3581_53	2209	0.644	0.595	0.697	0	26.755	1077	0.948	0.796	1.128	0.261	0.5484
SOMA_P55055_NR1H2_9016_12	2209	0.645	0.596	0.699	0	26.751	1077	0.579	0.421	0.795	3.142	0.0007
SOMA_Q8TC05_MDM1_7898_29	2209	1.542	1.426	1.668	0	26.737	1077	1.026	0.917	1.147	0.185	0.6537
SOMA_Q96R05_RBP7_14208_3	2209	1.555	1.435	1.684	0	26.554	1077	1.466	1.355	1.587	20.613	<0.0001
SOMA_P52803_EFNA5_2615_60	2209	1.558	1.437	1.688	0	26.55	1077	1.287	1.192	1.390	9.997	<0.0001

SOMA_P55083_MFAP4_5636_10	2209	1.597	1.467	1.738	0	26.533	1077	1.478	1.351	1.617	16.912	<0.0001
SOMA_P00734_F2_4157_2	2209	0.631	0.58	0.686	0	26.501	1077	0.271	0.164	0.449	6.432	<0.0001
SOMA_P24821_TNC_4155_3	2209	1.622	1.486	1.771	0	26.42	1077	1.712	1.563	1.875	30.173	<0.0001
SOMA_Q9Y5C1_ANGPTL3_10391_1	2209	1.581	1.454	1.718	0	26.375	1077	1.588	1.420	1.777	15.203	<0.0001
SOMA_P19429_TNNI3_5441_67	2209	1.548	1.429	1.676	0	26.313	1077	1.038	0.943	1.142	0.354	0.4422
SOMA_P15291_B4GALT1_13381_49	2209	1.603	1.471	1.747	0	26.304	1077	1.641	1.509	1.784	30.438	<0.0001
SOMA_Q16352_INA_11436_6	2209	1.569	1.445	1.703	0	26.242	1077	1.105	1.033	1.182	2.450	0.0036
SOMA_P54760_EPHB4_15530_33	2209	1.546	1.428	1.674	0	26.203	1077	1.448	1.337	1.568	19.038	<0.0001
SOMA_Q99435_NELL2_6022_57	2209	1.546	1.428	1.673	0	26.2	1077	1.084	1.023	1.147	2.228	0.0059
SOMA_Q9Y274_ST3GAL6_6947_4	2209	0.637	0.587	0.692	0	26.156	1077	0.602	0.527	0.689	12.983	<0.0001
SOMA_Q99969_RARRES2_3079_62	2209	1.58	1.453	1.718	0	26.035	1077	1.483	1.303	1.688	8.615	<0.0001
SOMA_P59901_LILRA4_8299_66	2209	0.636	0.585	0.691	0	25.956	1077	0.410	0.302	0.557	7.972	<0.0001
SOMA_Q8IYJ0_PIANP_9599_6	2209	1.556	1.435	1.687	0	25.939	1077	1.098	1.041	1.158	3.198	0.0006
SOMA_Q96T51_RUFY1_11425_31	2209	0.685	0.639	0.734	0	25.929	1077	0.405	0.291	0.564	7.067	<0.0001
SOMA_Q15848_ADIPOQ_3554_24	2209	1.62	1.483	1.769	0	25.921	1077	1.627	1.484	1.784	24.359	<0.0001
SOMA_Q8IZS8_CACNA2D_8885_6	2209	0.637	0.586	0.692	0	25.909	1077	0.668	0.558	0.798	5.028	<0.0001
SOMA_Q6ZTQ4_CDHR3_8222_49	2209	0.631	0.58	0.687	0	25.833	1077	0.942	0.757	1.173	0.226	0.5942
SOMA_O00339_MATN2_3325_2	2209	1.587	1.458	1.727	0	25.815	1077	1.461	1.329	1.606	14.423	<0.0001
SOMA_Q9UKK6_NXT1_9942_2	2209	1.56	1.437	1.693	0	25.814	1077	1.103	1.030	1.182	2.280	0.0052

SOMA_P82980_RBP5_19241_31	2209	1.587	1.458	1.728	0	25.809	1077	1.395	1.295	1.502	17.775	<0.0001
SOMA_Q9BXY4_RSPO3_8427_118	2209	1.578	1.451	1.716	0	25.802	1077	1.179	1.079	1.289	3.538	0.0003
SOMA_P52799_EFNB2_14131_37	2209	1.534	1.418	1.659	0	25.776	1077	1.264	1.178	1.356	10.166	<0.0001
SOMA_Q13283_G3BP1_19266_35	2209	1.566	1.442	1.701	0	25.756	1077	0.983	0.851	1.135	0.090	0.8119
SOMA_Q92743_HTRA1_15594_47	2209	1.613	1.477	1.761	0	25.719	1077	1.583	1.442	1.738	21.226	<0.0001
SOMA_Q8N2K1_UBE2J2_8802_24	2209	1.562	1.438	1.696	0	25.665	NA	NA	NA	NA	NA	NA
SOMA_Q9H492_MAP1LC3_17781_191	2209	1.579	1.452	1.718	0	25.649	1077	1.384	1.210	1.582	5.669	<0.0001
SOMA_Q9UGN4_CD300A_5630_48	2209	1.571	1.445	1.707	0	25.616	1077	1.491	1.371	1.621	20.113	<0.0001
SOMA_Q6EMK4_VASN_5682_13	2209	1.609	1.474	1.756	0	25.614	1077	1.671	1.521	1.836	25.967	<0.0001
SOMA_P01563_IFNA2_3497_13	2209	0.711	0.667	0.757	0	25.576	1077	0.832	0.667	1.038	0.987	0.103
SOMA_P02748_C9_3060_43	2209	1.568	1.443	1.704	0	25.557	1077	1.985	1.716	2.296	19.618	<0.0001
SOMA_P10092_CALCB_17170_15	2209	1.555	1.433	1.687	0	25.479	1077	1.035	0.954	1.124	0.391	0.4063
SOMA_Q8IVM0_CCDC50_12399_194	2209	1.55	1.429	1.681	0	25.419	1077	0.980	0.833	1.153	0.091	0.8104
SOMA_O15525_MAFG_19281_86	2209	1.567	1.442	1.703	0	25.393	1077	1.100	1.040	1.164	3.036	0.0009
SOMA_Q96E93_KLRG1_10548_35	2209	1.584	1.454	1.726	0	25.276	NA	NA	NA	NA	NA	NA
SOMA_Q9H8J5_MANSC1_9557_5	2209	1.581	1.452	1.722	0	25.236	1077	1.477	1.361	1.602	20.289	<0.0001
SOMA_Q9H4I3_TRABD_11262_39	2209	0.637	0.585	0.693	0	25.202	1077	0.648	0.439	0.957	1.533	0.0293
SOMA_P10082_PYY_3727_35	2209	1.581	1.452	1.722	0	25.197	1077	1.576	1.431	1.735	19.680	<0.0001
SOMA_Q16661_GUCA2B_6223_5	2209	1.572	1.445	1.71	0	25.149	1077	1.103	1.023	1.191	1.950	0.0112

SOMA_P19957_PI3_498254	2209	1.58	1.451	1.721	0	25.143	1077	1.365	1.256	1.484	12.616	<0.0001
SOMA_O94813_SLIT2_18930_28	2209	1.547	1.426	1.678	0	25.08	1077	1.111	1.045	1.181	3.129	0.0007
SOMA_O15400_STX7_8274_64	2209	1.526	1.41	1.652	0	24.974	1077	1.019	0.897	1.157	0.110	0.7767
SOMA_O95150_TNFSF15_2968_61	2209	1.577	1.448	1.718	0	24.858	1077	1.198	1.121	1.279	7.095	<0.0001
SOMA_P19404_NDUFV2_7748_11	2209	1.566	1.44	1.704	0	24.831	1077	1.216	1.106	1.337	4.299	<0.0001
SOMA_P04179_SOD2_5008_51	2209	0.626	0.573	0.683	0	24.827	1077	0.454	0.352	0.584	9.025	<0.0001
SOMA_Q9BXY4_RSPO3_13094_75	2209	1.547	1.425	1.679	0	24.816	1077	1.121	1.039	1.210	2.474	0.0034
SOMA_Q86UL8_MAGI2_14066_49	2209	1.552	1.428	1.686	0	24.502	1077	0.940	0.738	1.198	0.209	0.6181
SOMA_Q8IYJ0_PIANP_14114_18	2209	1.52	1.404	1.645	0	24.428	1077	1.102	1.038	1.170	2.817	0.0015
SOMA_Q155Q3_DIXDC1_13441_30	2209	0.636	0.583	0.693	0	24.278	1077	0.969	0.818	1.148	0.144	0.7175
SOMA_Q96RJ3_TNFRSF1_5383_14	2209	0.632	0.58	0.69	0	24.261	1077	1.053	0.983	1.128	0.847	0.1423
SOMA_Q6UY11_DLK2_9359_9	2209	1.551	1.427	1.685	0	24.254	1077	1.302	1.211	1.401	11.897	<0.0001
SOMA_Q01449_MYL7_19296_51	2209	0.638	0.585	0.695	0	24.247	1077	0.433	0.366	0.511	22.186	<0.0001
SOMA_P36776_LONP1_6398_12	2209	1.523	1.406	1.65	0	24.245	1077	0.793	0.676	0.931	2.347	0.0045
SOMA_Q86TD4_SRL_10940_25	2209	1.542	1.42	1.674	0	24.245	1077	1.247	1.162	1.338	9.088	<0.0001
SOMA_Q96D42_HAVCR1_9021_1	2209	1.546	1.423	1.679	0	24.182	1077	1.035	0.932	1.148	0.281	0.523
SOMA_Q9NQ30_ESM1_3805_16	2209	1.577	1.446	1.72	0	24.052	1077	1.406	1.299	1.522	16.417	<0.0001
SOMA_O75821_EIF3G_11454_87	2209	1.532	1.412	1.662	0	24.048	1077	0.996	0.862	1.151	0.020	0.9553
SOMA_P56704_WNT3A_13236_25	2209	0.657	0.607	0.712	0	23.884	1077	0.440	0.343	0.565	9.966	<0.0001

SOMA_Q9UN86_G3BP2_9831_12	2209	1.526	1.407	1.654	0	23.865	1077	1.060	0.916	1.226	0.361	0.4351
SOMA_Q9NX14_NDUFB11_7747_47	2209	0.666	0.616	0.72	0	23.76	1077	0.430	0.290	0.638	4.572	<0.0001
SOMA_P23280_CA6_3352_80	2209	0.635	0.582	0.693	0	23.751	1077	0.399	0.311	0.513	12.143	<0.0001
SOMA_Q8N6G6_ADAMTSL_16890_37	2209	1.542	1.419	1.676	0	23.647	1077	1.421	1.268	1.592	8.872	<0.0001
SOMA_O75436_VPS26A_19293_6	2209	1.512	1.396	1.637	0	23.629	1077	0.888	0.768	1.026	0.975	0.1059
SOMA_P20800_EDN2_12574_36	2209	1.511	1.396	1.637	0	23.601	1077	1.113	1.031	1.202	2.200	0.0063
SOMA_O94768_STK17B_5249_31	2209	0.662	0.612	0.717	0	23.48	1077	0.981	0.834	1.154	0.090	0.8136
SOMA_Q8IZT8_HS3ST5_10731_10	2209	0.639	0.586	0.697	0	23.459	1077	0.216	0.131	0.356	8.719	<0.0001
SOMA_P49961_ENTPD1_7999_23	2209	1.53	1.409	1.662	0	23.429	1077	1.120	1.062	1.181	4.536	<0.0001
SOMA_Q14116_IL18_5661_15	2209	1.545	1.42	1.68	0	23.414	1077	1.118	1.039	1.203	2.527	0.003
SOMA_P21815_IBSP_3415_61	2209	1.582	1.447	1.729	0	23.337	1077	1.478	1.379	1.583	27.753	<0.0001
SOMA_P15692_VEGFA_2597_8	2209	1.459	1.356	1.569	0	23.336	1077	1.495	1.377	1.623	20.994	<0.0001
SOMA_P12110_COL6A2_16753_46	2209	1.539	1.416	1.673	0	23.335	1077	1.166	1.112	1.222	9.865	<0.0001
SOMA_O95881_TXNDC12_19334_62	2209	1.535	1.412	1.668	0	23.226	1077	0.917	0.795	1.058	0.630	0.2345
SOMA_Q92804_TAF15_16865_62	2209	1.524	1.404	1.654	0	23.178	1077	1.062	0.923	1.222	0.395	0.403
SOMA_P84090_ERH_11614_29	2209	1.523	1.403	1.653	0	23.116	1077	1.079	0.966	1.206	0.749	0.1784
SOMA_Q5EE01_CENPW_8864_59	2209	0.665	0.614	0.72	0	23.105	1077	0.326	0.142	0.750	2.078	0.0084
SOMA_Q9Y680_FKBP7_9288_7	2209	1.486	1.376	1.605	0	23.096	1077	0.997	0.861	1.154	0.015	0.9657
SOMA_P39656_DDOST_17161_1	2209	1.517	1.398	1.645	0	23.026	1077	1.212	1.053	1.394	2.145	0.0072

SOMA_P01588_EPO_5813_58	2209	1.575	1.442	1.722	0	22.978	1077	1.283	1.221	1.348	22.382	<0.0001
SOMA_P19823_ITIH2_9326_33	2209	0.642	0.589	0.7	0	22.944	1077	0.626	0.547	0.717	10.964	<0.0001
SOMA_P20774_OGN_17224_12	2209	1.529	1.407	1.662	0	22.846	1077	1.127	1.037	1.225	2.314	0.0049
SOMA_P15529_CD46_17682_1	2209	1.556	1.427	1.697	0	22.84	1077	1.251	1.157	1.353	7.735	<0.0001
SOMA_Q4KMG0_CDON_4541_49	2209	0.647	0.594	0.705	0	22.834	1077	0.675	0.568	0.803	5.068	<0.0001
SOMA_Q9NUQ9_FAM49B_19176_27	2209	1.524	1.403	1.655	0	22.81	NA	NA	NA	NA	NA	NA
SOMA_O00253_AGRP_2813_11	2209	1.54	1.415	1.676	0	22.787	1077	1.088	1.035	1.143	3.041	0.0009
SOMA_P84157_MXRA7_8005_1	2209	1.554	1.425	1.695	0	22.719	1077	1.201	1.119	1.289	6.410	<0.0001
SOMA_Q8NBJ4_GOLM1_8983_7	2209	1.528	1.406	1.661	0	22.668	1077	1.061	0.985	1.143	0.925	0.1189
SOMA_Q08999_RBL2_13565_2	2209	1.503	1.387	1.629	0	22.65	1077	1.434	1.315	1.564	15.369	<0.0001
SOMA_Q9BYB0_SHANK3_13242_134	2209	1.508	1.391	1.635	0	22.628	1077	0.867	0.740	1.015	1.120	0.0758
SOMA_P55773_CCL23_2913_1	2209	1.578	1.443	1.727	0	22.558	1077	1.423	1.335	1.518	26.184	<0.0001
SOMA_Q9UNI1_CELA1_6107_3	2209	1.547	1.419	1.686	0	22.469	1077	1.779	1.567	2.019	18.297	<0.0001
SOMA_P09972_ALDOC_9876_20	2209	0.641	0.587	0.7	0	22.452	1077	1.075	0.933	1.239	0.497	0.3184
SOMA_Q9UKP5_ADAMTS6_6441_62	2209	1.514	1.395	1.644	0	22.312	1077	1.097	0.978	1.232	0.942	0.1143
SOMA_O14810_CPLX1_18332_17	2209	1.527	1.404	1.661	0	22.301	1077	1.051	0.979	1.128	0.763	0.1724
SOMA_Q7Z5A7_FAM19A5_5609_92	2209	1.516	1.396	1.646	0	22.295	NA	NA	NA	NA	NA	NA
SOMA_O00453_LST1_9531_24	2209	1.517	1.396	1.648	0	22.275	1077	1.270	1.152	1.401	5.799	<0.0001
SOMA_Q14392_LRRC32_7551_33	2209	1.524	1.401	1.657	0	22.267	1077	1.049	0.980	1.122	0.777	0.1669

SOMA_Q7Z2W9_MRPL21_8942_2	2209	0.646	0.593	0.705	0	22.245	1077	0.548	0.424	0.708	5.392	<0.0001
SOMA_Q92974_ARHGEF2_12848_9	2209	0.663	0.611	0.719	0	22.16	1077	0.852	0.599	1.211	0.430	0.3715
SOMA_O15075_DCLK1_17156_72	2209	1.511	1.392	1.64	0	22.149	1077	1.195	1.115	1.281	6.338	<0.0001
SOMA_P58340_MLF1_17820_170	2209	0.656	0.603	0.713	0	22.092	1077	0.677	0.482	0.952	1.606	0.0248
SOMA_Q15121_PEA15_18833_76	2209	1.514	1.394	1.644	0	22.072	1077	1.093	1.032	1.156	2.653	0.0022
SOMA_Q9UBP4_DKK3_3607_71	2209	1.536	1.41	1.673	0	22.033	1077	1.296	1.214	1.384	13.924	<0.0001
SOMA_O76095_JTB_9038_12	2209	1.51	1.391	1.64	0	21.974	1077	1.188	1.115	1.265	7.075	<0.0001
SOMA_O60260_PARK2_13013_41	2209	0.747	0.704	0.792	0	21.971	NA	NA	NA	NA	NA	NA
SOMA_Q14195_DPYSL3_12707_26	2209	0.65	0.596	0.708	0	21.962	1077	0.795	0.663	0.952	1.892	0.0128
SOMA_P53814_SMTN_12546_1	2209	1.5	1.383	1.627	0	21.955	1077	0.829	0.610	1.127	0.635	0.2319
SOMA_Q8TDY8_IGDCC4_9793_145	2209	0.644	0.59	0.704	0	21.933	1077	0.692	0.487	0.984	1.391	0.0406
SOMA_P58499_FAM3B_9177_6	2209	1.514	1.393	1.645	0	21.861	1077	1.370	1.248	1.503	10.464	<0.0001
SOMA_Q6UXN2_TREML4_11139_4	2209	1.532	1.406	1.669	0	21.834	NA	NA	NA	NA	NA	NA
SOMA_P01042_KNG1_15343_337	2209	0.674	0.623	0.73	0	21.729	1077	0.388	0.294	0.512	10.697	<0.0001
SOMA_P23443_RPS6KB1_8962_48	2209	0.661	0.608	0.719	0	21.641	1077	0.287	0.184	0.447	7.499	<0.0001
SOMA_P18850_ATF6_11277_23	2209	0.654	0.601	0.713	0	21.598	1077	0.831	0.709	0.973	1.661	0.0218
SOMA_Q9NZ72_STMN3_8019_73	2209	1.482	1.369	1.605	0	21.547	1077	1.085	1.020	1.156	1.986	0.0103
SOMA_P07711_CTSL_9275_2	2209	1.526	1.401	1.662	0	21.532	NA	NA	NA	NA	NA	NA
SOMA_Q9UBC7_GALP_9398_30	2209	0.789	0.752	0.828	0	21.525	1077	0.097	0.022	0.421	2.729	0.0019

SOMA_Q15485_FCN2_3313_21	2209	0.661	0.608	0.719	0	21.454	1077	0.996	0.856	1.159	0.019	0.9571
SOMA_Q9P0K1_ADAM22_7933_75	2209	0.678	0.627	0.734	0	21.438	1077	0.998	0.852	1.169	0.007	0.9837
SOMA_P22748_CA4_14069_61	2209	0.66	0.607	0.718	0	21.429	1077	0.046	0.016	0.131	8.203	<0.0001
SOMA_O95990_FAM107A_2760_2	2209	0.784	0.747	0.824	0	21.414	1077	0.815	0.554	1.198	0.527	0.2971
SOMA_Q7Z408_CSMD2_9971_5	2209	1.409	1.314	1.51	0	21.397	1077	1.051	0.919	1.201	0.330	0.4682
SOMA_P05161_ISG15_14151_4	2209	1.503	1.384	1.633	0	21.391	1077	1.122	1.010	1.246	1.505	0.0313
SOMA_P45877_PPIC_18819_21	2209	1.504	1.385	1.634	0	21.376	1077	1.263	1.144	1.395	5.414	<0.0001
SOMA_O75462_CRLF1_14747_9	2209	1.495	1.378	1.622	0	21.373	1077	1.077	0.995	1.166	1.169	0.0677
SOMA_Q9UPQ9_TNRC6B_9808_41	2209	1.484	1.37	1.608	0	21.317	1077	1.078	0.945	1.229	0.580	0.2629
SOMA_P48052_CPA2_9276_7	2209	0.777	0.739	0.818	0	21.295	1077	0.002	0.000	0.013	8.773	<0.0001
SOMA_Q6UWE3_CLPSL2_7767_1	2209	0.689	0.639	0.743	0	21.263	1077	0.492	0.328	0.739	3.203	0.0006
SOMA_P20618_PSMB1_12612_37	2209	1.441	1.337	1.552	0	21.21	1077	1.359	1.238	1.493	9.848	<0.0001
SOMA_Q13126_MTAP_9910_9	2209	0.675	0.623	0.731	0	21.204	1077	0.313	0.160	0.610	3.191	0.0006
SOMA_Q8N6M8_IQCF1_7991_54	2209	0.679	0.628	0.735	0	21.189	1077	0.267	0.175	0.407	9.080	<0.0001
SOMA_Q8N6T3_ARFGAP1_11556_19	2209	0.658	0.604	0.716	0	21.156	1077	0.418	0.323	0.540	10.587	<0.0001
SOMA_Q86Z14_KLB_19557_3	2209	0.656	0.602	0.715	0	21.121	1077	0.730	0.592	0.900	2.500	0.0032
SOMA_Q9NZC2_TREM2_16300_4	2209	1.482	1.368	1.606	0	21.117	1077	1.474	1.318	1.649	10.922	<0.0001
SOMA_P07360_C8G_14708_59	2209	0.661	0.607	0.719	0	21.028	1077	0.782	0.675	0.905	2.998	0.001
SOMA_Q8TBE7_SLC35G2_13501_10	2209	0.655	0.601	0.714	0	21.025	1077	0.368	0.259	0.523	7.604	<0.0001

SOMA_P08709_F7_3184_25	2209	0.663	0.61	0.721	0	21.008	1077	0.528	0.449	0.621	13.981	<0.0001
SOMA_Q9Y2X8_UBE2D4_13475_10	2209	1.5	1.381	1.63	0	20.998	1077	0.941	0.813	1.090	0.378	0.419
SOMA_Q8WYQ3_CHCHD10_11270_17	2209	1.489	1.373	1.616	0	20.977	1077	1.070	0.970	1.181	0.760	0.1736
SOMA_Q9UHG2_PCSK1N_9391_60	2209	1.485	1.37	1.611	0	20.934	1077	1.020	0.914	1.138	0.139	0.7261
SOMA_P42830_CXCL5_2979_8	2209	0.785	0.747	0.825	0	20.927	1077	1.047	0.945	1.161	0.420	0.3799
SOMA_Q96NZ8_WFIKKN1_3191_50	2209	0.715	0.668	0.766	0	20.91	1077	0.864	0.650	1.148	0.505	0.3126
SOMA_P02647_APOA1_2750_3	2209	0.676	0.624	0.733	0	20.896	1077	0.476	0.405	0.560	18.721	<0.0001
SOMA_Q96MM7_HS6ST2_13524_25	2209	1.507	1.386	1.64	0	20.888	1077	1.441	1.336	1.554	20.411	<0.0001
SOMA_Q7L1S5_CHST9_11646_4	2209	1.514	1.391	1.649	0	20.86	1077	1.055	0.980	1.137	0.814	0.1533
SOMA_Q49AH0_CDNF_4962_52	2209	1.492	1.374	1.62	0	20.846	1077	1.152	1.089	1.218	6.164	<0.0001
SOMA_Q6IN85_SMEK1_10866_60	2209	1.501	1.381	1.632	0	20.846	NA	NA	NA	NA	NA	NA
SOMA_Q93070_ART4_6576_1	2209	1.522	1.396	1.659	0	20.811	1077	1.380	1.288	1.479	19.107	<0.0001
SOMA_P61201_COPS2_14029_42	2209	0.666	0.612	0.724	0	20.738	1077	0.493	0.394	0.618	9.080	<0.0001
SOMA_O15232_MATN3_19361_78	2209	1.454	1.346	1.571	0	20.708	1077	1.350	1.264	1.441	18.519	<0.0001
SOMA_P98160_HSPG2_15626_223	2209	1.525	1.398	1.664	0	20.703	1077	1.196	1.144	1.250	14.524	<0.0001
SOMA_P13726_F3_4931_59	2209	1.492	1.374	1.621	0	20.659	1077	1.109	1.056	1.164	4.510	<0.0001
SOMA_Q9NNZ3_DNAJC4_8653_132	2209	0.666	0.613	0.724	0	20.618	1077	0.665	0.540	0.820	3.879	0.0001
SOMA_P01298_PPY_4588_1	2209	1.506	1.383	1.639	0	20.59	1077	1.294	1.214	1.380	14.492	<0.0001
SOMA_Q5TAT6_COL13A1_6570_1	2209	0.655	0.6	0.715	0	20.47	1077	0.562	0.452	0.700	6.603	<0.0001

SOMA_Q9BYZ8_REG4_11102_22	2209	1.491	1.372	1.62	0	20.362	1077	1.315	1.178	1.467	5.991	<0.0001
SOMA_Q9C075_KRT23_14237_1	2209	0.667	0.613	0.726	0	20.345	NA	NA	NA	NA	NA	NA
SOMA_Q9Y5H2_PCDHGA1_6563_78	2209	1.492	1.373	1.622	0	20.32	NA	NA	NA	NA	NA	NA
SOMA_Q96AP7_ESAM_7841_84	2209	1.484	1.367	1.611	0	20.312	1077	1.356	1.220	1.507	7.833	<0.0001
SOMA_P35318_ADM_14115_34	2209	0.672	0.619	0.73	0	20.295	1077	0.546	0.440	0.677	7.438	<0.0001
SOMA_P18859_ATP5J_7788_1	2209	1.524	1.396	1.664	0	20.275	NA	NA	NA	NA	NA	NA
SOMA_Q9UHD9_UBQLN2_19121_3	2209	1.48	1.364	1.607	0	20.262	1077	0.986	0.852	1.142	0.069	0.8525
SOMA_Q9NZV1_CRIM1_15492_1	2209	1.485	1.367	1.613	0	20.2	1077	1.008	0.894	1.136	0.045	0.9006
SOMA_Q12846_STX4_10668_5	2209	1.446	1.339	1.562	0	20.186	1077	0.904	0.775	1.054	0.702	0.1987
SOMA_Q9NRR5_UBQLN4_12720_71	2209	1.497	1.375	1.629	0	20.057	1077	0.983	0.850	1.136	0.088	0.8162
SOMA_P51580_TPMT_11218_84	2209	0.669	0.615	0.728	0	20.025	1077	0.543	0.440	0.671	7.825	<0.0001
SOMA_Q9H6B4_CLMP_10440_26	2209	1.511	1.385	1.648	0	19.982	1077	1.064	0.977	1.158	0.815	0.1532
SOMA_Q9H9V4_RNF122_11160_56	2209	0.676	0.622	0.734	0	19.957	1077	0.560	0.437	0.718	5.338	<0.0001
SOMA_P09651_HNRNPA1_12466_7	2209	1.452	1.342	1.57	0	19.945	1077	1.029	0.895	1.182	0.161	0.6903
SOMA_Q9UBX7_KLK11_2831_29	2209	1.483	1.365	1.611	0	19.921	1077	1.335	1.255	1.420	19.439	<0.0001
SOMA_Q9H2A7_CXCL16_2436_49	2209	1.514	1.387	1.652	0	19.883	1077	1.486	1.362	1.621	18.263	<0.0001
SOMA_Q9UHG3_PCYOX1_6431_68	2209	0.671	0.617	0.73	0	19.843	1077	0.548	0.460	0.654	10.616	<0.0001
SOMA_P05026_ATP1B1_13392_13	2209	0.683	0.631	0.741	0	19.793	1077	0.567	0.479	0.670	10.447	<0.0001
SOMA_P42785_PRCP_5722_78	2209	0.674	0.62	0.732	0	19.751	1077	0.610	0.515	0.723	7.958	<0.0001

SOMA_Q92688_ANP32B_4194_26	2209	1.47	1.355	1.595	0	19.696	1077	1.148	1.081	1.220	5.104	<0.0001
SOMA_Q96DR5_BPIFA2_16302_11	2209	0.669	0.614	0.728	0	19.671	1077	0.411	0.325	0.521	12.835	<0.0001
SOMA_Q9UHF5_IL17B_3499_77	2209	0.808	0.773	0.846	0	19.61	1077	0.906	0.732	1.122	0.437	0.3655
SOMA_Q6UXB4_CLEC4G_10781_19	2209	1.494	1.372	1.627	0	19.532	1077	1.272	1.176	1.375	8.731	<0.0001
SOMD P0DML2 CSH1 CS 13103 125 PASS	2209	0.675	0.62	0.734	0	19.518	NA	NA	NA	NA	NA	NA
SOMD P0DML3 CSH1 CS 13103 125 PASS	2209	0.675	0.62	0.734	0	19.518	NA	NA	NA	NA	NA	NA
SOMA_Q8IXJ6_SIRT2_5030_52	2209	0.667	0.611	0.727	0	19.516	1077	0.273	0.160	0.466	5.738	<0.0001
SOMA_Q8TDN1_KCNG4_13525_17	2209	0.69	0.638	0.747	0	19.489	1077	0.556	0.418	0.738	4.302	<0.0001
SOMA_P07339_CTSD_5508_62	2209	1.485	1.365	1.616	0	19.436	1077	1.080	1.000	1.165	1.310	0.0489
SOMA_P78333_GPC5_4991_12	2209	0.675	0.62	0.734	0	19.412	1077	0.128	0.066	0.249	8.835	<0.0001
SOMA_Q13219_PAPPA_4148_49	2209	1.483	1.363	1.613	0	19.384	1077	1.357	1.266	1.454	17.312	<0.0001
SOMA_P50461_CSRP3_9171_11	2209	1.484	1.364	1.614	0	19.382	1077	1.197	1.053	1.361	2.235	0.0058
SOMA_P43251_BTD_15644_1	2209	0.677	0.623	0.736	0	19.376	1077	0.546	0.466	0.639	13.268	<0.0001
SOMA_P55058_PLTP_15475_4	2209	1.535	1.401	1.682	0	19.3	1077	1.520	1.405	1.646	24.447	<0.0001
SOMA_O00584_RNASET2_16913_8	2209	1.428	1.323	1.541	0	19.296	1077	1.112	1.049	1.179	3.458	0.0003
SOMA_P15814_IGLL1_6485_59	2209	0.666	0.61	0.726	0	19.246	1077	0.621	0.496	0.778	4.483	<0.0001
SOMA_P43363_MAGEA10_13610_9	2209	0.707	0.656	0.761	0	19.219	1077	0.593	0.462	0.762	4.381	<0.0001
SOMA_O15389_SIGLEC5_16792_4	2209	1.477	1.359	1.606	0	19.161	1077	1.715	1.539	1.910	21.869	<0.0001
SOMA_Q9HCN8_SDF2L1_6990_44	2209	1.472	1.355	1.6	0	19.158	1077	1.103	1.025	1.187	2.072	0.0085

SOMA_P78310_CXADR_11204_80	2209	0.684	0.63	0.742	0	19.136	1077	0.278	0.198	0.391	12.721	<0.0001
SOMA_Q9ULB5_CDH7_7959_34	2209	0.688	0.635	0.746	0	19.123	1077	0.403	0.300	0.540	8.946	<0.0001
SOMA_P19256_CD58_10938_13	2209	1.433	1.326	1.548	0	19.122	1077	1.170	1.088	1.259	4.630	<0.0001
SOMA_Q5JXA9_SIRPB2_5669_26	2209	1.462	1.347	1.587	0	19.041	1077	1.089	0.985	1.203	1.016	0.0964
SOMA_Q8IYJ3_SYTL1_12892_10	2209	0.707	0.657	0.762	0	19.039	1077	0.020	0.002	0.171	3.460	0.0003
SOMA_P29323_EPHB2_8225_86	2209	1.466	1.35	1.592	0	19.028	1077	1.355	1.241	1.479	10.934	<0.0001
SOMA_P55001_MFAP2_9294_45	2209	1.481	1.36	1.611	0	18.998	1077	1.061	1.000	1.125	1.306	0.0494
SOMA_P35318_ADM_7922_5	2209	1.445	1.334	1.564	0	18.98	1077	0.999	0.868	1.150	0.003	0.9925
SOMA_Q96BS2_TESC_12831_21	2209	1.478	1.358	1.608	0	18.881	1077	1.105	0.963	1.268	0.811	0.1544
SOMA_Q9NRR1_CYTL1_8402_22	2209	1.465	1.349	1.591	0	18.857	1077	1.236	1.128	1.355	5.251	<0.0001
SOMA_P08253_MMP2_4160_49	2209	1.498	1.372	1.635	0	18.828	1077	1.557	1.408	1.720	17.339	<0.0001
SOMA_Q9H3T3_SEMA6B_5121_3	2209	1.487	1.365	1.621	0	18.793	1077	1.405	1.318	1.499	24.388	<0.0001
SOMA_Q8WZ42_TTN_11352_42	2209	1.47	1.352	1.598	0	18.774	1077	1.218	1.138	1.302	8.013	<0.0001
SOMA_Q15399_TLR1_11149_3	2209	0.663	0.606	0.725	0	18.741	1077	0.022	0.003	0.188	3.309	0.0005
SOMA_P36222_CHI3L1_11104_13	2209	1.486	1.363	1.619	0	18.723	1077	1.210	1.093	1.340	3.617	0.0002
SOMA_Q96JJ7_TMX3_5654_70	2209	1.487	1.364	1.621	0	18.714	1077	1.370	1.239	1.515	9.079	<0.0001
SOMA_P35900_KRT20_12975_11	2209	1.485	1.363	1.618	0	18.713	1077	1.110	1.027	1.199	2.084	0.0082
SOMA_Q9UN76_SLC6A14_13053_6	2209	0.763	0.719	0.809	0	18.712	1077	0.192	0.126	0.293	13.748	<0.0001
SOMA_P09455_RBP1_19279_42	2209	1.472	1.353	1.601	0	18.665	1077	1.054	0.995	1.117	1.134	0.0735

SOMA_Q13426_XRCC4_9886_28	2209	1.444	1.333	1.564	0	18.645	1077	1.071	0.992	1.155	1.109	0.0779
SOMA_Q9NRA0_SPHK2_4468_21	2209	0.775	0.733	0.819	0	18.638	1077	0.684	0.492	0.950	1.627	0.0236
SOMA_O95861_BPNT1_17814_8	2209	1.444	1.333	1.564	0	18.618	1077	0.791	0.683	0.916	2.777	0.0017
SOMA_Q13506_NAB1_13933_276	2209	0.672	0.616	0.733	0	18.616	1077	0.720	0.545	0.950	1.693	0.0203
SOMA_P29120_PCSK1_13388_57	2209	1.484	1.361	1.617	0	18.586	1077	1.222	1.142	1.307	8.250	<0.0001
SOMA_O60462_NRP2_15387_44	2209	1.467	1.349	1.595	0	18.526	1077	1.798	1.617	1.998	26.830	<0.0001
SOMA_P03950_ANG_4874_3	2209	1.472	1.352	1.601	0	18.499	1077	1.204	1.055	1.374	2.237	0.0058
SOMA_Q14512_FGFBP1_15494_11	2209	1.478	1.357	1.61	0	18.47	1077	1.194	1.094	1.302	4.176	<0.0001
SOMA_P55854_SUMO3_14623_26	2209	1.455	1.34	1.58	0	18.44	1077	0.904	0.783	1.044	0.769	0.1702
SOMA_P38571_LIPA_7219_152	2209	1.463	1.346	1.59	0	18.438	NA	NA	NA	NA	NA	NA
SOMA_A6H8Y1_BDP1_8929_7	2209	0.699	0.646	0.756	0	18.428	1077	0.572	0.435	0.753	4.165	<0.0001
SOMA_P58062_SPINK7_10974_20	2209	1.485	1.362	1.62	0	18.373	1077	1.288	1.199	1.383	11.445	<0.0001
SOMA_P50452_SERPINB_19130_81	2209	1.471	1.351	1.601	0	18.294	1077	1.107	1.033	1.186	2.381	0.0042
SOMA_O14791_APOL1_9506_10	2209	0.68	0.624	0.74	0	18.28	1077	0.534	0.327	0.872	1.915	0.0122
SOMA_Q96BQ1_FAM3D_13102_1	2209	1.468	1.349	1.597	0	18.266	1077	1.242	1.121	1.376	4.458	<0.0001
SOMA_P23141_CES1_15487_164	2209	0.692	0.638	0.751	0	18.22	1077	0.574	0.457	0.721	5.733	<0.0001
SOMA_Q14126_DSG2_9484_75	2209	1.474	1.353	1.606	0	18.211	1077	1.458	1.341	1.585	17.982	<0.0001
SOMA_Q9Y3E1_HDGFRP3_18899_82	2209	1.456	1.34	1.582	0	18.168	NA	NA	NA	NA	NA	NA
SOMA_Q92832_NELL1_6544_33	2209	0.681	0.626	0.742	0	18.122	1077	0.935	0.751	1.163	0.264	0.5451

SOMA_Q9UEU0_VTI1B_8963_8	2209	1.428	1.32	1.545	0	18.102	1077	0.901	0.766	1.059	0.687	0.2054
SOMA_O60704_TPST2_8024_64	2209	0.698	0.645	0.756	0	18.089	1077	0.569	0.463	0.700	7.002	<0.0001
SOMA_Q9BQI7_PSD2_9118_7	2209	0.68	0.624	0.741	0	18.006	1077	0.575	0.360	0.920	1.677	0.021
SOMA_Q9HCE7_SMURF1_11557_3	2209	1.439	1.328	1.56	0	17.992	1077	1.036	0.945	1.137	0.346	0.4507
SOMA_Q96S55_WRNIP1_12528_40	2209	0.715	0.664	0.771	0	17.957	1077	0.235	0.080	0.691	2.069	0.0085
SOMA_Q99653_CHP1_12458_79	2209	1.461	1.343	1.59	0	17.94	1077	1.124	1.020	1.237	1.752	0.0177
SOMA_O75344_FKBP6_12529_32	2209	0.681	0.625	0.742	0	17.916	1077	0.394	0.252	0.617	4.322	<0.0001
SOMA_P49773_HINT1_5900_11	2209	1.472	1.351	1.604	0	17.911	1077	0.994	0.855	1.155	0.027	0.9389
SOMA_O95989_NUDT3_12796_44	2209	1.467	1.347	1.598	0	17.893	1077	1.132	1.015	1.263	1.576	0.0265
SOMA_Q9BW91_NUDT9_9482_110	2209	1.456	1.339	1.583	0	17.89	1077	1.114	0.987	1.258	1.098	0.0798
SOMA_Q9ULW6_NAP1L2_13529_39	2209	0.685	0.63	0.745	0	17.867	1077	0.599	0.333	1.077	1.060	0.0871
SOMA_P07333_CSF1R_13682_47	2209	1.482	1.358	1.618	0	17.855	1077	1.520	1.371	1.685	14.787	<0.0001
SOMA_Q8WWA0_ITLN1_18830_1	2209	1.479	1.356	1.614	0	17.853	1077	1.426	1.283	1.584	10.375	<0.0001
SOMA_Q9HBX9_RXFP1_14135_3	2209	0.702	0.649	0.76	0	17.849	1077	0.953	0.789	1.152	0.207	0.6205
SOMA_Q96GA7_SDSL_17777_31	2209	1.443	1.33	1.566	0	17.84	1077	1.038	0.931	1.157	0.300	0.5006
SOMA_P61077_UBE2D3_19280_29	2209	0.687	0.632	0.747	0	17.836	1077	0.072	0.017	0.305	3.442	0.0004
SOMA_P04180_LCAT_15413_3	2209	0.683	0.627	0.744	0	17.791	1077	1.155	1.067	1.249	3.466	0.0003
SOMA_Q96H15_TIMD4_15449_33	2209	1.479	1.355	1.614	0	17.791	1077	1.288	1.216	1.363	17.425	<0.0001
SOMA_P50897_PPT1_9244_27	2209	1.458	1.34	1.586	0	17.772	1077	1.191	1.086	1.306	3.682	0.0002

SOMA_O76064_RNF8_14663_44	2209	0.685	0.629	0.745	0	17.746	1077	0.903	0.699	1.166	0.364	0.433
SOMA_P16949_STMN1_17367_5	2209	1.447	1.332	1.571	0	17.745	1077	1.106	1.002	1.220	1.341	0.0456
SOMA_Q8NFU3_TSTD1_19277_4	2209	1.453	1.337	1.58	0	17.738	1077	0.987	0.853	1.141	0.068	0.8557
SOMA_Q86Y01_DTX1_11430_49	2209	0.69	0.635	0.75	0	17.732	1077	0.544	0.458	0.646	11.339	<0.0001
SOMA_Q16842_ST3GAL2_6281_51	2209	1.449	1.334	1.575	0	17.726	1077	1.063	0.944	1.198	0.506	0.3118
SOMA_P15056_BRAF_15669_7	2209	0.734	0.685	0.786	0	17.703	1077	0.915	0.753	1.113	0.425	0.3761
SOMA_Q14011_CIRBP_12724_81	2209	1.443	1.33	1.567	0	17.694	1077	1.055	0.912	1.221	0.328	0.47
SOMA_Q13976_PRKG1_13067_5	2209	0.695	0.641	0.754	0	17.691	1077	0.218	0.105	0.453	4.356	<0.0001
SOMA_Q9HCZ1_ZNF334_12763_69	2209	0.692	0.637	0.751	0	17.674	1077	0.298	0.063	1.407	0.898	0.1263
SOMA_Q92874_DNASE1L_6324_11	2209	1.438	1.326	1.561	0	17.63	1077	1.062	0.988	1.141	0.989	0.1025
SOMA_Q9Y4C0_NRXN3_16323_8	2209	1.449	1.333	1.574	0	17.622	1077	1.286	1.194	1.384	10.658	<0.0001
SOMA_P15918_RAG1_11311_79	2209	0.679	0.623	0.741	0	17.62	1077	0.147	0.053	0.410	3.618	0.0002
SOMA_O43927_CXCL13_3487_32	2209	1.458	1.34	1.587	0	17.556	1077	1.207	1.123	1.298	6.462	<0.0001
SOMA_P23528_CFL1_4203_50	2209	0.686	0.631	0.747	0	17.544	1077	0.642	0.530	0.776	5.303	<0.0001
SOMA_Q9UK23_NAGPA_11208_15	2209	0.697	0.643	0.756	0	17.512	1077	0.795	0.684	0.925	2.516	0.003
SOMA_O60939_SCN2B_8353_15	2209	0.7	0.646	0.759	0	17.504	1077	0.916	0.717	1.169	0.319	0.4796
SOMA_P22792_CPN2_6415_90	2209	0.678	0.621	0.74	0	17.502	1077	0.556	0.466	0.663	10.146	<0.0001
SOMA_Q9H2U2_PPA2_18307_71	2209	0.703	0.649	0.761	0	17.472	1077	1.044	0.915	1.192	0.284	0.5199
SOMA_P28799_GRN_4992_49	2209	1.453	1.335	1.58	0	17.463	1077	1.521	1.398	1.653	21.985	<0.0001

SOMA_Q10472_GALNT1_7090_17	2209	1.457	1.339	1.587	0	17.381	1077	1.097	1.041	1.157	3.223	0.0006
SOMA_Q9P0L0_VAPA_7167_102	2209	1.406	1.302	1.519	0	17.363	1077	0.824	0.703	0.967	1.745	0.018
SOMA_P49913_CAMP_15481_45	2209	0.681	0.625	0.743	0	17.357	1077	0.812	0.691	0.955	1.931	0.0117
SOMA_O75121_MFAP3L_8837_8	2209	0.684	0.628	0.746	0	17.356	1077	0.695	0.459	1.053	1.066	0.0859
SOMA_Q9BWP8_COLEC11_4430_44	2209	1.48	1.354	1.617	0	17.34	1077	1.279	1.177	1.389	8.212	<0.0001
SOMA_P57087_JAM2_2997_8	2209	1.44	1.326	1.564	0	17.314	1077	1.240	1.139	1.350	6.186	<0.0001
SOMA_Q9P244_LRFN1_7910_41	2209	0.702	0.648	0.76	0	17.299	1077	0.340	0.214	0.540	5.322	<0.0001
SOMA_P08590_MYL3_18376_19	2209	1.465	1.344	1.598	0	17.277	1077	1.235	1.152	1.324	8.496	<0.0001
SOMA_Q13277_STX3_7186_111	2209	1.432	1.32	1.554	0	17.261	1077	1.049	0.944	1.166	0.429	0.3725
SOMA_Q9UKZ9_PCOLCE2_6081_52	2209	0.684	0.628	0.746	0	17.228	1077	0.551	0.469	0.646	12.555	<0.0001
SOMA_Q13045_FLII_12677_164	2209	1.413	1.306	1.528	0	17.208	1077	0.679	0.552	0.835	3.610	0.0002
SOMA_Q8TCZ2_CD99L2_10539_30	2209	0.807	0.769	0.847	0	17.202	1077	0.613	0.373	1.008	1.271	0.0536
SOMA_O75534_CSDE1_12735_39	2209	0.696	0.641	0.756	0	17.195	1077	0.776	0.408	1.478	0.355	0.4412
SOMA_Q99645_EPYC_9278_9	2209	1.45	1.333	1.578	0	17.122	NA	NA	NA	NA	NA	NA
SOMA_Q86VW1_SLC22A1_9969_8	2209	0.694	0.638	0.754	0	17.121	1077	0.450	0.364	0.557	12.800	<0.0001
SOMA_O75509_TNFRSF2_5404_53	2209	1.468	1.345	1.603	0	17.102	1077	1.394	1.281	1.517	13.914	<0.0001
SOMA_O14960_LECT2_16763_11	2209	0.686	0.63	0.748	0	17.091	1077	0.491	0.417	0.577	17.215	<0.0001
SOMA_P52848_NDST1_6927_7	2209	0.699	0.645	0.759	0	17.086	1077	0.723	0.617	0.848	4.168	<0.0001
SOMA_Q08ET2_SIGLEC1_8248_222	2209	1.458	1.338	1.589	0	17.066	1077	1.546	1.405	1.701	18.359	<0.0001

SOMA_Q92692_PVRL2_6245_4	2209	1.46	1.339	1.592	0	17.05	NA	NA	NA	NA	NA	NA
SOMA_P02778_CXCL10_4141_79	2209	1.441	1.326	1.566	0	17.047	1077	1.199	1.128	1.275	8.184	<0.0001
SOMA_O15197_EPHB6_5078_82	2209	1.464	1.342	1.598	0	17.032	1077	1.184	1.116	1.256	7.609	<0.0001
SOMA_Q9UBR2_CTSZ_4971_1	2209	1.451	1.333	1.58	0	17.01	1077	1.345	1.206	1.499	7.012	<0.0001
SOMA_Q99439_CNN2_18877_15	2209	1.463	1.341	1.596	0	17.005	1077	1.003	0.869	1.157	0.015	0.9657
SOMA_P15907_ST6GAL1_6035_2	2209	1.427	1.315	1.547	0	16.98	1077	1.095	0.994	1.207	1.185	0.0653
SOMA_Q8TAT2_FGFBP3_11219_95	2209	1.455	1.335	1.586	0	16.88	1077	1.275	1.185	1.373	10.060	<0.0001
SOMA_Q01973_ROR1_2590_69	2209	1.451	1.332	1.581	0	16.876	1077	1.428	1.281	1.592	9.880	<0.0001
SOMA_Q9NVA2_SEPT11_12620_3	2209	1.447	1.329	1.575	0	16.857	NA	NA	NA	NA	NA	NA
SOMA_Q06481_APLP2_10627_87	2209	1.432	1.318	1.555	0	16.778	1077	1.117	0.981	1.272	1.028	0.0937
SOMA_Q6WN34_CHRDL2_6086_15	2209	1.442	1.325	1.569	0	16.762	1077	1.431	1.304	1.570	13.448	<0.0001
SOMA_P05546_SERPIND_3316_58	2209	0.683	0.626	0.746	0	16.756	1077	0.542	0.466	0.631	14.635	<0.0001
SOMA_Q8NBI6_XXYLT1_6375_75	2209	1.405	1.299	1.52	0	16.715	1077	1.107	1.050	1.167	3.800	0.0002
SOMA_P00995_SPINK1_8243_55	2209	1.429	1.316	1.552	0	16.687	1077	0.957	0.771	1.187	0.161	0.69
SOMA_Q15063_POSTN_3457_57	2209	1.449	1.33	1.579	0	16.658	1077	1.350	1.212	1.503	7.346	<0.0001
SOMA_O60258_FGF17_3494_71	2209	0.823	0.786	0.861	0	16.657	1077	0.947	0.774	1.158	0.227	0.5936
SOMA_P78504_JAG1_5092_51	2209	1.414	1.305	1.531	0	16.644	1077	1.330	1.230	1.438	12.127	<0.0001
SOMA_Q9NPY3_CD93_14136_234	2209	1.454	1.334	1.586	0	16.644	1077	1.480	1.333	1.643	12.757	<0.0001
SOMA_Q9NPA1_KCNMB3_8905_20	2209	0.697	0.642	0.758	0	16.56	1077	0.612	0.494	0.758	5.152	<0.0001

SOMA_P26441_CNTF_3489_9	2209	0.812	0.773	0.852	0	16.558	1077	0.715	0.452	1.134	0.813	0.1538
SOMA_Q9Y281_CFL2_15339_32	2209	1.442	1.325	1.57	0	16.553	1077	1.355	1.245	1.475	11.680	<0.0001
SOMA_Q9HDB5_NRXN3_5111_15	2209	1.45	1.33	1.58	0	16.506	1077	1.292	1.195	1.396	9.992	<0.0001
SOMA_P08174_CD55_5069_9	2209	1.459	1.337	1.593	0	16.504	1077	1.338	1.235	1.449	12.122	<0.0001
SOMA_P49767_VEGFC_3132_1	2209	1.416	1.306	1.536	0	16.461	1077	1.057	0.931	1.200	0.404	0.3944
SOMA_O00182_LGALS9_9197_4	2209	1.438	1.322	1.565	0	16.46	1077	1.063	0.977	1.156	0.813	0.1537
SOMA_P08319_ADH4_8325_37	2209	0.681	0.622	0.745	0	16.396	1077	0.862	0.713	1.043	0.897	0.1269
SOMA_Q6NSJ0_KIAA116_8068_43	2209	0.718	0.665	0.776	0	16.388	NA	NA	NA	NA	NA	NA
SOMA_Q6Q788_APOA5_15363_32	2209	0.688	0.631	0.751	0	16.384	1077	0.452	0.351	0.582	9.120	<0.0001
SOMA_Q6UWS5_PET117_19303_64	2209	1.356	1.263	1.456	0	16.367	1077	1.361	1.237	1.497	9.635	<0.0001
SOMA_Q96J94_PIWIL1_12793_4	2209	0.682	0.623	0.745	0	16.346	1077	1.060	0.992	1.131	1.077	0.0837
SOMA_P02008_HBZ_6919_3	2209	0.691	0.634	0.753	0	16.327	1077	0.557	0.414	0.751	3.908	0.0001
SOMA_Q9BY76_ANGPTL4_3796_79	2209	1.435	1.319	1.562	0	16.298	1077	1.273	1.185	1.367	10.423	<0.0001
SOMA_A8MVW0_FAM171A_13479_8	2209	0.7	0.644	0.761	0	16.273	1077	0.796	0.616	1.028	1.092	0.081
SOMA_P06127_CD5_5596_75	2209	1.443	1.325	1.573	1.65E-13	16.24	1077	1.586	1.449	1.735	23.059	<0.0001
SOMA_O15072_ADAMTS3_8845_2	2209	0.69	0.633	0.753	1.65E-13	16.199	1077	0.359	0.220	0.585	4.400	<0.0001
SOMA_Q14393_GAS6_15391_114	2209	1.448	1.327	1.579	1.65E-13	16.196	1077	1.571	1.441	1.714	23.752	<0.0001
SOMA_P01270_PTH_5954_62	2209	1.418	1.306	1.539	1.65E-13	16.143	1077	1.072	1.012	1.136	1.744	0.018
SOMA_Q9HBE5_IL21R_9366_54	2209	1.418	1.306	1.539	1.65E-13	16.141	1077	1.138	1.070	1.209	4.446	<0.0001

SOMA_Q9H2B2_SYT4_17355_56	2209	0.69	0.633	0.753	1.65E-13	16.02	1077	0.549	0.417	0.722	4.741	<0.0001
SOMA_P40189_IL6ST_2620_4	2209	1.458	1.334	1.594	1.65E-13	16.015	1077	1.769	1.590	1.967	25.014	<0.0001
SOMA_P79483_HLA_DRB_6962_5	2209	0.698	0.642	0.76	1.65E-13	15.983	1077	0.686	0.554	0.849	3.271	0.0005
SOMA_P09326_CD48_3292_75	2209	1.425	1.311	1.549	1.65E-13	15.96	1077	1.337	1.228	1.455	10.737	<0.0001
SOMA_P25445_FAS_5392_73	2209	1.387	1.283	1.498	1.65E-13	15.924	1077	1.320	1.207	1.444	8.866	<0.0001
SOMA_Q9UBP6_METTL1_12514_16	2209	0.691	0.633	0.754	1.65E-13	15.917	1077	0.731	0.537	0.996	1.328	0.047
SOMA_Q6UXM1_LRIG3_3322_52	2209	0.703	0.647	0.764	1.65E-13	15.871	1077	0.828	0.714	0.960	1.913	0.0122
SOMA_P12268_IMPDH2_5250_53	2209	0.709	0.653	0.769	1.65E-13	15.851	1077	0.791	0.646	0.969	1.630	0.0234
SOMA_P07306_ASGR1_5452_71	2209	1.45	1.328	1.584	1.65E-13	15.838	1077	1.541	1.426	1.665	27.063	<0.0001
SOMA_P25686_DNAJB2_11438_6	2209	1.414	1.302	1.535	1.65E-13	15.812	1077	0.857	0.732	1.002	1.275	0.0531
SOMA_Q9UJY5_GGA1_13594_158	2209	1.409	1.299	1.529	1.65E-13	15.784	1077	0.849	0.736	0.978	1.623	0.0238
SOMA_Q9GZN4_PRSS22_4534_10	2209	1.422	1.308	1.546	1.65E-13	15.783	1077	1.471	1.340	1.615	15.261	<0.0001
SOMA_P00797_REN_3396_54	2209	1.434	1.316	1.562	3.31E-13	15.778	1077	1.408	1.252	1.583	7.917	<0.0001
SOMA_Q6UXI7_VIT_6234_74	2209	1.394	1.288	1.509	3.31E-13	15.656	1077	1.117	1.031	1.210	2.177	0.0067
SOMA_Q8N3Z0_PRSS35_9983_97	2209	0.724	0.67	0.782	3.31E-13	15.64	1077	0.011	0.002	0.059	6.909	<0.0001
SOMA_O95336_PGLS_17799_9	2209	1.408	1.297	1.527	3.31E-13	15.628	1077	0.891	0.756	1.049	0.781	0.1655
SOMA_Q8TDF5_NETO1_15298_199	2209	1.41	1.299	1.531	3.31E-13	15.625	1077	1.063	0.977	1.157	0.804	0.1571
SOMA_O75054_IGSF3_9715_15	2209	1.413	1.301	1.535	3.31E-13	15.611	1077	1.203	1.110	1.303	5.230	<0.0001
SOMA_P09958_FURIN_6276_16	2209	1.424	1.309	1.55	3.31E-13	15.61	1077	1.037	0.958	1.122	0.435	0.3669

SOMA_Q86SI9_C5orf38_6378_2	2209	0.709	0.653	0.77	3.31E-13	15.606	1077	0.526	0.436	0.636	10.609	<0.0001
SOMA_P14314_PRKCSH_5687_5	2209	1.414	1.302	1.537	3.31E-13	15.591	1077	1.250	1.160	1.348	8.213	<0.0001
SOMA_O43464_HTRA2_3317_33	2209	1.414	1.301	1.536	3.31E-13	15.558	1077	1.122	0.992	1.268	1.181	0.0659
SOMA_P51911_CNN1_15566_10	2209	1.404	1.294	1.523	4.96E-13	15.537	1077	1.006	0.873	1.160	0.029	0.9348
SOMA_Q96A84_EMID1_13021_12	2209	0.743	0.691	0.798	4.96E-13	15.531	NA	NA	NA	NA	NA	NA
SOMA_Q8N912_NRAC_13464_8	2209	0.691	0.633	0.755	4.96E-13	15.522	1077	0.943	0.747	1.191	0.206	0.6221
SOMA_P16035_TIMP2_2278_61	2209	1.436	1.317	1.567	4.96E-13	15.516	1077	1.548	1.397	1.716	16.054	<0.0001
SOMA_P17936_IGFBP3_2571_12	2209	0.695	0.637	0.759	4.96E-13	15.493	1077	0.754	0.649	0.875	3.676	0.0002
SOMA_P22004_BMP6_8459_10	2209	1.439	1.319	1.571	4.96E-13	15.492	1077	1.661	1.503	1.835	22.715	<0.0001
SOMA_Q765I0_UTS2B_6290_3	2209	1.417	1.303	1.541	4.96E-13	15.478	1077	1.027	0.906	1.163	0.168	0.6791
SOMA_O43557_TNFSF14_5988_49	2209	0.702	0.645	0.764	4.96E-13	15.47	1077	0.302	0.192	0.476	6.591	<0.0001
SOMA_Q16666_IFI16_13940_19	2209	1.4	1.291	1.517	4.96E-13	15.433	1077	1.099	0.988	1.222	1.087	0.0818
SOMA_Q9Y5G2_PCDHGB2_10803_22	2209	1.404	1.294	1.523	6.61E-13	15.386	NA	NA	NA	NA	NA	NA
SOMA_P10147_CCL3_3040_59	2209	1.425	1.308	1.552	6.61E-13	15.374	1077	1.089	1.032	1.149	2.723	0.0019
SOMA_Q8IW52_SLITRK4_7139_14	2209	1.432	1.313	1.562	6.61E-13	15.368	1077	1.386	1.271	1.512	12.727	<0.0001
SOMA_Q9UI46_DNAI1_11396_39	2209	0.717	0.661	0.777	6.61E-13	15.364	1077	0.684	0.425	1.099	0.934	0.1165
SOMA_P09496_CLTA_19257_11	2209	1.481	1.347	1.628	6.61E-13	15.357	1077	1.156	1.029	1.299	1.839	0.0145
SOMA_P16234_PDGFRA_10366_11	2209	1.408	1.297	1.53	6.61E-13	15.322	1077	1.058	0.963	1.161	0.622	0.2388
SOMA_P25445_FAS_9459_7	2209	1.403	1.293	1.522	6.61E-13	15.319	1077	1.195	1.107	1.289	5.319	<0.0001

SOMA_P07195_LDHB_3890_8	2209	1.429	1.311	1.558	6.61E-13	15.304	1077	1.189	1.068	1.324	2.815	0.0015
SOMA_Q8WXH0_SYNE2_9789_52	2209	1.435	1.315	1.566	6.61E-13	15.304	1077	1.034	0.899	1.191	0.195	0.6387
SOMA_P39059_COL15A1_8974_172	2209	1.418	1.303	1.543	8.27E-13	15.271	1077	1.248	1.121	1.389	4.304	<0.0001
SOMA_P40238_MPL_3473_78	2209	0.816	0.777	0.857	8.27E-13	15.268	1077	0.448	0.356	0.565	10.920	<0.0001
SOMA_Q13137_CALCOCO_12534_10	2209	1.426	1.308	1.553	9.92E-13	15.213	1077	1.230	1.143	1.325	7.399	<0.0001
SOMA_O14594_NCAN_15573_110	2209	0.707	0.65	0.769	9.92E-13	15.184	1077	0.815	0.696	0.955	1.946	0.0113
SOMA_P04843_RPN1_6458_6	2209	1.347	1.253	1.448	1.16E-12	15.123	1077	1.455	1.334	1.588	16.481	<0.0001
SOMA_P11142_HSPA8_5903_91	2209	1.399	1.289	1.518	1.32E-12	15.074	1077	1.107	1.037	1.182	2.613	0.0024
SOMA_P01138_NGF_5801_72	2209	1.4	1.29	1.519	1.32E-12	15.052	1077	1.005	0.881	1.147	0.029	0.9354
SOMA_Q6ZMJ2_SCARA5_10419_1	2209	1.422	1.305	1.55	1.49E-12	15.011	1077	1.621	1.478	1.778	23.935	<0.0001
SOMA_P31947_SFN_4829_43	2209	1.42	1.304	1.547	1.49E-12	14.994	1077	1.163	1.109	1.220	9.242	<0.0001
SOMA_Q07325_CXCL9_9188_119	2209	1.4	1.29	1.52	1.49E-12	14.986	1077	1.054	0.963	1.155	0.593	0.2551
SOMA_Q16512_PKN1_12562_1	2209	0.705	0.647	0.768	1.65E-12	14.96	1077	0.734	0.595	0.906	2.412	0.0039
SOMA_Q9UMF0_ICAM5_5124_69	2209	1.429	1.309	1.56	1.98E-12	14.882	1077	1.571	1.398	1.765	13.505	<0.0001
SOMA_O15130_NPFF_5617_41	2209	0.707	0.65	0.77	2.31E-12	14.795	1077	0.075	0.034	0.166	9.922	<0.0001
SOMA_P15121_AKR1B1_16606_85	2209	1.385	1.278	1.5	2.48E-12	14.791	1077	1.002	0.868	1.156	0.008	0.9807
SOMA_Q9UEF7_KL_15384_15	2209	0.713	0.656	0.775	2.81E-12	14.733	1077	0.861	0.716	1.035	0.958	0.1103
SOMA_Q9UEW3_MARCO_9003_99	2209	0.769	0.721	0.821	2.81E-12	14.72	1077	0.467	0.352	0.619	6.919	<0.0001
SOMA_P22897_MRC1_2637_77	2209	1.423	1.305	1.553	2.98E-12	14.701	1077	1.359	1.284	1.439	25.529	<0.0001

SOMA_P17174_GOT1_4912_17	2209	0.701	0.642	0.765	2.98E-12	14.7	1077	0.584	0.475	0.718	6.483	<0.0001
SOMA_A6NI73_LILRA5_8766_29	2209	1.402	1.29	1.524	3.14E-12	14.681	1077	1.424	1.295	1.565	12.633	<0.0001
SOMA_Q9UJA9_ENPP5_6556_5	2209	0.718	0.661	0.779	3.31E-12	14.662	1077	0.788	0.673	0.922	2.519	0.003
SOMA_Q13336_SLC14A1_13430_50	2209	0.771	0.724	0.823	3.31E-12	14.658	1077	0.208	0.119	0.365	7.356	<0.0001
SOMA_Q03692_COL10A1_15653_9	2209	1.395	1.285	1.514	3.31E-12	14.649	1077	0.986	0.838	1.161	0.061	0.8692
SOMA_Q8IYN2_TCEAL8_19109_32	2209	0.719	0.663	0.78	3.47E-12	14.632	1077	0.979	0.832	1.151	0.098	0.7972
SOMA_Q9BXR6_CFHR5_16055_3	2209	0.709	0.651	0.772	3.47E-12	14.625	1077	0.844	0.727	0.980	1.577	0.0265
SOMA_P21781_FGF7_4487_1	2209	1.404	1.291	1.528	3.64E-12	14.621	1077	0.957	0.825	1.110	0.252	0.5594
SOMA_P00736_C1R_3285_23	2209	0.715	0.658	0.777	3.64E-12	14.618	1077	0.914	0.786	1.063	0.612	0.2441
SOMA_Q8WUE5_CT55_9363_11	2209	0.816	0.776	0.858	4.13E-12	14.555	1077	1.041	0.943	1.149	0.373	0.4241
SOMA_Q12913_PTPRJ_8250_2	2209	0.724	0.668	0.785	4.46E-12	14.522	1077	0.936	0.784	1.117	0.334	0.4638
SOMA_P08237_PFKM_17384_110	2209	0.704	0.645	0.768	4.96E-12	14.484	1077	0.412	0.297	0.573	6.859	<0.0001
SOMA_P55773_CCL23_3028_36	2209	1.426	1.305	1.558	5.46E-12	14.43	1077	1.505	1.384	1.637	20.784	<0.0001
SOMA_P62318_SNRPD3_17774_38	2209	0.71	0.652	0.773	5.62E-12	14.419	1077	0.508	0.309	0.835	2.119	0.0076
SOMA_O43508_TNFSF12_5939_42	2209	0.706	0.647	0.77	5.95E-12	14.396	1077	0.837	0.627	1.118	0.642	0.2281
SOMA_P10145_CXCL8_3447_64	2209	1.415	1.298	1.543	5.95E-12	14.394	1077	1.157	1.101	1.215	8.243	<0.0001
SOMA_P55040_GEM_12817_1	2209	0.709	0.651	0.773	6.12E-12	14.387	1077	0.294	0.188	0.458	7.167	<0.0001
SOMA_P18031_PTPN1_3005_5	2209	0.716	0.659	0.779	6.12E-12	14.383	1077	0.733	0.590	0.910	2.309	0.0049
SOMA_P81277_PRLH_6543_182	2209	0.714	0.656	0.777	6.28E-12	14.372	1077	1.043	0.932	1.167	0.335	0.4628

SOMA_Q96JX3_SERAC1_8985_13	2209	0.714	0.656	0.777	6.45E-12	14.361	1077	0.656	0.433	0.994	1.330	0.0468
SOMA_P58417_NXPH1_4562_1	2209	0.71	0.652	0.774	6.61E-12	14.353	1077	0.631	0.535	0.745	7.329	<0.0001
SOMA_Q9NZK5_CECR1_6077_63	2209	1.411	1.295	1.538	6.78E-12	14.342	NA	NA	NA	NA	NA	NA
SOMA_Q9UIS9_MBD1_14294_61	2209	0.713	0.655	0.776	7.27E-12	14.306	1077	0.554	0.459	0.668	9.236	<0.0001
SOMA_P08254_MMP3_2788_55	2209	0.714	0.656	0.777	7.60E-12	14.295	1077	0.352	0.158	0.783	1.980	0.0105
SOMA_P61371_ISL1_11549_6	2209	0.75	0.698	0.806	7.60E-12	14.294	1077	1.083	0.990	1.186	1.089	0.0815
SOMA_Q9NRM6_IL17RB_6262_14	2209	1.378	1.271	1.493	7.77E-12	14.279	1077	0.802	0.683	0.943	2.119	0.0076
SOMA_P55774_CCL18_3044_3	2209	1.406	1.291	1.532	7.93E-12	14.276	1077	1.558	1.376	1.764	11.619	<0.0001
SOMA_Q6PL18_ATAD2_13043_157	2209	0.821	0.782	0.863	8.10E-12	14.262	1077	1.007	0.874	1.161	0.036	0.9201
SOMA_Q2TAL6_VWC2_15308_108	2209	1.393	1.282	1.513	8.76E-12	14.231	1077	1.341	1.198	1.502	6.425	<0.0001
SOMA_Q6UWV7_FAM159A_13431_74	2209	1.389	1.279	1.508	9.09E-12	14.211	NA	NA	NA	NA	NA	NA
SOMA_O14994_SYN3_7199_3	2209	0.808	0.766	0.852	9.75E-12	14.187	1077	0.740	0.493	1.111	0.833	0.1468
SOMA_Q9Y5Y6_ST14_10927_65	2209	0.8	0.756	0.846	9.75E-12	14.186	NA	NA	NA	NA	NA	NA
SOMA_Q12794_HYAL1_8309_12	2209	0.721	0.664	0.782	9.75E-12	14.182	1077	0.737	0.625	0.868	3.599	0.0003
SOMA_Q96EE4_CCDC126_6388_21	2209	0.711	0.652	0.775	9.75E-12	14.181	1077	0.284	0.140	0.577	3.312	0.0005
SOMA_P08670_VIM_15540_6	2209	1.376	1.269	1.491	1.21E-11	14.092	1077	1.122	1.023	1.229	1.851	0.0141
SOMA_O75973_C1QL1_6404_20	2209	1.419	1.299	1.55	1.22E-11	14.085	1077	1.183	1.114	1.257	7.330	<0.0001
SOMA_P21860_ERBB3_2617_56	2209	0.72	0.662	0.782	1.32E-11	14.054	1077	0.786	0.656	0.943	2.028	0.0094
SOMA_O43692_PI15_5745_64	2209	0.714	0.655	0.777	1.34E-11	14.047	1077	0.647	0.500	0.837	3.045	0.0009

SOMA_Q8WWV3_RTN4IP1_18180_58	2209	1.382	1.273	1.499	1.37E-11	14.038	1077	0.949	0.818	1.102	0.307	0.4934
SOMD P02671 FGA FGB 4907 56 PASS	2209	1.374	1.268	1.489	1.39E-11	14.032	NA	NA	NA	NA	NA	NA
SOMD P02675 FGA FGB 4907 56 PASS	2209	1.374	1.268	1.489	1.39E-11	14.032	NA	NA	NA	NA	NA	NA
SOMD P02679 FGA FGB 4907 56 PASS	2209	1.374	1.268	1.489	1.39E-11	14.032	NA	NA	NA	NA	NA	NA
SOMA_P14207_FOLR2_15587_20	2209	1.414	1.296	1.544	1.39E-11	14.03	1077	1.240	1.171	1.313	12.810	<0.0001
SOMA_P31321_PRKAR1B_12479_50	2209	1.374	1.268	1.489	1.39E-11	14.03	1077	1.051	0.933	1.184	0.384	0.413
SOMA_Q8WXC3_PYDC1_12835_101	2209	0.718	0.66	0.78	1.39E-11	14.03	1077	0.750	0.468	1.202	0.634	0.2321
SOMA_P13942_COL11A2_11278_4	2209	0.705	0.646	0.771	1.55E-11	13.982	1077	0.546	0.445	0.670	8.160	<0.0001
SOMA_Q86VB7_CD163_5028_59	2209	1.405	1.289	1.532	1.55E-11	13.979	1077	1.690	1.519	1.881	21.269	<0.0001
SOMA_Q13753_LAMC2_9580_5	2209	1.368	1.263	1.48	1.59E-11	13.973	1077	1.145	1.092	1.201	7.609	<0.0001
SOMA_O15173_PGRMC2_8681_93	2209	0.719	0.662	0.782	1.62E-11	13.964	1077	0.457	0.315	0.664	4.402	<0.0001
SOMA_P62308_SNRPG_19276_124	2209	1.373	1.267	1.487	1.65E-11	13.956	1077	0.604	0.412	0.886	2.005	0.0099
SOMA_P02679_FGG_4989_7	2209	1.362	1.259	1.473	1.75E-11	13.928	1077	1.474	1.341	1.620	15.064	<0.0001
SOMA_P16519_PCSK2_6117_4	2209	1.389	1.277	1.51	2.05E-11	13.863	1077	1.087	1.011	1.168	1.610	0.0245
SOMA_O75556_SCGB2A1_5001_6	2209	0.713	0.654	0.777	2.07E-11	13.857	1077	0.466	0.343	0.633	5.999	<0.0001
SOMA_Q9BUR5_APOO_9373_405	2209	0.821	0.78	0.863	2.33E-11	13.805	NA	NA	NA	NA	NA	NA
SOMA_O15247_CLIC2_17837_5	2209	1.389	1.277	1.51	2.60E-11	13.759	1077	1.209	1.110	1.316	4.891	<0.0001
SOMA_P07148_FABP1_11516_7	2209	1.4	1.284	1.525	2.61E-11	13.757	1077	1.299	1.209	1.396	11.975	<0.0001
SOMA_P45973_CBX5_4540_11	2209	0.72	0.662	0.783	2.89E-11	13.71	1077	0.916	0.735	1.143	0.359	0.4375

SOMA_P41970_ELK3_5707_55	2209	1.372	1.265	1.487	2.98E-11	13.698	1077	0.823	0.705	0.961	1.867	0.0136
SOMA_Q12904_AIMP1_2714_78	2209	0.741	0.686	0.8	2.99E-11	13.698	1077	0.126	0.066	0.241	9.365	<0.0001
SOMA_P35754_GLRX_18386_36	2209	1.345	1.247	1.451	3.06E-11	13.687	1077	0.986	0.836	1.162	0.063	0.8659
SOMA_Q13158_FADD_16593_3	2209	0.752	0.699	0.809	3.14E-11	13.675	1077	0.346	0.267	0.450	14.686	<0.0001
SOMA_Q8N5Y8_PARP16_7881_244	2209	0.722	0.664	0.785	3.36E-11	13.648	1077	1.069	0.954	1.198	0.603	0.2494
SOMA_O15460_P4HA2_11348_132	2209	0.714	0.655	0.779	3.41E-11	13.64	1077	0.509	0.082	3.150	0.330	0.4679
SOMA_Q9UBM4_OPTC_15430_165	2209	0.74	0.685	0.8	3.49E-11	13.629	1077	0.239	0.139	0.409	6.706	<0.0001
SOMA_Q10469_MGAT2_6909_40	2209	0.714	0.655	0.779	3.55E-11	13.623	1077	0.629	0.505	0.784	4.445	<0.0001
SOMA_Q9BUN1_MENT_5744_12	2209	0.721	0.663	0.784	3.70E-11	13.605	1077	0.593	0.469	0.750	4.887	<0.0001
SOMA_Q96J42_TXNDC15_6366_38	2209	1.395	1.28	1.519	3.84E-11	13.59	1077	1.318	1.226	1.416	13.260	<0.0001
SOMA_P62979_RPS27A_2846_24	2209	1.381	1.271	1.501	3.90E-11	13.583	1077	1.042	0.910	1.194	0.261	0.5487
SOMA_P03971_AMH_4923_79	2209	0.729	0.672	0.791	3.92E-11	13.581	1077	0.673	0.577	0.785	6.308	<0.0001
SOMA_Q96DU3_SLAMF6_5128_53	2209	1.397	1.282	1.523	4.08E-11	13.562	1077	1.024	0.919	1.140	0.173	0.6715
SOMA_Q99729_HNRNPAB_8894_80	2209	1.366	1.26	1.48	4.31E-11	13.538	1077	1.029	0.908	1.165	0.184	0.655
SOMA_P07451_CA3_3799_11	2209	1.39	1.277	1.513	4.40E-11	13.53	1077	1.233	1.110	1.369	4.027	<0.0001
SOMA_Q9NP55_BPIFA1_6473_55	2209	0.715	0.655	0.779	4.74E-11	13.497	1077	0.895	0.669	1.196	0.345	0.4521
SOMA_P56279_TCL1A_17210_2	2209	0.71	0.649	0.775	4.91E-11	13.482	1077	0.178	0.090	0.348	6.302	<0.0001
SOMA_O95841_ANGPTL1_11142_11	2209	1.37	1.263	1.486	5.04E-11	13.471	1077	1.221	1.134	1.314	6.935	<0.0001
SOMA_Q9H361_PABPC3_10447_18	2209	1.377	1.268	1.496	5.12E-11	13.464	1077	1.117	0.970	1.286	0.900	0.1258

SOMA_O14641_DVL2_13575_40	2209	0.718	0.659	0.782	5.69E-11	13.418	1077	0.750	0.649	0.867	4.014	<0.0001
SOMA_Q9NR61_DLL4_3305_6	2209	1.378	1.268	1.497	5.72E-11	13.416	1077	0.985	0.824	1.177	0.061	0.8684
SOMA_P18428_LBP_3074_6	2209	1.385	1.273	1.506	5.75E-11	13.413	1077	1.636	1.477	1.812	20.431	<0.0001
SOMA_Q9Y5Q6_INSL5_10462_14	2209	1.387	1.274	1.509	6.02E-11	13.393	1077	1.071	0.999	1.148	1.263	0.0546
SOMA_P14210_HGF_2681_23	2209	1.364	1.258	1.478	6.53E-11	13.358	1077	1.204	1.100	1.319	4.208	<0.0001
SOMD P0C0L4 C4A C4B 2182 54 PASS	2209	0.717	0.658	0.782	6.84E-11	13.337	NA	NA	NA	NA	NA	NA
SOMD P0C0L5 C4A C4B 2182 54 PASS	2209	0.717	0.658	0.782	6.84E-11	13.337	NA	NA	NA	NA	NA	NA
SOMA_Q9BSI4_TINF2_10638_1	2209	0.79	0.743	0.84	7.49E-11	13.299	1077	0.450	0.114	1.767	0.598	0.2522
SOMA_Q9HAV7_GRPEL1_7113_1	2209	1.379	1.268	1.499	7.55E-11	13.295	1077	1.231	1.108	1.369	3.939	0.0001
SOMA_P02774_GC_15589_1	2209	0.73	0.673	0.793	7.87E-11	13.277	1077	0.869	0.744	1.014	1.126	0.0748
SOMA_Q9H773_DCTPP1_4314_12	2209	1.384	1.272	1.507	8.07E-11	13.266	1077	1.653	1.506	1.814	25.356	<0.0001
SOMA_P52758_HRSP12_14636_25	2209	1.368	1.26	1.485	9.34E-11	13.202	NA	NA	NA	NA	NA	NA
SOMA_P04843_RPN1_10490_3	2209	1.354	1.251	1.465	9.41E-11	13.2	1077	1.038	0.947	1.138	0.375	0.4215
SOMA_Q63HQ2_EGFLAM_12338_27	2209	1.389	1.275	1.514	9.90E-11	13.177	1077	1.162	1.097	1.230	6.533	<0.0001
SOMA_O75398_DEAF1_6369_82	2209	0.726	0.667	0.789	1.03E-10	13.159	1077	0.576	0.405	0.819	2.671	0.0021
SOMA_Q9NSY2_STARD5_17383_4	2209	1.37	1.262	1.488	1.03E-10	13.159	1077	1.016	0.882	1.170	0.082	0.8287
SOMA_O95825_CRYZL1_9207_60	2209	0.725	0.667	0.789	1.05E-10	13.152	1077	0.482	0.355	0.654	5.576	<0.0001
SOMA_P42345_FRAP1_16780_6	2209	1.347	1.246	1.457	1.24E-10	13.08	NA	NA	NA	NA	NA	NA
SOMA_Q6NX45_ZNF774_12760_34	2209	0.71	0.649	0.777	1.32E-10	13.052	1077	0.748	0.494	1.132	0.772	0.1691

SOMD P01374 LTA LTB 3505 6 PASS	2209	0.731	0.673	0.794	1.32E-10	13.052	NA	NA	NA	NA	NA	NA
SOMD Q06643 LTA LTB 3505 6 PASS	2209	0.731	0.673	0.794	1.32E-10	13.052	NA	NA	NA	NA	NA	NA
SOMA_Q8TEY5_CREB3L4_11308_8	2209	1.383	1.27	1.506	1.33E-10	13.049	1077	1.172	1.087	1.264	4.427	<0.0001
SOMA_Q13510_ASAH1_5748_20	2209	1.355	1.251	1.468	1.36E-10	13.04	1077	0.794	0.670	0.941	2.117	0.0076
SOMA_Q9P2E7_PCDH10_9018_38	2209	1.38	1.268	1.503	1.38E-10	13.033	1077	1.110	1.025	1.201	1.997	0.0101
SOMA_P07911_UMOD_9451_20	2209	1.371	1.261	1.489	1.41E-10	13.024	1077	1.556	1.409	1.718	17.537	<0.0001
SOMA_P17813_ENG_4908_6	2209	1.388	1.273	1.513	1.46E-10	13.009	1077	1.714	1.552	1.892	25.856	<0.0001
SOMA_P29622_SERPINA_3449_58	2209	0.718	0.658	0.783	1.54E-10	12.986	1077	0.607	0.530	0.695	12.218	<0.0001
SOMA_P40197_GP5_7185_29	2209	0.72	0.66	0.785	1.61E-10	12.966	1077	0.684	0.587	0.797	5.949	<0.0001
SOMA_Q9ULS5_TMCC3_8922_4	2209	0.723	0.663	0.787	1.70E-10	12.943	1077	0.499	0.386	0.646	6.911	<0.0001
SOMA_Q14624_ITIH4_4811_33	2209	1.379	1.266	1.501	1.85E-10	12.906	1077	1.875	1.682	2.090	29.110	<0.0001
SOMA_O00391_QSOX1_6070_11	2209	0.748	0.692	0.808	1.91E-10	12.891	NA	NA	NA	NA	NA	NA
SOMA_Q96JJ6_JPH4_9111_40	2209	0.732	0.673	0.795	1.96E-10	12.88	1077	0.920	0.699	1.212	0.257	0.5536
SOMA_Q9HD89_RETN_3046_31	2209	1.376	1.264	1.497	1.96E-10	12.88	1077	1.417	1.279	1.570	10.596	<0.0001
SOMA_Q9UBP0_SPAST_8388_24	2209	0.71	0.648	0.777	2.06E-10	12.86	1077	0.963	0.774	1.197	0.136	0.7312
SOMA_P53674_CRYBB1_17751_68	2209	1.354	1.25	1.468	2.07E-10	12.857	1077	0.995	0.857	1.155	0.023	0.9477
SOMA_P30086_PEBP1_4276_10	2209	1.367	1.259	1.486	2.19E-10	12.832	1077	0.962	0.820	1.128	0.199	0.6324
SOMA_O95715_CXCL14_5730_60	2209	1.372	1.261	1.493	2.69E-10	12.743	1077	1.084	0.959	1.227	0.705	0.1974
SOMA_P33316_DUT_9995_6	2209	1.355	1.249	1.469	2.70E-10	12.742	1077	1.068	0.993	1.149	1.124	0.0751

SOMA_Q5JXM2_METTL24_9470_15	2209	1.372	1.261	1.492	2.79E-10	12.727	1077	1.078	0.973	1.195	0.826	0.1491
SOMA_Q9ULZ1_APLN_6622_90	2209	0.722	0.662	0.787	2.95E-10	12.703	1077	0.183	0.109	0.308	9.791	<0.0001
SOMA_P02654_APOC1_15364_101	2209	0.737	0.679	0.799	3.04E-10	12.69	1077	0.770	0.665	0.892	3.290	0.0005
SOMA_P35030_PRSS3_18864_7	2209	1.39	1.273	1.518	3.13E-10	12.677	1077	1.224	1.136	1.319	6.925	<0.0001
SOMA_Q9BXJ5_C1QTNF2_8820_2	2209	0.725	0.665	0.79	3.31E-10	12.653	NA	NA	NA	NA	NA	NA
SOMA_P05230_FGF1_3486_58	2209	0.815	0.771	0.861	3.38E-10	12.644	1077	0.961	0.778	1.187	0.148	0.7111
SOMA_P31751_AKT2_14685_17	2209	0.725	0.665	0.79	3.39E-10	12.642	1077	0.629	0.492	0.802	3.716	0.0002
SOMA_P08118_MSMB_10620_21	2209	1.377	1.264	1.5	3.87E-10	12.585	1077	1.282	1.154	1.424	5.454	<0.0001
SOMA_Q96A72_MAGOHB_16875_13	2209	1.341	1.239	1.451	4.11E-10	12.559	1077	0.883	0.680	1.146	0.458	0.3486
SOMA_Q5S007_LRRK2_10990_21	2209	0.732	0.673	0.796	4.21E-10	12.549	1077	0.448	0.283	0.709	3.217	0.0006
SOMA_Q99497_PARK7_19545_145	2209	0.73	0.671	0.794	4.40E-10	12.53	1077	1.045	0.914	1.194	0.285	0.5188
SOMA_Q5XLA6_CARD17_18267_74	2209	0.723	0.662	0.789	4.76E-10	12.495	1077	0.984	0.834	1.160	0.072	0.8472
SOMA_P05019_IGF1_2952_75	2209	0.721	0.66	0.787	5.21E-10	12.456	1077	0.803	0.686	0.941	2.185	0.0065
SOMA_Q8N128_FAM177A_8039_41	2209	0.731	0.671	0.795	5.32E-10	12.447	1077	0.574	0.477	0.690	8.422	<0.0001
SOMA_P30305_CDC25B_12427_8	2209	0.794	0.747	0.845	5.56E-10	12.428	1077	0.583	0.436	0.779	3.577	0.0003
SOMA_Q9GZQ8_MAP1LC3_11608_5	2209	1.357	1.25	1.473	5.59E-10	12.425	1077	0.889	0.771	1.025	0.980	0.1048
SOMA_P05160_F13B_5658_64	2209	0.72	0.659	0.787	6.18E-10	12.382	1077	0.688	0.593	0.798	6.100	<0.0001
SOMA_Q16619_CTF1_2889_37	2209	0.813	0.768	0.86	6.20E-10	12.381	1077	1.002	0.857	1.170	0.007	0.9847
SOMA_Q12884_FAP_5029_3	2209	0.725	0.664	0.791	6.38E-10	12.368	1077	0.747	0.606	0.921	2.201	0.0063

SOMA_O75674_TOM1L1_13652_2	2209	0.722	0.661	0.789	6.68E-10	12.348	1077	0.735	0.547	0.989	1.378	0.0419
SOMA_Q96LB9_PGLYRP3_10561_5	2209	0.76	0.706	0.819	7.12E-10	12.32	1077	0.023	0.001	0.546	1.706	0.0197
SOMA_Q5JTV8_TOR1AIP_9039_47	2209	0.743	0.685	0.805	7.18E-10	12.317	1077	0.595	0.501	0.705	8.624	<0.0001
SOMA_Q8IUR0_TRAPPC5_8671_378	2209	0.719	0.658	0.787	7.36E-10	12.306	1077	0.250	0.113	0.552	3.221	0.0006
SOMA_Q08AG7_MZT1_8072_19	2209	0.723	0.662	0.79	8.05E-10	12.267	NA	NA	NA	NA	NA	NA
SOMA_Q9BRX8_FAM213A_13423_94	2209	0.718	0.657	0.786	8.12E-10	12.264	NA	NA	NA	NA	NA	NA
SOMA_Q9UK55_SERPINA_13119_26	2209	0.728	0.668	0.793	8.12E-10	12.263	1077	0.682	0.579	0.804	5.281	<0.0001
SOMA_Q8N565_MREG_19145_4	2209	0.726	0.666	0.792	8.88E-10	12.224	1077	1.002	0.873	1.151	0.010	0.9768
SOMA_P01009_SERPINA_3580_25	2209	1.378	1.263	1.504	8.99E-10	12.219	1077	1.771	1.572	1.996	20.184	<0.0001
SOMA_Q14376_GALE_11457_53	2209	0.731	0.671	0.796	9.22E-10	12.208	1077	0.552	0.435	0.702	5.928	<0.0001
SOMA_P50238_CRIP1_18275_5	2209	0.745	0.687	0.807	9.26E-10	12.206	1077	0.601	0.501	0.720	7.441	<0.0001
SOMA_P07766_CD3E_8069_85	2209	0.735	0.676	0.8	9.67E-10	12.187	1077	0.676	0.579	0.789	6.169	<0.0001
SOMA_P50502_ST13_18387_7	2209	1.387	1.268	1.516	9.79E-10	12.182	1077	1.216	1.066	1.386	2.444	0.0036
SOMA_P02741_CRP_4337_49	2209	1.371	1.258	1.494	9.90E-10	12.177	1077	1.677	1.447	1.944	11.169	<0.0001
SOMA_O15392_BIRC5_3472_40	2209	0.727	0.667	0.793	1.05E-09	12.153	1077	0.751	0.596	0.946	1.817	0.0152
SOMA_P22626_HNRNPA2_5351_52	2209	1.332	1.231	1.441	1.16E-09	12.107	1077	1.032	0.903	1.180	0.191	0.644
SOMA_Q8N5H7_SH2D3C_12704_26	2209	0.727	0.666	0.793	1.19E-09	12.096	1077	0.599	0.367	0.979	1.387	0.041
SOMA_O95831_AIFM1_13424_51	2209	1.353	1.246	1.47	1.26E-09	12.074	NA	NA	NA	NA	NA	NA
SOMA_Q9Y6X2_PIAS3_13513_174	2209	0.723	0.662	0.791	1.32E-09	12.054	1077	0.677	0.444	1.032	1.156	0.0698

SOMA_P01732_CD8A_15462_28	2209	1.371	1.257	1.495	1.33E-09	12.048	1077	1.334	1.210	1.471	8.124	<0.0001
SOMA_P13929_ENO3_16616_137	2209	0.728	0.667	0.794	1.35E-09	12.042	1077	0.844	0.634	1.124	0.609	0.2462
SOMA_P04275_VWF_3050_7	2209	1.354	1.246	1.471	1.44E-09	12.015	1077	1.572	1.431	1.727	20.432	<0.0001
SOMA_Q16762_TST_12663_1	2209	1.361	1.25	1.481	1.44E-09	12.015	1077	1.239	1.098	1.399	3.291	0.0005
SOMA_Q5GLZ8_HERC4_7860_9	2209	0.754	0.697	0.815	1.47E-09	12.006	1077	0.039	0.013	0.121	7.761	<0.0001
SOMA_Q13613_MTMR1_11167_6	2209	0.733	0.673	0.798	1.48E-09	12.002	1077	0.876	0.557	1.377	0.248	0.5653
SOMA_000000_LOC6524_6561_77	2209	0.731	0.671	0.797	1.53E-09	11.989	NA	NA	NA	NA	NA	NA
SOMA_P00746_CFD_2946_52	2209	1.367	1.254	1.489	1.56E-09	11.981	1077	1.551	1.369	1.756	11.331	<0.0001
SOMA_P09228_CST2_4324_33	2209	1.366	1.254	1.489	1.60E-09	11.968	1077	1.206	1.122	1.296	6.419	<0.0001
SOMA_P49796_RGS3_12827_37	2209	0.728	0.667	0.795	1.72E-09	11.938	1077	1.086	0.979	1.204	0.928	0.118
SOMA_Q5GFL6_VWA2_7128_9	2209	0.741	0.683	0.805	1.76E-09	11.928	1077	0.000	0.000	0.000	5.847	<0.0001
SOMA_P42224_STAT1_10370_21	2209	1.341	1.237	1.454	1.77E-09	11.926	1077	1.052	0.921	1.203	0.342	0.4548
SOMA_O15537_RS1_6497_10	2209	0.723	0.661	0.79	1.87E-09	11.902	1077	0.701	0.437	1.126	0.848	0.1418
SOMA_P28067_HLA_DMA_10639_1	2209	1.328	1.228	1.437	1.88E-09	11.899	NA	NA	NA	NA	NA	NA
SOMA_Q9NX46_ADPRHL2_17332_3	2209	1.354	1.245	1.473	1.89E-09	11.895	NA	NA	NA	NA	NA	NA
SOMA_Q8NEQ5_C1orf16_6896_3	2209	0.738	0.678	0.802	1.92E-09	11.89	1077	0.519	0.415	0.651	7.915	<0.0001
SOMA_P35247_SFTPD_19590_46	2209	1.353	1.244	1.471	2.05E-09	11.862	1077	1.385	1.264	1.518	11.507	<0.0001
SOMA_Q96EU7_C1GALT1_5735_54	2209	0.783	0.732	0.838	2.08E-09	11.854	1077	0.777	0.651	0.926	2.304	0.005
SOMA_Q9Y240_CLEC11A_4500_50	2209	1.358	1.248	1.478	2.10E-09	11.851	1077	1.451	1.317	1.599	13.236	<0.0001

SOMA_Q5T4W7_ARTN_2939_10	2209	0.823	0.779	0.868	2.21E-09	11.829	1077	0.977	0.828	1.153	0.105	0.7849
SOMA_Q13247_SRSF6_11573_3	2209	1.362	1.25	1.483	2.23E-09	11.825	1077	1.144	1.063	1.230	3.483	0.0003
SOMA_Q96PJ5_FCRL4_8973_23	2209	0.744	0.685	0.807	2.27E-09	11.817	1077	0.572	0.290	1.127	0.973	0.1063
SOMA_Q6BCY4_CYB5R2_19143_38	2209	1.346	1.24	1.462	2.36E-09	11.8	1077	1.175	1.039	1.329	1.992	0.0102
SOMA_Q86U06_RBM23_11590_5	2209	1.336	1.233	1.448	2.48E-09	11.779	1077	1.036	0.920	1.166	0.254	0.5571
SOMA_Q86VH4_LRRTM4_8646_61	2209	0.735	0.675	0.8	2.48E-09	11.778	1077	0.931	0.722	1.200	0.236	0.5802
SOMA_P43489_TNFRSF4_3730_81	2209	1.349	1.241	1.465	2.62E-09	11.755	1077	1.019	0.914	1.137	0.138	0.7285
SOMA_P46531_NOTCH1_5107_7	2209	0.738	0.678	0.803	2.68E-09	11.745	1077	1.034	0.893	1.199	0.185	0.6537
SOMA_Q9UMF0_ICAM5_8245_27	2209	1.374	1.258	1.501	2.68E-09	11.745	1077	1.348	1.241	1.465	11.841	<0.0001
SOMD P01374 LTA LTB 3506 49 FLAG	2209	0.729	0.668	0.796	2.72E-09	11.739	NA	NA	NA	NA	NA	NA
SOMD Q06643 LTA LTB 3506 49 FLAG	2209	0.729	0.668	0.796	2.72E-09	11.739	NA	NA	NA	NA	NA	NA
SOMA_O14686_MLL2_13623_4	2209	0.735	0.675	0.801	2.76E-09	11.731	NA	NA	NA	NA	NA	NA
SOMA_Q8NC24_RELL2_6600_70	2209	0.733	0.672	0.799	2.77E-09	11.731	1077	0.980	0.819	1.173	0.082	0.8281
SOMA_P48775_TDO2_9880_33	2209	0.742	0.683	0.806	2.79E-09	11.727	1077	0.504	0.402	0.632	8.539	<0.0001
SOMA_O14672_ADAM10_8654_13	2209	0.731	0.669	0.797	2.81E-09	11.723	1077	0.679	0.380	1.213	0.719	0.191
SOMA_Q6MZM0_HEPHL1_6354_13	2209	0.739	0.68	0.804	2.83E-09	11.721	1077	0.567	0.453	0.712	6.037	<0.0001
SOMA_Q16881_TXNRD1_13967_14	2209	1.355	1.245	1.474	2.88E-09	11.714	1077	0.938	0.803	1.097	0.373	0.4237
SOMA_P14550_AKR1A1_4192_10	2209	1.338	1.233	1.45	2.98E-09	11.699	1077	0.981	0.839	1.148	0.089	0.8144
SOMD P02671 FGA FGB 2796 62 PASS	2209	1.321	1.222	1.428	3.12E-09	11.679	NA	NA	NA	NA	NA	NA

SOMD P02675 FGA FGB 2796 62 PASS	2209	1.321	1.222	1.428	3.12E-09	11.679	NA	NA	NA	NA	NA	NA
SOMD P02679 FGA FGB 2796 62 PASS	2209	1.321	1.222	1.428	3.12E-09	11.679	NA	NA	NA	NA	NA	NA
SOMA_Q9Y266_NUDC_8887_21	2209	1.344	1.237	1.459	3.27E-09	11.658	1077	0.964	0.834	1.115	0.204	0.6253
SOMA_P47895_ALDH1A3_9835_16	2209	0.738	0.678	0.804	4.12E-09	11.558	1077	0.524	0.426	0.646	8.876	<0.0001
SOMA_P12872_MLN_5631_83	2209	1.361	1.248	1.484	4.21E-09	11.549	1077	1.128	1.037	1.226	2.318	0.0048
SOMA_Q8IUB2_WFDC3_6384_19	2209	1.329	1.227	1.439	4.43E-09	11.527	1077	0.822	0.701	0.964	1.805	0.0157
SOMA_O14543_SOCS3_11440_58	2209	0.734	0.672	0.8	4.77E-09	11.495	1077	0.338	0.201	0.567	4.386	<0.0001
SOMA_P49862_KLK7_3378_49	2209	0.735	0.674	0.801	4.79E-09	11.492	1077	0.274	0.175	0.430	7.724	<0.0001
SOMA_O14683_TP53I11_13022_20	2209	0.738	0.678	0.804	4.85E-09	11.488	1077	0.465	0.352	0.615	7.152	<0.0001
SOMA_Q96C10_DHX58_14012_17	2209	0.731	0.669	0.798	4.88E-09	11.484	1077	0.000	0.000	0.040	3.164	0.0007
SOMA_P0DMV9_HSPA1B_18901_26	2209	1.349	1.239	1.467	5.55E-09	11.429	1077	1.503	1.369	1.650	16.910	<0.0001
SOMA_Q86VR8_FJX1_7921_65	2209	1.348	1.239	1.466	5.66E-09	11.42	1077	1.338	1.220	1.466	9.313	<0.0001
SOMA_Q9UM47_NOTCH3_5108_72	2209	1.353	1.242	1.474	5.96E-09	11.397	1077	1.537	1.356	1.741	10.813	<0.0001
SOMA_Q00887_PSG9_9335_28	2209	1.341	1.234	1.457	6.18E-09	11.382	1077	0.983	0.817	1.182	0.068	0.8542
SOMA_P06400_RB1_5024_67	2209	0.737	0.677	0.804	6.20E-09	11.381	1077	0.478	0.329	0.694	3.978	0.0001
SOMA_Q9NS98_SEMA3G_5628_21	2209	0.757	0.699	0.819	6.58E-09	11.355	1077	0.793	0.679	0.926	2.473	0.0034
SOMA_P28330_ACADL_17857_6	2209	0.751	0.693	0.814	6.65E-09	11.35	1077	0.858	0.519	1.420	0.259	0.5513
SOMA_O60235_TMPRSS1_6547_83	2209	0.735	0.673	0.802	7.01E-09	11.327	1077	0.824	0.533	1.274	0.417	0.3832
SOMA_P61978_HNRNPK_19333_4	2209	1.353	1.242	1.474	7.23E-09	11.314	1077	0.965	0.834	1.116	0.202	0.6286

SOMA_Q13478_IL18R1_14079_14	2209	1.339	1.233	1.455	7.61E-09	11.291	1077	1.097	1.023	1.175	2.041	0.0091
SOMA_P16519_PCSK2_15440_57	2209	0.737	0.676	0.804	7.74E-09	11.284	1077	1.071	0.986	1.164	0.977	0.1055
SOMA_P83859_QRFP_6463_59	2209	0.734	0.673	0.802	8.07E-09	11.266	1077	0.975	0.802	1.185	0.097	0.7999
SOMA_O95393_BMP10_3587_53	2209	1.332	1.228	1.445	8.09E-09	11.265	1077	1.297	1.194	1.410	9.052	<0.0001
SOMD P05556 ITGA1 I 3503 4 FLAG	2209	1.334	1.229	1.448	8.18E-09	11.26	NA	NA	NA	NA	NA	NA
SOMD P56199 ITGA1 I 3503 4 FLAG	2209	1.334	1.229	1.448	8.18E-09	11.26	NA	NA	NA	NA	NA	NA
SOMA_Q9H999_PANK3_12658_72	2209	0.739	0.678	0.806	8.26E-09	11.256	1077	0.738	0.578	0.942	1.834	0.0146
SOMA_P55285_CDH6_16312_45	2209	0.746	0.686	0.811	8.37E-09	11.25	1077	1.091	0.983	1.211	0.998	0.1005
SOMA_Q14696_MESDC2_15299_102	2209	1.339	1.232	1.455	8.39E-09	11.249	NA	NA	NA	NA	NA	NA
SOMA_P15428_HPGD_4995_16	2209	0.738	0.677	0.805	8.54E-09	11.241	1077	0.514	0.378	0.700	4.643	<0.0001
SOMA_Q16206_ENOX2_13422_66	2209	0.795	0.745	0.849	8.61E-09	11.238	1077	0.728	0.379	1.401	0.466	0.3422
SOMA_Q15363_TMED2_10761_5	2209	1.299	1.206	1.4	8.62E-09	11.237	1077	1.112	1.032	1.198	2.262	0.0055
SOMA_P59861_DEFB131_6934_8	2209	1.334	1.229	1.449	8.97E-09	11.22	NA	NA	NA	NA	NA	NA
SOMA_P23560_BDNF_14047_78	2209	0.748	0.689	0.813	9.58E-09	11.192	1077	0.435	0.346	0.548	11.889	<0.0001
SOMA_Q96PD5_PGLYRP2_5601_2	2209	0.758	0.7	0.82	9.75E-09	11.184	1077	0.482	0.413	0.561	20.165	<0.0001
SOMA_P12644_BMP4_15667_39	2209	1.362	1.247	1.488	1.05E-08	11.15	1077	1.553	1.408	1.712	17.993	<0.0001
SOMA_Q8N104_DEFB106_5664_57	2209	0.736	0.675	0.804	1.05E-08	11.15	1077	0.654	0.353	1.210	0.753	0.1764
SOMA_P35442_THBS2_14111_15	2209	1.341	1.233	1.459	1.07E-08	11.144	1077	1.020	0.905	1.150	0.127	0.7463
SOMA_Q8TE99_ACPL2_6079_59	2209	0.814	0.768	0.863	1.10E-08	11.132	NA	NA	NA	NA	NA	NA

SOMA_Q9Y5G3_PCDHGB1_11872_9	2209	0.741	0.68	0.807	1.13E-08	11.12	1077	0.860	0.581	1.273	0.345	0.4514
SOMA_Q5VWZ2_LYPLAL1_12428_2	2209	0.731	0.668	0.8	1.15E-08	11.112	1077	0.759	0.498	1.156	0.702	0.1988
SOMA_P10827_THRA_12527_50	2209	0.757	0.699	0.82	1.15E-08	11.11	1077	0.550	0.316	0.958	1.458	0.0349
SOMA_O94973_AP2A2_13621_31	2209	1.33	1.226	1.444	1.16E-08	11.109	1077	0.870	0.755	1.003	1.263	0.0546
SOMA_P26715_KLRC1_5629_58	2209	0.726	0.663	0.796	1.16E-08	11.108	1077	1.003	0.872	1.154	0.015	0.965
SOMA_Q96A58_RERG_17822_57	2209	0.743	0.683	0.809	1.28E-08	11.067	1077	0.416	0.159	1.086	1.136	0.0732
SOMA_Q9UQC9_CLCA2_8950_4	2209	0.742	0.681	0.808	1.28E-08	11.065	1077	1.103	0.986	1.235	1.061	0.0869
SOMA_Q8IZP7_HS6ST3_18896_23	2209	0.744	0.684	0.81	1.30E-08	11.06	1077	0.765	0.629	0.931	2.127	0.0075
SOMA_P52272_HNRNPM_12783_29	2209	1.336	1.229	1.452	1.32E-08	11.053	1077	1.039	0.917	1.177	0.259	0.5505
SOMA_O75368_SH3BGRL_12693_2	2209	1.341	1.233	1.459	1.38E-08	11.033	1077	0.787	0.676	0.917	2.686	0.0021
SOMA_P55287_CDH11_7763_25	2209	0.754	0.695	0.818	1.38E-08	11.033	1077	0.190	0.057	0.630	2.182	0.0066
SOMA_Q6UWR7_ENPP6_15579_26	2209	0.748	0.688	0.813	1.41E-08	11.024	1077	0.822	0.541	1.247	0.449	0.3559
SOMA_P49006_MARCKSL_19175_18	2209	1.292	1.2	1.392	1.48E-08	11.004	1077	1.226	1.126	1.336	5.536	<0.0001
SOMA_P56270_MAZ_13436_54	2209	1.326	1.223	1.439	1.53E-08	10.988	1077	1.130	1.066	1.198	4.402	<0.0001
SOMA_Q9UM44_HHLA2_14132_21	2209	0.739	0.678	0.807	1.57E-08	10.977	1077	0.984	0.825	1.175	0.064	0.8625
SOMA_Q6MZW2_FSTL4_9350_3	2209	0.812	0.765	0.862	1.63E-08	10.961	1077	0.576	0.343	0.966	1.436	0.0366
SOMA_Q6AZY7_SCARA3_11292_13	2209	0.749	0.689	0.814	1.66E-08	10.953	1077	1.035	0.932	1.149	0.281	0.5241
SOMA_Q6NUJ2_C11orf8_11116_16	2209	0.742	0.68	0.808	1.70E-08	10.942	1077	0.670	0.580	0.773	7.416	<0.0001
SOMA_P02771_AFP_5792_8	2209	1.324	1.221	1.436	1.78E-08	10.923	1077	0.950	0.726	1.242	0.151	0.706

SOMA_Q8N423_LILRB2_5091_28	2209	1.322	1.219	1.433	1.82E-08	10.912	1077	1.242	1.169	1.320	11.617	<0.0001
SOMA_P02675_FGB_18890_227	2209	0.744	0.683	0.811	1.92E-08	10.889	1077	0.789	0.625	0.996	1.336	0.0461
SOMA_P02786_TFRC_8795_48	2209	1.35	1.237	1.472	1.94E-08	10.885	1077	1.353	1.273	1.438	21.560	<0.0001
SOMA_Q9BRR6_ADPGK_6221_1	2209	1.339	1.23	1.457	2.18E-08	10.834	1077	1.342	1.229	1.465	10.273	<0.0001
SOMA_P06127_CD5_14065_11	2209	0.741	0.679	0.808	2.20E-08	10.831	1077	0.866	0.480	1.563	0.198	0.6332
SOMA_O95867_LY6G6C_6256_9	2209	1.319	1.217	1.43	2.28E-08	10.815	1077	1.056	0.920	1.213	0.358	0.4386
SOMA_Q9NPB0_SAYSD1_6594_64	2209	0.739	0.676	0.807	2.42E-08	10.789	1077	0.617	0.193	1.972	0.382	0.4148
SOMA_P34810_CD68_18922_27	2209	1.325	1.221	1.438	2.43E-08	10.787	1077	1.068	0.973	1.172	0.777	0.1672
SOMA_P41208_CETN2_13078_3	2209	0.756	0.697	0.82	2.48E-08	10.779	1077	0.698	0.557	0.875	2.740	0.0018
SOMA_Q14289_PTK2B_8918_64	2209	0.75	0.69	0.816	2.49E-08	10.777	1077	0.677	0.537	0.854	3.000	0.001
SOMA_Q8TBP5_FAM174A_6597_24	2209	0.749	0.689	0.815	2.58E-08	10.761	1077	0.789	0.484	1.287	0.465	0.3428
SOMA_Q9P0V8_SLAMF8_8994_65	2209	1.328	1.223	1.443	2.59E-08	10.76	1077	1.094	1.039	1.152	3.229	0.0006
SOMA_P01275_GCG_4891_50	2209	1.332	1.225	1.448	2.61E-08	10.757	1077	1.397	1.254	1.557	8.831	<0.0001
SOMA_Q9UNW1_MINPP1_5586_66	2209	1.356	1.241	1.482	2.67E-08	10.746	1077	1.460	1.337	1.593	16.630	<0.0001
SOMA_O75022_LILRB3_11334_7	2209	0.737	0.674	0.806	2.72E-08	10.739	1077	0.399	0.181	0.875	1.659	0.0219
SOMA_P51814_ZNF41_10003_15	2209	0.74	0.678	0.808	2.75E-08	10.733	1077	0.768	0.526	1.119	0.772	0.1691
SOMA_P83916_CBX1_18817_50	2209	1.312	1.212	1.421	2.77E-08	10.731	1077	0.707	0.371	1.349	0.533	0.2932
SOMA_Q6NW40_RGMB_3331_8	2209	1.349	1.236	1.472	2.79E-08	10.727	1077	1.266	1.155	1.387	6.376	<0.0001
SOMA_P08684_CYP3A4_7879_12	2209	0.743	0.681	0.81	2.84E-08	10.72	1077	0.949	0.735	1.224	0.165	0.6846

SOMA_Q96P66_GPR101_11371_1	2209	0.747	0.686	0.813	2.93E-08	10.706	1077	0.814	0.528	1.257	0.451	0.3541
SOMA_Q9Y4K0_LOXL2_6504_65	2209	1.326	1.221	1.44	2.99E-08	10.697	1077	1.124	1.052	1.200	3.257	0.0006
SOMA_A0PJX4_SHISA3_7057_18	2209	1.344	1.233	1.465	3.00E-08	10.695	NA	NA	NA	NA	NA	NA
SOMA_Q8IU85_CAMK1D_3418_12	2209	0.739	0.677	0.808	3.16E-08	10.673	1077	0.590	0.366	0.949	1.527	0.0297
SOMA_Q9HB29_IL1RL2_2994_71	2209	0.792	0.739	0.848	3.27E-08	10.659	1077	0.646	0.482	0.866	2.460	0.0035
SOMA_A6NGZ8_SMIM9_8888_33	2209	0.753	0.692	0.818	3.60E-08	10.616	1077	0.945	0.792	1.127	0.278	0.5273
SOMA_Q9UJW8_ZNF180_12771_19	2209	0.743	0.68	0.81	3.88E-08	10.584	1077	0.881	0.668	1.162	0.431	0.3704
SOMA_O43278_SPINT1_2828_82	2209	0.761	0.702	0.825	3.91E-08	10.581	1077	0.823	0.684	0.989	1.423	0.0377
SOMA_P06729_CD2_7100_31	2209	1.325	1.22	1.439	4.03E-08	10.568	1077	1.245	1.140	1.360	5.966	<0.0001
SOMA_Q9NXV2_KCTD5_12473_48	2209	0.745	0.684	0.813	4.07E-08	10.563	1077	0.678	0.485	0.948	1.640	0.0229
SOMA_P09669_COX6C_8903_1	2209	0.758	0.699	0.823	4.09E-08	10.561	1077	0.097	0.041	0.229	6.990	<0.0001
SOMA_O43687_AKAP7_18399_1	2209	0.749	0.687	0.815	4.20E-08	10.55	1077	0.697	0.528	0.922	1.948	0.0113
SOMA_Q9NPD8_UBE2T_12400_25	2209	0.748	0.687	0.815	4.37E-08	10.532	1077	0.928	0.732	1.177	0.268	0.539
SOMA_Q9HC36_RNMTL1_9584_105	2209	0.749	0.687	0.815	4.56E-08	10.514	NA	NA	NA	NA	NA	NA
SOMA_P55289_CDH12_10701_30	2209	1.321	1.217	1.434	4.69E-08	10.502	NA	NA	NA	NA	NA	NA
SOMA_P05186_ALPL_16926_44	2209	1.334	1.225	1.453	4.78E-08	10.493	1077	1.023	0.920	1.138	0.173	0.6707
SOMA_P49798_RGS4_17855_28	2209	0.743	0.68	0.811	4.84E-08	10.488	1077	0.802	0.492	1.309	0.423	0.3779
SOMA_Q9BXJ4_C1QTNF3_10749_18	2209	1.31	1.209	1.419	4.87E-08	10.485	NA	NA	NA	NA	NA	NA
SOMA_Q5DID0_UMODL1_9114_84	2209	0.744	0.681	0.812	5.01E-08	10.473	1077	0.882	0.615	1.266	0.305	0.4959

SOMA_P27918_CFP_2960_66	2209	0.746	0.684	0.814	5.27E-08	10.451	1077	0.572	0.495	0.662	13.266	<0.0001
SOMA_P78314_SH3BP2_7769_29	2209	1.31	1.209	1.419	5.29E-08	10.449	1077	0.947	0.747	1.201	0.185	0.6538
SOMA_Q08629_SPOCK1_5490_53	2209	0.74	0.676	0.809	5.39E-08	10.441	1077	0.939	0.775	1.138	0.281	0.5234
SOMA_Q16875_PFKFB3_12456_5	2209	0.766	0.708	0.829	5.54E-08	10.429	1077	0.767	0.623	0.944	1.917	0.0121
SOMA_P09237_MMP7_2789_26	2209	1.328	1.221	1.445	5.76E-08	10.413	1077	1.189	1.055	1.341	2.342	0.0046
SOMA_P05231_IL6_4673_13	2209	1.327	1.22	1.444	5.83E-08	10.407	1077	1.096	1.028	1.169	2.299	0.005
SOMA_O00443_PIK3C2A_14028_22	2209	0.769	0.712	0.832	6.14E-08	10.385	1077	0.630	0.454	0.875	2.234	0.0058
SOMA_P40259_CD79B_6351_55	2209	0.753	0.692	0.819	6.51E-08	10.36	1077	0.941	0.762	1.164	0.238	0.5775
SOMA_P00742_F10_3077_66	2209	0.758	0.698	0.823	6.59E-08	10.354	1077	0.727	0.629	0.840	4.818	<0.0001
SOMA_P00742_F10_4878_3	2209	0.758	0.698	0.823	6.67E-08	10.349	1077	0.733	0.635	0.847	4.630	<0.0001
SOMA_P36405_ARL3_12571_14	2209	1.326	1.219	1.443	6.84E-08	10.338	1077	0.962	0.831	1.114	0.216	0.6087
SOMA_P08684_CYP3A4_2943_5	2209	0.758	0.699	0.824	6.86E-08	10.337	1077	0.608	0.508	0.729	7.135	<0.0001
SOMA_Q15526_SURF1_8009_121	2209	0.754	0.693	0.82	6.86E-08	10.337	1077	0.498	0.368	0.674	5.205	<0.0001
SOMA_Q9GZT5_WNT10A_10044_12	2209	0.751	0.69	0.818	6.91E-08	10.333	1077	0.755	0.632	0.903	2.683	0.0021
SOMA_Q08554_DSC1_10882_12	2209	0.827	0.781	0.875	7.13E-08	10.32	1077	0.978	0.804	1.189	0.086	0.8207
SOMA_Q9UBI4_STOML1_17344_23	2209	0.745	0.682	0.813	7.28E-08	10.311	1077	0.280	0.185	0.425	8.683	<0.0001
SOMA_Q9Y581_INSL6_5754_76	2209	0.756	0.695	0.822	7.29E-08	10.31	1077	0.835	0.620	1.123	0.634	0.2321
SOMA_P17096_HMGA1_16536_3	2209	1.302	1.204	1.409	7.30E-08	10.309	1077	1.052	0.964	1.147	0.588	0.2579
SOMA_Q06124_PTPN11_3397_7	2209	1.352	1.236	1.479	7.48E-08	10.299	1077	1.092	0.956	1.247	0.715	0.1928

SOMA_P16112_ACAN_3280_49	2209	0.775	0.719	0.837	7.50E-08	10.298	1077	0.736	0.575	0.942	1.827	0.0149
SOMA_Q9NQS3_PVRL3_13557_3	2209	0.746	0.683	0.814	7.98E-08	10.271	NA	NA	NA	NA	NA	NA
SOMA_Q15561_TEAD4_12516_13	2209	1.318	1.214	1.432	8.01E-08	10.269	1077	0.975	0.796	1.195	0.092	0.8095
SOMA_Q99941_ATF6B_11387_3	2209	0.755	0.695	0.822	8.56E-08	10.24	1077	0.652	0.531	0.800	4.367	<0.0001
SOMA_P62979_RPS27A_4474_19	2209	1.313	1.21	1.424	8.62E-08	10.237	1077	1.050	0.943	1.169	0.423	0.3774
SOMA_Q9Y5H4_PCDHGA1_6457_50	2209	1.329	1.22	1.447	8.78E-08	10.23	1077	1.052	0.981	1.128	0.817	0.1525
SOMA_Q8WV74_NUDT8_7872_5	2209	0.744	0.681	0.813	9.19E-08	10.21	NA	NA	NA	NA	NA	NA
SOMA_Q86UU9_TAC4_6468_37	2209	0.744	0.681	0.813	9.73E-08	10.185	1077	0.774	0.520	1.153	0.683	0.2075
SOMA_Q8WW12_PCNP_17769_28	2209	1.324	1.217	1.441	9.78E-08	10.183	1077	0.938	0.811	1.085	0.412	0.3877
SOMA_P15813_CD1D_8749_194	2209	0.747	0.684	0.815	9.97E-08	10.174	1077	0.914	0.731	1.142	0.368	0.4285
SOMA_P33764_S100A3_15297_3	2209	0.8	0.748	0.856	1.01E-07	10.168	1077	0.025	0.001	0.648	1.580	0.0263
SOMA_Q9UHD0_IL19_3035_80	2209	1.346	1.231	1.472	1.01E-07	10.168	1077	1.435	1.313	1.568	14.778	<0.0001
SOMA_O94907_DKK1_3535_84	2209	0.752	0.69	0.819	1.02E-07	10.165	1077	0.759	0.638	0.904	2.712	0.0019
SOMA_Q9UBP9_GULP1_19180_38	2209	0.754	0.693	0.821	1.05E-07	10.153	1077	0.781	0.672	0.908	2.876	0.0013
SOMA_O95407_TNFRSF6_5070_76	2209	1.317	1.212	1.431	1.10E-07	10.132	1077	1.048	0.953	1.153	0.474	0.3359
SOMA_P18075_BMP7_2972_57	2209	1.326	1.218	1.443	1.10E-07	10.131	1077	1.028	0.940	1.124	0.264	0.5442
SOMA_Q8WXH2_JPH3_9089_77	2209	0.742	0.678	0.812	1.11E-07	10.128	1077	1.080	0.978	1.193	0.892	0.1283
SOMA_Q9Y240_CLEC11A_2966_65	2209	1.332	1.222	1.452	1.13E-07	10.12	1077	1.439	1.309	1.583	13.301	<0.0001
SOMA_P07992_ERCC1_12585_39	2209	0.749	0.687	0.817	1.13E-07	10.118	1077	0.986	0.826	1.177	0.058	0.8753

SOMA_P07477_PRSS1_3049_61	2209	1.316	1.212	1.43	1.15E-07	10.11	1077	1.225	1.119	1.340	4.999	<0.0001
SOMA_Q99759_MAP3K3_12990_39	2209	1.306	1.205	1.415	1.16E-07	10.107	1077	0.811	0.440	1.495	0.300	0.5015
SOMA_Q96C24_SYTL4_11563_51	2209	0.823	0.776	0.873	1.21E-07	10.09	1077	1.026	0.886	1.188	0.138	0.7285
SOMA_O60763_USO1_13639_101	2209	1.303	1.203	1.412	1.23E-07	10.083	1077	0.698	0.468	1.042	1.105	0.0785
SOMA_P14151_SELL_4831_4	2209	0.761	0.7	0.826	1.27E-07	10.07	1077	1.016	0.877	1.176	0.079	0.8341
SOMA_P30101_PDIA3_4719_58	2209	0.741	0.677	0.812	1.29E-07	10.062	1077	0.817	0.693	0.964	1.782	0.0165
SOMA_O43736_ITM2A_7765_15	2209	0.755	0.694	0.822	1.29E-07	10.061	1077	0.957	0.808	1.132	0.217	0.6068
SOMA_P27216_ANXA13_17835_28	2209	0.756	0.695	0.823	1.32E-07	10.051	1077	0.899	0.535	1.511	0.163	0.6865
SOMA_Q8WWK9_CKAP2_5345_51	2209	0.755	0.693	0.822	1.37E-07	10.035	1077	0.635	0.535	0.754	6.658	<0.0001
SOMA_Q5BIV9_SPRN_13447_42	2209	1.328	1.219	1.447	1.41E-07	10.025	1077	1.094	1.020	1.174	1.917	0.0121
SOMA_Q6UW15_REG3G_15476_6	2209	1.313	1.209	1.426	1.41E-07	10.023	1077	1.159	1.080	1.244	4.332	<0.0001
SOMA_P78346_RPP30_17511_10	2209	0.78	0.723	0.841	1.42E-07	10.019	1077	0.295	0.159	0.546	3.992	0.0001
SOMA_Q969W8_ZNF566_12795_2	2209	0.751	0.688	0.819	1.44E-07	10.015	1077	0.760	0.488	1.184	0.648	0.2251
SOMA_Q9NZQ7_CD274_5060_62	2209	1.319	1.213	1.434	1.47E-07	10.006	1077	1.096	1.013	1.186	1.640	0.0229
SOMA_Q969P0_IGSF8_6984_6	2209	1.302	1.202	1.41	1.51E-07	9.994	1077	1.099	1.018	1.186	1.818	0.0152
SOMD P27348 YWHABY 4707 50 PASS	2209	1.316	1.211	1.43	1.55E-07	9.982	NA	NA	NA	NA	NA	NA
SOMD P31946 YWHABY 4707 50 PASS	2209	1.316	1.211	1.43	1.55E-07	9.982	NA	NA	NA	NA	NA	NA
SOMD P31947 YWHABY 4707 50 PASS	2209	1.316	1.211	1.43	1.55E-07	9.982	NA	NA	NA	NA	NA	NA
SOMD P61981 YWHABY 4707 50 PASS	2209	1.316	1.211	1.43	1.55E-07	9.982	NA	NA	NA	NA	NA	NA

SOMD P62258 YWHABY 4707 50 PASS	2209	1.316	1.211	1.43	1.55E-07	9.982	NA	NA	NA	NA	NA	NA
SOMD P63104 YWHABY 4707 50 PASS	2209	1.316	1.211	1.43	1.55E-07	9.982	NA	NA	NA	NA	NA	NA
SOMD Q04917 YWHABY 4707 50 PASS	2209	1.316	1.211	1.43	1.55E-07	9.982	NA	NA	NA	NA	NA	NA
SOMA_Q8N9N8_EIF1AD_13545_97	2209	1.307	1.205	1.418	1.56E-07	9.979	1077	0.976	0.818	1.166	0.101	0.792
SOMA_P55087_AQP4_11363_58	2209	0.751	0.689	0.819	1.59E-07	9.972	1077	0.959	0.749	1.227	0.132	0.7373
SOMA_P04155_TFF1_9185_15	2209	1.328	1.218	1.447	1.63E-07	9.961	1077	1.417	1.289	1.557	12.256	<0.0001
SOMA_Q9BYR9_KRTAP2__14615_46	2209	1.319	1.213	1.435	1.65E-07	9.955	1077	1.099	1.038	1.164	2.884	0.0013
SOMA_Q6UW56_ATRAID_10823_19	2209	1.314	1.209	1.427	1.65E-07	9.954	NA	NA	NA	NA	NA	NA
SOMA_P54727_RAD23B_12522_6	2209	1.314	1.209	1.427	1.69E-07	9.945	1077	0.916	0.792	1.059	0.629	0.235
SOMA_Q9H3S3_TMPRSS5_8002_27	2209	0.812	0.762	0.865	1.70E-07	9.942	1077	0.565	0.180	1.778	0.482	0.3293
SOMA_O15041_SEMA3E_5363_51	2209	1.309	1.206	1.421	1.73E-07	9.936	1077	1.053	0.985	1.125	0.876	0.1329
SOMA_P0CG48_UBC_6647_55	2209	1.33	1.219	1.45	1.93E-07	9.887	1077	1.195	1.079	1.324	3.185	0.0007
SOMA_Q6UWV6_ENPP7_4435_66	2209	1.328	1.218	1.449	2.06E-07	9.86	1077	1.331	1.217	1.456	9.389	<0.0001
SOMA_Q9UBL9_P2RX2_9493_56	2209	0.749	0.685	0.818	2.10E-07	9.85	NA	NA	NA	NA	NA	NA
SOMA_P57738_TCTA_10489_19	2209	0.779	0.722	0.841	2.24E-07	9.822	NA	NA	NA	NA	NA	NA
SOMA_Q9NPI6_DCP1A_14008_22	2209	1.306	1.204	1.417	2.27E-07	9.817	1077	0.980	0.816	1.177	0.082	0.8284
SOMA_P07327_ADH1A_17396_23	2209	0.75	0.687	0.819	2.34E-07	9.803	1077	1.015	0.868	1.187	0.070	0.852
SOMA_Q5VVJ2_MYSM1_11536_9	2209	0.752	0.689	0.821	2.37E-07	9.798	1077	0.180	0.065	0.502	2.984	0.001
SOMA_P10242_MYB_11618_83	2209	0.755	0.692	0.823	2.40E-07	9.793	1077	0.756	0.551	1.036	1.085	0.0822

SOMA_Q8IYS5_OSCAR_7116_31	2209	0.751	0.688	0.82	2.43E-07	9.788	1077	0.952	0.749	1.211	0.161	0.69
SOMA_Q76N89_HECW1_12669_30	2209	0.757	0.695	0.825	2.44E-07	9.786	1077	0.897	0.706	1.139	0.428	0.373
SOMA_P13497_BMP1_3348_49	2209	0.762	0.701	0.829	2.59E-07	9.76	1077	0.485	0.408	0.577	15.582	<0.0001
SOMA_Q02383_SEMG2_6373_54	2209	1.325	1.216	1.445	2.61E-07	9.756	NA	NA	NA	NA	NA	NA
SOMA_P14923_JUP_16768_3	2209	1.3	1.2	1.41	2.62E-07	9.754	1077	0.998	0.863	1.153	0.012	0.9739
SOMA_P42081_CD86_5337_64	2209	0.795	0.741	0.853	2.69E-07	9.744	1077	0.321	0.035	2.955	0.501	0.3158
SOMA_Q9H3W5_LRRN3_10471_25	2209	0.776	0.717	0.839	2.69E-07	9.743	NA	NA	NA	NA	NA	NA
SOMA_P07988_SFTPB_10672_75	2209	1.329	1.218	1.451	2.71E-07	9.74	1077	1.182	1.098	1.272	5.026	<0.0001
SOMA_Q9BV40_VAMP8_7064_2	2209	0.754	0.691	0.822	2.85E-07	9.718	1077	0.001	0.000	48.449	0.707	0.1963
SOMA_Q09FC8_ZNF415_12811_55	2209	0.748	0.684	0.818	2.86E-07	9.716	1077	0.735	0.480	1.125	0.807	0.1559
SOMA_Q96LA6_FCRL1_5728_60	2209	0.752	0.689	0.821	2.88E-07	9.714	1077	0.706	0.561	0.889	2.510	0.0031
SOMA_P36507_MAP2K2_3628_3	2209	1.314	1.208	1.43	2.98E-07	9.698	1077	1.126	1.046	1.211	2.814	0.0015
SOMA_Q8N2Q7_NLGN1_15620_4	2209	1.296	1.197	1.404	3.00E-07	9.696	1077	1.209	1.134	1.288	8.207	<0.0001
SOMA_Q9Y2G1_MYRF_8843_34	2209	0.754	0.691	0.823	3.03E-07	9.691	1077	0.436	0.283	0.672	3.764	0.0002
SOMA_P02743_APCS_2474_54	2209	0.752	0.688	0.821	3.21E-07	9.666	1077	0.557	0.481	0.645	14.361	<0.0001
SOMA_Q9Y653_GPR56_18893_26	2209	1.332	1.219	1.455	3.23E-07	9.664	NA	NA	NA	NA	NA	NA
SOMA_Q9GZZ8_LACRT_7163_26	2209	0.757	0.695	0.825	3.25E-07	9.661	1077	0.641	0.518	0.793	4.373	<0.0001
SOMA_Q15768_EFNB3_7785_1	2209	0.763	0.702	0.829	3.26E-07	9.659	1077	1.018	0.895	1.158	0.104	0.7866
SOMA_P05412_JUN_10356_21	2209	0.757	0.695	0.825	3.27E-07	9.658	1077	0.635	0.473	0.852	2.612	0.0024

SOMA_Q6ZMJ4_IL34_4556_10	2209	0.751	0.687	0.82	3.40E-07	9.642	1077	0.860	0.739	1.001	1.283	0.0521
SOMA_P45985_MAP2K4_5242_37	2209	0.759	0.697	0.826	3.48E-07	9.631	1077	0.654	0.556	0.769	6.527	<0.0001
SOMA_P60842_EIF4A1_18829_4	2209	1.332	1.219	1.456	3.61E-07	9.616	1077	1.145	1.003	1.308	1.343	0.0453
SOMA_P19320_VCAM1_2967_8	2209	1.332	1.219	1.456	3.61E-07	9.615	1077	1.469	1.333	1.619	14.076	<0.0001
SOMA_P56134_ATP5J2_11539_4	2209	0.771	0.711	0.836	3.72E-07	9.603	NA	NA	NA	NA	NA	NA
SOMA_P48788_TNNI2_5440_26	2209	1.313	1.207	1.428	3.75E-07	9.599	1077	1.161	1.060	1.270	2.908	0.0012
SOMA_O60234_GMFG_13062_4	2209	1.321	1.212	1.44	3.83E-07	9.59	1077	0.973	0.823	1.150	0.126	0.7489
SOMA_P00451_F8_13499_30	2209	1.305	1.201	1.417	4.19E-07	9.55	1077	1.863	1.646	2.107	22.300	<0.0001
SOMA_A6NI73_LILRA5_7787_25	2209	1.319	1.21	1.437	4.31E-07	9.539	1077	1.342	1.204	1.495	6.981	<0.0001
SOMA_O14668_PRRG1_8306_54	2209	0.753	0.69	0.823	4.36E-07	9.533	1077	0.970	0.819	1.148	0.143	0.7202
SOMA_Q6UXQ4_C2orf66_5677_15	2209	0.754	0.691	0.823	4.40E-07	9.529	1077	0.287	0.016	5.095	0.403	0.395
SOMA_P0C0P6_NPS_6390_18	2209	0.761	0.699	0.828	4.54E-07	9.516	1077	0.611	0.515	0.724	7.854	<0.0001
SOMA_Q9BTE7_DCUN1D5_8760_10	2209	0.76	0.697	0.828	4.73E-07	9.498	1077	0.464	0.322	0.670	4.389	<0.0001
SOMA_O95969_SCGB1D2_6508_68	2209	0.76	0.697	0.828	4.95E-07	9.478	1077	0.753	0.606	0.936	1.975	0.0106
SOMA_Q02818_NUCB1_10451_11	2209	1.314	1.207	1.431	5.03E-07	9.471	1077	1.086	1.029	1.147	2.544	0.0029
SOMA_Q09028_RBBP4_15331_47	2209	1.308	1.203	1.423	5.16E-07	9.46	1077	1.141	1.033	1.261	2.039	0.0091
SOMA_Q8TAQ9_SUN3_8852_10	2209	0.755	0.691	0.824	5.37E-07	9.443	1077	0.782	0.491	1.245	0.522	0.3004
SOMA_Q96PZ7_CSMD1_9598_23	2209	0.752	0.688	0.822	5.39E-07	9.441	1077	0.466	0.364	0.596	8.894	<0.0001
SOMA_Q92952_KCNN1_13539_131	2209	0.752	0.688	0.822	5.44E-07	9.438	1077	0.890	0.693	1.142	0.444	0.3594

SOMA_O95379_TNFAIP8_12563_2	2209	0.751	0.687	0.821	5.45E-07	9.436	1077	0.571	0.445	0.733	4.943	<0.0001
SOMA_Q96JK2_DCAF5_11283_13	2209	0.757	0.694	0.826	5.50E-07	9.432	1077	0.498	0.303	0.820	2.209	0.0062
SOMA_P49913_CAMP_9384_17	2209	0.76	0.697	0.828	5.64E-07	9.421	1077	0.876	0.743	1.033	0.936	0.116
SOMA_Q86Y97_SUV420H_12452_32	2209	0.762	0.7	0.83	5.64E-07	9.421	NA	NA	NA	NA	NA	NA
SOMA_P35557_GCK_12960_9	2209	0.756	0.693	0.825	5.66E-07	9.42	1077	0.311	0.224	0.431	11.687	<0.0001
SOMA_P09493_TPM1_5033_27	2209	0.75	0.685	0.821	5.67E-07	9.419	1077	1.157	1.073	1.246	3.871	0.0001
SOMA_Q96PB7_OLFM3_11143_32	2209	0.763	0.701	0.831	6.19E-07	9.381	1077	0.160	0.071	0.362	4.982	<0.0001
SOMA_P04183_TK1_4301_58	2209	0.767	0.706	0.834	6.29E-07	9.374	1077	0.750	0.486	1.157	0.713	0.1936
SOMA_P22301_IL10_2773_50	2209	0.826	0.778	0.878	6.79E-07	9.341	1077	0.940	0.759	1.166	0.240	0.5748
SOMA_O14910_LIN7A_18910_45	2209	1.29	1.191	1.398	6.93E-07	9.332	1077	1.001	0.872	1.150	0.007	0.985
SOMA_Q9H299_SH3BGRL_17490_4	2209	1.302	1.198	1.415	6.94E-07	9.332	1077	0.863	0.746	1.000	1.303	0.0498
SOMA_P01042_KNG1_19631_13	2209	0.757	0.694	0.827	7.05E-07	9.325	1077	0.879	0.733	1.056	0.775	0.168
SOMA_O00757_FBP2_9867_23	2209	0.753	0.688	0.823	7.15E-07	9.319	1077	1.028	0.926	1.142	0.220	0.6023
SOMA_Q14012_CAMK1_3592_4	2209	0.769	0.707	0.835	7.24E-07	9.313	1077	0.530	0.429	0.654	8.490	<0.0001
SOMA_P58294_PROK1_2247_20	2209	0.841	0.796	0.888	7.40E-07	9.304	1077	0.974	0.829	1.144	0.126	0.7482
SOMA_Q5W0B1_RNF219_8822_163	2209	0.755	0.691	0.825	7.75E-07	9.284	NA	NA	NA	NA	NA	NA
SOMA_Q9UN71_PCDHGB4_10721_76	2209	1.298	1.195	1.41	8.07E-07	9.266	NA	NA	NA	NA	NA	NA
SOMA_Q6UXG2_KIAA132_10637_50	2209	0.777	0.718	0.842	8.37E-07	9.25	NA	NA	NA	NA	NA	NA
SOMA_P49961_ENTPD1_3182_38	2209	0.756	0.692	0.826	8.62E-07	9.238	1077	0.876	0.652	1.177	0.420	0.3805

SOMA_Q15113_PCOLCE_11237_49	2209	1.305	1.199	1.419	8.60E-07	9.238	1077	1.151	1.009	1.314	1.438	0.0365
SOMA_P36896_ACVR1B_2806_49	2209	0.838	0.792	0.886	8.69E-07	9.234	1077	0.694	0.493	0.975	1.454	0.0351
SOMA_P52209_PGD_4187_49	2209	0.758	0.694	0.827	8.80E-07	9.228	1077	0.811	0.690	0.954	1.952	0.0112
SOMA_Q7Z553_MDGA2_19372_7	2209	0.76	0.697	0.829	8.89E-07	9.224	1077	0.423	0.255	0.701	3.072	0.0008
SOMA_Q04912_MST1R_2640_3	2209	0.755	0.691	0.825	9.23E-07	9.208	1077	0.855	0.722	1.013	1.158	0.0694
SOMA_P03951_F11_2190_55	2209	0.761	0.697	0.83	9.41E-07	9.199	1077	0.622	0.533	0.724	8.924	<0.0001
SOMA_P21854_CD72_7009_8	2209	1.31	1.203	1.428	9.57E-07	9.192	1077	1.073	0.999	1.154	1.269	0.0538
SOMA_O43781_DYRK3_4359_87	2209	0.802	0.748	0.86	9.62E-07	9.19	1077	0.251	0.084	0.748	1.883	0.0131
SOMA_Q99726_SLC30A3_9081_39	2209	0.766	0.703	0.833	9.64E-07	9.189	1077	0.661	0.484	0.901	2.054	0.0088
SOMA_Q9NZU0_FLRT3_13123_3	2209	1.292	1.191	1.401	9.70E-07	9.186	1077	1.168	1.114	1.225	9.891	<0.0001
SOMA_P55103_INHBC_6408_2	2209	0.766	0.704	0.834	9.98E-07	9.174	1077	0.553	0.467	0.654	11.254	<0.0001
SOMA_P56937_HSD17B7_8272_22	2209	0.754	0.689	0.824	1.01E-06	9.169	1077	1.000	0.865	1.155	0.001	0.9973
SOMA_P13693_TPT1_3872_2	2209	1.29	1.19	1.398	1.02E-06	9.166	1077	0.922	0.791	1.074	0.531	0.2943
SOMA_O75381_PEX14_8300_82	2209	1.305	1.199	1.42	1.10E-06	9.132	1077	1.036	0.929	1.157	0.282	0.522
SOMA_P10645_CHGA_8476_11	2209	1.296	1.193	1.408	1.11E-06	9.127	1077	1.723	1.494	1.986	13.142	<0.0001
SOMA_P07492_GRP_5897_58	2209	0.762	0.699	0.831	1.11E-06	9.126	1077	0.967	0.799	1.170	0.138	0.7285
SOMA_O43291_SPINT2_2843_13	2209	1.307	1.2	1.424	1.12E-06	9.124	1077	0.994	0.858	1.151	0.031	0.9321
SOMA_Q9UNZ2_NSFL1C_4250_23	2209	1.3	1.196	1.414	1.12E-06	9.123	1077	0.869	0.747	1.011	1.156	0.0698
SOMA_O14777_NDC80_12730_3	2209	0.767	0.705	0.835	1.16E-06	9.109	1077	0.736	0.576	0.941	1.838	0.0145

SOMA_O14625_CXCL11_3038_9	2209	1.287	1.187	1.394	1.17E-06	9.105	1077	1.227	1.135	1.327	6.501	<0.0001
SOMA_O75962_TRIO_11126_102	2209	0.756	0.691	0.826	1.19E-06	9.096	1077	0.947	0.747	1.202	0.183	0.6555
SOMA_P50222_MEOX2_18259_15	2209	0.763	0.7	0.832	1.19E-06	9.096	1077	0.908	0.711	1.159	0.357	0.439
SOMA_O15467_CCL16_4913_78	2209	1.293	1.191	1.404	1.27E-06	9.07	1077	1.298	1.160	1.453	5.273	<0.0001
SOMA_P11049_CD37_18202_22	2209	0.822	0.773	0.875	1.30E-06	9.059	1077	0.448	0.229	0.874	1.733	0.0185
SOMA_P01036_CST4_14076_74	2209	1.307	1.2	1.424	1.32E-06	9.053	1077	1.044	0.957	1.139	0.479	0.3317
SOMA_P43080_GUCA1A_10008_43	2209	0.768	0.706	0.836	1.38E-06	9.032	1077	0.809	0.528	1.240	0.480	0.3312
SOMA_O43866_CD5L_3293_2	2209	1.308	1.2	1.425	1.40E-06	9.028	1077	1.202	1.069	1.352	2.674	0.0021
SOMA_P38646_HSPA9_13492_44	2209	1.301	1.196	1.415	1.42E-06	9.021	1077	1.117	1.020	1.223	1.767	0.0171
SOMA_P01242_GH2_10978_39	2209	1.293	1.191	1.404	1.45E-06	9.012	1077	1.039	0.957	1.127	0.444	0.36
SOMA_P84095_RHOG_12540_25	2209	0.762	0.698	0.831	1.50E-06	8.997	1077	0.593	0.472	0.744	5.174	<0.0001
SOMA_P05305_EDN1_6495_14	2209	1.292	1.19	1.402	1.50E-06	8.996	1077	1.063	0.985	1.147	0.942	0.1144
SOMA_O60494_CUBN_12904_180	2209	0.829	0.78	0.88	1.54E-06	8.984	1077	0.034	0.003	0.441	2.011	0.0098
SOMA_O95944_NCR2_2734_49	2209	0.84	0.794	0.888	1.55E-06	8.981	1077	1.053	0.965	1.150	0.613	0.244
SOMA_Q04828_AKR1C1_12618_50	2209	0.766	0.703	0.834	1.59E-06	8.972	1077	0.254	0.148	0.438	6.093	<0.0001
SOMA_P37173_TGFBR2_5133_17	2209	0.763	0.699	0.832	1.61E-06	8.965	1077	0.964	0.800	1.161	0.156	0.6988
SOMA_Q9H3S1_SEMA4A_16915_153	2209	0.77	0.708	0.838	1.62E-06	8.964	1077	0.898	0.750	1.075	0.615	0.2428
SOMA_Q14956_GPNMB_8289_8	2209	1.287	1.187	1.396	1.64E-06	8.959	1077	1.151	1.049	1.264	2.521	0.003
SOMA_Q9H7M9_C10orf5_14123_34	2209	1.29	1.188	1.4	1.64E-06	8.957	NA	NA	NA	NA	NA	NA

SOMA_Q92478_CLEC2B_7786_83	2209	0.769	0.706	0.837	1.70E-06	8.942	1077	1.002	0.870	1.154	0.010	0.9776
SOMA_Q5T601_ADGRF1_11243_90	2209	0.772	0.71	0.839	1.72E-06	8.938	1077	0.449	0.155	1.303	0.851	0.1409
SOMA_Q9GZP0_PDGFD_9341_1	2209	1.289	1.188	1.399	1.74E-06	8.933	1077	1.065	0.932	1.216	0.452	0.3535
SOMA_P10720_PF4V1_5663_18	2209	0.758	0.694	0.829	1.81E-06	8.915	1077	1.045	0.947	1.154	0.420	0.3802
SOMA_Q8TCW7_ZPLD1_5590_11	2209	0.773	0.712	0.84	1.97E-06	8.878	1077	1.055	0.944	1.180	0.460	0.3468
SOMA_P40394_ADH7_11377_19	2209	0.763	0.699	0.833	1.99E-06	8.874	1077	1.000	0.864	1.157	0.001	0.9973
SOMA_P80370_DLK1_6496_60	2209	1.306	1.198	1.423	2.01E-06	8.87	1077	1.184	1.101	1.273	5.264	<0.0001
SOMA_Q9UK76_HN1_19335_2	2209	0.763	0.699	0.833	2.02E-06	8.868	NA	NA	NA	NA	NA	NA
SOMA_P24310_COX7A1_8390_25	2209	0.773	0.711	0.84	2.08E-06	8.855	1077	0.759	0.526	1.095	0.853	0.1404
SOMA_Q9H4G4_GLIPR2_15522_2	2209	0.765	0.701	0.834	2.19E-06	8.833	1077	0.833	0.718	0.965	1.817	0.0152
SOMA_O75431_MTX2_8839_4	2209	0.756	0.691	0.828	2.23E-06	8.825	1077	1.010	0.887	1.150	0.056	0.8791
SOMA_P02786_TFRC_6895_1	2209	1.313	1.202	1.434	2.23E-06	8.825	1077	1.158	1.108	1.210	10.048	<0.0001
SOMA_Q8WY21_SORCS1_15636_49	2209	1.3	1.194	1.416	2.26E-06	8.819	1077	1.106	1.046	1.169	3.388	0.0004
SOMA_P21741_MDK_2911_27	2209	1.299	1.194	1.415	2.28E-06	8.815	1077	1.177	1.062	1.304	2.742	0.0018
SOMA_Q8WWQ8_STAB2_3399_31	2209	0.762	0.698	0.832	2.31E-06	8.81	1077	0.674	0.514	0.884	2.360	0.0044
SOMA_O75914_PAK3_3387_1	2209	0.763	0.698	0.833	2.32E-06	8.808	1077	0.698	0.399	1.223	0.679	0.2093
SOMA_O60479_DLX3_11422_2	2209	0.78	0.72	0.846	2.42E-06	8.789	1077	0.332	0.082	1.344	0.913	0.1223
SOMA_Q13018_PLA2R1_10916_44	2209	1.297	1.192	1.411	2.44E-06	8.786	1077	1.452	1.279	1.649	8.035	<0.0001
SOMA_Q9NS85_CA10_13666_222	2209	0.771	0.708	0.839	2.44E-06	8.786	1077	0.665	0.498	0.887	2.256	0.0055

SOMA_P21333_FLNA_12906_137	2209	1.302	1.195	1.419	2.51E-06	8.773	NA	NA	NA	NA	NA	NA
SOMA_O00241_SIRPB1_6247_9	2209	1.297	1.192	1.411	2.62E-06	8.755	1077	1.332	1.211	1.465	8.449	<0.0001
SOMA_Q9UKM7_MAN1B1_7071_23	2209	0.788	0.729	0.852	2.66E-06	8.748	1077	0.765	0.657	0.891	3.229	0.0006
SOMA_Q02223_TNFRSF1_2665_26	2209	1.301	1.194	1.418	2.80E-06	8.726	1077	1.129	1.057	1.205	3.543	0.0003
SOMA_O75354_ENTPD6_8932_1	2209	0.763	0.699	0.834	2.84E-06	8.72	1077	0.768	0.550	1.075	0.907	0.1237
SOMA_Q13838_DDX39B_9742_59	2209	1.281	1.182	1.389	2.85E-06	8.718	1077	1.082	0.958	1.223	0.688	0.2051
SOMA_Q9Y639_NPTN_7194_36	2209	1.291	1.187	1.403	2.91E-06	8.709	1077	1.226	1.121	1.342	5.035	<0.0001
SOMA_Q9UHL0_DDX25_13984_23	2209	0.758	0.692	0.83	2.99E-06	8.697	1077	0.817	0.562	1.187	0.540	0.2886
SOMA_Q8N257_HIST3H2_18823_52	2209	1.283	1.183	1.392	3.13E-06	8.677	NA	NA	NA	NA	NA	NA
SOMA_Q9H900_ZWILCH_18302_204	2209	0.772	0.709	0.84	3.18E-06	8.671	1077	0.315	0.162	0.612	3.192	0.0006
SOMA_A8MVW5_HEPACAM_9116_28	2209	0.758	0.692	0.83	3.19E-06	8.669	1077	1.110	1.026	1.201	2.026	0.0094
SOMA_Q08211_DHX9_10527_22	2209	0.81	0.756	0.868	3.22E-06	8.665	1077	0.940	0.768	1.150	0.263	0.5462
SOMA_Q9Y6J6_KCNE2_10427_2	2209	1.303	1.195	1.421	3.23E-06	8.663	NA	NA	NA	NA	NA	NA
SOMA_P11441_UBL4A_11490_42	2209	1.277	1.178	1.383	3.24E-06	8.662	1077	0.850	0.731	0.988	1.458	0.0348
SOMA_P27348_YWHAQ_7625_27	2209	1.298	1.192	1.414	3.26E-06	8.66	1077	0.874	0.724	1.054	0.800	0.1587
SOMA_P11473_VDR_10023_32	2209	0.788	0.728	0.852	3.28E-06	8.657	1077	0.515	0.360	0.736	3.558	0.0003
SOMA_Q13588_GRAP_12820_1	2209	0.772	0.709	0.84	3.31E-06	8.653	1077	0.772	0.638	0.935	2.098	0.008
SOMA_A6NC05_C5orf63_13378_80	2209	0.781	0.72	0.847	3.31E-06	8.652	1077	0.067	0.010	0.462	2.212	0.0061
SOMA_Q9UK00_C3orf18_6994_19	2209	0.771	0.708	0.84	3.34E-06	8.649	1077	0.770	0.651	0.911	2.638	0.0023

SOMA_O95255_ABCC6_8935_22	2209	0.767	0.703	0.837	3.42E-06	8.639	1077	0.974	0.819	1.158	0.117	0.7635
SOMA_Q9UGM5_FETUB_3367_8	2209	0.78	0.718	0.846	3.43E-06	8.638	1077	0.624	0.532	0.731	8.288	<0.0001
SOMA_Q8WXI8_CLEC4D_7752_31	2209	0.762	0.697	0.833	3.48E-06	8.631	1077	0.529	0.298	0.939	1.530	0.0295
SOMA_P35813_PPM1A_12619_14	2209	1.307	1.197	1.427	3.49E-06	8.63	1077	1.065	0.980	1.158	0.862	0.1375
SOMA_O00338_SULT1C2_17811_78	2209	0.773	0.711	0.841	3.54E-06	8.624	1077	0.413	0.206	0.828	1.894	0.0128
SOMA_P05543_SERPINA_2706_69	2209	0.771	0.708	0.84	3.59E-06	8.618	1077	0.849	0.732	0.985	1.507	0.0311
SOMA_P25311_AZGP1_9312_8	2209	1.307	1.197	1.427	3.70E-06	8.604	1077	1.450	1.292	1.627	9.576	<0.0001
SOMA_Q16778_HIST2H2_14143_8	2209	1.283	1.182	1.393	3.75E-06	8.599	NA	NA	NA	NA	NA	NA
SOMA_Q99584_S100A13_7223_60	2209	1.308	1.198	1.429	3.82E-06	8.591	1077	1.425	1.319	1.539	18.522	<0.0001
SOMA_Q8WXF3_RLN3_6027_31	2209	0.771	0.708	0.84	3.88E-06	8.584	1077	0.581	0.411	0.823	2.660	0.0022
SOMA_Q06136_KDSR_19483_16	2209	0.771	0.708	0.84	4.02E-06	8.569	1077	0.912	0.505	1.645	0.120	0.7586
SOMA_P13796_LCP1_17231_1	2209	0.773	0.71	0.841	4.05E-06	8.565	1077	1.021	0.882	1.183	0.108	0.7797
SOMA_P01024_C3_4900_8	2209	0.766	0.702	0.837	4.09E-06	8.561	1077	0.941	0.810	1.094	0.370	0.427
SOMA_Q13907_IDI1_17712_7	2209	1.298	1.191	1.415	4.15E-06	8.555	1077	1.310	1.155	1.486	4.573	<0.0001
SOMA_P01579_IFNG_15346_31	2209	0.771	0.708	0.84	4.19E-06	8.551	1077	0.692	0.508	0.943	1.706	0.0197
SOMA_Q9Y473_ZNF175_12716_3	2209	0.765	0.7	0.836	4.25E-06	8.544	1077	0.899	0.734	1.102	0.514	0.3059
SOMA_Q9P2E7_PCDH10_8780_2	2209	0.764	0.699	0.835	4.29E-06	8.541	1077	0.744	0.597	0.927	2.073	0.0085
SOMA_P05154_SERPINA_3389_7	2209	0.775	0.713	0.843	4.29E-06	8.54	1077	0.550	0.486	0.622	20.684	<0.0001
SOMA_Q08050_FOXM1_10056_5	2209	1.278	1.179	1.386	4.33E-06	8.536	1077	0.534	0.203	1.408	0.689	0.2045

SOMA_Q8NG35_DEFB105_10962_46	2209	0.82	0.768	0.876	4.34E-06	8.535	NA	NA	NA	NA	NA	NA
SOMA_Q8N387_MUC15_6417_55	2209	1.301	1.192	1.419	4.39E-06	8.53	NA	NA	NA	NA	NA	NA
SOMA_Q16548_BCL2A1_3413_50	2209	0.771	0.707	0.84	4.48E-06	8.522	1077	0.723	0.566	0.923	2.029	0.0094
SOMA_Q96QV1_HHIP_10833_64	2209	0.77	0.706	0.84	4.51E-06	8.519	1077	0.714	0.574	0.889	2.593	0.0026
SOMA_Q8N0X7_SPG20_11161_5	2209	1.275	1.177	1.382	4.54E-06	8.516	NA	NA	NA	NA	NA	NA
SOMA_Q9UQE7_SMC3_14324_52	2209	1.276	1.177	1.382	4.57E-06	8.513	1077	1.065	0.933	1.216	0.459	0.3476
SOMA_Q86UD1_OAF_6414_8	2209	1.266	1.171	1.369	4.58E-06	8.512	1077	1.520	1.341	1.723	10.218	<0.0001
SOMA_O00515_LAD1_6407_63	2209	0.778	0.716	0.846	4.61E-06	8.509	1077	0.400	0.156	1.027	1.244	0.057
SOMA_P13686_ACP5_3232_28	2209	0.771	0.708	0.84	4.66E-06	8.504	1077	0.793	0.667	0.943	2.060	0.0087
SOMA_P51649_ALDH5A1_17792_158	2209	0.784	0.724	0.85	4.68E-06	8.503	1077	0.219	0.109	0.441	4.671	<0.0001
SOMA_Q92838_EDA_2826_53	2209	0.778	0.717	0.846	4.71E-06	8.5	1077	1.068	0.979	1.164	0.857	0.1391
SOMA_P01213_PDYN_19638_9	2209	0.775	0.712	0.843	4.92E-06	8.481	1077	0.609	0.518	0.715	8.772	<0.0001
SOMA_Q9NZH6_IL37_2723_9	2209	0.765	0.7	0.836	4.96E-06	8.478	1077	0.791	0.679	0.922	2.574	0.0027
SOMA_P54278_PMS2_11312_40	2209	0.764	0.699	0.836	5.38E-06	8.442	1077	0.322	0.138	0.752	2.051	0.0089
SOMA_Q9BWE0_REPIN1_13554_78	2209	0.77	0.706	0.84	5.43E-06	8.438	1077	0.469	0.149	1.475	0.709	0.1953
SOMA_Q9NYZ4_SIGLEC8_7864_3	2209	0.767	0.703	0.838	5.43E-06	8.438	1077	0.760	0.490	1.178	0.659	0.2193
SOMA_Q5J8M3_EMC4_13516_46	2209	0.778	0.716	0.846	5.44E-06	8.437	1077	0.515	0.379	0.701	4.607	<0.0001
SOMA_Q7Z3B1_NEGR1_7050_5	2209	1.304	1.194	1.424	5.48E-06	8.434	1077	1.095	1.007	1.191	1.477	0.0334
SOMA_Q9BX67_JAM3_2998_53	2209	1.278	1.178	1.387	5.54E-06	8.429	1077	0.830	0.713	0.965	1.815	0.0153

SOMD P07357 C8A C8B 2429 27 FLAG	2209	0.766	0.701	0.837	5.59E-06	8.425	NA	NA	NA	NA	NA	NA
SOMD P07358 C8A C8B 2429 27 FLAG	2209	0.766	0.701	0.837	5.59E-06	8.425	NA	NA	NA	NA	NA	NA
SOMD P07360 C8A C8B 2429 27 FLAG	2209	0.766	0.701	0.837	5.59E-06	8.425	NA	NA	NA	NA	NA	NA
SOMA_P68431_HIST1H3_14146_92	2209	1.278	1.178	1.387	5.65E-06	8.421	NA	NA	NA	NA	NA	NA
SOMA_O95256_IL18RAP_2993_1	2209	0.84	0.793	0.89	5.69E-06	8.418	1077	1.077	1.013	1.145	1.751	0.0177
SOMA_Q96LR4_FAM19A4_6511_17	2209	0.769	0.704	0.839	5.79E-06	8.41	NA	NA	NA	NA	NA	NA
SOMA_O76036_NCR1_8360_169	2209	1.294	1.188	1.41	5.91E-06	8.401	1077	1.038	0.948	1.136	0.373	0.424
SOMA_P20783_NTF3_4145_58	2209	1.289	1.184	1.403	5.96E-06	8.398	1077	1.138	1.071	1.210	4.470	<0.0001
SOMA_O00330_PDHX_18319_7	2209	1.282	1.18	1.392	6.17E-06	8.383	1077	0.961	0.821	1.125	0.206	0.6223
SOMA_P15260_IFNGR1_5825_49	2209	1.297	1.19	1.415	6.21E-06	8.38	1077	0.986	0.834	1.166	0.059	0.872
SOMA_Q8NBJ7_SUMF2_6069_71	2209	1.274	1.175	1.382	6.35E-06	8.37	1077	1.232	1.153	1.317	9.122	<0.0001
SOMA_P21757_MSR1_11207_3	2209	0.789	0.728	0.854	6.40E-06	8.366	1077	0.971	0.813	1.159	0.130	0.742
SOMA_Q14213_EBI3_10851_77	2209	1.293	1.187	1.41	6.52E-06	8.359	1077	1.205	1.131	1.284	8.053	<0.0001
SOMA_Q15582_TGFBI_3283_21	2209	1.301	1.192	1.421	6.58E-06	8.355	1077	1.243	1.090	1.417	2.935	0.0012
SOMA_O95157_NXPH3_6054_6	2209	0.782	0.721	0.849	6.64E-06	8.351	1077	0.694	0.555	0.869	2.850	0.0014
SOMA_P08069_IGF1R_4232_19	2209	1.299	1.19	1.418	7.19E-06	8.316	1077	1.592	1.409	1.798	13.129	<0.0001
SOMA_Q9UMR7_CLEC4A_8853_2	2209	0.786	0.725	0.852	7.22E-06	8.315	1077	1.139	1.045	1.240	2.538	0.0029
SOMA_Q9HAU4_SMURF2_13985_12	2209	0.758	0.69	0.831	7.31E-06	8.309	1077	0.955	0.777	1.175	0.177	0.665
SOMA_P49703_ARL4D_18413_24	2209	1.285	1.181	1.398	7.35E-06	8.307	1077	0.967	0.765	1.222	0.108	0.7793

SOMA_P28066_PSMA5_18925_24	2209	0.784	0.722	0.85	7.37E-06	8.306	1077	0.627	0.538	0.731	8.635	<0.0001
SOMA_O95544_NADK_13624_17	2209	1.291	1.185	1.407	7.58E-06	8.293	1077	1.026	0.921	1.143	0.192	0.6434
SOMA_Q9HCU0_CD248_15491_20	2209	1.286	1.182	1.399	7.77E-06	8.282	1077	1.416	1.273	1.575	9.810	<0.0001
SOMA_Q8TEB7_RNF128_8633_18	2209	0.769	0.705	0.84	7.88E-06	8.276	1077	0.735	0.559	0.966	1.567	0.0271
SOMA_Q969E3_UCN3_10756_34	2209	1.275	1.175	1.383	7.98E-06	8.271	1077	1.059	0.949	1.182	0.515	0.3054
SOMA_O43567_RNF13_8087_250	2209	0.778	0.714	0.846	8.16E-06	8.261	1077	0.355	0.240	0.524	6.703	<0.0001
SOMA_Q15303_ERBB4_2618_10	2209	0.842	0.795	0.892	8.19E-06	8.26	1077	0.677	0.440	1.042	1.117	0.0764
SOMA_O43761_SYNGR3_13375_48	2209	0.77	0.705	0.841	8.43E-06	8.247	1077	0.466	0.151	1.432	0.739	0.1825
SOMA_Q13522_PPP1R1A_17706_4	2209	1.297	1.188	1.416	8.58E-06	8.239	1077	1.200	1.115	1.292	5.894	<0.0001
SOMA_P02751_FN1_3434_34	2209	1.278	1.177	1.388	8.77E-06	8.23	1077	0.963	0.823	1.128	0.193	0.641
SOMA_Q15700_DLG2_19620_16	2209	1.266	1.169	1.371	8.83E-06	8.227	1077	0.790	0.677	0.922	2.550	0.0028
SOMA_Q0D2K0_NIPAL4_12864_9	2209	0.772	0.707	0.842	8.89E-06	8.224	1077	0.340	0.090	1.289	0.949	0.1125
SOMA_P26842_CD27_5412_53	2209	0.795	0.736	0.859	8.92E-06	8.223	1077	0.578	0.465	0.718	6.161	<0.0001
SOMA_Q13477_MADCAM1_11258_41	2209	0.774	0.71	0.844	8.91E-06	8.223	1077	0.688	0.531	0.891	2.332	0.0047
SOMA_P37837_TALDO1_11347_9	2209	1.314	1.199	1.441	9.04E-06	8.217	1077	1.316	1.186	1.461	6.597	<0.0001
SOMA_Q96NI6_LRFN5_6587_6	2209	0.769	0.704	0.84	9.11E-06	8.213	1077	0.776	0.491	1.227	0.556	0.2783
SOMA_O14732_IMPA2_12581_39	2209	0.771	0.707	0.842	9.31E-06	8.204	1077	1.006	0.867	1.167	0.028	0.937
SOMA_Q14498_RBM39_4284_18	2209	1.268	1.17	1.374	9.65E-06	8.188	1077	1.049	0.940	1.171	0.408	0.3909
SOMA_P48745_NOV_2737_22	2209	0.797	0.738	0.86	9.69E-06	8.186	NA	NA	NA	NA	NA	NA

SOMA_Q9BYC5_FUT8_8244_16	2209	0.776	0.713	0.846	9.73E-06	8.185	1077	0.598	0.446	0.802	3.226	0.0006
SOMA_Q9HB63_NTN4_3327_27	2209	1.302	1.191	1.423	1.02E-05	8.165	1077	1.356	1.270	1.448	19.091	<0.0001
SOMA_P01833_PIGR_3216_2	2209	1.292	1.184	1.408	1.03E-05	8.16	1077	1.235	1.147	1.330	7.689	<0.0001
SOMA_Q8N6C8_LILRA3_6391_52	2209	1.277	1.176	1.387	1.03E-05	8.16	1077	1.418	1.272	1.580	9.556	<0.0001
SOMA_P28074_PSMB5_12580_7	2209	0.773	0.708	0.843	1.03E-05	8.159	1077	1.087	1.009	1.172	1.548	0.0283
SOMA_O00238_BMPR1B_10550_37	2209	0.821	0.768	0.878	1.04E-05	8.155	1077	0.942	0.736	1.205	0.199	0.6326
SOMA_P60903_S100A10_15318_75	2209	0.776	0.713	0.846	1.05E-05	8.152	1077	0.594	0.398	0.885	1.976	0.0106
SOMA_P80370_DLK1_8380_244	2209	0.778	0.715	0.847	1.05E-05	8.152	1077	0.982	0.828	1.163	0.081	0.8303
SOMA_P13725_OSM_2693_20	2209	0.77	0.705	0.841	1.08E-05	8.138	1077	0.894	0.734	1.090	0.570	0.2691
SOMA_Q96BD6_SPSB1_13942_140	2209	0.777	0.713	0.846	1.09E-05	8.136	1077	0.701	0.535	0.919	2.000	0.01
SOMA_P54819_AK2_11368_32	2209	1.27	1.171	1.377	1.11E-05	8.129	1077	0.850	0.739	0.978	1.639	0.023
SOMA_Q96KJ9_COX4I2_7850_1	2209	0.769	0.703	0.841	1.19E-05	8.096	1077	0.835	0.647	1.077	0.781	0.1656
SOMA_Q8N3J6_CADM2_16907_3	2209	1.292	1.184	1.409	1.21E-05	8.091	1077	1.064	0.999	1.133	1.260	0.0549
SOMA_O15068_MCF2L_13934_3	2209	0.795	0.735	0.859	1.23E-05	8.084	1077	0.779	0.576	1.052	0.986	0.1032
SOMA_P36268_GGT2_6334_9	2209	0.777	0.713	0.846	1.24E-05	8.079	1077	0.582	0.421	0.805	2.964	0.0011
SOMA_P0C6S8_LINGO3_10827_67	2209	0.83	0.779	0.884	1.24E-05	8.078	1077	0.934	0.754	1.155	0.278	0.5273
SOMA_Q07507_DPT_4979_34	2209	1.272	1.172	1.381	1.29E-05	8.063	1077	1.046	0.927	1.181	0.330	0.4681
SOMA_Q9UP52_TFR2_11537_12	2209	0.778	0.714	0.847	1.30E-05	8.061	1077	0.950	0.707	1.277	0.135	0.7332
SOMA_Q9UM21_MGAT4A_9392_43	2209	0.808	0.751	0.869	1.34E-05	8.045	1077	0.245	0.062	0.969	1.347	0.0449

SOMA_Q9UN67_PCDHB10_9963_19	2209	0.789	0.728	0.856	1.35E-05	8.042	1077	1.023	0.909	1.151	0.150	0.7081
SOMA_Q9BZX2_UCK2_12515_45	2209	0.801	0.743	0.864	1.36E-05	8.04	1077	0.798	0.385	1.656	0.264	0.545
SOMA_Q6UWL6_KIRREL2_16609_106	2209	1.278	1.175	1.39	1.41E-05	8.022	1077	1.018	0.915	1.133	0.131	0.7398
SOMA_P54725_RAD23A_10058_1	2209	1.286	1.18	1.402	1.43E-05	8.016	1077	0.925	0.800	1.069	0.537	0.2907
SOMA_Q9UJQ7_SCP2D1_14175_78	2209	1.286	1.18	1.401	1.45E-05	8.012	1077	1.144	1.073	1.220	4.440	<0.0001
SOMA_Q96LZ2_MAGEB10_11456_2	2209	0.777	0.713	0.847	1.45E-05	8.01	1077	0.866	0.653	1.149	0.498	0.3177
SOMA_P01037_CST1_5459_33	2209	1.287	1.181	1.403	1.47E-05	8.006	1077	1.121	1.027	1.223	1.971	0.0107
SOMA_Q12860_CNTN1_2974_61	2209	0.779	0.715	0.848	1.47E-05	8.006	1077	0.795	0.680	0.928	2.426	0.0037
SOMA_Q9HAP6_LIN7B_15612_5	2209	1.278	1.175	1.39	1.48E-05	8.004	1077	0.736	0.419	1.295	0.541	0.2878
SOMA_P19367_HK1_13131_5	2209	0.775	0.71	0.845	1.48E-05	8.002	1077	0.768	0.600	0.982	1.448	0.0356
SOMA_Q8TAC9_SCAMP5_13509_5	2209	0.771	0.706	0.843	1.49E-05	8.001	1077	0.863	0.582	1.281	0.332	0.4655
SOMA_O14929_HAT1_19327_31	2209	0.776	0.712	0.846	1.49E-05	8	1077	0.930	0.789	1.096	0.414	0.3851
SOMA_P49459_UBE2A_17743_14	2209	0.824	0.771	0.88	1.53E-05	7.988	1077	0.966	0.808	1.155	0.152	0.7046
SOMA_O75477_ERLIN1_8776_10	2209	0.771	0.705	0.843	1.58E-05	7.974	1077	0.562	0.428	0.740	4.420	<0.0001
SOMA_P53634_CTSC_3178_5	2209	0.83	0.778	0.884	1.59E-05	7.973	1077	0.391	0.124	1.235	0.960	0.1097
SOMA_P10966_CD8B_9310_2	2209	0.784	0.722	0.852	1.61E-05	7.967	1077	0.524	0.362	0.760	3.192	0.0006
SOMA_P78324_SIRPA_5430_66	2209	1.263	1.166	1.368	1.65E-05	7.956	1077	1.447	1.256	1.667	6.505	<0.0001
SOMA_Q9NS18_GLRX2_12486_8	2209	1.272	1.171	1.382	1.74E-05	7.931	1077	1.070	0.967	1.184	0.725	0.1883
SOMA_Q96GD4_AURKB_3346_72	2209	0.774	0.708	0.845	1.75E-05	7.929	1077	0.680	0.461	1.003	1.283	0.0521

SOMA_P09486_SPARC_3043_49	2209	0.789	0.728	0.856	1.76E-05	7.928	1077	0.597	0.508	0.701	9.475	<0.0001
SOMA_Q9Y2G5_POFUT2_6042_52	2209	1.261	1.165	1.366	1.77E-05	7.924	NA	NA	NA	NA	NA	NA
SOMA_Q6S5H5_POTEG_7960_53	2209	0.783	0.72	0.852	1.81E-05	7.916	1077	0.556	0.179	1.724	0.510	0.3093
SOMA_Q9UJ37_ST6GALN_7823_22	2209	0.775	0.709	0.846	1.82E-05	7.913	1077	0.837	0.693	1.011	1.191	0.0644
SOMA_Q9UBB9_TFIP11_9043_7	2209	0.779	0.715	0.849	1.82E-05	7.912	1077	0.877	0.705	1.092	0.617	0.2413
SOMA_Q9Y458_TBX22_11146_4	2209	1.269	1.169	1.378	1.83E-05	7.911	1077	1.005	0.877	1.153	0.027	0.9393
SOMA_P26992_CNTFR_14101_2	2209	1.278	1.175	1.391	1.90E-05	7.895	1077	1.202	1.109	1.302	5.112	<0.0001
SOMA_P62837_UBE2D2_18842_24	2209	0.781	0.717	0.85	1.93E-05	7.887	1077	1.072	0.966	1.189	0.714	0.1934
SOMA_Q9UBL0_ARPP21_12860_7	2209	0.774	0.709	0.845	1.94E-05	7.885	1077	0.834	0.560	1.243	0.429	0.3725
SOMA_Q6ZNG9_KRBA2_11917_8	2209	0.813	0.757	0.873	1.98E-05	7.876	NA	NA	NA	NA	NA	NA
SOMA_Q8IUC8_GALNT13_10908_2	2209	0.81	0.753	0.871	1.98E-05	7.876	1077	0.389	0.112	1.349	0.865	0.1366
SOMA_Q96KP4_CNDP2_3192_3	2209	0.774	0.708	0.845	1.98E-05	7.875	1077	0.653	0.388	1.100	0.962	0.1092
SOMA_Q9H477_RBKS_19467_3	2209	0.845	0.798	0.896	2.00E-05	7.871	1077	0.957	0.763	1.202	0.151	0.7066
SOMA_Q9UQQ1_NAALADL_17505_125	2209	0.781	0.717	0.85	2.01E-05	7.869	1077	0.740	0.478	1.146	0.752	0.1771
SOMA_P49914_MTHFS_14107_1	2209	1.268	1.168	1.376	2.02E-05	7.867	1077	0.940	0.813	1.087	0.393	0.4044
SOMA_O95460_MATN4_7083_74	2209	0.773	0.708	0.845	2.03E-05	7.865	1077	0.327	0.206	0.521	5.611	<0.0001
SOMA_P78368_CSNK1G2_12653_13	2209	0.777	0.712	0.848	2.07E-05	7.858	1077	0.834	0.645	1.077	0.786	0.1638
SOMA_O95295_SNAPIN_6975_52	2209	0.806	0.748	0.868	2.07E-05	7.857	1077	0.375	0.085	1.658	0.708	0.1958
SOMA_P02458_COL2A1_18875_125	2209	0.779	0.714	0.849	2.15E-05	7.84	1077	0.497	0.373	0.663	5.697	<0.0001

SOMA_Q9Y3C8_UFC1_3405_6	2209	1.273	1.171	1.384	2.15E-05	7.84	1077	0.903	0.783	1.041	0.797	0.1597
SOMA_O43609_SPRY1_9512_24	2209	0.776	0.711	0.847	2.17E-05	7.837	NA	NA	NA	NA	NA	NA
SOMA_Q13951_CBFB_10048_7	2209	1.285	1.178	1.401	2.20E-05	7.831	1077	1.046	0.927	1.180	0.334	0.464
SOMA_P48723_HSPA13_17515_6	2209	1.252	1.158	1.353	2.22E-05	7.826	1077	1.163	1.082	1.250	4.409	<0.0001
SOMA_Q9NRX6_TMEM167_13421_17	2209	0.775	0.71	0.847	2.26E-05	7.819	1077	0.862	0.571	1.301	0.320	0.479
SOMA_Q14155_ARHGEF7_13932_45	2209	0.799	0.739	0.863	2.29E-05	7.812	1077	0.845	0.610	1.169	0.510	0.3089
SOMA_Q9H972_C14orf9_6439_59	2209	0.787	0.724	0.855	2.41E-05	7.791	1077	0.681	0.576	0.804	5.230	<0.0001
SOMA_Q15768_EFNB3_2514_65	2209	1.266	1.167	1.375	2.41E-05	7.79	1077	1.112	1.021	1.210	1.842	0.0144
SOMA_Q9NYL9_TMOD3_12861_13	2209	1.247	1.155	1.346	2.54E-05	7.768	1077	0.702	0.592	0.833	4.303	<0.0001
SOMA_Q9UGT4_SUSD2_10021_1	2209	0.78	0.715	0.85	2.54E-05	7.767	NA	NA	NA	NA	NA	NA
SOMA_P51553_IDH3G_19275_68	2209	1.267	1.167	1.375	2.58E-05	7.762	1077	1.222	1.104	1.352	3.966	0.0001
SOMA_P22087_FBL_13994_1	2209	0.78	0.716	0.851	2.61E-05	7.757	1077	0.653	0.449	0.951	1.582	0.0262
SOMA_Q15653_NFKBIB_17387_27	2209	0.771	0.704	0.844	2.69E-05	7.743	1077	0.636	0.329	1.229	0.749	0.1782
SOMA_Q8NFT8_DNER_9769_48	2209	0.785	0.722	0.854	2.69E-05	7.743	1077	0.579	0.314	1.069	1.094	0.0805
SOMA_O14796_SH2D1B_18840_205	2209	0.822	0.768	0.88	2.70E-05	7.741	1077	0.107	0.011	1.071	1.242	0.0572
SOMA_P08833_IGFBP1_2771_35	2209	1.27	1.168	1.38	2.80E-05	7.726	1077	1.225	1.130	1.329	6.061	<0.0001
SOMA_P11021_HSPA5_16588_10	2209	1.267	1.167	1.376	2.80E-05	7.726	1077	1.342	1.229	1.466	10.233	<0.0001
SOMA_P60520_GABARAP_12494_99	2209	1.266	1.166	1.374	2.80E-05	7.725	1077	0.784	0.665	0.924	2.423	0.0038
SOMA_Q9NRN5_OLFML3_8660_5	2209	1.22	1.138	1.308	2.84E-05	7.719	1077	1.201	1.045	1.381	1.996	0.0101

SOMA_Q8TDH9_MUTED_19116_1	2209	0.78	0.715	0.85	2.85E-05	7.718	NA	NA	NA	NA	NA	NA
SOMA_Q96QR1_SCGB3A1_6252_62	2209	1.282	1.176	1.398	2.86E-05	7.716	1077	1.450	1.340	1.570	19.370	<0.0001
SOMA_Q9Y4Z0_LSM4_16854_17	2209	0.783	0.719	0.853	2.88E-05	7.713	1077	0.772	0.646	0.923	2.349	0.0045
SOMA_Q8N6K0_TEX29_13500_9	2209	0.778	0.712	0.849	2.95E-05	7.704	NA	NA	NA	NA	NA	NA
SOMA_P17535_JUND_19602_36	2209	1.269	1.167	1.378	2.99E-05	7.698	1077	1.055	0.967	1.151	0.644	0.227
SOMA_P05090_APOD_4712_28	2209	0.782	0.717	0.852	3.03E-05	7.691	1077	0.809	0.574	1.141	0.644	0.2271
SOMA_O00755_WNT7A_4889_82	2209	0.774	0.708	0.847	3.06E-05	7.687	1077	0.573	0.324	1.016	1.247	0.0567
SOMA_P30793_GCH1_11185_145	2209	0.782	0.718	0.852	3.09E-05	7.684	1077	0.978	0.831	1.151	0.103	0.7887
SOMA_P01569_IFNA5_6210_100	2209	0.777	0.712	0.849	3.09E-05	7.683	1077	0.974	0.803	1.181	0.103	0.7881
SOMA_O95644_NFATC1_14286_2	2209	0.8	0.74	0.865	3.11E-05	7.68	1077	0.609	0.485	0.766	4.667	<0.0001
SOMA_O76050_NEURL1_13604_27	2209	0.786	0.722	0.855	3.26E-05	7.659	1077	0.549	0.212	1.423	0.663	0.2174
SOMA_P22362_CCL1_2770_51	2209	0.777	0.711	0.849	3.31E-05	7.654	1077	0.837	0.692	1.011	1.186	0.0651
SOMA_O60909_B4GALT2_9595_11	2209	0.787	0.724	0.856	3.38E-05	7.644	1077	1.015	0.884	1.165	0.081	0.8305
SOMA_Q14451_GRB7_11281_6	2209	0.779	0.713	0.85	3.38E-05	7.644	1077	0.772	0.634	0.939	2.010	0.0098
SOMA_Q15075_EEA1_14043_12	2209	0.777	0.711	0.849	3.44E-05	7.636	1077	0.615	0.471	0.802	3.470	0.0003
SOMA_O14979_HNRNPDL_10852_114	2209	1.265	1.165	1.374	3.45E-05	7.635	1077	1.069	0.927	1.233	0.445	0.3588
SOMA_Q96KG7_MEGF10_11168_3	2209	0.79	0.727	0.858	3.50E-05	7.629	1077	0.960	0.785	1.172	0.163	0.6864
SOMA_Q9BUQ8_DDX23_13946_8	2209	0.78	0.714	0.851	3.51E-05	7.628	1077	0.482	0.305	0.761	2.752	0.0018
SOMA_P36888_FLT3_3437_80	2209	0.781	0.716	0.852	3.54E-05	7.624	1077	0.748	0.582	0.960	1.650	0.0224

SOMA_P21333_FLNA_11171_25	2209	1.255	1.158	1.358	3.54E-05	7.623	1077	0.687	0.517	0.911	2.032	0.0093
SOMA_Q9NTK1_DEPP_7178_59	2209	0.781	0.716	0.852	3.62E-05	7.614	NA	NA	NA	NA	NA	NA
SOMA_O00175_CCL24_4128_27	2209	0.83	0.777	0.886	3.67E-05	7.608	1077	0.968	0.804	1.166	0.135	0.733
SOMA_Q07444_KLRC3_11571_75	2209	0.801	0.741	0.866	3.75E-05	7.598	1077	0.671	0.354	1.271	0.656	0.2206
SOMA_O43915_FIGF_14705_1	2209	1.268	1.166	1.378	3.78E-05	7.595	NA	NA	NA	NA	NA	NA
SOMA_O95156_NXPH2_9511_61	2209	0.787	0.723	0.856	3.80E-05	7.593	1077	1.065	0.971	1.169	0.736	0.1835
SOMA_Q9NTU7_CBLN4_5688_65	2209	0.778	0.712	0.85	3.82E-05	7.591	1077	0.672	0.563	0.801	4.999	<0.0001
SOMA_P01133_EGF_5509_7	2209	0.779	0.714	0.851	3.83E-05	7.59	1077	0.558	0.447	0.697	6.601	<0.0001
SOMA_P13501_CCL5_5480_49	2209	0.794	0.732	0.861	3.83E-05	7.59	1077	0.570	0.482	0.674	10.285	<0.0001
SOMA_O95631_NTN1_6649_51	2209	1.276	1.171	1.391	3.90E-05	7.582	1077	1.185	1.073	1.309	3.077	0.0008
SOMA_Q13561_DCTN2_5879_51	2209	1.279	1.173	1.394	3.95E-05	7.577	1077	1.198	1.105	1.299	4.958	<0.0001
SOMA_Q9BWV2_SPATA9_7809_22	2209	1.251	1.156	1.353	3.96E-05	7.575	1077	0.974	0.828	1.147	0.122	0.7556
SOMA_P26640_VARS_13083_18	2209	0.787	0.723	0.856	4.03E-05	7.568	NA	NA	NA	NA	NA	NA
SOMA_Q15036_SNX17_12845_18	2209	0.784	0.719	0.854	4.05E-05	7.565	1077	0.671	0.566	0.795	5.372	<0.0001
SOMA_Q9BS86_ZPBP_8635_283	2209	0.797	0.736	0.863	4.08E-05	7.562	1077	0.739	0.627	0.871	3.517	0.0003
SOMA_Q9UKW4_VAV3_9830_109	2209	0.786	0.722	0.856	4.22E-05	7.547	1077	0.476	0.381	0.594	10.228	<0.0001
SOMA_Q969J5_IL22RA2_5087_5	2209	0.785	0.72	0.855	4.25E-05	7.544	1077	0.580	0.452	0.746	4.663	<0.0001
SOMA_P21583_KITLG_9377_25	2209	0.784	0.719	0.854	4.33E-05	7.536	1077	0.976	0.812	1.174	0.098	0.7983
SOMA_Q8TDQ1_CD300LF_5623_11	2209	0.779	0.714	0.851	4.35E-05	7.535	1077	0.357	0.142	0.897	1.546	0.0285

SOMA_Q12836_ZP4_7766_25	2209	0.781	0.715	0.852	4.38E-05	7.531	1077	0.972	0.827	1.142	0.138	0.7275
SOMA_Q8NBP7_PCSK9_5231_79	2209	0.786	0.721	0.856	4.39E-05	7.531	1077	0.879	0.753	1.025	0.997	0.1006
SOMA_P58401_NRXN2_8876_51	2209	0.776	0.71	0.849	4.39E-05	7.53	1077	0.411	0.201	0.840	1.830	0.0148
SOMA_Q8IWI9_MGA_11587_5	2209	0.791	0.728	0.859	4.51E-05	7.519	1077	0.590	0.284	1.225	0.805	0.1568
SOMA_P12757_SKIL_14670_1	2209	0.784	0.72	0.855	4.55E-05	7.515	1077	0.721	0.599	0.868	3.259	0.0006
SOMA_P30990_NTS_7857_22	2209	1.265	1.164	1.375	4.59E-05	7.511	1077	1.114	1.005	1.234	1.401	0.0397
SOMA_Q96HD1_CRELD1_7628_40	2209	1.272	1.168	1.385	4.72E-05	7.499	1077	1.389	1.230	1.568	6.953	<0.0001
SOMA_P14543_NID1_3213_65	2209	1.273	1.169	1.387	4.80E-05	7.491	1077	1.490	1.338	1.659	12.490	<0.0001
SOMA_Q5VZY2_PPAPDC1_13548_53	2209	0.786	0.721	0.856	4.81E-05	7.49	NA	NA	NA	NA	NA	NA
SOMA_P00734_F2_5316_54	2209	0.785	0.72	0.855	4.84E-05	7.488	1077	0.890	0.771	1.027	0.957	0.1105
SOMA_Q9Y646_CPQ_9394_19	2209	0.792	0.729	0.86	4.90E-05	7.483	1077	0.778	0.664	0.912	2.719	0.0019
SOMA_Q2TBF2_WSCD2_6274_15	2209	1.261	1.161	1.369	4.92E-05	7.481	1077	0.935	0.805	1.088	0.414	0.3855
SOMA_P27707_DCK_9836_20	2209	0.853	0.807	0.903	4.96E-05	7.477	1077	0.863	0.664	1.122	0.566	0.2717
SOMA_P14174_MIF_8221_19	2209	1.285	1.176	1.405	5.02E-05	7.472	1077	1.058	0.928	1.207	0.399	0.3993
SOMA_Q9Y3C4_TPRKB_12417_46	2209	0.79	0.727	0.859	5.03E-05	7.472	1077	0.878	0.731	1.055	0.782	0.1651
SOMA_Q16890_TPD52L1_17710_40	2209	0.8	0.739	0.866	5.16E-05	7.46	1077	1.060	0.996	1.127	1.175	0.0669
SOMD O75462 CRLF1 C 2607 54 PASS	2209	0.834	0.782	0.89	5.17E-05	7.459	NA	NA	NA	NA	NA	NA
SOMD Q9UBD9 CRLF1 C 2607 54 PASS	2209	0.834	0.782	0.89	5.17E-05	7.459	NA	NA	NA	NA	NA	NA
SOMA_Q9Y4X1_UGT2A1_8907_11	2209	0.789	0.725	0.858	5.20E-05	7.457	1077	0.600	0.494	0.728	6.616	<0.0001

SOMA_Q8NAT1_POMGNT2_6359_50	2209	1.274	1.169	1.389	5.43E-05	7.438	1077	1.068	0.990	1.151	1.046	0.0899
SOMA_P63098_PPP3R1_15545_13	2209	1.282	1.173	1.4	5.63E-05	7.423	1077	0.953	0.823	1.105	0.281	0.5237
SOMA_Q9HD43_PTPRH_11988_24	2209	0.827	0.773	0.885	5.66E-05	7.42	1077	0.654	0.191	2.240	0.302	0.4988
SOMA_Q9UBX8_B4GALT6_10832_24	2209	0.788	0.724	0.858	5.73E-05	7.415	1077	0.829	0.696	0.986	1.465	0.0343
SOMA_P55000_SLURP1_6401_73	2209	1.27	1.166	1.384	5.85E-05	7.405	NA	NA	NA	NA	NA	NA
SOMA_Q8NAC3_IL17RC_5468_67	2209	1.263	1.162	1.373	6.04E-05	7.392	1077	0.987	0.829	1.174	0.056	0.8787
SOMA_Q08830_FGL1_5581_28	2209	1.273	1.168	1.388	6.14E-05	7.385	1077	1.531	1.368	1.713	12.927	<0.0001
SOMA_Q969H8_MYDGF_9248_36	2209	1.254	1.156	1.36	6.51E-05	7.359	1077	0.973	0.814	1.164	0.116	0.7655
SOMA_Q99519_NEU1_15426_5	2209	0.796	0.734	0.864	6.77E-05	7.343	1077	0.320	0.148	0.690	2.440	0.0036
SOMA_Q8IW41_MAPKAPK_8382_47	2209	0.795	0.732	0.863	6.95E-05	7.331	1077	0.674	0.556	0.816	4.260	<0.0001
SOMA_P47874_OMP_18240_6	2209	0.841	0.79	0.895	7.04E-05	7.326	1077	1.059	0.996	1.126	1.166	0.0682
SOMA_Q96FJ2_DYNLL2_11493_169	2209	1.252	1.155	1.358	7.10E-05	7.321	1077	0.857	0.734	1.000	1.296	0.0506
SOMA_Q9UBS4_DNAJB11_7110_2	2209	1.26	1.16	1.369	7.16E-05	7.318	1077	1.053	0.910	1.218	0.312	0.4879
SOMA_Q9P0B6_CCDC167_7797_11	2209	0.789	0.724	0.859	7.62E-05	7.291	1077	1.043	0.954	1.141	0.454	0.3519
SOMA_P02810_PRH1_10502_15	2209	0.825	0.769	0.884	7.82E-05	7.279	NA	NA	NA	NA	NA	NA
SOMA_P07951_TPM2_4472_5	2209	0.78	0.713	0.853	7.84E-05	7.279	1077	0.816	0.700	0.950	2.049	0.0089
SOMA_Q9Y328_NSG2_13409_9	2209	0.787	0.722	0.858	8.10E-05	7.264	NA	NA	NA	NA	NA	NA
SOMA_P01906_HLA_DQA_7757_5	2209	1.263	1.161	1.374	8.19E-05	7.259	1077	1.015	0.892	1.155	0.088	0.8163
SOMA_P55735_SEC13_14689_3	2209	0.782	0.715	0.854	8.20E-05	7.259	1077	0.711	0.599	0.844	4.015	<0.0001

SOMA_Q6P5S2_LEG1_7154_92	2209	0.791	0.727	0.861	8.40E-05	7.248	1077	0.648	0.533	0.787	4.900	<0.0001
SOMA_Q03426_MVK_11391_69	2209	0.817	0.76	0.879	8.46E-05	7.246	1077	0.493	0.239	1.018	1.253	0.0559
SOMA_Q8N428_GALNT16_8923_94	2209	1.25	1.153	1.355	8.61E-05	7.238	1077	1.140	1.025	1.269	1.793	0.0161
SOMA_Q9UKY0_PRND_18231_147	2209	0.797	0.735	0.865	8.71E-05	7.233	1077	0.065	0.019	0.227	4.745	<0.0001
SOMA_Q99523_SORT1_11300_32	2209	0.78	0.713	0.853	8.91E-05	7.223	1077	1.000	0.868	1.151	0.002	0.9956
SOMA_Q8N475_FSTL5_7099_33	2209	0.797	0.734	0.865	8.93E-05	7.222	1077	0.991	0.841	1.166	0.041	0.9093
SOMA_Q15080_NCF4_14060_67	2209	0.798	0.736	0.866	8.95E-05	7.221	1077	0.832	0.571	1.212	0.472	0.3373
SOMA_Q8N695_SLC5A8_13691_10	2209	0.792	0.727	0.862	9.61E-05	7.19	1077	0.871	0.746	1.017	1.094	0.0805
SOMA_Q9C098_DCLK3_7826_1	2209	0.782	0.715	0.855	0.000101	7.17	1077	0.850	0.509	1.419	0.272	0.5348
SOMA_Q8NCW6_GALNT11_8700_325	2209	0.781	0.714	0.854	0.000101	7.167	1077	0.733	0.509	1.055	1.023	0.0947
SOMA_Q99418_CYTH2_12533_135	2209	0.798	0.735	0.866	0.000102	7.166	1077	1.119	0.999	1.253	1.279	0.0526
SOMA_Q9NZ45_CISD1_7745_3	2209	1.258	1.157	1.367	0.000102	7.166	1077	1.011	0.897	1.140	0.068	0.8553
SOMA_Q8N729_NPW_9986_14	2209	1.262	1.16	1.373	0.000104	7.157	1077	0.982	0.845	1.141	0.090	0.813
SOMA_O15146_MUSK_11547_84	2209	0.791	0.726	0.861	0.000105	7.153	1077	0.574	0.346	0.952	1.502	0.0315
SOMA_Q9H7X2_C1orf11_8366_19	2209	0.778	0.71	0.853	0.000105	7.153	1077	0.997	0.859	1.157	0.014	0.9691
SOMA_Q9UJU6_DBNL_4978_54	2209	0.793	0.728	0.862	0.000106	7.149	1077	0.781	0.645	0.946	1.939	0.0115
SOMA_Q9H3M7_TXNIP_11682_7	2209	0.785	0.719	0.857	0.000106	7.148	1077	0.158	0.069	0.364	4.847	<0.0001
SOMA_Q06609_RAD51_2871_73	2209	0.785	0.719	0.857	0.000108	7.141	1077	0.827	0.661	1.033	1.027	0.094
SOMA_Q8N0W4_NLGN4X_5357_60	2209	1.249	1.152	1.354	0.000109	7.136	1077	1.012	0.893	1.146	0.069	0.8527

SOMA_Q92851_CASP10_5340_24	2209	0.787	0.722	0.859	0.000109	7.136	1077	0.946	0.725	1.234	0.167	0.6815
SOMA_Q6IA17_SIGIRR_8326_63	2209	0.79	0.726	0.861	0.000109	7.135	1077	0.735	0.501	1.079	0.935	0.1161
SOMA_P02760_AMBP_15453_3	2209	1.259	1.158	1.369	0.000109	7.134	1077	1.024	0.884	1.185	0.123	0.7534
SOMA_Q01581_HMGCS1_13496_19	2209	0.784	0.718	0.857	0.000112	7.125	1077	1.089	0.953	1.244	0.678	0.2099
SOMA_Q7Z7B8_DEFB128_6360_7	2209	0.796	0.732	0.865	0.000112	7.125	1077	0.709	0.376	1.338	0.539	0.2887
SOMA_Q15375_EPHA7_15580_2	2209	1.263	1.16	1.375	0.000112	7.122	1077	1.022	0.915	1.142	0.154	0.7017
SOMA_Q6MZM9_PRR27_9607_39	2209	0.791	0.726	0.861	0.000113	7.119	1077	0.648	0.473	0.887	2.175	0.0067
SOMA_P43403_ZAP70_4476_22	2209	0.784	0.717	0.857	0.000116	7.107	1077	0.838	0.721	0.974	1.667	0.0215
SOMA_Q96E11_MRRF_12355_223	2209	0.81	0.75	0.875	0.000119	7.098	1077	0.913	0.691	1.207	0.280	0.5243
SOMA_O14867_BACH1_12451_62	2209	0.789	0.723	0.86	0.00012	7.093	1077	0.793	0.666	0.945	2.031	0.0093
SOMA_Q92922_SMARCC1_11180_17	2209	0.782	0.715	0.856	0.00012	7.093	1077	0.913	0.722	1.155	0.349	0.4477
SOMA_Q92918_MAP4K1_8954_30	2209	1.259	1.157	1.369	0.000122	7.087	1077	0.885	0.684	1.147	0.449	0.3558
SOMA_Q6UWM7_LCTL_10890_135	2209	0.845	0.795	0.899	0.000122	7.086	1077	1.004	0.877	1.149	0.018	0.9593
SOMA_P35225_IL13_3072_4	2209	0.78	0.713	0.854	0.000124	7.079	1077	0.286	0.080	1.019	1.272	0.0535
SOMA_P99999_CYCS_2942_50	2209	0.809	0.749	0.874	0.000125	7.076	1077	0.527	0.372	0.746	3.516	0.0003
SOMA_Q8N690_DEFB119_8315_5	2209	0.784	0.718	0.857	0.000126	7.073	1077	1.023	0.901	1.160	0.138	0.7274
SOMA_Q9Y5F6_PCDHGC5_7983_1	2209	0.783	0.715	0.856	0.000128	7.067	1077	0.246	0.116	0.523	3.582	0.0003
SOMA_O00399_DCTN6_18311_44	2209	1.266	1.161	1.38	0.000129	7.062	1077	0.902	0.775	1.051	0.731	0.1856
SOMA_Q6PCB0_VWA1_6385_63	2209	1.263	1.159	1.376	0.000129	7.062	1077	1.132	1.067	1.202	4.394	<0.0001

SOMA_Q5T6X4_FAM162B_11163_7	2209	0.783	0.716	0.856	0.00013	7.059	1077	1.040	0.944	1.146	0.369	0.4273
SOMA_Q495A1_TIGIT_16299_13	2209	0.799	0.736	0.868	0.00013	7.057	1077	0.484	0.268	0.872	1.802	0.0158
SOMA_O60869_EDF1_12415_122	2209	0.787	0.721	0.859	0.000131	7.054	1077	0.587	0.433	0.795	3.227	0.0006
SOMA_Q16557_PSG3_6444_15	2209	1.257	1.156	1.367	0.000132	7.051	1077	0.992	0.847	1.163	0.034	0.9251
SOMA_Q6ZUB0_SPATA31_9524_46	2209	0.787	0.721	0.859	0.000133	7.05	1077	0.228	0.008	6.282	0.417	0.3826
SOMA_P02775_PPBP_16765_52	2209	0.806	0.745	0.873	0.000134	7.044	1077	0.605	0.516	0.708	9.401	<0.0001
SOMA_Q99728_BARD1_13977_28	2209	0.78	0.712	0.854	0.000135	7.043	1077	0.000	0.000	0.236	1.675	0.0211
SOMA_Q16549_PCSK7_4459_68	2209	0.788	0.722	0.86	0.000136	7.04	1077	0.933	0.800	1.087	0.430	0.3719
SOMA_Q14005_IL16_2774_10	2209	1.256	1.155	1.366	0.000136	7.039	1077	1.241	1.109	1.389	3.778	0.0002
SOMA_P20138_CD33_3166_92	2209	1.271	1.164	1.388	0.000137	7.037	1077	1.342	1.180	1.526	5.115	<0.0001
SOMA_Q50LG9_LRRC24_9989_12	2209	0.79	0.724	0.861	0.000138	7.034	1077	0.894	0.595	1.344	0.229	0.5905
SOMA_Q6NUJ1_PSAPL1_8814_33	2209	0.791	0.726	0.862	0.000141	7.025	1077	0.559	0.437	0.717	5.376	<0.0001
SOMA_P50895_BCAM_2816_50	2209	1.245	1.149	1.349	0.000141	7.024	1077	1.067	0.998	1.140	1.244	0.0571
SOMA_P08493_MGP_6520_87	2209	1.248	1.151	1.354	0.000141	7.022	1077	1.125	0.992	1.277	1.172	0.0673
SOMA_Q9BV47_DUSP26_8967_6	2209	0.798	0.734	0.867	0.000142	7.021	1077	0.936	0.762	1.151	0.275	0.531
SOMA_P22466_GAL_15390_3	2209	0.793	0.728	0.863	0.000144	7.016	1077	0.741	0.587	0.935	1.941	0.0115
SOMA_Q13946_PDE7A_5178_5	2209	0.846	0.796	0.9	0.000145	7.013	1077	0.889	0.714	1.107	0.533	0.293
SOMA_Q15181_PPA1_5021_13	2209	0.791	0.726	0.862	0.000145	7.013	1077	0.852	0.702	1.035	0.975	0.106
SOMA_Q9H3H3_C11orf6_8307_47	2209	1.245	1.148	1.349	0.000146	7.01	1077	0.802	0.694	0.927	2.548	0.0028

SOMA_Q8TD07_RAET1E_7800_85	2209	0.789	0.723	0.861	0.000148	7.003	NA	NA	NA	NA	NA	NA
SOMA_O15217_GSTA4_14645_253	2209	0.795	0.73	0.865	0.000148	7.002	1077	0.836	0.665	1.051	0.906	0.1241
SOMA_Q96NW4_ANKRD27_12445_50	2209	0.798	0.735	0.867	0.00015	6.996	1077	1.050	0.954	1.156	0.502	0.3149
SOMA_Q9NWM8_FKBP14_9340_17	2209	1.252	1.152	1.36	0.00015	6.996	1077	1.016	0.902	1.145	0.102	0.7902
SOMA_Q9HAW9_UGT1A8_8899_75	2209	0.793	0.728	0.863	0.000151	6.995	1077	0.975	0.816	1.165	0.108	0.779
SOMA_P00325_ADH1B_9834_62	2209	0.785	0.719	0.859	0.000151	6.993	1077	1.041	0.916	1.184	0.269	0.538
SOMA_Q8NDV2_GPR26_13540_1	2209	0.788	0.722	0.86	0.000152	6.991	1077	0.948	0.767	1.171	0.207	0.6209
SOMA_Q4LDR2_CTXN3_10467_58	2209	0.842	0.791	0.897	0.000153	6.988	NA	NA	NA	NA	NA	NA
SOMA_P51668_UBE2D1_19247_1	2209	0.787	0.721	0.86	0.000154	6.984	1077	0.975	0.799	1.188	0.097	0.7994
SOMA_Q7Z434_MAVS_8783_216	2209	1.263	1.159	1.377	0.000156	6.98	NA	NA	NA	NA	NA	NA
SOMA_Q9Y2I2_NTNG1_5637_81	2209	1.262	1.158	1.375	0.000158	6.974	1077	1.094	1.018	1.175	1.824	0.015
SOMA_Q05315_CLC_11094_104	2209	0.846	0.795	0.9	0.000163	6.961	1077	0.117	0.006	2.499	0.770	0.1698
SOMA_Q53S33_BOLA3_5980_55	2209	1.244	1.147	1.348	0.00017	6.942	1077	0.948	0.818	1.097	0.326	0.4719
SOMA_O60880_SH2D1A_4567_82	2209	1.248	1.15	1.354	0.000171	6.941	1077	1.024	0.894	1.173	0.137	0.7291
SOMA_Q96C36_PYCR2_18328_36	2209	0.797	0.733	0.867	0.000176	6.927	1077	0.498	0.264	0.937	1.512	0.0307
SOMA_P49863_GZMK_9545_156	2209	0.793	0.728	0.864	0.000177	6.926	1077	0.899	0.549	1.471	0.173	0.6715
SOMA_Q6UXZ4_UNC5D_16307_22	2209	1.259	1.156	1.371	0.000178	6.923	1077	1.202	1.093	1.322	3.805	0.0002
SOMA_Q9UHP3_USP25_9215_117	2209	0.786	0.719	0.86	0.00018	6.917	1077	0.608	0.286	1.294	0.706	0.1967
SOMA_P31371_FGF9_19584_33	2209	0.791	0.726	0.863	0.000184	6.907	1077	0.710	0.463	1.087	0.939	0.115

SOMA_P46109_CRKL_9877_28	2209	1.245	1.148	1.35	0.000186	6.904	1077	0.745	0.626	0.886	3.067	0.0009
SOMA_Q5SRI9_MANEA_8014_359	2209	1.225	1.136	1.32	0.000187	6.902	1077	1.181	1.078	1.295	3.415	0.0004
SOMA_Q13822_ENPP2_16892_23	2209	1.274	1.165	1.394	0.000188	6.899	1077	1.272	1.154	1.402	5.937	<0.0001
SOMA_O00592_PODXL_8792_17	2209	0.788	0.722	0.861	0.000189	6.897	NA	NA	NA	NA	NA	NA
SOMA_Q9BXP8_PAPPA2_5756_66	2209	0.793	0.727	0.864	0.000195	6.882	1077	0.943	0.767	1.160	0.237	0.5799
SOMA_Q96P47_AGAP3_13960_15	2209	0.792	0.726	0.863	0.000196	6.88	1077	1.065	0.966	1.174	0.687	0.2054
SOMA_P29350_PTPN6_4318_12	2209	1.273	1.164	1.393	0.000198	6.876	1077	0.954	0.819	1.112	0.260	0.5489
SOMA_P51451_BLK_11338_49	2209	0.792	0.726	0.864	0.000199	6.873	1077	0.835	0.616	1.131	0.612	0.2445
SOMA_Q7LG56_RRM2B_8925_25	2209	1.248	1.149	1.355	0.000202	6.868	1077	0.915	0.786	1.064	0.603	0.2492
SOMA_Q8IZF2_ADGRF5_6409_57	2209	0.802	0.739	0.871	0.000203	6.865	1077	0.892	0.616	1.292	0.264	0.5442
SOMA_Q92519_TRIB2_12363_70	2209	0.856	0.808	0.907	0.000205	6.86	1077	0.970	0.796	1.183	0.115	0.7671
SOMA_Q15084_PDIA6_5650_9	2209	1.243	1.146	1.348	0.000209	6.853	1077	0.905	0.779	1.051	0.717	0.1917
SOMA_P55199_ELL_11459_81	2209	0.795	0.73	0.866	0.000212	6.847	1077	0.906	0.757	1.084	0.551	0.2812
SOMA_P16870_CPE_5343_74	2209	0.805	0.743	0.873	0.000214	6.843	1077	1.111	1.048	1.178	3.351	0.0004
SOMA_Q8WUJ1_CYB5D2_10924_258	2209	1.249	1.149	1.356	0.000214	6.842	1077	0.940	0.718	1.231	0.185	0.6538
SOMA_Q9UBQ6_EXTL2_6528_95	2209	0.798	0.733	0.868	0.000214	6.842	1077	0.071	0.005	1.060	1.258	0.0552
SOMA_Q9Y4P9_SPEF1_12876_39	2209	0.79	0.723	0.862	0.000219	6.833	1077	0.780	0.404	1.504	0.339	0.4577
SOMA_Q07817_BCL2L1_4423_77	2209	0.791	0.724	0.863	0.00022	6.831	1077	0.836	0.719	0.972	1.696	0.0201
SOMA_Q8NCW5_APOA1BP_16621_77	2209	1.258	1.155	1.37	0.000223	6.825	NA	NA	NA	NA	NA	NA

SOMA_Q8NDI1_EHBP1_12813_18	2209	0.799	0.734	0.868	0.000223	6.825	1077	0.574	0.362	0.909	1.746	0.0179
SOMA_Q7Z7B7_DEFB132_9022_49	2209	0.791	0.725	0.864	0.00023	6.811	1077	0.738	0.599	0.908	2.387	0.0041
SOMA_P61587_RND3_15439_21	2209	0.809	0.747	0.876	0.000231	6.808	1077	0.952	0.759	1.194	0.174	0.6694
SOMA_P84101_SERF2_18274_2	2209	0.794	0.728	0.865	0.000232	6.807	1077	0.910	0.647	1.281	0.229	0.5904
SOMA_Q8N4C9_C17orf7_6571_75	2209	0.789	0.722	0.862	0.000232	6.807	1077	0.558	0.353	0.883	1.893	0.0128
SOMA_P02655_APOC2_6350_43	2209	1.248	1.149	1.356	0.000234	6.803	1077	0.976	0.792	1.202	0.087	0.8181
SOMA_P08133_ANXA6_5335_73	2209	0.801	0.738	0.87	0.000238	6.797	1077	0.628	0.536	0.736	8.041	<0.0001
SOMA_P61960_UFM1_3836_51	2209	1.238	1.143	1.341	0.000239	6.794	1077	0.835	0.684	1.020	1.107	0.0781
SOMA_Q15637_SF1_12777_11	2209	1.249	1.149	1.358	0.000241	6.791	1077	0.995	0.857	1.156	0.022	0.951
SOMA_P62745_RHOB_18166_4	2209	1.258	1.155	1.372	0.000249	6.776	1077	1.053	0.956	1.160	0.530	0.295
SOMA_Q9NT99_LRRC4B_11911_13	2209	0.852	0.802	0.905	0.00025	6.774	1077	1.048	0.955	1.151	0.492	0.3222
SOMA_P01286_GHRH_9487_60	2209	0.791	0.724	0.863	0.000256	6.765	1077	0.721	0.433	1.200	0.681	0.2083
SOMA_O00451_GFRA2_2515_14	2209	1.258	1.154	1.371	0.000256	6.764	1077	1.391	1.259	1.538	10.053	<0.0001
SOMA_Q9P2J2_IGSF9_10518_14	2209	0.849	0.798	0.903	0.000259	6.76	NA	NA	NA	NA	NA	NA
SOMA_P40225_THPO_5947_90	2209	1.259	1.155	1.373	0.00026	6.758	1077	1.127	1.025	1.238	1.876	0.0133
SOMA_O75351_VPS4B_12668_7	2209	0.796	0.731	0.867	0.000263	6.753	1077	0.805	0.687	0.943	2.143	0.0072
SOMA_P02649_APOE_2418_55	2209	0.8	0.736	0.87	0.000264	6.751	1077	0.620	0.503	0.763	5.159	<0.0001
SOMA_P20807_CAPN3_12385_4	2209	0.795	0.73	0.867	0.000267	6.746	1077	0.520	0.286	0.945	1.495	0.032
SOMA_Q96FT7_ASIC4_6951_26	2209	0.793	0.727	0.865	0.000272	6.738	1077	1.062	0.962	1.172	0.626	0.2364

SOMA_O95971_CD160_17677_47	2209	1.257	1.153	1.37	0.000279	6.727	1077	1.084	1.009	1.164	1.552	0.0281
SOMA_Q7Z2E3_APTX_17736_105	2209	0.811	0.749	0.877	0.000296	6.702	1077	0.722	0.512	1.018	1.200	0.0631
SOMA_Q15399_TLR1_16324_38	2209	0.793	0.727	0.865	0.000297	6.7	1077	0.652	0.429	0.992	1.339	0.0459
SOMA_Q86XE5_HOGA1_10024_44	2209	0.809	0.747	0.876	0.000303	6.691	1077	0.930	0.708	1.221	0.222	0.6004
SOMA_Q9NPH9_IL26_16760_2	2209	0.797	0.731	0.868	0.000306	6.688	1077	0.379	0.252	0.571	5.447	<0.0001
SOMA_P35609_ACTN2_9844_138	2209	1.25	1.149	1.361	0.000307	6.686	1077	1.218	1.114	1.331	4.871	<0.0001
SOMA_P61647_ST8SIA6_6930_95	2209	0.794	0.727	0.866	0.000312	6.679	1077	0.882	0.558	1.394	0.229	0.5908
SOMA_P52566_ARHGDIB_9846_32	2209	1.238	1.142	1.342	0.000319	6.67	1077	0.820	0.713	0.942	2.286	0.0052
SOMA_Q13442_PDAP1_19240_265	2209	1.246	1.146	1.353	0.000319	6.67	1077	0.825	0.709	0.960	1.884	0.0131
SOMA_Q9UNA0_ADAMTS5_3168_8	2209	1.242	1.144	1.348	0.000329	6.655	1077	1.049	0.966	1.138	0.589	0.2579
SOMA_Q6EBC2_IL31_10455_196	2209	0.8	0.735	0.87	0.000331	6.653	1077	0.913	0.693	1.203	0.286	0.5174
SOMA_Q01459_CTBS_6115_40	2209	1.254	1.151	1.366	0.000333	6.651	NA	NA	NA	NA	NA	NA
SOMA_P15328_FOLR1_17455_42	2209	1.249	1.148	1.359	0.000335	6.648	1077	1.163	1.081	1.252	4.277	<0.0001
SOMA_P17655_CAPN2_14684_17	2209	1.258	1.153	1.373	0.000339	6.642	1077	1.106	1.022	1.197	1.915	0.0121
SOMA_O60506_SYNCRIP_4224_7	2209	0.798	0.732	0.869	0.00035	6.629	1077	0.790	0.676	0.923	2.525	0.003
SOMA_P50406_HTR6_13561_5	2209	0.801	0.736	0.871	0.000351	6.628	1077	1.004	0.857	1.177	0.019	0.9576
SOMA_Q4V9L6_TMEM119_11110_4	2209	0.791	0.724	0.865	0.000352	6.626	1077	0.557	0.375	0.826	2.439	0.0036
SOMA_P43220_GLP1R_13085_18	2209	0.853	0.804	0.906	0.00036	6.616	1077	0.423	0.054	3.346	0.382	0.4152
SOMA_Q86SJ2_AMIGO2_14134_49	2209	1.249	1.148	1.359	0.000362	6.614	1077	1.023	0.926	1.129	0.182	0.6572

SOMA_P42892_ECE1_8767_44	2209	0.792	0.725	0.866	0.000363	6.612	1077	0.816	0.624	1.068	0.856	0.1392
SOMA_Q86WA9_SLC26A1_13502_2	2209	0.8	0.735	0.871	0.000378	6.596	1077	1.049	0.963	1.143	0.561	0.2749
SOMA_Q9Y663_HS3ST3A_8268_98	2209	1.251	1.149	1.362	0.000379	6.595	1077	0.910	0.781	1.061	0.643	0.2274
SOMA_Q92870_APBB2_12761_12	2209	0.834	0.778	0.893	0.000381	6.592	1077	0.878	0.563	1.370	0.246	0.5671
SOMA_Q13642_FHL1_16814_13	2209	0.84	0.786	0.897	0.000385	6.588	1077	0.865	0.597	1.254	0.352	0.4447
SOMA_Q86WC4_OSTM1_19353_25	2209	0.864	0.818	0.914	0.000384	6.588	1077	0.876	0.702	1.093	0.619	0.2404
SOMA_Q9H2E6_SEMA6A_7945_10	2209	1.252	1.149	1.363	0.000394	6.578	1077	1.422	1.269	1.593	8.891	<0.0001
SOMA_Q9UK80_USP21_12681_63	2209	0.816	0.755	0.881	0.000394	6.577	1077	0.576	0.339	0.978	1.388	0.0409
SOMA_P48736_PIK3CG_3391_10	2209	0.788	0.72	0.863	0.000397	6.574	1077	0.806	0.692	0.939	2.246	0.0057
SOMA_P18627_LAG3_9950_229	2209	1.25	1.148	1.362	0.000402	6.568	1077	1.128	1.009	1.261	1.456	0.035
SOMA_P61964_WDR5_17760_128	2209	1.254	1.15	1.366	0.000402	6.568	1077	1.003	0.867	1.162	0.016	0.9633
SOMA_P31997_CEACAM8_18873_8	2209	1.247	1.146	1.356	0.000404	6.567	1077	1.144	1.086	1.205	6.360	<0.0001
SOMA_Q16401_PSMD5_10716_35	2209	0.838	0.784	0.897	0.00041	6.56	1077	0.906	0.712	1.154	0.372	0.4245
SOMA_A1L170_C1orf22_7989_5	2209	1.248	1.147	1.359	0.000416	6.554	1077	1.141	1.039	1.252	2.252	0.0056
SOMA_Q9Y2T1_AXIN2_12925_105	2209	0.8	0.734	0.871	0.000418	6.552	1077	0.258	0.091	0.732	1.964	0.0109
SOMA_P09603_CSF1_3738_54	2209	1.24	1.142	1.346	0.000422	6.547	1077	1.069	1.003	1.140	1.394	0.0403
SOMA_Q9Y5E5_PCDHB4_10522_167	2209	0.795	0.728	0.868	0.000428	6.541	NA	NA	NA	NA	NA	NA
SOMA_P27037_ACVR2A_19208_8	2209	0.793	0.726	0.866	0.000429	6.54	1077	0.499	0.250	0.994	1.318	0.0481
SOMA_O96013_PAK4_13719_19	2209	1.236	1.14	1.341	0.000432	6.538	1077	0.745	0.644	0.863	4.087	<0.0001

SOMA_Q06190_PPP2R3A_13665_35	2209	0.806	0.743	0.876	0.000431	6.538	1077	0.152	0.057	0.404	3.807	0.0002
SOMA_O14807_MRAS_18297_8	2209	0.798	0.732	0.87	0.000443	6.527	1077	0.816	0.591	1.126	0.666	0.2158
SOMA_O14896_IRF6_9999_1	2209	1.239	1.141	1.345	0.000447	6.523	1077	0.810	0.673	0.974	1.596	0.0253
SOMA_P07093_SERPINE_19154_41	2209	0.801	0.736	0.872	0.000466	6.505	1077	0.630	0.523	0.760	5.861	<0.0001
SOMA_O15205_UBD_17772_7	2209	0.795	0.728	0.868	0.00047	6.501	1077	0.700	0.463	1.058	1.042	0.0908
SOMA_P01019_AGT_3484_60	2209	0.796	0.729	0.869	0.000472	6.498	1077	0.685	0.576	0.815	4.707	<0.0001
SOMA_Q92876_KLK6_3450_4	2209	0.795	0.729	0.868	0.000473	6.498	1077	0.858	0.723	1.019	1.088	0.0816
SOMA_P0C8F1_PATE4_8065_245	2209	0.8	0.734	0.871	0.000475	6.496	1077	0.703	0.456	1.084	0.955	0.1108
SOMA_Q6GPH6_ITPRIPL_9221_6	2209	1.229	1.135	1.329	0.000476	6.495	1077	0.913	0.783	1.064	0.612	0.2443
SOMA_Q9NZ94_NLGN3_17427_26	2209	0.837	0.782	0.896	0.000488	6.484	1077	0.125	0.016	0.983	1.317	0.0481
SOMA_P61106_RAB14_14283_12	2209	1.244	1.144	1.353	0.000498	6.476	1077	0.996	0.859	1.155	0.018	0.9598
SOMA_Q96EC8_YIPF6_9984_12	2209	0.798	0.731	0.87	0.000499	6.475	1077	0.914	0.712	1.175	0.315	0.4837
SOMA_O95866_G6B_8659_68	2209	0.79	0.722	0.865	0.000514	6.462	NA	NA	NA	NA	NA	NA
SOMA_Q9H3M0_KCNF1_12834_3	2209	0.802	0.736	0.873	0.000515	6.461	1077	0.723	0.532	0.982	1.423	0.0377
SOMA_Q9UMX1_SUFU_17468_1	2209	0.83	0.772	0.891	0.000519	6.458	1077	0.981	0.816	1.178	0.078	0.8362
SOMA_O60476_MAN1A2_9077_10	2209	0.804	0.739	0.874	0.000524	6.454	1077	1.026	0.888	1.185	0.138	0.727
SOMA_O14598_VCY_6295_67	2209	0.808	0.744	0.877	0.000539	6.441	NA	NA	NA	NA	NA	NA
SOMA_P02753_RBP4_15633_6	2209	1.246	1.145	1.356	0.000542	6.439	1077	1.083	0.940	1.249	0.568	0.2704
SOMA_P08294_SOD3_5660_51	2209	1.247	1.146	1.358	0.000545	6.437	1077	1.106	1.007	1.214	1.461	0.0346

SOMA_P0CG47_UBB_6651_74	2209	1.245	1.144	1.355	0.000551	6.432	1077	0.990	0.856	1.145	0.049	0.8923
SOMA_P45452_MMP13_4925_54	2209	1.242	1.142	1.35	0.000553	6.43	1077	1.211	1.140	1.287	9.301	<0.0001
SOMA_P47972_NPTX2_6521_35	2209	0.805	0.741	0.875	0.000557	6.427	1077	0.994	0.856	1.153	0.030	0.9337
SOMA_P01189_POMC_9204_33	2209	1.249	1.146	1.36	0.00056	6.425	1077	1.062	0.974	1.157	0.767	0.171
SOMA_O14618_CCS_13068_139	2209	0.806	0.742	0.876	0.00056	6.424	1077	0.886	0.604	1.299	0.272	0.5351
SOMA_P31943_HNRNPH1_14309_8	2209	0.809	0.745	0.878	0.000571	6.416	1077	1.030	0.923	1.149	0.221	0.6006
SOMA_Q9BQE4_VIMP_11286_78	2209	0.803	0.738	0.874	0.000591	6.401	NA	NA	NA	NA	NA	NA
SOMA_Q01826_SATB1_13511_29	2209	0.805	0.74	0.875	0.000595	6.398	1077	0.612	0.318	1.175	0.854	0.1399
SOMA_Q9BXJ7_AMN_4322_28	2209	0.845	0.792	0.902	0.000601	6.394	1077	0.979	0.812	1.180	0.085	0.823
SOMA_Q6ZVN8_HFE2_3332_57	2209	0.796	0.729	0.87	0.000604	6.392	NA	NA	NA	NA	NA	NA
SOMA_Q6IE38_SPINK14_8587_21	2209	1.253	1.148	1.368	0.00061	6.388	1077	1.058	0.931	1.203	0.416	0.3835
SOMA_P27144_AK4_17432_25	2209	0.812	0.749	0.88	0.000611	6.387	1077	0.719	0.560	0.923	2.021	0.0095
SOMA_P24592_IGFBP6_14088_38	2209	1.244	1.143	1.354	0.000615	6.384	1077	1.352	1.197	1.527	5.905	<0.0001
SOMD P24863 CDK8 CC 3359 11 PASS	2209	0.797	0.73	0.87	0.000632	6.372	NA	NA	NA	NA	NA	NA
SOMD P49336 CDK8 CC 3359 11 PASS	2209	0.797	0.73	0.87	0.000632	6.372	NA	NA	NA	NA	NA	NA
SOMA_Q9Y6N9_USH1C_17725_37	2209	0.854	0.803	0.908	0.000643	6.365	1077	0.485	0.288	0.816	2.195	0.0064
SOMA_P15248_IL9_5834_18	2209	1.249	1.146	1.361	0.000646	6.363	1077	1.053	0.976	1.136	0.731	0.1858
SOMA_P42704_LRPPRC_9046_46	2209	0.803	0.737	0.874	0.000659	6.354	NA	NA	NA	NA	NA	NA
SOMA_Q14118_DAG1_8369_102	2209	1.237	1.139	1.343	0.000659	6.354	1077	1.028	0.921	1.148	0.204	0.6252

SOMA_Q96G74_OTUD5_12480_9	2209	0.797	0.73	0.871	0.000671	6.346	1077	0.780	0.662	0.920	2.507	0.0031
SOMA_Q9BQG1_SYT3_10452_24	2209	0.797	0.729	0.87	0.000673	6.345	NA	NA	NA	NA	NA	NA
SOMA_O75771_RAD51L3_12554_10	2209	0.806	0.741	0.877	0.00068	6.34	NA	NA	NA	NA	NA	NA
SOMA_P08185_SERPINA_4785_30	2209	0.843	0.788	0.901	0.000682	6.339	1077	0.000	0.000	0.004	2.653	0.0022
SOMA_P16403_HIST1H1_2987_37	2209	1.226	1.133	1.328	0.000684	6.338	NA	NA	NA	NA	NA	NA
SOMA_Q96CS3_FAF2_9738_7	2209	1.224	1.131	1.324	0.000701	6.327	1077	0.719	0.617	0.838	4.625	<0.0001
SOMA_Q13651_IL10RA_10344_334	2209	0.821	0.76	0.886	0.000703	6.326	1077	0.714	0.533	0.956	1.629	0.0235
SOMA_Q03167_TGFBR3_3009_3	2209	1.254	1.148	1.37	0.000713	6.32	1077	1.429	1.286	1.587	10.500	<0.0001
SOMA_O75096_LRP4_19558_10	2209	1.241	1.141	1.35	0.000714	6.319	1077	1.152	1.060	1.253	3.060	0.0009
SOMA_Q8NHP8_PLBD2_6536_54	2209	0.802	0.736	0.874	0.000718	6.316	1077	0.572	0.409	0.798	2.984	0.001
SOMA_O00189_AP4M1_10076_1	2209	0.798	0.73	0.871	0.000726	6.312	1077	0.592	0.458	0.765	4.216	<0.0001
SOMA_P05113_IL5_11071_1	2209	0.856	0.806	0.909	0.000727	6.311	1077	0.414	0.212	0.805	2.026	0.0094
SOMA_P01210_PENK_9076_25	2209	1.242	1.141	1.351	0.000734	6.307	1077	1.075	0.942	1.228	0.547	0.2837
SOMA_P05408_SCG5_15358_28	2209	0.797	0.73	0.871	0.000734	6.307	1077	0.864	0.739	1.009	1.187	0.065
SOMA_Q8NC54_KCT2_6365_62	2209	0.795	0.726	0.869	0.000739	6.304	NA	NA	NA	NA	NA	NA
SOMA_Q9BTV7_CABLES2_7105_7	2209	0.8	0.733	0.872	0.000739	6.304	1077	0.474	0.216	1.037	1.210	0.0617
SOMA_Q8IWT1_SCN4B_7911_29	2209	0.805	0.74	0.876	0.000749	6.299	1077	0.575	0.324	1.020	1.233	0.0584
SOMA_Q9UKA2_FBXL4_11416_23	2209	0.808	0.744	0.878	0.000753	6.296	1077	1.059	0.962	1.166	0.620	0.2399
SOMA_Q9NQ38_SPINK5_8028_22	2209	1.235	1.137	1.34	0.000756	6.294	1077	1.083	1.027	1.142	2.476	0.0033

SOMA_Q96JN8_NEURL4_13468_5	2209	0.801	0.735	0.874	0.000772	6.285	1077	0.845	0.394	1.815	0.176	0.6663
SOMA_Q92620_DHX38_13645_14	2209	0.799	0.732	0.872	0.000776	6.283	1077	0.481	0.265	0.876	1.779	0.0166
SOMA_Q96DB5_RMDN1_7096_30	2209	1.228	1.133	1.33	0.000788	6.276	1077	0.943	0.802	1.109	0.322	0.4769
SOMA_A2RU67_KIAA146_7892_132	2209	0.794	0.725	0.869	0.000804	6.268	NA	NA	NA	NA	NA	NA
SOMA_P04114_APOB_2797_56	2209	0.8	0.733	0.873	0.000802	6.268	1077	1.010	0.872	1.171	0.050	0.8922
SOMA_Q9UIW2_PLXNA1_9005_16	2209	1.225	1.132	1.326	0.000806	6.266	1077	1.107	1.035	1.183	2.545	0.0029
SOMA_Q15762_CD226_5062_60	2209	1.238	1.139	1.345	0.000808	6.265	1077	1.178	1.099	1.263	5.393	<0.0001
SOMA_Q9NZU1_FLRT1_4547_59	2209	0.842	0.788	0.901	0.000809	6.265	1077	0.724	0.608	0.864	3.485	0.0003
SOMA_Q9BY67_CADM1_3326_58	2209	1.248	1.144	1.361	0.000817	6.261	1077	1.370	1.229	1.529	7.802	<0.0001
SOMA_Q13740_ALCAM_5451_1	2209	1.254	1.148	1.371	0.000829	6.254	1077	1.324	1.218	1.440	10.344	<0.0001
SOMA_Q9Y5W5_WIF1_16070_7	2209	1.236	1.137	1.342	0.000829	6.254	1077	1.163	1.078	1.253	4.074	<0.0001
SOMA_Q9UHY1_NRBP1_12616_45	2209	0.811	0.747	0.88	0.000836	6.25	1077	0.964	0.801	1.160	0.155	0.6991
SOMA_Q14511_NEDD9_12862_14	2209	0.797	0.729	0.871	0.000839	6.249	1077	0.858	0.549	1.341	0.300	0.5012
SOMA_Q6UWI4_SHISA2_10449_31	2209	0.796	0.728	0.871	0.00084	6.249	1077	0.869	0.735	1.028	0.996	0.1009
SOMA_Q9P0T7_TMEM9_8597_1	2209	0.814	0.751	0.883	0.000844	6.247	1077	0.636	0.537	0.753	6.814	<0.0001
SOMA_P42680_TEC_16079_2	2209	0.81	0.746	0.88	0.000848	6.245	1077	0.685	0.584	0.802	5.530	<0.0001
SOMA_P05015_IFNA16_6421_52	2209	0.795	0.726	0.87	0.000849	6.244	1077	0.776	0.497	1.214	0.574	0.2669
SOMA_P27815_PDE4A_18918_86	2209	0.854	0.803	0.908	0.000854	6.241	1077	0.809	0.571	1.146	0.633	0.2327
SOMA_P61952_GNG11_18282_1	2209	0.799	0.732	0.873	0.000854	6.241	1077	0.656	0.509	0.846	2.936	0.0012

SOMA_Q15418_RPS6KA1_12329_21	2209	0.807	0.742	0.878	0.000895	6.221	1077	0.133	0.020	0.882	1.436	0.0367
SOMA_Q7Z5L3_C1QL2_15670_15	2209	0.813	0.75	0.882	0.000895	6.221	1077	0.923	0.754	1.129	0.361	0.4352
SOMA_P41252_IARS_12815_9	2209	0.792	0.723	0.868	0.000899	6.219	NA	NA	NA	NA	NA	NA
SOMA_P0DMV8_HSPA1A_4124_24	2209	1.237	1.138	1.345	0.000902	6.217	1077	1.311	1.214	1.416	11.278	<0.0001
SOMA_Q96HF1_SFRP2_7751_121	2209	0.799	0.732	0.873	0.00091	6.214	1077	0.764	0.543	1.075	0.912	0.1223
SOMA_O95399_UTS2_6020_52	2209	0.799	0.732	0.873	0.000917	6.21	1077	0.910	0.784	1.056	0.670	0.2138
SOMA_Q6DN72_FCRL6_6617_12	2209	0.797	0.729	0.871	0.000919	6.209	1077	0.645	0.480	0.868	2.419	0.0038
SOMA_Q6BAA4_FCRLB_15583_18	2209	1.232	1.135	1.337	0.000922	6.208	1077	0.960	0.777	1.187	0.150	0.708
SOMA_P42701_IL12RB1_2632_5	2209	0.84	0.784	0.899	0.000932	6.203	1077	1.008	0.882	1.152	0.044	0.9034
SOMA_Q9H5P4_PDZD7_12391_27	2209	0.8	0.732	0.873	0.000939	6.2	1077	0.648	0.264	1.590	0.464	0.3434
SOMA_P43628_KIR2DL3_17153_46	2209	1.245	1.142	1.357	0.000942	6.198	1077	1.054	0.981	1.132	0.821	0.1511
SOMA_Q5VU65_NUP210L_9606_4	2209	1.243	1.141	1.355	0.00095	6.195	1077	0.907	0.670	1.228	0.277	0.5287
SOMA_Q9Y5P4_COL4A3B_13535_2	2209	1.226	1.131	1.328	0.00097	6.186	NA	NA	NA	NA	NA	NA
SOMA_Q9NVQ4_FAIM_16594_44	2209	1.246	1.142	1.359	0.000981	6.181	1077	0.890	0.564	1.403	0.211	0.6155
SOMA_Q96PE5_OPALIN_7736_28	2209	0.798	0.729	0.872	0.000985	6.179	1077	0.094	0.017	0.518	2.178	0.0066
SOMA_Q9GZN6_SLC6A16_13056_18	2209	0.849	0.795	0.905	0.000989	6.177	1077	0.993	0.855	1.153	0.033	0.9269
SOMD Q00535 CDK5 CD3358 51 FLAG	2209	0.799	0.731	0.873	0.000989	6.177	NA	NA	NA	NA	NA	NA
SOMD Q15078 CDK5 CD3358 51 FLAG	2209	0.799	0.731	0.873	0.000989	6.177	NA	NA	NA	NA	NA	NA
SOMA_P41182_BCL6_13111_79	2209	0.809	0.744	0.88	0.001	6.171	1077	1.004	0.883	1.142	0.024	0.9457

SOMA_Q8NFB2_TMEM185_14633_26	2209	0.804	0.737	0.876	0.00101	6.167	1077	0.997	0.858	1.158	0.015	0.967
SOMA_Q7Z6I6_ARHGAP3_12807_89	2209	1.239	1.139	1.349	0.00104	6.155	1077	1.023	0.890	1.175	0.125	0.7504
SOMA_P36941_LTBR_2636_10	2209	0.799	0.731	0.873	0.00104	6.154	1077	0.893	0.748	1.066	0.677	0.2104
SOMA_P49959_MRE11A_11319_106	2209	0.803	0.736	0.876	0.00105	6.152	NA	NA	NA	NA	NA	NA
SOMA_A5PKW4_PSD_13055_53	2209	0.804	0.738	0.877	0.00108	6.138	1077	1.029	0.931	1.137	0.237	0.5795
SOMA_P06396_GSN_16607_78	2209	0.803	0.736	0.876	0.00111	6.128	1077	0.723	0.629	0.832	5.259	<0.0001
SOMD P07942 LAMA2 L18347 15 PASS	2209	1.25	1.144	1.365	0.00111	6.125	NA	NA	NA	NA	NA	NA
SOMD P11047 LAMA2 L18347 15 PASS	2209	1.25	1.144	1.365	0.00111	6.125	NA	NA	NA	NA	NA	NA
SOMD P24043 LAMA2 L18347 15 PASS	2209	1.25	1.144	1.365	0.00111	6.125	NA	NA	NA	NA	NA	NA
SOMA_Q9HCR9_PDE11A_5252_33	2209	0.805	0.738	0.877	0.00112	6.124	1077	1.012	0.874	1.172	0.059	0.8722
SOMA_P78543_BTG2_11325_8	2209	0.8	0.732	0.874	0.00114	6.115	1077	0.843	0.599	1.186	0.485	0.3275
SOMA_B1AKI9_ISM1_8355_80	2209	1.235	1.136	1.344	0.00115	6.113	NA	NA	NA	NA	NA	NA
SOMA_P01583_IL1A_4851_25	2209	0.838	0.782	0.899	0.00117	6.106	1077	0.428	0.213	0.862	1.759	0.0174
SOMA_P25774_CTSS_3181_50	2209	1.246	1.142	1.359	0.00117	6.104	1077	1.430	1.251	1.636	6.779	<0.0001
SOMA_Q8NFU4_FDCSP_17346_61	2209	0.806	0.74	0.878	0.0012	6.093	1077	1.029	0.918	1.153	0.207	0.6215
SOMA_Q9Y286_SIGLEC7_2742_68	2209	1.244	1.14	1.356	0.0012	6.092	1077	1.076	1.019	1.136	2.086	0.0082
SOMA_P17405_SMPD1_10818_36	2209	1.242	1.139	1.353	0.00124	6.08	1077	1.265	1.136	1.408	4.728	<0.0001
SOMA_P01591_IGJ_15306_20	2209	0.808	0.742	0.879	0.00124	6.079	NA	NA	NA	NA	NA	NA
SOMA_P13804_ETFA_17435_43	2209	0.827	0.767	0.892	0.00124	6.078	1077	1.015	0.862	1.194	0.064	0.8621

SOMA_O60575_SPINK4_19213_1	2209	1.261	1.15	1.382	0.00125	6.077	1077	1.133	1.054	1.219	3.119	0.0008
SOMA_Q9Y5E7_PCDHB2_10748_216	2209	0.835	0.777	0.897	0.00126	6.074	1077	0.945	0.778	1.147	0.248	0.5647
SOMA_Q9P286_PAK7_3388_58	2209	0.81	0.744	0.881	0.00127	6.069	NA	NA	NA	NA	NA	NA
SOMA_Q96FJ0_STAMBPL_12401_3	2209	0.801	0.733	0.875	0.00128	6.066	1077	0.748	0.617	0.907	2.502	0.0031
SOMA_Q00613_HSF1_11616_9	2209	1.227	1.131	1.332	0.00128	6.065	1077	0.908	0.566	1.459	0.161	0.6907
SOMA_Q13148_TARDBP_12046_51	2209	0.844	0.789	0.903	0.00128	6.064	1077	0.768	0.614	0.959	1.698	0.02
SOMA_P25786_PSMA1_7880_9	2209	0.8	0.732	0.874	0.00129	6.06	1077	0.876	0.615	1.247	0.336	0.4617
SOMA_O60676_CST8_10572_65	2209	1.239	1.138	1.35	0.00132	6.053	1077	1.280	1.156	1.418	5.654	<0.0001
SOMA_Q9ULG6_CCPG1_8889_5	2209	0.809	0.744	0.88	0.00132	6.051	1077	0.100	0.007	1.339	1.086	0.0819
SOMA_Q7L7L0_HIST3H2_14144_3	2209	1.228	1.131	1.332	0.00133	6.047	NA	NA	NA	NA	NA	NA
SOMA_Q9H0T7_RAB17_11118_107	2209	0.801	0.734	0.875	0.00135	6.042	1077	0.893	0.768	1.037	0.857	0.1389
SOMA_Q9H257_CARD9_11405_150	2209	0.809	0.744	0.881	0.00135	6.041	1077	0.889	0.683	1.157	0.419	0.3808
SOMA_Q8WUY8_NAT14_11120_49	2209	0.823	0.761	0.889	0.00136	6.04	1077	0.347	0.152	0.796	1.903	0.0125
SOMA_P78357_CNTNAP1_14617_7	2209	0.867	0.818	0.918	0.00137	6.037	NA	NA	NA	NA	NA	NA
SOMA_Q5SZK8_FREM2_7246_4	2209	0.835	0.776	0.897	0.00138	6.034	1077	1.051	0.900	1.227	0.277	0.5286
SOMA_P12830_CDH1_18429_10	2209	1.235	1.135	1.343	0.00138	6.032	1077	1.087	0.981	1.206	0.956	0.1106
SOMA_P17066_HSPA6_13672_3	2209	0.828	0.768	0.893	0.0014	6.026	1077	0.672	0.533	0.848	3.090	0.0008
SOMA_Q9Y4E8_USP15_19161_1	2209	1.238	1.137	1.348	0.00142	6.021	1077	0.869	0.744	1.017	1.098	0.0797
SOMA_Q92932_PTPRN2_12958_8	2209	0.835	0.777	0.897	0.00142	6.02	NA	NA	NA	NA	NA	NA

SOMA_Q9H6D8_FNDC4_13451_2	2209	0.805	0.738	0.878	0.00143	6.017	1077	0.958	0.769	1.195	0.151	0.7061
SOMA_P12110_COL6A2_9080_1	2209	0.812	0.747	0.883	0.00145	6.01	NA	NA	NA	NA	NA	NA
SOMA_Q9UNP4_ST3GAL5_10588_39	2209	0.859	0.808	0.913	0.00146	6.01	1077	0.971	0.807	1.168	0.122	0.755
SOMA_P20366_TAC1_10503_55	2209	0.805	0.739	0.878	0.00147	6.005	NA	NA	NA	NA	NA	NA
SOMA_Q1W4C9_SPINK13_10624_45	2209	0.851	0.798	0.908	0.00148	6.003	1077	0.042	0.004	0.430	2.124	0.0075
SOMA_Q9UHP7_CLEC2D_7054_87	2209	0.824	0.762	0.89	0.00149	5.999	1077	0.921	0.769	1.104	0.427	0.3742
SOMA_Q8N5W8_FAM24B_8775_61	2209	0.804	0.737	0.878	0.00154	5.986	1077	1.038	0.935	1.152	0.316	0.4829
SOMA_O60565_GREM1_18878_15	2209	1.237	1.136	1.347	0.00157	5.978	1077	1.065	0.993	1.143	1.100	0.0794
SOMA_P15516_HTN3_10603_1	2209	1.229	1.132	1.336	0.00156	5.978	1077	1.078	1.013	1.147	1.746	0.0179
SOMA_Q12792_TWF1_12871_10	2209	1.23	1.132	1.336	0.00156	5.978	1077	1.025	0.890	1.181	0.134	0.7342
SOMA_Q5TEA6_SEL1L2_9082_25	2209	0.81	0.745	0.882	0.00159	5.972	1077	0.793	0.669	0.939	2.140	0.0072
SOMA_P20023_CR2_15570_99	2209	0.807	0.74	0.879	0.00166	5.953	1077	0.695	0.581	0.830	4.209	<0.0001
SOMA_P82279_CRB1_12012_33	2209	0.853	0.8	0.909	0.00166	5.953	NA	NA	NA	NA	NA	NA
SOMA_O14653_GOSR2_10426_21	2209	0.809	0.743	0.881	0.00166	5.952	1077	0.718	0.562	0.917	2.097	0.008
SOMA_Q99895_CTRC_5626_20	2209	0.807	0.74	0.88	0.00166	5.951	1077	0.846	0.590	1.211	0.443	0.3604
SOMA_Q9P0R6_GSKIP_12849_25	2209	1.227	1.13	1.333	0.0017	5.942	1077	1.069	0.967	1.181	0.717	0.1919
SOMA_Q5JS37_NHLRC3_9087_8	2209	0.805	0.738	0.879	0.00174	5.933	1077	0.896	0.628	1.280	0.263	0.5463
SOMA_Q8N1N2_DYNAP_10692_48	2209	1.23	1.131	1.337	0.00174	5.932	NA	NA	NA	NA	NA	NA
SOMA_Q9H074_PAIP1_12430_78	2209	0.808	0.742	0.881	0.00175	5.929	1077	1.019	0.903	1.149	0.117	0.7641

SOMA_P62993_GRB2_5464_52	2209	1.226	1.129	1.331	0.00177	5.926	1077	0.834	0.726	0.958	1.981	0.0104
SOMA_Q00888_PSG4_5649_83	2209	1.237	1.135	1.347	0.00179	5.92	1077	0.976	0.793	1.202	0.086	0.82
SOMA_Q9BZP6_CHIA_16591_71	2209	0.821	0.759	0.889	0.0018	5.916	1077	0.960	0.791	1.164	0.170	0.6764
SOMA_P31946_YWHAB_14156_33	2209	1.221	1.126	1.324	0.00185	5.905	1077	0.798	0.681	0.935	2.280	0.0052
SOMA_Q8TD46_CD200R1_5103_30	2209	0.81	0.744	0.882	0.00193	5.887	1077	1.076	0.980	1.182	0.901	0.1256
SOMA_Q9Y5Y0_FLVCR1_9073_35	2209	0.811	0.745	0.883	0.00194	5.885	NA	NA	NA	NA	NA	NA
SOMA_P48061_CXCL12_3516_60	2209	1.231	1.132	1.34	0.00194	5.884	1077	1.496	1.297	1.726	7.511	<0.0001
SOMA_O95897_OLFM2_19377_14	2209	0.815	0.75	0.885	0.00195	5.882	1077	0.778	0.641	0.944	1.957	0.011
SOMA_O00198_HRK_8008_28	2209	0.803	0.735	0.878	0.00196	5.88	1077	0.842	0.534	1.327	0.339	0.4584
SOMA_Q96IY4_CPB2_3518_54	2209	0.814	0.749	0.885	0.00196	5.88	1077	0.751	0.646	0.872	3.773	0.0002
SOMA_Q6UVW9_CLEC2A_10953_14	2209	0.835	0.777	0.899	0.00197	5.877	1077	1.053	0.957	1.160	0.537	0.2902
SOMA_Q9Y4G6_TLN2_14082_56	2209	1.228	1.13	1.335	0.00198	5.876	1077	0.671	0.481	0.935	1.737	0.0183
SOMA_P23352_KAL1_6603_18	2209	1.231	1.131	1.339	0.00199	5.874	NA	NA	NA	NA	NA	NA
SOMA_P09238_MMP10_8479_4	2209	1.234	1.133	1.344	0.00201	5.87	1077	1.066	0.995	1.142	1.150	0.0708
SOMA_P61086_UBE2K_17380_2	2209	1.232	1.132	1.34	0.00205	5.861	1077	0.868	0.754	0.999	1.316	0.0483
SOMA_O75460_ERN1_11229_16	2209	0.816	0.752	0.886	0.00205	5.86	1077	1.048	0.944	1.163	0.419	0.3812
SOMA_Q16718_NDUFA5_19200_16	2209	0.802	0.733	0.877	0.00206	5.859	1077	0.875	0.540	1.418	0.231	0.588
SOMA_Q9UKF5_ADAM29_13549_15	2209	0.803	0.734	0.878	0.00209	5.853	1077	0.835	0.594	1.174	0.524	0.2992
SOMA_P63208_SKP1_3902_21	2209	0.817	0.752	0.887	0.0021	5.85	1077	0.825	0.657	1.036	1.010	0.0977

SOMA_O15031_PLXNB2_9216_100	2209	1.241	1.137	1.355	0.00212	5.845	1077	1.304	1.175	1.448	6.221	<0.0001
SOMA_P08581_MET_2837_3	2209	0.814	0.748	0.885	0.00226	5.818	1077	0.934	0.801	1.088	0.422	0.3782
SOMA_P43234_CTSO_9264_11	2209	1.222	1.126	1.326	0.00228	5.815	1077	1.137	1.039	1.244	2.292	0.0051
SOMA_P34820_BMP8B_15668_19	2209	0.812	0.746	0.884	0.00229	5.813	1077	0.873	0.735	1.036	0.926	0.1187
SOMA_Q92888_ARHGEF1_13976_9	2209	0.812	0.746	0.884	0.00229	5.812	1077	0.677	0.526	0.871	2.620	0.0024
SOMA_P0CG37_CFC1_3294_55	2209	1.221	1.126	1.325	0.00231	5.809	1077	1.019	0.912	1.140	0.133	0.7356
SOMA_P01106_MYC_10362_35	2209	0.802	0.733	0.878	0.00231	5.808	1077	0.721	0.403	1.287	0.572	0.268
SOMA_Q9BYW2_SETD2_12647_52	2209	0.808	0.741	0.882	0.00231	5.808	1077	0.909	0.691	1.195	0.306	0.4939
SOMA_P54315_PNLIPRP_6627_25	2209	1.237	1.134	1.349	0.00235	5.801	1077	1.177	1.080	1.282	3.697	0.0002
SOMA_Q8N8S7_ENAH_9757_29	2209	1.223	1.126	1.327	0.00236	5.8	1077	0.844	0.732	0.973	1.706	0.0197
SOMA_O95238_SPDEF_10012_5	2209	0.803	0.735	0.879	0.00237	5.798	1077	0.794	0.641	0.984	1.458	0.0348
SOMA_P12319_FCER1A_9254_18	2209	0.804	0.735	0.879	0.00239	5.794	1077	0.737	0.635	0.856	4.179	<0.0001
SOMA_Q8TD33_SCGB1C1_5960_49	2209	1.233	1.132	1.344	0.00241	5.791	NA	NA	NA	NA	NA	NA
SOMA_O43524_FOXO3_11540_37	2209	1.218	1.124	1.321	0.00247	5.779	1077	0.935	0.757	1.155	0.272	0.5351
SOMA_Q14956_GPNMB_8606_39	2209	1.23	1.13	1.338	0.00247	5.779	1077	1.220	1.123	1.326	5.582	<0.0001
SOMA_Q96JW4_SLC41A2_12511_83	2209	0.816	0.751	0.887	0.00248	5.778	1077	0.684	0.277	1.692	0.386	0.4115
SOMA_P84077_ARF1_19239_5	2209	1.233	1.132	1.343	0.0025	5.774	1077	1.053	0.958	1.158	0.544	0.2856
SOMA_P14138_EDN3_15383_200	2209	0.814	0.748	0.886	0.00251	5.773	1077	0.154	0.048	0.490	2.808	0.0016
SOMA_P10909_CLU_4542_24	2209	0.813	0.747	0.885	0.00254	5.768	1077	0.915	0.788	1.064	0.605	0.2485

SOMA_Q92565_RAPGEF5_9059_14	2209	0.806	0.738	0.881	0.00254	5.768	1077	0.979	0.817	1.174	0.085	0.8227
SOMA_Q9NYY1_IL20_4138_25	2209	0.857	0.805	0.913	0.00257	5.763	1077	0.998	0.863	1.153	0.011	0.9756
SOMA_Q9NPJ6_MED4_14021_81	2209	0.814	0.748	0.886	0.00259	5.759	1077	0.464	0.281	0.765	2.575	0.0027
SOMA_P32856_STX2_7738_299	2209	1.229	1.13	1.338	0.00259	5.758	1077	1.029	0.922	1.150	0.218	0.6054
SOMA_P19835_CEL_9796_4	2209	0.814	0.748	0.885	0.00261	5.756	1077	0.779	0.643	0.945	1.952	0.0112
SOMA_O75326_SEMA7A_7019_13	2209	1.227	1.128	1.335	0.00267	5.746	1077	1.167	1.090	1.249	5.082	<0.0001
SOMA_Q9NTG7_SIRT3_17495_141	2209	0.808	0.74	0.882	0.00269	5.743	1077	0.365	0.224	0.594	4.296	<0.0001
SOMA_Q495A1_TIGIT_9638_2	2209	1.232	1.131	1.342	0.00269	5.742	1077	0.812	0.318	2.075	0.178	0.6636
SOMA_Q8IWL3_HSCB_19316_2	2209	0.805	0.737	0.88	0.00277	5.729	1077	0.860	0.736	1.005	1.237	0.058
SOMA_Q86SG7_LYG2_15416_54	2209	0.815	0.749	0.886	0.0028	5.726	1077	1.011	0.857	1.192	0.046	0.899
SOMA_Q9NP95_FGF20_2763_66	2209	0.843	0.786	0.905	0.00281	5.723	1077	0.657	0.423	1.021	1.211	0.0616
SOMA_O00194_RAB27B_13596_3	2209	0.819	0.755	0.889	0.00282	5.722	1077	0.650	0.386	1.093	0.983	0.104
SOMA_Q6UX15_LAYN_2635_61	2209	1.241	1.136	1.357	0.00283	5.72	1077	1.087	1.027	1.151	2.411	0.0039
SOMA_P04090_RLN2_6621_10	2209	0.81	0.743	0.883	0.00288	5.714	1077	0.847	0.666	1.077	0.756	0.1754
SOMD P19875 CXCL3 C 3148 49 FLAG	2209	0.819	0.754	0.889	0.0029	5.709	NA	NA	NA	NA	NA	NA
SOMD P19876 CXCL3 C 3148 49 FLAG	2209	0.819	0.754	0.889	0.0029	5.709	NA	NA	NA	NA	NA	NA
SOMA_Q9NRJ3_CCL28_2890_59	2209	1.227	1.128	1.335	0.00299	5.697	1077	1.123	1.040	1.213	2.495	0.0032
SOMA_Q6P179_ERAP2_8960_3	2209	0.822	0.759	0.891	0.00302	5.693	1077	0.937	0.790	1.112	0.340	0.4573
SOMA_P02042_HBD_6992_67	2209	0.804	0.735	0.88	0.00302	5.692	1077	0.793	0.624	1.009	1.230	0.0589

SOMA_Q96FQ6_S100A16_17836_17	2209	1.225	1.126	1.332	0.00304	5.69	1077	0.914	0.688	1.216	0.269	0.5383
SOMA_O75083_WDR1_10723_41	2209	0.844	0.787	0.905	0.00307	5.685	NA	NA	NA	NA	NA	NA
SOMA_Q86XP6_GKN2_6416_8	2209	1.226	1.127	1.334	0.00307	5.684	1077	1.009	0.884	1.152	0.048	0.8951
SOMA_O14757_CHEK1_2853_68	2209	0.824	0.76	0.892	0.0031	5.681	1077	0.814	0.637	1.041	0.998	0.1005
SOMA_P30042_C21orf3_5981_6	2209	0.806	0.738	0.881	0.00311	5.679	NA	NA	NA	NA	NA	NA
SOMA_Q13489_BIRC3_4973_18	2209	0.806	0.737	0.881	0.00312	5.678	1077	0.802	0.604	1.065	0.893	0.128
SOMA_Q9BQB4_SOST_13101_60	2209	1.23	1.129	1.34	0.00313	5.676	1077	1.101	0.988	1.227	1.080	0.0831
SOMA_Q969E4_TCEAL3_19112_2	2209	1.217	1.122	1.319	0.00318	5.67	1077	1.044	0.939	1.161	0.374	0.4226
SOMA_Q9UBK2_PPARGC1_11825_27	2209	0.862	0.81	0.917	0.00324	5.662	1077	0.633	0.373	1.075	1.043	0.0906
SOMA_P17931_LGALS3_3066_12	2209	1.231	1.13	1.342	0.00329	5.656	1077	1.172	1.028	1.337	1.757	0.0175
SOMA_P29373_CRABP2_11696_7	2209	1.23	1.129	1.34	0.00335	5.647	1077	1.017	0.880	1.175	0.087	0.8191
SOMA_P06748_NPM1_8870_38	2209	0.803	0.734	0.88	0.00336	5.646	1077	0.910	0.728	1.137	0.393	0.4048
SOMA_Q9UBM8_MGAT4C_7208_60	2209	1.223	1.125	1.33	0.00337	5.645	1077	1.034	0.929	1.152	0.270	0.537
SOMA_P07237_P4HB_4278_14	2209	0.841	0.782	0.903	0.00337	5.644	1077	0.444	0.263	0.751	2.611	0.0024
SOMA_Q9UBH0_IL1F5_18375_28	2209	0.81	0.742	0.884	0.00338	5.643	NA	NA	NA	NA	NA	NA
SOMA_Q8WTT0_CLEC4C_9094_5	2209	0.814	0.747	0.886	0.0034	5.64	1077	0.678	0.548	0.838	3.471	0.0003
SOMA_Q00796_SORD_15447_45	2209	0.816	0.75	0.888	0.00344	5.636	1077	0.931	0.786	1.101	0.395	0.4025
SOMA_Q9H4M7_PLEKHA4_10939_16	2209	0.852	0.797	0.911	0.00357	5.62	1077	0.824	0.553	1.228	0.467	0.3409
SOMA_Q8IW00_VSTM4_7242_14	2209	0.805	0.735	0.881	0.00358	5.619	1077	0.768	0.536	1.101	0.823	0.1504

SOMA_Q92575_UBXN4_9997_12	2209	1.217	1.121	1.32	0.00359	5.617	1077	0.885	0.767	1.021	1.031	0.0932
SOMA_Q9Y2R0_COA3_7888_58	2209	0.809	0.741	0.884	0.00359	5.617	1077	0.842	0.672	1.055	0.870	0.1349
SOMA_P08571_CD14_16914_104	2209	1.229	1.128	1.339	0.00367	5.608	1077	1.698	1.512	1.906	18.439	<0.0001
SOMA_Q86VH4_LRRTM4_6572_10	2209	0.814	0.747	0.887	0.00369	5.605	1077	0.656	0.368	1.172	0.810	0.1549
SOMA_Q96GP6_SCARF2_9925_56	2209	1.212	1.119	1.314	0.00372	5.602	1077	1.014	0.882	1.165	0.071	0.8494
SOMA_Q8N474_SFRP1_3221_54	2209	1.233	1.13	1.346	0.00373	5.601	1077	1.137	1.028	1.258	1.914	0.0122
SOMA_P30044_PRDX5_5017_19	2209	0.81	0.742	0.884	0.00392	5.578	1077	0.834	0.420	1.654	0.219	0.6033
SOMA_Q8N8U9_BMPER_15368_3	2209	1.224	1.125	1.331	0.00393	5.578	1077	1.214	1.122	1.314	5.830	<0.0001
SOMA_Q13361_MFAP5_6440_31	2209	0.819	0.754	0.89	0.00396	5.575	1077	0.743	0.406	1.360	0.474	0.336
SOMA_Q6UX41_BTNL8_9026_40	2209	1.214	1.12	1.317	0.00396	5.575	1077	0.808	0.523	1.249	0.472	0.3377
SOMA_Q15628_TRADD_13507_51	2209	0.816	0.749	0.888	0.00398	5.572	1077	0.189	0.087	0.409	4.633	<0.0001
SOMA_P52594_AGFG1_11681_8	2209	1.215	1.12	1.318	0.00401	5.569	1077	0.833	0.720	0.963	1.866	0.0136
SOMA_Q92600_RQCD1_8975_26	2209	1.215	1.12	1.317	0.00404	5.566	NA	NA	NA	NA	NA	NA
SOMA_O15297_PPM1D_11315_148	2209	0.813	0.746	0.887	0.00407	5.563	1077	0.300	0.079	1.142	1.111	0.0775
SOMA_Q9NSD4_ZNF275_9609_25	2209	0.81	0.742	0.885	0.00411	5.559	1077	0.793	0.563	1.118	0.731	0.1859
SOMA_Q7Z6M1_RABEPK_13599_15	2209	0.809	0.741	0.884	0.00411	5.558	1077	0.847	0.644	1.115	0.627	0.2362
SOMA_P43403_ZAP70_3837_6	2209	0.811	0.743	0.885	0.00414	5.556	1077	1.044	0.939	1.161	0.369	0.4275
SOMA_Q8IWU5_SULF2_8305_18	2209	0.822	0.758	0.892	0.00414	5.555	1077	0.754	0.574	0.991	1.364	0.0433
SOMA_Q6UWJ8_CD164L2_6915_2	2209	0.817	0.751	0.889	0.00418	5.551	1077	0.955	0.798	1.144	0.207	0.6205

SOMA_Q8IWT3_CUL9_12991_49	2209	0.849	0.792	0.909	0.00418	5.55	1077	0.959	0.770	1.195	0.150	0.7087
SOMA_P57771_RGS8_11666_72	2209	0.817	0.751	0.889	0.00434	5.535	1077	0.899	0.752	1.076	0.608	0.2467
SOMA_Q96B67_ARRDC3_12352_70	2209	1.224	1.125	1.333	0.00434	5.534	1077	0.910	0.677	1.223	0.274	0.5318
SOMA_P35558_PCK1_18182_24	2209	0.811	0.743	0.886	0.00435	5.533	1077	0.887	0.744	1.057	0.743	0.1807
SOMA_Q6GTX8_LAIR1_11284_24	2209	0.808	0.738	0.883	0.00438	5.531	1077	0.563	0.309	1.026	1.216	0.0607
SOMA_Q9Y5X2_SNX8_6925_26	2209	1.216	1.12	1.321	0.00444	5.524	1077	0.733	0.624	0.859	3.871	0.0001
SOMA_Q4G148_GXYLT1_8229_1	2209	0.816	0.749	0.889	0.00446	5.522	1077	0.940	0.771	1.146	0.268	0.5401
SOMA_Q9BQF6_SENP7_12626_6	2209	0.811	0.743	0.886	0.00457	5.512	1077	1.035	0.930	1.151	0.279	0.526
SOMA_Q8TB73_NDNF_6604_59	2209	0.808	0.739	0.884	0.00472	5.498	1077	1.018	0.889	1.164	0.096	0.8009
SOMA_P12273_PIP_6060_2	2209	0.818	0.751	0.89	0.00476	5.494	1077	0.661	0.540	0.810	4.171	<0.0001
SOMA_P09012_SNRPA_12678_66	2209	1.209	1.116	1.31	0.00483	5.488	1077	0.953	0.799	1.137	0.225	0.5957
SOMA_Q9HDB5_NRXN3_9799_3	2209	0.812	0.744	0.886	0.00483	5.488	NA	NA	NA	NA	NA	NA
SOMA_Q8WZ82_OVCA2_17697_2	2209	1.217	1.12	1.322	0.00483	5.487	1077	0.797	0.689	0.922	2.654	0.0022
SOMA_P03956_MMP1_4924_32	2209	1.218	1.121	1.324	0.00487	5.485	1077	1.371	1.209	1.555	6.072	<0.0001
SOMA_Q8N4V1_MMGT1_7225_51	2209	0.829	0.766	0.897	0.00489	5.483	1077	0.492	0.271	0.892	1.711	0.0194
SOMA_P05062_ALDOB_18185_118	2209	0.816	0.749	0.889	0.00495	5.477	1077	1.014	0.882	1.166	0.075	0.8422
SOMA_Q9Y6H1_CHCHD2_8015_144	2209	0.809	0.739	0.884	0.00499	5.474	NA	NA	NA	NA	NA	NA
SOMA_Q9UK05_GDF2_4880_21	2209	0.813	0.745	0.887	0.00505	5.468	1077	1.007	0.872	1.163	0.035	0.9226
SOMD P27348 YWHABY 4179 57 PASS	2209	1.219	1.121	1.325	0.00506	5.468	NA	NA	NA	NA	NA	NA

SOMD P31946 YWHAB Y 4179 57 PASS	2209	1.219	1.121	1.325	0.00506	5.468	NA	NA	NA	NA	NA	NA
SOMD P31947 YWHAB Y 4179 57 PASS	2209	1.219	1.121	1.325	0.00506	5.468	NA	NA	NA	NA	NA	NA
SOMD P61981 YWHAB Y 4179 57 PASS	2209	1.219	1.121	1.325	0.00506	5.468	NA	NA	NA	NA	NA	NA
SOMD P62258 YWHAB Y 4179 57 PASS	2209	1.219	1.121	1.325	0.00506	5.468	NA	NA	NA	NA	NA	NA
SOMD P63104 YWHAB Y 4179 57 PASS	2209	1.219	1.121	1.325	0.00506	5.468	NA	NA	NA	NA	NA	NA
SOMD Q04917 YWHAB Y 4179 57 PASS	2209	1.219	1.121	1.325	0.00506	5.468	NA	NA	NA	NA	NA	NA
SOMA_Q9GZP0_PDGFD_17140_57	2209	0.82	0.754	0.892	0.00511	5.463	1077	0.692	0.583	0.822	4.567	<0.0001
SOMA_P13928_ANXA8_18290_6	2209	0.811	0.742	0.886	0.00514	5.461	1077	0.979	0.813	1.180	0.083	0.8266
SOMA_P21964_COMT_18382_109	2209	0.816	0.749	0.889	0.00515	5.46	1077	0.455	0.353	0.586	9.019	<0.0001
SOMA_Q96MN2_NLRP4_12794_6	2209	0.813	0.744	0.887	0.00535	5.443	1077	0.840	0.599	1.178	0.504	0.3132
SOMA_Q9P021_CRIPT_19294_26	2209	0.812	0.743	0.887	0.00544	5.436	1077	0.491	0.167	1.446	0.705	0.1971
SOMA_P48431_SOX2_18294_26	2209	0.811	0.742	0.886	0.00546	5.435	1077	1.039	0.906	1.192	0.232	0.586
SOMA_Q13901_C1D_7821_6	2209	0.817	0.751	0.89	0.0055	5.431	1077	0.316	0.054	1.832	0.702	0.1987
SOMA_O14967_CLGN_10600_24	2209	0.825	0.76	0.895	0.00552	5.43	1077	1.018	0.883	1.173	0.092	0.81
SOMA_Q969Q4_ARL11_12433_8	2209	0.823	0.758	0.894	0.00553	5.429	1077	0.618	0.489	0.780	4.275	<0.0001
SOMA_P63167_DYNLL1_3881_49	2209	0.85	0.794	0.911	0.00569	5.417	1077	1.029	0.900	1.176	0.171	0.674
SOMA_P40313_CTRL_9229_9	2209	0.813	0.744	0.887	0.00569	5.416	1077	0.859	0.688	1.072	0.750	0.1777
SOMA_P78552_IL13RA1_2633_52	2209	1.211	1.117	1.314	0.00575	5.412	1077	1.140	1.052	1.235	2.879	0.0013
SOMA_Q96QU1_PCDH15_14228_1	2209	0.83	0.767	0.899	0.00599	5.394	NA	NA	NA	NA	NA	NA

SOMA_O00264_PGRMC1_7863_50	2209	1.216	1.119	1.321	0.00607	5.389	1077	0.993	0.858	1.150	0.032	0.928
SOMA_P51512_MMP16_9719_145	2209	1.22	1.121	1.327	0.0062	5.379	1077	0.982	0.839	1.148	0.088	0.8159
SOMA_Q8IY33_MICALL2_12891_1	2209	0.821	0.755	0.893	0.00622	5.378	1077	0.339	0.113	1.016	1.272	0.0535
SOMA_Q9ULB1_NRXN1_8971_9	2209	0.819	0.752	0.892	0.00631	5.372	1077	0.668	0.550	0.811	4.320	<0.0001
SOMA_P09467_FBP1_7206_20	2209	0.814	0.746	0.889	0.00631	5.371	1077	0.963	0.818	1.133	0.190	0.6464
SOMA_Q9Y5F3_PCDHB1_9941_70	2209	0.818	0.751	0.891	0.00634	5.37	1077	0.713	0.441	1.154	0.772	0.169
SOMA_P61601_NCALD_17816_58	2209	0.836	0.775	0.902	0.00639	5.366	1077	0.077	0.023	0.257	4.493	<0.0001
SOMA_O95994_AGR2_4959_2	2209	0.817	0.75	0.891	0.00644	5.363	1077	0.497	0.307	0.805	2.349	0.0045
SOMA_Q99259_GAD1_11280_6	2209	0.806	0.735	0.884	0.00645	5.362	1077	1.047	0.956	1.147	0.494	0.3209
SOMA_O00212_RHOD_12442_4	2209	1.214	1.118	1.319	0.00649	5.359	1077	1.067	0.963	1.182	0.670	0.214
SOMA_Q9Y696_CLIC4_15314_49	2209	1.206	1.113	1.306	0.00654	5.356	1077	0.811	0.700	0.940	2.273	0.0053
SOMA_Q9Y6W8_ICOS_14084_191	2209	1.224	1.123	1.334	0.00654	5.356	NA	NA	NA	NA	NA	NA
SOMA_O95363_FARS2_13941_82	2209	0.831	0.768	0.899	0.00661	5.351	1077	0.928	0.703	1.227	0.221	0.6008
SOMA_Q9UBP8_KAAG1_19492_5	2209	0.821	0.755	0.893	0.00672	5.344	1077	0.998	0.868	1.148	0.011	0.9747
SOMA_O94768_STK17B_8399_6	2209	0.813	0.744	0.888	0.00678	5.34	1077	0.874	0.639	1.195	0.400	0.3984
SOMA_Q9UKW6_ELF5_13457_33	2209	0.813	0.744	0.888	0.00694	5.33	1077	0.832	0.455	1.522	0.260	0.5498
SOMA_Q7LGC8_CHST3_7189_55	2209	0.815	0.746	0.889	0.00697	5.328	1077	0.663	0.294	1.493	0.494	0.3206
SOMA_P42773_CDKN2C_12521_3	2209	0.829	0.764	0.898	0.00702	5.325	1077	0.783	0.595	1.030	1.095	0.0803
SOMA_P56278_MTCP1_19374_72	2209	0.839	0.778	0.904	0.00703	5.325	1077	0.425	0.138	1.308	0.867	0.1357

SOMA_Q6UWL2_SUSD1_9582_93	2209	0.816	0.748	0.89	0.00704	5.324	1077	0.984	0.829	1.167	0.069	0.8536
SOMA_P30711_GSTT1_19230_12	2209	1.231	1.126	1.345	0.00706	5.323	1077	0.997	0.864	1.150	0.016	0.9644
SOMA_P10809_HSPD1_2682_68	2209	1.21	1.115	1.312	0.00722	5.313	1077	1.063	0.918	1.231	0.382	0.4149
SOMA_Q8WWM9_CYGB_11546_7	2209	1.218	1.119	1.325	0.00725	5.311	1077	0.891	0.668	1.187	0.368	0.429
SOMA_Q6ZP65_CCDC64_11158_40	2209	1.212	1.116	1.316	0.00726	5.31	NA	NA	NA	NA	NA	NA
SOMA_O95972_BMP15_11129_66	2209	0.819	0.751	0.892	0.00754	5.294	1077	0.847	0.620	1.158	0.526	0.2982
SOMA_O95760_IL33_12898_5	2209	0.817	0.748	0.891	0.0077	5.285	NA	NA	NA	NA	NA	NA
SOMA_P48357_LEPR_9566_103	2209	0.814	0.745	0.89	0.00788	5.274	NA	NA	NA	NA	NA	NA
SOMA_Q9H0V9_LMAN2L_8013_9	2209	1.207	1.113	1.31	0.00795	5.271	1077	0.964	0.822	1.130	0.189	0.6478
SOMA_P54296_MYOM2_13534_20	2209	1.216	1.118	1.323	0.00796	5.27	1077	1.047	0.944	1.161	0.413	0.3861
SOMA_Q8IZ57_NRSN1_11654_77	2209	0.816	0.748	0.891	0.008	5.268	1077	1.064	0.979	1.155	0.846	0.1425
SOMA_Q32P28_LEPRE1_6273_58	2209	0.81	0.74	0.887	0.00806	5.265	NA	NA	NA	NA	NA	NA
SOMA_Q07444_KLRC3_7795_14	2209	1.22	1.12	1.329	0.00824	5.255	1077	1.177	1.055	1.314	2.452	0.0035
SOMA_O95754_SEMA4F_9932_49	2209	0.81	0.74	0.887	0.00837	5.248	1077	0.784	0.385	1.597	0.299	0.5026
SOMA_P40616_ARL1_12392_30	2209	0.816	0.747	0.891	0.00844	5.245	1077	0.895	0.722	1.111	0.501	0.3155
SOMA_O00214_LGALS8_4909_68	2209	0.816	0.747	0.891	0.00849	5.242	1077	0.836	0.587	1.189	0.496	0.319
SOMA_Q9NYJ7_DLL3_9974_8	2209	0.824	0.758	0.896	0.00864	5.234	1077	0.121	0.059	0.248	8.100	<0.0001
SOMA_B0FP48_UPK3BL_6633_43	2209	0.813	0.744	0.889	0.00876	5.228	NA	NA	NA	NA	NA	NA
SOMA_Q8NFQ8_TOR1AIP_10553_8	2209	1.213	1.116	1.319	0.00877	5.228	1077	1.081	0.993	1.177	1.147	0.0713

SOMA_P31949_S100A11_14011_17	2209	1.222	1.121	1.333	0.00891	5.221	1077	0.721	0.328	1.584	0.381	0.4158
SOMA_O95926_SYF2_19438_68	2209	1.215	1.117	1.322	0.00908	5.213	1077	0.795	0.640	0.988	1.410	0.0389
SOMA_P00326_ADH1C_15525_294	2209	0.818	0.749	0.892	0.00917	5.208	1077	0.972	0.823	1.148	0.131	0.7389
SOMA_Q9ULW2_FZD10_11647_6	2209	0.818	0.749	0.892	0.00928	5.203	1077	0.858	0.646	1.139	0.540	0.2884
SOMA_P15559_NQO1_9837_60	2209	1.219	1.119	1.328	0.00937	5.199	1077	0.994	0.856	1.155	0.026	0.9422
SOMA_Q5T2T1_MPP7_12732_13	2209	0.843	0.783	0.908	0.00945	5.195	1077	0.957	0.791	1.158	0.188	0.6488
SOMA_Q6PDA7_SPAG11A_5726_49	2209	0.815	0.745	0.891	0.00945	5.195	1077	0.989	0.839	1.167	0.046	0.9
SOMA_Q99075_HBEGF_14094_29	2209	0.825	0.759	0.897	0.00958	5.19	1077	0.657	0.548	0.788	5.211	<0.0001
SOMA_Q6ZMB0_B3GNT6_7082_2	2209	0.817	0.749	0.892	0.00962	5.188	1077	1.069	0.976	1.171	0.820	0.1512
SOMA_P29320_EPHA3_3432_21	2209	0.853	0.796	0.914	0.00963	5.187	1077	1.075	1.011	1.143	1.698	0.02
SOMA_O75781_PALM_18306_1	2209	0.815	0.746	0.891	0.00974	5.182	1077	0.818	0.600	1.115	0.691	0.2039
SOMA_P80511_S100A12_5852_6	2209	1.212	1.115	1.318	0.00985	5.177	1077	1.201	1.122	1.284	6.974	<0.0001
SOMA_Q9UKL4_GJD2_11678_105	2209	0.821	0.754	0.895	0.00989	5.176	1077	0.929	0.718	1.201	0.241	0.5738
SOMA_Q16222_UAP1_13580_2	2209	0.831	0.767	0.901	0.00991	5.175	1077	0.826	0.529	1.291	0.397	0.4011
SOMA_Q9BQY6_WFDC6_13412_5	2209	0.816	0.747	0.891	0.00994	5.174	1077	0.890	0.656	1.208	0.341	0.4557
SOMA_Q96KB5_PBK_17475_18	2209	0.844	0.783	0.909	0.0101	5.165	1077	0.631	0.388	1.028	1.192	0.0643
SOMA_Q8N3T6_TMEM132_7173_141	2209	1.201	1.109	1.301	0.0102	5.163	1077	1.137	1.068	1.212	4.165	<0.0001
SOMA_P11498_PC_13990_1	2209	1.214	1.115	1.321	0.0104	5.153	1077	1.171	1.079	1.270	3.807	0.0002
SOMA_Q8IXQ5_KLHL7_12625_138	2209	0.819	0.75	0.893	0.0104	5.152	1077	0.854	0.651	1.120	0.597	0.253

SOMA_Q9NZI2_KCNIP1_13650_11	2209	0.818	0.75	0.893	0.0105	5.149	1077	1.137	1.052	1.229	2.942	0.0011
SOMA_Q8WVQ1_CANT1_6480_1	2209	1.221	1.119	1.332	0.0107	5.142	1077	1.045	0.932	1.171	0.346	0.4508
SOMA_Q9UBQ0_VPS29_14318_1	2209	0.82	0.752	0.894	0.0107	5.142	1077	0.615	0.442	0.857	2.393	0.004
SOMA_P37802_TAGLN2_11636_33	2209	1.215	1.116	1.324	0.0107	5.14	1077	0.936	0.800	1.094	0.395	0.403
SOMA_Q9BY41_HDAC8_2859_69	2209	0.82	0.752	0.894	0.0107	5.14	1077	0.833	0.707	0.982	1.536	0.0291
SOMA_P53805_RCAN1_13465_5	2209	0.818	0.749	0.893	0.0108	5.136	1077	0.396	0.135	1.156	1.045	0.0902
SOMA_Q969E1_LEAP2_5708_1	2209	1.218	1.117	1.327	0.0108	5.135	1077	1.533	1.356	1.734	11.001	<0.0001
SOMA_P02814_SMR3B_8595_75	2209	0.818	0.749	0.893	0.0109	5.133	NA	NA	NA	NA	NA	NA
SOMA_Q6ZN66_GBP6_7818_101	2209	0.818	0.75	0.893	0.0113	5.118	1077	0.447	0.234	0.851	1.845	0.0143
SOMA_Q7L0J3_SV2A_12880_1	2209	0.822	0.755	0.896	0.0115	5.11	1077	0.968	0.817	1.146	0.152	0.7043
SOMA_P12821_ACE_10714_7	2209	0.825	0.758	0.897	0.0116	5.105	1077	0.654	0.527	0.810	3.979	0.0001
SOMA_Q8IZU9_KIRREL3_4557_61	2209	0.867	0.815	0.923	0.0116	5.104	1077	0.680	0.501	0.922	1.883	0.0131
SOMA_Q92541_RTF1_7741_111	2209	0.818	0.749	0.894	0.012	5.093	1077	0.926	0.689	1.246	0.213	0.6123
SOMA_Q14145_KEAP1_12568_14	2209	0.824	0.756	0.897	0.012	5.092	1077	0.863	0.664	1.121	0.568	0.2703
SOMA_P06870_KLK1_6443_68	2209	0.814	0.743	0.891	0.0121	5.089	NA	NA	NA	NA	NA	NA
SOMA_Q96NY8_PVRL4_5734_13	2209	1.205	1.11	1.308	0.0121	5.086	NA	NA	NA	NA	NA	NA
SOMA_Q4QY38_DEFB134_10610_8	2209	0.847	0.787	0.911	0.0122	5.082	NA	NA	NA	NA	NA	NA
SOMA_Q9UGI8_TES_17419_17	2209	0.813	0.742	0.891	0.0123	5.081	1077	0.000	0.000	0.002	4.429	<0.0001
SOMA_P43359_MAGEA5_18192_69	2209	0.815	0.745	0.892	0.0123	5.08	1077	0.919	0.787	1.074	0.542	0.2873

SOMA_Q8N9I0_SYT2_9577_26	2209	0.826	0.759	0.899	0.0127	5.067	1077	0.992	0.850	1.156	0.039	0.9146
SOMA_P32926_DSG3_16317_20	2209	0.829	0.764	0.901	0.0129	5.06	1077	0.574	0.457	0.721	5.740	<0.0001
SOMD P08476 INHBA I 8467 9 PASS	2209	1.209	1.112	1.314	0.013	5.057	NA	NA	NA	NA	NA	NA
SOMD P09529 INHBA I 8467 9 PASS	2209	1.209	1.112	1.314	0.013	5.057	NA	NA	NA	NA	NA	NA
SOMA_P04808_RLN1_6300_14	2209	1.199	1.107	1.299	0.0132	5.05	NA	NA	NA	NA	NA	NA
SOMA_Q9UBU3_GHRL_6518_85	2209	1.204	1.109	1.307	0.0132	5.049	1077	0.961	0.825	1.119	0.218	0.606
SOMA_Q99650_OSMR_10892_8	2209	0.857	0.801	0.918	0.0133	5.047	1077	1.011	0.878	1.164	0.057	0.8765
SOMA_Q9HD40_SEPSECS_17357_33	2209	0.815	0.745	0.892	0.0134	5.044	1077	0.874	0.678	1.127	0.524	0.2989
SOMA_Q8N554_ZNF276_14692_3	2209	0.82	0.751	0.895	0.0134	5.043	1077	0.872	0.751	1.012	1.145	0.0717
SOMA_O75882_ATRN_15499_11	2209	0.828	0.761	0.9	0.0136	5.037	1077	0.843	0.679	1.047	0.909	0.1232
SOMA_P06737_PYGL_11441_11	2209	0.821	0.753	0.896	0.0141	5.022	1077	0.875	0.743	1.031	0.956	0.1108
SOMA_Q8N6H7_ARFGAP2_11664_32	2209	1.203	1.108	1.305	0.0141	5.021	1077	0.846	0.733	0.977	1.652	0.0223
SOMA_O95868_LY6G6D_6469_62	2209	0.812	0.741	0.891	0.0141	5.019	1077	0.949	0.787	1.144	0.234	0.5832
SOMA_Q9BY32_ITPA_18916_25	2209	1.212	1.113	1.32	0.0143	5.014	1077	0.968	0.836	1.120	0.179	0.6618
SOMA_Q9UKR0_KLK12_3199_54	2209	0.821	0.752	0.896	0.0145	5.01	1077	0.911	0.783	1.059	0.647	0.2254
SOMA_Q9UKJ1_PILRA_8825_4	2209	0.818	0.748	0.894	0.015	4.993	1077	0.858	0.601	1.224	0.400	0.3979
SOMA_Q9Y4D7_PLXND1_19561_216	2209	1.216	1.115	1.326	0.0151	4.99	1077	1.368	1.230	1.522	8.080	<0.0001
SOMA_Q15327_ANKRD1_15361_37	2209	0.819	0.749	0.895	0.0152	4.987	1077	0.959	0.792	1.160	0.178	0.6639
SOMA_Q6B9Z1_IGFL4_6353_60	2209	1.217	1.115	1.328	0.0153	4.985	NA	NA	NA	NA	NA	NA

SOMA_Q86XI6_PPP1R3B_12768_3	2209	0.821	0.751	0.896	0.0153	4.985	1077	0.980	0.833	1.153	0.093	0.8079
SOMA_Q16585_SGCB_7034_4	2209	1.221	1.117	1.334	0.0155	4.98	NA	NA	NA	NA	NA	NA
SOMA_O00763_ACACB_12900_29	2209	0.822	0.754	0.897	0.0157	4.973	NA	NA	NA	NA	NA	NA
SOMA_Q9Y496_KIF3A_13961_18	2209	0.825	0.758	0.899	0.0157	4.973	1077	1.127	0.983	1.293	1.063	0.0865
SOMA_Q86V40_TRABD2A_9401_57	2209	1.202	1.108	1.305	0.0158	4.97	1077	0.856	0.733	0.999	1.319	0.048
SOMA_P35222_CTNNB1_8424_269	2209	0.818	0.748	0.895	0.016	4.965	1077	0.849	0.653	1.104	0.652	0.2229
SOMA_P17927_CR1_19556_12	2209	1.216	1.115	1.327	0.0162	4.96	1077	1.442	1.300	1.599	11.405	<0.0001
SOMA_Q9Y3Z3_SAMHD1_11303_7	2209	0.831	0.766	0.903	0.0163	4.957	1077	0.800	0.370	1.726	0.245	0.5689
SOMA_Q9UBU2_DKK2_15678_71	2209	0.828	0.761	0.901	0.0166	4.95	1077	0.738	0.628	0.868	3.625	0.0002
SOMA_P98155_VLDLR_18938_3	2209	0.821	0.752	0.897	0.0166	4.949	1077	0.587	0.197	1.748	0.470	0.3389
SOMA_Q96PB7_OLFM3_10643_16	2209	0.853	0.795	0.916	0.0166	4.949	1077	0.932	0.727	1.194	0.238	0.578
SOMA_P08236_GUSB_15562_24	2209	0.83	0.764	0.902	0.0167	4.947	1077	0.516	0.413	0.645	8.201	<0.0001
SOMA_P10914_IRF1_17462_19	2209	0.87	0.817	0.926	0.0167	4.947	1077	0.896	0.665	1.206	0.330	0.4682
SOMA_P13385_TDGF1_5810_25	2209	1.203	1.108	1.307	0.0171	4.936	1077	1.219	1.142	1.302	8.566	<0.0001
SOMA_Q9Y5E2_PCDHB7_8071_114	2209	0.831	0.765	0.903	0.0176	4.924	1077	0.332	0.136	0.810	1.813	0.0154
SOMA_Q8NAU1_FNDC5_8041_5	2209	0.816	0.746	0.894	0.018	4.914	NA	NA	NA	NA	NA	NA
SOMA_Q96GG9_DCUN1D1_17366_6	2209	0.831	0.765	0.903	0.0182	4.908	1077	0.629	0.534	0.742	7.477	<0.0001
SOMA_Q92563_SPOCK2_5491_12	2209	0.822	0.753	0.898	0.0183	4.907	1077	1.096	0.947	1.267	0.661	0.2184
SOMA_O00499_BIN1_9574_11	2209	0.822	0.753	0.898	0.0194	4.88	1077	0.900	0.690	1.175	0.358	0.4382

SOMA_P32455_GBP1_15326_64	2209	1.209	1.11	1.317	0.0196	4.876	1077	1.164	1.049	1.291	2.377	0.0042
SOMA_P61244_MAX_12411_60	2209	1.203	1.107	1.307	0.0196	4.875	1077	0.827	0.709	0.965	1.810	0.0155
SOMA_Q14240_EIF4A2_18824_7	2209	1.217	1.114	1.33	0.0197	4.875	1077	1.037	0.930	1.157	0.290	0.5124
SOMA_P55287_CDH11_16305_10	2209	1.209	1.11	1.318	0.0197	4.874	1077	1.138	1.004	1.291	1.363	0.0434
SOMA_P49589_CARS_14098_28	2209	1.208	1.109	1.315	0.0199	4.87	NA	NA	NA	NA	NA	NA
SOMA_Q13563_PKD2_13745_10	2209	0.826	0.757	0.9	0.0199	4.869	1077	0.872	0.498	1.528	0.199	0.6325
SOMA_P15104_GLUL_19238_12	2209	1.209	1.11	1.317	0.0201	4.866	1077	1.446	1.314	1.590	13.425	<0.0001
SOMA_P06239_LCK_3452_17	2209	0.861	0.805	0.921	0.0202	4.864	1077	0.926	0.751	1.140	0.330	0.4679
SOMA_P09681_GIP_16292_288	2209	1.2	1.105	1.303	0.0202	4.864	1077	1.133	1.038	1.237	2.280	0.0053
SOMA_P48739_PITPNB_12484_67	2209	0.819	0.749	0.896	0.0205	4.856	1077	0.794	0.667	0.945	2.031	0.0093
SOMA_Q9UN74_PCDHA4_10533_1	2209	0.825	0.757	0.9	0.0209	4.848	NA	NA	NA	NA	NA	NA
SOMA_Q96DC9_OTUB2_12493_42	2209	0.823	0.754	0.899	0.0213	4.84	1077	0.613	0.402	0.935	1.638	0.023
SOMA_Q6E0U4_DMKN_8535_102	2209	0.826	0.757	0.9	0.0213	4.839	1077	1.239	1.138	1.349	6.105	<0.0001
SOMA_Q96LZ3_PPP3R2_18866_8	2209	0.824	0.755	0.899	0.0216	4.833	1077	0.813	0.651	1.015	1.172	0.0673
SOMA_Q9NX09_DDIT4_17817_22	2209	0.827	0.759	0.901	0.0218	4.83	1077	0.773	0.517	1.154	0.683	0.2076
SOMA_Q8TE54_SLC26A7_13979_3	2209	1.197	1.103	1.299	0.0218	4.829	1077	1.110	1.007	1.224	1.443	0.0361
SOMA_Q53RT3_ASPRV1_13023_8	2209	0.841	0.777	0.91	0.0225	4.815	NA	NA	NA	NA	NA	NA
SOMA_Q9H7M9_C10orf5_11531_24	2209	1.199	1.104	1.302	0.0227	4.812	NA	NA	NA	NA	NA	NA
SOMA_Q9H4A9_DPEP2_8327_26	2209	0.83	0.763	0.903	0.0228	4.81	1077	1.276	1.125	1.447	3.831	0.0001

SOMA_Q9UBT3_DKK4_3365_7	2209	0.831	0.764	0.904	0.0229	4.809	1077	0.809	0.679	0.964	1.753	0.0176
SOMA_Q9UKK9_NUDT5_17761_2	2209	1.201	1.105	1.305	0.0229	4.809	1077	1.004	0.863	1.167	0.018	0.9586
SOMA_Q13115_DUSP4_10035_6	2209	0.843	0.78	0.911	0.023	4.807	1077	0.785	0.552	1.117	0.748	0.1785
SOMA_P52597_HNRNPF_9764_79	2209	1.196	1.103	1.297	0.023	4.805	1077	0.794	0.689	0.914	2.872	0.0013
SOMA_Q99706_KIR2DL4_5095_21	2209	1.201	1.105	1.306	0.0239	4.789	1077	1.058	0.978	1.144	0.803	0.1572
SOMA_Q9HBK9_AS3MT_18417_3	2209	0.826	0.757	0.901	0.0239	4.789	1077	0.965	0.789	1.180	0.138	0.7278
SOMA_P15848_ARSB_3172_28	2209	0.837	0.772	0.908	0.0241	4.786	1077	1.061	0.985	1.143	0.923	0.1193
SOMA_Q99593_TBX5_11202_70	2209	0.827	0.758	0.901	0.0241	4.786	1077	0.550	0.358	0.845	2.197	0.0064
SOMA_P37287_PIGA_10547_42	2209	0.85	0.789	0.915	0.0243	4.782	NA	NA	NA	NA	NA	NA
SOMA_Q16186_ADRM1_9057_19	2209	0.819	0.748	0.897	0.0244	4.78	1077	1.030	0.934	1.137	0.258	0.5521
SOMA_P60022_DEFB1_6629_3	2209	0.827	0.759	0.902	0.0246	4.777	1077	1.028	0.888	1.190	0.145	0.7163
SOMA_Q16773_CCBL1_17365_7	2209	0.828	0.76	0.902	0.0247	4.775	NA	NA	NA	NA	NA	NA
SOMA_Q9NNX6_CD209_3029_52	2209	0.832	0.765	0.905	0.0247	4.775	1077	0.771	0.633	0.938	2.021	0.0095
SOMA_P04279_SEMG1_7115_5	2209	0.831	0.764	0.904	0.0248	4.773	1077	0.855	0.733	0.997	1.336	0.0461
SOMA_P12271_RLBP1_12936_38	2209	0.86	0.803	0.921	0.0249	4.772	1077	0.844	0.589	1.210	0.447	0.357
SOMA_Q9UHQ4_BCAP29_11570_94	2209	0.837	0.772	0.908	0.0251	4.768	1077	0.964	0.789	1.178	0.143	0.7192
SOMA_P08700_IL3_4717_55	2209	0.863	0.807	0.923	0.0257	4.757	1077	0.934	0.733	1.191	0.235	0.5819
SOMA_P62937_PPIA_3844_2	2209	1.195	1.101	1.296	0.0257	4.757	1077	0.828	0.721	0.952	2.089	0.0081
SOMA_Q6AZY7_SCARA3_12915_26	2209	0.84	0.775	0.909	0.0257	4.757	NA	NA	NA	NA	NA	NA

SOMA_P41236_PPP1R2_19152_4	2209	0.829	0.761	0.903	0.0259	4.754	1077	0.651	0.545	0.777	5.666	<0.0001
SOMA_Q9UBR5_CKLF_6102_2	2209	0.821	0.75	0.898	0.0262	4.748	NA	NA	NA	NA	NA	NA
SOMA_P45984_MAPK9_15604_18	2209	1.206	1.107	1.314	0.0264	4.745	1077	0.871	0.737	1.030	0.969	0.1075
SOMA_O75355_ENTPD3_4436_1	2209	0.823	0.753	0.9	0.0266	4.743	1077	0.912	0.778	1.068	0.597	0.2528
SOMA_P07585_DCN_2666_53	2209	1.195	1.101	1.297	0.0273	4.731	1077	1.063	0.995	1.137	1.151	0.0707
SOMA_P78310_CXADR_9258_14	2209	0.869	0.815	0.927	0.0273	4.73	NA	NA	NA	NA	NA	NA
SOMA_Q08334_IL10RB_2631_50	2209	0.823	0.753	0.9	0.0275	4.728	1077	1.027	0.899	1.175	0.159	0.6934
SOMA_Q9BUP3_HTATIP2_7753_21	2209	0.829	0.761	0.903	0.0276	4.726	1077	0.689	0.391	1.216	0.702	0.1985
SOMA_Q14257_RCN2_10645_72	2209	0.823	0.753	0.9	0.0277	4.724	NA	NA	NA	NA	NA	NA
SOMA_Q02413_DSG1_2976_58	2209	0.821	0.751	0.899	0.0278	4.723	1077	0.935	0.732	1.195	0.229	0.5908
SOMA_P54762_EPHB1_17680_12	2209	1.206	1.107	1.314	0.0282	4.717	1077	1.020	0.919	1.132	0.148	0.7106
SOMA_P09874_PARP1_10534_40	2209	0.863	0.806	0.923	0.0284	4.714	1077	0.983	0.841	1.150	0.079	0.8335
SOMA_Q8IUB5_WFDC13_9345_436	2209	0.844	0.781	0.912	0.0287	4.709	1077	0.066	0.020	0.218	5.067	<0.0001
SOMA_P30048_PRDX3_8358_30	2209	1.197	1.102	1.3	0.0289	4.706	1077	1.007	0.871	1.165	0.036	0.9208
SOMA_P43631_KIR2DS2_10428_1	2209	1.202	1.104	1.307	0.0291	4.703	1077	1.236	1.146	1.333	7.412	<0.0001
SOMA_P06307_CCK_6918_183	2209	1.202	1.105	1.308	0.0294	4.697	1077	1.170	1.090	1.256	4.878	<0.0001
SOMA_P04085_PDGFA_4499_21	2209	0.835	0.769	0.907	0.0299	4.691	1077	0.720	0.616	0.841	4.461	<0.0001
SOMA_Q9HBB8_CDHR5_9962_1	2209	0.824	0.754	0.901	0.0304	4.683	1077	0.865	0.402	1.861	0.149	0.7101
SOMA_Q9Y2Z0_SUGT1_17818_22	2209	1.196	1.101	1.298	0.031	4.675	1077	0.841	0.732	0.967	1.830	0.0148

SOMA_Q99935_PROL1_6530_63	2209	0.829	0.76	0.904	0.0313	4.67	NA	NA	NA	NA	NA	NA
SOMA_Q9H1F0_WFDC10A_13429_3	2209	1.204	1.105	1.312	0.0315	4.668	1077	1.066	0.983	1.156	0.904	0.1246
SOMA_Q9Y5G5_PCDHGA8_14635_28	2209	0.873	0.82	0.93	0.0318	4.664	NA	NA	NA	NA	NA	NA
SOMA_Q6UXB1_IGFL3_6961_14	2209	0.827	0.757	0.903	0.0318	4.663	1077	0.852	0.713	1.018	1.105	0.0786
SOMA_Q9BX46_RBM24_11380_84	2209	0.826	0.756	0.902	0.0319	4.662	1077	0.621	0.145	2.657	0.284	0.5203
SOMA_P01024_C3_5803_24	2209	1.216	1.111	1.331	0.032	4.661	1077	1.251	1.174	1.333	11.362	<0.0001
SOMA_P52788_SMS_18179_56	2209	0.861	0.804	0.923	0.0325	4.655	1077	0.972	0.784	1.205	0.099	0.7953
SOMA_Q8WV07_ORAOV1_18195_3	2209	0.825	0.755	0.902	0.0326	4.653	NA	NA	NA	NA	NA	NA
SOMA_O75427_LRCH4_11252_30	2209	0.842	0.778	0.912	0.0326	4.652	1077	1.104	1.015	1.200	1.672	0.0213
SOMA_Q9UQ72_PSG11_7846_44	2209	0.826	0.756	0.902	0.0327	4.651	1077	0.380	0.053	2.713	0.476	0.3343
SOMA_O95249_GOSR1_7805_52	2209	0.826	0.756	0.902	0.0328	4.65	1077	0.173	0.049	0.612	2.189	0.0065
SOMA_Q04756_HGFAC_3617_80	2209	0.836	0.769	0.908	0.0328	4.65	1077	0.711	0.618	0.817	5.809	<0.0001
SOMA_Q969K3_RNF34_13386_248	2209	0.817	0.745	0.897	0.033	4.647	1077	0.949	0.757	1.191	0.185	0.6525
SOMA_Q9NPF2_CHST11_7779_86	2209	1.189	1.097	1.288	0.0335	4.64	1077	1.050	0.971	1.135	0.660	0.219
SOMA_Q9P2D1_CHD7_14005_2	2209	0.827	0.757	0.903	0.0336	4.639	1077	0.873	0.610	1.249	0.339	0.458
SOMA_Q8IVD9_NUDCD3_4254_6	2209	1.189	1.097	1.288	0.0342	4.631	1077	1.089	1.015	1.169	1.765	0.0172
SOMA_Q9NZ52_GGA3_11683_19	2209	1.191	1.098	1.291	0.0344	4.628	1077	0.795	0.671	0.942	2.105	0.0079
SOMA_Q8NEA5_C19orf1_9445_44	2209	0.829	0.76	0.904	0.0349	4.622	1077	0.992	0.844	1.165	0.035	0.9217
SOMA_P13727_PRG2_7258_5	2209	1.198	1.102	1.302	0.035	4.621	1077	1.030	0.933	1.137	0.255	0.5565

SOMA_O95997_PTTG1_17727_1	2209	0.861	0.804	0.923	0.0354	4.616	1077	0.923	0.732	1.163	0.304	0.4963
SOMA_P08238_HSP90AB_5467_15	2209	1.196	1.101	1.3	0.0355	4.615	1077	0.891	0.769	1.031	0.919	0.1205
SOMA_Q9H4P4_RNF41_13411_21	2209	1.194	1.099	1.296	0.0364	4.604	1077	0.959	0.783	1.175	0.163	0.6875
SOMA_P09564_CD7_12008_3	2209	1.201	1.103	1.308	0.0367	4.6	1077	1.109	1.025	1.200	2.013	0.0097
SOMA_O60828_PQBP1_11432_11	2209	0.841	0.776	0.912	0.0386	4.577	1077	0.951	0.756	1.196	0.177	0.6649
SOMA_Q96DB9_FXYD5_10622_9	2209	0.836	0.768	0.909	0.0388	4.576	NA	NA	NA	NA	NA	NA
SOMA_P55075_FGF8_2443_10	2209	0.829	0.759	0.905	0.039	4.574	1077	0.937	0.754	1.164	0.254	0.5573
SOMA_P58400_NRXN1_5110_84	2209	1.2	1.102	1.306	0.0396	4.567	1077	1.027	0.909	1.160	0.173	0.6712
SOMA_O75716_STK16_3471_49	2209	0.825	0.755	0.903	0.0396	4.566	1077	0.755	0.575	0.990	1.377	0.042
SOMA_P52799_EFNB2_8772_5	2209	0.829	0.759	0.905	0.0399	4.564	1077	0.961	0.755	1.224	0.126	0.7476
SOMA_Q96CN7_ISOC1_9816_37	2209	1.198	1.101	1.304	0.0399	4.563	1077	0.887	0.767	1.027	0.964	0.1086
SOMA_P20231_TPSB2_3403_1	2209	1.198	1.101	1.303	0.0401	4.561	1077	1.203	1.050	1.378	2.113	0.0077
SOMA_O95067_CCNB2_18208_3	2209	0.83	0.76	0.905	0.0401	4.56	1077	0.880	0.730	1.061	0.745	0.1798
SOMA_P53999_SUB1_19236_24	2209	1.191	1.098	1.292	0.0403	4.559	1077	1.079	0.963	1.210	0.719	0.191
SOMA_P08631_HCK_3374_49	2209	0.869	0.814	0.928	0.0404	4.558	1077	0.992	0.846	1.163	0.035	0.9228
SOMA_P12259_F5_4906_35	2209	0.839	0.773	0.911	0.0406	4.555	1077	0.735	0.629	0.859	3.979	0.0001
SOMA_Q9NS62_THSD1_5621_64	2209	0.834	0.767	0.908	0.041	4.551	1077	0.868	0.723	1.042	0.887	0.1296
SOMA_Q9NZU0_FLRT3_9128_34	2209	0.825	0.753	0.903	0.0411	4.55	1077	1.015	0.889	1.160	0.084	0.8249
SOMA_P49795_RGS19_12713_365	2209	0.837	0.77	0.91	0.0413	4.548	1077	0.246	0.098	0.615	2.567	0.0027

SOMA_P43626_KIR2DL1_17200_50	2209	0.829	0.759	0.905	0.0417	4.544	1077	0.877	0.702	1.095	0.608	0.2467
SOMA_P20674_COX5A_18226_148	2209	1.192	1.098	1.294	0.0418	4.542	1077	0.917	0.791	1.064	0.596	0.2538
SOMA_Q8N386_LRRC25_9317_4	2209	0.83	0.761	0.906	0.0418	4.542	1077	0.730	0.570	0.936	1.886	0.013
SOMA_P51671_CCL11_5301_7	2209	1.196	1.1	1.3	0.0427	4.533	1077	1.074	0.985	1.171	0.967	0.1078
SOMA_O43715_TRIAP1_18277_28	2209	0.842	0.776	0.913	0.0432	4.528	1077	1.010	0.886	1.152	0.056	0.8792
SOMA_Q9BY11_PACSIN1_12676_1	2209	0.841	0.776	0.912	0.0433	4.527	1077	1.083	0.988	1.186	1.054	0.0883
SOMA_Q9H9Q2_COPS7B_12384_92	2209	0.835	0.767	0.909	0.0436	4.524	1077	0.693	0.560	0.857	3.150	0.0007
SOMA_P0DJ93_SMIM13_13452_113	2209	0.828	0.757	0.905	0.0442	4.518	1077	0.940	0.733	1.205	0.205	0.6236
SOMA_Q9UKJ5_CHIC2_10914_4	2209	0.841	0.775	0.912	0.0445	4.515	NA	NA	NA	NA	NA	NA
SOMA_Q9NR23_GDF3_16755_195	2209	0.827	0.756	0.904	0.0449	4.511	1077	0.907	0.704	1.168	0.349	0.4479
SOMA_Q9UL26_RAB22A_12408_333	2209	0.829	0.759	0.905	0.0451	4.509	1077	1.074	0.964	1.196	0.709	0.1955
SOMA_P11684_SCGB1A1_10569_28	2209	1.199	1.101	1.305	0.0452	4.507	NA	NA	NA	NA	NA	NA
SOMA_Q01201_RELB_11464_9	2209	0.831	0.762	0.907	0.0453	4.507	1077	1.035	0.905	1.185	0.213	0.6126
SOMA_Q96LW7_C9orf89_7778_104	2209	1.204	1.103	1.314	0.0455	4.505	NA	NA	NA	NA	NA	NA
SOMA_P12724_RNASE3_15576_158	2209	1.194	1.098	1.297	0.0456	4.504	1077	1.429	1.277	1.600	9.247	<0.0001
SOMA_P14859_POU2F1_11715_1	2209	0.832	0.763	0.907	0.046	4.5	1077	0.835	0.573	1.219	0.455	0.351
SOMA_Q9NNX1_TUFT1_5690_49	2209	0.833	0.764	0.908	0.046	4.5	1077	0.682	0.420	1.107	0.915	0.1217
SOMA_O60667_FAIM3_6574_11	2209	1.203	1.103	1.312	0.0463	4.497	NA	NA	NA	NA	NA	NA
SOMA_Q9Y2I1_NISCH_12738_43	2209	0.841	0.775	0.912	0.0463	4.497	1077	0.713	0.507	1.003	1.285	0.0519

SOMA_P24158_PRTN3_13720_95	2209	1.203	1.102	1.312	0.0464	4.496	1077	1.313	1.172	1.470	5.596	<0.0001
SOMA_Q8N142_ADSSL1_13998_26	2209	0.833	0.765	0.908	0.047	4.49	NA	NA	NA	NA	NA	NA
SOMA_P04233_CD74_6974_6	2209	0.829	0.759	0.906	0.0475	4.485	1077	1.135	1.049	1.227	2.809	0.0016
SOMA_O95711_LY86_3623_84	2209	1.2	1.101	1.308	0.0481	4.48	1077	1.088	0.975	1.214	0.876	0.133
SOMA_P17676_CEBPB_15675_3	2209	0.83	0.76	0.906	0.0485	4.477	1077	0.920	0.785	1.079	0.516	0.3044
SOMA_O14965_AURKA_3091_70	2209	0.826	0.754	0.904	0.0488	4.474	1077	0.936	0.732	1.196	0.224	0.5973
SOMA_P17022_ZNF18_11372_2	2209	0.835	0.766	0.909	0.0489	4.473	1077	0.996	0.852	1.163	0.020	0.9555
SOMA_Q96CU9_FOXRED1_8017_23	2209	0.838	0.771	0.911	0.0494	4.468	1077	0.643	0.453	0.914	1.861	0.0138
SOMA_Q9H8S9_MOB1A_12426_19	2209	0.828	0.758	0.906	0.0495	4.467	1077	0.634	0.493	0.815	3.415	0.0004
SOMA_Q9P2E9_RRBP1_9913_4	2209	1.195	1.098	1.3	0.0497	4.466	1077	0.974	0.788	1.204	0.092	0.8092
SOMA_Q9NY97_B3GNT2_7980_72	2209	0.832	0.763	0.908	0.0498	4.465	1077	0.988	0.848	1.151	0.058	0.8751
SOMA_O75874_IDH1_18338_26	2209	0.819	0.746	0.9	0.0499	4.464	1077	0.747	0.634	0.881	3.301	0.0005
SOMA_P01861_IGHG4_13231_90	2209	1.2	1.101	1.308	0.0498	4.464	1077	1.001	0.868	1.155	0.006	0.987

Table S10. Results from adjusted cox regression models for death outcomes in the PHFS, replicated in the WashU cohort.

SOMA_ID	PHFS						WashU					
	N	sHR	Standardized.LB.95CI	Standardized.UB.95CI	Alpha.corrected.P.value	minus_log10_P	NA	sHR	Standardized_LB_95CI	Standardized_UB_95CI	minus_log10_P	P_value
SOMA_P61956_SUMO2_19555_1	1867	1.559	1.398	1.738	2.31E-12	14.81	1077	1.206965866	1.134	1.285	8.459	<0.0001
SOMA_Q99988_GDF15_4374_45	1867	1.684	1.479	1.918	5.13E-12	14.465	1077	1.843312345	1.697	2.002	47.022	<0.0001
SOMA_O95633_FSTL3_3438_10	1867	1.66	1.459	1.889	2.03E-11	13.865	1077	1.646905598	1.525	1.778	36.345	<0.0001
SOMA_Q9H0R8_GABARAP_12661_44	1867	1.529	1.371	1.706	4.32E-11	13.539	1077	1.08302897	1.028	1.141	2.576	0.0027
SOMA_P61769_B2M_3485_28	1867	1.57	1.394	1.769	1.92E-10	12.89	1077	1.333803109	1.252	1.421	18.196	<0.0001
SOMA_O95166_GABARAP_17735_130	1867	1.495	1.344	1.663	1.92E-10	12.889	1077	1.251274061	1.185	1.321	15.276	<0.0001
SOMA_Q4LDE5_SVEP1_11109_56	1867	1.601	1.41	1.817	4.85E-10	12.488	1077	1.477912121	1.378	1.585	27.295	<0.0001
SOMA_Q12805_EFEMP1_8480_29	1867	1.583	1.399	1.792	5.62E-10	12.423	1077	1.414966317	1.329	1.506	26.964	<0.0001
SOMA_Q4LDE5_SVEP1_11178_21	1867	1.591	1.4	1.808	1.59E-09	11.973	1077	1.501023415	1.397	1.612	28.003	<0.0001
SOMA_P51858_HDGF_8953_47	1867	1.403	1.276	1.542	4.15E-09	11.555	1077	1.096267692	1.024	1.174	2.081	0.0083
SOMA_O00244_ATOX1_19233_75	1867	1.469	1.318	1.636	4.80E-09	11.492	1077	1.10645743	1.051	1.165	3.884	0.0001
SOMA_P01034_CST3_2609_59	1867	1.533	1.359	1.728	4.82E-09	11.49	1077	1.439708604	1.341	1.546	22.925	<0.0001
SOMA_Q86TH1_ADAMTSL_6379_62	1867	1.45	1.305	1.612	8.16E-09	11.261	1077	1.684438113	1.544	1.838	30.996	<0.0001
SOMA_P07998_RNASE1_7211_2	1867	1.499	1.334	1.683	1.37E-08	11.037	1077	1.246783262	1.177	1.320	13.347	<0.0001
SOMA_Q9UBX5_FBLN5_15585_304	1867	1.473	1.318	1.648	1.64E-08	10.959	1077	2.19702729	1.971	2.449	44.982	<0.0001

SOMA_P00533_EGFR_2677_1	1867	0.699	0.628	0.778	7.07E-08	10.324	1077	0.588232785	0.506	0.683	11.421	<0.0001
SOMA_Q9HCB6_SPON1_4297_62	1867	1.497	1.327	1.689	8.20E-08	10.259	1077	1.493970509	1.392	1.603	28.245	<0.0001
SOMA_P07108_DBI_16919_1	1867	1.42	1.276	1.579	1.84E-07	9.907	1077	1.243996306	1.135	1.363	5.541	<0.0001
SOMA_P0DJI8_SAA1_15515_2	1867	1.385	1.254	1.529	1.94E-07	9.884	1077	1.572084811	1.452	1.702	28.311	<0.0001
SOMA_Q93091_RNASE6_5646_20	1867	1.415	1.271	1.574	3.12E-07	9.679	1077	1.178900501	1.117	1.245	8.526	<0.0001
SOMA_Q14508_WFDC2_11388_75	1867	1.484	1.314	1.676	3.17E-07	9.672	1077	1.91650158	1.737	2.115	37.520	<0.0001
SOMA_Q2UY09_COL28A1_10702_1	1867	1.454	1.294	1.632	3.75E-07	9.599	1077	1.39442374	1.297	1.499	18.761	<0.0001
SOMA_P52823_STC1_4930_21	1867	1.384	1.249	1.533	7.51E-07	9.297	1077	1.666572227	1.518	1.830	26.010	<0.0001
SOMA_Q96DZ1_ERLEC1_8957_72	1867	1.387	1.25	1.538	8.75E-07	9.231	1077	1.123107128	1.048	1.203	3.029	0.0009
SOMA_P67809_YBX1_9751_72	1867	1.362	1.235	1.503	1.05E-06	9.15	1077	1.350464184	1.234	1.478	10.154	<0.0001
SOMA_Q9BQT9_CLSTN3_6291_55	1867	1.409	1.263	1.572	1.13E-06	9.121	1077	1.33450056	1.236	1.441	12.679	<0.0001
SOMA_Q07654_TFF3_8323_163	1867	1.421	1.27	1.589	1.15E-06	9.113	1077	1.141254895	1.063	1.226	3.546	0.0003
SOMA_P12111_COL6A3_11196_31	1867	1.436	1.28	1.612	1.16E-06	9.108	1077	1.270693692	1.193	1.353	13.000	<0.0001
SOMA_Q01974_ROR2_7861_9	1867	1.427	1.274	1.598	1.18E-06	9.103	1077	1.242805536	1.170	1.320	11.674	<0.0001
SOMA_P35442_THBS2_3339_33	1867	1.453	1.29	1.636	1.20E-06	9.096	1077	1.760384365	1.625	1.907	42.975	<0.0001
SOMA_Q86Y30_BAGE2_6294_11	1867	1.377	1.243	1.526	1.43E-06	9.019	1077	1.118516828	1.020	1.226	1.770	0.017
SOMA_Q9H4D0_CLSTN2_18882_7	1867	1.402	1.258	1.563	1.63E-06	8.961	1077	1.290485293	1.222	1.363	19.345	<0.0001
SOMA_P22223_CDH3_2643_57	1867	0.702	0.627	0.787	1.68E-06	8.947	1077	0.375557545	0.320	0.441	31.865	<0.0001
SOMA_P51858_HDGF_16758_96	1867	1.352	1.227	1.49	1.74E-06	8.933	1077	1.123859283	1.049	1.204	3.069	0.0009

SOMA_Q9NP99_TREM1_9266_1	1867	1.41	1.262	1.575	1.77E-06	8.926	1077	1.448201858	1.350	1.553	24.491	<0.0001
SOMA_O60760_HPGDS_12549_33	1867	0.745	0.677	0.819	1.82E-06	8.913	1077	0.336287278	0.261	0.434	16.290	<0.0001
SOMA_Q01638_IL1RL1_4234_8	1867	1.402	1.257	1.564	1.84E-06	8.909	1077	1.478757658	1.387	1.576	32.380	<0.0001
SOMA_Q01995_TAGLN_15640_54	1867	1.489	1.308	1.694	2.36E-06	8.799	1077	1.374296462	1.287	1.467	20.666	<0.0001
SOMA_P61626_LYZ_4920_10	1867	1.405	1.258	1.569	2.42E-06	8.789	1077	1.363669165	1.263	1.472	14.655	<0.0001
SOMA_Q07654_TFF3_4721_54	1867	1.421	1.267	1.593	2.61E-06	8.757	1077	1.243202055	1.161	1.332	9.258	<0.0001
SOMA_P61916_NPC2_6259_60	1867	1.393	1.249	1.553	3.39E-06	8.642	NA	NA	NA	NA	NA	NA
SOMA_Q9Y5H3_PCDHGA1_6321_65	1867	1.401	1.254	1.565	3.67E-06	8.608	1077	1.039773942	0.961	1.125	0.482	0.3293
SOMA_P06276_BCHE_15514_26	1867	0.713	0.637	0.798	6.00E-06	8.395	1077	0.367614632	0.312	0.434	31.770	<0.0001
SOMA_P40121_CAPG_4968_50	1867	1.365	1.23	1.515	7.22E-06	8.314	1077	1.272793806	1.177	1.376	8.823	<0.0001
SOMA_P05452_CLEC3B_5701_81	1867	0.743	0.672	0.821	8.33E-06	8.252	1077	0.479869325	0.411	0.560	19.874	<0.0001
SOMA_Q9BX93_PLA2G12_9380_2	1867	0.732	0.659	0.813	8.40E-06	8.249	1077	0.370605003	0.298	0.462	18.092	<0.0001
SOMA_Q9UHV9_PFDN2_19243_2	1867	1.367	1.23	1.519	9.91E-06	8.177	1077	1.127492663	1.061	1.198	3.986	0.0001
SOMA_P59901_LILRA4_8299_66	1867	0.76	0.692	0.834	1.19E-05	8.097	1077	0.410149508	0.302	0.557	7.972	<0.0001
SOMA_P19823_ITIH2_9326_33	1867	0.754	0.685	0.83	1.37E-05	8.036	1077	0.62605304	0.547	0.717	10.964	<0.0001
SOMA_P17900_GM2A_15441_6	1867	1.403	1.25	1.575	1.44E-05	8.015	1077	1.337917148	1.253	1.428	17.641	<0.0001
SOMA_P0DJI9_SAA2_18832_65	1867	1.335	1.209	1.474	1.46E-05	8.008	1077	1.259818905	1.192	1.332	15.503	<0.0001
SOMA_Q8WWZ8_OIT3_6296_36	1867	1.353	1.22	1.501	1.67E-05	7.951	1077	1.214325049	1.121	1.315	5.745	<0.0001
SOMA_P14555_PLA2G2A_2692_74	1867	1.344	1.215	1.488	1.68E-05	7.948	1077	1.30496317	1.225	1.390	15.785	<0.0001

SOMA_Q9Y5P4_COL4A3B_13950_9	1867	1.361	1.224	1.513	1.93E-05	7.888	NA	NA	NA	NA	NA	NA
SOMA_P01011_SERPINA_4153_11	1867	1.36	1.223	1.512	2.15E-05	7.841	1077	1.413559087	1.336	1.495	32.713	<0.0001
SOMA_Q8IUL8_CILP2_8841_65	1867	0.741	0.668	0.822	2.32E-05	7.808	1077	0.379306615	0.303	0.474	16.778	<0.0001
SOMA_P55957_BID_5798_3	1867	1.325	1.202	1.462	2.63E-05	7.753	1077	1.067982255	1.001	1.139	1.338	0.046
SOMA_Q76LX8_ADAMTS1_3175_51	1867	0.746	0.674	0.827	3.02E-05	7.693	1077	0.533224567	0.456	0.624	14.301	<0.0001
SOMA_Q01844_EWSR1_12988_49	1867	1.339	1.208	1.483	3.78E-05	7.596	1077	1.367713662	1.257	1.488	12.501	<0.0001
SOMA_P42167_TMPO_8265_225	1867	1.35	1.214	1.502	4.70E-05	7.501	1077	1.252518895	1.145	1.371	6.013	<0.0001
SOMA_P39060_COL18A1_2201_17	1867	1.366	1.223	1.525	4.80E-05	7.492	1077	1.533551798	1.426	1.649	29.980	<0.0001
SOMA_P62995_TRA2B_12373_73	1867	1.328	1.2	1.47	6.02E-05	7.393	1077	1.189627602	1.131	1.251	10.923	<0.0001
SOMA_P51965_UBE2E1_14326_4	1867	1.322	1.196	1.461	6.68E-05	7.348	1077	1.071345497	0.936	1.227	0.497	0.3181
SOMA_O75475_PSIP1_17176_13	1867	1.309	1.188	1.442	7.12E-05	7.32	1077	1.196410291	1.112	1.287	5.866	<0.0001
SOMA_P07949_RET_3220_40	1867	0.752	0.678	0.833	8.86E-05	7.225	1077	0.251606523	0.170	0.372	11.350	<0.0001
SOMA_Q92626_PXDN_13463_1	1867	1.381	1.228	1.553	0.0001111	7.127	1077	1.281957894	1.206	1.363	14.730	<0.0001
SOMA_Q99727_TIMP4_6462_12	1867	1.32	1.193	1.461	0.0001112	7.127	1077	1.303740852	1.190	1.429	7.880	<0.0001
SOMA_Q9BUJ0_ABHD14A_5715_4	1867	1.318	1.192	1.458	0.0001171	7.104	1077	1.092793106	0.990	1.206	1.112	0.0774
SOMA_O15123_ANGPT2_2602_2	1867	1.406	1.241	1.592	0.0001208	7.091	1077	1.922764974	1.745	2.119	39.056	<0.0001
SOMA_Q93045_STMN2_10900_272	1867	1.314	1.189	1.452	0.0001206	7.091	1077	1.068184803	0.983	1.160	0.927	0.1183
SOMA_Q6P988_NOTUM_8252_2	1867	0.744	0.668	0.829	0.0001228	7.084	1077	0.217291702	0.156	0.303	18.728	<0.0001
SOMA_P08949_NMB_9321_400	1867	1.355	1.213	1.514	0.0001231	7.083	1077	1.103377349	1.042	1.169	3.082	0.0008

SOMA_Q9Y274_ST3GAL6_6947_4	1867	0.759	0.686	0.839	0.0001298	7.06	1077	0.602415202	0.527	0.689	12.983	<0.0001
SOMA_P00797_REN_3396_54	1867	1.296	1.178	1.425	0.0001485	7.001	1077	1.407744496	1.252	1.583	7.917	<0.0001
SOMA_O14737_PDCD5_12517_52	1867	1.328	1.196	1.474	0.0001519	6.991	1077	1.337015893	1.172	1.525	4.831	<0.0001
SOMD O14793 GDF11M 2765 4 PASS	1867	0.769	0.698	0.847	0.0001536	6.986	NA	NA	NA	NA	NA	NA
SOMD O95390 GDF11M 2765 4 PASS	1867	0.769	0.698	0.847	0.0001536	6.986	NA	NA	NA	NA	NA	NA
SOMA_P58335_ANTXR2_15559_5	1867	0.77	0.7	0.848	0.0001582	6.974	1077	0.511694299	0.427	0.613	12.355	<0.0001
SOMA_Q4KMG0_CDON_4541_49	1867	0.773	0.703	0.85	0.0001745	6.931	1077	0.674977705	0.568	0.803	5.068	<0.0001
SOMA_O60330_PCDHGA1_6938_21	1867	1.346	1.206	1.503	0.0001938	6.886	1077	1.067325169	0.999	1.141	1.266	0.0542
SOMA_P15090_FABP4_15386_7	1867	1.328	1.195	1.475	0.000208	6.855	1077	1.778072001	1.600	1.975	26.065	<0.0001
SOMA_Q9NUQ9_FAM49B_19176_27	1867	1.292	1.174	1.421	0.0002265	6.818	NA	NA	NA	NA	NA	NA
SOMA_P15814_IGLL1_6485_59	1867	0.77	0.698	0.849	0.0002376	6.797	1077	0.621474708	0.496	0.778	4.483	<0.0001
SOMA_P49773_HINT1_5900_11	1867	1.311	1.185	1.451	0.0002435	6.786	1077	0.994142585	0.855	1.155	0.027	0.9389
SOMA_P36776_LONP1_6398_12	1867	1.279	1.166	1.402	0.0002569	6.763	1077	0.793343639	0.676	0.931	2.347	0.0045
SOMA_P09651_HNRNPA1_12466_7	1867	1.282	1.168	1.407	0.0002694	6.742	1077	1.028629761	0.895	1.182	0.161	0.6903
SOMA_O95990_FAM107A_2760_2	1867	0.816	0.756	0.881	0.0002718	6.739	1077	0.814506422	0.554	1.198	0.527	0.2971
SOMA_Q9NZ72_STMN3_8019_73	1867	1.27	1.16	1.39	0.0003421	6.639	1077	1.085480526	1.020	1.156	1.986	0.0103
SOMA_O95445_APOM_10445_20	1867	0.771	0.698	0.852	0.0004674	6.503	1077	0.491144499	0.371	0.651	6.137	<0.0001
SOMA_P05413_FABP3_5437_63	1867	1.286	1.168	1.417	0.0005063	6.468	1077	1.452441956	1.333	1.583	16.814	<0.0001
SOMA_Q9Y3E7_VPS24_12508_9	1867	1.301	1.175	1.441	0.0006399	6.367	NA	NA	NA	NA	NA	NA

SOMA_Q13291_SLAMF1_7953_20	1867	1.278	1.162	1.405	0.000667	6.349	1077	1.051388292	0.978	1.130	0.755	0.1756
SOMA_Q9BXJ4_C1QTNF3_7251_64	1867	0.792	0.723	0.867	0.0006699	6.347	1077	0.302262699	0.235	0.388	20.203	<0.0001
SOMA_O94856_NFASC_7179_69	1867	1.292	1.17	1.428	0.0006825	6.339	1077	1.327462603	1.233	1.429	13.368	<0.0001
SOMA_Q6ZTQ4_CDHR3_8222_49	1867	0.791	0.722	0.867	0.0007024	6.326	1077	0.94230835	0.757	1.173	0.226	0.5942
SOMA_P03973_SLPI_4413_3	1867	1.31	1.179	1.456	0.0007706	6.286	1077	1.159409963	1.106	1.216	9.058	<0.0001
SOMA_Q16629_SRSF7_12987_12	1867	1.306	1.177	1.45	0.0008267	6.255	1077	1.507017036	1.395	1.628	24.666	<0.0001
SOMA_Q6UXM1_LRIG3_3322_52	1867	0.784	0.712	0.863	0.0009681	6.187	1077	0.827834823	0.714	0.960	1.913	0.0122
SOMA_Q8TDY8_IGDCC4_9793_145	1867	0.78	0.707	0.86	0.0009827	6.18	1077	0.692042754	0.487	0.984	1.391	0.0406
SOMA_Q9C005_DPY30_13943_38	1867	1.304	1.174	1.45	0.001157	6.109	1077	1.448381297	1.322	1.587	14.757	<0.0001
SOMA_P00747_PLG_3710_49	1867	0.763	0.685	0.85	0.001254	6.074	1077	0.45505155	0.386	0.536	20.219	<0.0001
SOMA_P35858_IGFALS_6605_17	1867	0.774	0.699	0.857	0.001263	6.071	1077	0.543838008	0.470	0.629	15.710	<0.0001
SOMA_Q9C075_KRT23_14237_1	1867	0.789	0.718	0.867	0.001276	6.067	NA	NA	NA	NA	NA	NA
SOMA_Q9UJJ9_GNPTG_10666_7	1867	1.307	1.175	1.455	0.001378	6.033	1077	1.462358461	1.350	1.584	19.951	<0.0001
SOMA_Q07960_ARHGAP1_11955_1	1867	1.28	1.16	1.413	0.001417	6.021	1077	0.991861233	0.845	1.164	0.036	0.9201
SOMA_Q9H6Z4_RANBP3_14037_18	1867	1.308	1.174	1.456	0.001535	5.986	1077	1.371874006	1.258	1.496	12.136	<0.0001
SOMD P02671 FGA FGB 4907 56 PASS	1867	1.267	1.152	1.393	0.001613	5.965	NA	NA	NA	NA	NA	NA
SOMD P02675 FGA FGB 4907 56 PASS	1867	1.267	1.152	1.393	0.001613	5.965	NA	NA	NA	NA	NA	NA
SOMD P02679 FGA FGB 4907 56 PASS	1867	1.267	1.152	1.393	0.001613	5.965	NA	NA	NA	NA	NA	NA
SOMA_O60911_CTSV_3364_76	1867	0.786	0.714	0.866	0.001625	5.962	1077	0.729634426	0.491	1.085	0.922	0.1196

SOMA_P09529_INHBB_13676_46	1867	1.304	1.172	1.452	0.001623	5.962	1077	1.289047204	1.213	1.370	15.569	<0.0001
SOMA_P62979_RPS27A_2846_24	1867	1.262	1.149	1.385	0.001721	5.937	1077	1.042353018	0.910	1.194	0.261	0.5487
SOMA_Q8IZS8_CACNA2D_8885_6	1867	0.787	0.715	0.867	0.001846	5.906	1077	0.667503177	0.558	0.798	5.028	<0.0001
SOMA_Q92743_HTRA1_15594_47	1867	1.299	1.169	1.444	0.001845	5.906	1077	1.583036969	1.442	1.738	21.226	<0.0001
SOMA_Q9UNK0_STX8_10903_50	1867	1.303	1.171	1.45	0.001867	5.901	1077	1.102278075	0.966	1.257	0.834	0.1465
SOMA_Q01105_SET_5364_7	1867	0.771	0.694	0.857	0.001894	5.895	1077	0.512004782	0.438	0.598	16.512	<0.0001
SOMA_P16152_CBR1_12381_26	1867	1.305	1.172	1.454	0.001968	5.878	1077	1.471592157	1.363	1.588	22.467	<0.0001
SOMA_P19438_TNFRSF1_2654_19	1867	1.304	1.171	1.452	0.002077	5.855	1077	1.420242272	1.327	1.520	23.540	<0.0001
SOMA_Q06141_REG3A_15304_1	1867	1.322	1.18	1.481	0.002296	5.811	1077	1.399010626	1.315	1.489	25.410	<0.0001
SOMA_Q02487_DSC2_13126_52	1867	1.328	1.183	1.491	0.002367	5.798	1077	1.237714598	1.164	1.317	10.876	<0.0001
SOMA_Q8TDN1_KCNG4_13525_17	1867	0.794	0.723	0.872	0.002367	5.798	1077	0.55574989	0.418	0.738	4.302	<0.0001
SOMA_Q99729_HNRNPAB_8894_80	1867	1.251	1.142	1.371	0.002393	5.793	1077	1.028772681	0.908	1.165	0.184	0.655
SOMA_Q9Y3E2_BOLA1_15370_5	1867	1.281	1.158	1.417	0.002401	5.792	1077	1.083087315	0.988	1.187	1.060	0.0871
SOMA_O60704_TPST2_8024_64	1867	0.801	0.731	0.877	0.002413	5.79	1077	0.569257084	0.463	0.700	7.002	<0.0001
SOMA_Q9HD15_SRA1_18220_141	1867	1.288	1.162	1.429	0.002439	5.785	1077	1.226896109	1.114	1.351	4.518	<0.0001
SOMA_Q16627_CCL14_2900_53	1867	1.325	1.181	1.487	0.002463	5.781	1077	1.621687824	1.510	1.741	39.856	<0.0001
SOMA_Q9BU40_CHRDL1_3362_61	1867	1.338	1.188	1.507	0.002495	5.775	1077	1.631698348	1.486	1.791	24.031	<0.0001
SOMA_Q9NR71_ASAH2_3212_30	1867	0.771	0.693	0.858	0.00266	5.747	1077	0.356589905	0.277	0.459	14.986	<0.0001
SOMA_Q9UKP5_ADAMTS6_6441_62	1867	1.277	1.155	1.412	0.002674	5.745	1077	1.097467485	0.978	1.232	0.942	0.1143

SOMA_P02741_CRP_4337_49	1867	1.269	1.151	1.4	0.002687	5.743	1077	1.67685398	1.447	1.944	11.169	<0.0001
SOMA_Q9NRM6_IL17RB_6262_14	1867	1.255	1.143	1.377	0.002755	5.732	1077	0.802387006	0.683	0.943	2.119	0.0076
SOMA_P02679_FGG_4989_7	1867	1.25	1.14	1.37	0.002863	5.716	1077	1.474250288	1.341	1.620	15.064	<0.0001
SOMA_Q9P0K1_ADAM22_7933_75	1867	0.805	0.736	0.881	0.003082	5.683	1077	0.998355642	0.852	1.169	0.007	0.9837
SOMA_P15907_ST6GAL1_6035_2	1867	1.262	1.146	1.389	0.003092	5.682	1077	1.095372165	0.994	1.207	1.185	0.0653
SOMA_Q86Y82_STX12_10418_36	1867	1.276	1.154	1.412	0.003389	5.642	1077	1.122786044	0.990	1.273	1.147	0.0713
SOMA_Q9BQI0_AIF1L_18871_24	1867	1.316	1.174	1.475	0.003762	5.597	1077	1.379658031	1.257	1.514	10.966	<0.0001
SOMA_P30050_RPL12_19183_164	1867	1.264	1.146	1.394	0.003783	5.594	1077	1.345098932	1.212	1.493	7.605	<0.0001
SOMA_Q8NBJ4_GOLM1_17456_53	1867	1.29	1.16	1.434	0.003876	5.584	1077	1.271140098	1.204	1.342	17.346	<0.0001
SOMA_P29353_SHC1_5272_55	1867	1.263	1.146	1.393	0.004222	5.546	1077	0.94741683	0.818	1.097	0.329	0.4692
SOMA_Q86Z14_KLB_19557_3	1867	0.793	0.72	0.874	0.004369	5.532	1077	0.729843634	0.592	0.900	2.500	0.0032
SOMA_Q5JXA9_SIRPB2_5669_26	1867	1.261	1.144	1.39	0.004412	5.527	1077	1.08854446	0.985	1.203	1.016	0.0964
SOMA_P68402_PAFAH1B_2642_4	1867	1.266	1.147	1.399	0.004596	5.51	1077	1.092097023	1.022	1.167	2.016	0.0096
SOMA_P43652_AFM_4763_31	1867	0.786	0.71	0.87	0.004667	5.503	1077	0.493993016	0.428	0.571	20.954	<0.0001
SOMA_O00622_CYR61_6264_9	1867	1.274	1.151	1.411	0.004828	5.488	NA	NA	NA	NA	NA	NA
SOMA_P46108_CRK_4976_57	1867	1.267	1.147	1.4	0.004934	5.479	1077	1.168436468	1.013	1.348	1.483	0.0329
SOMA_Q9GZX9_TWSG1_9234_8	1867	1.308	1.168	1.465	0.005017	5.471	1077	1.412961278	1.316	1.517	20.771	<0.0001
SOMA_P08697_SERPINF_3024_18	1867	0.785	0.709	0.87	0.005093	5.465	1077	0.682785869	0.592	0.787	6.848	<0.0001
SOMA_P83916_CBX1_18817_50	1867	1.241	1.133	1.359	0.005089	5.465	1077	0.707445462	0.371	1.349	0.533	0.2932

SOMA_Q16270_IGFBP7_3320_49	1867	1.302	1.165	1.456	0.00514	5.461	1077	1.773689649	1.610	1.954	30.299	<0.0001
SOMA_P11686_SFTPC_5738_25	1867	1.287	1.157	1.431	0.005175	5.458	NA	NA	NA	NA	NA	NA
SOMA_Q92838_EDA_2826_53	1867	0.809	0.739	0.885	0.005318	5.446	1077	1.067683734	0.979	1.164	0.857	0.1391
SOMA_Q13145_BAMBI_8811_24	1867	1.279	1.152	1.419	0.005563	5.426	1077	1.172311413	1.095	1.255	5.267	<0.0001
SOMA_Q6UX46_FAM150B_6284_7	1867	1.253	1.139	1.378	0.005583	5.425	NA	NA	NA	NA	NA	NA
SOMA_P22466_GAL_13389_8	1867	0.797	0.723	0.877	0.00562	5.422	NA	NA	NA	NA	NA	NA
SOMA_P18440_NAT1_12632_14	1867	0.793	0.718	0.875	0.006331	5.37	1077	0.321448021	0.241	0.429	13.869	<0.0001
SOMA_Q12906_ILF3_12759_47	1867	1.264	1.144	1.398	0.006429	5.363	1077	1.279885616	1.187	1.380	9.806	<0.0001
SOMA_P42830_CXCL5_2979_8	1867	0.828	0.765	0.898	0.006606	5.352	1077	1.047259627	0.945	1.161	0.420	0.3799
SOMA_P34096_RNASE4_5644_60	1867	1.278	1.151	1.419	0.006713	5.344	1077	1.901053546	1.685	2.145	24.727	<0.0001
SOMA_P0C7M6_IQCF3_13439_6	1867	0.8	0.728	0.88	0.006808	5.338	1077	0.197875277	0.107	0.365	6.659	<0.0001
SOMA_O14594_NCAN_15573_110	1867	0.806	0.735	0.884	0.007384	5.303	1077	0.81523861	0.696	0.955	1.946	0.0113
SOMA_O15525_MAFG_19281_86	1867	1.261	1.142	1.394	0.007585	5.291	1077	1.10021531	1.040	1.164	3.036	0.0009
SOMA_P49755_TMED10_6506_54	1867	1.29	1.156	1.439	0.00801	5.267	1077	1.227444577	1.150	1.310	9.181	<0.0001
SOMA_P02765_AHSG_3581_53	1867	0.813	0.743	0.889	0.008048	5.265	1077	0.948045324	0.796	1.128	0.261	0.5484
SOMA_P19827_ITIH1_7955_195	1867	0.789	0.712	0.874	0.008448	5.244	1077	0.499157519	0.436	0.571	23.349	<0.0001
SOMA_Q9BXJ1_C1QTNF1_6304_8	1867	1.274	1.148	1.415	0.008608	5.236	1077	1.455343935	1.357	1.561	24.894	<0.0001
SOMA_Q96EE4_CCDC126_6388_21	1867	0.797	0.723	0.88	0.009064	5.214	1077	0.284491896	0.140	0.577	3.312	0.0005
SOMA_Q68G75_LEMD1_8040_9	1867	1.251	1.135	1.379	0.00938	5.199	1077	1.085527212	0.979	1.204	0.921	0.12

SOMA_P18065_IGFBP2_8469_41	1867	1.325	1.173	1.497	0.009447	5.196	1077	1.778671298	1.619	1.954	32.417	<0.0001
SOMA_P52848_NDST1_6927_7	1867	0.809	0.737	0.887	0.009493	5.193	1077	0.723415652	0.617	0.848	4.168	<0.0001
SOMA_O60258_FGF17_3494_71	1867	0.841	0.78	0.907	0.009576	5.19	1077	0.946640739	0.774	1.158	0.227	0.5936
SOMA_P18859_ATP5J_7788_1	1867	1.28	1.15	1.426	0.00961	5.188	NA	NA	NA	NA	NA	NA
SOMA_P16112_ACAN_3280_49	1867	0.808	0.736	0.886	0.009891	5.176	1077	0.735615012	0.575	0.942	1.827	0.0149
SOMA_P41271_NBL1_2944_66	1867	1.293	1.156	1.446	0.009919	5.174	1077	1.215822982	1.146	1.290	10.116	<0.0001
SOMA_Q8TDQ0_HAVCR2_5134_52	1867	1.272	1.145	1.412	0.01036	5.155	1077	1.552663666	1.434	1.681	26.833	<0.0001
SOMA_Q14116_IL18_5661_15	1867	1.268	1.143	1.406	0.01049	5.15	1077	1.117778651	1.039	1.203	2.527	0.003
SOMA_O75339_CILP_5717_2	1867	1.257	1.137	1.388	0.01083	5.136	1077	1.096148985	0.955	1.259	0.715	0.1928
SOMA_Q99784_OLFM1_5703_26	1867	1.277	1.148	1.422	0.01118	5.122	1077	1.070613274	1.003	1.143	1.393	0.0405
SOMA_Q9HC57_WFDC1_9316_67	1867	1.285	1.152	1.435	0.0112	5.121	1077	1.508905111	1.390	1.638	22.045	<0.0001
SOMA_P45379_TNNT2_5315_22	1867	1.277	1.147	1.421	0.01132	5.117	1077	1.20104972	1.127	1.280	7.694	<0.0001
SOMA_O14786_NRP1_5542_22	1867	1.301	1.159	1.46	0.01143	5.112	1077	1.182114449	1.124	1.243	10.127	<0.0001
SOMA_Q2I0M5_RSPO4_8464_31	1867	1.309	1.163	1.473	0.01195	5.093	1077	1.175664354	1.108	1.247	7.106	<0.0001
SOMA_P84090_ERH_11614_29	1867	1.251	1.134	1.381	0.01247	5.074	1077	1.079256615	0.966	1.206	0.749	0.1784
SOMA_P61978_HNRNPK_19333_4	1867	1.248	1.132	1.376	0.01264	5.068	1077	0.964689147	0.834	1.116	0.202	0.6286
SOMA_P10721_KIT_2475_1	1867	0.795	0.719	0.88	0.01292	5.059	1077	0.629383013	0.532	0.744	7.214	<0.0001
SOMA_Q9HCE7_SMURF1_11557_3	1867	1.238	1.127	1.361	0.01362	5.036	1077	1.036266023	0.945	1.137	0.346	0.4507
SOMA_Q9GZV9_FGF23_3807_1	1867	1.259	1.137	1.393	0.01418	5.018	1077	1.401165188	1.310	1.499	22.067	<0.0001

SOMA_P11597_CETP_7131_207	1867	1.272	1.143	1.415	0.01429	5.015	NA	NA	NA	NA	NA	NA
SOMA_Q16842_ST3GAL2_6281_51	1867	1.245	1.13	1.372	0.01428	5.015	1077	1.063278733	0.944	1.198	0.506	0.3118
SOMA_Q6UWE3_CLPSL2_7767_1	1867	0.81	0.737	0.889	0.01496	4.995	1077	0.491921018	0.328	0.739	3.203	0.0006
SOMA_O95336_PGLS_17799_9	1867	1.24	1.127	1.364	0.0152	4.988	1077	0.890606247	0.756	1.049	0.781	0.1655
SOMA_P60842_EIF4A1_18829_4	1867	1.254	1.134	1.386	0.01568	4.974	1077	1.145242072	1.003	1.308	1.343	0.0453
SOMA_P02654_APOC1_15364_101	1867	0.806	0.732	0.887	0.01574	4.973	1077	0.770065317	0.665	0.892	3.290	0.0005
SOMA_Q92804_TAF15_16865_62	1867	1.246	1.13	1.375	0.01594	4.967	1077	1.061756752	0.923	1.222	0.395	0.403
SOMA_Q8IUB2_WFDC3_6384_19	1867	1.229	1.121	1.347	0.01611	4.962	1077	0.82215506	0.701	0.964	1.805	0.0157
SOMA_A6NI73_LILRA5_7787_25	1867	1.224	1.118	1.339	0.01657	4.95	1077	1.341890348	1.204	1.495	6.981	<0.0001
SOMA_P03950_ANG_4874_3	1867	1.242	1.127	1.368	0.01674	4.946	1077	1.204012822	1.055	1.374	2.237	0.0058
SOMA_P08319_ADH4_8325_37	1867	0.808	0.735	0.889	0.0174	4.928	1077	0.862495498	0.713	1.043	0.897	0.1269
SOMA_Q9BQI7_PSD2_9118_7	1867	0.804	0.729	0.886	0.01742	4.928	1077	0.575330112	0.360	0.920	1.677	0.021
SOMA_P01563_IFNA2_3497_13	1867	0.811	0.739	0.891	0.01749	4.926	1077	0.832073721	0.667	1.038	0.987	0.103
SOMA_P20333_TNFRSF1_3152_57	1867	1.269	1.141	1.412	0.01803	4.913	1077	1.084704523	1.033	1.139	2.945	0.0011
SOMA_P14649_MYL6B_14227_21	1867	1.253	1.133	1.386	0.01806	4.912	1077	1.20313164	1.126	1.286	7.355	<0.0001
SOMA_P19429_TNNI3_5441_67	1867	1.25	1.131	1.382	0.01897	4.891	1077	1.038230769	0.943	1.142	0.354	0.4422
SOMA_Q96GP6_SCARF2_8956_96	1867	1.322	1.166	1.5	0.01991	4.869	1077	1.550144749	1.435	1.674	28.199	<0.0001
SOMA_Q9H4F8_SMOC1_13118_5	1867	1.267	1.139	1.409	0.02002	4.867	1077	1.346082018	1.236	1.466	11.078	<0.0001
SOMA_P20333_TNFRSF1_8368_102	1867	1.27	1.14	1.414	0.0201	4.865	1077	1.173892507	1.120	1.230	10.746	<0.0001

SOMA_Q8WUE5_CT55_9363_11	1867	0.844	0.782	0.911	0.02053	4.856	1077	1.041076837	0.943	1.149	0.373	0.4241
SOMD P02671 FGA FGB 2796 62 PASS	1867	1.22	1.115	1.336	0.02218	4.822	NA	NA	NA	NA	NA	NA
SOMD P02675 FGA FGB 2796 62 PASS	1867	1.22	1.115	1.336	0.02218	4.822	NA	NA	NA	NA	NA	NA
SOMD P02679 FGA FGB 2796 62 PASS	1867	1.22	1.115	1.336	0.02218	4.822	NA	NA	NA	NA	NA	NA
SOMA_O14907_TAX1BP3_12498_12	1867	1.246	1.128	1.377	0.02298	4.807	1077	1.066089531	0.926	1.228	0.426	0.3746
SOMA_Q15485_FCN2_3313_21	1867	0.81	0.736	0.892	0.024	4.787	1077	0.995853873	0.856	1.159	0.019	0.9571
SOMA_Q96A72_MAGOHB_16875_13	1867	1.219	1.114	1.333	0.02475	4.774	1077	0.882797047	0.680	1.146	0.458	0.3486
SOMA_Q99439_CNN2_18877_15	1867	1.251	1.13	1.386	0.02586	4.755	1077	1.003140158	0.869	1.157	0.015	0.9657
SOMA_Q9H3U7_SMOC2_15635_4	1867	1.247	1.128	1.38	0.02626	4.748	1077	1.296096608	1.215	1.383	14.469	<0.0001
SOMA_Q9HAV5_EDA2R_3083_71	1867	1.255	1.131	1.392	0.02696	4.736	1077	1.10611239	1.037	1.180	2.676	0.0021
SOMA_Q86VZ4_LRP11_6713_4	1867	1.237	1.122	1.363	0.0274	4.729	1077	1.335054603	1.230	1.449	11.367	<0.0001
SOMA_Q9UBC7_GALP_9398_30	1867	0.842	0.778	0.911	0.02784	4.722	1077	0.096675578	0.022	0.421	2.729	0.0019
SOMA_O75056_SDC3_16612_28	1867	0.799	0.721	0.886	0.02862	4.71	1077	0.233328696	0.170	0.321	18.474	<0.0001
SOMA_P26441_CNTF_3489_9	1867	0.843	0.78	0.912	0.02863	4.71	1077	0.715431733	0.452	1.134	0.813	0.1538
SOMA_Q8IZP7_HS6ST3_18896_23	1867	0.811	0.737	0.893	0.02898	4.704	1077	0.765177872	0.629	0.931	2.127	0.0075
SOMA_Q155Q3_DIXDC1_13441_30	1867	0.815	0.742	0.895	0.02921	4.701	1077	0.969271521	0.818	1.148	0.144	0.7175
SOMA_P42081_CD86_5337_64	1867	0.822	0.751	0.9	0.03029	4.685	1077	0.321112451	0.035	2.955	0.501	0.3158
SOMA_O75462_CRLF1_14747_9	1867	1.243	1.125	1.375	0.031	4.675	1077	1.076745991	0.995	1.166	1.169	0.0677
SOMA_Q07820_MCL1_10396_6	1867	1.237	1.121	1.365	0.03326	4.644	1077	1.08488212	0.995	1.183	1.184	0.0655

SOMA_P31415_CASQ1_11263_57	1867	0.798	0.719	0.886	0.03437	4.629	1077	0.535377839	0.435	0.660	8.362	<0.0001
SOMA_Q96KN2_CNDP1_5456_59	1867	0.79	0.708	0.881	0.03456	4.627	1077	0.534212193	0.451	0.633	12.458	<0.0001
SOMA_P00325_ADH1B_9834_62	1867	0.819	0.746	0.898	0.03561	4.614	1077	1.041123674	0.916	1.184	0.269	0.538
SOMA_Q9BUD6_SPON2_8099_42	1867	1.262	1.133	1.407	0.03581	4.611	1077	1.760788564	1.592	1.948	27.416	<0.0001
SOMA_Q6WN34_CHRDL2_6086_15	1867	1.247	1.126	1.382	0.03622	4.606	1077	1.430911632	1.304	1.570	13.448	<0.0001
SOMA_O60234_GMFG_13062_4	1867	1.246	1.125	1.381	0.03878	4.576	1077	0.973016756	0.823	1.150	0.126	0.7489
SOMA_Q9NX46_ADPRHL2_17332_3	1867	1.232	1.118	1.358	0.03958	4.567	NA	NA	NA	NA	NA	NA
SOMA_Q7LFX5_CHST15_4469_78	1867	1.247	1.125	1.382	0.04039	4.558	1077	1.625043398	1.483	1.780	24.750	<0.0001
SOMA_P41970_ELK3_5707_55	1867	1.227	1.115	1.349	0.04042	4.557	1077	0.822758485	0.705	0.961	1.867	0.0136
SOMA_P09211_GSTP1_4911_49	1867	1.264	1.133	1.41	0.04088	4.552	1077	1.136056552	1.000	1.291	1.301	0.05
SOMA_Q8WXI7_MUC16_15565_102	1867	1.252	1.127	1.39	0.04171	4.543	1077	1.062750721	1.002	1.127	1.381	0.0416
SOMA_P22626_HNRNPA2_5351_52	1867	1.219	1.111	1.338	0.04183	4.542	1077	1.032153742	0.903	1.180	0.191	0.644
SOMA_Q13045_FLII_12677_164	1867	1.229	1.116	1.354	0.04195	4.541	1077	0.67899461	0.552	0.835	3.610	0.0002
SOMA_Q13277_STX3_7186_111	1867	1.231	1.117	1.358	0.04524	4.507	1077	1.049296152	0.944	1.166	0.429	0.3725
SOMA_P98172_EFNB1_13104_32	1867	1.244	1.123	1.379	0.04579	4.502	1077	1.200304952	1.127	1.278	7.856	<0.0001
SOMA_P04264_KRT1_9931_20	1867	1.239	1.12	1.37	0.0469	4.491	1077	1.258847524	1.186	1.336	13.555	<0.0001
SOMA_O60939_SCN2B_8353_15	1867	0.822	0.749	0.902	0.04781	4.483	1077	0.915712326	0.717	1.169	0.319	0.4796
SOMA_P05161_ISG15_14151_4	1867	1.226	1.113	1.349	0.04933	4.469	1077	1.12211103	1.010	1.246	1.505	0.0313
SOMA_P0C0P6_NPS_6390_18	1867	0.821	0.748	0.901	0.04999	4.463	1077	0.610830041	0.515	0.724	7.854	<0.0001

Table S11. Cis- Mendelian randomization estimates for the associations between genetically predited protein levels using pQTLs from the deCODE cohort and death as an outcome

UniProtID	Protein	aptamer_id	outcome	snps	ivw.OR	ivw.l	ivw.u	ivw.p	wm.OR	wm.l	wm.u	wm.p	egger.OR	egger.l	egger.u	egger.p	Egger.intercept.p	Q.p	I2	F.statistic	p.adjd
Q2I0M5	RSPO4	8464_31	Death	3.000	4.938	1.581	15.421	0.006	5.367	1.507	19.115	0.010	5.229	0.094	291.285	0.568	0.977	0.785	0.00	72.1	0.79
Q15485	FCN2	3313_21	Death	14.000	0.627	0.430	0.913	0.015	0.641	0.403	1.017	0.059	0.800	0.403	1.588	0.536	0.415	0.993	0.00	125.7	0.79
P15814	IGLL1	6485_59	Death	7.000	0.713	0.522	0.974	0.034	0.726	0.514	1.026	0.070	0.672	0.433	1.045	0.138	0.599	0.886	0.00	580.1	0.79
O60760	HPGDS	12549_33	Death	3.000	0.675	0.462	0.987	0.043	0.712	0.478	1.060	0.095	0.642	0.083	4.963	0.744	0.962	0.381	0.00	1112.1	0.79
Q9GZV9	FGF23	3807_1	Death	1.000	18.057	1.083	301.178	0.044	----	----	----	----	----	----	----	----	----	----	----	39.6	0.79
P12111	COL6A3	11196_31	Death	2.000	0.377	0.131	1.081	0.070	----	----	----	----	----	----	----	----	----	----	----	150.3	0.79
Q07654	TFF3	4721_54	Death	3.000	0.316	0.089	1.126	0.076	0.839	0.161	4.386	0.836	0.040	0.000	49.001	0.538	0.665	0.169	43.79	50.1	0.79
Q07654	TFF3	8323_163	Death	3.000	0.316	0.089	1.126	0.076	0.839	0.161	4.373	0.835	0.040	0.000	49.001	0.538	0.665	0.169	43.79	50.1	0.79
O95445	APOM	10445_20	Death	3.000	0.615	0.358	1.057	0.078	0.591	0.343	1.017	0.058	0.478	0.213	1.073	0.324	0.552	0.488	0.00	226.4	0.79
Q9HD15	SRA1	18220_141	Death	1.000	2.880	0.877	9.460	0.081	----	----	----	----	----	----	----	----	----	----	----	202.8	0.79
Q86VZ4	LRP11	6713_4	Death	5.000	1.236	0.973	1.568	0.082	1.259	1.020	1.556	0.032	1.516	0.988	2.325	0.153	0.335	0.273	22.23	2905.9	0.79
O00244	ATOX1	19233_75	Death	1.000	0.169	0.021	1.374	0.096	----	----	----	----	----	----	----	----	----	----	----	68.2	0.79
P05452	CLEC3B	5701_81	Death	2.000	0.531	0.248	1.139	0.104	----	----	----	----	----	----	----	----	----	----	----	249.5	0.79
P08319	ADH4	8325_37	Death	1.000	8.789	0.607	127.260	0.111	----	----	----	----	----	----	----	----	----	----	----	40.3	0.79
Q01638	IL1RL1	4234_8	Death	5.000	0.825	0.647	1.053	0.123	0.777	0.587	1.029	0.078	0.881	0.541	1.434	0.644	0.803	0.686	0.00	1798.9	0.79
Q6UX46	FAM150B	6284_7	Death	1.000	10.153	0.480	214.741	0.137	----	----	----	----	----	----	----	----	----	----	----	31.3	0.79

P07108	DBI	16919_1	Death	3.000	0.633	0.346	1.158	0.138	0.648	0.356	1.177	0.154	0.874	0.305	2.503	0.844	0.592	0.754	0.00	266.8	0.79
Q99784	OLFM1	5703_26	Death	5.000	0.552	0.248	1.229	0.146	0.357	0.132	0.965	0.042	0.373	0.061	2.269	0.363	0.689	0.475	0.00	90.8	0.79
Q9UBX5	FBLN5	15585_304	Death	2.000	2.755	0.694	10.944	0.150	----	----	----	----	----	----	----	----	----	----	----	65.0	0.79
A6NI73	LILRA5	7787_25	Death	7.000	1.393	0.887	2.186	0.150	1.382	0.853	2.241	0.189	1.177	0.552	2.512	0.691	0.559	0.651	0.00	184.9	0.79
P20333	TNFRSF1	3152_57	Death	3.000	0.624	0.327	1.192	0.153	0.708	0.361	1.388	0.314	0.852	0.349	2.077	0.784	0.520	0.414	0.00	161.8	0.79
P20333	TNFRSF1	8368_102	Death	3.000	0.624	0.327	1.192	0.153	0.708	0.368	1.362	0.300	0.852	0.349	2.077	0.784	0.520	0.414	0.00	161.8	0.79
Q93091	R----SE6	5646_20	Death	18.000	1.164	0.943	1.436	0.157	1.168	0.913	1.493	0.217	1.111	0.759	1.627	0.596	0.668	0.424	2.62	461.6	0.79
P43652	AFM	4763_31	Death	3.000	1.467	0.858	2.508	0.162	1.568	0.928	2.649	0.093	2.581	0.899	7.413	0.329	0.422	0.219	34.10	114.4	0.79
Q8IZS8	CAC----2D	8885_6	Death	13.000	1.361	0.880	2.104	0.166	1.359	0.771	2.397	0.289	0.738	0.302	1.801	0.518	0.142	0.472	0.00	117.9	0.79
P49773	HINT1	5900_11	Death	1.000	3.195	0.593	17.200	0.176	----	----	----	----	----	----	----	----	----	----	----	99.2	0.79
Q12805	EFEMP1	8480_29	Death	3.000	0.520	0.201	1.349	0.179	0.487	0.190	1.249	0.134	0.386	0.026	5.661	0.613	0.854	0.913	0.00	148.6	0.79
Q8TDY8	IGDCC4	9793_145	Death	4.000	0.706	0.424	1.178	0.183	0.596	0.336	1.059	0.078	0.492	0.149	1.622	0.364	0.572	0.457	0.00	230.6	0.79
Q16270	IGFBP7	3320_49	Death	6.000	1.355	0.863	2.128	0.186	1.432	0.891	2.301	0.138	1.309	0.436	3.928	0.656	0.906	0.385	4.91	253.3	0.79
Q86Z14	KLB	19557_3	Death	4.000	0.845	0.659	1.085	0.187	0.819	0.630	1.063	0.134	0.843	0.416	1.707	0.681	0.983	0.669	0.00	1883.4	0.79
Q9BX93	PLA2G12	9380_2	Death	2.000	0.389	0.095	1.589	0.189	----	----	----	----	----	----	----	----	----	----	----	75.2	0.79
P02765	AHSG	3581_53	Death	6.000	0.766	0.515	1.141	0.190	0.780	0.519	1.174	0.234	0.904	0.468	1.745	0.778	0.563	0.884	0.00	443.3	0.79
Q86TH1	ADAMTSL	6379_62	Death	5.000	0.535	0.209	1.367	0.191	0.694	0.237	2.029	0.504	0.861	0.035	21.303	0.933	0.794	0.550	0.00	71.5	0.79
Q96KN2	CNDP1	5456_59	Death	9.000	1.257	0.884	1.788	0.203	1.268	0.838	1.919	0.261	1.184	0.606	2.314	0.636	0.806	0.972	0.00	251.0	0.80
Q92626	PXDN	13463_1	Death	3.000	0.546	0.208	1.438	0.221	0.526	0.186	1.487	0.226	0.547	0.013	22.796	0.805	0.997	0.273	23.02	121.5	0.80

P03973	SLPI	4413_3	Death	1.000	2.249	0.614	8.236	0.221	----	----	----	----	----	----	----	----	----	----	----	174.0	0.80
Q14508	WFDC2	11388_75	Death	2.000	0.387	0.083	1.810	0.228	----	----	----	----	----	----	----	----	----	----	----	43.8	0.80
P51858	HDGF	16758_96	Death	1.000	3.473	0.433	27.852	0.241	----	----	----	----	----	----	----	----	----	----	----	74.0	0.80
P51858	HDGF	8953_47	Death	1.000	3.473	0.433	27.852	0.241	----	----	----	----	----	----	----	----	----	----	----	74.0	0.80
P19438	TNFRSF1	2654_19	Death	1.000	0.346	0.058	2.057	0.243	----	----	----	----	----	----	----	----	----	----	----	82.8	0.80
Q9HCB6	SPON1	4297_62	Death	6.000	1.315	0.829	2.085	0.245	1.314	0.808	2.136	0.271	1.507	0.603	3.767	0.430	0.793	0.900	0.00	245.5	0.80
P42081	CD86	5337_64	Death	1.000	0.234	0.020	2.797	0.251	----	----	----	----	----	----	----	----	----	----	----	51.3	0.80
P52848	NDST1	6927_7	Death	3.000	1.568	0.718	3.426	0.259	1.907	0.832	4.374	0.127	26.958	0.058	12571.078	0.484	0.528	0.220	33.96	139.3	0.80
P42830	CXCL5	2979_8	Death	1.000	1.706	0.661	4.399	0.269	----	----	----	----	----	----	----	----	----	----	----	327.9	0.80
Q8TDQ0	HAVCR2	5134_52	Death	5.000	1.245	0.843	1.839	0.271	1.269	0.837	1.923	0.262	1.649	0.790	3.443	0.275	0.448	0.695	0.00	378.6	0.80
Q06141	REG3A	15304_1	Death	10.000	1.323	0.801	2.185	0.274	0.996	0.540	1.835	0.989	1.910	0.951	3.835	0.106	0.165	0.185	28.19	147.6	0.80
P35442	THBS2	3339_33	Death	12.000	1.230	0.848	1.784	0.276	1.038	0.721	1.495	0.841	0.754	0.392	1.450	0.418	0.129	0.077	39.63	339.3	0.80
P17900	GM2A	15441_6	Death	6.000	0.789	0.504	1.234	0.300	0.887	0.552	1.427	0.621	0.874	0.407	1.875	0.747	0.748	0.685	0.00	258.4	0.83
P01011	SERPINA	4153_11	Death	9.000	0.793	0.508	1.240	0.309	0.821	0.485	1.388	0.461	0.896	0.432	1.857	0.776	0.661	0.994	0.00	167.1	0.83
P34096	RNASE4	5644_60	Death	9.000	1.228	0.826	1.825	0.311	1.613	1.048	2.484	0.030	1.918	1.017	3.619	0.084	0.143	0.372	7.61	214.6	0.83
O14594	NCAN	15573_110	Death	1.000	1.574	0.644	3.849	0.320	----	----	----	----	----	----	----	----	----	----	----	422.6	0.83
Q4LDE5	SVEP1	11109_56	Death	11.000	0.792	0.500	1.256	0.322	0.731	0.416	1.283	0.275	1.220	0.302	4.930	0.786	0.538	0.859	0.00	143.9	0.83
Q4LDE5	SVEP1	11178_21	Death	11.000	0.792	0.500	1.256	0.322	0.731	0.404	1.322	0.300	1.220	0.302	4.930	0.786	0.538	0.859	0.00	143.9	0.83
Q6WN34	CHRDL2	6086_15	Death	5.000	1.203	0.831	1.741	0.329	1.216	0.842	1.756	0.297	1.377	0.849	2.234	0.286	0.424	0.564	0.00	329.6	0.83

Q9P0K1	ADAM22	7933_75	Death	4.000	1.283	0.771	2.135	0.338	1.330	0.754	2.344	0.324	2.089	0.847	5.150	0.251	0.304	0.394	0.00	449.5	0.83
O14786	NRP1	5542_22	Death	5.000	1.242	0.793	1.946	0.344	1.102	0.694	1.750	0.680	0.847	0.384	1.870	0.709	0.329	0.420	0.00	275.8	0.83
P00747	PLG	3710_49	Death	5.000	0.749	0.411	1.365	0.345	0.717	0.378	1.358	0.307	0.478	0.181	1.262	0.233	0.331	0.469	0.00	194.9	0.83
P00533	EGFR	2677_1	Death	5.000	1.481	0.644	3.404	0.355	1.707	0.632	4.606	0.291	0.252	0.020	3.213	0.367	0.243	0.631	0.00	83.7	0.84
Q96DZ1	ERLEC1	8957_72	Death	1.000	2.193	0.371	12.963	0.386	----	----	----	----	----	----	----	----	----	----	----	84.2	0.89
P02654	APOC1	15364_101	Death	5.000	0.834	0.550	1.264	0.392	0.911	0.590	1.408	0.675	1.399	0.661	2.962	0.445	0.195	0.460	0.00	359.8	0.89
P02741	CRP	4337_49	Death	3.000	1.425	0.626	3.241	0.399	1.253	0.531	2.954	0.607	0.582	0.087	3.897	0.676	0.494	0.534	0.00	148.4	0.90
P05161	ISG15	14151_4	Death	2.000	1.265	0.711	2.251	0.423	----	----	----	----	----	----	----	----	----	----	----	466.5	0.91
O94856	NFASC	7179_69	Death	6.000	1.137	0.818	1.581	0.445	1.202	0.851	1.699	0.296	1.564	0.824	2.970	0.243	0.300	0.658	0.00	364.2	0.91
Q76LX8	ADAMTS1	3175_51	Death	12.000	0.890	0.658	1.204	0.450	0.889	0.627	1.262	0.512	0.813	0.479	1.379	0.461	0.627	0.417	2.78	478.0	0.91
Q9NUQ9	FAM49B	19176_27	Death	1.000	2.758	0.181	42.133	0.466	----	----	----	----	----	----	----	----	----	----	----	38.1	0.91
Q9Y274	ST3GAL6	6947_4	Death	5.000	1.139	0.802	1.617	0.468	1.113	0.739	1.676	0.608	1.124	0.549	2.300	0.770	0.976	0.784	0.00	608.9	0.91
P0DJI9	SAA2	18832_65	Death	7.000	0.883	0.627	1.244	0.476	0.888	0.639	1.235	0.481	1.011	0.608	1.681	0.968	0.546	0.313	15.38	382.2	0.91
Q92743	HTRA1	15594_47	Death	2.000	0.602	0.149	2.436	0.477	----	----	----	----	----	----	----	----	----	----	----	95.6	0.91
Q8IUL8	CILP2	8841_65	Death	2.000	1.613	0.429	6.067	0.479	----	----	----	----	----	----	----	----	----	----	----	71.2	0.91
Q6UXM1	LRIG3	3322_52	Death	5.000	0.865	0.576	1.301	0.487	0.835	0.539	1.294	0.420	0.619	0.268	1.430	0.343	0.440	0.642	0.00	278.0	0.91
Q02487	DSC2	13126_52	Death	3.000	0.765	0.358	1.634	0.488	0.778	0.358	1.690	0.525	0.885	0.158	4.967	0.912	0.866	0.526	0.00	156.4	0.91
Q99729	HNRNPAB	8894_80	Death	1.000	0.456	0.049	4.233	0.490	----	----	----	----	----	----	----	----	----	----	----	70.6	0.91
P01034	CST3	2609_59	Death	3.000	0.810	0.444	1.479	0.493	0.815	0.451	1.472	0.497	0.511	0.140	1.863	0.495	0.565	0.700	0.00	280.7	0.91

O14737	PDCD5	12517_52	Death	6.000	0.857	0.551	1.333	0.493	0.833	0.530	1.311	0.430	0.533	0.154	1.841	0.376	0.454	0.397	3.05	316.7	0.91
P09529	FGA FGB	13676_46	Death	5.000	0.835	0.495	1.408	0.498	0.935	0.579	1.509	0.782	1.897	0.755	4.766	0.266	0.139	0.242	26.89	258.2	0.91
P35858	IGFALS	6605_17	Death	5.000	0.649	0.180	2.340	0.508	0.516	0.186	1.431	0.203	1.121	0.034	36.590	0.953	0.801	0.051	57.64	90.9	0.91
O15123	ANGPT2	2602_2	Death	2.000	1.435	0.479	4.299	0.519	----	----	----	----	----	----	----	----	----	----	----	127.8	0.91
Q6P988	NOTUM	8252_2	Death	3.000	0.716	0.259	1.979	0.519	0.640	0.230	1.779	0.392	0.273	0.030	2.483	0.455	0.505	0.492	0.00	128.8	0.91
Q7LFX5	CHST15	4469_78	Death	2.000	1.455	0.451	4.692	0.530	----	----	----	----	----	----	----	----	----	----	----	98.1	0.91
P19827	ITIH1	7955_195	Death	3.000	1.111	0.800	1.542	0.531	1.085	0.787	1.496	0.619	0.838	0.493	1.425	0.632	0.405	0.296	17.77	1521.5	0.91
P18065	IGFBP2	8469_41	Death	1.000	0.437	0.029	6.542	0.549	----	----	----	----	----	----	----	----	----	----	----	36.5	0.92
P16860	NPPB	16751_15	Death	4.000	0.858	0.515	1.428	0.555	0.893	0.550	1.450	0.646	1.617	0.627	4.170	0.425	0.267	0.353	7.99	357.4	0.92
P16860	NPPB	7655_11	Death	4.000	0.858	0.515	1.428	0.555	0.893	0.544	1.464	0.653	1.617	0.627	4.170	0.425	0.267	0.353	7.99	357.4	0.92
Q99727	TIMP4	6462_12	Death	4.000	1.119	0.749	1.672	0.582	1.072	0.707	1.626	0.744	0.812	0.263	2.503	0.751	0.620	0.553	0.00	418.2	0.95
P07949	RET	3220_40	Death	7.000	1.116	0.748	1.665	0.592	1.057	0.654	1.710	0.820	1.015	0.370	2.784	0.978	0.854	0.343	11.35	257.5	0.95
Q9H3U7	SMOC2	15635_4	Death	9.000	0.895	0.592	1.355	0.601	0.975	0.613	1.550	0.915	1.333	0.575	3.089	0.524	0.322	0.911	0.00	213.6	0.96
Q9NP99	TREM1	9266_1	Death	7.000	1.104	0.758	1.609	0.607	1.068	0.688	1.658	0.769	1.054	0.516	2.155	0.891	0.882	0.634	0.00	285.6	0.96
Q07820	MCL1	10396_6	Death	1.000	0.696	0.168	2.884	0.617	----	----	----	----	----	----	----	----	----	----	----	65.6	0.96
P04264	KRT1	9931_20	Death	1.000	1.617	0.217	12.051	0.639	----	----	----	----	----	----	----	----	----	----	----	72.2	0.97
P40121	CAPG	4968_50	Death	4.000	0.872	0.490	1.554	0.642	0.865	0.483	1.549	0.625	0.936	0.325	2.690	0.913	0.877	0.929	0.00	234.6	0.97
P00797	REN	3396_54	Death	3.000	0.711	0.165	3.063	0.647	0.809	0.163	4.005	0.795	1.140	0.000	6604.212	0.981	0.932	0.914	0.00	41.4	0.97
Q96EE4	CCDC126	6388_21	Death	3.000	1.126	0.668	1.896	0.656	1.149	0.688	1.918	0.596	0.607	0.199	1.851	0.541	0.418	0.431	0.00	406.2	0.97

Q01974	ROR2	7861_9	Death	5.000	1.157	0.598	2.242	0.665	1.084	0.521	2.254	0.830	1.123	0.067	18.695	0.941	0.987	0.879	0.00	144.2	0.97
P39060	COL18A1	2201_17	Death	4.000	0.854	0.389	1.872	0.693	0.845	0.368	1.943	0.692	0.693	0.154	3.110	0.679	0.813	0.747	0.00	126.8	0.97
Q9NR71	ASAH2	3212_30	Death	5.000	0.934	0.657	1.329	0.706	0.925	0.698	1.224	0.584	1.049	0.623	1.767	0.867	0.589	0.239	27.35	951.6	0.97
Q9UNK0	STX8	10903_50	Death	1.000	0.619	0.051	7.542	0.707	----	----	----	----	----	----	----	----	----	----	----	49.2	0.97
Q8NBJ4	GOLM1	17456_53	Death	3.000	0.887	0.456	1.724	0.723	0.841	0.426	1.660	0.617	0.386	0.115	1.299	0.367	0.350	0.257	26.40	153.9	0.97
Q8IZP7	HS6ST3	18896_23	Death	1.000	1.274	0.308	5.269	0.738	----	----	----	----	----	----	----	----	----	----	----	133.0	0.97
Q99988	GDF15	4374_45	Death	8.000	0.905	0.499	1.642	0.743	1.104	0.645	1.892	0.718	1.684	0.659	4.303	0.318	0.148	0.051	49.95	232.1	0.97
Q9HC57	WFDC1	9316_67	Death	14.000	0.939	0.639	1.380	0.747	1.185	0.715	1.965	0.511	1.213	0.613	2.403	0.589	0.424	0.953	0.00	168.3	0.97
Q16627	CCL14	2900_53	Death	3.000	1.043	0.803	1.356	0.751	1.029	0.782	1.354	0.837	0.879	0.582	1.328	0.650	0.448	0.464	0.00	1240.7	0.97
Q9H4F8	SMOC1	13118_5	Death	5.000	0.918	0.539	1.563	0.752	1.037	0.578	1.862	0.902	1.359	0.338	5.473	0.695	0.592	0.430	0.00	182.0	0.97
P09211	GSTP1	4911_49	Death	3.000	0.852	0.315	2.304	0.753	0.833	0.296	2.339	0.728	2.021	0.022	183.715	0.811	0.759	0.301	16.77	112.8	0.97
P14555	PLA2G2A	2692_74	Death	12.000	1.050	0.773	1.427	0.753	0.922	0.726	1.172	0.509	0.905	0.576	1.422	0.676	0.414	0.017	52.56	974.1	0.97
Q4KMG0	CDON	4541_49	Death	7.000	1.061	0.728	1.546	0.760	1.071	0.765	1.500	0.689	1.126	0.680	1.864	0.664	0.659	0.324	13.93	489.4	0.97
O75056	SDC3	16612_28	Death	2.000	0.771	0.142	4.200	0.764	----	----	----	----	----	----	----	----	----	----	----	44.2	0.97
O60704	TPST2	8024_64	Death	4.000	0.901	0.405	2.005	0.798	0.951	0.431	2.100	0.901	1.181	0.220	6.340	0.864	0.776	0.707	0.00	107.2	0.97
Q8WXI7	MUC16	15565_102	Death	1.000	0.883	0.335	2.329	0.802	----	----	----	----	----	----	----	----	----	----	----	339.2	0.97
P08697	SERPINF	3024_18	Death	3.000	0.886	0.343	2.289	0.803	0.908	0.349	2.363	0.843	0.191	0.008	4.702	0.496	0.497	0.599	0.00	127.7	0.97
Q01995	TAGLN	15640_54	Death	2.000	1.174	0.328	4.196	0.805	----	----	----	----	----	----	----	----	----	----	----	51.2	0.97
O95336	PGLS	17799_9	Death	2.000	1.215	0.253	5.841	0.808	----	----	----	----	----	----	----	----	----	----	----	65.3	0.97

P61626	LYZ	4920_10	Death	9.000	0.951	0.631	1.432	0.809	0.757	0.531	1.078	0.122	0.674	0.280	1.621	0.408	0.422	0.122	37.08	468.6	0.97
P02679	FGG	4989_7	Death	1.000	1.132	0.408	3.144	0.812	----	----	----	----	----	----	----	----	----	----	----	240.6	0.97
P03950	ANG	4874_3	Death	12.000	1.037	0.769	1.398	0.813	1.050	0.731	1.508	0.792	1.125	0.638	1.981	0.693	0.699	0.832	0.00	274.2	0.97
Q9BXJ4	C1QTNF3	7251_64	Death	2.000	0.889	0.331	2.387	0.815	----	----	----	----	----	----	----	----	----	----	----	137.2	0.97
O95633	FSTL3	3438_10	Death	2.000	1.181	0.274	5.085	0.824	----	----	----	----	----	----	----	----	----	----	----	52.9	0.97
P0DJI8	SAA1	15515_2	Death	7.000	0.960	0.659	1.399	0.831	0.903	0.575	1.416	0.655	1.059	0.521	2.152	0.880	0.723	0.383	5.81	335.1	0.97
P16152	CBR1	12381_26	Death	4.000	0.899	0.334	2.423	0.833	1.038	0.351	3.069	0.946	1.002	0.054	18.428	0.999	0.949	0.252	26.59	71.8	0.97
O60911	CTSV	3364_76	Death	3.000	1.076	0.522	2.221	0.842	1.081	0.498	2.348	0.844	1.488	0.107	20.780	0.817	0.843	0.920	0.00	185.7	0.97
P41271	NBL1	2944_66	Death	2.000	1.171	0.159	8.637	0.877	----	----	----	----	----	----	----	----	----	----	----	39.7	0.98
Q9BQI0	AIF1L	18871_24	Death	9.000	0.978	0.709	1.349	0.890	0.959	0.682	1.347	0.807	1.043	0.639	1.703	0.872	0.742	0.928	0.00	422.3	0.98
P06276	BCHE	15514_26	Death	2.000	0.977	0.699	1.365	0.891	----	----	----	----	----	----	----	----	----	----	----	1920.7	0.98
Q9Y3E2	BOLA1	15370_5	Death	5.000	1.024	0.723	1.451	0.893	1.044	0.740	1.473	0.805	1.105	0.703	1.738	0.695	0.659	0.739	0.00	1166.7	0.98
Q9C005	DPY30	13943_38	Death	1.000	0.869	0.082	9.178	0.907	----	----	----	----	----	----	----	----	----	----	----	68.4	0.98
P16112	ACAN	3280_49	Death	3.000	0.952	0.310	2.926	0.932	0.964	0.305	3.047	0.951	1.129	0.068	18.812	0.946	0.914	0.962	0.00	93.4	0.98
Q9BUD6	SPON2	8099_42	Death	4.000	0.971	0.492	1.916	0.933	0.986	0.591	1.644	0.956	1.259	0.399	3.971	0.733	0.609	0.195	36.16	300.9	0.98
P58335	ANTXR2	15559_5	Death	5.000	0.959	0.343	2.680	0.936	0.906	0.434	1.890	0.793	0.145	0.036	0.581	0.072	0.057	0.039	60.41	129.6	0.98
P59901	LILRA4	8299_66	Death	10.000	1.014	0.702	1.466	0.939	0.971	0.623	1.513	0.896	1.103	0.608	2.002	0.756	0.875	0.605	0.00	225.6	0.98
O14907	TAX1BP3	12498_12	Death	1.000	1.056	0.224	4.981	0.946	----	----	----	----	----	----	----	----	----	----	----	119.9	0.98
Q9H4D0	CLSTN2	18882_7	Death	12.000	0.983	0.555	1.741	0.953	0.909	0.513	1.609	0.742	0.667	0.199	2.240	0.527	0.562	0.056	42.91	183.8	0.98

O75462	CRLF1	14747_9	Death	4.000	0.983	0.528	1.831	0.957	0.962	0.526	1.761	0.901	0.541	0.164	1.786	0.420	0.348	0.603	0.00	216.0	0.98
Q9GZX9	TWSG1	9234_8	Death	2.000	0.965	0.263	3.543	0.958	----	----	----	----	----	----	----	----	----	----	----	84.7	0.98
P07998	RNASE1	7211_2	Death	8.000	0.991	0.661	1.486	0.964	0.936	0.553	1.583	0.805	1.001	0.461	2.171	0.998	0.867	0.805	0.00	184.1	0.98
Q96GP6	SCARF2	8956_96	Death	1.000	1.026	0.288	3.650	0.969	----	----	----	----	----	----	----	----	----	----	----	248.6	0.98
Q9UJJ9	GNPTG	10666_7	Death	8.000	0.988	0.549	1.778	0.969	0.913	0.533	1.564	0.740	0.686	0.343	1.372	0.328	0.159	0.175	31.69	188.4	0.98
P52823	STC1	4930_21	Death	2.000	1.022	0.286	3.645	0.973	----	----	----	----	----	----	----	----	----	----	----	77.3	0.98
P08949	NMB	9321_400	Death	2.000	0.999	0.371	2.692	0.999	----	----	----	----	----	----	----	----	----	----	----	153.0	1.00

Table S12. Cis- Mendelian randomization estimates for the associations between genetically predited protein levels using pQTLs from the Fenland cohort and death as an outcome

UniProtID	Protein	SomaScan.id	outcome	snps	ivw.OR	ivw.l	ivw.u	ivw.p	wm.OR	wm.l	wm.u	wm.p	egger.OR	egger.l	egger.u	egger.p	Egger.intercept.p	Q.p	I2	F.statistic	p.adjd
Q12805	EFEMP1	SeqId_8480_29	Death	2	0.489	0.285	0.839	0.009	----	----	----	----	----	----	----	----	----	----	----	221.764	0.670
P52823	STC1	SeqId_4930_21	Death	1	4.788	1.416	16.191	0.012	----	----	----	----	----	----	----	----	----	----	----	89.114	0.670
O00244	ATOX1	SeqId_19233_75	Death	1	0.215	0.052	0.897	0.035	----	----	----	----	----	----	----	----	----	----	----	53.402	0.726
Q15485	FCN2	SeqId_3313_21	Death	4	0.768	0.600	0.983	0.036	0.752	0.575	0.984	0.038	0.581	0.273	1.240	0.296	0.526	0.901	0.000	413.043	0.726
Q4LDE5	SVEP1	SeqId_11109_56	Death	3	0.504	0.262	0.972	0.041	0.495	0.237	1.034	0.061	0.445	0.043	4.568	0.619	0.930	0.977	0.000	92.583	0.726
P03950	ANG	SeqId_4874_3	Death	2	1.230	1.003	1.510	0.047	----	----	----	----	----	----	----	----	----	----	----	1480.807	0.726
P34096	RNASE4	SeqId_5644_60	Death	2	1.438	0.996	2.076	0.053	----	----	----	----	----	----	----	----	----	----	----	411.426	0.726
P61626	LYZ	SeqId_4920_10	Death	2	0.824	0.672	1.010	0.063	----	----	----	----	----	----	----	----	----	----	----	1599.356	0.726
Q96KN2	CNDP1	SeqId_5456_59	Death	5	1.274	0.983	1.650	0.067	1.203	0.871	1.662	0.262	1.305	0.675	2.524	0.486	0.941	0.743	0.000	301.114	0.726
P05452	CLEC3B	SeqId_5701_81	Death	2	0.611	0.359	1.041	0.070	----	----	----	----	----	----	----	----	----	----	----	182.252	0.726
Q9NP99	TREM1	SeqId_9266_1	Death	2	1.532	0.966	2.431	0.070	----	----	----	----	----	----	----	----	----	----	----	290.487	0.726
Q8TDY8	IGDCC4	SeqId_9793_145	Death	1	0.617	0.361	1.054	0.077	----	----	----	----	----	----	----	----	----	----	----	289.000	0.734
Q8IZP7	HS6ST3	SeqId_18896_23	Death	1	3.061	0.783	11.972	0.108	----	----	----	----	----	----	----	----	----	----	----	60.989	0.768
Q9HD15	SRA1	SeqId_18220_141	Death	1	1.870	0.866	4.037	0.111	----	----	----	----	----	----	----	----	----	----	----	222.507	0.768
Q99729	HNRNPAB	SeqId_8894_80	Death	1	2.490	0.788	7.869	0.120	----	----	----	----	----	----	----	----	----	----	----	66.423	0.768
O14786	NRP1	SeqId_5542_22	Death	1	1.430	0.910	2.249	0.121	----	----	----	----	----	----	----	----	----	----	----	493.474	0.768

Q9HCB6	SPON1	SeqId_4297_62	Death	2	1.327	0.920	1.915	0.131	----	----	----	----	----	----	----	----	----	----	----	484.503	0.768
P15814	IGLL1	SeqId_6485_59	Death	1	0.724	0.474	1.107	0.136	----	----	----	----	----	----	----	----	----	----	----	459.674	0.768
Q4LDE5	SVEP1	SeqId_11178_21	Death	1	0.474	0.172	1.309	0.150	----	----	----	----	----	----	----	----	----	----	----	126.563	0.768
Q9Y5P4	COL4A3B	SeqId_13950_9	Death	1	2.172	0.736	6.408	0.160	----	----	----	----	----	----	----	----	----	----	----	100.911	0.768
Q92743	HTRA1	SeqId_15594_47	Death	2	0.564	0.253	1.260	0.163	----	----	----	----	----	----	----	----	----	----	----	89.057	0.768
P20333	TNFRSF1	SeqId_8368_102	Death	1	0.478	0.169	1.352	0.164	----	----	----	----	----	----	----	----	----	----	----	102.367	0.768
P07949	RET	SeqId_3220_40	Death	3	1.197	0.929	1.541	0.165	1.179	0.895	1.554	0.242	0.673	0.144	3.138	0.703	0.594	0.555	0.000	632.552	0.768
P17900	GM2A	SeqId_15441_6	Death	1	0.717	0.447	1.150	0.167	----	----	----	----	----	----	----	----	----	----	----	564.063	0.768
Q92626	PXDN	SeqId_13463_1	Death	1	0.533	0.203	1.397	0.200	----	----	----	----	----	----	----	----	----	----	----	115.976	0.768
P14555	PLA2G2A	SeqId_2692_74	Death	1	0.878	0.713	1.080	0.217	----	----	----	----	----	----	----	----	----	----	----	4830.250	0.768
Q9BQI0	AIF1L	SeqId_18871_24	Death	3	0.915	0.793	1.055	0.222	0.908	0.789	1.046	0.183	0.847	0.671	1.071	0.398	0.567	0.453	0.000	2869.876	0.768
P09529	FGA FGB	SeqId_13676_46	Death	1	0.739	0.453	1.204	0.225	----	----	----	----	----	----	----	----	----	----	----	539.669	0.768
Q7LFX5	CHST15	SeqId_4469_78	Death	1	1.833	0.681	4.933	0.230	----	----	----	----	----	----	----	----	----	----	----	97.516	0.768
P00747	PLG	SeqId_3710_49	Death	3	0.739	0.447	1.220	0.237	0.725	0.426	1.232	0.234	0.364	0.110	1.200	0.345	0.422	0.404	0.000	173.567	0.768
Q16270	IGFBP7	SeqId_3320_49	Death	1	1.267	0.855	1.876	0.238	----	----	----	----	----	----	----	----	----	----	----	722.266	0.768
P00533	EGFR	SeqId_2677_1	Death	2	1.685	0.685	4.145	0.256	----	----	----	----	----	----	----	----	----	----	----	57.822	0.768
Q02487	DSC2	SeqId_13126_52	Death	2	1.368	0.786	2.380	0.267	----	----	----	----	----	----	----	----	----	----	----	172.230	0.768
P07108	DBI	SeqId_16919_1	Death	1	0.755	0.459	1.241	0.267	----	----	----	----	----	----	----	----	----	----	----	560.111	0.768
P42830	CXCL5	SeqId_2979_8	Death	1	1.171	0.885	1.549	0.269	----	----	----	----	----	----	----	----	----	----	----	1239.781	0.768

P51858	HDGF	SeqId_8953_47	Death	1	2.584	0.469	14.252	0.276	----	----	----	----	----	----	----	----	----	----	----	49.000	0.768
Q96GP6	SCARF2	SeqId_8956_96	Death	2	1.374	0.769	2.456	0.284	----	----	----	----	----	----	----	----	----	----	----	233.582	0.768
O94856	NFASC	SeqId_7179_69	Death	1	1.164	0.881	1.539	0.286	----	----	----	----	----	----	----	----	----	----	----	1195.184	0.768
Q9P0K1	ADAM22	SeqId_7933_75	Death	2	1.289	0.808	2.055	0.286	----	----	----	----	----	----	----	----	----	----	----	278.595	0.768
Q2I0M5	RSPO4	SeqId_8464_31	Death	1	0.432	0.092	2.023	0.286	----	----	----	----	----	----	----	----	----	----	----	43.283	0.768
P08319	ADH4	SeqId_8325_37	Death	1	2.114	0.529	8.451	0.290	----	----	----	----	----	----	----	----	----	----	----	65.237	0.768
P51858	HDGF	SeqId_16758_96	Death	1	2.157	0.514	9.057	0.294	----	----	----	----	----	----	----	----	----	----	----	71.598	0.768
Q01638	IL1RL1	SeqId_4234_8	Death	2	0.911	0.763	1.087	0.301	----	----	----	----	----	----	----	----	----	----	----	2858.894	0.768
Q01995	TAGLN	SeqId_15640_54	Death	1	1.934	0.549	6.814	0.305	----	----	----	----	----	----	----	----	----	----	----	59.381	0.768
P06276	BCHE	SeqId_15514_26	Death	2	1.108	0.906	1.355	0.318	----	----	----	----	----	----	----	----	----	----	----	1595.384	0.768
O14594	NCAN	SeqId_15573_110	Death	1	1.695	0.599	4.793	0.320	----	----	----	----	----	----	----	----	----	----	----	155.707	0.768
Q86Z14	KLB	SeqId_19557_3	Death	1	0.888	0.702	1.123	0.321	----	----	----	----	----	----	----	----	----	----	----	2601.000	0.768
A6NI73	LILRA5	SeqId_7787_25	Death	2	1.425	0.706	2.877	0.324	----	----	----	----	----	----	----	----	----	----	----	517.770	0.768
Q06141	REG3A	SeqId_15304_1	Death	3	1.533	0.644	3.650	0.335	1.397	0.841	2.322	0.197	12.039	1.064	136.267	0.294	0.335	0.020	74.468	150.451	0.779
P01011	SERPINA	SeqId_4153_11	Death	3	0.845	0.594	1.200	0.346	0.846	0.586	1.221	0.371	0.966	0.571	1.636	0.919	0.623	0.798	0.000	336.270	0.790
P35858	IGFALS	SeqId_6605_17	Death	2	0.689	0.310	1.532	0.361	----	----	----	----	----	----	----	----	----	----	----	195.375	0.794
P08697	SERPINF	SeqId_3024_18	Death	2	0.819	0.533	1.258	0.362	----	----	----	----	----	----	----	----	----	----	----	286.951	0.794
Q9UBX5	FBLN5	SeqId_15585_304	Death	1	1.797	0.489	6.606	0.378	----	----	----	----	----	----	----	----	----	----	----	55.184	0.798
Q86VZ4	LRP11	SeqId_6713_4	Death	2	1.072	0.919	1.251	0.378	----	----	----	----	----	----	----	----	----	----	----	3199.686	0.798

P52848	NDST1	SeqId_6927_7	Death	1	1.406	0.639	3.094	0.397	----	----	----	----	----	----	----	----	----	----	----	202.515	0.824
O75462	CRLF1	SeqId_14747_9	Death	1	0.753	0.377	1.504	0.422	----	----	----	----	----	----	----	----	----	----	----	230.325	0.853
Q9HC57	WFDC1	SeqId_9316_67	Death	3	0.892	0.672	1.185	0.432	0.873	0.645	1.182	0.381	0.784	0.417	1.473	0.587	0.730	0.812	0.000	434.785	0.853
P07998	RNASE1	SeqId_7211_2	Death	1	1.320	0.659	2.647	0.434	----	----	----	----	----	----	----	----	----	----	----	233.653	0.853
O60911	CTSV	SeqId_3364_76	Death	2	0.797	0.439	1.448	0.457	----	----	----	----	----	----	----	----	----	----	----	146.728	0.883
Q8NBJ4	GOLM1	SeqId_17456_53	Death	2	0.835	0.483	1.442	0.517	----	----	----	----	----	----	----	----	----	----	----	151.661	0.982
Q4KMG0	CDON	SeqId_4541_49	Death	1	1.093	0.815	1.467	0.552	----	----	----	----	----	----	----	----	----	----	----	1677.098	0.992
Q6UXM1	LRIG3	SeqId_3322_52	Death	1	0.728	0.245	2.161	0.567	----	----	----	----	----	----	----	----	----	----	----	109.551	0.992
P49773	HINT1	SeqId_5900_11	Death	1	1.484	0.371	5.932	0.577	----	----	----	----	----	----	----	----	----	----	----	57.760	0.992
P02671	FGA FGB	SeqId_4907_56	Death	1	1.312	0.501	3.440	0.580	----	----	----	----	----	----	----	----	----	----	----	123.602	0.992
P02675	FGA FGB	SeqId_4907_56	Death	1	1.312	0.501	3.440	0.580	----	----	----	----	----	----	----	----	----	----	----	123.602	0.992
P02679	FGA FGB	SeqId_4907_56	Death	1	1.312	0.501	3.440	0.580	----	----	----	----	----	----	----	----	----	----	----	123.602	0.992
P02679	FGG	SeqId_4989_7	Death	1	1.324	0.489	3.582	0.580	----	----	----	----	----	----	----	----	----	----	----	115.879	0.992
P02671	FGA FGB	SeqId_2796_62	Death	1	1.326	0.488	3.607	0.580	----	----	----	----	----	----	----	----	----	----	----	114.616	0.992
P02675	FGA FGB	SeqId_2796_62	Death	1	1.326	0.488	3.607	0.580	----	----	----	----	----	----	----	----	----	----	----	114.616	0.992
P02679	FGA FGB	SeqId_2796_62	Death	1	1.326	0.488	3.607	0.580	----	----	----	----	----	----	----	----	----	----	----	114.616	0.992
P19827	ITIH1	SeqId_7955_195	Death	1	1.053	0.874	1.269	0.587	----	----	----	----	----	----	----	----	----	----	----	3906.250	0.992
P35442	THBS2	SeqId_3339_33	Death	2	1.075	0.825	1.402	0.592	----	----	----	----	----	----	----	----	----	----	----	841.976	0.992
Q9H3U7	SMOC2	SeqId_15635_4	Death	2	0.830	0.406	1.697	0.609	----	----	----	----	----	----	----	----	----	----	----	355.320	0.995

P01034	CST3	SeqId_2609_59	Death	2	0.926	0.652	1.316	0.669	----	----	----	----	----	----	----	----	----	----	----	455.652	0.995
Q86TH1	ADAMTSL	SeqId_6379_62	Death	1	0.760	0.190	3.042	0.698	----	----	----	----	----	----	----	----	----	----	----	49.000	0.995
P19823	ITIH2	SeqId_9326_33	Death	1	1.042	0.844	1.286	0.703	----	----	----	----	----	----	----	----	----	----	----	2300.162	0.995
Q93091	RNASE6	SeqId_5646_20	Death	2	1.025	0.901	1.167	0.706	----	----	----	----	----	----	----	----	----	----	----	5661.422	0.995
Q99727	TIMP4	SeqId_6462_12	Death	2	1.074	0.738	1.561	0.710	----	----	----	----	----	----	----	----	----	----	----	585.313	0.995
Q6P988	NOTUM	SeqId_8252_2	Death	1	0.879	0.432	1.787	0.722	----	----	----	----	----	----	----	----	----	----	----	136.819	0.995
Q9UJJ9	GNPTG	SeqId_10666_7	Death	1	0.946	0.687	1.304	0.736	----	----	----	----	----	----	----	----	----	----	----	1203.788	0.995
P39060	COL18A1	SeqId_2201_17	Death	2	1.086	0.667	1.768	0.740	----	----	----	----	----	----	----	----	----	----	----	201.736	0.995
Q9NR71	ASAH2	SeqId_3212_30	Death	2	1.112	0.582	2.125	0.748	----	----	----	----	----	----	----	----	----	----	----	964.240	0.995
Q9H4F8	SMOC1	SeqId_13118_5	Death	5	1.047	0.789	1.389	0.750	1.082	0.775	1.511	0.644	1.269	0.547	2.942	0.617	0.666	0.885	0.000	293.511	0.995
Q9H4D0	CLSTN2	SeqId_18882_7	Death	4	0.881	0.403	1.927	0.752	0.593	0.350	1.005	0.052	0.390	0.087	1.756	0.345	0.349	0.020	69.629	141.186	0.995
Q99784	OLFM1	SeqId_5703_26	Death	1	1.224	0.341	4.400	0.757	----	----	----	----	----	----	----	----	----	----	----	84.834	0.995
O60760	HPGDS	SeqId_12549_33	Death	1	1.048	0.754	1.457	0.780	----	----	----	----	----	----	----	----	----	----	----	1068.787	0.995
Q8TDQ0	HAVCR2	SeqId_5134_52	Death	1	1.032	0.808	1.319	0.801	----	----	----	----	----	----	----	----	----	----	----	2500.000	0.995
Q8WXI7	MUC16	SeqId_15565_102	Death	1	0.886	0.343	2.288	0.802	----	----	----	----	----	----	----	----	----	----	----	153.288	0.995
P59901	LILRA4	SeqId_8299_66	Death	4	0.955	0.661	1.381	0.808	1.016	0.732	1.409	0.926	1.148	0.430	3.063	0.809	0.722	0.160	41.909	371.362	0.995
P16860	NPPB	SeqId_7655_11	Death	1	1.075	0.600	1.925	0.809	----	----	----	----	----	----	----	----	----	----	----	332.361	0.995
Q16627	CCL14	SeqId_2900_53	Death	1	1.022	0.848	1.232	0.819	----	----	----	----	----	----	----	----	----	----	----	3428.753	0.995
Q9BUD6	SPON2	SeqId_8099_42	Death	1	1.054	0.652	1.705	0.831	----	----	----	----	----	----	----	----	----	----	----	554.059	0.995

P02741	CRP	SeqId_4337_49	Death	1	1.086	0.477	2.471	0.845	----	----	----	----	----	----	----	----	----	----	----	161.653	0.995
Q9Y274	ST3GAL6	SeqId_6947_4	Death	2	0.984	0.828	1.170	0.859	----	----	----	----	----	----	----	----	----	----	----	3257.992	0.995
Q76LX8	ADAMTS1	SeqId_3175_51	Death	1	0.976	0.737	1.293	0.866	----	----	----	----	----	----	----	----	----	----	----	1999.367	0.995
Q9BX93	PLA2G12	SeqId_9380_2	Death	1	0.915	0.307	2.723	0.873	----	----	----	----	----	----	----	----	----	----	----	70.141	0.995
Q9Y3E2	BOLA1	SeqId_15370_5	Death	1	0.982	0.786	1.228	0.874	----	----	----	----	----	----	----	----	----	----	----	3697.798	0.995
O14907	TAX1BP3	SeqId_12498_12	Death	1	0.928	0.363	2.373	0.876	----	----	----	----	----	----	----	----	----	----	----	124.408	0.995
Q9GZX9	TWSG1	SeqId_9234_8	Death	2	0.964	0.607	1.532	0.878	----	----	----	----	----	----	----	----	----	----	----	275.031	0.995
Q01974	ROR2	SeqId_7861_9	Death	2	1.057	0.502	2.228	0.883	----	----	----	----	----	----	----	----	----	----	----	105.896	0.995
Q9Y3E7	VPS24	SeqId_12508_9	Death	1	1.099	0.289	4.180	0.890	----	----	----	----	----	----	----	----	----	----	----	70.302	0.995
Q96EE4	CCDC126	SeqId_6388_21	Death	1	0.971	0.627	1.505	0.896	----	----	----	----	----	----	----	----	----	----	----	628.338	0.995
P58335	ANTXR2	SeqId_15559_5	Death	2	0.935	0.306	2.861	0.907	----	----	----	----	----	----	----	----	----	----	----	207.014	0.995
P03973	SLPI	SeqId_4413_3	Death	1	0.956	0.359	2.545	0.929	----	----	----	----	----	----	----	----	----	----	----	137.224	0.995
P02654	APOC1	SeqId_15364_101	Death	2	1.028	0.547	1.932	0.932	----	----	----	----	----	----	----	----	----	----	----	252.447	0.995
P12111	COL6A3	SeqId_11196_31	Death	1	1.057	0.284	3.943	0.934	----	----	----	----	----	----	----	----	----	----	----	82.291	0.995
O60704	TPST2	SeqId_8024_64	Death	1	1.022	0.587	1.778	0.938	----	----	----	----	----	----	----	----	----	----	----	355.178	0.995
Q9BXJ4	C1QTNF3	SeqId_7251_64	Death	1	1.026	0.518	2.035	0.940	----	----	----	----	----	----	----	----	----	----	----	223.424	0.995
Q6WN34	CHRDL2	SeqId_6086_15	Death	1	0.969	0.349	2.690	0.951	----	----	----	----	----	----	----	----	----	----	----	115.976	0.995
P0DJI8	SAA1	SeqId_15515_2	Death	2	0.987	0.615	1.582	0.956	----	----	----	----	----	----	----	----	----	----	----	648.834	0.995
O14737	PDCD5	SeqId_12517_52	Death	2	1.009	0.729	1.396	0.958	----	----	----	----	----	----	----	----	----	----	----	528.953	0.995

Q99988	GDF15	SeqId_4374_45	Death	2	0.988	0.329	2.970	0.983	----	----	----	----	----	----	----	----	----	----	----	538.517	0.995
P05161	ISG15	SeqId_14151_4	Death	2	1.003	0.739	1.361	0.983	----	----	----	----	----	----	----	----	----	----	----	1174.360	0.995
P0DJI9	SAA2	SeqId_18832_65	Death	2	0.995	0.588	1.682	0.984	----	----	----	----	----	----	----	----	----	----	----	531.883	0.995
P09211	GSTP1	SeqId_4911_49	Death	1	0.993	0.482	2.045	0.984	----	----	----	----	----	----	----	----	----	----	----	211.994	0.995
O15123	ANGPT2	SeqId_2602_2	Death	1	1.008	0.220	4.632	0.991	----	----	----	----	----	----	----	----	----	----	----	54.128	0.995
Q8IZS8	CACNA2D	SeqId_8885_6	Death	2	1.006	0.294	3.445	0.993	----	----	----	----	----	----	----	----	----	----	----	139.947	0.995
P16112	ACAN	SeqId_3280_49	Death	2	1.002	0.472	2.130	0.995	----	----	----	----	----	----	----	----	----	----	----	113.843	0.995

Table S13. Mendelian randomization estimates for the associations between genetically predited protein levels using pQTLs from the deCODE cohort and death as an outcome

UniProtID	Protein	aptamer_id	outcome	snps	ivw.OR	ivw.l	ivw.u	ivw.p	wm.OR	wm.l	wm.u	wm.p	egger.OR	egger.l	egger.u	egger.p	Egger.intercept.p	Q.p	I2	F.statistic	p.ajd
Q4LDE5	SVEP1	11109_56	Death	43	0.626	0.480	0.816	0.0005	0.689	0.467	1.016	0.060	0.831	0.467	1.478	0.532	0.352	0.978	0.00	84.63	0.12
Q2I0M5	RSPO4	8464_31	Death	6	3.717	1.492	9.260	0.0048	4.515	1.373	14.849	0.013	7.303	0.268	198.787	0.304	0.698	0.483	0.00	55.06	0.54
Q9HCB6	SPON1	4297_62	Death	15	1.642	1.104	2.444	0.0144	1.442	0.891	2.332	0.136	1.130	0.537	2.377	0.752	0.281	0.723	0.00	125.07	0.74
Q9UBX5	FBLN5	15585_304	Death	5	2.634	1.154	6.014	0.0215	3.064	1.192	7.880	0.020	14.674	0.730	294.921	0.178	0.334	0.792	0.00	85.81	0.74
A6NI73	LILRA5	7787_25	Death	20	1.445	1.054	1.981	0.0221	1.627	1.073	2.466	0.022	1.723	1.022	2.905	0.056	0.301	0.907	0.00	205.36	0.74
Q8WUE5	CT55	9363_11	Death	5	2.705	1.137	6.436	0.0244	3.173	1.240	8.116	0.016	3.541	0.115	108.638	0.522	0.880	0.932	0.00	89.43	0.74
P98172	EFNB1	13104_32	Death	1	0.262	0.079	0.867	0.0282	----	----	----	----	----	----	----	----	----	----	----	183.42	0.74
Q9BX93	PLA2G12	9380_2	Death	21	0.716	0.524	0.979	0.0362	0.695	0.458	1.056	0.088	0.606	0.360	1.020	0.075	0.500	0.838	0.00	168.95	0.74
Q9Y5H3	PCDHGA1	6321_65	Death	6	0.360	0.135	0.961	0.0413	0.324	0.094	1.114	0.074	0.048	0.003	0.907	0.113	0.240	0.435	0.00	51.10	0.74
P0C7M6	IQCF3	13439_6	Death	5	2.430	1.035	5.703	0.0413	2.760	1.072	7.106	0.035	6.506	0.445	95.013	0.265	0.496	0.828	0.00	91.07	0.74
Q9BQI7	PSD2	9118_7	Death	41	0.804	0.650	0.993	0.0428	0.647	0.476	0.878	0.005	0.613	0.429	0.875	0.010	0.135	0.908	0.00	176.30	0.74
Q9GZV9	FGF23	3807_1	Death	1	18.057	1.083	301.178	0.0439	----	----	----	----	----	----	----	----	----	----	----	39.60	0.74
P05413	FABP3	5437_63	Death	3	0.337	0.116	0.979	0.0455	0.281	0.084	0.940	0.039	0.627	0.009	43.519	0.865	0.819	0.535	0.00	80.74	0.74
Q14508	WFDC2	11388_75	Death	5	0.291	0.087	0.977	0.0458	0.312	0.075	1.295	0.109	2.006	0.019	213.163	0.789	0.465	0.281	20.93	43.31	0.74
P26441	CNTF	3489_9	Death	4	2.686	1.000	7.212	0.0499	3.679	1.276	10.608	0.016	10.051	0.431	234.208	0.287	0.474	0.691	0.00	84.16	0.75
P42830	CXCL5	2979_8	Death	7	1.828	0.985	3.393	0.0561	1.863	0.880	3.945	0.104	2.884	0.681	12.218	0.210	0.535	0.879	0.00	120.50	0.78

Q9HD15	SRA1	18220_141	Death	3	2.463	0.964	6.291	0.0596	2.439	0.877	6.787	0.088	1.637	0.047	57.576	0.831	0.836	0.460	0.00	105.10	0.78
Q12805	EFEMP1	8480_29	Death	6	0.439	0.182	1.055	0.0656	0.514	0.198	1.336	0.172	0.259	0.034	1.975	0.262	0.615	0.383	5.28	91.57	0.78
O14786	NRP1	5542_22	Death	17	1.347	0.972	1.868	0.0740	1.050	0.669	1.647	0.832	0.869	0.471	1.606	0.661	0.138	0.638	0.00	140.99	0.78
O14594	NCAN	15573_110	Death	17	1.520	0.957	2.414	0.0758	1.533	0.802	2.932	0.196	1.375	0.547	3.461	0.509	0.732	0.871	0.00	77.26	0.78
Q4LDE5	SVEP1	11178_21	Death	37	0.793	0.614	1.024	0.0759	0.910	0.621	1.336	0.632	0.946	0.593	1.508	0.816	0.475	0.864	0.00	98.87	0.78
Q92838	EDA	2826_53	Death	7	1.827	0.939	3.555	0.0761	2.633	1.220	5.684	0.014	4.491	0.895	22.529	0.128	0.282	0.809	0.00	100.97	0.78
Q13145	BAMBI	8811_24	Death	1	0.050	0.002	1.531	0.0863	----	----	----	----	----	----	----	----	----	----	----	32.28	0.80
Q8TDN1	KCNG4	13525_17	Death	11	1.550	0.928	2.590	0.0940	1.603	0.890	2.889	0.116	2.839	0.993	8.118	0.083	0.202	0.781	0.00	115.51	0.80
P01563	IFNA2	3497_13	Death	8	1.618	0.915	2.860	0.0980	2.162	1.106	4.225	0.024	5.910	1.407	24.822	0.051	0.096	0.646	0.00	128.08	0.80
P05452	CLEC3B	5701_81	Death	4	0.547	0.267	1.120	0.0988	0.542	0.254	1.155	0.113	0.483	0.125	1.873	0.403	0.823	0.821	0.00	144.61	0.80
P45379	TNNT2	5315_22	Death	4	2.238	0.853	5.869	0.1017	2.467	0.865	7.036	0.091	13.732	0.825	228.491	0.209	0.304	0.580	0.00	93.87	0.80
Q86Y30	BAGE2	6294_11	Death	3	0.278	0.060	1.298	0.1035	0.257	0.041	1.620	0.148	761.122	0.000	4199369282.663	0.556	0.497	0.254	27.12	43.03	0.80
Q86Y30	BAGE2	6294_11	Death	3	0.278	0.060	1.298	0.1035	0.257	0.041	1.623	0.148	761.122	0.000	4199369282.663	0.556	0.497	0.254	27.12	43.03	0.80
Q92626	PXDN	13463_1	Death	5	0.293	0.066	1.299	0.1062	0.408	0.152	1.098	0.076	1.859	0.085	40.712	0.720	0.299	0.024	64.27	86.94	0.80
P19438	TNFRSF1	2654_19	Death	8	0.483	0.196	1.192	0.1142	0.299	0.102	0.873	0.027	0.088	0.006	1.239	0.122	0.229	0.104	41.09	65.65	0.80
Q13291	SLAMF1	7953_20	Death	4	2.580	0.773	8.615	0.1234	1.947	0.557	6.809	0.297	0.332	0.001	122.433	0.750	0.562	0.836	0.00	58.83	0.80
Q86TH1	ADAMTSL	6379_62	Death	9	0.563	0.270	1.176	0.1263	0.743	0.305	1.807	0.512	1.315	0.100	17.263	0.841	0.531	0.830	0.00	65.79	0.80
P34096	RNASE4	5644_60	Death	13	1.324	0.924	1.896	0.1267	1.614	1.033	2.522	0.036	2.054	1.120	3.767	0.040	0.098	0.583	0.00	163.51	0.80
O60258	FGF17	3494_71	Death	6	1.900	0.832	4.337	0.1276	1.559	0.604	4.025	0.359	7.716	0.916	64.970	0.133	0.220	0.747	0.00	86.56	0.80

Q99439	CNN2	18877_15	Death	2	0.485	0.190	1.237	0.1299	----	----	----	----	----	----	----	----	----	----	----	175.46	0.80
P35442	THBS2	3339_33	Death	28	1.289	0.925	1.796	0.1334	1.042	0.710	1.529	0.833	1.042	0.615	1.764	0.880	0.486	0.028	36.68	181.38	0.80
Q99784	OLFM1	5703_26	Death	12	0.633	0.345	1.159	0.1380	0.413	0.184	0.925	0.032	0.823	0.208	3.257	0.787	0.712	0.384	6.30	73.17	0.80
Q9P0K1	ADAM22	7933_75	Death	20	1.298	0.917	1.837	0.1409	1.155	0.734	1.817	0.532	1.471	0.759	2.850	0.268	0.478	0.496	0.00	132.65	0.80
Q9HCE7	SMURF1	11557_3	Death	3	2.133	0.777	5.854	0.1414	1.812	0.601	5.459	0.291	1.028	0.027	39.438	0.991	0.733	0.237	30.61	96.70	0.80
P07108	DBI	16919_1	Death	6	0.644	0.353	1.176	0.1523	0.679	0.374	1.232	0.203	0.708	0.264	1.896	0.530	0.875	0.352	10.00	156.79	0.80
Q9GZX9	TWSG1	9234_8	Death	4	0.499	0.186	1.340	0.1680	0.532	0.177	1.596	0.260	0.424	0.070	2.591	0.451	0.858	0.992	0.00	62.51	0.80
Q8TDY8	IGDCC4	9793_145	Death	21	0.780	0.547	1.112	0.1692	0.648	0.400	1.049	0.078	0.758	0.400	1.437	0.406	0.837	0.849	0.00	86.41	0.80
Q01105	SET	5364_7	Death	3	0.422	0.122	1.455	0.1719	0.419	0.114	1.543	0.191	3.525	0.094	131.816	0.619	0.437	0.455	0.00	62.13	0.80
O00622	CYR61	6264_9	Death	3	0.284	0.046	1.741	0.1737	0.250	0.032	1.936	0.184	0.000	0.000	194.251	0.419	0.460	0.522	0.00	40.57	0.80
Q8TDQ0	HAVCR2	5134_52	Death	28	1.221	0.916	1.627	0.1742	1.191	0.809	1.752	0.376	1.107	0.659	1.858	0.705	0.779	0.907	0.00	126.04	0.80
P15814	IGLL1	6485_59	Death	41	0.824	0.624	1.089	0.1743	0.752	0.536	1.056	0.100	0.652	0.429	0.993	0.053	0.122	0.051	28.07	156.17	0.80
Q9BU40	CHRDL1	3362_61	Death	6	2.038	0.714	5.818	0.1833	1.361	0.333	5.552	0.668	0.003	0.000	10.746	0.239	0.194	0.483	0.00	41.43	0.80
P43652	AFM	4763_31	Death	10	1.458	0.835	2.544	0.1847	1.554	0.917	2.635	0.101	1.511	0.690	3.306	0.332	0.943	0.200	26.50	63.23	0.80
P59901	LILRA4	8299_66	Death	33	0.819	0.608	1.102	0.1877	0.926	0.606	1.414	0.721	0.904	0.525	1.555	0.717	0.517	0.190	17.51	110.12	0.80
O95990	FAM107A	2760_2	Death	9	1.548	0.804	2.982	0.1913	2.249	1.121	4.511	0.022	7.984	1.835	34.738	0.028	0.041	0.390	5.45	105.17	0.80
P19823	ITIH2	9326_33	Death	34	1.209	0.909	1.609	0.1921	1.271	0.920	1.756	0.145	1.174	0.716	1.926	0.529	0.986	0.032	33.47	123.47	0.80
O60704	TPST2	8024_64	Death	10	0.723	0.443	1.180	0.1940	0.694	0.381	1.265	0.233	0.781	0.310	1.967	0.615	0.907	0.982	0.00	105.09	0.80
Q6UXM1	LRIG3	3322_52	Death	49	1.147	0.931	1.412	0.1975	0.923	0.656	1.298	0.644	1.391	0.960	2.016	0.088	0.069	0.365	5.46	114.64	0.80

O00244	ATOX1	19233_75	Death	2	0.356	0.071	1.780	0.2086	----	----	----	----	----	----	----	----	----	----	----	55.46	0.80
Q07654	TFF3	4721_54	Death	18	0.751	0.479	1.176	0.2103	0.965	0.603	1.544	0.882	1.136	0.602	2.143	0.699	0.213	0.345	9.20	82.43	0.80
P01011	SERPINA	4153_11	Death	11	0.762	0.498	1.166	0.2110	0.822	0.487	1.386	0.462	0.964	0.484	1.923	0.920	0.425	0.994	0.00	144.95	0.80
P02765	AHSG	3581_53	Death	15	0.788	0.541	1.147	0.2135	0.784	0.521	1.180	0.243	0.824	0.476	1.428	0.503	0.811	0.898	0.00	206.48	0.80
Q5JXA9	SIRPB2	5669_26	Death	2	3.926	0.454	33.958	0.2140	----	----	----	----	----	----	----	----	----	----	----	34.24	0.80
Q9NR71	ASAH2	3212_30	Death	43	0.880	0.719	1.077	0.2165	0.949	0.718	1.254	0.713	0.900	0.659	1.229	0.510	0.987	0.692	0.00	171.63	0.80
P52848	NDST1	6927_7	Death	11	1.414	0.816	2.449	0.2169	1.842	0.994	3.414	0.052	1.940	0.496	7.591	0.366	0.701	0.152	30.92	91.34	0.80
Q6UWE3	CLPSL2	7767_1	Death	8	1.630	0.741	3.583	0.2242	2.462	1.072	5.654	0.034	13.375	2.102	85.098	0.033	0.046	0.304	16.05	89.44	0.81
P49773	HINT1	5900_11	Death	2	2.415	0.570	10.229	0.2314	----	----	----	----	----	----	----	----	----	----	----	69.79	0.81
O75475	PSIP1	17176_13	Death	1	3.872	0.403	37.242	0.2412	----	----	----	----	----	----	----	----	----	----	----	62.79	0.81
P51858	HDGF	16758_96	Death	1	3.427	0.437	26.887	0.2412	----	----	----	----	----	----	----	----	----	----	----	75.36	0.81
P51858	HDGF	8953_47	Death	1	3.473	0.433	27.852	0.2412	----	----	----	----	----	----	----	----	----	----	----	73.99	0.81
P62995	TRA2B	12373_73	Death	2	0.310	0.044	2.198	0.2415	----	----	----	----	----	----	----	----	----	----	----	45.17	0.81
P04264	KRT1	9931_20	Death	7	1.769	0.664	4.713	0.2542	1.655	0.484	5.660	0.422	33.197	0.363	3032.037	0.189	0.249	0.768	0.00	48.32	0.82
P01034	CST3	2609_59	Death	9	0.716	0.403	1.272	0.2546	0.778	0.419	1.443	0.425	0.793	0.311	2.022	0.642	0.828	0.396	4.75	116.41	0.82
P0C0P6	NPS	6390_18	Death	4	1.585	0.713	3.524	0.2588	1.385	0.578	3.319	0.465	1.536	0.226	10.443	0.704	0.958	0.439	0.00	89.37	0.82
P35858	IGFALS	6605_17	Death	15	0.800	0.540	1.185	0.2647	0.967	0.569	1.642	0.901	1.196	0.551	2.592	0.658	0.280	0.670	0.00	88.83	0.82
P08319	ADH4	8325_37	Death	6	1.852	0.626	5.477	0.2654	1.507	0.376	6.034	0.563	0.374	0.002	74.005	0.734	0.589	0.498	0.00	43.77	0.82
O60760	HPGDS	12549_33	Death	36	0.855	0.646	1.131	0.2728	0.711	0.492	1.027	0.069	0.748	0.465	1.201	0.238	0.662	0.318	8.85	142.14	0.82

Q06141	REG3A	15304_1	Death	23	1.224	0.853	1.758	0.2729	0.851	0.503	1.440	0.547	1.463	0.866	2.472	0.170	0.333	0.259	14.82	105.45	0.82
P0DJI8	SAA1	15515_2	Death	27	0.856	0.647	1.131	0.2735	0.713	0.475	1.070	0.103	1.059	0.648	1.731	0.822	0.282	0.816	0.00	141.60	0.82
P0DJI9	SAA2	18832_65	Death	27	0.869	0.664	1.138	0.3076	0.864	0.622	1.201	0.384	0.835	0.577	1.208	0.347	0.573	0.554	0.00	155.08	0.83
Q9NP99	TREM1	9266_1	Death	26	1.158	0.868	1.545	0.3196	0.989	0.640	1.529	0.962	1.001	0.575	1.743	0.998	0.607	0.823	0.00	111.14	0.83
Q9H3U7	SMOC2	15635_4	Death	20	0.836	0.587	1.192	0.3224	1.074	0.690	1.673	0.751	1.150	0.612	2.159	0.669	0.260	0.611	0.00	124.38	0.83
P15090	FABP4	15386_7	Death	2	0.497	0.123	2.003	0.3256	----	----	----	----	----	----	----	----	----	----	----	71.45	0.83
O95445	APOM	10445_20	Death	21	0.847	0.608	1.180	0.3264	0.800	0.521	1.227	0.306	0.748	0.464	1.207	0.248	0.693	0.998	0.00	83.03	0.83
O14907	TAX1BP3	12498_12	Death	6	1.601	0.600	4.273	0.3477	1.146	0.330	3.978	0.831	0.313	0.008	12.975	0.574	0.418	0.867	0.00	48.41	0.83
O95633	FSTL3	3438_10	Death	3	0.540	0.148	1.967	0.3502	1.035	0.205	5.224	0.966	362.221	0.002	84616686.045	0.522	0.484	0.077	60.91	48.57	0.83
P31415	CASQ1	11263_57	Death	4	0.569	0.174	1.862	0.3509	0.542	0.127	2.304	0.407	5.242	0.006	4261.991	0.676	0.576	0.624	0.00	51.96	0.83
P20333	TNFRSF1	8368_102	Death	14	0.753	0.413	1.370	0.3526	0.750	0.386	1.457	0.396	0.710	0.260	1.943	0.518	0.854	0.139	29.73	69.98	0.83
Q8WWZ8	OIT3	6296_36	Death	3	0.457	0.087	2.396	0.3546	0.357	0.035	3.639	0.385	0.061	0.000	65045.243	0.760	0.820	0.210	35.91	39.59	0.83
Q8WWZ8	OIT3	6296_36	Death	3	0.457	0.087	2.396	0.3546	0.357	0.037	3.479	0.375	0.061	0.000	65045.243	0.760	0.820	0.210	35.91	39.59	0.83
Q01844	EWSR1	12988_49	Death	1	2.981	0.293	30.312	0.3559	----	----	----	----	----	----	----	----	----	----	----	47.00	0.83
Q9UHV9	PFDN2	19243_2	Death	4	1.864	0.496	7.006	0.3569	1.140	0.225	5.782	0.874	776.595	1.166	517145.117	0.183	0.206	0.304	17.50	52.73	0.83
Q7LFX5	CHST15	4469_78	Death	16	1.206	0.806	1.804	0.3627	1.246	0.771	2.013	0.370	1.366	0.630	2.963	0.443	0.655	0.067	37.14	124.97	0.83
Q92743	HTRA1	15594_47	Death	8	0.695	0.317	1.527	0.3653	0.720	0.246	2.107	0.549	0.366	0.042	3.183	0.397	0.565	0.911	0.00	52.20	0.83
Q16270	IGFBP7	3320_49	Death	26	1.180	0.823	1.692	0.3669	1.431	0.888	2.306	0.141	1.746	0.916	3.329	0.104	0.185	0.628	0.00	99.61	0.83
Q01995	TAGLN	15640_54	Death	3	0.594	0.191	1.851	0.3694	1.053	0.282	3.927	0.939	9.212	0.661	128.476	0.347	0.269	0.068	62.85	50.01	0.83

P42167	TMPO	8265_225	Death	3	0.450	0.078	2.590	0.3710	0.531	0.076	3.730	0.524	146.801	0.000	2271432284.154	0.660	0.606	0.329	10.03	39.84	0.83
Q9BXJ1	C1QTNF1	6304_8	Death	17	0.846	0.585	1.222	0.3721	0.700	0.448	1.095	0.119	0.570	0.300	1.080	0.105	0.218	0.264	16.15	154.91	0.83
P29353	SHC1	5272_55	Death	3	1.819	0.485	6.827	0.3753	1.434	0.329	6.242	0.631	0.259	0.002	31.506	0.679	0.553	0.484	0.00	57.15	0.83
P61956	SUMO2	19555_1	Death	3	0.553	0.149	2.058	0.3770	0.619	0.149	2.581	0.511	33.831	0.095	12067.949	0.449	0.391	0.311	14.30	56.96	0.83
Q8IUL8	CILP2	8841_65	Death	6	1.568	0.578	4.257	0.3770	1.102	0.328	3.703	0.875	0.531	0.024	11.561	0.708	0.504	0.679	0.00	50.14	0.83
P08697	SERPINF	3024_18	Death	5	0.690	0.303	1.573	0.3772	0.725	0.282	1.867	0.505	1.240	0.222	6.911	0.822	0.541	0.631	0.00	91.98	0.83
P03950	ANG	4874_3	Death	24	1.127	0.861	1.476	0.3842	1.060	0.764	1.471	0.728	0.855	0.531	1.375	0.524	0.239	0.932	0.00	162.10	0.83
P00533	EGFR	2677_1	Death	9	1.357	0.681	2.705	0.3858	1.805	0.732	4.454	0.200	1.361	0.233	7.960	0.742	0.970	0.758	0.00	64.86	0.83
Q96DZ1	ERLEC1	8957_72	Death	1	2.193	0.371	12.963	0.3864	----	----	----	----	----	----	----	----	----	----	----	84.19	0.83
Q6P988	NOTUM	8252_2	Death	9	0.738	0.370	1.470	0.3870	0.687	0.303	1.557	0.368	0.684	0.161	2.903	0.623	0.911	0.788	0.00	80.77	0.83
Q8NBJ4	GOLM1	17456_53	Death	31	0.900	0.707	1.146	0.3939	0.814	0.572	1.157	0.251	0.728	0.501	1.058	0.107	0.078	0.560	0.00	146.22	0.83
P22223	CDH3	2643_57	Death	16	1.191	0.795	1.784	0.3959	1.312	0.786	2.189	0.299	1.769	0.846	3.701	0.152	0.187	0.903	0.00	108.24	0.83
P41970	ELK3	5707_55	Death	5	1.555	0.560	4.318	0.3966	0.765	0.210	2.787	0.685	2.069	0.043	99.785	0.738	0.870	0.377	5.17	62.13	0.83
P36776	LONP1	6398_12	Death	5	1.560	0.555	4.381	0.3989	0.758	0.228	2.526	0.652	1.941	0.048	78.914	0.749	0.877	0.354	9.17	60.58	0.83
O94856	NFASC	7179_69	Death	12	1.145	0.836	1.569	0.3991	1.203	0.853	1.696	0.291	1.212	0.792	1.857	0.397	0.684	0.721	0.00	205.01	0.83
Q8IUB2	WFDC3	6384_19	Death	5	1.560	0.554	4.396	0.4003	0.759	0.220	2.613	0.662	1.938	0.045	84.316	0.754	0.880	0.353	9.27	61.10	0.83
P16112	ACAN	3280_49	Death	7	1.398	0.636	3.073	0.4050	0.978	0.337	2.837	0.967	0.986	0.089	10.958	0.991	0.768	0.455	0.00	65.45	0.83
P41271	NBL1	2944_66	Death	8	1.842	0.436	7.779	0.4060	2.148	0.549	8.406	0.272	0.684	0.007	69.665	0.877	0.681	0.025	56.19	42.01	0.83
P17900	GM2A	15441_6	Death	10	0.807	0.475	1.372	0.4286	0.891	0.552	1.438	0.637	0.974	0.380	2.500	0.958	0.610	0.101	38.51	174.94	0.84

P61769	B2M	3485_28	Death	19	0.760	0.382	1.512	0.4347	0.801	0.417	1.539	0.506	1.135	0.312	4.128	0.850	0.497	0.485	0.00	90.21	0.84
Q9NRM6	IL17RB	6262_14	Death	5	1.529	0.526	4.445	0.4357	0.753	0.233	2.435	0.635	1.561	0.026	92.424	0.844	0.962	0.345	10.66	57.90	0.84
Q9Y3E2	BOLA1	15370_5	Death	34	1.107	0.855	1.433	0.4399	1.049	0.802	1.373	0.728	1.058	0.772	1.449	0.730	0.486	0.074	27.34	331.78	0.84
Q155Q3	DIXDC1	13441_30	Death	7	1.503	0.532	4.251	0.4420	1.353	0.386	4.743	0.637	4.689	0.146	151.032	0.423	0.545	0.770	0.00	43.31	0.84
Q6UX46	FAM150B	6284_7	Death	4	1.848	0.381	8.964	0.4457	1.351	0.214	8.546	0.749	0.000	0.000	14525.846	0.449	0.419	0.649	0.00	32.99	0.84
P02654	APOC1	15364_101	Death	12	0.864	0.593	1.259	0.4465	0.909	0.599	1.380	0.655	0.949	0.518	1.738	0.868	0.662	0.915	0.00	186.08	0.84
Q16842	ST3GAL2	6281_51	Death	2	0.439	0.052	3.676	0.4474	----	----	----	----	----	----	----	----	----	----	----	44.12	0.84
P67809	YBX1	9751_72	Death	9	1.244	0.708	2.187	0.4483	1.070	0.528	2.169	0.850	0.741	0.168	3.274	0.704	0.436	0.775	0.00	104.88	0.84
Q12906	ILF3	12759_47	Death	3	1.651	0.448	6.084	0.4510	2.359	0.512	10.870	0.271	3017.039	8.302	1096466.682	0.229	0.238	0.038	69.34	63.53	0.84
P09211	GSTP1	4911_49	Death	5	0.714	0.295	1.725	0.4539	0.758	0.287	2.003	0.576	2.426	0.020	298.425	0.742	0.636	0.369	6.56	90.98	0.84
P19827	ITIH1	7955_195	Death	52	0.912	0.717	1.161	0.4557	0.920	0.652	1.298	0.635	0.927	0.614	1.399	0.720	0.846	0.749	0.00	125.10	0.84
P18065	IGFBP2	8469_41	Death	5	0.661	0.221	1.977	0.4587	0.652	0.160	2.661	0.552	0.518	0.000	1802.638	0.884	0.960	0.828	0.00	38.64	0.84
Q9NX46	ADPRHL2	17332_3	Death	4	0.629	0.182	2.176	0.4645	0.507	0.130	1.980	0.329	0.336	0.026	4.347	0.492	0.643	0.952	0.00	47.15	0.84
P30050	RPL12	19183_164	Death	3	1.487	0.512	4.322	0.4660	1.291	0.430	3.878	0.649	0.310	0.017	5.617	0.574	0.443	0.267	24.23	91.07	0.84
Q99727	TIMP4	6462_12	Death	25	1.121	0.821	1.529	0.4727	1.046	0.684	1.599	0.835	1.083	0.648	1.809	0.764	0.832	0.954	0.00	111.02	0.84
P06276	BCHE	15514_26	Death	48	0.921	0.736	1.153	0.4749	0.962	0.700	1.324	0.814	0.966	0.668	1.396	0.853	0.627	0.590	0.00	142.62	0.84
P19429	TNNI3	5441_67	Death	1	0.367	0.023	5.940	0.4807	----	----	----	----	----	----	----	----	----	----	----	38.50	0.84
Q15485	FCN2	3313_21	Death	20	0.897	0.659	1.219	0.4863	0.842	0.545	1.300	0.438	0.807	0.476	1.369	0.437	0.702	0.567	0.00	120.92	0.84
O95336	PGLS	17799_9	Death	6	0.748	0.331	1.693	0.4863	0.685	0.248	1.895	0.466	0.556	0.129	2.392	0.475	0.653	0.916	0.00	49.80	0.84

P07998	RNASE1	7211_2	Death	11	0.859	0.548	1.346	0.5065	0.933	0.561	1.553	0.791	1.368	0.619	3.024	0.458	0.180	0.205	25.11	143.93	0.85
O14737	PDCD5	12517_52	Death	9	0.866	0.565	1.326	0.5076	0.855	0.528	1.384	0.523	0.797	0.380	1.671	0.567	0.749	0.425	1.04	227.12	0.85
P00747	PLG	3710_49	Death	22	0.872	0.581	1.308	0.5077	0.613	0.390	0.965	0.035	0.524	0.274	1.003	0.065	0.105	0.199	19.80	97.56	0.85
Q9H0R8	GABARAP	12661_44	Death	2	0.596	0.128	2.769	0.5088	----	----	----	----	----	----	----	----	----	----	----	61.32	0.85
Q9BQT9	CLSTN3	6291_55	Death	3	1.737	0.336	8.984	0.5099	1.756	0.239	12.908	0.580	1.185	0.000	71185187556855900.000	0.995	0.987	0.439	0.00	41.21	0.85
O95166	GABARAP	17735_130	Death	3	0.655	0.184	2.332	0.5133	0.661	0.178	2.460	0.537	1.283	0.001	1122.650	0.954	0.874	0.958	0.00	63.67	0.85
P15907	ST6GAL1	6035_2	Death	1	0.375	0.016	8.852	0.5430	----	----	----	----	----	----	----	----	----	----	----	29.90	0.88
Q9HAV5	EDA2R	3083_71	Death	1	0.642	0.154	2.674	0.5430	----	----	----	----	----	----	----	----	----	----	----	150.61	0.88
Q9BXJ4	C1QTNF3	7251_64	Death	7	0.771	0.332	1.794	0.5465	0.879	0.345	2.239	0.787	1.187	0.191	7.386	0.861	0.588	0.302	16.73	65.02	0.88
P49755	TMED10	6506_54	Death	18	0.908	0.660	1.250	0.5551	0.953	0.681	1.335	0.782	0.968	0.620	1.511	0.888	0.864	0.399	4.68	239.35	0.88
P51965	UBE2E1	14326_4	Death	5	1.241	0.598	2.577	0.5616	1.111	0.503	2.456	0.794	1.028	0.259	4.076	0.971	0.768	0.701	0.00	116.64	0.88
Q9NUQ9	FAM49B	19176_27	Death	2	1.847	0.232	14.711	0.5621	----	----	----	----	----	----	----	----	----	----	----	36.79	0.88
P39060	COL18A1	2201_17	Death	9	0.816	0.407	1.639	0.5684	0.863	0.410	1.816	0.698	0.829	0.165	4.154	0.826	0.920	0.236	23.27	84.86	0.88
Q16629	SRSF7	12987_12	Death	1	2.457	0.111	54.604	0.5699	----	----	----	----	----	----	----	----	----	----	----	36.20	0.88
P00325	ADH1B	9834_62	Death	1	2.555	0.100	64.932	0.5699	----	----	----	----	----	----	----	----	----	----	----	34.78	0.88
P61626	LYZ	4920_10	Death	36	1.076	0.834	1.389	0.5722	0.824	0.583	1.164	0.272	0.783	0.510	1.200	0.269	0.175	0.241	13.53	163.49	0.88
P08949	NMB	9321_400	Death	9	0.824	0.415	1.636	0.5796	0.980	0.431	2.228	0.962	0.607	0.091	4.064	0.623	0.771	0.485	0.00	84.94	0.88
Q6WN34	CHRDL2	6086_15	Death	17	1.098	0.786	1.535	0.5834	1.204	0.843	1.718	0.307	1.400	0.896	2.187	0.160	0.099	0.751	0.00	123.68	0.88
Q9HC57	WFDC1	9316_67	Death	29	0.921	0.686	1.237	0.5850	0.914	0.621	1.345	0.648	0.844	0.491	1.449	0.544	0.667	0.982	0.00	121.81	0.88

P16860	NPPB	16751_15	Death	1	1.510	0.330	6.920	0.5954	----	----	----	----	----	----	----	----	----	----	----	126.64	0.89
P00797	REN	3396_54	Death	6	0.734	0.231	2.332	0.5998	0.566	0.156	2.053	0.386	0.492	0.000	648.827	0.856	0.915	0.269	21.96	38.96	0.89
Q96GP6	SCARF2	8956_96	Death	5	1.230	0.564	2.683	0.6030	1.202	0.453	3.192	0.712	0.574	0.054	6.063	0.676	0.563	0.743	0.00	94.81	0.89
P16860	NPPB	7655_11	Death	7	0.876	0.524	1.464	0.6123	0.901	0.552	1.471	0.676	0.983	0.393	2.459	0.972	0.761	0.278	19.86	227.18	0.90
P83916	CBX1	18817_50	Death	2	1.465	0.331	6.480	0.6150	----	----	----	----	----	----	----	----	----	----	----	67.08	0.90
Q07820	MCL1	10396_6	Death	1	0.696	0.168	2.884	0.6174	----	----	----	----	----	----	----	----	----	----	----	65.60	0.90
Q9H4D0	CLSTN2	18882_7	Death	27	1.093	0.761	1.569	0.6321	0.917	0.555	1.516	0.736	0.872	0.409	1.856	0.725	0.609	0.268	13.33	107.55	0.91
Q99988	GDF15	4374_45	Death	19	0.908	0.607	1.359	0.6390	1.173	0.724	1.898	0.517	1.154	0.572	2.330	0.695	0.374	0.200	20.94	126.28	0.91
Q8WXI7	MUC16	15565_102	Death	17	0.914	0.625	1.336	0.6431	0.873	0.547	1.392	0.568	0.748	0.389	1.442	0.400	0.549	0.672	0.00	121.36	0.91
P18440	NAT1	12632_14	Death	14	0.907	0.600	1.371	0.6434	0.883	0.544	1.432	0.613	0.779	0.392	1.548	0.490	0.796	0.594	0.00	124.38	0.91
P03973	SLPI	4413_3	Death	7	1.195	0.554	2.576	0.6497	1.154	0.425	3.134	0.778	2.897	0.317	26.508	0.390	0.409	0.429	0.00	68.52	0.91
O60330	PCDHGA1	6938_21	Death	2	1.489	0.260	8.525	0.6547	----	----	----	----	----	----	----	----	----	----	----	47.83	0.91
Q9BQI0	AIF1L	18871_24	Death	20	1.068	0.797	1.432	0.6589	0.961	0.665	1.390	0.834	0.937	0.600	1.462	0.777	0.437	0.857	0.00	216.44	0.92
O15525	MAFG	19281_86	Death	3	1.372	0.315	5.973	0.6738	1.516	0.269	8.558	0.637	259.437	0.000	36422666994141200.000	0.795	0.805	0.902	0.00	45.90	0.93
P02741	CRP	4337_49	Death	18	0.918	0.614	1.372	0.6751	1.110	0.672	1.833	0.683	0.704	0.312	1.589	0.410	0.353	0.327	10.62	120.64	0.93
Q76LX8	ADAMTS1	3175_51	Death	52	1.045	0.844	1.295	0.6832	0.916	0.656	1.280	0.608	0.918	0.638	1.319	0.645	0.416	0.947	0.00	158.60	0.93
P05161	ISG15	14151_4	Death	9	1.162	0.560	2.410	0.6865	1.351	0.779	2.342	0.284	2.045	0.657	6.369	0.257	0.264	0.051	48.27	141.47	0.93
Q9C005	DPY30	13943_38	Death	6	0.802	0.271	2.370	0.6897	0.868	0.256	2.936	0.820	0.951	0.006	143.039	0.985	0.952	0.984	0.00	45.31	0.93
Q86Z14	KLB	19557_3	Death	51	0.961	0.782	1.180	0.7011	0.850	0.657	1.101	0.219	0.830	0.615	1.121	0.230	0.137	0.219	12.94	211.45	0.94

O60939	SCN2B	8353_15	Death	10	0.923	0.593	1.438	0.7233	1.000	0.660	1.516	0.999	1.105	0.591	2.069	0.762	0.487	0.130	34.71	191.11	0.95
Q86Y82	STX12	10418_36	Death	2	0.784	0.202	3.051	0.7261	----	----	----	----	----	----	----	----	----	----	----	78.47	0.95
Q13045	FLII	12677_164	Death	2	0.790	0.209	2.989	0.7282	----	----	----	----	----	----	----	----	----	----	----	81.95	0.95
Q9Y5P4	COL4A3B	13950_9	Death	3	0.772	0.175	3.401	0.7320	1.077	0.192	6.032	0.933	2.801	0.002	3559.820	0.825	0.777	0.521	0.00	48.45	0.95
Q96KN2	CNDP1	5456_59	Death	22	1.048	0.799	1.376	0.7341	0.963	0.673	1.378	0.836	0.843	0.540	1.318	0.463	0.509	0.723	0.00	194.13	0.95
Q9NZ72	STMN3	8019_73	Death	3	0.829	0.271	2.538	0.7425	0.781	0.216	2.822	0.706	14.806	0.000	5933723.320	0.753	0.736	0.908	0.00	70.67	0.96
Q8IZP7	HS6ST3	18896_23	Death	5	0.847	0.306	2.347	0.7500	1.200	0.358	4.030	0.768	2.241	0.089	56.221	0.657	0.559	0.395	1.93	59.62	0.96
Q9UKP5	ADAMTS6	6441_62	Death	1	0.605	0.024	15.010	0.7591	----	----	----	----	----	----	----	----	----	----	----	31.82	0.96
Q68G75	LEMD1	8040_9	Death	1	0.606	0.025	14.834	0.7591	----	----	----	----	----	----	----	----	----	----	----	32.32	0.96
Q02487	DSC2	13126_52	Death	9	0.870	0.355	2.130	0.7604	0.725	0.341	1.539	0.402	0.622	0.084	4.625	0.657	0.760	0.043	49.93	79.11	0.96
Q93091	RNASE6	5646_20	Death	41	0.971	0.798	1.180	0.7661	1.163	0.908	1.489	0.233	1.384	1.024	1.870	0.041	0.030	0.358	6.18	236.60	0.96
Q07960	ARHGAP1	11955_1	Death	4	0.853	0.280	2.597	0.7796	1.000	0.292	3.423	0.999	0.333	0.005	20.906	0.655	0.689	0.971	0.00	56.31	0.96
O75056	SDC3	16612_28	Death	10	1.107	0.541	2.266	0.7800	0.942	0.368	2.409	0.900	4.418	0.600	32.531	0.183	0.171	0.631	0.00	65.68	0.96
P10721	KIT	2475_1	Death	24	1.062	0.697	1.616	0.7800	1.168	0.644	2.118	0.610	1.493	0.543	4.109	0.446	0.504	0.314	10.63	79.81	0.96
P42081	CD86	5337_64	Death	5	0.798	0.163	3.895	0.7801	0.401	0.100	1.617	0.199	5.954	0.007	4928.166	0.639	0.580	0.081	51.85	60.78	0.96
P58335	ANTXR2	15559_5	Death	16	1.078	0.633	1.836	0.7818	0.796	0.406	1.559	0.505	0.294	0.099	0.875	0.045	0.022	0.165	25.66	83.38	0.96
O75339	CILP	5717_2	Death	2	1.329	0.156	11.348	0.7946	----	----	----	----	----	----	----	----	----	----	----	33.60	0.96
Q6ZTQ4	CDHR3	8222_49	Death	5	1.110	0.499	2.469	0.7977	1.015	0.464	2.219	0.970	0.575	0.197	1.678	0.386	0.211	0.249	25.86	98.65	0.96
O15123	ANGPT2	2602_2	Death	10	0.927	0.514	1.672	0.7999	0.947	0.441	2.033	0.889	0.486	0.139	1.699	0.291	0.287	0.827	0.00	73.73	0.96

O60234	GMFG	13062_4	Death	2	0.755	0.084	6.820	0.8022	----	----	----	----	----	----	----	----	----	----	----	33.34	0.96
Q92804	TAF15	16865_62	Death	2	1.289	0.174	9.572	0.8039	----	----	----	----	----	----	----	----	----	----	----	38.19	0.96
P09529	FGAFGB	13676_46	Death	13	0.950	0.609	1.480	0.8193	0.948	0.611	1.470	0.810	1.053	0.486	2.282	0.899	0.791	0.200	24.12	165.84	0.97
P20333	TNFRSF1	3152_57	Death	11	0.900	0.353	2.299	0.8263	0.741	0.285	1.925	0.539	0.675	0.103	4.445	0.693	0.765	0.049	45.51	52.62	0.97
P14555	PLA2G2A	2692_74	Death	40	0.979	0.807	1.188	0.8281	0.891	0.707	1.123	0.329	0.947	0.724	1.238	0.691	0.798	0.334	7.60	334.99	0.97
Q07654	TFF3	8323_163	Death	11	0.939	0.526	1.678	0.8322	1.072	0.600	1.916	0.814	1.270	0.528	3.054	0.606	0.483	0.943	0.00	83.58	0.97
P12111	COL6A3	11196_31	Death	3	0.846	0.174	4.113	0.8360	1.160	0.187	7.195	0.874	203.169	0.000	168567379.172	0.585	0.573	0.466	0.00	40.88	0.97
Q01974	ROR2	7861_9	Death	9	0.941	0.520	1.704	0.8407	0.991	0.511	1.921	0.979	2.387	0.320	17.786	0.424	0.367	0.648	0.00	104.21	0.97
Q16627	CCL14	2900_53	Death	14	1.024	0.806	1.302	0.8458	1.047	0.783	1.399	0.759	1.047	0.768	1.429	0.776	0.893	0.979	0.00	299.33	0.97
Q2UY09	COL28A1	10702_1	Death	5	0.889	0.263	2.999	0.8492	1.156	0.279	4.796	0.841	1.615	0.011	240.324	0.863	0.834	0.568	0.00	40.63	0.97
Q86VZ4	LRP11	6713_4	Death	66	0.981	0.796	1.209	0.8584	1.149	0.888	1.487	0.292	1.064	0.786	1.441	0.689	0.947	0.028	26.52	215.57	0.97
Q4KMG0	CDON	4541_49	Death	16	1.032	0.714	1.492	0.8677	1.065	0.764	1.484	0.710	1.019	0.625	1.660	0.942	0.992	0.090	34.04	234.10	0.97
Q99729	HNRNPAB	8894_80	Death	4	0.917	0.320	2.625	0.8711	0.916	0.248	3.384	0.895	0.326	0.014	7.815	0.561	0.560	0.830	0.00	64.85	0.97
Q9BUD6	SPON2	8099_42	Death	9	0.961	0.594	1.555	0.8714	0.990	0.585	1.677	0.971	0.971	0.448	2.105	0.944	0.941	0.732	0.00	161.36	0.97
Q9UJJ9	GNPTG	10666_7	Death	16	0.964	0.610	1.524	0.8757	0.845	0.505	1.415	0.523	0.852	0.432	1.682	0.652	0.653	0.261	16.86	114.13	0.97
Q96EE4	CCDC126	6388_21	Death	24	1.026	0.732	1.439	0.8800	1.010	0.652	1.566	0.963	0.941	0.536	1.655	0.836	0.501	0.640	0.00	118.72	0.97
Q8IZS8	CACNA2D	8885_6	Death	30	0.976	0.679	1.403	0.8957	0.893	0.553	1.444	0.645	0.734	0.385	1.396	0.353	0.352	0.288	11.47	97.67	0.97
P14649	MYL6B	14227_21	Death	3	1.076	0.343	3.377	0.9007	0.654	0.176	2.435	0.527	0.189	0.013	2.805	0.440	0.376	0.059	64.63	59.49	0.97
Q9UNK0	STX8	10903_50	Death	4	1.073	0.334	3.443	0.9063	0.694	0.167	2.875	0.614	0.061	0.000	10.200	0.397	0.379	0.473	0.00	52.54	0.97

Q93045	STMN2	10900_272	Death	4	1.055	0.415	2.682	0.9109	0.877	0.317	2.423	0.800	0.071	0.001	3.818	0.323	0.301	0.328	12.88	93.13	0.97
Q9BUJ0	ABHD14A	5715_4	Death	1	1.104	0.137	8.876	0.9258	----	----	----	----	----	----	----	----	----	----	----	69.87	0.97
P09651	HNRNPA1	12466_7	Death	1	1.074	0.241	4.785	0.9258	----	----	----	----	----	----	----	----	----	----	----	135.62	0.97
P60842	EIF4A1	18829_4	Death	1	1.095	0.163	7.364	0.9258	----	----	----	----	----	----	----	----	----	----	----	83.23	0.97
Q96A72	MAGOHB	16875_13	Death	1	1.159	0.052	26.069	0.9258	----	----	----	----	----	----	----	----	----	----	----	31.60	0.97
P22626	HNRNPA2	5351_52	Death	1	1.113	0.117	10.623	0.9258	----	----	----	----	----	----	----	----	----	----	----	57.44	0.97
Q9UBC7	GALP	9398_30	Death	1	1.169	0.041	33.134	0.9270	----	----	----	----	----	----	----	----	----	----	----	33.08	0.97
P40121	CAPG	4968_50	Death	16	1.020	0.665	1.563	0.9294	0.878	0.498	1.547	0.652	0.638	0.275	1.482	0.314	0.245	0.692	0.00	96.64	0.97
P02679	FGG	4989_7	Death	6	1.034	0.479	2.232	0.9322	1.037	0.430	2.501	0.936	1.281	0.227	7.217	0.793	0.820	0.799	0.00	76.21	0.97
O60911	CTSV	3364_76	Death	23	0.985	0.651	1.489	0.9425	1.430	0.803	2.548	0.224	2.319	0.783	6.868	0.144	0.109	0.660	0.00	76.38	0.97
Q9H4F8	SMOC1	13118_5	Death	9	1.025	0.512	2.051	0.9448	1.090	0.604	1.966	0.776	0.977	0.163	5.867	0.980	0.969	0.045	49.48	118.51	0.97
O75462	CRLF1	14747_9	Death	5	0.980	0.533	1.801	0.9473	0.962	0.526	1.759	0.900	0.746	0.265	2.100	0.617	0.512	0.711	0.00	182.28	0.97
P55957	BID	5798_3	Death	2	0.947	0.169	5.319	0.9511	----	----	----	----	----	----	----	----	----	----	----	39.31	0.97
P16152	CBR1	12381_26	Death	6	0.977	0.443	2.156	0.9543	1.770	0.605	5.183	0.297	0.916	0.112	7.530	0.939	0.925	0.490	0.00	59.62	0.97
P07949	RET	3220_40	Death	21	1.012	0.662	1.548	0.9555	1.020	0.637	1.631	0.935	0.774	0.359	1.665	0.519	0.700	0.039	37.10	129.62	0.97
Q9Y274	ST3GAL6	6947_4	Death	98	0.996	0.869	1.143	0.9597	0.960	0.806	1.143	0.647	1.015	0.865	1.192	0.854	0.567	0.427	1.95	360.00	0.97
Q9Y3E7	VPS24	12508_9	Death	3	1.016	0.208	4.968	0.9839	1.137	0.177	7.291	0.892	299.806	0.000	29024838500080300000.000	0.823	0.823	0.957	0.00	39.27	0.99
Q01638	IL1RL1	4234_8	Death	70	0.998	0.812	1.228	0.9886	0.812	0.628	1.049	0.111	0.813	0.616	1.073	0.148	0.065	0.243	10.14	199.29	0.99
P52823	STC1	4930_21	Death	9	1.005	0.508	1.987	0.9893	0.753	0.351	1.614	0.465	0.273	0.036	2.039	0.246	0.223	0.148	33.71	93.50	0.99

Table S14. Mendelian randomization estimates for the associations between genetically predited protein levels using pQTLs from the Fenland cohort and death as an outcome

UniProtID	Protein	SomaScan.id	outcome	snps	ivw.OR	ivw.l	ivw.u	ivw.p	wm.OR	wm.l	wm.u	wm.p	egger.OR	egger.l	egger.u	egger.p	Egger.intercept.p	Q.p	I2	F.statistic	p.adj
Q9BQI7	PSD2	SeqId_9118_7	Death	4	0.668	0.506	0.882	0.004	0.685	0.517	0.909	0.009	0.543	0.220	1.341	0.317	0.678	0.270	23.473	492.419	0.417
Q01105	SET	SeqId_5364_7	Death	4	0.443	0.236	0.833	0.011	0.477	0.230	0.989	0.047	0.340	0.015	7.975	0.572	0.883	0.706	0.000	65.275	0.417
Q4LDE5	SVEP1	SeqId_11109_56	Death	4	0.427	0.220	0.829	0.012	0.507	0.269	0.957	0.036	2.032	0.338	12.198	0.519	0.217	0.182	38.257	111.525	0.417
Q2I0M5	RSPO4	SeqId_8464_31	Death	2	0.295	0.105	0.830	0.021	----	----	----	----	----	----	----	----	----	----	----	51.822	0.417
Q9UBX5	FBLN5	SeqId_15585_304	Death	2	2.330	1.090	4.982	0.029	----	----	----	----	----	----	----	----	----	----	----	84.990	0.417
Q9Y5H3	PCDHGA1	SeqId_6321_65	Death	1	0.226	0.058	0.873	0.031	----	----	----	----	----	----	----	----	----	----	----	64.000	0.417
Q13145	BAMBI	SeqId_8811_24	Death	1	0.303	0.102	0.899	0.031	----	----	----	----	----	----	----	----	----	----	----	84.903	0.417
P19429	TNNI3	SeqId_5441_67	Death	1	0.283	0.089	0.893	0.031	----	----	----	----	----	----	----	----	----	----	----	75.939	0.417
Q13277	STX3	SeqId_7186_111	Death	1	0.300	0.100	0.898	0.031	----	----	----	----	----	----	----	----	----	----	----	83.592	0.417
Q8WWZ8	OIT3	SeqId_6296_36	Death	1	0.259	0.074	0.911	0.035	----	----	----	----	----	----	----	----	----	----	----	74.225	0.417
O60330	PCDHGA1	SeqId_6938_21	Death	1	0.268	0.077	0.929	0.038	----	----	----	----	----	----	----	----	----	----	----	65.148	0.417
P46108	CRK	SeqId_4976_57	Death	1	0.219	0.052	0.919	0.038	----	----	----	----	----	----	----	----	----	----	----	49.000	0.417
Q16842	ST3GAL2	SeqId_6281_51	Death	1	0.219	0.052	0.919	0.038	----	----	----	----	----	----	----	----	----	----	----	49.000	0.417
P16152	CBR1	SeqId_12381_26	Death	1	0.319	0.108	0.942	0.039	----	----	----	----	----	----	----	----	----	----	----	86.224	0.417
P15814	IGLL1	SeqId_6485_59	Death	7	0.697	0.495	0.982	0.039	0.728	0.498	1.064	0.101	0.650	0.340	1.243	0.250	0.805	0.316	14.975	138.592	0.417
Q9H6Z4	RANBP3	SeqId_14037_18	Death	1	0.223	0.053	0.935	0.040	----	----	----	----	----	----	----	----	----	----	----	49.000	0.417

Q155Q3	DIXDC1	SeqId_13441_30	Death	1	4.704	1.071	20.657	0.040	----	----	----	----	----	----	----	----	----	----	----	46.046	0.417
P04264	KRT1	SeqId_9931_20	Death	1	0.232	0.057	0.946	0.042	----	----	----	----	----	----	----	----	----	----	----	59.172	0.417
P34096	RNASE4	SeqId_5644_60	Death	1	1.491	1.012	2.196	0.043	----	----	----	----	----	----	----	----	----	----	----	759.691	0.417
Q92838	EDA	SeqId_2826_53	Death	2	2.561	1.028	6.382	0.043	----	----	----	----	----	----	----	----	----	----	----	70.411	0.417
Q4LDE5	SVEP1	SeqId_11178_21	Death	3	0.431	0.178	1.041	0.061	0.539	0.285	1.020	0.058	2.903	0.480	17.551	0.453	0.273	0.087	58.989	132.234	0.561
P05452	CLEC3B	SeqId_5701_81	Death	2	0.611	0.359	1.041	0.070	----	----	----	----	----	----	----	----	----	----	----	182.252	0.561
P61769	B2M	SeqId_3485_28	Death	1	0.304	0.084	1.102	0.070	----	----	----	----	----	----	----	----	----	----	----	72.250	0.561
Q9NP99	TREM1	SeqId_9266_1	Death	2	1.532	0.966	2.431	0.070	----	----	----	----	----	----	----	----	----	----	----	290.487	0.561
Q8TDY8	IGDCC4	SeqId_9793_145	Death	1	0.617	0.361	1.054	0.077	----	----	----	----	----	----	----	----	----	----	----	289.000	0.587
Q9HCB6	SPON1	SeqId_4297_62	Death	3	1.372	0.963	1.955	0.080	1.347	0.957	1.895	0.087	1.186	0.589	2.387	0.716	0.719	0.783	0.000	343.123	0.587
P19438	TNFRSF1	SeqId_2654_19	Death	1	0.262	0.056	1.233	0.090	----	----	----	----	----	----	----	----	----	----	----	50.083	0.587
Q9NX46	ADPRHL2	SeqId_17332_3	Death	1	0.323	0.087	1.194	0.090	----	----	----	----	----	----	----	----	----	----	----	70.302	0.587
P58335	ANTXR2	SeqId_15559_5	Death	1	0.558	0.284	1.098	0.091	----	----	----	----	----	----	----	----	----	----	----	228.766	0.587
Q9NR71	ASAH2	SeqId_3212_30	Death	1	1.252	0.964	1.626	0.092	----	----	----	----	----	----	----	----	----	----	----	1697.440	0.587
P52823	STC1	SeqId_4930_21	Death	2	0.422	0.154	1.161	0.095	----	----	----	----	----	----	----	----	----	----	----	49.809	0.587
Q15485	FCN2	SeqId_3313_21	Death	3	0.806	0.624	1.041	0.098	0.856	0.645	1.135	0.281	0.563	0.219	1.444	0.443	0.580	0.548	0.000	463.941	0.587
P11597	CETP	SeqId_7131_207	Death	2	0.851	0.703	1.032	0.101	----	----	----	----	----	----	----	----	----	----	----	2065.014	0.587
Q8IZP7	HS6ST3	SeqId_18896_23	Death	1	3.061	0.783	11.972	0.108	----	----	----	----	----	----	----	----	----	----	----	60.989	0.609
P10721	KIT	SeqId_2475_1	Death	4	0.595	0.307	1.156	0.126	0.536	0.267	1.077	0.080	1.245	0.221	7.006	0.827	0.459	0.234	29.631	108.137	0.689

Q8NBJ4	GOLM1	SeqId_17456_53	Death	4	0.796	0.589	1.076	0.137	0.702	0.555	0.888	0.003	0.512	0.320	0.820	0.108	0.175	0.120	48.654	773.163	0.698
Q7LFX5	CHST15	SeqId_4469_78	Death	3	1.569	0.863	2.851	0.139	1.908	1.210	3.007	0.005	3.252	0.475	22.284	0.442	0.574	0.098	56.922	250.144	0.698
Q9BX93	PLA2G12	SeqId_9380_2	Death	5	0.798	0.587	1.086	0.151	0.747	0.527	1.057	0.099	0.600	0.333	1.081	0.187	0.346	0.736	0.000	241.681	0.698
Q9P0K1	ADAM22	SeqId_7933_75	Death	2	1.813	0.805	4.085	0.151	----	----	----	----	----	----	----	----	----	----	----	200.324	0.698
Q9C005	DPY30	SeqId_13943_38	Death	1	0.336	0.075	1.505	0.154	----	----	----	----	----	----	----	----	----	----	----	59.172	0.698
O60258	FGF17	SeqId_3494_71	Death	1	2.797	0.680	11.507	0.154	----	----	----	----	----	----	----	----	----	----	----	57.327	0.698
O75056	SDC3	SeqId_16612_28	Death	1	1.552	0.848	2.841	0.154	----	----	----	----	----	----	----	----	----	----	----	363.929	0.698
Q6UXM1	LRIG3	SeqId_3322_52	Death	2	0.461	0.156	1.358	0.160	----	----	----	----	----	----	----	----	----	----	----	88.648	0.698
Q9HAV5	EDA2R	SeqId_3083_71	Death	2	0.553	0.237	1.291	0.171	----	----	----	----	----	----	----	----	----	----	----	88.160	0.698
P08319	ADH4	SeqId_8325_37	Death	2	2.048	0.730	5.743	0.173	----	----	----	----	----	----	----	----	----	----	----	60.494	0.698
P00747	PLG	SeqId_3710_49	Death	3	0.703	0.423	1.169	0.174	0.747	0.436	1.277	0.286	0.412	0.128	1.321	0.376	0.500	0.582	0.000	168.204	0.698
P98172	EFNB1	SeqId_13104_32	Death	1	0.518	0.197	1.360	0.182	----	----	----	----	----	----	----	----	----	----	----	131.484	0.698
O00244	ATOX1	SeqId_19233_75	Death	2	0.416	0.114	1.519	0.184	----	----	----	----	----	----	----	----	----	----	----	51.201	0.698
Q99988	GDF15	SeqId_4374_45	Death	1	1.317	0.876	1.982	0.186	----	----	----	----	----	----	----	----	----	----	----	792.020	0.698
Q9BXJ1	C1QTNF1	SeqId_6304_8	Death	6	0.872	0.711	1.069	0.187	0.801	0.636	1.008	0.058	0.725	0.509	1.031	0.148	0.276	0.583	0.000	510.182	0.698
Q9BQI0	AIF1L	SeqId_18871_24	Death	2	0.908	0.786	1.048	0.187	----	----	----	----	----	----	----	----	----	----	----	4286.383	0.698
O14786	NRP1	SeqId_5542_22	Death	4	1.256	0.887	1.779	0.199	1.345	0.915	1.975	0.131	2.010	0.878	4.603	0.240	0.345	0.453	0.000	213.623	0.698
Q92626	PXDN	SeqId_13463_1	Death	1	0.533	0.203	1.397	0.200	----	----	----	----	----	----	----	----	----	----	----	115.976	0.698
Q9HD15	SRA1	SeqId_18220_141	Death	2	1.466	0.815	2.637	0.202	----	----	----	----	----	----	----	----	----	----	----	184.180	0.698

Q12805	EFEMP1	SeqId_8480_29	Death	2	0.587	0.258	1.334	0.204	----	----	----	----	----	----	----	----	----	----	----	196.312	0.698
Q9HC57	WFDC1	SeqId_9316_67	Death	5	0.842	0.643	1.102	0.210	0.883	0.670	1.163	0.376	1.029	0.659	1.608	0.907	0.353	0.385	3.808	300.185	0.698
Q9C075	KRT23	SeqId_14237_1	Death	2	0.811	0.584	1.127	0.213	----	----	----	----	----	----	----	----	----	----	----	703.590	0.698
P0DJI8	SAA1	SeqId_15515_2	Death	3	0.843	0.643	1.105	0.216	0.857	0.650	1.128	0.270	0.963	0.638	1.455	0.888	0.553	0.664	0.000	406.403	0.698
P14555	PLA2G2A	SeqId_2692_74	Death	1	0.878	0.713	1.080	0.217	----	----	----	----	----	----	----	----	----	----	----	4830.250	0.698
O14594	NCAN	SeqId_15573_110	Death	2	1.723	0.725	4.092	0.218	----	----	----	----	----	----	----	----	----	----	----	102.353	0.698
P17900	GM2A	SeqId_15441_6	Death	2	0.760	0.484	1.192	0.232	----	----	----	----	----	----	----	----	----	----	----	305.541	0.722
O60911	CTSV	SeqId_3364_76	Death	3	1.503	0.765	2.952	0.237	1.553	0.820	2.941	0.176	0.379	0.060	2.399	0.490	0.367	0.253	27.141	111.515	0.722
Q16270	IGFBP7	SeqId_3320_49	Death	1	1.267	0.855	1.876	0.238	----	----	----	----	----	----	----	----	----	----	----	722.266	0.722
Q8WUE5	CT55	SeqId_9363_11	Death	1	1.973	0.631	6.167	0.242	----	----	----	----	----	----	----	----	----	----	----	92.985	0.722
Q99784	OLFM1	SeqId_5703_26	Death	2	1.664	0.695	3.984	0.253	----	----	----	----	----	----	----	----	----	----	----	86.950	0.722
P00533	EGFR	SeqId_2677_1	Death	2	1.685	0.685	4.145	0.256	----	----	----	----	----	----	----	----	----	----	----	57.822	0.722
Q2UY09	COL28A1	SeqId_10702_1	Death	1	0.496	0.146	1.692	0.263	----	----	----	----	----	----	----	----	----	----	----	75.939	0.722
Q9H4D0	CLSTN2	SeqId_18882_7	Death	3	0.731	0.421	1.270	0.266	0.591	0.352	0.994	0.047	0.497	0.131	1.888	0.492	0.633	0.227	32.530	171.914	0.722
P07108	DBI	SeqId_16919_1	Death	1	0.755	0.459	1.241	0.267	----	----	----	----	----	----	----	----	----	----	----	560.111	0.722
P02765	AHSG	SeqId_3581_53	Death	2	2.593	0.481	13.967	0.267	----	----	----	----	----	----	----	----	----	----	----	46.812	0.722
Q06141	REG3A	SeqId_15304_1	Death	5	1.426	0.758	2.685	0.271	1.092	0.633	1.883	0.752	3.734	0.316	44.125	0.373	0.486	0.026	63.661	130.719	0.722
O75475	PSIP1	SeqId_17176_13	Death	1	2.314	0.512	10.458	0.276	----	----	----	----	----	----	----	----	----	----	----	62.775	0.722
P51858	HDGF	SeqId_8953_47	Death	1	2.584	0.469	14.252	0.276	----	----	----	----	----	----	----	----	----	----	----	49.000	0.722

P07949	RET	SeqId_3220_40	Death	1	1.221	0.849	1.758	0.282	----	----	----	----	----	----	----	----	----	----	----	965.775	0.722
P42830	CXCL5	SeqId_2979_8	Death	5	1.142	0.895	1.457	0.285	1.157	0.894	1.497	0.268	1.221	0.824	1.807	0.393	0.701	0.982	0.000	339.653	0.722
O94856	NFASC	SeqId_7179_69	Death	1	1.164	0.881	1.539	0.286	----	----	----	----	----	----	----	----	----	----	----	1195.184	0.722
P51858	HDGF	SeqId_16758_96	Death	1	2.157	0.514	9.057	0.294	----	----	----	----	----	----	----	----	----	----	----	71.598	0.725
Q02487	DSC2	SeqId_13126_52	Death	1	1.401	0.743	2.643	0.297	----	----	----	----	----	----	----	----	----	----	----	240.250	0.725
P22223	CDH3	SeqId_2643_57	Death	4	1.220	0.838	1.777	0.299	1.169	0.818	1.669	0.392	1.285	0.416	3.973	0.706	0.931	0.246	27.623	269.197	0.725
Q01995	TAGLN	SeqId_15640_54	Death	1	1.934	0.549	6.814	0.305	----	----	----	----	----	----	----	----	----	----	----	59.381	0.725
Q8WXI7	MUC16	SeqId_15565_102	Death	4	0.818	0.556	1.204	0.309	0.891	0.572	1.388	0.608	1.397	0.498	3.920	0.590	0.387	0.748	0.000	174.635	0.725
P0DJI9	SAA2	SeqId_18832_65	Death	2	0.852	0.625	1.161	0.310	----	----	----	----	----	----	----	----	----	----	----	455.969	0.725
Q86Z14	KLB	SeqId_19557_3	Death	1	0.888	0.702	1.123	0.321	----	----	----	----	----	----	----	----	----	----	----	2601.000	0.743
Q92743	HTRA1	SeqId_15594_47	Death	1	0.618	0.232	1.648	0.337	----	----	----	----	----	----	----	----	----	----	----	111.420	0.770
P05161	ISG15	SeqId_14151_4	Death	2	0.896	0.714	1.125	0.344	----	----	----	----	----	----	----	----	----	----	----	655.238	0.774
Q9Y5P4	COL4A3B	SeqId_13950_9	Death	2	1.403	0.693	2.840	0.347	----	----	----	----	----	----	----	----	----	----	----	126.195	0.774
P35858	IGFALS	SeqId_6605_17	Death	2	0.692	0.317	1.514	0.357	----	----	----	----	----	----	----	----	----	----	----	211.714	0.788
Q99729	HNRNPAB	SeqId_8894_80	Death	2	1.462	0.638	3.350	0.370	----	----	----	----	----	----	----	----	----	----	----	105.211	0.798
Q9UJJ9	GNPTG	SeqId_10666_7	Death	1	0.378	0.044	3.224	0.374	----	----	----	----	----	----	----	----	----	----	----	20.250	0.798
Q86VZ4	LRP11	SeqId_6713_4	Death	2	1.072	0.919	1.251	0.378	----	----	----	----	----	----	----	----	----	----	----	3199.686	0.798
P0C0P6	NPS	SeqId_6390_18	Death	1	0.601	0.193	1.875	0.380	----	----	----	----	----	----	----	----	----	----	----	77.440	0.798
Q86Y30	BAGE2	SeqId_6294_11	Death	2	0.532	0.128	2.217	0.386	----	----	----	----	----	----	----	----	----	----	----	48.476	0.798

Q8IZS8	CACNA2D	SeqId_8885_6	Death	3	0.653	0.248	1.722	0.389	0.645	0.339	1.227	0.182	2.243	0.021	235.672	0.791	0.686	0.019	74.868	166.301	0.798
Q9H3U7	SMOC2	SeqId_15635_4	Death	3	0.799	0.478	1.336	0.392	0.778	0.490	1.235	0.287	1.130	0.143	8.906	0.926	0.786	0.181	41.414	256.815	0.798
O95445	APOM	SeqId_10445_20	Death	3	1.369	0.664	2.822	0.395	1.297	0.722	2.328	0.384	6.814	1.178	39.428	0.278	0.311	0.163	44.914	116.209	0.798
P67809	YBX1	SeqId_9751_72	Death	3	1.187	0.790	1.785	0.410	1.122	0.787	1.599	0.525	0.510	0.173	1.501	0.436	0.353	0.235	30.973	343.052	0.819
O75462	CRLF1	SeqId_14747_9	Death	1	0.753	0.377	1.504	0.422	----	----	----	----	----	----	----	----	----	----	----	230.325	0.833
P01011	SERPINA	SeqId_4153_11	Death	2	0.863	0.600	1.240	0.425	----	----	----	----	----	----	----	----	----	----	----	478.382	0.833
P07998	RNASE1	SeqId_7211_2	Death	1	1.320	0.659	2.647	0.434	----	----	----	----	----	----	----	----	----	----	----	233.653	0.841
P11686	SFTPC	SeqId_5738_25	Death	5	0.898	0.681	1.185	0.448	0.904	0.653	1.252	0.544	0.948	0.502	1.790	0.880	0.864	0.872	0.000	298.269	0.861
P20333	TNFRSF1	SeqId_8368_102	Death	2	0.692	0.264	1.811	0.453	----	----	----	----	----	----	----	----	----	----	----	78.775	0.862
O60939	SCN2B	SeqId_8353_15	Death	2	1.336	0.617	2.891	0.462	----	----	----	----	----	----	----	----	----	----	----	89.351	0.862
P61626	LYZ	SeqId_4920_10	Death	2	0.842	0.529	1.339	0.467	----	----	----	----	----	----	----	----	----	----	----	1310.804	0.862
P14649	MYL6B	SeqId_14227_21	Death	2	1.479	0.515	4.241	0.467	----	----	----	----	----	----	----	----	----	----	----	50.642	0.862
A6NI73	LILRA5	SeqId_7787_25	Death	5	1.162	0.759	1.780	0.490	1.403	0.979	2.010	0.065	1.737	0.711	4.244	0.312	0.389	0.067	54.438	295.850	0.896
P52848	NDST1	SeqId_6927_7	Death	4	1.324	0.583	3.009	0.502	1.523	0.819	2.831	0.183	24.021	2.836	203.429	0.100	0.112	0.046	62.483	114.817	0.910
O00622	CYR61	SeqId_6264_9	Death	2	0.621	0.151	2.549	0.508	----	----	----	----	----	----	----	----	----	----	----	62.839	0.911
P49773	HINT1	SeqId_5900_11	Death	3	0.691	0.223	2.140	0.522	1.068	0.333	3.428	0.912	1.115	0.000	132948171.845	0.993	0.968	0.148	47.582	57.693	0.928
P35442	THBS2	SeqId_3339_33	Death	1	1.096	0.822	1.462	0.532	----	----	----	----	----	----	----	----	----	----	----	1413.760	0.938
P01034	CST3	SeqId_2609_59	Death	2	0.885	0.598	1.311	0.543	----	----	----	----	----	----	----	----	----	----	----	383.620	0.947
O14793	GDF11	SeqId_2765_4	Death	1	1.269	0.560	2.877	0.568	----	----	----	----	----	----	----	----	----	----	----	179.148	0.947

O95390	GDF11	SeqId_2765_4	Death	1	1.269	0.560	2.877	0.568	----	----	----	----	----	----	----	----	----	----	----	179.148	0.947
Q01638	IL1RL1	SeqId_4234_8	Death	4	0.942	0.766	1.160	0.574	0.915	0.763	1.097	0.337	0.756	0.571	1.001	0.190	0.189	0.232	30.017	1467.106	0.947
P59901	LILRA4	SeqId_8299_66	Death	4	0.847	0.474	1.516	0.577	0.999	0.715	1.397	0.996	1.788	0.964	3.317	0.206	0.109	0.007	75.352	353.578	0.947
P02671	FGA FGB	SeqId_4907_56	Death	1	1.312	0.501	3.440	0.580	----	----	----	----	----	----	----	----	----	----	----	123.602	0.947
P02675	FGA FGB	SeqId_4907_56	Death	1	1.312	0.501	3.440	0.580	----	----	----	----	----	----	----	----	----	----	----	123.602	0.947
P02679	FGA FGB	SeqId_4907_56	Death	1	1.312	0.501	3.440	0.580	----	----	----	----	----	----	----	----	----	----	----	123.602	0.947
P02679	FGG	SeqId_4989_7	Death	1	1.324	0.489	3.582	0.580	----	----	----	----	----	----	----	----	----	----	----	115.879	0.947
P02671	FGA FGB	SeqId_2796_62	Death	1	1.326	0.488	3.607	0.580	----	----	----	----	----	----	----	----	----	----	----	114.616	0.947
P02675	FGA FGB	SeqId_2796_62	Death	1	1.326	0.488	3.607	0.580	----	----	----	----	----	----	----	----	----	----	----	114.616	0.947
P02679	FGA FGB	SeqId_2796_62	Death	1	1.326	0.488	3.607	0.580	----	----	----	----	----	----	----	----	----	----	----	114.616	0.947
Q01974	ROR2	SeqId_7861_9	Death	3	0.769	0.302	1.958	0.582	0.966	0.461	2.024	0.927	8.688	0.535	141.023	0.370	0.330	0.143	48.574	87.292	0.947
P08697	SERPINF	SeqId_3024_18	Death	1	0.861	0.504	1.469	0.582	----	----	----	----	----	----	----	----	----	----	----	372.263	0.947
Q96EE4	CCDC126	SeqId_6388_21	Death	4	0.919	0.667	1.266	0.605	0.963	0.680	1.363	0.831	1.391	0.524	3.692	0.576	0.472	0.571	0.000	284.446	0.963
Q96GP6	SCARF2	SeqId_8956_96	Death	2	1.536	0.295	8.002	0.610	----	----	----	----	----	----	----	----	----	----	----	159.576	0.963
Q6P988	NOTUM	SeqId_8252_2	Death	2	0.852	0.458	1.583	0.612	----	----	----	----	----	----	----	----	----	----	----	100.984	0.963
Q8IUB2	WFDC3	SeqId_6384_19	Death	2	1.250	0.528	2.960	0.612	----	----	----	----	----	----	----	----	----	----	----	75.494	0.963
P41970	ELK3	SeqId_5707_55	Death	2	1.233	0.541	2.810	0.618	----	----	----	----	----	----	----	----	----	----	----	82.502	0.965
Q9NRM6	IL17RB	SeqId_6262_14	Death	2	1.231	0.535	2.834	0.625	----	----	----	----	----	----	----	----	----	----	----	80.240	0.967
P36776	LONP1	SeqId_6398_12	Death	3	1.211	0.556	2.637	0.631	1.135	0.485	2.655	0.771	1.331	0.010	178.952	0.928	0.976	0.919	0.000	66.041	0.969

Q07960	ARHGAP1	SeqId_11955_1	Death	2	0.828	0.371	1.849	0.646	----	----	----	----	----	----	----	----	----	----	----	83.856	0.984
Q86Y82	STX12	SeqId_10418_36	Death	1	0.787	0.277	2.237	0.653	----	----	----	----	----	----	----	----	----	----	----	104.040	0.987
P51965	UBE2E1	SeqId_14326_4	Death	3	1.118	0.675	1.852	0.663	1.049	0.687	1.603	0.823	0.720	0.174	2.978	0.729	0.625	0.217	34.614	245.287	0.992
O60704	TPST2	SeqId_8024_64	Death	4	0.925	0.645	1.325	0.669	0.891	0.591	1.343	0.581	0.499	0.218	1.142	0.241	0.247	0.379	2.669	231.381	0.992
P61956	SUMO2	SeqId_19555_1	Death	2	0.846	0.386	1.857	0.677	----	----	----	----	----	----	----	----	----	----	----	95.964	0.992
Q93091	RNASE6	SeqId_5646_20	Death	1	1.028	0.903	1.170	0.680	----	----	----	----	----	----	----	----	----	----	----	11274.579	0.992
P09529	FGA FGB	SeqId_13676_46	Death	2	0.878	0.467	1.649	0.685	----	----	----	----	----	----	----	----	----	----	----	345.890	0.992
O95633	FSTL3	SeqId_3438_10	Death	1	1.360	0.305	6.065	0.687	----	----	----	----	----	----	----	----	----	----	----	54.533	0.992
P19823	ITIH2	SeqId_9326_33	Death	1	1.042	0.844	1.286	0.703	----	----	----	----	----	----	----	----	----	----	----	2300.162	0.995
P01563	IFNA2	SeqId_3497_13	Death	1	1.230	0.416	3.640	0.709	----	----	----	----	----	----	----	----	----	----	----	90.751	0.995
P39060	COL18A1	SeqId_2201_17	Death	2	1.086	0.667	1.768	0.740	----	----	----	----	----	----	----	----	----	----	----	201.736	0.995
P03973	SLPI	SeqId_4413_3	Death	2	0.877	0.402	1.910	0.740	----	----	----	----	----	----	----	----	----	----	----	99.480	0.995
O95166	GABARAP	SeqId_17735_130	Death	1	0.840	0.282	2.504	0.754	----	----	----	----	----	----	----	----	----	----	----	114.325	0.995
Q9H0R8	GABARAP	SeqId_12661_44	Death	1	0.832	0.263	2.629	0.754	----	----	----	----	----	----	----	----	----	----	----	103.101	0.995
Q9H4F8	SMOC1	SeqId_13118_5	Death	2	1.068	0.708	1.609	0.754	----	----	----	----	----	----	----	----	----	----	----	302.606	0.995
Q13045	FLII	SeqId_12677_164	Death	2	0.872	0.355	2.140	0.765	----	----	----	----	----	----	----	----	----	----	----	78.554	0.995
Q9GZX9	TWSG1	SeqId_9234_8	Death	2	1.073	0.664	1.734	0.774	----	----	----	----	----	----	----	----	----	----	----	250.772	0.995
P03950	ANG	SeqId_4874_3	Death	1	1.068	0.681	1.675	0.774	----	----	----	----	----	----	----	----	----	----	----	974.332	0.995
O60760	HPGDS	SeqId_12549_33	Death	1	1.048	0.754	1.457	0.780	----	----	----	----	----	----	----	----	----	----	----	1068.787	0.995

P05413	FABP3	SeqId_5437_63	Death	1	1.290	0.215	7.730	0.781	----	----	----	----	----	----	----	----	----	----	----	49.000	0.995
P06276	BCHE	SeqId_15514_26	Death	5	1.032	0.822	1.294	0.788	1.017	0.806	1.283	0.885	0.991	0.711	1.382	0.963	0.769	0.902	0.000	491.471	0.995
Q9UHV9	PFDN2	SeqId_19243_2	Death	2	0.885	0.362	2.164	0.788	----	----	----	----	----	----	----	----	----	----	----	73.565	0.995
P16860	NPPB	SeqId_7655_11	Death	1	1.075	0.600	1.925	0.809	----	----	----	----	----	----	----	----	----	----	----	332.361	0.995
Q9Y274	ST3GAL6	SeqId_6947_4	Death	3	0.983	0.857	1.128	0.812	0.980	0.854	1.125	0.778	0.966	0.769	1.213	0.815	0.856	0.457	0.000	2204.445	0.995
P19827	ITIH1	SeqId_7955_195	Death	4	1.021	0.858	1.214	0.819	1.055	0.882	1.260	0.559	1.207	0.887	1.643	0.354	0.328	0.375	3.531	1108.034	0.995
Q9BUD6	SPON2	SeqId_8099_42	Death	1	1.054	0.652	1.705	0.831	----	----	----	----	----	----	----	----	----	----	----	554.059	0.995
Q12906	ILF3	SeqId_12759_47	Death	1	0.854	0.201	3.623	0.831	----	----	----	----	----	----	----	----	----	----	----	53.402	0.995
Q16627	CCL14	SeqId_2900_53	Death	2	1.020	0.850	1.224	0.832	----	----	----	----	----	----	----	----	----	----	----	1795.903	0.995
Q9NZ72	STMN3	SeqId_8019_73	Death	2	1.078	0.528	2.199	0.837	----	----	----	----	----	----	----	----	----	----	----	117.491	0.995
Q96DZ1	ERLEC1	SeqId_8957_72	Death	2	0.909	0.363	2.278	0.839	----	----	----	----	----	----	----	----	----	----	----	72.208	0.995
Q86TH1	ADAMTSL	SeqId_6379_62	Death	4	0.936	0.483	1.813	0.844	1.041	0.482	2.248	0.919	1.034	0.090	11.909	0.981	0.941	0.781	0.000	66.345	0.995
P49755	TMED10	SeqId_6506_54	Death	1	0.980	0.775	1.239	0.863	----	----	----	----	----	----	----	----	----	----	----	2097.640	0.995
Q76LX8	ADAMTS1	SeqId_3175_51	Death	1	0.976	0.737	1.293	0.866	----	----	----	----	----	----	----	----	----	----	----	1999.367	0.995
Q9Y3E7	VPS24	SeqId_12508_9	Death	2	1.089	0.398	2.981	0.869	----	----	----	----	----	----	----	----	----	----	----	60.740	0.995
Q93045	STMN2	SeqId_10900_272	Death	3	0.962	0.544	1.701	0.894	1.054	0.569	1.951	0.867	0.241	0.004	15.129	0.622	0.628	0.726	0.000	118.905	0.995
P02741	CRP	SeqId_4337_49	Death	5	0.975	0.640	1.488	0.908	0.968	0.575	1.630	0.903	0.450	0.067	3.027	0.472	0.474	0.667	0.000	116.166	0.995
P0C7M6	IQCF3	SeqId_13439_6	Death	2	1.074	0.300	3.851	0.913	----	----	----	----	----	----	----	----	----	----	----	155.612	0.995
Q99727	TIMP4	SeqId_6462_12	Death	2	1.025	0.632	1.661	0.921	----	----	----	----	----	----	----	----	----	----	----	443.766	0.995

P42167	TMPO	SeqId_8265_225	Death	1	1.070	0.257	4.452	0.926	----	----	----	----	----	----	----	----	----	----	----	59.172	0.995
Q9BUJ0	ABHD14A	SeqId_5715_4	Death	1	1.078	0.221	5.256	0.926	----	----	----	----	----	----	----	----	----	----	----	47.929	0.995
Q9NUQ9	FAM49B	SeqId_19176_27	Death	1	1.080	0.214	5.458	0.926	----	----	----	----	----	----	----	----	----	----	----	45.822	0.995
P09651	HNRNPA1	SeqId_12466_7	Death	1	1.052	0.363	3.048	0.926	----	----	----	----	----	----	----	----	----	----	----	124.694	0.995
Q9UNK0	STX8	SeqId_10903_50	Death	1	1.070	0.257	4.452	0.926	----	----	----	----	----	----	----	----	----	----	----	59.172	0.995
P30050	RPL12	SeqId_19183_164	Death	1	1.040	0.452	2.395	0.926	----	----	----	----	----	----	----	----	----	----	----	173.024	0.995
P29353	SHC1	SeqId_5272_55	Death	1	1.050	0.376	2.928	0.926	----	----	----	----	----	----	----	----	----	----	----	114.325	0.995
O95336	PGLS	SeqId_17799_9	Death	1	1.079	0.217	5.355	0.926	----	----	----	----	----	----	----	----	----	----	----	55.007	0.995
P60842	EIF4A1	SeqId_18829_4	Death	1	1.055	0.343	3.241	0.926	----	----	----	----	----	----	----	----	----	----	----	82.291	0.995
Q92804	TAF15	SeqId_16865_62	Death	1	1.072	0.247	4.663	0.926	----	----	----	----	----	----	----	----	----	----	----	55.675	0.995
Q96A72	MAGOHB	SeqId_16875_13	Death	1	1.076	0.229	5.070	0.926	----	----	----	----	----	----	----	----	----	----	----	50.083	0.995
Q99439	CNN2	SeqId_18877_15	Death	1	1.082	0.206	5.678	0.926	----	----	----	----	----	----	----	----	----	----	----	43.763	0.995
P22626	HNRNPA2	SeqId_5351_52	Death	1	1.076	0.232	4.982	0.926	----	----	----	----	----	----	----	----	----	----	----	60.063	0.995
O14737	PDCD5	SeqId_12517_52	Death	2	0.984	0.681	1.421	0.930	----	----	----	----	----	----	----	----	----	----	----	417.596	0.995
P02654	APOC1	SeqId_15364_101	Death	2	1.028	0.547	1.932	0.932	----	----	----	----	----	----	----	----	----	----	----	252.447	0.995
P12111	COL6A3	SeqId_11196_31	Death	1	1.057	0.284	3.943	0.934	----	----	----	----	----	----	----	----	----	----	----	82.291	0.995
O14907	TAX1BP3	SeqId_12498_12	Death	2	0.967	0.438	2.139	0.935	----	----	----	----	----	----	----	----	----	----	----	89.470	0.995
Q9BXJ4	C1QTNF3	SeqId_7251_64	Death	1	1.026	0.518	2.035	0.940	----	----	----	----	----	----	----	----	----	----	----	223.424	0.995
P18440	NAT1	SeqId_12632_14	Death	2	1.016	0.663	1.556	0.943	----	----	----	----	----	----	----	----	----	----	----	621.989	0.995

Q6WN34	CHRDL2	SeqId_6086_15	Death	1	0.969	0.349	2.690	0.951	----	----	----	----	----	----	----	----	----	----	----	115.976	0.995
P00797	REN	SeqId_3396_54	Death	1	0.958	0.210	4.362	0.955	----	----	----	----	----	----	----	----	----	----	----	51.235	0.995
Q96KN2	CNDP1	SeqId_5456_59	Death	4	1.006	0.772	1.311	0.963	0.912	0.663	1.257	0.575	0.751	0.404	1.395	0.460	0.413	0.445	0.000	382.565	0.995
Q6UWE3	CLPSL2	SeqId_7767_1	Death	2	0.979	0.373	2.570	0.966	----	----	----	----	----	----	----	----	----	----	----	276.064	0.995
P09211	GSTP1	SeqId_4911_49	Death	1	0.993	0.482	2.045	0.984	----	----	----	----	----	----	----	----	----	----	----	211.994	0.995
Q9Y3E2	BOLA1	SeqId_15370_5	Death	3	1.002	0.833	1.205	0.987	1.002	0.828	1.212	0.987	0.984	0.758	1.278	0.925	0.883	0.953	0.000	1551.769	0.995
Q8TDQ0	HAVCR2	SeqId_5134_52	Death	2	0.997	0.659	1.509	0.990	----	----	----	----	----	----	----	----	----	----	----	1272.541	0.995
O15123	ANGPT2	SeqId_2602_2	Death	1	1.008	0.220	4.632	0.991	----	----	----	----	----	----	----	----	----	----	----	54.128	0.995
Q4KMG0	CDON	SeqId_4541_49	Death	1	1.012	0.102	10.024	0.992	----	----	----	----	----	----	----	----	----	----	----	19.612	0.995
P16112	ACAN	SeqId_3280_49	Death	2	1.002	0.472	2.130	0.995	----	----	----	----	----	----	----	----	----	----	----	113.843	0.995

Table S15. Summary of results from Mendelian randomization analyses for the associations between genetically predited protein levels using pQTLs from the Fenland and deCODE cohorts and DHFA and death as outcomes

	cis-MR	cis+trans MR
**DHFA**		
deCODE	CCDC126, CD55, CCL14, NEGR1	CD55, CCL14, DPYSL3, EPHA4, ANTXR2, CHST15
Fenland	CCDC126, SVEP1, CD55, ADH7	EPHA4, CD55
**Death**		
deCODE	RSPO4, FCN2, IGLL1, HPGDS, FGF23	SVEP1, RSPO4, SPON1, FBLN5, LILRA5, CT55, EFNB1, PLA2G12, PCDHGA1, IQCF3, PSD2, FGF23, FABP3, WFDC2, CNTF
Fenland	EFEMP1, STC1, ATOX1, FCN2, SVEP1, ANG	PSD2, SET, SVEP1, RSPO4, FBLN5, PCDHGA1, BAMBI, TNNI3, STX3, OIT3, PCDHGA1, CRK, ST3GAL2, CBR1, IGLL1, RANBP3, DIXDC1, KRT1, RNASE4, EDA

Table S16. Results from genetic colocalization results between proteins as exposures and death and DHFA as outcomes

Exposure	Outcome	N SNPs	H0	H1	H2	H3	H4	H4/[H3+H4]
**Outcome: DHFA**								
CD55	DHFA	647	0.00%	28.60%	0.00%	1.58%	69.80%	97.79%
CCDC126	DHFA	728	0.00%	21.20%	0.00%	6.31%	72.50%	91.99%
SVEP1	DHFA	1061	0.00%	50.20%	0.00%	5.73%	44.10%	88.50%
RSPO4	DHFA	149	0.00%	75.60%	0.00%	0.52%	23.90%	97.86%
FGF23	DHFA	4	0.02%	76.60%	0.00%	0.01%	23.40%	99.97%
IGLL1	DHFA	115	0.00%	68.70%	0.00%	0.67%	30.60%	97.85%
CCL14	DHFA	189	0.00%	48.50%	0.00%	0.73%	50.80%	98.58%
FBLN5	DHFA	138	0.00%	79.10%	0.00%	0.42%	20.40%	97.97%
FCN2	DHFA	615	0.00%	35.30%	0.00%	1.17%	63.50%	98.19%
**Outcome: Death**								
CD55	Death	647	0.00%	55.87%	0.00%	2.10%	42.03%	95.24%
CCDC126	Death	728	0.00%	69.80%	0.00%	2.77%	27.40%	90.82%
SVEP1	Death	1061	0.00%	63.50%	0.00%	4.17%	32.30%	88.57%
RSPO4	Death	149	0.00%	61.78%	0.00%	0.76%	37.47%	98.02%
FGF23	Death	4	0.01%	40.50%	0.00%	0.01%	59.50%	99.98%
IGLL1	Death	115	0.00%	46.30%	0.00%	0.36%	53.30%	99.34%
CCL14	Death	189	0.00%	67.50%	0.00%	1.59%	30.90%	95.11%
FBLN5	Death	138	0.00%	69.50%	0.00%	0.65%	29.80%	97.86%
FCN2	Death	615	0.00%	50.60%	0.00%	1.72%	47.70%	96.52%

Figure S1. Summary of proteome-wide Mendelian randomization analyses for HF adverse outcomes death and DHFA.

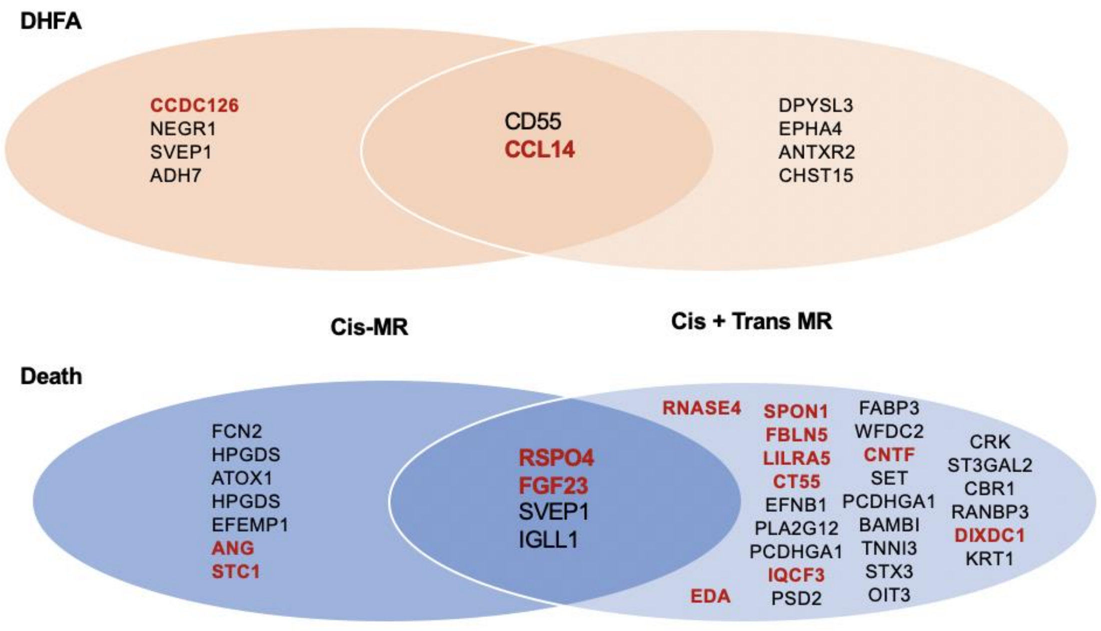

Proteins that have detrimental effects are bolded and shown in red.
